# Chiral arylsulfinylamides as reagents for visible light-mediated asymmetric alkene aminoarylations

**DOI:** 10.1038/s41557-023-01414-8

**Published:** 2024-01-16

**Authors:** Cédric Hervieu, Mariia S. Kirillova, Yawen Hu, Sergio Cuesta-Galisteo, Estíbaliz Merino, Cristina Nevado

**Affiliations:** 1 Department of Chemistry, University of Zurich, Zurich, Switzerland; 2 Universidad de Alcalá, Departamento de Química Orgánica y Química Inorgánica, Instituto de Investigación Andrés M. del Río (IQAR), Facultad de Farmacia, Madrid, Spain; 3 Instituto Ramón y Cajal de Investigación Sanitaria (IRYCIS), Madrid, Spain

**Keywords:** Synthetic chemistry methodology, Synthetic chemistry methodology, Photocatalysis, Photocatalysis

## Abstract

Two- or one-electron-mediated difunctionalizations of internal alkenes represent straightforward approaches to assemble molecular complexity by the simultaneous formation of two contiguous C*sp*^3^ stereocentres. Although racemic versions have been extensively explored, asymmetric variants, especially those involving open-shell C-centred radical species, are much more limited both in number and scope. Here we describe enantioenriched arylsulfinylamides as all-in-one reagents for the efficient asymmetric, intermolecular aminoarylation of alkenes. Under mild photoredox conditions, nitrogen addition of the arylsulfinylamide onto the double bond, followed by 1,4-translocation of the aromatic ring, produce, in a single operation, the corresponding aminoarylation adducts in enantiomerically enriched form. The sulfinyl group acts here as a traceless chiral auxiliary, as it is eliminated in situ under the mild reaction conditions. Optically pure β,β-diarylethylamines, aryl-α,β-ethylenediamines and α-aryl-β-aminoalcohols, prominent motifs in pharmaceuticals, bioactive natural products and ligands for transition metals, are thereby accessible with excellent levels of regio-, relative and absolute stereocontrol.

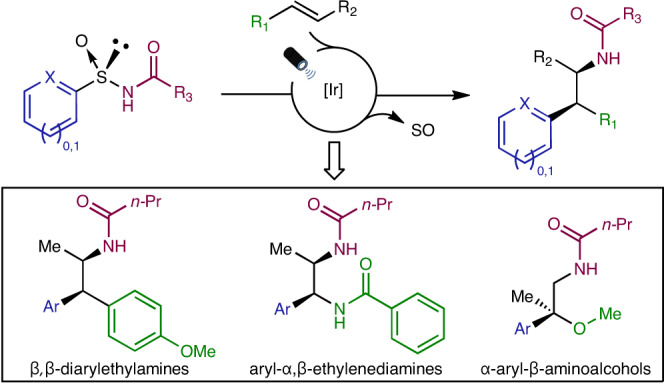

## Abstract (web summary)

Single-electron-mediated difunctionalizations of internal olefins allow the simultaneous formation of two contiguous C*sp*^3^-stereocentres. Here, we describe enantioenriched arylsulfinylamides as all-in-one reagents for the efficient asymmetric, intermolecular aminoarylation of alkenes. Mechanistic studies revealed an interesting dichotomy in the initiation of the reaction depending on the olefin redox potential.

## Main

**Fig. 1:**
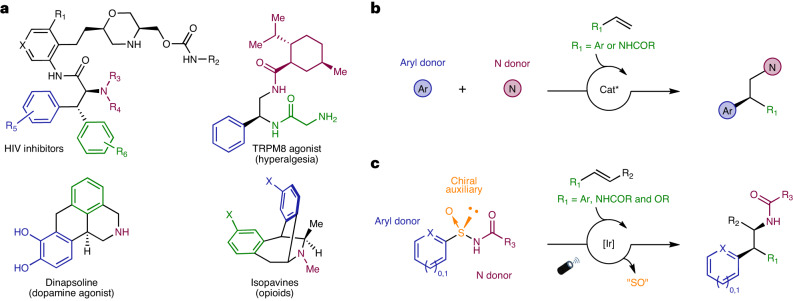
Relevance of β-arylethylamine motifs and strategies towards their asymmetric assembly. **a**, Examples of bioactive compounds featuring β-arylethylamines. TRPM8, transient receptor potential melastatin subtype 8. **b**, Previous examples of asymmetric three-component intermolecular alkene aminoarylations to attain β*-*arylethylamines^38,39^. Reactions are limited to the utilization of a single class of terminal olefins, either styrenes (*R*_1_ = Ar) or vinyl amides (*R*_1_ = NHCOR), and thus to the generation of a single stereogenic centre. *N*-fluoro-*N*-methylsulfonamides (NFAS) and *O*-acyl hydroxylmethylamine were used as N-atom donors, providing access exclusively to *N*-Me tertiary amine products. **c**, This work describes an asymmetric intermolecular alkene aminoarylation using arylsulfinylamides as multifunctional all-in-one reagents featuring a traceless chiral auxiliary. The reaction tolerates a wide variety of N-atom donors and is compatible with both 1,2-disubstituted styrenes, vinyl amides and vinyl ethers, thus providing access to valuable β,β-diarylethylamines, aryl-α,β-ethylenediamines and α-aryl-β-aminoalcohols. Excellent levels of both relative and absolute stereocontrol are achieved in the two newly forged stereogenic centres, governed by the configuration of the chiral sulfoxide tether. Characterization of the reaction mechanism revealed an interesting dichotomy in the initiation of the photoredox catalytic cycle wherein either electron-rich alkenes or sulfinylamides are preferentially activated at the expense of the Ir photocatalyst.

Nature’s secondary metabolites, as well as de novo-designed small-molecule probes, are substantially populated with nitrogen atoms. HIV inhibitors^1^, ion-channel modulators^2^, opioids^3,4^ and endogenous neurotransmitters^5^ (Fig. 1a) are representative examples of relevant bioactive compounds showcasing N-containing motifs, many of which feature amines substituted with an aromatic group in the β-position. Access to these prominent chemical blueprints in enantiomerically pure form is crucial, not only for accurate target engagement studies, but also for the optimization of their pharmacological profiles. A representative example is *R-*(+)-dinapsoline, a selective and efficient D_1_ dopamine agonist^5^, which has been found to be 161-fold more potent than its *S*-(−)-enantiomer.

The asymmetric synthesis of β-arylethylamines has typically relied on additions^6–10^ or hydrogenations of alkenes^11,12^, as well as on ring-opening^13–15^ and condensation reactions^16^. These processes require multistep sequences encompassing highly tailored reaction conditions such as cryogenic temperatures and/or high pressures as well as precise metal/ligand combinations. This represents a significant limitation and hampers their broad applicability. In sharp contrast, two-^17–20^ or one-electron-mediated^21–27^ multicomponent aminoarylation reactions featuring alkenes as highly abundant feedstocks represent a powerful, atom-economic alternative to access these ubiquitous motifs as two contiguous C*sp*^3^–C and C*sp*^3^–N bonds can be forged in a single operation^28–34^. Despite the intrinsic potential to impart both relative and absolute stereocontrol in the newly formed stereocentres^35^, asymmetric variants of these transformations, especially in intermolecular settings, are extremely scarce. Benzohydroxamic acid derivatives^18,36^ and *ortho*-iodoanilines^37^ have been showcased as non-cleavable C,N-tethered reagents to orchestrate asymmetric annulations with alkenes. Additionally, a handful of examples featuring three-component reactions have been reported (Fig. 1b). In 2017, the addition of *N*-fluoro-*N*-alkylsulfonamides (NFSA)-derived radicals and (hetero)arylboronic acids across the *π*-system in the presence of a chiral BOX-ligated copper catalyst to yield β,β-diarylethylamines with excellent levels of absolute stereocontrol was demonstrated^38^. More recently, an asymmetric Minisci reaction involving quinoline derivatives and *O*-acyl hydroxylmethylamine with *N*-vinylacetamide as a radical acceptor was reported^39^. Notwithstanding the undisputable synthetic utility of these transformations, limitations regarding both the type of N donors and the olefinic partners justify the quest for alternative, more flexible strategies in this context.

An elegant light-mediated intermolecular aminoarylation of alkenes using arylsulfonylacetamides as bifunctional reagents has been demonstrated^40–43^. The reaction, only applicable to electron-rich styrenes, furnished the corresponding β,β-diarylethylamines in racemic form^40^. Recently, our group exploited the ability of chiral *N*-sulfinyl moieties to impart absolute stereocontrol in the challenging assembly of all-C quaternary centres^44^.

Inspired by these results, we hypothesized that the addition of a nitrogen atom bound to a chiral arylsulfoxide group onto the terminal position of a 1,2-disubstituted alkene could control the absolute stereochemistry in the formation of the newly created C*sp*^3^–N bond as well as on the neighbouring C*sp*^3^–C*sp*^2^ centre generated upon a radical Truce–Smiles rearrangement of the corresponding aryl moiety. In this Article we describe enantioenriched arylsulfinylamides as multifunctional all-in-one reagents able to forge, regio- and stereoselectively, two contiguous C*sp*^3^–C and C*sp*^3^–N bonds across a variety of *π*-systems. A photochemically enabled addition of the nitrogen atom onto the terminal position of styrenes, vinyl amides and vinyl ethers furnishes a C-radical intermediate, which, upon translocation of the aromatic ring, delivers enantioenriched β,β-diarylethylamines, aryl-α,β-ethylenediamines and α-aryl-β-aminoalcohols, respectively. The mild reaction conditions and broad functional-group tolerance, combined with excellent regio-, diastereo- and enantioselectivity, highlight both the generality and synthetic utility of these transformations in the assembly of relevant blueprints populating pharmaceuticals, bioactive natural products and ligands for transition-metal catalysis.

## Results

### Reaction optimization

Enantiopure (*S*_S_)-*N*-(*p*-tolylsulfinyl)butyramide **1a** and *trans*-anethole were chosen as model substrates for our initial investigations. Reactions under blue light-emitting diode irradiation in the presence of different photocatalysts were performed, combining these two starting materials in a 1:1.2 ratio (experimental details are provided in Supplementary Table 1). Extensive screening revealed that, using 1 mol% of (Ir[dF(CF_3_)ppy]_2_(dtbpy))PF_6_ and 0.3 equiv. of potassium benzoate as the base in an isopropanol/trifluoroethanol/water mixture at ambient temperature, the desired β,β-diarylethylamine **2.1** could be produced in 53% yield with excellent diasteroselectivity (>20:1 d.r.) and promising 89:11 enantiomeric ratio (e.r.). Adjusting the stoichiometry between **1a** and the olefin to a 1:2 ratio and decreasing the reaction temperature to −20 °C furnished **2.1** in an improved 83% yield with almost perfect levels of both relative and absolute stereocontrol (>20:1 d.r.; >99:1 e.r.). Furthermore, the efficiency of stereochemical information transfer was maintained when the reaction was scaled up tenfold, affording **2.1** in 58% yield (>20:1 d.r. and 98:2 e.r.; experimental details are provided in Supplementary Fig. 4). Additional experiments in the presence of radical inhibitors or excluding the photocatalyst, the light or the base resulted in the recovery of both unreacted starting materials (experimental details are provided in Supplementary Table 2).

### Reaction scope

With the optimal conditions in hand, we set out to explore the compatibility of different N-atom donors and aryl migrating groups within the all-in-one arylsulfinylamide reagents. To this end, modifications on both the N-atom donor and the aryl migrating group were investigated. Alkyl amide derivatives featuring a diverse set of substitution patterns were also compatible with the standard reaction conditions, furnishing the corresponding adducts (CH_2_PMP, **2.2**; CHEtPh, **2.3**; Cy, **2.4**; (CH_2_)_2_CO_2_Et, **2.5**; CH_2_CH(OTBS)Me, **2.6**) with excellent levels of regio- and both relative and absolute stereocontrol (Table 1). Furthermore, successful incorporation of aromatic and heteroaromatic substituted amides and even the *tert*-butyl carbamate derivative (**2.7**–**2.9**) emphasizes the functional-group compatibility of the method. Furthermore, carbamate derivative (**2.9**) could also be transformed under the standard conditions, providing access to the corresponding free amine upon acid hydrolysis, with complete retention of the stereochemical information (Supplementary Information, compound **2.39**).

**Table 1:**
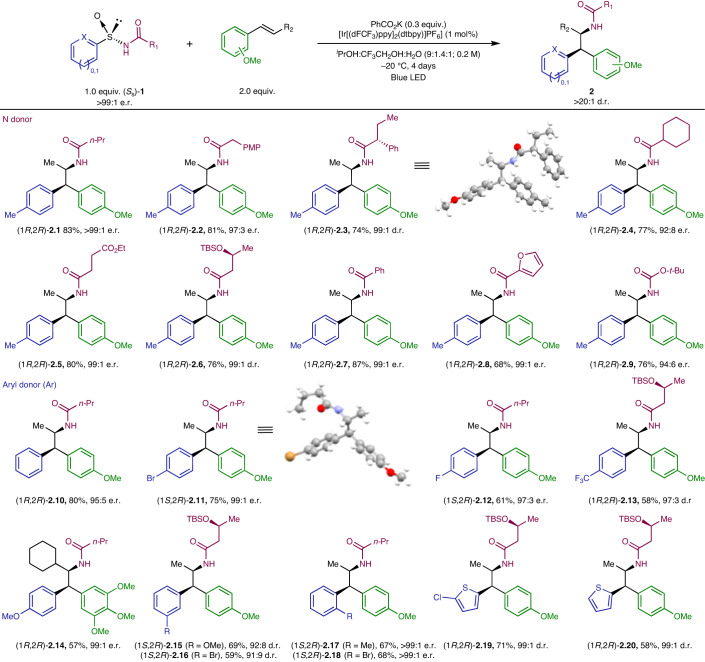
Arylsulfinylamide scope of the intermolecular aminoarylation of styrenes

Unless otherwise noted, reactions were carried out under the standard conditions. Full conversion of the starting material was observed, and yields are reported after purification by column chromatography in silica gel. All compounds were obtained with >20:1 d.r. The d.r. and e.r. values were determined by ^1^H NMR of the crude reaction mixture and by chiral stationary HPLC of the isolated products, respectively. *n*-Pr, *n*-propyl; PMP, *p*-methoxyphenyl; Ph, phenyl; TBS, *t*-butyldimethylsilyl; *t*-Bu, *t*-butyl.

The scope with respect to the migrating aromatic groups was investigated next. Transposition of a simple phenyl group proceeded smoothly under standard conditions to give **2.10** in high yield. Interestingly, substrates bearing both electron-withdrawing and electron-donating groups in the *para*-position of the arene proved to be suitable precursors, furnishing the corresponding β,β-diarylethylamines (**2.11**–**2.14**) in good yields with outstanding levels of stereocontrol. The *meta*-methoxy and *meta*-bromo derivatives also delivered the desired products (*m*-OMe, **2.15**; *m*-Br, **2.16**), although with slightly lower stereoinduction. In contrast, more sterically hindered substrates bearing *ortho*-substituted aromatic rings (*o*-Me, **2.17**; *o*-Br, **2.18**) were obtained with excellent enantioselectivities. To our delight, the *ortho*-bromo adduct **2.18** was quantitatively converted into the corresponding indoline in the presence of the Pd catalyst with retention of configuration, highlighting the synthetic potential of the obtained aminoarylation products (Supplementary Information, compound **2.40**). Moreover, heteroaryl migration also took place under the standard conditions, furnishing the corresponding thiophene derivatives **2.19** and **2.20** in good yields, with excellent levels of both relative and absolute stereocontrol.

X-ray crystallographic analysis of compounds **2.3** and **2.11** confirmed the *syn* addition of the N atom and the arene across the *π*-system. Adduct **2.11**, stemming from an (*S*_S_)-arylsulfinylamide precursor containing a Br atom, enabled us to assign the absolute configuration of the major diastereoisomer produced in this reaction as (1*S*,2*R*). It is important to note that the substitution pattern in the aromatic ring affects the priority of the groups at the new asymmetric carbon atom. As a result, a (1*R*,2*R*) configuration can be assigned to most of the obtained compounds. The reaction proved to be stereospecific: when the (*R*)-enantiomer of the arylsulfinylamide (*R*_S_)-**1a′** was used as a precursor, the opposite enantiomer of the β,β-diarylethylamine product (1*S*,2*S*)-**2.1′** could be obtained in similar yield and e.r. (experimental details are provided in Supplementary Fig. 28).

The compatibility of the reaction between (*S*_S_)-*N*-(*p*-tolylsulfinyl)butyramide **1a** with different styrene partners was also explored (Table 2). Although simple styrenes (*R*^3^ = H) were not competent substrates, phenethyl, cyclohexyl, 4-tetrahydropyranyl and carbinyl acetate groups at the terminal position of the double bond were effectively accommodated in the aminoarylation process. The corresponding β,β-diarylethylamines (**2.21**–**2.25**) were obtained in moderate to good yields with high enantioselectivity. Moreover, a chiral *para*-methoxy styrene derived from (*R*)-citronellal provided the corresponding aminoarylation adduct **2.26** in moderate yield but with excellent levels of regio- and both relative and absolute stereocontrol.

**Table 2:**
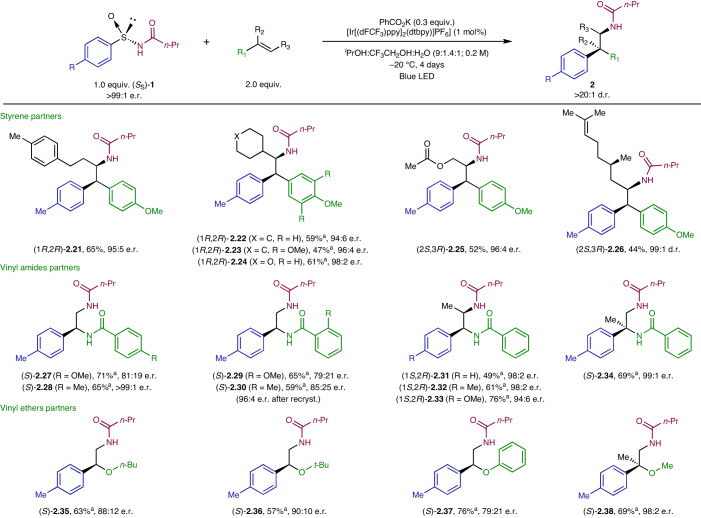
Scope of the alkene partner for the intermolecular aminoarylation with arylsulfinylamide (*S*_S_)-**1**

Unless otherwise noted, reactions were carried out under the standard conditions. Full conversion of the starting material was observed, and yields are reported after purification by column chromatography in silica gel. All compounds were obtained with >20:1 d.r. The d.r. and e.r. values were determined by ^1^H NMR of the crude reaction mixture and by chiral stationary HPLC of the isolated products, respectively.

^a^5 mol% of [Ir[(dFCF_3_)ppy]_2_(dtbpy)]PF_6_] at 0 °C. *n*-Bu, *n*-butyl.

To further expand the scope of this multicomponent radical cascade, different electron-rich olefins were surveyed. To our delight, aromatic vinyl amides turned out to be suitable partners, providing efficient access to aryl-α,β-ethylenediamines. These motifs are not only present in biologically active compounds^2,45^, but have also been prominently used as bidentate ligands in transition-metal complexes^46,47^. Thus, α,β-diamine derivatives (*p*-OMe, **2.27**; *p*-Me, **2.28**; *o*-OMe, **2.29**; *o***-**Me, **2.30**) were accessed in good yields and with moderate to excellent enantioselectivities. Furthermore, α- and β-methyl-substituted vinyl benzamides were efficiently transformed into the corresponding adducts (β-Me, **2.31**–**2.33**; α-Me, **2.34**) featuring an even more sterically demanding quaternary stereocentre with perfect stereochemical information transfer. Finally, vinyl ethers could be successfully engaged in the reaction, providing access to protected α-aryl-β-aminoalcohols (**2.35**–**2.38**) in good yields with moderate to excellent enantioselectivities.

### Mechanistic investigations

**Fig. 2:**
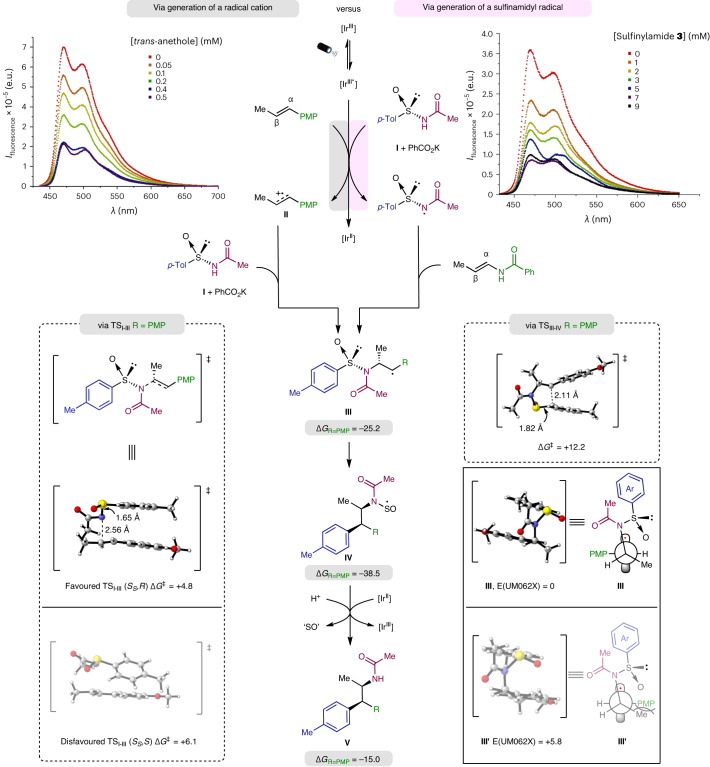
Mechanistic studies and proposed reaction mechanism. Results of Stern−Volmer experiments using *trans*-anethole (top left) and arylsulfinylamide **3** (top right) as quenchers (I, intensity, e.u., arbitrary energy units). The proposed reaction mechanism is shown, featuring two different initiation pathways: formation of a radical cation for electron-rich olefins (grey) and formation of an amidyl radical in the case of vinylamide acceptors (pink). DFT calculations were performed on *trans*-anethole as a benchmark substrate. Optimized transition states, relevant structural parameters, starting materials, products, reaction intermediates and transition states were computed at the M062X/6-31+G(d,p) level (IEFPCM, integral equation formalism with the polarizable continuum model), with the solvent 2-propanol at −20 °C (R = PMP). Energies are given in units of kcal mol^−1^. For further details, see Supplementary section ‘DFT calculations’. **TS**_**I–III**_ (*S*_S_,*R*) visualizes the enantiodetermining step in which **I** adds to the alkene radical cation **II** in line with the absolute configuration observed in the aminoarylation products. The alternative **TS**_**I–III**_ (*S*_S_,*S*), is 1.3 kcal mol^−1^ higher in energy than **TS**_**I–III**_ (*S*_S_,*R*). Conformations were calculated for intermediates **III** and **III′**. The conformer yielding the minor isomer is disfavoured by steric factors: the PMP group adopts an unfavourable *syn*-periplanar disposition with the methyl group, unlike the case of the major diastereomer experimentally obtained, in which these groups exhibit a less sterically demanding *anti*-periplanar geometry. PMP, *p*-methoxyphenyl, *p*-Tol, *p*-methylphenyl.

Having demonstrated the synthetic utility of this methodology, we focused our investigations on the underlying reaction mechanism. First, Stern–Volmer fluorescence quenching studies were performed to shed light on the potential species activated by the photocatalyst at the outset of the reaction^48^. The experiments were conducted using [Ir[(dFCF_3_)ppy]_2_(dtbpy)]PF_6_ excited with light (430 nm) in the presence of the different reactants. In the case of *trans*-anethole, a decrease in fluorescence intensity was observed as a function of olefin concentration (Fig. 2, top left). In sharp contrast, (*E*)-*N*-(prop-1-en-1-yl)benzamide did not quench the excited photocatalyst, even at high concentrations (Supplementary Figs. 7–12). Cyclic voltammetry of this vinylamide (*E*_1/2_ = +1.45 V versus saturated calomel electrode (SCE) in MeCN) confirmed the mismatched redox potential with respect to that of the photocatalyst (*E*_1/2_ = +1.26 V versus SCE in MeCN) (Supplementary Fig. 25)^40^. Interestingly, fluorescence quenching was not observed at low concentrations of arylsulfinylamide **1a** and potassium benzoate. Increasing the concentration of either arylsulfinylamide **1a** or both **1a** and base (Supplementary Figs. 13–18) led to oxidation of the reagent^49^. A more soluble tetrabutylammonium-conjugated sulfinylamide salt **3** proved to be an efficient quencher of the iridium photocatalyst (Fig. 2, top right), in line with the reduction potential measured by cyclic voltammetry (*E*_1/2_ = +0.57 V versus SCE in MeCN) (Supplementary Fig. 26).

These results indicate that different mechanisms might be operating at the outset of the reaction, depending on the olefinic partner. In the case of electron-rich styrenes, the formation of a radical cation via single-electron oxidation can be confidently proposed as the initial step of the photocatalytic cycle. In contrast, single-electron oxidation of the deprotonated arylsulfinylamide by the excited Ir photocatalyst to form an N-centred radical seems to be a more likely first step in the case of poorly oxidizable olefins.

To gain additional insights into the stereochemical outcome of these transformations, several control experiments as well as density functional theory (DFT) calculations were performed using anethole derivatives as benchmark substrates. First, the standard reaction conditions were applied in three independent experiments featuring *cis*-, *trans*- and a 1:1 mixture of *cis*- and *trans*-anethole. The formation of the corresponding products was analysed by ^1^H NMR (experimental details are provided in Supplementary Fig. 29). In all three cases, the aminoarylation adduct **2.1** was obtained in comparable yields, with almost identical d.r. and e.r. values. Next, and this time in the absence of arylsulfinylamide **1a**, *cis*- and *trans*-anethole were separately subjected to the standard reaction conditions and their potential isomerization^50^ was monitored by ^1^H NMR. A plot of temporal concentration versus time revealed that, after only 10 min, both isomers converge to an ~1.7:1 *cis*-to-*trans* ratio (Supplementary Figs. 5 and 6). Such a photostationary state, reached in a much faster regime than the aminoarylation reaction itself, suggests that both isomers will be present at the outset of the reaction, regardless of the initial alkene geometry. Following olefin oxidation to the corresponding radical cation **II**, addition of the arylsulfinylamide **I** will proceed at the β-carbon atom so that the absolute configuration of the first stereogenic centre is thus defined by that of the chiral sulfinyl moiety. DFT calculations revealed a low-energy transition state **TS**_**I–III**_ (*S*_S_,*R*) (Δ*G*^ǂ^ = +4.8 kcal mol^−1^) for this step, which delivers the benzylic radical **III** in a net exothermic process (Δ*G* = −25.2 kcal mol^−1^). Intermediate **III** undergoes a 1,4-aryl shift. No radical Meisenheimer intermediate could be located along the reaction energy profile^51^. Instead, a spirocyclic transition state **TS**_**III–IV**_ was found to precede the exothermic formation of SO-centred radical **IV** (Δ*G* = −38.5 kcal mol^−1^). **TS**_**III–IV**_ can be considered an early transition state in which the new C–C bond between the benzylic radical and the migrating aromatic group is only marginally formed (*d*_Cbn−C*sp*2_ = 2.11 Å in **TS**_**III–IV**_ versus *d*_Cbn−C*sp*2_ = 1.52 Å in **IV**), and the S(O)–C bond is barely elongated (*d*_S–C*sp*2_ = 1.82 Å in **TS**_**III–IV**_ versus d_S–C*sp*2_ = 1.80 Å in **III**). Formation of the minor diastereoisomer can be traced back to the generation of intermediate **III′** before the aryl transposition. Conformational analysis of the two intermediates suggests that the aryl translocation preferentially takes place through a trajectory in which the steric interactions between the PMP group and the methyl substituent within the anethole are minimized (ΔΔ*G*_**III/III′**_ = +5.8 kcal mol^−1^). DFT calculations support the notion of the aryl migration being the rate-determining step (**TS**_**III–IV**_, Δ*G*^ǂ^ = +12.2 kcal mol^−1^). As a result, and regardless of any potential kinetic preference for the formation and/or subsequent reactivity of either a *Z*- or an *E*-anethole-derived radical cation, the fast interconversion of **III′** into **III** by rotation along the C_α_–C_β_ bond supports the *syn* relative configuration observed in the aminoarylation products (additional details are provided in Supplementary Fig. 30). The photocatalytic cycle is closed thereafter by oxidation of **IV** to **V** by Ir(II) to recover the Ir(III) catalyst. The precise fate of the sulfur-based chiral linker is challenging to assess. However, having detected the bisulfite (HSO_3_^−^) anion using commercially available colorimetric test strips, we can confirm that sulfur(IV) species account at least in part for the SO lost^52^ (additional details are provided in Supplementary Fig. 27). Additionally, Fig. 2 shows **TS**_**I–III**_ (*S*_S_,*S*), the alternative transition state for the enantiodetermining step, in which **I** adds to the alkene radical cation **II**. **TS**_**I–III**_ (*S*_S_,*S*) is 1.3 kcal mol^−1^ higher in energy than **TS**_**I–III**_ (*S*_S_,*R*), which rationalizes the absolute configuration observed in the aminoarylation products.

## Conclusion

Here we have described an asymmetric intermolecular aminoarylation of alkenes. A photoredox-mediated radical cascade capitalizes on a chiral all-in-one arylsulfinylamide reagent featuring a traceless chiral auxiliary to forge two vicinal C*sp*^3^–C*sp*^2^ and C*sp*^3^–N bonds across the π-system in a stereocontrolled manner. Mechanistic investigations revealed the likelihood of multiple reaction pathways operating in these transformations. In the case of electron-rich styrenes, the formation of a radical cation via single-electron oxidation can be confidently proposed at the outset of the reaction. In contrast, the single-electron oxidation of the deprotonated arylsulfinylamide by the excited Ir photocatalyst to form an N-centred radical seems favoured in the case of poorly oxidizable olefins. The C–N bond formation is stereocontrolled by the chirality of the sulfoxide, whereas the subsequent transposition of the aromatic ring with concomitant elimination of the sulfinyl tether proceeds in a highly diastereoselective manner governed by steric factors. The β,β-diarylethylamines, aryl-α,β-ethylenediamines and α-aryl-β-aminoalcohols, ubiquitous motifs in bioactive molecules as well as in bidentate transition-metal ligands, are obtained herein with very high levels of regio- and both relative and absolute stereocontrol, thus highlighting the synthetic utility of this methodology.

## Methods

To an oven-dried Schlenk tube (5 ml), the corresponding arylsulfinylamide (0.1 mmol, 1 equiv.), PhCO_2_K (4.8 mg, 0.03 mmol, 0.3 equiv.) and [Ir[(dFCF_3_)ppy]_2_(dtbpy)]PF_6_ (1.1 mg, 0.001 mmol, 1 mol%) were sequentially added under a flow of nitrogen. The flask was evacuated and then backfilled with N_2_ (three times). Trifluoroethanol (72 μl) and *i*-PrOH:H_2_O (0.5 ml, 9:1 (vol:vol)) were then added to the reaction mixture, followed by the olefin (0.2 mmol, 2.0 equiv.). The reaction was sparged with argon for 15 min. The Schlenk tube was placed in the photoreactor and stirred at 1,400 r.p.m. under blue-light irradiation (EvoluChem 30W, HCK1012-01-008) at −20 °C. After four days, the reaction mixture was diluted with ethyl acetate (10 ml) and transferred into a separatory funnel. The mixture was washed with a 5 wt% aqueous LiCl solution (3 × 10 ml). The organic phases were dried over MgSO_4_, filtered, and concentrated in vacuo. The residue was purified by flash chromatography using a mixture of ethyl acetate and *n*-hexane.

## Online content

Any methods, additional references, Nature Portfolio reporting summaries, source data, extended data, supplementary information, acknowledgements, peer review information; details of author contributions and competing interests; and statements of data and code availability are available at 10.1038/s41557-023-01414-8.

### Supplementary information

Supplementary Information General information, reaction optimization data, experimental procedures, additional experiments, compound characterization including spectroscopic and analytical data for all new compounds, X-ray crystallographic data, NMR and HPLC spectra, computational details, Supplementary Figs. 1–30, Tables 1–3 and references.

Supplementary Data 1 Crystallographic data for compounds **1s′**; CCDC reference, 2270374.

Supplementary Data 2 Crystallographic data for compounds **2.3**; CCDC reference, 2212927.

Supplementary Data 3 Crystallographic data for compounds **2.11**; CCDC reference, 2212932.

Supplementary Data 4 Coordinates of DFT calculations.

## Data Availability

All data collected as part of this study are available within the Article and its Supplementary Information. Crystallographic data for the structures reported in this Article have been deposited at the Cambridge Crystallographic Data Centre, under deposition nos. CCDC 2270374 (**1s′**), 2212927 (**2.3**) and 2212932 (**2.11**). Copies of the data can be obtained free of charge via https://www.ccdc.cam.ac.uk/structures/.
