# Supplementary material for: Chiral arylsulfinylamides as reagents for visible light-mediated asymmetric alkene aminoarylations
Source: Nat Chem. 2024 Jan 16;16(4):607–14. doi: 10.1038/s41557-023-01414-8 (PMC10997517; doi:10.1038/s41557-023-01414-8)
Supplement: Supplementary file 1 — General information, reaction optimization data, experimental procedures, additional experiments, compound characterization including spectroscopic and analytical data for all new compounds, X-ray crystallographic data, NMR and HPLC spectra, computational details, Supplementary Figs. 1–30, Tables 1–3 and references. [file 41557_2023_1414_MOESM1_ESM.pdf]

nature chemistry

Article https://doi.org/10.1038/s41557-023-01414-8

Chiral arylsulfinylamides as reagents for visible light-mediated asymmetric alkene aminoarylations

In the format provided by the authors and unedited

General information **S2**

Optimization of reaction conditions **S4**

General procedure for the synthesis of arylsulfinylamides (1a-1t) **S5**

General procedure for the synthesis of products (2.1-2.38) **S6**

Procedure for the synthesis of amine (2.39) **S8**

Procedure for the synthesis of indoline (2.40) **S8**

Procedure for the synthesis of tetrabutylammonium sulfinylamide (3) **S9**

Synthesis of (*R*)-1-(4,8-dimethylnona-1,7-dien-1-yl)-4-methoxybenzene **S9**

Control experiments **S10**

Characterization data of *N*-sulfinylamines **S27**

Characterization data of arylsulfinylamides (1a-1t) **S30**

Characterization data of products (2.1-2.40) **S40**

X-Ray crystallographic data **S61**

Copies of NMR spectra and HPLC traces **S65**

DFT calculations **S214**

References **S217**

General information

Unless otherwise stated, reactions were carried out using dry solvents under nitrogen atmosphere. Starting materials were purchased from Merck, Fluka and TCI. Pentane, hexane and ethyl acetate were purchased with HPLC quality, degassed by purging thoroughly with nitrogen and dried over activated molecular sieves of appropriate size. Alternatively, tetrahydrofuran, acetonitrile and dichloromethane were dried using a solvent purification system (Pure Solv PS-MD-4EN, Innovative Technology Inc.) equipped with alumina drying columns under argon. Conversion was monitored by thin layer chromatography (TLC) using Merck TLC silica gel 60 F254 and visualized by UV-light at 254 nm or using Advion MS. Flash column chromatography was performed over silica gel (230-400 mesh). All NMR spectra were recorded on AV2-400 and 500 MHz Bruker spectrometers. Chemical shifts are given in ppm and the spectra are calibrated using the residual chloroform signals (7.26 ppm for ^{1}H NMR and 77.16 ppm for ^{13}C NMR), the residual acetone signals (2.05 ppm for ^{1}H NMR and 29.8 ppm, for ^{13}C NMR) and the residual dimethyl sulfoxide signals (2.50 ppm for ^{1}H NMR and 39.5 ppm, for ^{13}C NMR). Multiplicities are abbreviated as follows: singlet (s), doublet (d), triplet (t), quartet (q), doublet-doublet (dd), quintet (quint), septet (sept), multiplet (m), and broad (br). Infrared spectra were recorded on a JASCO FT/IR-4100 spectrometer. Absorptions are reported in wavenumber (cm^{-1}). High-resolution electrospray ionization and electronic impact mass spectrometry were performed on a Finnigan MAT 900 (Thermo Finnigan, San Jose, CA; USA) double focusing magnetic sector mass spectrometer. Ten spectra were acquired. A mass accuracy $\leq$ 2 ppm was obtained in the peak matching acquisition mode by using a solution containing 2 <l PEG200, 2 <l PPG450, and 1.5 mg NaOAc (all obtained from Merck, CH-Buchs) dissolved in 100 mL MeOH (HPLC Supra grade, Scharlau, E-Barcelona) as internal standard. GC-MS analysis was done on a Finnigan Voyager GC8000 Top. For the analysis of the reaction headspace, a Thermo-Finegan Trace GC ultra + Trace DSQ device equipped with a GC capillary column (Carbowax), 20 m length, 0.25 mm i.d., 0.25 mm film thickness from Ohio Valley Specialty (Marietta, Ohio) was used. Melting points were measured on melting point operators: MPM-MHV from Müller + Krempel AG and are uncorrected. The enantiomeric ratios were determined by chiral HPLC analysis performed on JASCO HPLC system equipped with a PU-980 pump, a UV-970 detector, measured at 254 nm and a chiral column. Optical rotations were measured on a JASCO P-1010 polarimeter. Fluorescence quenching was recorded with a SpectroFluorometer

FS5 using Fluoracle software. Cyclic voltammetry was recorded with a Metrohm 797 VA Computrace under N_2 atmosphere.

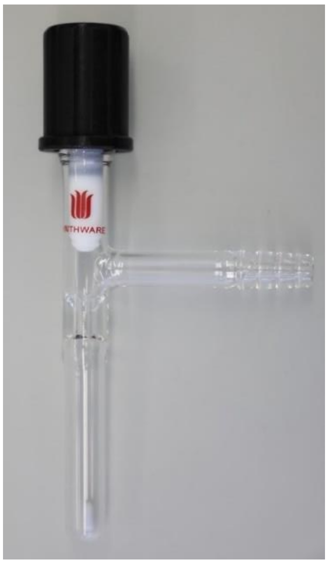

Supplementary Figure 1: 5 mL Schlenk tube used to set up the reaction.

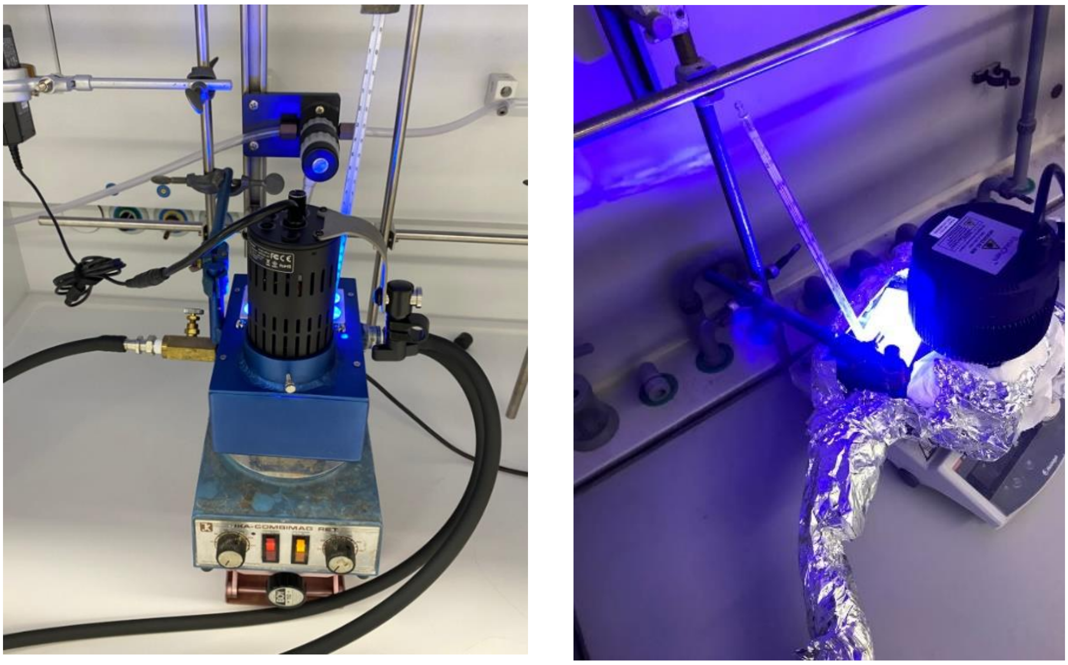

Supplementary Figure 2: Set-up of the photoredox reaction

All photoredox reactions were performed in a 5 mL Schlenk tube (Figure S1) under maximum stirring rate (1400 rpm). The set-up of the photoredox reaction (Figure S2) was composed of the EvoluChem^{TM} PhotoRedOx Box TC from HepatoChem equipped with EvoluChem^{TM} blue LEDs array (30W, HCK1012-01-008). In addition, a Julabo chiller was used to keep the temperature inside the photoreactor at -20 °C.

Optimization of the reaction conditions

Supplementary Table 1. Reaction optimization results

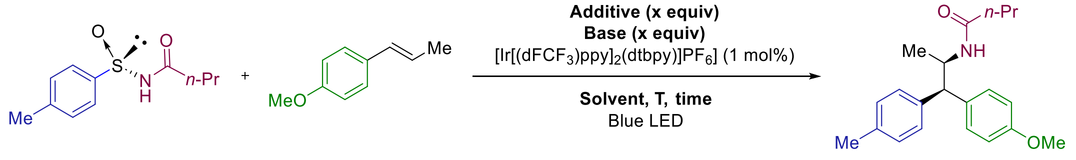

(S_s)-**1a** **>99:1 e.r.** 1.0 equiv — **x equiv** — **2.1** **>20:1 d.r.**

Entry	Solvent (M)	Additive (equiv)	Additive (equiv)	Olefin (equiv)	Temperature (°C)	Time (h)	Yield (%)	e.r.
1	DMSO (0.1 M)	-	$PhCO_2K$ (0.3)	1.2	32	12	Trace	-
2	1,4-Dioxane (0.1 M)	-	$PhCO_2K$ (0.3)	1.2	32	12	0	-
3	Toluene (0.1 M)	-	$PhCO_2K$ (0.3)	1.2	32	12	0	-
4	C_6F_6 (0.1 M)	-	$PhCO_2K$ (0.3)	1.2	32	12	0	-
5	HFIP (0.1 M)	-	$PhCO_2K$ (0.3)	1.2	32	12	0	-
6	$PhCF_3$ (0.1 M)	-	$PhCO_2K$ (0.3)	1.2	32	12	11	75:25
7	MeCN (0.1 M)	-	$PhCO_2K$ (0.3)	1.2	32	12	10	75:25
8	DMF (0.1 M)	-	$PhCO_2K$ (0.3)	1.2	32	12	24	70:30
9	*i*-PrOH (0.1 M)	-	$PhCO_2K$ (0.3)	1.2	32	12	31	80:20
10	*i*-PrOH:H_2O (9:1 ; 0.2 M)	-	$PhCO_2K$ (0.3)	1.2	32	12	42	86:14
11	*i*-PrOH:H_2O (9:1 ; 0.2 M)	LiCl (1)	$PhCO_2K$ (0.3)	1.2	32	12	41	88:12
12	*i*-PrOH:H_2O (9:1 ; 0.2 M)	LiOH (1)	$PhCO_2K$ (0.3)	1.2	32	12	25	87:13
13	*i*-PrOH:H_2O (9:1 ; 0.2 M)	HFIP (10)	$PhCO_2K$ (0.3)	1.2	32	12	50	88:12
14	*i*-PrOH:H_2O (9:1 ; 0.2 M)	CF_3CH_2OH (10)	$PhCO_2K$ (0.3)	1.2	32	12	53	90:10
15	*i*-PrOH:H_2O (9:1 ; 0.2 M)	CF_3CH_2OH (10)	$PhCO_2K$ (0.3)	1.2	18	12	48	92:8
16	*i*-PrOH:H_2O (9:1 ; 0.2 M)	CF_3CH_2OH (10)	$PhCO_2K$ (0.3)	1.2	-14	36	62	94:6
17	*i*-PrOH:H_2O (9:1 ; 0.2 M)	CF_3CH_2OH (10)	$PhCO_2K$ (1.0)	1.2	-20	96	trace	>99:1
18	*i*-PrOH:H_2O (9:1 ; 0.2 M)	CF_3CH_2OH (10)	K_3PO_4 (0.3)	1.2	-20	96	33	-
19[a]	*i*-PrOH:H_2O (9:1 ; 0.2 M)	CF_3CH_2OH (10)	$NBu_4OP(O)(OBu)_2$ (0.3)	1.2	-20	96	trace	-
20[b]	*i*-PrOH:H_2O (9:1 ; 0.2 M)	CF_3CH_2OH (10)	$PhCO_2K$ (0.3)	1.2	-20	96	25	-
21[a]	*i*-PrOH:H_2O (9:1 ; 0.2 M)	CF_3CH_2OH (10)	$PhCO_2K$ (0.3)	1.2	-20	96	38	-
22	*i*-PrOH:H_2O (9:1 ; 0.2 M)	CF_3CH_2OH (10)	$PhCO_2K$ (0.3)	1.2	-20	96	58	-
**23**	***i*-PrOH:H_2O (9:1 ; 0.2 M)**	**CF_3CH_2OH (10)**	**$PhCO_2K$ (0.3)**	**2**	**-20**	**96**	**83**	**>99:1**

[a] $[Ir(dFCF_3)ppy_2(5,5'\text{-}dCF_3bpy)]PF_6$ was used as photocatalyst; [b] 4CzIPN was used as photocatalyst.

Supplementary Table 2. Control experiments

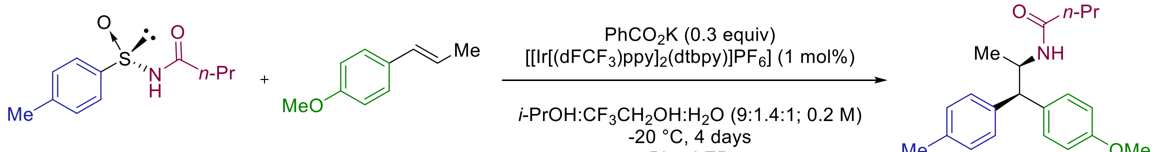

Blue LED

(S_S)-**1a** **>99:1 e.r.** 1.0 equiv — 2.0 equiv — **2.1** **>20:1 d.r.**

Entry	Control	Yield (%)[a]	e.r.
1	no photocatalyst	0	-
2	no base	0	-
3	no light	0	-
4	no CF_3CH_2OH	56	94:6
5	TEMPO (2 equiv)	0	-
6	BHT (2 equiv)	0	-

[a]Alkene and sulfinylamide were recovered

General procedure for the synthesis of arylsulfinylamides (1a-1t)

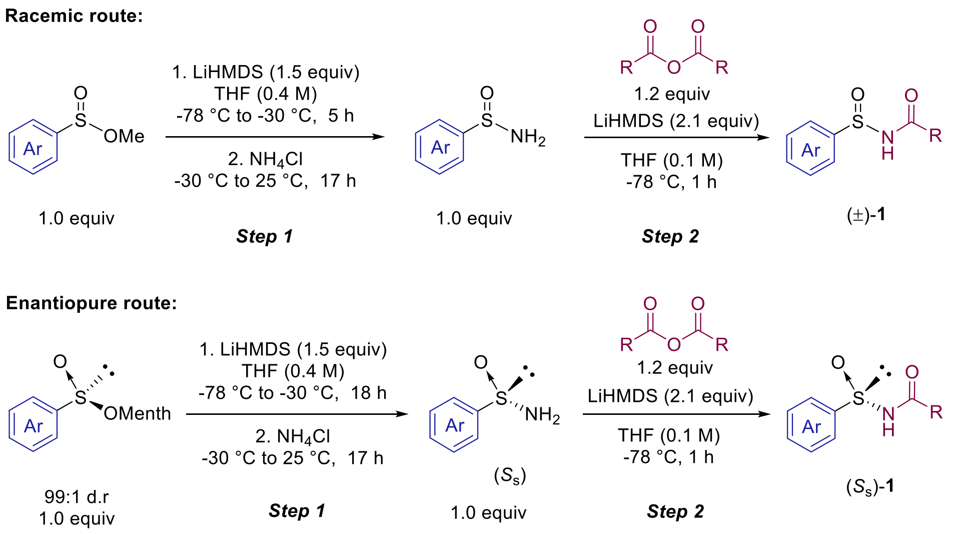

Supplementary Figure 3: Synthetic route for the preparation of the arylsulfinylamides

Step 1

Arylsulfinylamines were prepared by modification of a previously reported procedure.[1] In a flame-dried 50 mL two-necked round bottomed flask, the corresponding sulfinate (4 mmol, 1 equiv) was dissolved in THF (0.4 M, 10 mL) under nitrogen atmosphere and cooled to -78 °C (dry ice-acetone bath). Then LiHMDS (1 M in THF, 6 mL, 6 mmol, 1.5 equiv) was added dropwise and the resulting mixture was warmed up to -30 °C and stirred for 5 h (for racemic substrate) or 18 h (for enantioenriched substrate). At that time, full conversion of starting material was detected by TLC. The reaction was quenched at -30 °C with an aqueous saturated NH_4Cl solution and stirred additionally for 17 h at 24 °C. The phases were separated, and aqueous phase was extracted with CH_2Cl_2 (3 x 20 mL). The combined organic layers were washed with brine (10 mL), dried over Na_2SO_4, filtered, and concentrated *in vacuo*. The residue was purified by flash chromatography using a mixture EtOAc:CH_2Cl_2 to afford the corresponding arylsulfinylamines.

Step 2

In a 50 mL two-necked round bottomed flask under nitrogen flow, the corresponding sulfinylamide (2 mmol, 1 equiv) was dissolved in THF (10 mL) and cooled to -78 °C. Then LiHMDS (1.0 M in THF, 4.2 mL, 4.2 mmol, 2.1 equiv) was added dropwise. After 20 min, the symmetrical anhydride (2.4 mmol, 1,2 equiv) was added slowly. After 1 hour, the crude mixture was diluted with ethyl acetate (10 mL) and quenched with an aqueous saturated $NaHCO_3$ solution (3 x 10 mL). The organic layers was dried over $MgSO_4$, filtered and concentrated *in vacuo* and the residue was purified by flash chromatography using a mixture EtOAc:*n*-hexane.

General procedure for the synthesis of products (2.1-2.38)

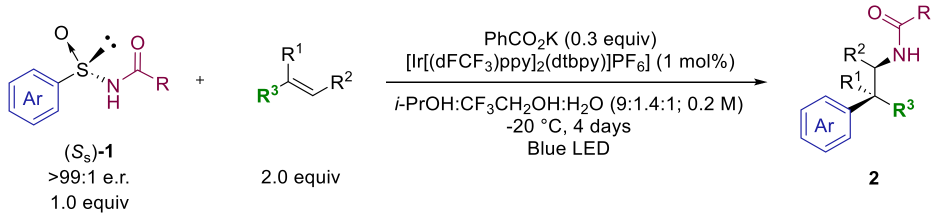

To an oven-dried Schlenk tube (5 mL) the corresponding arylsulfinylamide (0.1 mmol, 1 equiv), $PhCO_2K$ (4.8 mg, 0.03 mmol, 0.3 equiv), and $Ir[(dFCF_3)ppy]_2(dtbpy)]PF_6$ (1.1 mg, 0.001 mmol, 1 mol%) were sequentially added under a flow of nitrogen. The flask was evacuated and then filled back with N_2 (three times). Then, trifluoroethanol (72 μL) and *i*-PrOH:H_2O (9:1 (v:v), 0.5 mL) were added to the reaction mixture followed by the olefin (0.2 mmol, 2.0 equiv). The reaction was sparged with argon for 15 min. The Schlenk tube was placed in the photo-reactor and stirred at 1400 rpm under blue light irradiation at -20°C. After 4 days, the reaction mixture was diluted with EtOAc (10 mL) and transferred into a separatory funnel. The mixture was washed with a 5 wt% aqueous LiCl solution (3 x 10 mL). The organic phase was dried over $MgSO_4$, filtered, and concentrated *in vacuo*. The residue was purified by flash chromatography using a mixture EtOAc:*n*-hexane.

General procedure for the large scale synthesis of product (2.1)

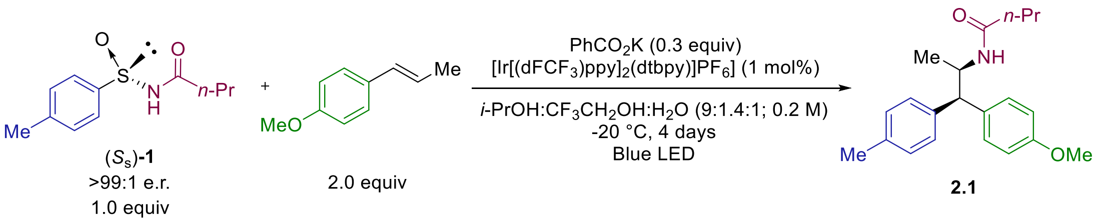

To an oven-dried Schlenk tube (20 mL) the corresponding arylsulfinylamide (1.0 mmol, 1 equiv), $PhCO_2K$ (48 mg, 0.3 mmol, 0.3 equiv), and Ir[(dFCF$_3$)ppy]$_2$(dtbpy)]PF$_6$ (11.1 mg, 0.01 mmol, 1 mol%) were sequentially added under a flow of nitrogen. The flask was evacuated and then filled back with N_2 (three times). Then, trifluoroethanol (720 μL) and *i*-PrOH:H_2O (9:1 (v:v), 5.0 mL) were added to the reaction mixture followed by the olefin (2.0 mmol, 2.0 equiv). The reaction was sparged with argon for 15 min. The Schlenk tube was stirred at 1400 rpm under blue light irradiation at -10°C. After 6 days, the reaction mixture was diluted with EtOAc (50 mL) and transferred into a separatory funnel. The mixture was washed with a 5 wt% aqueous LiCl solution (3 x 20 mL). The organic phase was dried over $MgSO_4$, filtered, and concentrated *in vacuo*. The residue was purified by flash chromatography using a mixture EtOAc:*n*-hexane affording **2.1** (>20:1 d.r, 58% yield, 98:2 e.r).

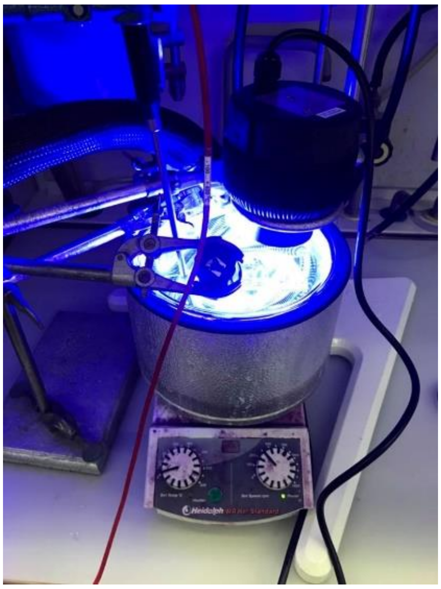

Supplementary Figure 4: Set-up of the large scale photoredox reaction

Procedure for the synthesis of amine (2.39)

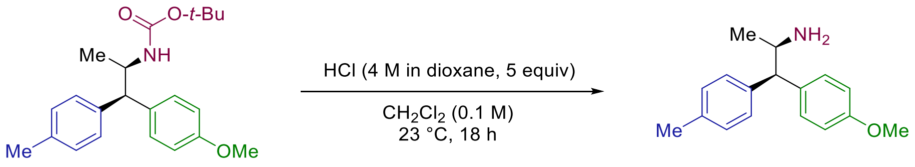

2.9
94:6 e.r.
1.0 equiv

2.39
64%; 94:6 e.r.

In a 25 mL two-necked round bottomed flask, the corresponding carbamate **2.9** (14.5 mg, 0.041 mmol, 1 equiv) was dissolved in HPLC grade CH_2Cl_2 (0.41 mL). Then HCl solution (4.0 M in dioxane, 51 µL, 0.204 mmol, 5 equiv) was added dropwise. The reaction mixture was stirred for 18 h (upon that time full conversion of starting material was observed by TLC; EtOAc:*n*-hexane, 1:2), the crude mixture was diluted with CH_2Cl_2 (10 mL) and quenched with an 0.5 M aqueous NaOH solution (20 mL) and washed with brine. The organic layers were dried over $MgSO_4$, filtered, and concentrated *in vacuo* to give amine **2.39** (6.7 mg, 64% yield).

Procedure for the synthesis of indoline (2.40)

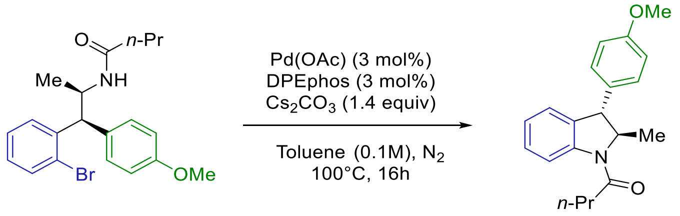

2.18
99:1 e.r.
1.0 equiv

2.40
98%, 99:1 e.r

An oven-dried 5 mL pressure tube equipped with a magnetic stirring bar was charged with $Pd(OAc)_2$ (0.018 mmol, 3.0 mol%) and DPEphos (0.018 mmol, 3 mol%). The Schlenk was evacuated and flushed with argon. A solution of **2.18** (0.6 mmol; 1 equiv.) in toluene (0.2 mL) was added via cannula. The reaction mixture was heated at 100 °C for 2 min to get a homogeneous solution. Then, Cs_2CO_3 (0.84 mmol, 1.4 equiv.) and toluene (0.2 mL) were added. The reaction tube was sealed and heated at 100 °C until **2.18** was consumed (16 h). The reaction mixture was allowed to cool down to room temperature and concentrated under reduced pressure. The residue was purified by column chromatography using a mixture EtOAc:*n*-hexane to afford **2.40** as a colorless oil.

Procedure for the synthesis of tetrabutylammonium sulfinylamide (3)

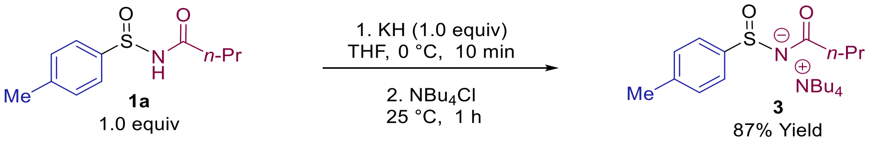

To an oven-dried Schlenk tube (20 mL) was added potassium hydride (30% dispersion wt% in mineral oil, 1.0 mmol, 1 equiv.). The flask was evacuated and backfilled with N_2 at which point 10 mL of pentane was added. The flask was gently swirled, and the pentane-mineral oil solution was removed with care taken not to remove the KH. The KH was washed twice more with pentane and the pentane-mineral oil solutions were removed as described. The flask was evacuated and backfilled with N_2 a final time and reweighed to ensure the mass of the solid KH had not decreased from the washes. The KH was suspended in THF (5 mL) and cooled to 0°C in an ice bath. To this rapidly stirring suspension was added a fine suspension of the 4-arylsulfinylamide **1a** (1.0 mmol, 1 equiv.) in THF (2.5 mL). The solution was left to stir for 10 minutes in the ice bath before being removed to warm to room temperature. Tetrabutylammonium chloride (0.760 mmol, 1 equiv) was added as a fine suspension in THF (5.0 mL). The solution was left to stir for 1 hour at room temperature. The contents of the reaction were filtered through a 1 cm pad of Celite on a medium porosity frit. The Celite was washed with additional THF (5 mL), CH_2Cl_2 (5 mL), and the filtrate was concentrated to give a pale-yellow thick oil. Using a sand bath heated to 70°C, this oil was dried in vacuo overnight to give the product as a yellow oil.

Synthesis of (*R*)-1-(4,8-dimethylnona-1,7-dien-1-yl)-4-methoxybenzene[2]

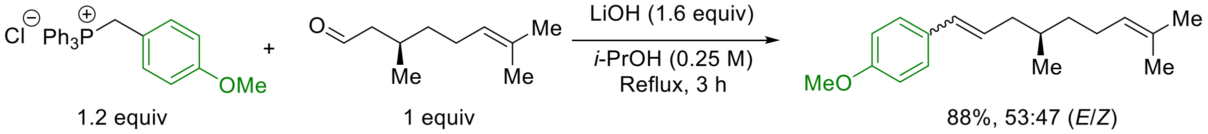

LiOH (1.6 mmol) was added to a suspension of the phosphonium salt (1.2 mmol) in isopropyl alcohol (4 mL). After 15 min, (+)-citronellal (1.0 mmol) was added to the mixture and the reaction was heated to reflux for 3 h. The mixture was quenched with water and extracted with Et_2O. The organic phase was washed with brine, dried with Na_2SO_4, and evaporated in vacuum. The residue was purified by silica gel flash chromatography (*n*-hexane/EtOAc 95:5) to afford the product as a mixture of *E*/*Z* isomers.

Control experiments:

Profile for *E*-anethole isomerization under the reaction conditions

To a flame dried J-Young cap NMR tube containing a capillary filled with dimethylsulfoxide-*d*6, Ir[(dFCF$_3$)ppy]$_2$(dtbpy)]PF$_6$ (0.001 mmol, 1 mol%) was added under a flow of nitrogen. The NMR tube was evacuated and then filled back with N_2 (three times). Then, trifluoroethanol (1 mmol, 10 equiv) and *i*-PrOH:H_2O (9:1 (v:v), 0.5 mL) followed by the *E*-anethole (0.2 mmol, 2.0 equiv) were added in the reaction mixture. The reaction was sparged with argon for 15 min. Finally, trimethyl(phenyl)silane (0.1 mmol) was added to the reaction as internal standard. Time zero (t = 0) ^{1}H-NMR spectrum was recorded prior irradiation. Then the NMR tube was placed in the photoreactor under blue light irradiation at -20°C. After the allotted time, the light was turned off and reaction solution was analyzed by ^{1}H-NMR. Temporal concentrations (%) of both isomers were calculated based on the internal standard and they were plotted against time in minutes. After 10 min a photostationary state was reached featuring a 1:1.78 ration of *E* vs *Z* anethole

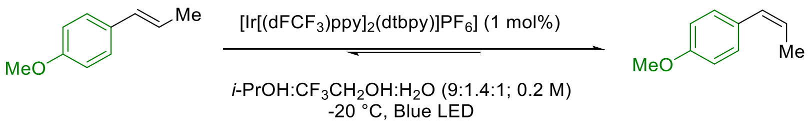

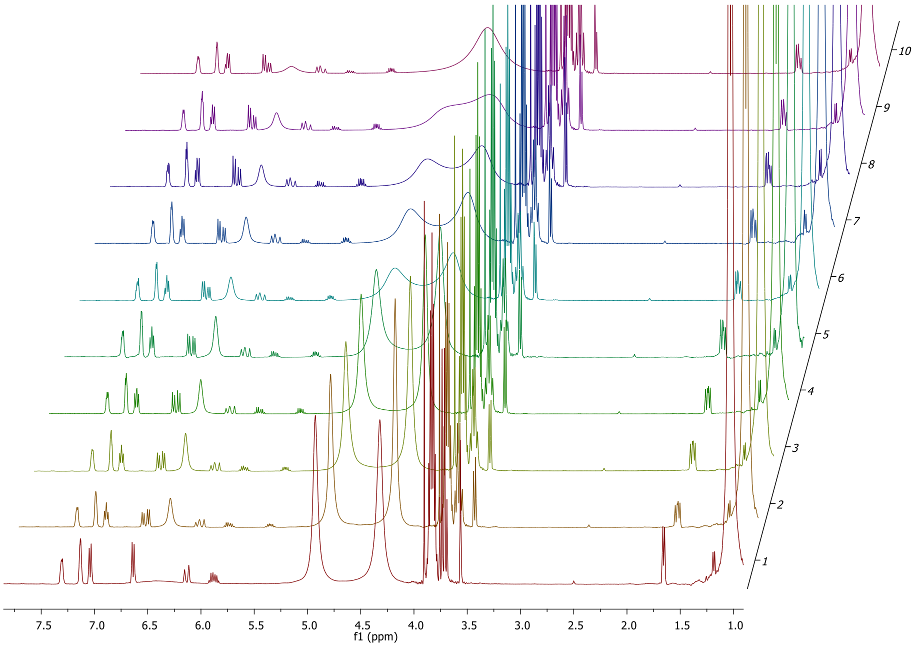

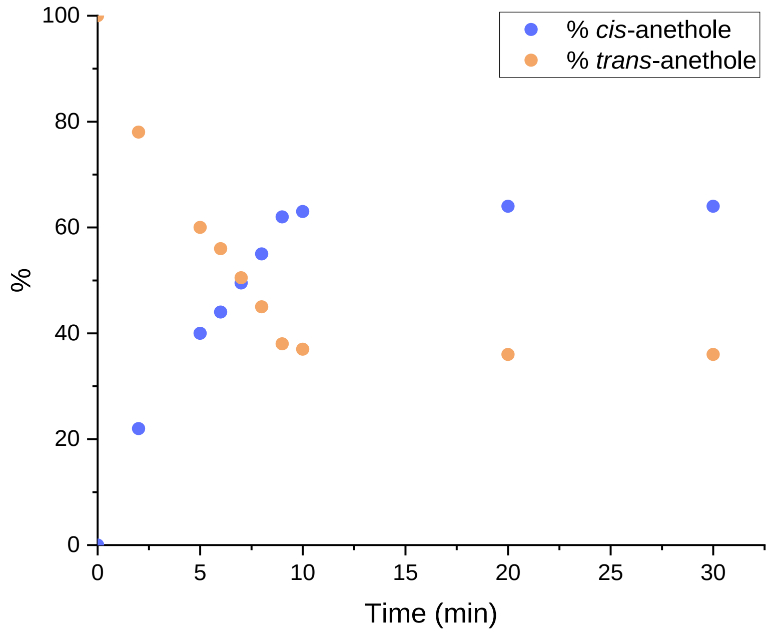

Supplementary Figure 5: Plotted isomerization profile for *E*-anethole.

Profile for *Z*-anethole isomerization under the reaction conditions

To a flame dried J-Young cap NMR tube containing a capillary filled with dimethylsulfoxide-*d*6, Ir[(dFCF$_3$)ppy]$_2$(dtbpy)]PF$_6$ (0.001 mmol, 1 mol%) was added under a flow of nitrogen. The NMR tube was evacuated and then filled back with N$_2$ (three times). Then, trifluoroethanol (1 mmol, 10 equiv) and *i*-PrOH:H$_2$O (9:1 (v:v), 0.5 mL) followed by the *Z*-anethole (0.2 mmol, 2.0 equiv) were added in the reaction mixture. The reaction was sparged with argon for 15 min. Finally, trimethyl(phenyl)silane (0.1 mmol) was added to the reaction as internal standard. Time zero (t = 0) ^{1}H-NMR spectrum was recorded prior irradiation. Then the NMR tube was placed in the photoreactor under blue light irradiation at -20°C. After the allotted time, the light was turned off and reaction solution was analyzed by ^{1}H-NMR. Temporal concentrations (%) of both isomers were calculated based on the internal standard and they were plotted against time in minutes. After 10 min a photostationary state was reached featuring a 1.70:1 ration of Z vs *E* anethole

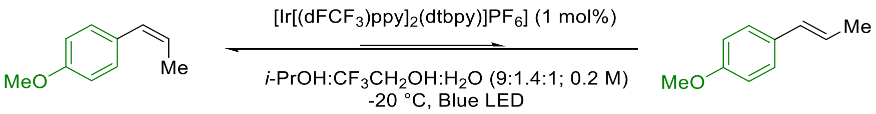

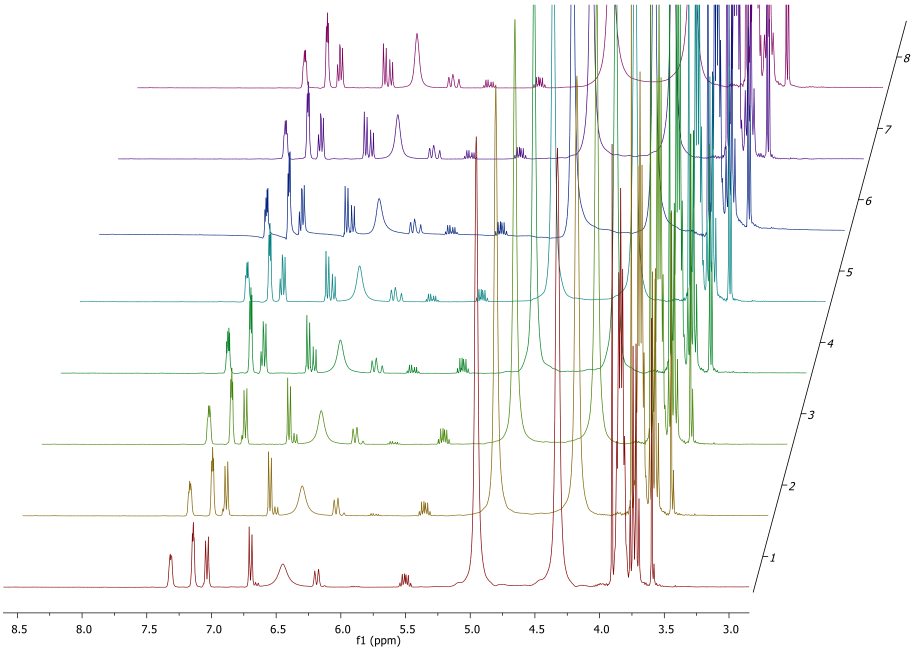

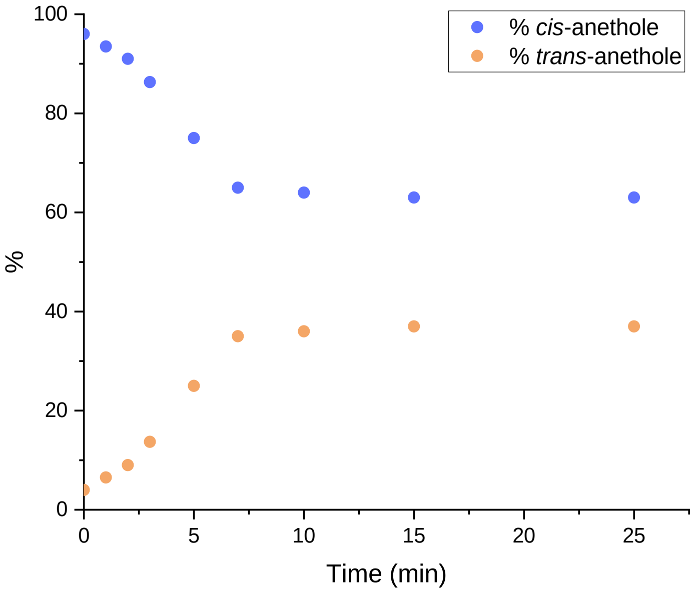

Supplementary Figure 6: Plotted isomerization profile for *Z*-anethole.

Stern-Volmer quenching experiments

Fluorescence quenching of [Ir[(dFCF$_3$)ppy]$_2$(dtbpy)]PF$_6$] was recorded with a SpectroFluorometer FS5 using Fluoracle software. Samples consisting of noted concentration of quencher in *i*-PrOH:CF$_3$CH$_2$OH:H$_2$O (9:1.4:1) were prepared and degassed by sparging with argon for 10 minutes. The solutions were irradiated at 430 nm and luminescence was measured at 593 nm. I_0/I values were generated from the average of three scans taken per quencher concentration. [Ir] = 4x10^{-5} M.

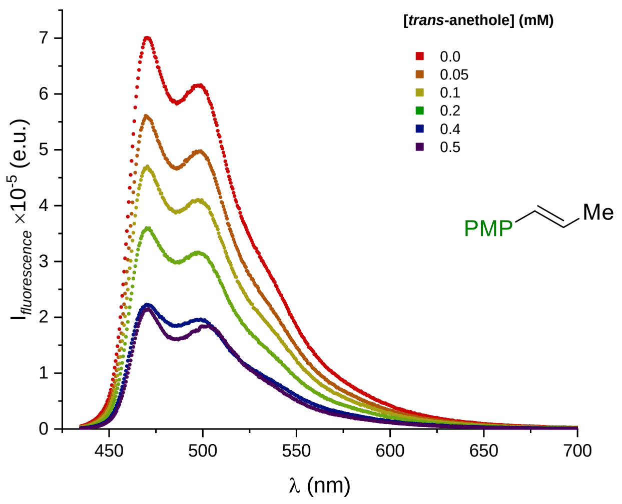

Supplementary Figure 7: Evolution of fluorescence in presence of *trans*-anethole.

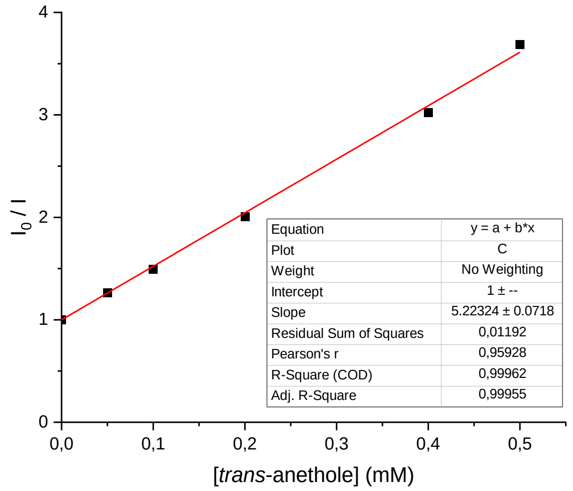

Supplementary Figure 8: Plotted ratio of fluorescence intensity with concentration of *trans*-anethole.

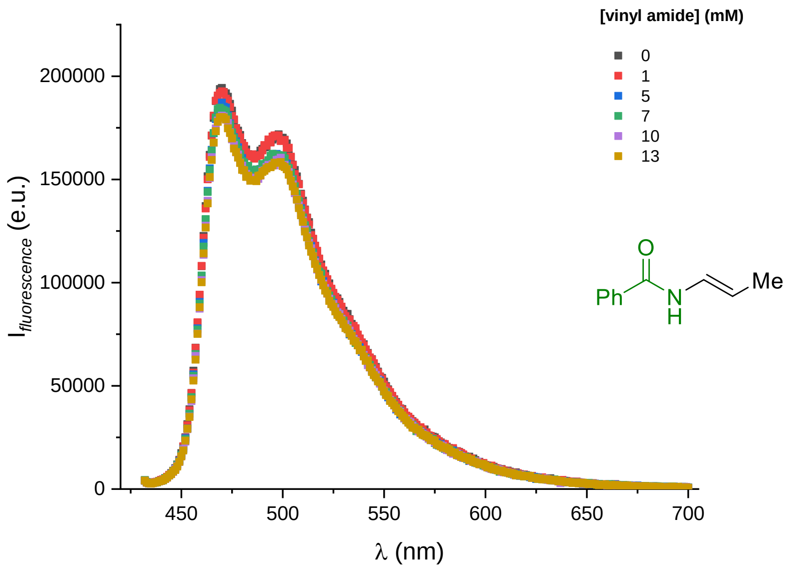

Supplementary Figure 9: Evolution of fluorescence in presence of vinylamide.

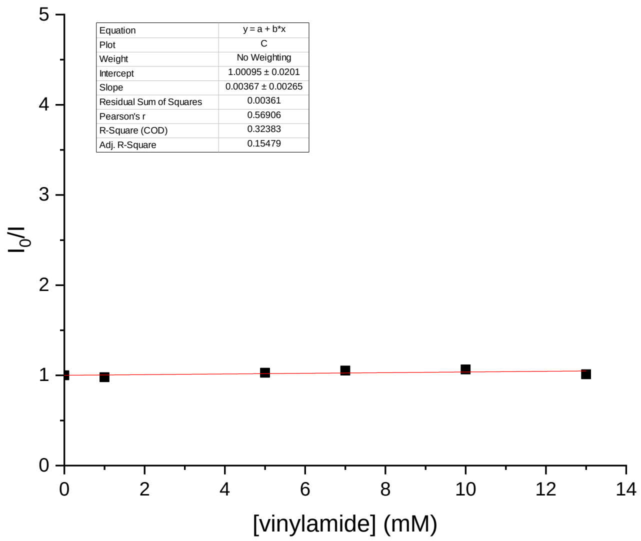

Supplementary Figure 10: Plotted ratio of fluorescence intensity with concentration of vinylamide.

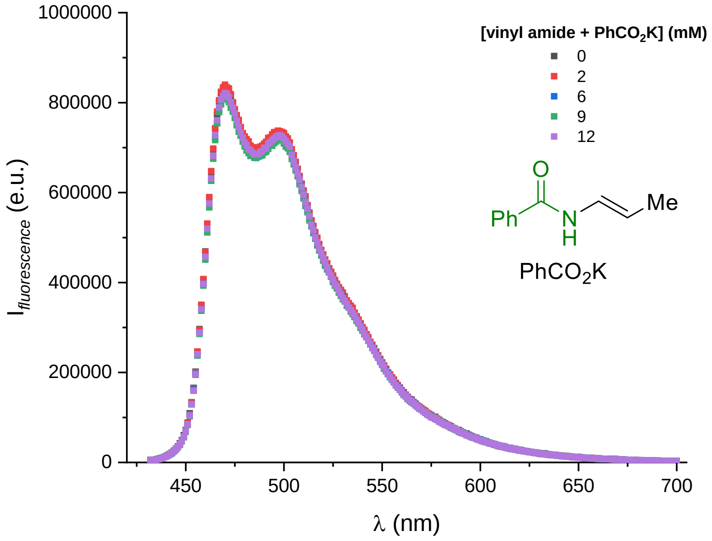

Supplementary Figure 11: Evolution of fluorescence in presence of vinylamide and $PhCO_2K$

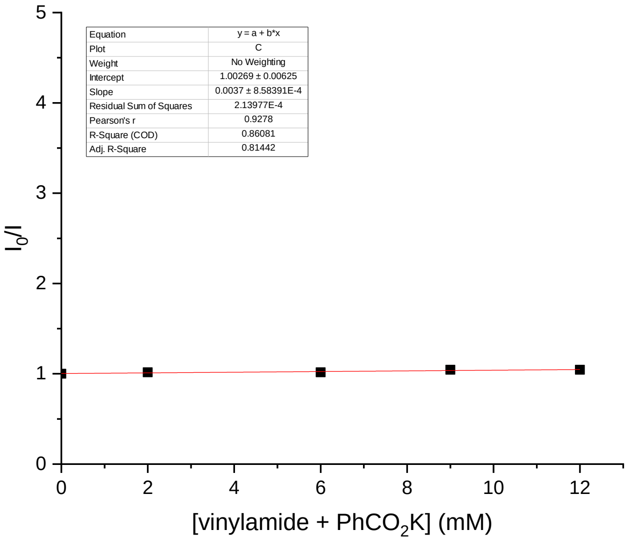

Supplementary Figure 12: Plotted ratio of fluorescence intensity with concentration of vinylamide and $PhCO_2K$.

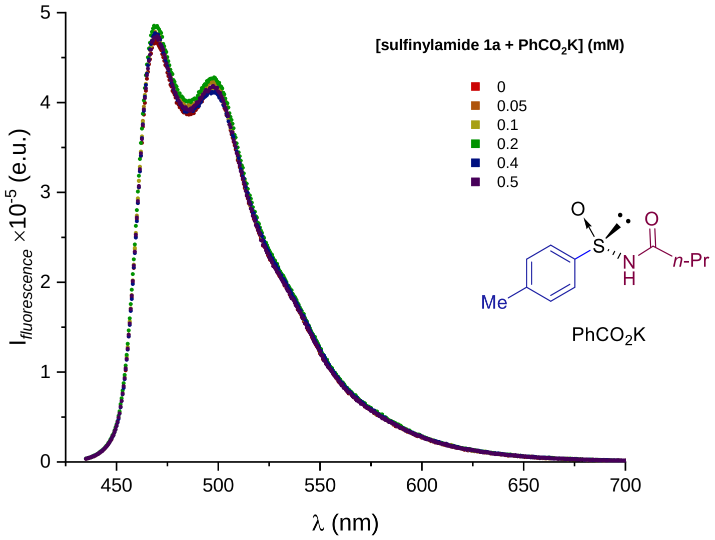

Supplementary Figure 13: Evolution of fluorescence in presence of arylsulfinylamide **1a**.

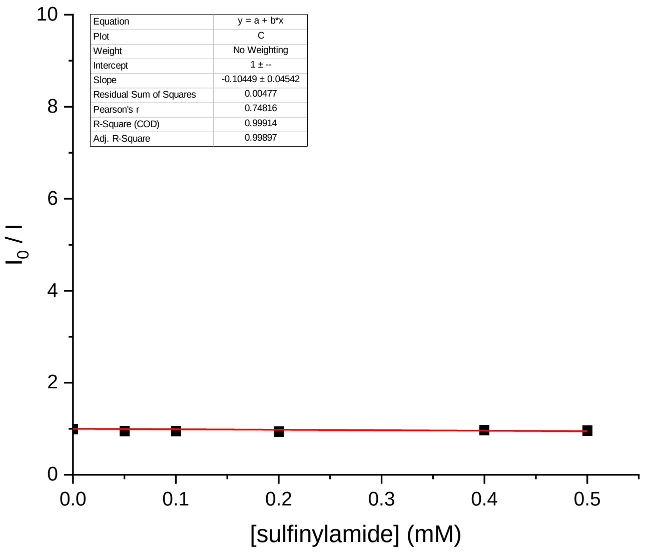

Supplementary Figure 14: Plotted ratio of fluorescence intensity with concentration of arylsulfinylamide **1a**.

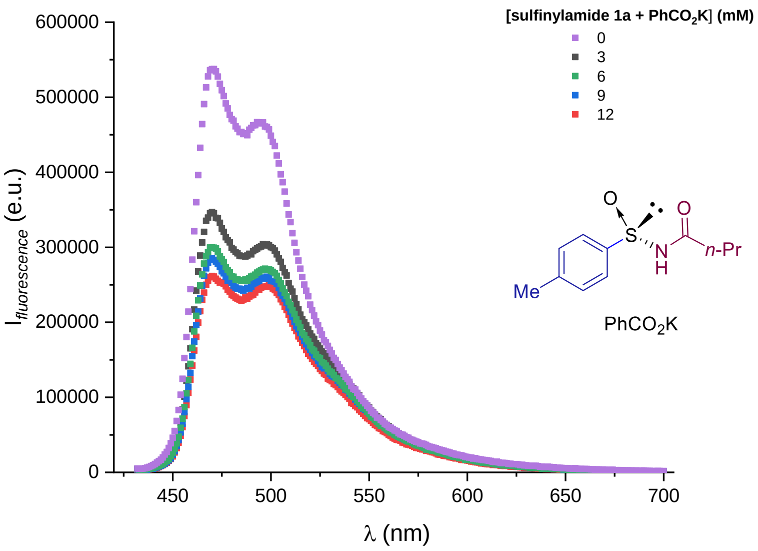

Supplementary Figure 15: Evolution of fluorescence in presence of arylsulfinylamide **1a** and $PhCO_2K$

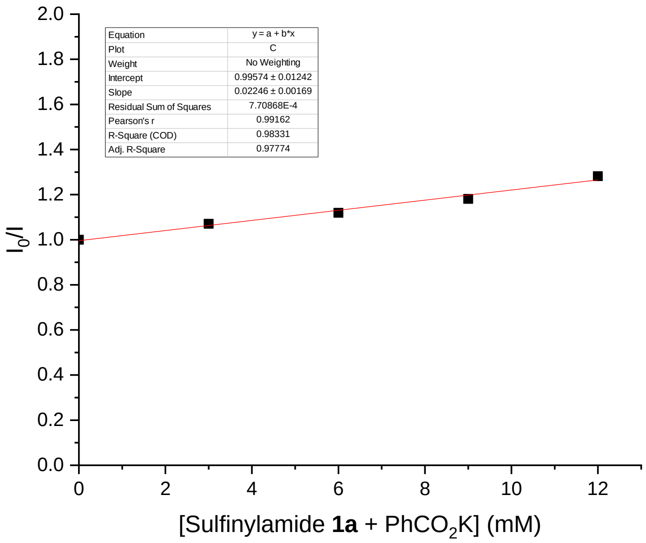

Supplementary Figure 16: Plotted ratio of fluorescence intensity with concentration of arylsulfinylamide **1a** and $PhCO_2K$.

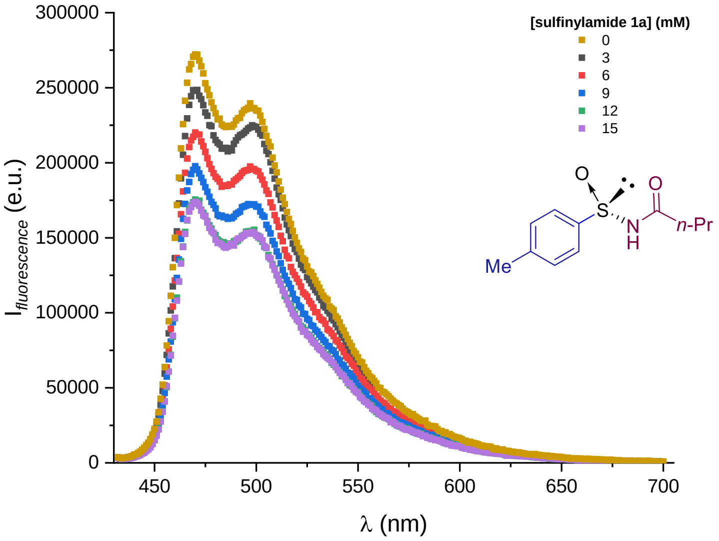

Supplementary Figure 17: Evolution of fluorescence in presence of arylsulfinylamide **1a** at high concentation.

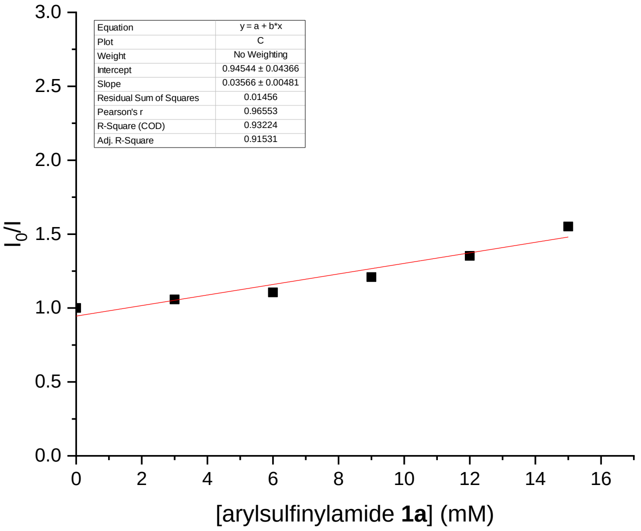

Equation	y = a + b*x
Plot	C
Weight	No Weighting
Intercept	0.94544 ± 0.04366
Slope	0.03566 ± 0.00481
Residual Sum of Squares	0.01456
Pearson's r	0.96553
R-Square (COD)	0.93224
Adj. R-Square	0.91531

Supplementary Figure 18: Plotted ratio of fluorescence intensity with concentration of arylsulfinylamide **1a** at high concentation.

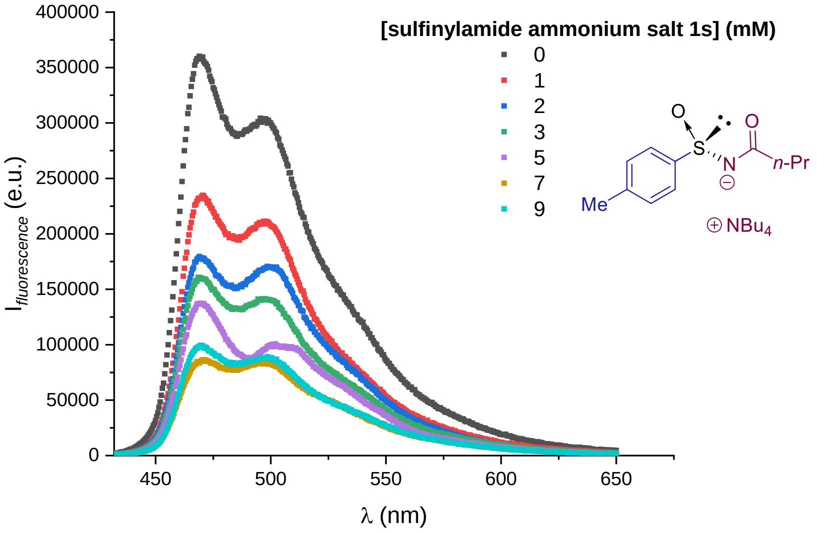

Supplementary Figure 19: Evolution of fluorescence in presence of arylsulfinylamide ammonium salt **3**

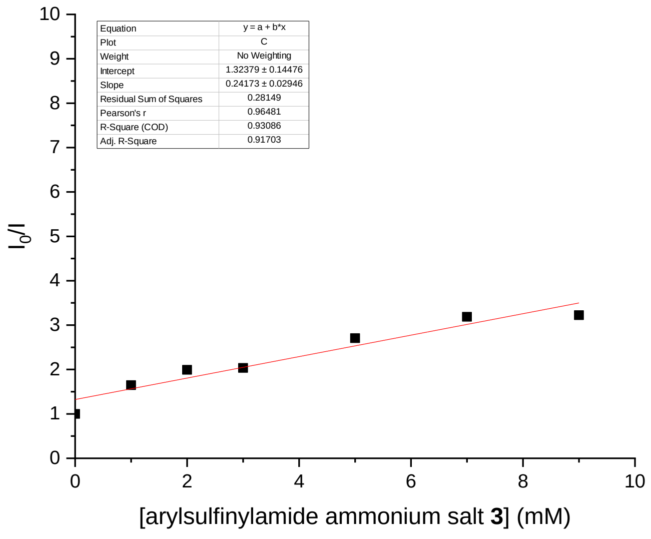

Equation	y = a + b*x
Plot	C
Weight	No Weighting
Intercept	1.32379 ± 0.14476
Slope	0.24173 ± 0.02946
Residual Sum of Squares	0.28149
Pearson's r	0.96481
R-Square (COD)	0.93086
Adj. R-Square	0.91703

Supplementary Figure 20: Plotted ratio of fluorescence intensity with concentration of arylsulfinylamide ammonium salt **3**.

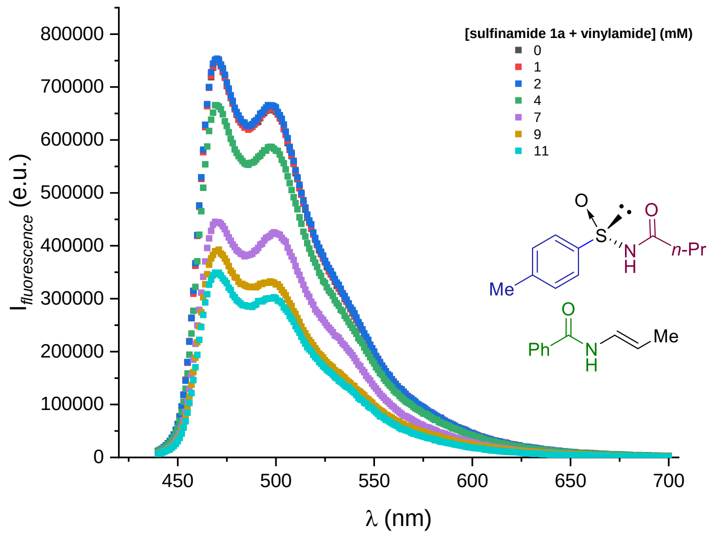

Supplementary Figure 21: Evolution of fluorescence in presence of arylsulfinylamide **1a** and vinylamide

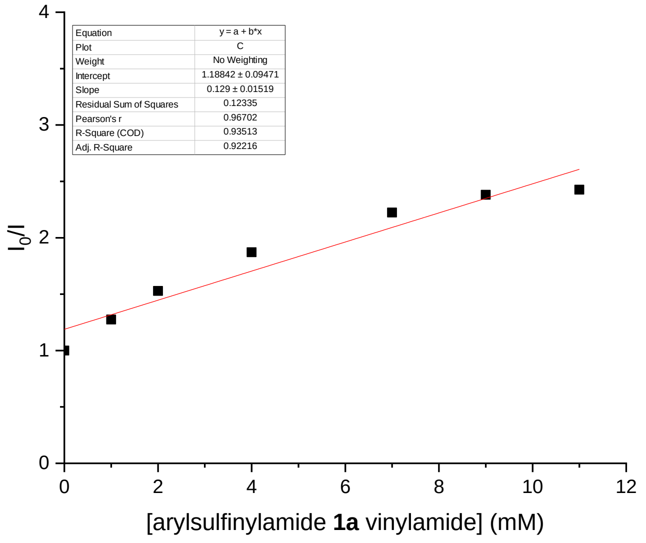

Supplementary Figure 22: Plotted ratio of fluorescence intensity with concentration of arylsulfinylamide **1a** and vinylamide

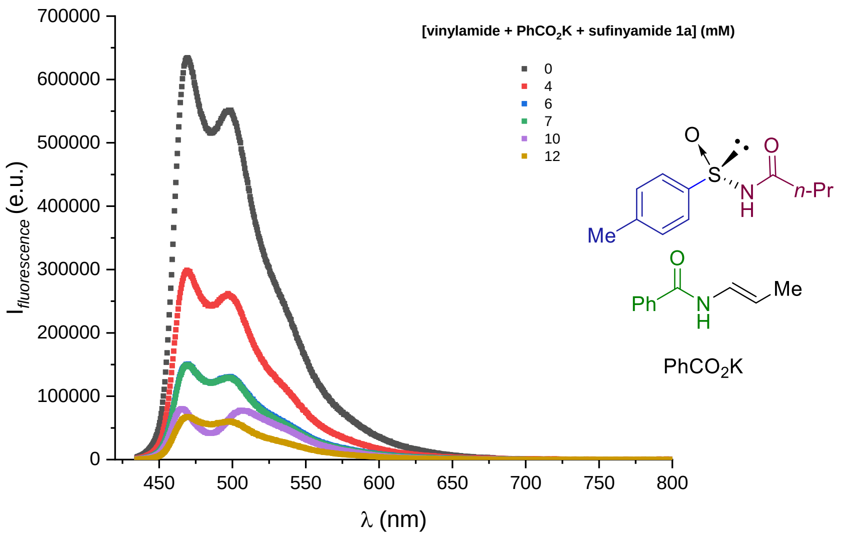

Supplementary Figure 23: Evolution of fluorescence in presence of arylsulfinylamide **1a** and vinylamide and $PhCO_2K$

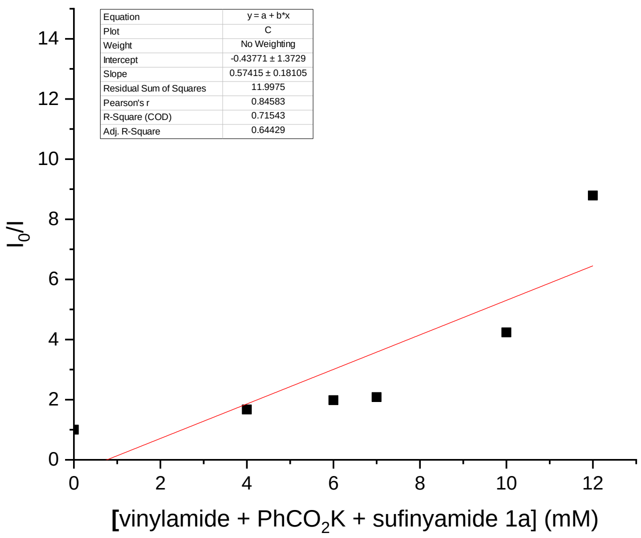

Supplementary Figure 24: Plotted ratio of fluorescence intensity with concentration of arylsulfinylamide **1a** and vinylamide and $PhCO_2K$

Cyclic voltammetry experiment

Cyclic voltammetry experiment was performed in a three-electrode cell connected under nitrogen at room temperature. A working glass carbon electrode, platinum wire counter electrode and Ag/AgCl reference electrode were employed. Anhydrous degassed MeCN (10 mL) containing 1.0 mmol $^{n}Bu_4NBF_4$ was poured into the electrochemical cell in all experiments. The concentration of compounds is 3 mM. The scan rate is 1V/s. All cyclic voltammograms were normalized by adding 1.0 equiv freshly sublimed ferrocene and collecting a new voltammogram. The ½ wave penitential of the Fc/Fc+ peak was identified and set to 0.0 V. Data was analyzed using MATLAB by subtracting a background current prior to identifying the maximum current (Cp) and determining the potential (Ep/2) at half this value (Cp/2). The reductive potential of (*E*)-*N*-(prop-1-en-1-yl)benzamide was calculated as $E_{1/2=}$ 1.05 V vs Fc/Fc^+. The reductive potential of arylsulfinylamide ammonium salt **3** was calculated as $E_{1/2=}$ 0.17 vs Fc/Fc^+.

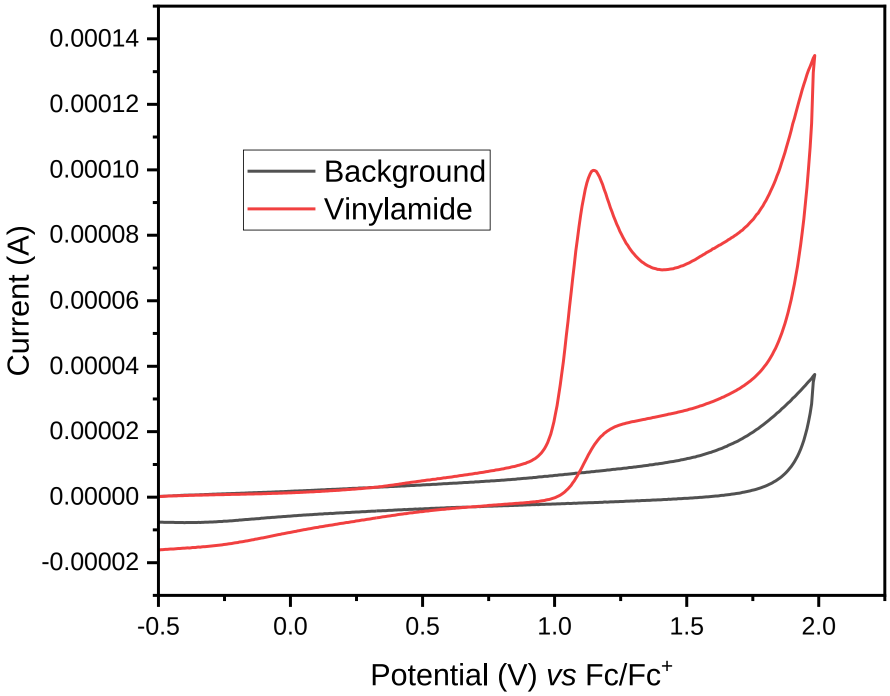

Supplementary Figure 25: Cyclic voltammetry of (*E*)-*N*-(prop-1-en-1-yl)benzamide

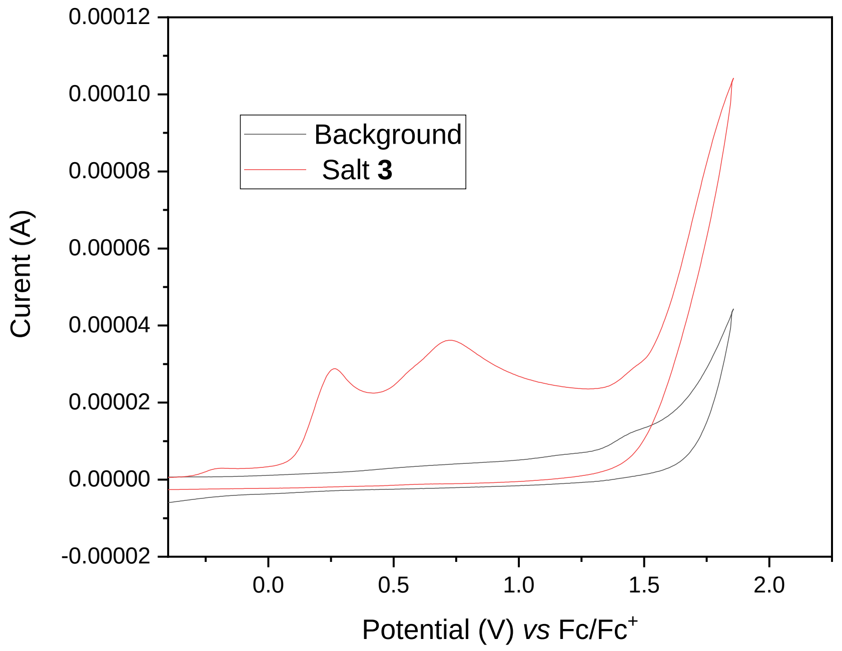

Supplementary Figure 26: Cyclic voltammetry of arylsulfinylamide ammonium salt **3**

Conclusion: The reductive potential of vinylamide was calculated as $E_{1/2=}$ 1.05 V *vs* Fc/Fc$^+$ ($E_{1/2=}$ 1.45 V *vs* SCE). The reductive potential of arylsulfinylamide ammonium salt **3** was calculated as $E_{1/2=}$ 0.17 *vs* Fc/Fc$^+$ ($E_{1/2=}$ 0.57 V *vs* SCE). [3]

Bisulfite detection test

The reaction of racemic *N*-(*p*-tolylsulfinyl)butyramide **1a** with *trans*-anethole was carried out under the standard conditions. A commercially available colorimetric strip test (MQuant Sulite test Merck) was performed on the crude reaction mixture. The bisulfite (HSO_3^-) ion was detected in the crude mixture. A control experiment excluding alkene tested negative for bisulfite by the same analysis. This result supports the hypothesis that S(IV) is formed indirectly via sulfinamide degradation following Smiles-Truce rearrangement.

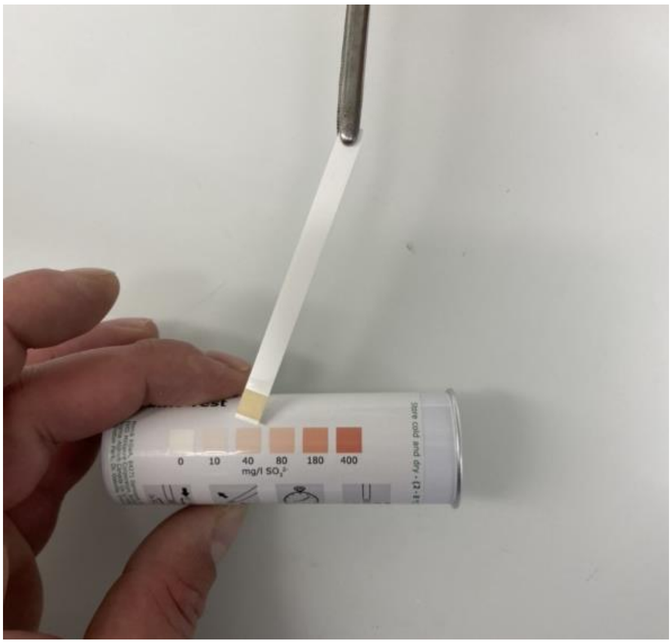

Supplementary Figure 27: Result of sulfite colorimetric test strips in the crude reaction mixture

Experiment to explain the observed stereoselectivity

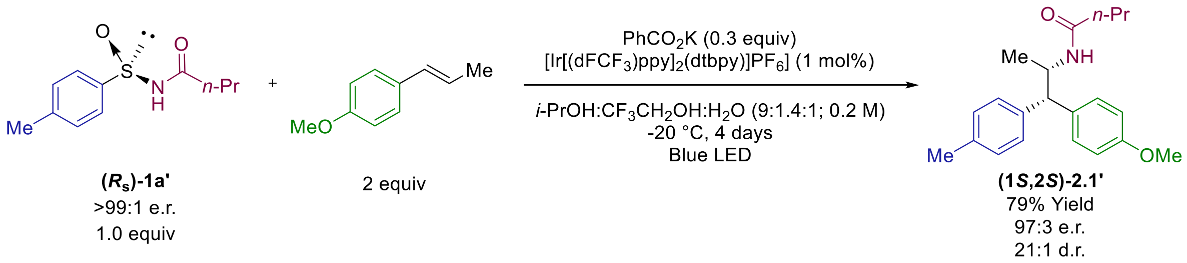

Supplementary Figure 28: Influence of the absolute configuration of the arylsulfinylamides. The use of the opposite enantiomer in the sulfinyl moiety in **1** results in the formation of the opposite enantiomer of the product **2**. The reaction is thus, stereospecific.

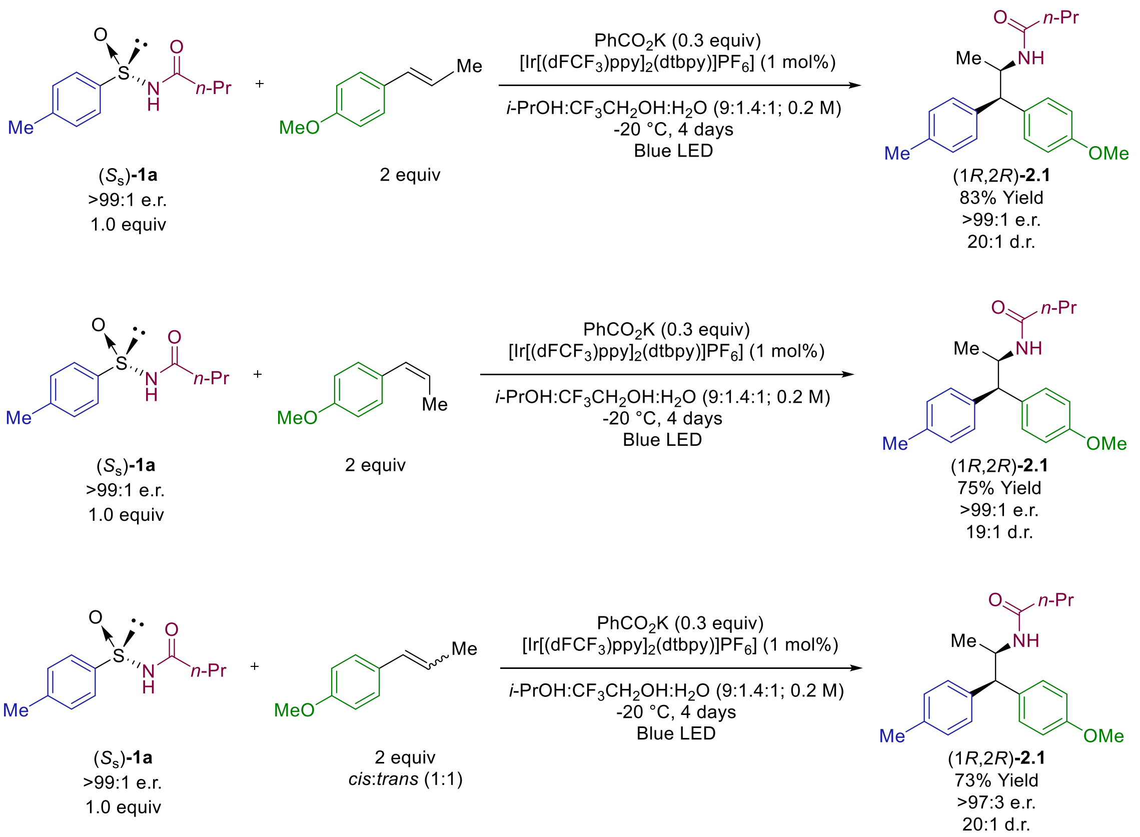

Supplementary Figure 29: Experiments in the presence of *E*-, *Z*- and a 1:1 mixture of *E*- and *Z*-anethole. In all three cases, a similar outcome is observed thus demonstrating that the geometry of the olefin does not affect the stereochemical outcome of the reaction.

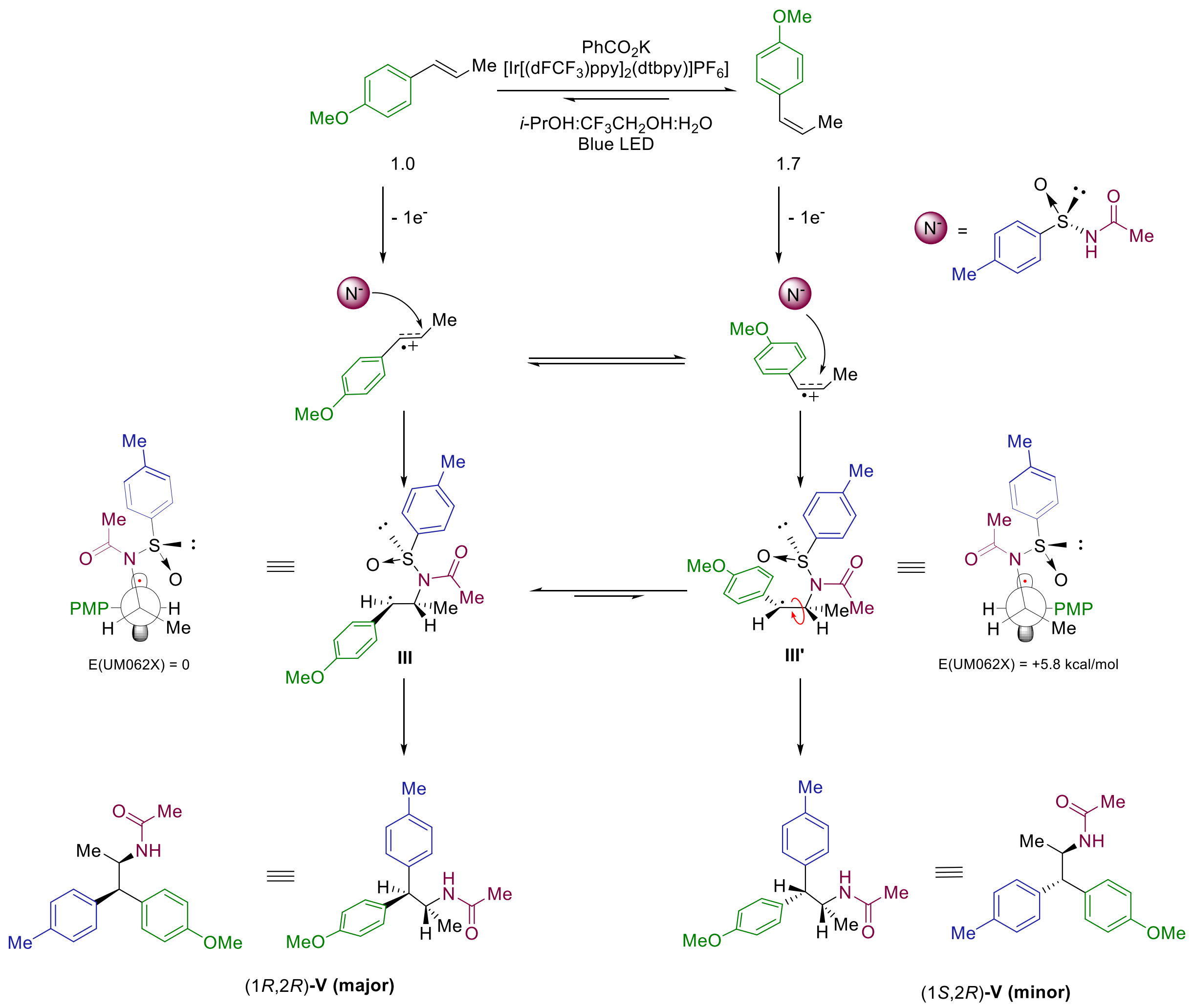

Supplementary Figure 30: Detailed proposal to explain the stereochemical outcome of the reaction including the potential isomerization of *E*- and *Z*-anethole and the interconversion of intermediates **III** and **III'** under the reaction conditions.

__Characterization data of *N*-sulfinylamides__

(*S*)-Benzenesulfinamide

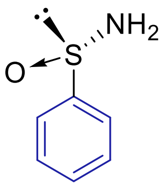

White solid, 98% yield, m.p. = 118-119 °C. ^{1}H NMR (400 MHz, $CDCl_3$) δ 7.78 – 7.72 (m, 2H), 7.55 – 7.48 (m, 3H), 4.34 (br s, 2H). ^{13}C NMR (101 MHz, $CDCl_3$) δ 146.5, 131.1, 128.9 (2C), 125.4 (2C). IR (film) ν (cm^{-1}) 3275, 3056, 1473, 1443, 1013. HR-MS (ESI) *m/z* calcd for C_6H_8ONS 142.03211, found 142.03217 [$M+H^+$]. $[\alpha]_D^{25}$ = +68.9 (*c* 0.14, EtOAc). HPLC conditions: AD-H column, *n*-hexane:*i*-PrOH = 95:5, flow rate = 0.5 mL·min^{-1}, t_R = 37.6 min (major), t_R = 40.5 min (minor).

(*S*)-4-Bromobenzenesulfinamide

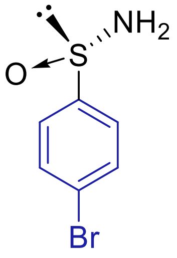

White solid, 92% yield, m.p. = 154-155°C. ^{1}H NMR (400 MHz, $CDCl_3$) δ 7.64 (d, *J* = 8.8 Hz, 2H), 7.60 (d, *J* = 8.9 Hz, 2H), 4.34 (br s, 2H). ^{13}C NMR (101 MHz, $CDCl_3$) δ 145.5, 132.1 (2C), 127.2 (2C), 125.9. IR (film) ν (cm^{-1}) 3270, 3062, 1572, 1462, 995. HR-MS (ESI) *m/z* calcd for C_6H_7ONSBr 219.94262, found 219.94260 [$M+H^+$]. $[\alpha]_D^{25}$ = +52.1 (*c* 0.14, EtOAc). HPLC conditions: AD-H column, *n*-hexane:*i*-PrOH = 95:5, flow rate = 0.5 mL·min^{-1}, t_R = 26.4 min (major), t_R = 29.8 min (minor).

(*S*)-4-Fluorobenzenesulfinamide

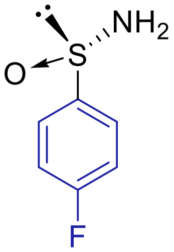

Obtained from glucose sulfinate derivative. White solid, 97% yield, m.p. = 161-162 °C. ^{1}H NMR (400 MHz, $CDCl_3$) δ 7.81 – 7.63 (m, 2H), 7.19 (t, *J* = 8.6 Hz, 2H), 4.47 (br s, 2H). ^{13}C NMR (101 MHz, $CDCl_3$) δ 164.4 (d, *J* = 251.6 Hz), 142.0 (s), 127.8 (d, *J* = 9.0 Hz, 2C), 116.1 (d, *J* = 22.5 Hz, 2C). ^{19}F NMR (376 MHz, $CDCl_3$) δ -109. IR (film) ν (cm^{-1}) 3265, 3073, 1582, 1473, 1223, 998. HR-MS (ESI) *m/z* calcd for C_6H_7ONFS 160.02269, found 160.02272 [$M+H^+$]. $[\alpha]_D^{25}$ = +88.7 (*c* 0.14, EtOAc). HPLC conditions: OD-H column, *n*-hexane:*i*-PrOH = 95:5, flow rate = 1.0 mL·min^{-1}, t_R = 33.4 min (minor), t_R = 56.6 min (major).

4-(Trifluoromethyl)benzenesulfinamide

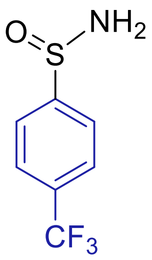

White solid, 88% yield, m.p. = 125-126 °C. ^{1}H NMR (400 MHz, $CDCl_3$) δ 7.89 (d, *J* = 8.1 Hz, 2H), 7.77 (d, *J* = 8.2 Hz, 2H), 4.46 (br s, 2H). ^{13}C NMR (101 MHz, $CDCl_3$) δ 150.4, 133.2 (q, *J* = 32.7 Hz), 126.2 (2C), 125.9 (q, *J* = 3.7 Hz, 2C), 123.5 (q, *J* = 272.6 Hz). ^{19}F NMR (376 MHz, $CDCl_3$) δ -62.8 (s). IR (film) ν (cm^{-1}) 3270, 3062, 1564, 1316, 1028. HR-MS (ESI) *m/z* calcd for $C_7H_5ONF_3S$ 208.00494, found 208.00481 [$M-H^+$].

(*S*)-4-Methoxybenzenesulfinamide

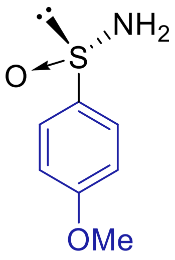

White solid, 91% yield, m.p. = 129-130 °C. ^{1}H NMR (400 MHz, $CDCl_3$) δ 7.78 – 7.55 (m, 2H), 7.11 – 6.89 (m, 2H), 4.26 (br s, 2H), 3.86 (s, 3H). ^{13}C NMR (101 MHz, $CDCl_3$) δ 161.9, 137.8, 127.1 (2C), 114.3 (2C), 55.5. IR (film) ν (cm^{-1}) 3275, 3062, 1575, 1487, 1241, 1000. HR-MS (ESI) *m/z* calcd for $C_7H_{10}O_2NS$ 172.04268, found 172.04257 [$M+H^+$]. $[\alpha]_D^{25}$ = +73.8 (*c* 0.14, EtOAc). HPLC conditions: OD-H column, *n*-hexane:*i*-PrOH = 90:10, flow rate = 1.0 mL·min^{-1}, t_R = 22.0 min (minor), t_R = 35.9 min (major).

3-Methoxybenzenesulfinamide

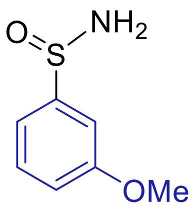

White solid, 91% yield, m.p. = 136-137 °C. ^{1}H NMR (500 MHz, $CDCl_3$) δ 7.36 (t, *J* = 7.9 Hz, 1H), 7.29 – 7.24 (m, 2H), 7.01 – 6.94 (m, 1H), 4.34 (br s, 2H), 3.81 (s, 3H). ^{13}C NMR (126 MHz, $CDCl_3$) δ 160.0, 147.9, 129.9, 117.6, 117.5, 109.8, 55.5. IR (film) ν (cm^{-1}) 3265, 3088, 1591, 1470.5, 1233, 1030. HR-MS (ESI) *m/z* calcd for $C_7H_{10}O_2NS$ 172.04268, found 172.04270 [$M+H^+$].

3-Bromobenzenesulfinamide

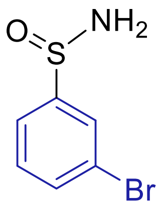

White solid, 85% yield, m.p. = 197-198 °C. ^{1}H NMR (500 MHz, $CDCl_3$) δ 7.91 (t, *J* = 1.7 Hz, 1H), 7.71 – 7.65 (m, 1H), 7.66 – 7.59 (m, 1H), 7.39 (t, *J* = 7.9 Hz, 1H), 4.37 (br s, 2H). ^{13}C NMR (126 MHz, $CDCl_3$) δ 148.6, 134.2,

130.4, 128.6, 124.2, 123.1. IR (film) ν (cm^{-1}) 3297, 3067, 1560, 1447, 1403, 1015. HR-MS (ESI) *m/z* calcd for C_6H_7ONSBr 219.94262, found 219.94230 [$M+H^+$].

(*S*)-2-Methylbenzenesulfinamide

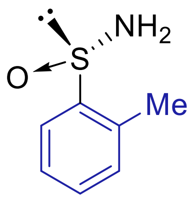

White solid, 84% yield, m.p. = 107-108 °C. ^1H NMR (400 MHz, $CDCl_3$) δ 8.08 – 7.96 (m, 1H), 7.49 – 7.38 (m, 2H), 7.28 – 7.18 (m, 1H), 4.14 (br s, 2H), 2.49 (s, 3H). ^{13}C NMR (101 MHz, $CDCl_3$) δ 144.3, 135.8, 131.2, 130.9, 126.6, 122.7, 18.6. IR (film) ν (cm^{-1}) 3327, 3208, 2584, 1738, 1467, 1033. HR-MS (ESI) *m/z* calcd for $C_7H_{10}ONS$ 156.04776, found 156.04777 [$M+H^+$]. $[\alpha]_D^{25}$ = +193.7 (*c* 0.14, EtOAc). HPLC conditions: AD-H column, *n*-hexane:*i*-PrOH = 95:5, flow rate = 0.5 mL·min^{-1}, t_R =37.6 min (minor), t_R = 40.2 min (major).

(*S*)-2-Bromobenzenesulfinamide

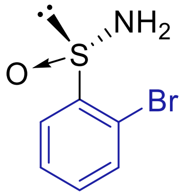

White solid, 86% yield, m.p. = 146-147 °C. ^1H NMR (400 MHz, $CDCl_3$) δ 8.03 (dd, *J* = 7.8, 1.6 Hz, 1H), 7.61 (dd, *J* = 7.9, 1.0 Hz, 1H), 7.54 (td, *J* = 7.7, 1.1 Hz, 1H), 7.38 (td, *J* = 7.7, 1.7 Hz, 1H), 4.23 (br s, 2H). ^{13}C NMR (101 MHz, $CDCl_3$) δ 146.0, 133.6, 132.7, 128.2, 125.1, 120.3. IR (film) ν (cm^{-1}) 3344, 3223, 1565, 14444, 1000, 1014. HR-MS (ESI) *m/z* calcd for C_6H_7ONSBr 219.94262, found 219.94273 [$M+H^+$]. $[\alpha]_D^{25}$ = +217.5 (*c* 0.14, EtOAc). HPLC conditions: AD-H column, *n*-hexane:*i*-PrOH = 95:5, flow rate = 0.5 mL·min^{-1}, t_R = 28.7 min (minor), t_R = 39.3 min (major).

(*S*)-5-chlorothiophene-2-sulfinamide[4]

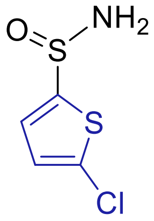

White solid, 65% yield, m.p. = 107-109 °C. ^1H NMR (400 MHz, Acetone-d_6) δ 7.21 (d, *J* = 3.9 Hz, 1H), 7.11 (d, *J* = 3.9 Hz, 1H), 6.01 (s, 2H). ^{13}C NMR (101 MHz, Acetone-d_6) δ 134.9, 129.6, 129.6, 128.7. IR (film) ν (cm^{-1}) 3083, 1770, 1415, 1033, 1020, 884. HR-MS (ESI) *m/z* calcd for $C_4H_5ONS_2Cl$ 181.94956, found 181.94960 [$M+H^+$].

Characterization data of Arylsulfinylamides (1a-1t)

(*S*)-*N*-(*p*-Tolylsulfinyl)butyramide (1a)

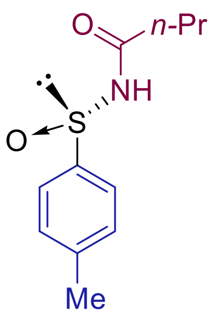

White solid, 86% yield, m.p. = 137-139 °C. ^{1}H NMR (400 MHz, $CDCl_3$) δ 7.60 (d, *J* = 8.3 Hz, 2H), 7.41 (s, 1H), 7.36 (d, *J* = 8.0 Hz, 2H), 2.44 (s, 3H), 2.37 (br s, 2H), 1.77 – 1.68 (m, 2H), 0.98 (t, *J* = 7.4 Hz, 3H). ^{13}C NMR (101 MHz, $CDCl_3$) δ 174.1, 142.6, 140.4, 130.6 (2C), 124.9 (2C), 38.1, 21.6, 18.5, 13.7. IR (film) ν (cm^{-1}) 3082, 1692, 1421, 1089, 804. HR-MS (ESI) *m/z* calcd for $C_{11}H_{14}O_2NS$ 224.07507, found 224.07492 [M-H$^+$]. $[\alpha]_D^{25}$ = +96.5 (*c* 0.14, EtOAc). HPLC conditions: ODH column, *n*-hexane:EtOH = 90:10, flow rate = 0.5 mL·min^{-1}, t_R = 14.6 min (minor), t_R = 19.7 min (major).

(*R*)-*N*-(*p*-Tolylsulfinyl)butyramide (1a')

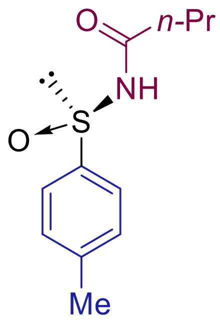

White solid, 80% yield $[\alpha]_D^{25}$ = -100.3 (*c* 0.14, EtOAc). HPLC conditions: ODH column, *n*-hexane:EtOH = 90:10, flow rate = 0.5 mL·min^{-1}, t_R = 13.2 min (major), t_R = 17.6 min (minor).

(*S*)-2-(4-Methoxyphenyl)-*N*-(*p*-tolylsulfinyl)acetamide (1b)

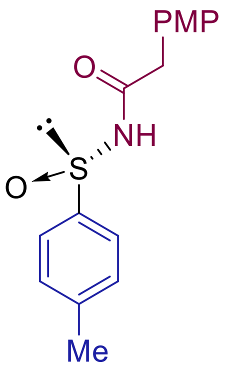

White solid, 64% yield, m.p. = 146-148 °C. ^{1}H NMR (400 MHz, $CDCl_3$) δ 7.49 (d, *J* = 8.3 Hz, 2H), 7.35 – 7.27 (m, 3H), 7.15 (d, *J* = 8.5 Hz, 2H), 6.86 (d, *J* = 8.7 Hz, 2H), 3.79 (s, 3H), 3.68 (brs, 2H), 2.41 (s, 3H). ^{13}C NMR (101 MHz, $CDCl_3$) δ 172.1, 159.2, 142.7, 140.5, 130.6 (2C), 130.2 (2C), 125.1, 124.8 (2C), 114.6 (2C), 55.4, 42.8, 21.6. IR (film) ν (cm^{-1}) 3168, 2931, 1737, 1682, 1412, 1091, 814. HR-MS (ESI) *m/z* calcd for $C_{16}H_{16}O_3NS$ 302.08564, found 302.08557 [M-H$^+$]. $[\alpha]_D^{25}$ = +90.1 (*c* 0.14, EtOAc). HPLC conditions: ODH column, *n*-hexane:EtOH = 90:10, flow rate = 0.5 mL·min^{-1}, t_R = 28.1 min (minor), t_R = 43.2 min (major).

(*R*)-2-Phenyl-*N*-[(*S*)-*p*-tolylsulfinyl]butanamide (1c)

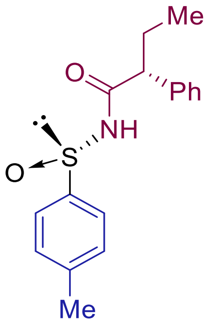

White solid, 44% yield, m.p. = 145-147 °C. ^{1}H NMR (400 MHz, $CDCl_3$) δ 7.49 – 7.18 (m, 10H), 3.45 (br s, 1H), 2.39 (s, 3H), 2.26 – 2.09 (m, 1H), 1.90 – 1.74 (m, 1H), 0.90 (t, *J* = 7.4 Hz, 3H). ^{13}C NMR (101 MHz, $CDCl_3$) δ 174.1, 142.4, 140.8, 138.5, 130.0 (2C), 129.1 (2C), 128.2 (2C), 127.8, 124.7 (2C), 54.9, 26.1, 21.5, 12.1. IR (film) ν (cm^{-1}) 3187, 2926, 1737, 1674, 1413, 1066, 813. HR-MS (ESI) *m/z* calcd for $C_{17}H_{18}O_2NS$ 300.10637, found 300.10619 [M-H$^+$]. $[\alpha]_D^{25}$ = +109.1 (*c* 0.14, EtOAc). HPLC conditions: ODH column, *n*-hexane:EtOH = 90:10, flow rate = 0.5 mL·min^{-1}, t_R = 12.5 min (minor), t_R = 17.1 min (major).

(*S*)-*N*-(*p*-Tolylsulfinyl)cyclohexanecarboxamide (1d)

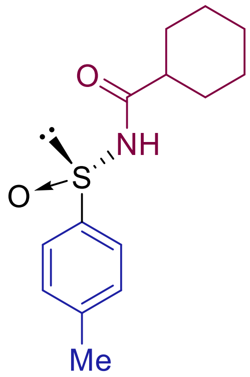

White solid, 80% yield, m.p. = 141-143 °C. ^{1}H NMR (400 MHz, $CDCl_3$) δ 7.77 (br s, 1H) 7.55 (d, *J* = 8.2 Hz, 2H), 7.32 (d, *J* = 8.1 Hz, 2H), 2.42 (s, 3H), 2.26 (br s, 1H), 1.92 – 1.55 (m, 5H), 1.55 – 1.38 (m, 2H), 1.30 – 1.18 (m, 3H). ^{13}C NMR (101 MHz, $CDCl_3$) δ 176.6, 142.7, 140.8, 130.2 (2C), 124.8 (2C), 45.0, 29.4, 29.1, 25.6, 25.5, 25.4, 21.6. IR (film) ν (cm^{-1}) 3167, 2931, 1737, 1682, 1412, 1091, 814. HR-MS (ESI) *m/z* calcd for $C_{14}H_{18}O_2NS$ 264.10637, found 264.10615 [M-H$^+$]. $[\alpha]_D^{25}$ = +82.7 (*c* 0.14, EtOAc). HPLC conditions: ODH column, *n*-hexane:EtOH = 90:10, flow rate = 0.5 mL·min^{-1}, t_R = 9.5 min (major), t_R = 16.2 min (minor).

Ethyl (*S*)-4-oxo-4-[(*p*-tolylsulfinyl)amino]butanoate (1e)

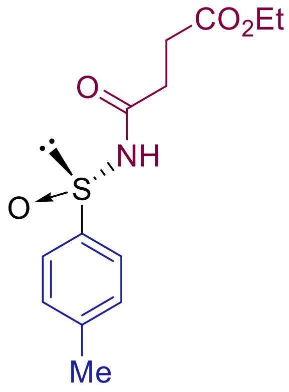

White solid, 43% yield, m.p. = 119-121 °C. ^{1}H NMR (400 MHz, Acetone-*d*$_6$) δ 9.81 (br s, 1H), 7.62 (d, *J* = 8.3 Hz, 2H), 7.41 (d, *J* = 7.9 Hz, 2H), 4.09 (q, *J* = 7.1 Hz, 2H), 2.64 – 2.52 (m, 4H), 2.42 (s, 3H), 1.21 (t, *J* = 7.1 Hz, 3H). ^{13}C NMR (101 MHz, Acetone-*d*$_6$) δ 173.4, 172.7, 142.8, 142.4, 130.6 (2C), 125.7 (2C), 60.9, 31.2, 29.1, 21.3, 14.5. IR (film) ν (cm^{-1}) 3136, 1733, 1474, 1052, 807. HR-MS (ESI) *m/z* calcd for $C_{13}H_{16}O_4NS$ 282.08055, found 282.08035 [M-H$^+$]. $[\alpha]_D^{25}$ = +49.6 (*c* 0.14, EtOAc). HPLC conditions: ODH column, *n*-hexane:EtOH = 90:10, flow rate = 0.5 mL·min^{-1}, t_R = 22.0 min (minor), t_R = 34.3 min (major).

(*S*)-3-[(*tert*-Butyldimethylsilyl)oxy]-*N*-[(*S*)-*p*-tolylsulfinyl]butanamide (1f)

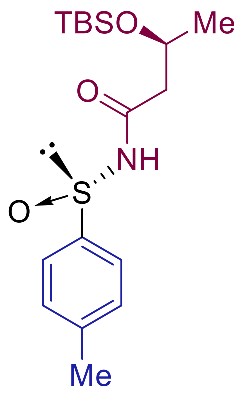

Oil, 52% yield. ^{1}H NMR (400 MHz, Acetone-d_6) δ 9.59 (br s, 1H), 7.62 (d, *J* = 8.2 Hz, 2H), 7.41 (d, *J* = 7.9 Hz, 2H), 4.26 (h, *J* = 6.1 Hz, 1H), 2.49 (d, *J* = 5.4 Hz, 2H), 2.42 (s, 3H), 1.19 (d, *J* = 6.1 Hz, 3H), 0.84 (s, 9H), 0.08 (s, 3H), 0.07 (s, 3H). ^{13}C NMR (101 MHz, Acetone-d_6) δ 172.5, 142.9, 142.6, 130.7 (2C), 125.7 (2C), 66.7, 46.8, 26.3 (3C), 24.1, 21.4, 18.6, -4.4, -4.6. IR (film) ν (cm^{-1}) 2929, 1708, 1376, 1001, 832. HR-MS (ESI) *m/z* calcd for $C_{17}H_{28}O_3NSSi$ 354.15646, found 354.15639 [M-H$^+$]. $[\alpha]_D^{25}$ = +39.0 (*c* 0.14, EtOAc). HPLC conditions: ODH column, *n*-hexane:EtOH = 90:10, flow rate = 0.5 mL·min^{-1}, t_R = 9.3 min (minor), t_R = 10.6 min (major).

(*S*)-*N*- (*p*-Tolylsulfinyl)benzamide (1g)

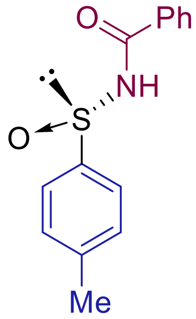

White solid, 60% yield, m.p. = 125-127 °C. ^{1}H NMR (400 MHz, $CDCl_3$) δ 8.46 (s, 1H), 7.84 – 7.77 (m, 2H), 7.66 (d, *J* = 8.3 Hz, 2H), 7.61 – 7.52 (m, 1H), 7.49 – 7.40 (m, 2H), 7.35 (d, *J* = 7.9 Hz, 2H), 2.44 (s, 3H). ^{13}C NMR (101 MHz, $CDCl_3$) δ 167.6, 142.7, 140.8, 133.3, 131.8, 130.2 (2C), 128.9 (2C), 128.2 (2C), 125.0 (2C), 21.6. IR (film) ν (cm^{-1}) 3227, 2954, 1737, 1649, 1385, 1093, 809. HR-MS (ESI) *m/z* calcd for $C_{14}H_{12}O_2NS$ 258.05942, found 258.05930 [M-H$^+$]. $[\alpha]_D^{25}$ = +201.2 (*c* 0.14, EtOAc). HPLC conditions: ODH column, *n*-hexane:EtOH = 90:10, flow rate = 0.5 mL·min^{-1}, t_R = 23.7 min (minor), t_R = 29.4 min (major).

(*S*)-*N*- (*p*-Tolylsulfinyl) furan-2-carboxamide (1h)

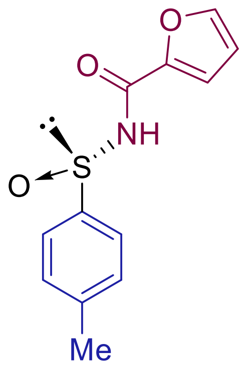

White solid, 62% yield, m.p. = 159-161 °C. ^{1}H NMR (400 MHz, $CDCl_3$) δ 8.12 (s, 1H), 7.70 (d, *J* = 8.3 Hz, 2H), 7.46 (dd, *J* = 1.7, 0.8 Hz, 1H), 7.39 (d, *J* = 7.9 Hz, 2H), 7.33 (dd, *J* = 3.6, 0.7 Hz, 1H), 6.56 (dd, *J* = 3.6, 1.7 Hz, 1H), 2.45 (s, 3H). ^{13}C NMR (101 MHz, $CDCl_3$) δ 157.5, 145.8, 145.6, 143.0, 140.8, 130.4 (2C), 124.9 (2C), 118.0, 113.1, 21.7. IR (film) ν (cm^{-1}) 3132, 1736, 1662, 1413, 1167, 806. HR-MS (ESI) *m/z* calcd for $C_{12}H_{10}O_3NS$ 248.03869, found 248.03851 [M-H$^+$]. $[\alpha]_D^{25}$ = +148.0 (*c* 0.14, EtOAc). HPLC conditions: ODH column, *n*-hexane:EtOH = 90:10, flow rate = 0.5 mL·min^{-1}, t_R = 27.5 min (major), t_R = 32.4 min (minor).

tert-Butyl (_S_)-(_p_-tolylsulfinyl)carbamate (1i)

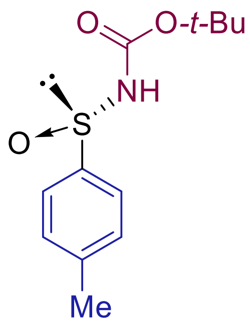

White solid, 62% yield, m.p. = 107-109 °C. ^{1}H NMR (400 MHz, $CDCl_3$) δ 7.63 (d, *J* = 8.3 Hz, 2H), 7.34 (d, *J* = 7.9 Hz, 2H), 6.57 (s, 1H), 2.43 (s, 3H), 1.52 (s, 9H). ^{13}C NMR (101 MHz, $CDCl_3$) δ 152.5, 142.5, 140.7, 130.0 (2C), 124.7 (2C), 83.7, 28.1 (3C), 21.4. IR (film) ν (cm^{-1}) 3132, 2924, 1736, 1662, 1413, 1065, 806. HR-MS (ESI) *m/z* calcd for $C_{12}H_{16}O_3NS$ 254.08564, found 254.08555 [$M-H^+$]. $[\alpha]_D^{25}$ = +162.3 (*c* 0.14, EtOAc). HPLC conditions: ODH column, *n*-hexane:EtOH = 90:10, flow rate = 0.5 mL·min^{-1}, t_R = 10.6 min (minor), t_R = 27.5 min (major).

(_S_)-_N_-(phenylsulfinyl)butyramide (1j)

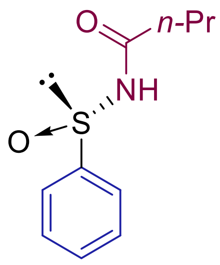

White solid, 87% yield, m.p. = 61-63 °C. ^{1}H NMR (400 MHz, $CDCl_3$) δ 7.84 (br s, 1H), 7.74 – 7.67 (m, 2H), 7.60 – 7.49 (m, 3H), 2.37 (br s, 2H), 1.76 – 1.67 (m, 2H), 0.98 (t, *J* = 7.4 Hz, 3H). ^{13}C NMR (101 MHz, $CDCl_3$) δ 173.8, 143.8, 132.1, 129.6 (2C), 124.9, 124.8, 38.0 18.5, 13.7. IR (film) ν (cm^{-1}) 3062, 2359, 1658, 1429, 1057, 813. HR-MS (ESI) *m/z* calcd for $C_{10}H_{12}O_2$ NS 210.05942, found 210.05934 [$M-H^+$]. $[\alpha]_D^{25}$ = +43.0 (*c* 0.14, EtOAc). HPLC conditions: ODH column, *n*-hexane:EtOH = 90:10, flow rate = 0.5 mL·min^{-1}, t_R = 14.5 min (minor), t_R = 16.7 min (major).

(_S_)-_N_-[(4-bromophenyl)sulfinyl]butyramide (1k)

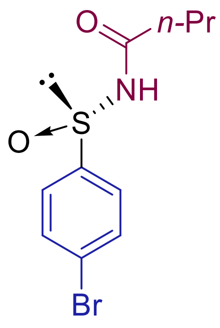

White solid, 69% yield, m.p. = 141-143 °C. ^{1}H NMR (400 MHz, $CDCl_3$) δ 7.80 -7.60 (m, 3H), 7.56 (d, *J* = 8.6 Hz, 2H), 2.37 (br s, 2H), 1.72 (h, *J* = 7.4 Hz, 2H), 0.98 (t, *J* = 7.4 Hz, 3H). ^{13}C NMR (101 MHz, $CDCl_3$) δ 173.8, 142.9, 132.8 (2C), 126.9, 126.6 (2C), 37.9, 18.4, 13.7. IR (film) ν (cm^{-1}) 3082, 1685, 1403, 1051, 811. HR-MS (ESI) *m/z* calcd for $C_{10}H_{11}O_2NBrS$ 287.96994, found 287.96992 [$M-H^+$]. $[\alpha]_D^{25}$ = +153.8 (*c* 0.14, EtOAc). HPLC conditions: ODH column, *n*-hexane:EtOH = 90:10, flow rate = 0.5 mL·min^{-1}, t_R = 17.5 min (minor), t_R = 24.8 min (major).

(*S*)-*N*-[(4-fluorophenyl)sulfinyl]butyramide (1l)

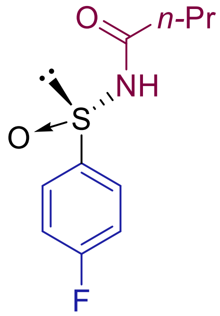

White solid, 89% yield, m.p. = 136-137 °C. ^{1}H NMR (400 MHz, Acetone-d_6) δ 9.90 (br s, 1H), 7.95 – 7.69 (m, 2H), 7.42 – 7.31 (m, 2H), 2.33 (t, *J* = 7.3 Hz, 2H), 1.64 (h, *J* = 7.4 Hz, 2H), 0.92 (t, *J* = 7.4 Hz, 3H). ^{13}C NMR (101 MHz, Acetone-d_6) δ 174.4, 165.4 (d, *J* = 24.9 Hz), 141.1 (d, *J* = 2.9 Hz), 128.4 (d, *J* = 9.2 Hz, 2C), 117.0 (d, *J* = 22.9 Hz, 2C), 38.3, 18.9, 13.8. ^{19}F NMR (377 MHz, Acetone-d_6) δ -110.7. IR (film) ν (cm^{-1}) 3108, 1702, 1320, 1052, 817. HR-MS (ESI) *m/z* calcd for $C_{10}H_{13}O_2NFS$ 230.06455, found 230.06466 [M+H$^+$]. $[\alpha]_D^{25}$ = +101.2 (*c* 0.14, EtOAc). HPLC conditions: ODH column, *n*-hexane:EtOH = 90:10, flow rate = 0.5 mL·min^{-1}, t_R = 14.8 min (minor), t_R = 17.7 min (major).

(*S*)-3-[(*tert*-Butyldimethylsilyl)oxy]-*N*-[(*S*)-(4-trifluoromethylphenyl)sulfinyl]butanamide (1m)

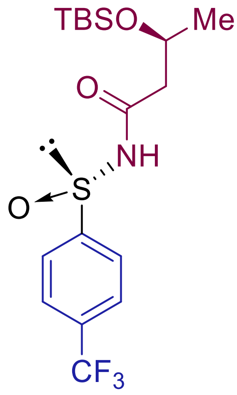

Obtained by separation of diasteroisomer on column chromatography, using general procedure **Step 2** on the racemic sulfinamine. Oil, 39% yield. ^{1}H NMR (400 MHz, Acetone-d_6) δ 9.87 (br s, 1H), 7.99 (d, *J* = 8.6 Hz, 2H), 7.96 (d, *J* = 8.7 Hz, 2H), 4.35 (dq, *J* = 6.1, 12.2 Hz, 1H), 2.50 (d, *J* = 5.8 Hz, 2H), 1.20 (d, *J* = 6.2 Hz, 3H), 0.83 (s, 9H), 0.07 (s, 3H), 0.07 (s, 3H). ^{13}C NMR (101 MHz, $CDCl_3$) δ 171.6, 149.3, 132.6 (q, *J* = 32.5 Hz), 126.1 (q, *J* = 7.5, Hz), 125.9 (2C), 123.9 (q, *J* = 271.8 Hz, 2C), 65.6, 45.9, 25.3 (3C), 23.2, 17.6, -5.4, -5.6. ^{19}F NMR (377 MHz, Acetone-d_6) δ -63.4. IR (film) ν (cm^{-1}) 3213, 1671, 1322, 1126, 811. HR-MS (ESI) *m/z* calcd for $C_{17}H_{25}O_3NF_3SSi$ 408.12820, found 408.12833 [M-H$^+$]. $[\alpha]_D^{25}$ = +46.9 (*c* 0.14, EtOAc). HPLC conditions: ODH column, *n*-hexane:EtOH = 90:10, flow rate = 0.5 mL·min^{-1}, t_R = 9.8 min (major), t_R = 12.3 min (minor).

(*S*)-*N*-[(4-Methoxyphenyl)sulfinyl]butyramide (1n)

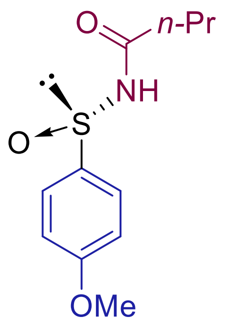

White solid, 60% yield, m.p. = 85-87 °C. ^{1}H NMR (400 MHz, $CDCl_3$) δ 8.39 (br s, 1H), 7.51 (d, *J* = 8.8 Hz, 2H), 6.97 (d, *J* = 8.9 Hz, 2H), 3.85 (s, 3H), 2.34 (br s, 2H), 1.68 (h, *J* = 7.4 Hz, 2H), 0.96 (t, *J* = 7.4 Hz, 3H). ^{13}C NMR (101 MHz, $CDCl_3$) δ 174.4, 162.5, 134.4, 126.6 (2C), 114.8 (2C), 55.6, 37.9, 18.4, 13.7. IR (film) ν (cm^{-1}) 3203, 2942, 1666, 1421, 1254 1076, 795. HR-MS (ESI) *m/z* calcd for $C_{11}H_{14}O_3NS$: 240.06999, found 240.06987 [M-H$^+$]. $[\alpha]_D^{25}$ = +85.6 (*c* 0.14, EtOAc). HPLC conditions: ODH column, *n*-hexane:EtOH = 90:10, flow rate = 0.5 mL·min^{-1}, t_R = 17.3 min (minor), t_R = 26.0 min (major).

(*S*)-3-[(*tert*-Butyldimethylsilyl)oxy]-*N*-[(*S*)-(3-methoxyphenyl)sulfinyl]butanamide (1o)

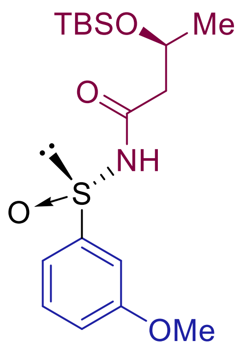

Obtained by separation of diasteroisomer on column chromatography, using general procedure **Step 2** on the racemic sulfinamine. Oil, 25% yield. ^{1}H NMR (400 MHz, Acetone-d_6) δ 9.75 (br s, 1H), 7.49 (dd, *J* = 11.8, 4.1 Hz, 1H), 7.30 – 7.24 (m, 2H), 7.12 (ddd, *J* = 8.2, 2.6, 0.9 Hz, 1H), 4.41 – 4.30 (m, 1H), 3.87 (s, 3H), 2.49 (d, *J* = 5.9 Hz, 2H), 1.20 (d, *J* = 6.2 Hz, 3H), 0.83 (s, 9H), 0.08 (s, 3H), 0.07 (s, 3H). ^{13}C NMR (101 MHz, Acetone-d_6) δ 172.6, 161.2, 146.7, 131.2, 118.4, 117.6, 110.4, 66.6, 56.0, 46.7, 26.2 (3C), 23.9, 18.5, -4.4, -4.7. IR (film) ν (cm^{-1}) 3448, 2358, 1739, 1379, 1159, 815. HR-MS (ESI) *m/z* calcd for $C_{17}H_{28}O_4NSSi$ 370.15138, found 370.15126 [M-H$^+$]. $[\alpha]_D^{25}$ = +38.1 (*c* 0.14, EtOAc). HPLC conditions: ODH column, *n*-hexane:EtOH = 90:10, flow rate = 0.5 mL·min^{-1}, t_R = 9.8 min (major), t_R = 11.4 min (minor).

(*S*)-3-[(*tert*-Butyldimethylsilyl)oxy]-*N*-[(*S*)-(3-bromophenyl))sulfinyl]butanamide (1p)

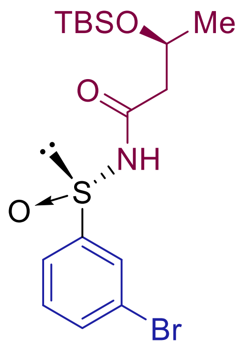

Obtained by separation of diasteroisomer on column chromatography, using general procedure **Step 2** on the racemic sulfinamine. Oil, 29% yield. ^{1}H NMR (400 MHz, Acetone-d_6) δ 9.85 (br s, 1H), 7.89 (t, *J* = 1.8 Hz, 1H), 7.79 – 7.70 (m, 2H), 7.56 (t, *J* = 7.9 Hz, 1H), 4.39 – 4.30 (m, 1H), 2.50 (d, *J* = 5.8 Hz, 2H), 1.20 (d, *J* = 6.2 Hz, 3H), 0.85 (s, 9H), 0.08 (s, 3H), 0.07 (s, 3H). ^{13}C NMR (101 MHz, Acetone-d_6) δ 172.6, 148.0, 135.4, 132.2, 128.5, 124.9, 123.6, 66.6, 46.9, 26.3 (3C), 24.2, 18.6, -4.4, -4.6. IR (film) ν (cm^{-1}) 3166, 2932, 1738, 1410, 1095, 809. HR-MS (ESI) *m/z* calcd for $C_{16}H_{25}O_3NBrSSi$ 418.05133, found 418.05148 [M-H$^+$]. $[\alpha]_D^{25}$ = +27.9 (*c* 0.14, EtOAc). HPLC conditions: ODH column, *n*-hexane:EtOH = 90:10, flow rate = 0.5 mL·min^{-1}, t_R = 14.5 min (major), t_R = 19.5 min (minor).

(*S*)-*N*-(*o*-Tolylsulfinyl)butyramide (1q)

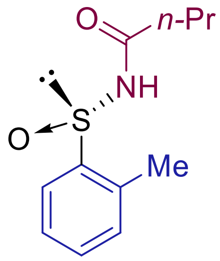

White solid, 69% yield, m.p. = 92-94 °C. ^{1}H NMR (400 MHz, $CDCl_3$) δ 7.99 (d, *J* = 7.2 Hz, 1H), 7.52 – 7.31 (m, 3H), 7.26 – 7.23 (m, 1H), 2.38 (s, 3H), 2.38 – 2.35 (m, 2H), 1.87 – 1.60 (m, 2H), 0.97 (t, *J* = 7.3 Hz, 3H). ^{13}C NMR (101 MHz, $CDCl_3$) δ 173.4, 141.5, 135.6, 132.1, 131.4, 127.2, 123.8, 38.1, 18.5, 18.3, 13.8. IR (film) ν (cm^{-1}) 3082, 1692, 1421, 1089, 804. HR-MS (ESI) *m/z* calcd for $C_{11}H_{14}O_2NS$ 224.07501, found 224.07492 [M-H$^+$]. $[\alpha]_D^{25}$ = +127.1 (*c* 0.14, EtOAc). HPLC conditions: ODH column, *n*-hexane:EtOH = 90:10, flow rate = 0.5 mL·min^{-1}, t_R = 13.1 min (minor), t_R = 19.8 min (major).

(*S*)-*N*-[(*o*-Bromophenyl)sulfinyl]butyramide (1r)

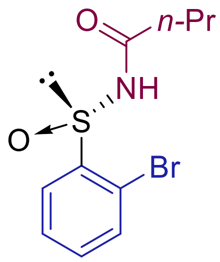

White solid, 80% yield, m.p. = 90-92 °C. ^{1}H NMR (400 MHz, $CDCl_3$) δ 8.18 – 7.92 (m, 2H), 7.62 (dd, *J* = 7.9, 1.1 Hz, 1H), 7.54 (t, *J* = 7.6 Hz, 1H), 7.42 (td, *J* = 7.7, 1.7 Hz, 1H), 2.36 (br s, 2H), 1.75 – 1.65 (m, 2H), 0.96 (t, *J* = 7.5 Hz, 3H). ^{13}C NMR (101 MHz, $CDCl_3$) δ 178.3, 142.6, 133.8, 133.5, 128.4, 126.8, 120.1, 37.0, 18.5, 13.8. IR (film) ν (cm^{-1}) 3082, 1685, 1403, 1051, 811. HR-MS (ESI) *m/z* calcd for $C_{10}H_{11}O_2NBrS$ 287.96994, found 287.96986 [M-H$^+$]. $[\alpha]_D^{25}$ = +213.7 (*c* 0.14, EtOAc). HPLC conditions: ODH column, *n*-hexane:EtOH = 90:10, flow rate = 0.5 mL·min^{-1}, t_R = 15.0 min (minor), t_R = 17.1 min (major).

(*S*)-3-[(*tert*-Butyldimethylsilyl)oxy]-*N*-[(*S*)-(5-chlorothiophen-2-yl)sulfinyl]butanamide (1s)

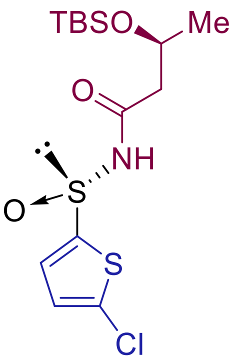

Obtained by separation of diasteroisomers on column chromatography, using general procedure **Step 2** (using 1 equiv of NaHMDS instead of LiHMDS) on the racemic sulfinamine. Oil, 12% yield. ^{1}H NMR (500 MHz, $CDCl_3$) δ 9.20 (s, 1H), 7.32 (d, *J* = 4.0 Hz, 1H), 6.96 (d, *J* = 4.0 Hz, 1H), 4.28 – 4.18 (m, 1H), 2.64 (dd, *J* = 15.2, 4.2 Hz, 1H), 2.43 (dd, *J* = 15.2, 4.0 Hz, *2*H), 1.29 (d, J = 6.4 Hz, 3H), 0.76 (s, 9H), 0.05 (s, 3H), 0.04 (s, 3H). ^{13}C NMR (126 MHz, $CDCl_3$) δ 172.1, 144.1, 136.9, 129.2, 127.3, 65.7, 45.5, 25.7, 22.4, 17.9, -4.4, -5.0. IR (film) ν (cm^{-1}) 3201, 1682, 1410, 1324, 1112, 1095, 811. HR-MS (ESI) *m/z* calcd for $C_{14}H_{24}O_3NClKS_2Si$ 420.02870, found 420.02889 [$M+K^+$]. $[\alpha]_D^{25}$ = +145.3 (*c* 0.14, EtOAc). HPLC conditions: ADH column, *n*-hexane:EtOH = 98:2, flow rate = 0.5 mL·min^{-1}, t_R = 21.9 min (major), t_R = 28.7 min (minor).

(*R*)-3-[(*tert*-Butyldimethylsilyl)oxy]-*N*-[(*S*)-(5-chlorothiophen-2-yl)sulfinyl]butanamide (1s’)

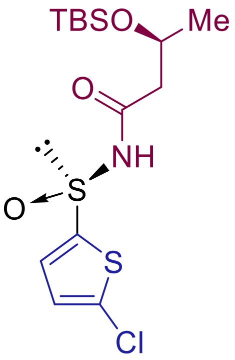

Obtained by separation of diasteroisomers on column chromatography, using general procedure **Step 2** (using 1 equiv of NaHMDS instead of LiHMDS) on the racemic sulfinamine. Oil, 17% yield. ^{1}H NMR (500 MHz, $CDCl_3$) δ 9.16 (s, 1H), 7.34 (d, *J* = 4.0 Hz, 1H), 6.97 (d, *J* = 4.0 Hz, 1H), 4.21 (dd, *J* = 10.5, 4.4 Hz, 1H), 2.66 (dd, *J* = 15.4, 4.1 Hz, 1H), 2.45 (dd, *J* = 15.3, 3.6 Hz, 1H), 1.25 (d, *J* = 6.3 Hz, 3H), 0.78 (s, 9H), 0.07 (s, 3H), 0.04 (s, 3H). ^{13}C NMR (126 MHz, $CDCl_3$) δ 171.6, 144.1, 136.9, 129.2, 127.2, 65.6, 45.6, 25.7, 22.7, 17.8, -4.7, -4.9. $[\alpha]_D^{25}$ = -144.3 (*c* 0.14 EtOAc). HPLC conditions: ADH column, *n*-hexane:EtOH = 98:2, flow rate = 0.5 mL·min^{-1}, t_R = 24.9 min (minor), t_R = 29.2 min (major).

(*S*)-3-[(*tert*-Butyldimethylsilyl)oxy]-*N*-[(*S*)-(thiophen-2-yl)sulfinyl]butanamide (1t)

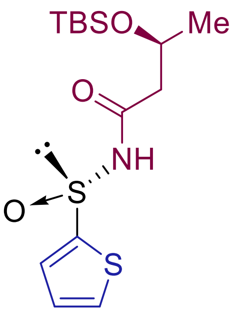

Obtained by separation of diasteroisomers on column chromatography, using general procedure **Step 2** (using 1 equiv of NaHMDS instead of LiHMDS) on the racemic sulfinamine. Oil, 16% yield. ^{1}H NMR (500 MHz, $CDCl_3$) δ 9.17 (s, 1H), 7.64 (dd, *J* = 5.0, 1.3 Hz, 1H), 7.55 (dd, *J* = 3.7, 1.3 Hz, 1H), 7.15 (dd, *J* = 5.0, 3.7 Hz, 1H), 4.20 (dd, *J* = 10.6, 4.5 Hz, 1H), 2.66 (dd, *J* = 15.4, 4.1 Hz, 1H), 2.49 – 2.42 (m, 1H), 1.24 (d, *J* = 6.3 Hz, 3H), 0.74 (s, 9H), 0.05 (s, 3H), 0.00 (s, *J* = 3.0 Hz, 3H). ^{13}C NMR (126 MHz, $CDCl_3$) δ 171.7, 146.1, 131.4, 129.6, 127.9, 65.6, 45.5, 25.8, 25.7, 22.7, 17.8, -4.7, -5.0. IR (film) ν (cm^{-1}) 3213, 2975, 1671, 1322, 1126, 811. HR-MS (ESI) *m/z* calcd for $C_{14}H_{26}O_3NClS_2Si$ 348.11179, found 348.11122 [M+H$^+$]. $[\alpha]_D^{25}$ = +183.5 (*c* 0.14 EtOAc). HPLC conditions: ODH column, *n*-hexane:EtOH = 90:10, flow rate = 1.0 mL·min^{-1}, t_R = 5.8 min (major), t_R = 6.8 min (minor).

(*R*)-3-[(*tert*-Butyldimethylsilyl)oxy]-*N*-[(*S*)-(thiophen-2-yl)sulfinyl]butanamide (1t')

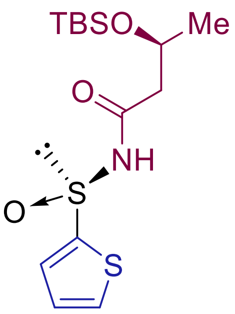

Obtained by separation of diasteroisomers on column chromatography, using general procedure **Step 2** (using 1 equiv of NaHMDS instead of LiHMDS) on the racemic sulfinamine. Oil, 19% yield. ^{1}H NMR (500 MHz, $CDCl_3$) δ 9.12 (s, 1H), 7.63 (d, *J* = 4.9 Hz, 1H), 7.54 (d, *J* = 3.7 Hz, 1H), 7.16 – 7.13 (m, 1H), 4.23 (dd, *J* = 10.5, 4.3 Hz, 1H), 2.65 (dd, *J* = 15.1, 4.2 Hz, 1H), 2.49 – 2.38 (m, 1H), 1.30 (d, *J* = 6.4 Hz, 3H), 0.73 (s, 9H), 0.03 (s, 3H), 0.01 (s, 3H). ^{13}C NMR (126 MHz, $CDCl_3$) δ 172.0, 146.1, 131.4, 129.5, 127.9, 65.6, 45.5, 25.7, 22.4, 17.9, -4.6, -5.1. $[\alpha]_D^{25}$ = -183.5 (*c* 0.14 EtOAc). HPLC conditions: ODH column, *n*-hexane:EtOH = 90:10, flow rate = 1.0 mL·min^{-1}, t_R = 6.1 min (minor), t_R = 6.9 min (major).

Tetrabutylammonium butyryl(*p*-tolylsulfinyl)amide (3)

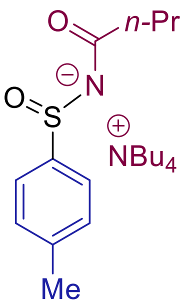

Yellow oil, 87% yield. ^{1}H NMR (400 MHz, Acetonitrile-d_3) δ 7.51 (d, *J* = 8.2 Hz, 2H), 7.22 (d, *J* = 7.6 Hz, 2H), 3.77 – 2.81 (m, 8H), 2.74 (s, 8H), 2.34 (s, 3H), 2.16 – 2.03 (m, 2H), 1.66 – 1.49 (m, 8H), 1.33 (h, *J* =

7.4 Hz, 8H), 0.95 (t, *J* = 7.3 Hz, 12H), 0.88 (t, *J* = 7.4 Hz, 3H). ^{13}C NMR (101 MHz, CD_3CN) δ = 180.4, 148.4, 148.4, 140.2, 129.8 (2C), 126.0 (2C), 59.3 (4C), 41.2, 24.3 (4C), 21.3, 20.5, 20.3 (4C), 14.5, 13.8 (4C). IR (film) ν (cm^{-1}) 2959, 2935, 2872, 1685, 1549, 1488, 1458, 1358, 1036, 796. HR-MS (ESI) *m/z* calcd for $C_{11}H_{14}NO_2S$ 224.07507, found 224.07509 [M-NBu_4]$^-$.

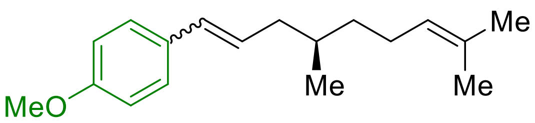

(*R*)-1-(4,8-Dimethylnona-1,7-dien-1-yl)-4-methoxybenzene

Oil, 88% yield. ^{1}H NMR (400 MHz, $CDCl_3$) δ 7.21 (d, *J* = 8.8 Hz, 2H), 7.16 (d, *J* = 8.7 Hz, 2H), 6.80 (d, *J* = 8.8 Hz, 2H), 6.76 (d, *J* = 8.8 Hz, 2H), 6.32 (d, *J* = 11.7 Hz, 1H), 6.25 (d, *J* = 15.7 Hz, 1H), 6.00 (dt, *J* = 15.6, 7.3 Hz, 1H), 5.53 (dt, *J* = 11.7, 7.2 Hz, 1H), 5.11 – 4.99 (m, 2H), 3.72 (s, 3H), 3.71 (s, 3H), 2.34 – 2.22 (m, 1H), 2.19 – 2.07 (m, 2H), 2.04 – 1.85 (m, 5H), 1.63 (d, *J* = 1.4 Hz, 3H), 1.62 (d, *J* = 1.8 Hz, 3H), 1.55 (d, *J* = 1.3 Hz, 3H), 1.53 (d, *J* = 1.3 Hz, 3H), 1.37 – 1.28 (m, 4H) 1.20 – 1.05 (m, 2H), 0.87 (d, *J* = 6.7 Hz, 3H), 0.86 (d, *J* = 6.7 Hz, 3H). $[\alpha]_D^{25}$ = -13.1 (*c* 0.14 $CHCl_3$).

Characterization data of products (2.1-2.40)

N-[(1*R*,2*R*)-1-(4-Methoxyphenyl)-1-(*p*-tolyl)propan-2-yl]butyramide (2.1)

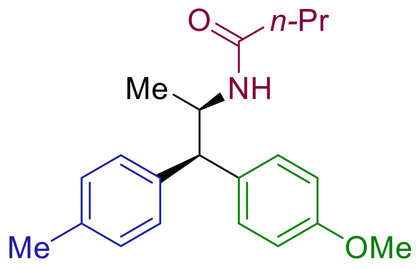

White solid, 83% yield, m.p. = 123-125 °C. ^{1}H NMR (400 MHz, $CDCl_3$) δ 7.17 (d, *J* = 8.7 Hz, 2H), 7.14 (d, *J* = 8.2 Hz, 2H), 7.07 (d, *J* = 8.0 Hz, 2H), 6.80 (d, *J* = 8.7 Hz, 2H), 5.16 (d, *J* = 8.5 Hz, 1H), 4.90 – 4.76 (m, 1H), 3.78 – 3.70 (m, 4H), 2.27 (s, 3H), 2.06 – 1.82 (m, 2H), 1.59 – 1.41 (m, 2H), 1.10 (d, *J* = 6.4 Hz, 3H), 0.77 (t, *J* = 7.4 Hz, 3H). ^{13}C NMR (101 MHz, $CDCl_3$) δ 172.3, 158.4, 139.3, 136.2, 134.9, 129.4 (2C), 129.2 (2C), 128.1 (2C), 114.2 (2C), 56.9, 55.4, 47.5, 39.1, 21.1, 20.6, 19.2, 13.7. IR (film) ν (cm^{-1}) 3270, 2928, 1638, 1511, 1249, 1031, 807. HR-MS (ESI) *m/z* calcd for $C_{21}H_{28}O_2N$ 326.21146, found 326.21102 $[M+H^+]$. $[\alpha]_D^{25}$ = +19.4 (*c* 0.14, EtOAc). HPLC conditions: ODH column, *n*-hexane:*i*-PrOH = 95:5, flow rate = 1.0 mL·min^{-1}, t_R = 10.3 min (major), t_R = 14.4 min (minor).

N-[(1*S*,2*S*)-1-(4-Methoxyphenyl)-1-(*p*-tolyl)propan-2-yl]butyramide (2.1')

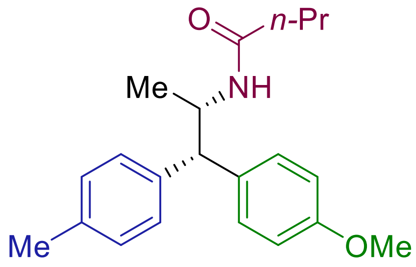

White solid, 83% yield, m.p. = 123-125 °C. $[\alpha]_D^{25}$ = -20.3 (*c* 0.14, EtOAc). HPLC conditions: ODH column, *n*-hexane:*i*-PrOH = 95:5, flow rate = 1.0 mL·min^{-1}, t_R = 10.2 min (minor), t_R = 14.1 min (major).

2-(4-Methoxyphenyl)-*N*-[(1*R*,2*R*)-1-(4-methoxyphenyl)-1-(*p*-tolyl)propan-2-yl]acetamide (2.2)

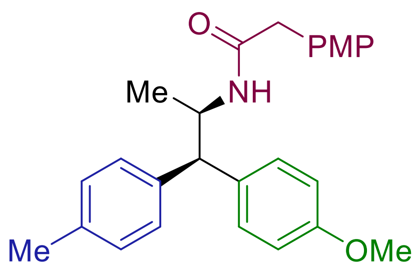

White solid, 81% yield, m.p. = 59- 61ºC. ^{1}H NMR (400 MHz, $CDCl_3$) δ 7.11 (d, *J* = 8.7 Hz, 2H), 7.03 (d, *J* = 8.0 Hz, 2H), 6.98 (d, *J* = 8.2 Hz, 2H), 6.81 – 6.74 (m, 4H), 6.72 (d, *J* = 8.8 Hz, 2H), 5.12 (d, *J* = 8.6 Hz, 1H), 4.73 – 4.63 (m, 1H), 3.80 (s, 3H), 3.75 (s, 3H), 3.61 (d, *J* = 9.4 Hz, 1H), 3.34 (s, 2H), 2.31 (s, 3H), 1.05 (d, *J* = 6.4 Hz, 3H).^{13}C NMR (101 MHz, $CDCl_3$) δ 170.7, 158.8, 158.3, 139.1, 136.1, 134.5, 130.7 (2C), 129.4 (2C), 129.1 (2C), 128.1 (2C), 126.7, 114.4 (2C), 114.1 (2C), 56.8, 55.4, 55.3, 47.9, 43.1, 21.1, 20.3. IR (film) ν (cm^{-1}) 3282, 2930, 1638, 1509, 1244, 1173, 1031, 806, 638. HR-MS (ESI) *m/z* calcd for $C_{26}H_{30}O_3N$ 404.22202, found 404.22154 [$M+H^+$]. $[\alpha]_D^{25}$ = +23.8 (*c* 0.14, EtOAc). HPLC conditions: ADH column, *n*-hexane:*i*-PrOH = 95:5, flow rate = 1.0 mL·min^{-1}, t_R = 21.4 min (major), t_R = 31.4 min (minor).

(*R*)-*N*-[(1*R*,2*R*)-1-(4-Methoxyphenyl)-1-(*p*-tolyl)propan-2-yl]-2-phenylbutanamide (2.3)

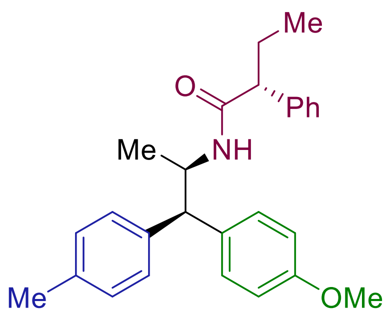

White solid, 74 % yield, m.p. = 101 – 103ºC. ^{1}H NMR (400 MHz, $CDCl_3$) δ 7.21 – 7.15 (m, 3H), 7.07 – 7.03 (m, 2H), 6.98 – 6.89 (m, 6H), 6.77 – 6.72 (m, 2H), 5.12 (d, *J* = 8.5 Hz, 1H), 4.86 – 4.68 (m, 1H), 3.74 (s, 3H), 3.63 (d, *J* = 9.2 Hz, 1H), 3.06 (dd, *J* = 8.4, 6.7 Hz, 1H), 2.27 (s, 3H), 2.20 – 2.03 (m, 1H), 1.65 – 1.55 (m, 1H), 1.06 (d, *J* = 6.5 Hz, 3H), 0.77 (t, *J* = 7.4 Hz, 3H).^{13}C NMR (101 MHz, $CDCl_3$) δ 172.6, 158.3, 139.7, 138.9, 136.0, 134.6, 129.3 (2C), 129.1 (2C), 128.7 (2C), 128.2 (2C), 128.1 (2C), 127.0, 114.0 (2C), 56.7, 55.3, 55.3, 47.6, 25.8, 21.1, 20.4, 12.4. IR (film) ν (cm^{-1}) 3303, 2929, 1639, 1509, 1253, 1176, 1034, 825, 805, 698, 547. HR-MS (ESI) *m/z* calcd for $C_{27}H_{32}O_2N$ 402.24276, found 402.24275 [$M+H^+$]. $[\alpha]_D^{25}$ = +26.1 (*c* 0.14, EtOAc). HPLC conditions: ADH column, *n*-hexane:*i*-PrOH = 95:5, flow rate = 1.0 mL·min^{-1}, t_R = 25.0 min (minor), t_R = 64.6 min (major).

_N_-[(1_R_,2_R_)-1-(4-Methoxyphenyl)-1-(_p_-tolyl)propan-2-yl]cyclohexanecarboxamide (2.4)

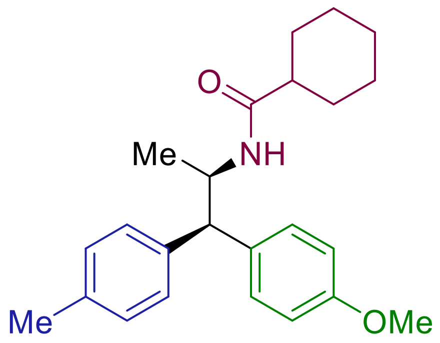

Oil, 77% yield. ^{1}H NMR (400 MHz, $CDCl_3$) δ 7.17 (d, J = 8.7 Hz, 2H), 7.13 (d, J = 8.1 Hz, 2H), 7.06 (d, J = 7.7 Hz, 2H), 6.80 (d, J = 8.7 Hz, 2H), 5.17 (d, J = 8.7 Hz, 1H), 4.86 – 4.73 (m, 1H), 3.79 – 3.72 (m, 1H), 3.77 (s, 3H), 2.27 (s, 3H), 1.92 – 1.80 (m, 1H), 1.74 – 1.44 (m, 5H), 1.33 – 1.11 (m, 5H), 1.09 (d, J = 6.5 Hz, 3H). ^{13}C NMR (101 MHz, $CDCl_3$) δ 175.3, 158.3, 139.3, 136.2, 134.9, 129.3 (2C), 129.2 (2C), 128.2 (2C), 114.1 (2C), 57.0, 55.4, 47.1, 45.7, 29.6, 29.4, 25.9, 25.8, 25.7, 21.1, 20.6. IR (film) ν (cm^{-1}) 3283, 1638, 1509, 1245, 1173, 1031, 806, 554. HR-MS (ESI) _m/z_ calcd for $C_{24}H_{32}O_2N$ 366.24276, found 366.24246 [$M+H^+$]. $[\alpha]_D^{25}$ = +25.7 (_c_ 0.14, EtOAc). HPLC conditions: ADH column, _n_-hexane:_i_-PrOH = 95:5, flow rate = 1.0 mL·min^{-1}, t_R = 23.1 min (minor), t_R = 33.8 min (major).

Ethyl 4-[((1_R_,2_R_)-1-(4-methoxyphenyl)-1-(_p_-tolyl)propan-2-yl)amino]-4-oxobutanoate (2.5)

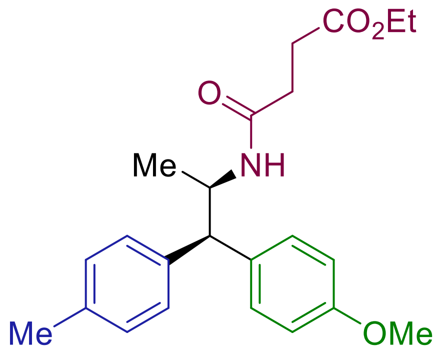

Oil, 80% yield. ^{1}H NMR (400 MHz, $CDCl_3$) δ 7.16 (d, J = 8.7 Hz, 2H), 7.13 (d, J = 8.1 Hz, 2H), 7.06 (d, J = 8.0 Hz, 2H), 6.80 (d, J = 8.7 Hz, 2H), 5.59 (br d, J = 8.8 Hz, 1H), 4.86 – 4.72 (m, 1H), 4.11 – 4.02 (m, 2H), 3.79 – 3.71 (m, 1H), 3.76 (s, 3H), 2.55 (dt, J = 17.1, 6.9 Hz, 1H), 2.41 – 2.32 (m, 1H), 2.29 – 2.24 (m, 2H), 2.27 (s, 3H), 1.23 (t, J = 7.1 Hz, 3H), 1.09 (d, J = 6.5 Hz, 3H). ^{13}C NMR (101 MHz, $CDCl_3$) δ 173.2, 170.9, 158.4, 139.3, 136.2, 134.8, 129.3 (2C), 129.2 (2C), 128.1 (2C), 114.2 (2C), 60.8, 56.9, 55.3, 47.7, 31.6, 29.8, 21.1, 20.4, 14.3. IR (film) ν (cm^{-1}) 3062, 2981, 1734, 1698, 1426, 1374, 1133, 1088, 807. HR-MS (ESI) _m/z_ calcd for $C_{23}H_{30}O_4N$ 384.21693, found 384.21701 [$M+H^+$]. $[\alpha]_D^{25}$ = +19.9 (_c_ 0.14, EtOAc). HPLC conditions: ADH column, _n_-hexane:_i_-PrOH = 95:5, flow rate = 1.0 mL·min^{-1}, t_R = 30.4 min (minor), t_R = 41.0 min (major).

(*S*)-3-[(*tert*-Butyldimethylsilyl)oxy)-*N*-((1*R*,2*R*)-1-(4-methoxyphenyl)-1-(*p*-tolyl)propan-2-yl]butanamide (2.6)

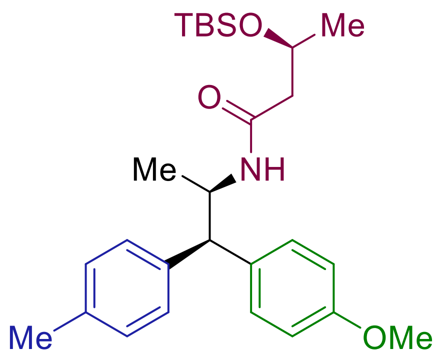

Oil, 76% yield. ^{1}H NMR (400 MHz, Acetone-d_6) δ 7.27 – 7.17 (m, 4H), 7.02 (d, *J* = 7.8 Hz, 2H), 6.88 –6.80 (m, 3H), 4.88 – 4.71 (m, 1H), 4.21 – 4.12 (m, 1H), 3.83 (d, *J* = 11.1 Hz, 1H), 3.73 (s, 3H), 2.22 (s, 3H), 2.12 (dd, *J* = 14.0, 6.4 Hz, 1H), 1.95 (dd, *J* = 14.0, 5.9 Hz, 1H), 1.03 (d, *J* = 6.5 Hz, 3H), 0.87 – 0.80 (m, 12H), 0.04 (s, 3H), 0.04 (s, 3H). ^{13}C NMR (101 MHz, Acetone-d_6) δ 169.7, 159.2, 141.4, 136.6, 136.0, 129.8 (2C), 129.5 (2C), 128.8 (2C), 114.7 (2C), 66.8, 58.1, 55.4, 47.8, 47.2, 26.2 (3C), 23.5, 20.9, 20.9, 18.5, -4.5, -4.7. IR (film) ν (cm^{-1}) 2954, 2857, 1704, 1410, 1253, 1001, 806. HR-MS (ESI) *m/z* calcd for $C_{27}H_{42}O_3NSi$ 456.29285, found 456.29277 [M+H$^+$]. $[\alpha]_D^{25}$ = +14.5 (*c* 0.14, EtOAc). HPLC conditions: ADH column, *n*-hexane:*i*-PrOH = 95:5, flow rate = 1.0 mL·min^{-1}, t_R = 12.8 min (minor), t_R = 17.6 min (major).

***N*-[(1*R*,2*R*)-1-(4-Methoxyphenyl)-1-(*p*-tolyl)propan-2-yl]benzamide (2.7)**

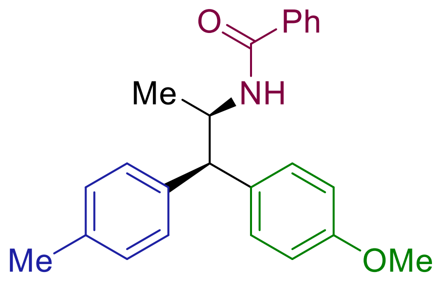

White solid, 87 % yield, m.p. = 78 – 80 ºC. ^{1}H NMR (400 MHz, $CDCl_3$) δ 7.52 – 7.47 (m, 2H), 7.46 – 7.38 (m, 1H), 7.37 – 7.31 (m, 2H), 7.24 – 7.16 (m, 4H), 7.09 (d, *J* = 8.0 Hz, 2H), 6.83 (d, *J* = 8.7 Hz, 2H), 5.87 (d, *J* = 8.5 Hz, 1H), 5.06 – 4.94 (m, 1H), 3.93 (d, *J* = 9.1 Hz, 1H), 3.77 (s, 3H), 2.27 (s, 3H), 1.23 (d, *J* = 6.5 Hz, 3H). ^{13}C NMR (101 MHz, $CDCl_3$) δ 167.0, 158.4, 139.1, 136.4, 135.2, 134.7, 131.3, 129.5 (2C), 129.3 (2C), 128.6 (2C), 128.2 (2C), 126.8 (2C), 114.2 (2C), 56.7, 55.4, 48.1, 21.1, 20.5. IR (film) ν (cm^{-1}) 3305, 2929, 1639, 1509, 1252, 1035, 806. HR-MS (ESI) *m/z* calcd for $C_{24}H_{26}O_2N$ 360.19581, found 360.19541 [M+H$^+$]. $[\alpha]_D^{25}$ = +43.5 (*c* 0.14, EtOAc). HPLC conditions: ADH column, *n*-hexane:*i*-PrOH = 95:5, flow rate = 1.0 mL·min^{-1}, t_R = 12.9 min (major), t_R = 25.3 min (minor).

_N_-[(1_R_,2_R_)-1-(4-Methoxyphenyl)-1-(_p_-tolyl)propan-2-yl]furan-2-carboxamide (2.8)

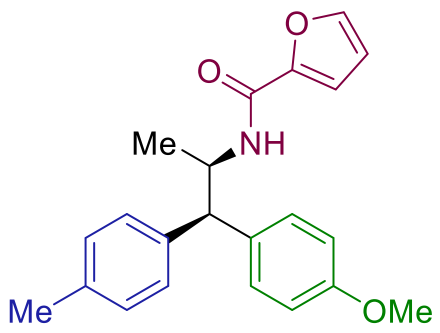

White solid, 68% yield, m.p. = 61 – 63ºC. ^{1}H NMR (400 MHz, Acetone-d$_6$) δ 7.52 (dd, *J* = 1.7, 0.8 Hz, 1H), 7.32 (br d, *J* = 8.9 Hz, 1H), 7.30 – 7.12 (m, 4H), 7.00 (d, *J* = 7.9 Hz, 2H), 6.92 (dd, *J* = 3.4, 0.7 Hz, 1H), 6.90 – 6.79 (m, 2H), 6.48 (dd, *J* = 3.4, 1.8 Hz, 1H), 5.10 – 4.88 (m, 1H), 4.11 (d, *J* = 11.1 Hz, 1H), 3.74 (s, 3H), 2.18 (s, 3H), 1.15 (d, *J* = 6.5 Hz, 3H).^{13}C NMR (101 MHz, Acetone-*d*$_6$) δ 159.3, 157.9, 149.7, 144.9, 141.4, 136.7, 136.2, 130.0 (2C), 129.7 (2C), 128.8 (2C), 114.9 (2C), 113.9, 112.6, 57.4, 55.5, 48.1, 20.9, 20.8. IR (film) ν (cm^{-1}) 2969, 2929, 1726, 1510, 1375, 1198, 802. HR-MS (ESI) *m/z* calcd for $C_{22}H_{24}O_3N$ 350.17507, found 350.17491 [M+H^{+}]. $[\alpha]_D^{25}$ = +31.2 (*c* 0.14, EtOAc). HPLC conditions: ODH column, *n*-hexane:*i*-PrOH = 95:5, flow rate = 1.0 mL·min^{-1}, t_R = 11.3 min (major), t_R = 20.5 min (minor).

***tert*-Butyl [(1_R_,2_R_)-1-(4-methoxyphenyl)-1-(_p_-tolyl)propan-2-yl]carbamate (2.9)**

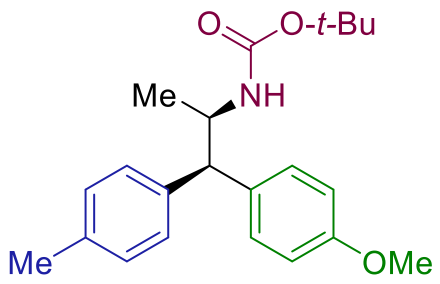

White solid, 76 % yield, m.p. 59 – 61 ºC. ^{1}H NMR (400 MHz, CDCl$_3$) δ 7.19 – 7.12 (m, 4H), 7.07 (d, *J* = 8.0 Hz, 2H), 6.81 (d, *J* = 8.7 Hz, 2H), 4.43 (br s, 1H), 4.29 (br s, 1H), 3.76 (m, 4H), 2.28 (s, 3H), 1.36 (s, 9H), 1.10 (d, *J* = 6.4 Hz, 3H).^{13}C NMR (101 MHz, CDCl$_3$) δ 158.3, 155.4, 139.4, 135.9, 135.1, 129.3 (2C), 129.2 (2C), 128.3 (2C), 114.1 (2C), 79.2, 57.2, 55.3, 49.1, 28.5 (3C), 21.1, 20.8. IR (film) ν (cm^{-1}) 2969, 1737, 1718, 1509 ,1365, 1176, 1032. HR-MS (ESI) *m/z* calcd for $C_{22}H_{29}O_3NNa$ 378.20396, found 378.20370 [M+Na^{+}]. $[\alpha]_D^{25}$ = +36.1 (*c* 0.14, EtOAc). HPLC conditions: ADH column, *n*-hexane:*i*-PrOH = 95:5, flow rate = 1.0 mL·min^{-1}, t_R = 9.3 min (major), t_R = 11.0 min (minor).

_N_-[(1_R_,2_R_)-1-(4-Methoxyphenyl)-1-phenylpropan-2-yl]butyramide (2.10)

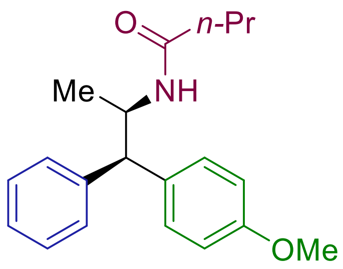

Oil, 80% yield. ^{1}H NMR (400 MHz, $CDCl_3$) δ 7.28 – 7.23 (m, 4H), 7.20 – 7.13 (m, 3H), 6.80 (d, *J* = 8.7 Hz, 2H), 5.23 (d, *J* = 8.8 Hz, 1H), 4.90 – 4.79 (m, 1H), 3.78 – 3.72 (m, 1H), 3.75 (s, 3H), 1.94 (dt, *J* = 14.3, 7.5 Hz, 1H), 1.85 (dt, *J* = 14.3, 7.4 Hz, 1H), 1.48 – 1.37 (m, 2H), 1.09 (d, *J* = 6.4 Hz, 3H), 0.72 (t, *J* = 7.4 Hz, 3H). ^{13}C NMR (101 MHz, $CDCl_3$) δ 172.5, 158.2, 142.2, 134.5, 129.1 (2C), 128.7 (2C), 128.1 (2C), 126.7, 114.0 (2C), 57.4, 55.3, 47.4, 39.0, 20.6, 19.2, 13.6. IR (film) ν (cm^{-1}) 2968, 2360, 1737, 1509, 1373, 1067, 804. HR-MS (ESI) *m/z* calcd for $C_{20}H_{26}O_2N$ 312.19581, found 312.19556 [$M+H^+$]. $[\alpha]_D^{25}$ = +19.5 (*c* 0.14, EtOAc). HPLC conditions: ADH column, *n*-hexane:*i*-PrOH = 95:5, flow rate = 1.0 mL·min^{-1}, t_R = 19.7 min (minor), t_R = 33.0 min (major).

_N_-[(1_S_,2_R_)-1-(4-Bromophenyl)-1-(4-methoxyphenyl)propan-2-yl]butyramide (2.11)

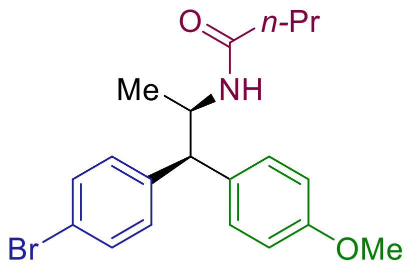

White solid, 75% yield, m.p. = 109 – 111 °C. ^{1}H NMR (400 MHz, $CDCl_3$) δ 7.38 (d, *J* = 8.5 Hz, 2H), 7.18 – 7.11 (m, 4H), 6.82 (d, *J* = 8.7 Hz, 2H), 5.13 (d, *J* = 8.7 Hz, 1H), 4.95 – 3.78 (m, 1H), 3.78 – 3.71 (m, 4H), 2.05 – 1.88 (m, 2H), 1.55 – 1.41 (m, 2H), 1.10 (d, *J* = 6.5 Hz, 3H), 0.78 (t, *J* = 7.4 Hz, 3H). ^{13}C NMR (101 MHz, $CDCl_3$) δ 172.3, 158.6, 141.5, 133.9, 131.7 (2C), 129.9 (2C), 129.2 (2C), 120.5, 114.3 (2C), 57.1, 55.4, 47.3, 39.0, 20.5, 19.2, 13.7. IR (film) ν (cm^{-1}) 3297, 2364, 1738, 1508, 1248, 999, 809. HR-MS (ESI) *m/z* calcd for $C_{20}H_{25}O_2NBr$ 390.10623, found 390.10607 [$M+H^+$]. $[\alpha]_D^{25}$ = +20.3 (*c* 0.14, EtOAc). HPLC conditions: ADH column, *n*-hexane:*i*-PrOH = 95:5, flow rate = 1.0 mL·min^{-1}, t_R = 19.7 min (minor), t_R = 29.2 min (major).

N-[(1S,2R)-1-(4-Fluorophenyl)-1-(4-methoxyphenyl)propan-2-yl]butyramide (2.12)

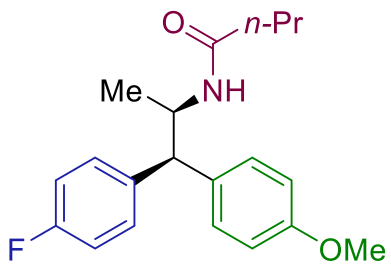

White solid, 61% yield, m.p. = 127 – 129 °C. ^{1}H NMR (400 MHz, Acetone-d_6) δ 7.39 – 7.32 (m, 2H), 7.25 (d, J = 8.7 Hz, 2H), 7.00 - 6.91 (m, 2H), 6.89 – 6.80 (m, 3H), 4.80 (ddq, J = 11.2, 9.2, 6.5 Hz, 1H), 3.92 (d, J = 11.2 Hz, 1H), 3.74 (s, 3H), 1.98 – 1.79 (m, 2H), 1.58 – 1.26 (m, 2H), 1.03 (d, J = 6.5 Hz, 3H), 0.69 (t, J = 7.4 Hz, 3H). ^{13}C NMR (101 MHz, Acetone-d_6) δ 171.9, 162.2 (d, J = 242.0 Hz), 159.4, 140.8 (d, J = 3.1 Hz), 136.4, 130.7 (d, J = 3.1 Hz, 2C), 129.9 (2C), 115.5 (d, J = 21.1 Hz, 2C), 114.9 (2C), 57.7, 55.5, 48.0, 38.9, 20.9, 19.8, 13.9. ^{19}F NMR (377 MHz, Acetone-d_6) δ -119.12. IR (film) ν (cm^{-1}) 3270, 2969, 1740, 1505, 1373, 1217, 1030, 820. HR-MS (ESI) *m/z* calcd for $C_{20}H_{25}O_2NF$ 330.18638, found 330.18580 [M+H$^+$]. $[\alpha]_D^{25}$ = +17.3 (*c* 0.14, EtOAc). HPLC conditions: ADH column, *n*-hexane:*i*-PrOH = 95:5, flow rate = 1.0 mL·min^{-1}, t_R = 23.7 min (minor), t_R = 44.8 min (major).

(S)-3-[(*tert*-Butyldimethylsilyl)oxy)-N-((1R,2R)-1-(4-methoxyphenyl)-1-(4-(trifluoromethyl)phenyl)propan-2-yl]butanamide (2.13)

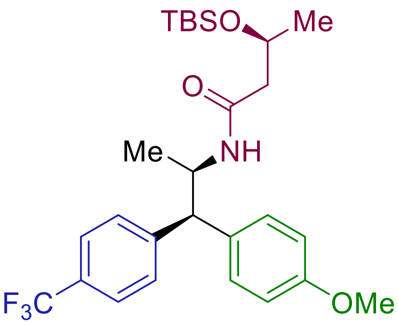

Oil, 58% yield. ^{1}H NMR (400 MHz, Acetone-d_6) δ 7.57 (s, 4H), 7.29 (d, J = 8.7 Hz, 2H), 6.96 (d, J = 8.9 Hz, 1H), 6.87 (br d, J = 8.8 Hz, 2H), 4.99 – 4.48 (m, 1H), 4.05 (d, J = 11.2 Hz, 1H), 4.02 – 3.96 (m, 1H), 3.74 (s, 3H), 2.14 (dd, J = 13.8, 5.0 Hz, 1H), 2.03 – 1.97 (m, 1H), 1.07 (d, J = 6.5 Hz, 3H), 1.02 (d, J = 6.1 Hz, 3H), 0.83 (s, 9H), 0.01 (s, 3H), -0.02 (s, 3H). ^{13}C NMR (101 MHz, Acetone-d_6) δ 169.8, 159.5, 149.2, 135.3, 129.9 (2C), 129.7 (2C), 127.68 (q, J = 32.1 Hz), 125.83 (q, J = 3.8 Hz, 2C), 124.6 (q, J = 270.8 Hz) 114.9 (2C), 66.9, 58.1, 55.5, 47.9, 47.2, 26.2 (3C), 23.7, 20.5, 18.5, -4.6, -4.7. ^{19}F NMR (377 MHz, Acetone-d_6) δ -62.81. IR (film) ν (cm^{-1}) 3301, 2930, 1643, 1511, 1324, 1067, 812. HR-MS (ESI) *m/z* calcd for $C_{27}H_{39}O_3NF_3Si$ 510.26458, found 510.26480 [M+H$^+$]. $[\alpha]_D^{25}$ = +13.5 (*c* 0.14, EtOAc). HPLC conditions: ADH column, *n*-hexane:*i*-PrOH = 97:3, flow rate = 1.0 mL·min^{-1}, t_R = 21.6 min (minor), t_R = 32.7 min (major).

N-[(1R,2R)-1-Cyclohexyl-2-(4-methoxyphenyl)-2-(3,4,5-trimethoxyphenyl)ethyl] butyramide (2.14)

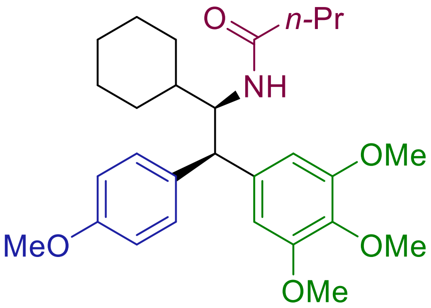

Oil, 57% yield. ^{1}H NMR (400 MHz, $CDCl_3$) δ 7.18 (d, J = 8.7 Hz, 2H), 6.80 (d, J = 8.7 Hz, 2H), 6.49 (s, 2H), 5.01 (d, J = 10.3 Hz, 1H), 4.69 (td, J = 10.0, 3.9 Hz, 1H), 3.94 (d, J = 9.8 Hz, 1H), 3.81 (s, 6H), 3.79 (s, 3H), 3.75 (s, 3H), 2.00 (ddd, J = 14.8, 8.0, 7.0 Hz, 1H), 1.90 (dt, J = 14.4, 7.4 Hz, 1H), 1.80 – 1.68 (m, 4H), 1.63 – 1.58 (m, 1H) 1.48 – 1.38 (m, 2H), 1.38 – 1.32 (m, 1H) 1.17 – 1.02 (m, 4H), 1.00 – 0.92 (m, 1H) 0.77 (t, J = 7.4 Hz, 3H). ^{13}C NMR (101 MHz, $CDCl_3$) δ 172.8, 158.4, 153.4 (2C), 138.9, 136.6, 134.4, 129.4 (2C), 114.0 (2C), 104.9 (2C), 60.9, 56.3 (2C), 55.4, 55.1, 53.0, 39.6, 39.1, 31.5, 26.6, 26.5, 26.3, 26.2, 19.3, 13.8. IR (film) ν (cm^{-1}) 2996, 2360, 1737, 1457, 1231, 1125, 831. HR-MS (ESI) *m/z* calcd for $C_{28}H_{40}O_5N$ 470.29010, found 470.29018 [$M+H^+$]. $[\alpha]_D^{25}$ = +28.8 (*c* 0.14, EtOAc). HPLC conditions: ADH column, *n*-hexane:*i*-PrOH = 95:5, flow rate = 1.0 mL·min^{-1}, t_R = 32.4 min (minor), t_R = 37.1 min (major).

(S)-3-[(tert-Butyldimethylsilyl)oxy)-N-((1S,2R)-1-(3-methoxyphenyl)-1-(4-methoxyphenyl)propan-2-yl]butanamide (2.15)

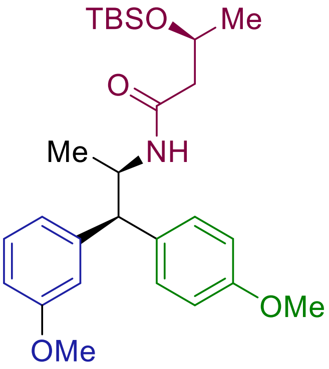

Oil, 69% yield. ^{1}H NMR (400 MHz, Acetone-d_6) δ 7.27 (d, J = 8.7 Hz, 2H), 7.11 (t, J = 7.9 Hz, 1H), 6.98 – 6.94 (m, 1H), 6.89 (d, J = 7.8 Hz, 2H), 6.84 (d, J = 8.6 Hz, 2H), 6.67 (dd, J = 8.2, 2.3 Hz, 1H), 4.92 – 4.77 (m, 1H), 4.17 (h, J = 6.1 Hz, 1H), 3.84 (d, J = 11.2 Hz, 1H), 3.74 (s, 3H), 3.74 (s, 3H), 2.14 (dd, J = 14.0, 6.3 Hz, 1H), 1.96 (dd, J = 14.0, 6.0 Hz, 1H), 1.03 (d, J = 6.5 Hz, 3H), 0.86 (s, 9H), 0.84 (d, J = 6.0 Hz, 3H), 0.05 (s, 3H), 0.04 (s, 3H). ^{13}C NMR (101 MHz, Acetone-d_6) δ 169.9, 160.7, 159.4, 146.1, 136.4, 129.9 (2C), 129.9, 121.3, 114.9 (2C), 114.6, 112.4, 67.00, 58.7, 55.5, 55.4, 47.8, 47.4, 26.3 (3C), 23.7, 21.0, 18.6, -4.4, -4.6. IR (film) ν (cm^{-1}) 3062, 1735, 1511, 1229, 1001, 810.HR-MS (ESI) *m/z* calcd for $C_{27}H_{42}O_4NSi$ 472.28776, found 472.28788 [$M+H^+$]. $[\alpha]_D^{25}$ = +19.9 (*c* 0.14, EtOAc). HPLC conditions: ADH column, *n*-hexane:*i*-PrOH = 95:5, flow rate = 1.0 mL·min^{-1}, t_R = 12.0 min (minor), t_R = 15.2 min (major).

(*S*)-3-[(*tert*-Butyldimethylsilyl)oxy)-*N*-((1*S*,2*R*)-1-(3-bromophenyl)-1-(4-methoxyphenyl)propan-2-yl]butanamide (2.16)

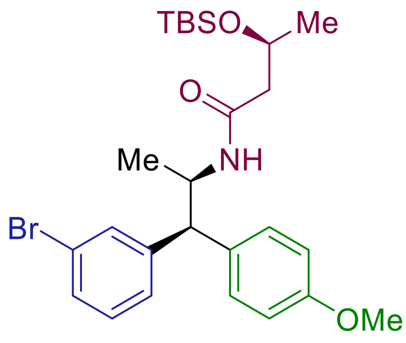

White solid, 59% yield, m.p. = 101 – 103 °C. ^{1}H NMR (400 MHz, Acetone-d_6) δ 7.49 (t, *J* = 1.8 Hz, 1H), 7.36 (d, *J* = 7.7 Hz, 1H), 7.32 – 7.26 (m, 3H), 7.19 (t, *J* = 7.8 Hz, 1H), 6.95 (br d, *J* = 8.9 Hz, 1H), 6.90 – 6.84 (m, 2H), 4.82 – 4.72 (m, 1H), 4.06 – 3.96 (m, 1H), 3.94 (d, *J* = 11.1 Hz, 1H), 3.75 (s, 3H), 2.22 – 2.13 (m, 1H), 2.03 – 1.98 (m, 1H) 1.08 – 1.02 (m, 6H), 0.85 (s, 9H), 0.03 (s, 3H), 0.01 (s, 3H). ^{13}C NMR (101 MHz, Acetone-d_6) δ 169.8, 159.5, 147.3, 135.5, 132.1, 130.9, 129.9 (2C), 129.9, 127.6, 122.6, 114.9 (2C), 67.0, 57.9, 55.4, 47.9, 47.2, 26.2 (3C), 23.7, 20.5, 18.5, -4.5, -4.6. IR (film) ν (cm^{-1}) 3296, 1738, 1508, 1377, 999, 809. HR-MS (ESI) *m/z* calcd for $C_{26}H_{39}O_3NBrSi$ 520.18771, found 520.18820 [M+H$^+$]. $[\alpha]_D^{25}$ = +20.5 (*c* 0.14, EtOAc). HPLC conditions: ADH column, *n*-hexane:*i*-PrOH = 95:5, flow rate = 1.0 mL·min^{-1}, t_R = 9.2 min (major), t_R = 13.5 min (minor).

***N*-[(1*S*,2*R*)-1-(4-Methoxyphenyl)-1-(*o*-tolyl)propan-2-yl]butyramide (2.17)**

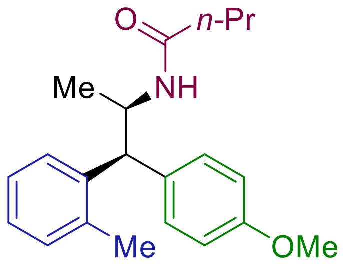

White solid, 67% yield, m.p. = 139-140 °C ^{1}H NMR (400 MHz, $CDCl_3$) δ 7.43 (d, *J* = 7.7 Hz, 1H), 7.22 – 7.16 (m, 1H), 7.15 – 7.10 (m, 2H), 7.09 – 7.04 (m, 2H), 6.78 (d, *J* = 8.7 Hz, 2H), 5.16 (br d, *J* = 8.1 Hz, 1H), 4.81 – 4.69 (m, 1H), 3.92 (d, *J* = 10.6 Hz, 1H), 3.75 (s, 3H), 2.25 (s, 3H), 2.05 – 1.98 (m, 2H), 1.52 – 1.43 (m, 2H), 1.09 (d, *J* = 6.3 Hz, 3H), 0.77 (t, *J* = 7.4 Hz, 3H). ^{13}C NMR (101 MHz, $CDCl_3$) δ 172.5, 158.3, 140.0, 136.4, 133.4, 130.8, 129.9 (2C), 126.8, 126.6, 126.4, 114.0 (2C), 55.3, 52.9, 48.1, 39.0, 20.7, 20.1, 19.3, 13.7. IR (film) ν (cm^{-1}) 3066, 2362, 1739, 1633, 1509, 1248, 1035, 827. HR-MS (ESI) *m/z* calcd for $C_{21}H_{28}O_2N$ 326.21146, found 326.21101 [M+H$^+$]. $[\alpha]_D^{25}$ = +18.3 (*c* 0.14, EtOAc). HPLC conditions: ADH column, *n*-hexane:*i*-PrOH = 95:5, flow rate = 1.0 mL·min^{-1}, t_R = 28.5 min (major), t_R = 37.4 min (minor).

N-[(1S,2R)-1-(2-Bromophenyl)-1-(4-methoxyphenyl)propan-2-yl]butyramide (2.18)

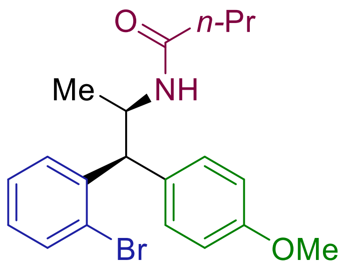

White solid, 68% yield, m.p. = 111-113 °C. ^{1}H NMR (400 MHz, $CDCl_3$) δ 7.50 – 7.46 (m, 2H), 7.27 – 7.25 (m, 3H), 7.03 – 6.98 (m, 1H), 6.82 (d, J = 8.8 Hz, 2H), 5.32 (d, J = 9.4 Hz, 1H), 4.91 – 4.81 (m, 1H), 4.32 (d, J = 11.4 Hz, 1H), 3.76 (s, 3H), 1.98 (dt, J = 14.8, 7.4 Hz, 1H), 1.88 (dt, J = 14.3, 7.4 Hz, 1H), 1.43 – 1.36 (m, 2H), 1.13 (d, J = 6.4 Hz, 3H), 0.70 (t, J = 7.4 Hz, 3H). ^{13}C NMR (101 MHz, $CDCl_3$) δ 172.4, 158.4, 141.5, 132.9, 132.9, 129.7 (2C), 129.1, 128.2, 128.0, 125.4, 114.2 (2C), 55.6, 55.3, 48.3, 38.9, 20.9, 19.2, 13.6. IR (film) ν (cm^{-1}) 3281, 2926, 1738, 1639, 1511, 1370, 1106, 832. HR-MS (ESI) *m/z* calcd for $C_{20}H_{25}O_2NBr$ 390.10632, found 390.10623 [$M+H^+$]. $[\alpha]_D^{25}$ = +21.1 (*c* 0.14, EtOAc). HPLC conditions: ADH column, *n*-hexane:*i*-PrOH = 95:5, flow rate = 1.0 mL·min^{-1}, t_R = 19.3 min (minor), t_R = 29.7 min (major).

(S)-3-[(tert-Butyldimethylsilyl)oxy]-N-[(1R,2R)-1-(5-chlorothiophen-2-yl)-1-(4-methoxyphenyl)propan-2-yl]butanamide (2.19)

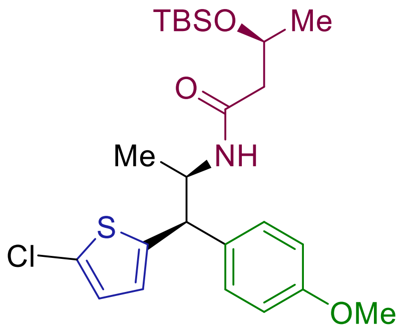

Oil, 71% yield. ^{1}H NMR (500 MHz, $CDCl_3$) 7.18 (d, J = 8.7 Hz, 2H), 6.84 (d, J = 8.7 Hz, 2H), 6.72 (dd, J = 3.8, 0.7 Hz, 1H), 6.69 (d, J = 3.8 Hz, 1H), 6.39 (d, J = 9.0 Hz, 1H), 4.77 – 4.64 (m, 1H), 4.26 – 4.10 (m, 1H), 3.94 (d, J = 9.2 Hz, 1H), 3.78 (s, 3H), 2.37 (dd, J = 15.0, 4.5 Hz, 1H), 2.19 (dd, J = 15.0, 5.3 Hz, 1H), 1.05 (d, J = 6.6 Hz, 3H), 0.99 (d, J = 6.3 Hz, 3H), 0.88 (s, 9H), 0.06 (s, 3H), 0.06 (s, 3H). ^{13}C NMR (101 MHz, $CDCl_3$) δ 170.5, 158.9, 145.1, 133.1, 129.4 (2C), 128.2, 125.8, 124.0, 114.2 (2C), 66.1, 55.4, 52.9, 48.6, 46.0, 25.9, 22.6, 20.1, 18.1, -4.5, -4.9. IR (film) ν (cm^{-1}) 3066, 1679, 1666, 1537, 1284, 1161, 1020, 826. HR-MS (ESI) *m/z* calcd for $C_{24}H_{37}O_3NClSSi$ 482.19465, found 482.19470 [$M+H^+$]. $[\alpha]_D^{25}$ = +120.1 (*c* 0.14, EtOAc). HPLC conditions: ODH column, *n*-hexane:*i*-PrOH = 95:5, flow rate = 1.0 mL·min^{-1}, t_R = 12.6 min (minor), t_R = 15.6 min (major).

(*S*)-3-[(*tert*-Butyldimethylsilyl)oxy]-*N*-[(1*R*,2*R*)-1-(thiophen-2-yl)-1-(4-methoxyphenyl)propan-2-yl]butanamide (2.20)

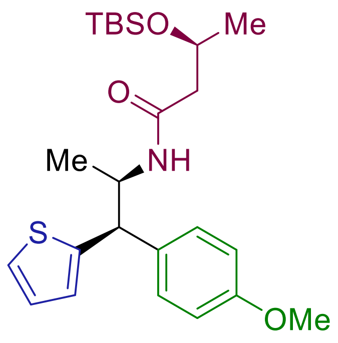

Oil, 58% yield. ^{1}H NMR (400 MHz, $CDCl_3$) δ 7.22 (d, *J* = 8.7 Hz, 2H), 6.95 (d, *J* = 3.4 Hz, 1H), 6.91 – 6.87 (m, 1H), 6.83 (d, *J* = 8.7 Hz, 2H), 6.37 (d, *J* = 8.8 Hz, 1H), 4.80 – 4.72 (m, 1H), 4.17 – 4.09 (m, 1H), 4.06 (d, *J* = 9.2 Hz, 1H), 3.77 (s, 3H), 2.34 (dd, *J* = 15.0, 4.5 Hz, 1H), 2.16 (dd, *J* = 15.0, 5.3 Hz, 1H), 1.07 (d, *J* = 6.6 Hz, 3H), 0.91 (d, *J* = 6.3 Hz, 3H), 0.88 (s, 9H), 0.05 (s, 3H), 0.05 (s, 3H). ^{13}C NMR (101 MHz, $CDCl_3$) δ 170.4, 158.7, 146.1, 133.8, 129.4, 126.8, 124.7, 123.9, 114.1, 66.1, 55.4, 52.6, 49.1, 46.0, 25.9, 22.5, 20.2, 18.1, -4.5, -4.9. IR (film) ν (cm^{-1}) 3015, 1685, 1670, 1535, 1288, 1176, 1024, 825. HR-MS (ESI) *m/z* calcd for $C_{24}H_{38}ONSSi$ 448.23362, found 448.23343 [M+H^{+}]. $[\alpha]_D^{25}$ = +98.1 (*c* 0.14, EtOAc). HPLC conditions: ADH column, *n*-hexane:*i*-PrOH = 95:5, flow rate = 1.0 mL·min^{-1}, t_R = 20.1 min (minor), t_R = 26.1 min (major).

***N*-[(1*R*,2*R*)-1-(4-Methoxyphenyl)-1,4-di-*p*-tolylbutan-2-yl]butyramide (2.21)**

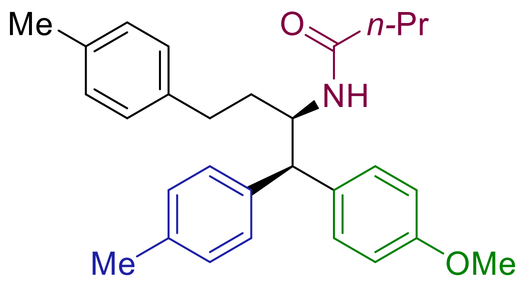

White solid, 65% yield, m.p. 187-189 °C. ^{1}H NMR (400 MHz, $CDCl_3$) δ 7.15 (d, *J* = 8.7 Hz, 2H), 7.11 (d, *J* = 8.1 Hz, 2H), 7.05 (d, *J* = 7.9 Hz, 4H), 6.99 (d, *J* = 8.0 Hz, 2H), 6.78 (d, *J* = 8.7 Hz, 2H), 5.08 (d, *J* = 9.6 Hz, 1H), 4.85 (qd, *J* = 9.3, 3.3 Hz, 1H), 3.89 (d, *J* = 9.3 Hz, 1H), 3.75 (s, 3H), 2.61 (tt, *J* = 14.0, 6.3 Hz, 2H), 2.30 (s, 3H), 2.27 (s, 3H), 2.02 – 1.88 (m, 3H), 1.60 – 1.50 (m, 1H), 1.50 – 1.42 (m, 2H), 0.78 (t, *J* = 7.4 Hz, 3H). ^{13}C NMR (101 MHz, $CDCl_3$) δ 172.7, 158.3, 139.4, 138.9, 136.2, 135.4, 134.7, 129.3 (2C), 129.2 (2C), 129.1 (2C), 128.4 (2C), 128.2 (2C), 114.2 (2C), 55.2, 55.3, 51.3, 39.1, 36.2, 31.9, 21.1, 21.0, 19.2, 13.8. IR (film) ν (cm^{-1}) 3295, 2924, 2362, 1633, 1509, 1247, 1033, 806. HR-MS (ESI) *m/z* calcd for $C_{29}H_{36}O_2N$ 430.27406, found 430.27465 [M+H^{+}]. $[\alpha]_D^{25}$ = +27.3 (*c* 0.14, EtOAc). HPLC conditions: ADH column, *n*-hexane:*i*-PrOH = 95:5, flow rate = 1.0 mL·min^{-1}, t_R = 37.4 min (minor), t_R = 54.8 min (major).

N-[(1R,2R)-1-Cyclohexyl-2-(4-methoxyphenyl)-2-(p-tolyl)ethyl]butyramide (2.22)

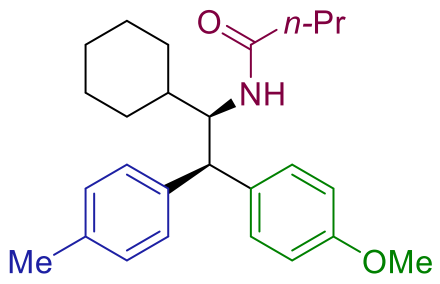

Oil, 59% yield. ^{1}H NMR (400 MHz, $CDCl_3$) δ 7.21 (d, J = 8.7 Hz, 2H), 7.13 (d, J = 8.1 Hz, 2H), 7.03 (d, J = 7.9 Hz, 2H), 6.80 (d, J = 8.7 Hz, 2H), 5.00 (br d, J = 10.5 Hz, 1H), 4.75 (td, J = 10.4, 3.3 Hz, 1H), 3.94 (d, J = 10.5 Hz, 1H), 3.76 (s, 3H), 2.25 (s, 3H), 2.04 – 1.93 (m, 1H), 1.91 – 1.83 (m, 1H), 1.78 – 1.61 (m, 5H), 1.48 – 1.37 (m, 2H), 1.12 – 1.01 (m, 4H), 0.91 – 0.81 (m, 2H), 0.74 (t, J = 7.4 Hz, 3H). ^{13}C NMR (101 MHz, $CDCl_3$) δ 172.8, 158.2, 139.9, 136.0, 135.3, 129.3 (2C), 128.9 (2C), 128.1 (2C), 114.3 (2C), 55.3, 55.2, 52.9, 39.4, 39.1, 31.5, 26.6, 26.3, 26.2, 26.1, 21.1, 19.2, 13.8. IR (film) ν (cm^{-1}) 3295, 2924, 2369, 1633, 1509, 1247, 1033, 806. HR-MS (ESI) *m/z* calcd for $C_{26}H_{36}O_2N$ 394.27406, found 394.27409 $[M+H^+]$. $[\alpha]_D^{25}$ = +29.3 (*c* 0.14, EtOAc). HPLC conditions: ADH column, *n*-hexane:*i*-PrOH = 95:5, flow rate = 1.0 mL·min^{-1}, t_R = 5.7 min (major), t_R = 8.8 min (minor).

N-[(1R,2R)-1-Cyclohexyl-2-(p-tolyl)-2-(3,4,5-trimethoxyphenyl)ethyl]butyramide (2.23)

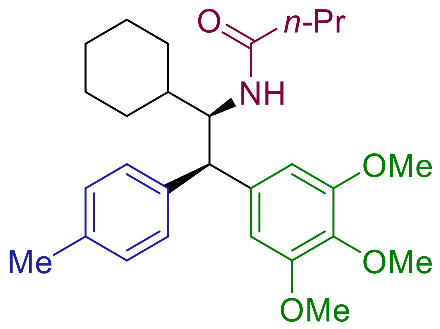

Oil, 47% yield. ^{1}H NMR (400 MHz, $CDCl_3$) δ 7.15 (d, J = 8.1 Hz, 2H), 7.06 (d, J = 7.9 Hz, 2H), 6.51 (s, 2H), 5.01 (br d, J = 10.3 Hz, 1H), 4.72 (td, J = 10.1, 3.9 Hz, 1H), 3.95 (d, J = 9.8 Hz, 1H), 3.81 (s, 6H), 3.79 (s, 3H), 2.27 (s, 3H), 2.05 – 1.96 (m, 1H), 1.92 – 1.84 (m, 1H), 1.79 – 1.68 (m, 7H), 1.46 – 1.36 (m, 2H), 1.16 – 1.05 (m, 3H), 1.00 – 0.92 (m, 1H), 0.75 (t, J = 7.4 Hz, 3H).^{13}C NMR (101 MHz, $CDCl_3$) δ 172.8, 153.4 (2C), 139.2, 138.8, 136.6, 136.4, 129.4 (2C), 128.3 (2C), 105.0 (2C), 60.9, 56.3 (2C), 55.0, 53.5, 39.6, 39.1, 31.5, 26.5, 26.5, 26.3, 26.2, 21.1, 19.2, 13.8. IR (film) ν (cm^{-1}) 3298, 2359, 1641, 1456, 1253, 1124, 1038, 814. HR-MS (ESI) *m/z* calcd for $C_{28}H_{40}O_4N$ 454.29519, found 454.29509 $[M+H^+]$. $[\alpha]_D^{25}$ = +22.2 (*c* 0.14, EtOAc). HPLC conditions: ADH column, *n*-hexane:*i*-PrOH = 95:5, flow rate = 1.0 mL·min^{-1}, t_R = 19.7 min (major), t_R = 23.9 min (minor).

N-[(1R,2R)-2-(4-Methoxyphenyl)-1-(tetrahydro-2H-pyran-4-yl)-2-(p-tolyl)ethyl] butyramide (2.24)

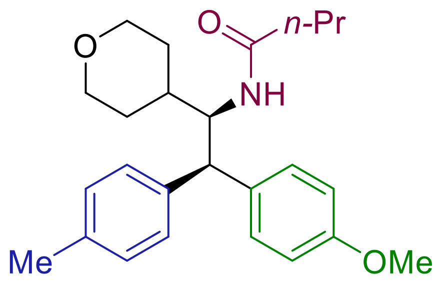

Oil, 61% yield. ^{1}H NMR (400 MHz, $CDCl_3$) δ 7.21 (d, J = 8.7 Hz, 2H), 7.13 (d, J = 8.1 Hz, 2H), 7.04 (d, J = 7.9 Hz, 2H), 6.81 (d, J = 8.7 Hz, 2H), 5.15 (ddd, J = 9.6, 6.4, 3.3 Hz, 1H), 4.82 (td, J = 10.2, 2.3 Hz, 1H), 3.98 – 3.86 (m, 3H), 3.76 (s, 3H), 3.30 – 3.13 (m, 2H), 2.25 (s, 3H), 2.00 (dt, J = 14.7, 7.4 Hz, 1H), 1.87 (dt, J = 14.4, 7.4 Hz, 1H), 1.66 – 1.46 (m, 5H), 1.44 – 1.32 (m, 2H), 0.73 (t, J = 7.4 Hz, 3H). ^{13}C NMR (101 MHz, $CDCl_3$) δ 173.2, 158.4, 139.4, 136.3, 134.7, 129.5 (2C), 128.7 (2C), 127.9 (2C), 114.4 (2C), 68.0, 67.8, 55.3, 54.5, 52.6, 38.9, 36.9, 30.9, 26.2, 21.1, 19.2, 13.7. IR (film) ν (cm^{-1}) 3298, 2925, 2359, 1641, 1507, 1253, 1124, 814. HR-MS (ESI) m/z calcd for $C_{25}H_{34}O_3N$ 396.25332, found 396.25312 [$M+H^+$]. $[\alpha]_D^{25}$ = +26.5 (c 0.14, EtOAc). HPLC conditions: ADH column, n-hexane:i-PrOH = 95:5, flow rate = 1.0 mL·min^{-1}, t_R = 41.6 min (minor), t_R = 48.4 min (major).

(2S,3R)-2-Butyramido-3-(4-methoxyphenyl)-3-(p-tolyl)propyl acetate (2.25)

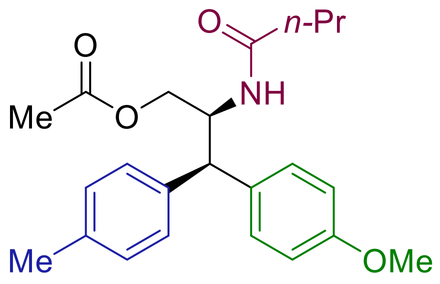

Oil, 52% yield. ^{1}H NMR (400 MHz, $CDCl_3$) δ 7.16 (dd, J = 8.3, 6.2 Hz, 4H), 7.07 (d, J = 7.9 Hz, 2H), 6.80 (d, J = 8.7 Hz, 2H), 5.28 (d, J = 9.3 Hz, 1H), 5.07 – 5.00 (m, 1H), 4.09 (dd, J = 11.3, 3.6 Hz, 1H), 4.01 (d, J = 10.4 Hz, 1H), 3.92 (dd, J = 11.3, 5.0 Hz, 1H), 3.75 (s, 3H), 2.27 (s, 3H), 2.07 (s, 3H), 1.97 (dtd, J = 21.6, 14.4, 7.1 Hz, 2H), 1.51 – 1.40 (m, 2H), 0.76 (t, J = 7.4 Hz, 3H). ^{13}C NMR (101 MHz, $CDCl_3$) δ 172.2, 170.9, 158.6, 138.3, 136.5, 133.8, 129.5 (2C), 129.0 (2C), 128.1 (2C), 114.4 (2C), 65.2, 55.4, 51.9, 50.3, 38.9, 21.1, 21.0, 19.2, 13.6. IR (film) ν (cm^{-1}) 3065, 2360, 1740, 1509, 1246, 1032, 812. HR-MS (ESI) m/z calcd for $C_{23}H_{30}O_4N$ 384.21693, found 384.21655 [$M+H^+$]. $[\alpha]_D^{25}$ = +27.1 (c 0.14, EtOAc). HPLC conditions: ADH column, n-hexane:i-PrOH = 95:5, flow rate = 1.0 mL·min^{-1}, t_R = 38.8 min (minor), t_R = 55.4 min (major).

***N*-[(1*R*,2*R*,4*R*)-1-(4-Methoxyphenyl)-4,8-dimethyl-1-(*p*-tolyl)non-7-en-2-yl]butyramide (2.26)**

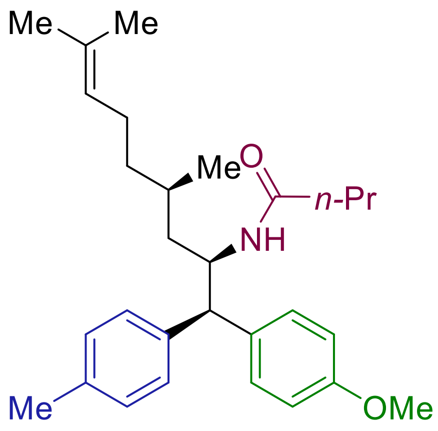

Oil, 44% yield. ^{1}H NMR (400 MHz, $CDCl_3$) δ 7.19 (d, *J* = 8.7 Hz, 2H), 7.12 (d, *J* = 8.1 Hz, 2H), 7.05 (d, *J* = 8.0 Hz, 2H), 6.81 (d, *J* = 8.7 Hz, 2H), 5.09 (t, *J* = 7.0 Hz, 1H), 4.97 (d, *J* = 9.9 Hz, 1H), 4.87 (ddd, *J* = 19.1, 9.7, 3.2 Hz, 1H), 3.83 (d, *J* = 8.2 Hz, 1H), 3.76 (s, 3H), 2.27 (s, 3H), 1.99 – 1.81 (m, 5H), 1.67 (s, 3H), 1.58 (s, 3H), 1.52 – 1.36 (m, 6H), 0.84 (d, *J* = 6.3 Hz, 3H), 0.76 (t, *J* = 7.4 Hz, 3H). ^{13}C NMR (101 MHz, $CDCl_3$) δ 172.2, 158.2, 139.4, 136.0, 135.1, 131.1, 129.29 (2C), 129.2 (2C), 128.4 (2C), 125.1, 114.1 (2C), 56.0, 55.3, 49.3, 42.3, 39.1, 35.9, 29.6, 25.9, 25.8, 21.1, 20.5, 19.2, 17.8, 13.7. IR (film) ν (cm^{-1}) 3270, 2969, 1740, 1505, 1373, 1217, 1030, 820. HR-MS (ESI) *m/z* calcd for $C_{29}H_{42}O_2N$ 436.32101, found 436.32113 [$M+H^+$]. $[\alpha]_D^{25}$ = +100.4 (*c* 0.14, EtOAc). HPLC conditions: ADH column, *n*-hexane:*i*-PrOH = 95:5, flow rate = 1.0 mL·min^{-1}, t_R = 12.3 min (major), t_R = 15.2 min (minor).

(*S*)-*N*-[2-Butyramido-1-(*p*-tolyl)ethyl]-4-methoxybenzamide (2.27)

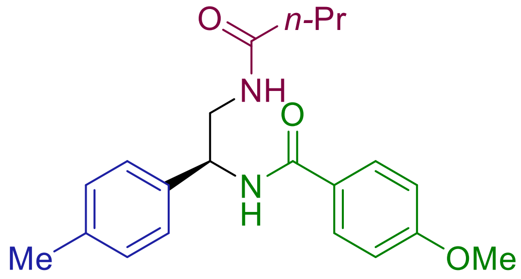

White solid, 71% yield, m.p. = 138 – 140 ºC. ^{1}H NMR (400 MHz, $CDCl_3$) δ 7.90 – 7.76 (m, 3H), 7.22 (d, *J* = 8.1 Hz, 2H), 7.14 (d, *J* = 7.9 Hz, 2H), 6.92 (d, *J* = 8.8 Hz, 2H), 6.09 (br s, 1H), 5.23 – 5.13 (m, 1H), 3.84 (s, 3H), 3.83 – 3.72 (m, 1H), 3.61 – 3.51 (m, 1H), 2.32 (s, 3H), 2.25 – 2.10 (m, 2H), 1.76 – 1.52 (m, 2H), 0.89 (t, *J* = 7.4 Hz, 3H). ^{13}C NMR (126 MHz, $CDCl_3$) δ 175.8, 167.0, 162.4, 137.5, 136.9, 129.6 (2C), 129.2 (2C), 126.5 (2C), 126.3, 113.9 (2C), 56.2, 55.5, 45.4, 38.7, 21.2, 19.3, 13.8. IR (film) ν (cm^{-1}) 3388, 2962, 1607, 1306, 1022, 814. HR-MS (ESI) *m/z* calcd for $C_{21}H_{27}O_3N_2$ 355.20162, found 355.20139 [$M+H^+$]. $[\alpha]_D^{25}$ = +18.3 (*c* 0.14, EtOAc). HPLC conditions: ADH column, *n*-hexane:*i*-PrOH = 90:10, flow rate = 1.0 mL·min^{-1}, t_R = 20.8 min (major), t_R = 57.9 min (minor).

(*S*)-*N*-[2-Butyramido-1-(*p*-tolyl)ethyl]-4-methylbenzamide (2.28)

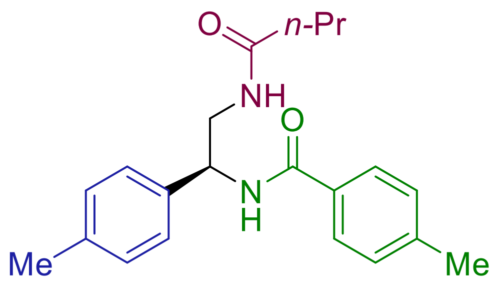

White solid, 65 % yield, m.p. = 106 – 109 ºC. ^{1}H NMR (400 MHz, $CDCl_3$) 7.86 (br d, *J* = 6.3 Hz, 1H), 7.74 (d, *J* = 8.2 Hz, 2H), 7.25 – 7.19 (m, 4H), 7.14 (d, *J* = 7.9 Hz, 2H), 6.15 (br t, *J* = 4.9 Hz, 1H), 5.17 (ddd, *J* = 9.6, 6.4, 3.3 Hz, 1H), 3.80 (ddd, *J* = 14.3, 9.4, 7.0 Hz, 1H), 3.54 (ddd, *J* = 14.3, 5.4, 3.4 Hz, 1H), 2.38 (s, 3H), 2.32 (s, 3H), 2.15 (td, *J* = 7.4, 3.8 Hz, 2H), 1.65 – 1.55 (m, 2H), 0.87 (t, *J* = 7.4 Hz, 3H). ^{13}C NMR (101 MHz, $CDCl_3$) δ 175.8, 167.5, 142.2, 137.6, 136.8, 131.1, 129.6 (2C), 129.4 (2C), 127.3 (2C), 126.5 (2C), 56.1, 45.4, 38.6, 21.6, 21.2, 19.3, 13.8. IR (film) ν (cm^{-1}) 3299, 2923, 1630, 1506, 1325, 1023, 815. HR-MS (ESI) *m/z* calcd for $C_{21}H_{27}O_2N_2$ 339.20670, found 339.20708 [$M+H^+$]. $[\alpha]_D^{25}$ = +21.3 (*c* 0.14, EtOAc). HPLC conditions: ADH column, *n*-hexane:*i*-PrOH = 95:5, flow rate = 1.0 mL·min^{-1}, t_R = 32.2 min (major), t_R = 39.1 min (minor).

(*S*)-*N*-[2-Butyramido-1-(*p*-tolyl)ethyl]- 2-methoxybenzamide (2.29)

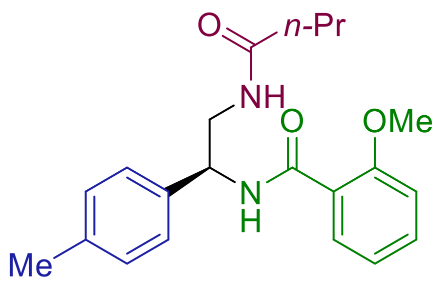

Oil, 65% yield. ^{1}H NMR (400 MHz, $CDCl_3$) δ 8.55 (br d, *J* = 7.3 Hz, 1H), 8.17 (dd, *J* = 7.8, 1.8 Hz, 1H), 7.54 – 7.38 (m, 1H), 7.31 – 7.24 (m, 2H), 7.17 (d, *J* = 8.0 Hz, 2H), 7.09 – 6.90 (m, 2H), 6.35 (br s, 1H), 5.38 – 5.26 (m, 1H), 3.96 (s, 3H), 3.83 – 3.74 (m, 1H), 3.62 (dt, *J* = 13.7, 4.2 Hz, 1H), 2.34 (s, 3H), 2.13 (td, *J* = 7.3, 2.1 Hz, 2H), 1.66 – 1.54 (m, 2H), 0.87 (t, *J* = 7.4 Hz, 3H). ^{13}C NMR (101 MHz, $CDCl_3$) δ 166.1, 157.9, 137.7, 136.8, 133.8, 133.2, 132.8, 132.4, 129.7 (2C), 126.6 (2C), 121.3, 111.5, 56.1, 54.1, 45.8, 38.8, 21.2, 19.2, 13.8. IR (film) ν (cm^{-1}) 3330, 2962, 1640, 1538, 1306, 1182, 1022, 814. HR-MS (ESI) *m/z* calcd for $C_{21}H_{27}O_3N_2$ 355.20162, found 355.20126 [$M+H^+$]. $[\alpha]_D^{25}$ = +12.6 (*c* 0.14, EtOAc). HPLC conditions: ADH column, *n*-hexane:*i*-PrOH = 70:30, flow rate = 1.0 mL·min^{-1}, t_R = 6.8 min (major), t_R = 11.5 min (minor).

(*S*)-*N*-[2-Butyramido-1-(*p*-tolyl)ethyl]- 2-methylbenzamide (2.30)

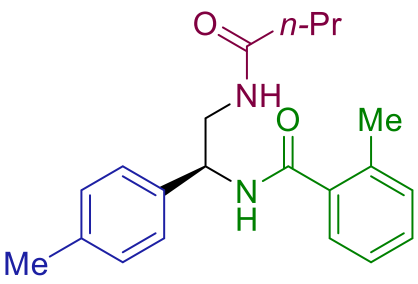

White solid, 59% yield, m.p. 131 – 132 °C. NMR (400 MHz, $CDCl_3$) δ 7.47 – 7.37 (m, 1H), 7.34 – 7.28 (m, 1H), 7.26 – 7.16 (m, 6H), 6.99 (d, *J* = 8.1 Hz, 1H), 6.14 (br s, 1H), 5.29 – 5.17 (m, 1H), 3.81 (ddd, *J* = 14.1, 9.5, 6.7 Hz, 1H), 3.81 (ddd, *J* = 14.1, 4.9, 3.7 Hz, 1H), 2.42 (s, 3H), 2.34 (s, 3H), 2.19 – 2.13 (m, 2H), 1.69 – 1.62 (m, 2H), 0.91 (t, *J* = 7.4 Hz, 3H). ^{13}C NMR (126 MHz, $CDCl_3$) δ 174.8, 170.4, 137.9, 136.6, 136.4, 135.8, 131.3, 130.2, 129.8 (2C), 127.0, 126.6 (2C), 126.0, 54.9, 45.5, 38.7, 21.2, 20.2, 19.3, 13.9. IR (film) ν (cm^{-1}) 3305, 2960, 1633, 1533, 1368, 1109, 814. HR-MS (ESI) *m/z* calcd for $C_{21}H_{27}O_2N_2$ 339.20670, found 339.20664 [M+H$^+$]; $[\alpha]_D^{25}$ = +20.1 (*c* 0.14, EtOAc). HPLC conditions: ADH column, *n*-hexane:*i*-PrOH = 95:5, flow rate = 1.0 mL·min^{-1}, t_R = 17.1 min (major), t_R = 27.5 min (minor).

***N*-[(1*S*,2*R*)-2-Butyramido-1-phenylpropyl]benzamide (2.31)**

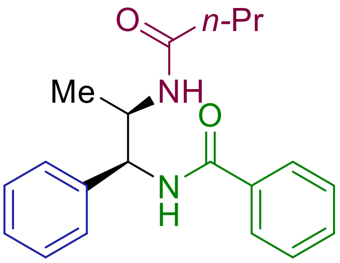

White solid, 49% yield, m.p. = 139 – 141 °C,. ^{1}H NMR (500 MHz, $CDCl_3$) ^{1}H NMR (400 MHz, $CDCl_3$) δ 9.01 (d, *J* = 5.7 Hz, 1H), 7.95 – 7.89 (m, 2H), 7.52 – 7.24 (m, 8H), 5.18 (d, *J* = 7.6 Hz, 1H), 5.05 (dd, *J* = 5.9, 2.0 Hz, 1H), 4.54 (pd, *J* = 7.1, 2.1 Hz, 1H), 2.20 (td, *J* = 7.4, 2.0 Hz, 2H), 1.70 (h, *J* = 7.6 Hz, 2H), 1.21 (d, *J* = 7.1 Hz, 3H), 0.98 (t, *J* = 7.4 Hz, 3H). ^{13}C NMR (101 MHz, $CDCl_3$) δ 175.3, 166.4, 137.9, 134.0, 131.5, 128.7, 128.3 (2C), 127.9 (2C), 127.8 (2C), 127.4 (2C), 60.8, 49. 6, 38.8, 19.4, 18.7, 14.0. IR (film) ν (cm^{-1}) 3015, 1682, 1677, 1537, 1288, 1166, 1025, 826. HR-MS (ESI) *m/z* calcd for $C_{20}H_{25}O_2N$ 325.19105, found 325.19092 [M+H$^+$]. $[\alpha]_D^{25}$ = +39.6 (*c* 0.14, EtOAc). HPLC conditions: ODH column, *n*-hexane:*i*-PrOH = 95:5, flow rate = 1.0 mL·min^{-1}, t_R = 11.6 min (major), t_R = 20.5 min (minor).

_N_-[(1_S_,2_R_)-2-Butyramido-1-(_p_-tolyl)propyl]benzamide (2.32)

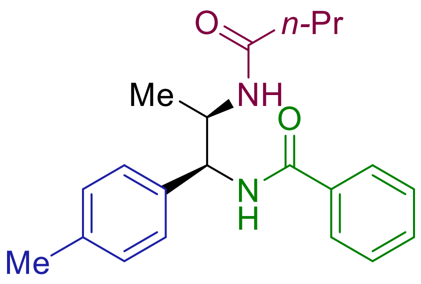

White solid, 61% yield, m.p. 151 – 153 °C. ^{1}H NMR (400 MHz, $CDCl_3$) δ 8.96 (br d, *J* = 5.8 Hz, 1H), 7.91 (d, *J* = 6.6 Hz, 2H), 7.55 – 7.41 (m, 4H), 7.18 – 7.10 (m, 3H), 5.24 (br d, *J* = 7.6 Hz, 1H), 5.00 (dd, *J* = 5.8, 2.0 Hz, 1H), 4.51 (pd, *J* = 7.1, 2.0 Hz, 1H), 2.32 (s, 3H), 2.19 (td, *J* = 7.2, 1.5 Hz, 2H), 1.70 (h, *J* = 7.4 Hz, 2H), 1.20 (d, *J* = 7.1 Hz, 3H), 0.98 (t, *J* = 7.4 Hz, 3H).^{13}C NMR (101 MHz, $CDCl_3$) δ 175.3, 166.5, 137.4, 134.8, 134.1, 131.5, 129.1 (2C), 128.7 (2C), 127.7 (2C), 127.4 (2C), 60.6, 49.6, 38.8, 21.2, 19.4, 18.6, 14.0. IR (film) ν (cm^{-1}) 3304, 2961, 1633, 1533, 1318, 1076, 814. HR-MS (ESI) *m/z* calcd for $C_{21}H_{27}O_2N_2$ 339.20670, found 339.20673 [M+H^{+}]; $[\alpha]_D^{25}$ = +28.9 (*c* 0.14, EtOAc). HPLC conditions: ADH column, *n*-hexane:*i*-PrOH = 95:5, flow rate = 1.0 mL·min^{-1}, t_R = 30.7 min (major), t_R = 58.7 min (minor).

_N_-[(1_S_,2_R_)-2-Butyramido-1-(4-methoxyphenyl)propyl]benzamide (2.33)

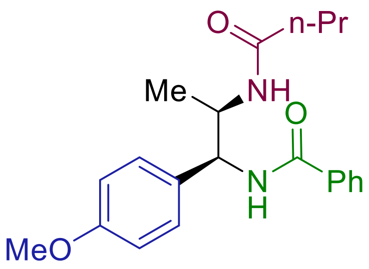

White solid, 76% yield, m.p. = 183-185 °C ^{1}H NMR (400 MHz, $CDCl_3$) δ 8.95 (d, *J* = 5.5 Hz, 1H), 7.97 – 7.86 (m, 2H), 7.50 – 7.39 (m, 3H), 7.18 (d, *J* = 8.7 Hz, 2H), 6.86 (d, *J* = 8.7 Hz, 2H), 5.20 (d, *J* = 7.4 Hz, 1H), 4.99 (dd, *J* = 5.8, 1.8 Hz, 1H), 4.59 – 4.46 (m, 1H), 3.78 (s, 3H), 2.20 (td, *J* = 7.3, 1.5 Hz, 2H), 1.71 (dd, *J* = 14.8, 7.4 Hz, 2H), 1.19 (d, *J* = 7.0 Hz, 3H), 0.99 (t, *J* = 7.4 Hz, 3H). ^{13}C NMR (101 MHz, $CDCl_3$) δ 175.2, 166.4, 159.2, 134.1, 131.5, 123.0, 128.9 (2C), 128.6 (2C), 127.4 (2C), 113.8 (2C), 60.3, 55.4, 49.6, 38.8, 19.5, 18.7, 14.0. IR (film) ν (cm^{-1}) 3299, 2964, 2169, 1642, 1487, 1356, 1167, 1042, 898. HR-MS (ESI) *m/z* calcd for $C_{21}H_{27}O_3N_2$ 355.20162, found 355.20158 [M+H^{+}]. $[\alpha]_D^{25}$ = +26.1 (*c* 0.14, EtOAc). HPLC conditions: ODH column, *n*-hexane:*i*-PrOH = 95:5, flow rate = 1.0 mL·min^{-1}, t_R = 11.0 min (major), t_R = 20.6 min (minor).

(*S*)-*N*-[1-Butyramido-2-(*p*-tolyl)propan-2-yl]benzamide (2.34)

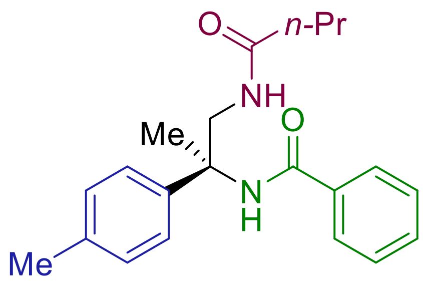

Oil, 69% yield. ^{1}H NMR (400 MHz, $CDCl_3$) 8.35 (br s, 1H), 8.01 – 7.85 (m, 2H), 7.52 – 7.36 (m, 3H), 7.23 (d, *J* = 8.3 Hz, 2H), 7.13 (d, *J* = 8.0 Hz, 2H), 6.03 (t, *J* = 6.2 Hz 1H), 3.68 (dd, *J* = 14.3, 6.9 Hz, 1H), 3.42 (dd, *J* = 14.3, 6.1 Hz, 1H), 2.31 (s, 3H), 2.19 (dd, *J* = 12.2, 4.4 Hz, 2H), 1.91 (s, 3H), 1.66 (h, *J* = 7.4 Hz, 2H), 0.93 (t, *J* = 7.4 Hz, 3H). ^{13}C NMR (101 MHz, $CDCl_3$) δ 175.9, 166.5, 139.8, 136.7, 134.6, 131.5, 129.4 (2C), 128.6 (2C), 127.3 (2C), 125.4 (2C), 61.2, 52.1, 38.7, 23.5, 21.1, 19.4, 13.9. IR (film) ν (cm^{-1}) 3304, 2962, 1633, 1538, 1339, 1293, 716, 693. HR-MS (ESI) *m/z* calcd for $C_{21}H_{27}O_2N_2$ 339.20670, found 339.20604 [$M+H^+$]; $[\alpha]_D^{25}$ = +17.3 (*c* 0.14, EtOAc). HPLC conditions: ADH column, *n*-hexane:*i*-PrOH = 95:5, flow rate = 1.0 mL·min^{-1}, t_R = 10.0 min (minor), t_R = 12.2 min (major).

(*S*)-*N*-(2-Butoxy-2-(*p*-tolyl)ethyl)butyramide (2.35)

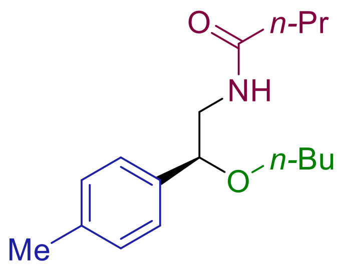

Oil, 63% yield. ^{1}H NMR (400 MHz, $CDCl_3$) δ 7.19 (d, *J* = 8.2 Hz, 2H), 7.16 (d, *J* = 8.1 Hz, 2H), 5.86 (s, 1H), 4.31 (dd, *J* = 8.9, 4.0 Hz, 1H), 3.69 (ddd, *J* = 13.8, 7.7, 4.0 Hz, 1H), 3.37 (dt, *J* = 9.3, 6.6 Hz, 1H), 3.25 (dt, *J* = 9.3, 6.5 Hz, 1H), 3.20 – 3.10 (m, 1H), 2.34 (s, 3H), 2.22 – 2.12 (m, 2H), 1.72 – 1.61 (m, 2H), 1.54 (dt, *J* = 10.6, 6.8 Hz, 2H), 1.36 (ddd, *J* = 11.0, 7.3, 3.2 Hz, 2H), 0.95 (t, *J* = 7.4 Hz, 3H), 0.89 (t, *J* = 7.4 Hz, 3H). ^{13}C NMR (101 MHz, $CDCl_3$) δ 173.0, 137.81, 136.9, 129.3 (2C), 126.7 (2C), 80.5, 68.8, 45.8, 38.9, 32.0, 21.3, 19.5, 19.3, 14.0, 13.9. IR (film) ν (cm^{-1}) 3299, 2965,1642, 1549, 1487, 1228, 1019, 899. HR-MS (ESI) *m/z* calcd for $C_{17}H_{27}O_2NNa$ 300.19319, found 300.19340 [$M+Na^+$]. $[\alpha]_D^{25}$ = +33.8 (*c* 0.14, EtOAc). HPLC conditions: ADH column, *n*-hexane:*i*-PrOH = 95:5, flow rate = 1.0 mL·min^{-1}, t_R = 7.8 min (major), t_R = 11.5 min (minor).

(*S*)-*N*-[2-*tert*-Butoxy-2-(*p*-tolyl)ethyl]butyramide (2.36)

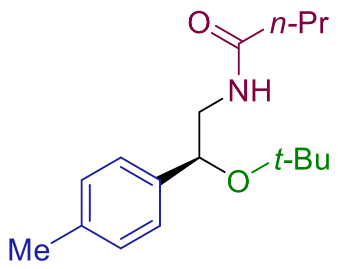

Oil, 59% yield. ^{1}H NMR (400 MHz, $CDCl_3$) δ 7.24 (d, *J* = 8.1 Hz, 2H), 7.12 (d, *J* = 7.8 Hz, 2H), 5.78 (s, 1H), 4.59 (dd, *J* = 8.7, 4.1 Hz, 1H), 3.59 (ddd, *J* = 13.7, 7.6, 4.2 Hz, 1H), 3.01 (ddd, *J* = 13.4, 8.7, 4.3 Hz, 1H), 2.33 (s, 3H), 2.18 – 2.11 (m, 2H), 1.70 – 1.60 (m, 2H), 1.13 (s, 9H), 0.94 (t, *J* = 7.4 Hz, 3H). ^{13}C NMR (101 MHz, $CDCl_3$) δ 172.9, 140.4, 137.0, 129.1 (2C), 126.2 (2C), 74.8, 72.9, 47.0, 38.9, 28.8, 21.3, 19.2, 14.0. IR (film) ν (cm^{-1}) 3200, 2970,1642, 1550, 1478, 1227, 1019, 898. HR-MS (ESI) *m/z* calcd for $C_{17}H_{27}O_2NNa$ 300.19312, found 300.19355 [M+Na$^+$]. $[\alpha]_D^{25}$ = +60.5 (*c* 0.14, EtOAc). HPLC conditions: ODH column, *n*-hexane:*i*-PrOH = 95:5, flow rate = 1.0 mL·min^{-1}, t_R = 7.1 min (major), t_R = 10.2min (minor).

(*S*)-*N*-[2-Phenoxy-2-(*p*-tolyl)ethyl]butyramide (2.37)

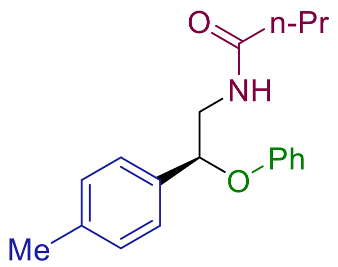

Oil, 76% yield. ^{1}H NMR (400 MHz, $CDCl_3$) δ 7.28 – 7.24 (m, 2H), 7.24 – 7.10 (m, 3H), 6.92 – 6.81 (m, 3H), 5.89 (s, 1H), 5.23 (dd, *J* = 8.7, 3.7 Hz, 1H), 3.89 (ddd, *J* = 14.0, 7.7, 3.8 Hz, 1H), 3.48 – 3.36 (m, 1H), 2.32 (s, 3H), 2.19 – 2.12 (m, 2H), 1.64 (dq, *J* = 14.7, 7.4 Hz, 2H), 0.91 (t, *J* = 7.4 Hz, 3H). ^{13}C NMR (101 MHz, $CDCl_3$) δ 173.2, 157.9, 137.8, 135.8, 129.6 (2C), 129.6 (2C), 126.2 (2C), 121.2, 115.9 (2C), 78.8, 46.1, 42.3, 38.8, 19.2, 11.4. IR (film) ν (cm^{-1}) 3299, 2169, 1642, 1487, 1227, 1019, 898. HR-MS (ESI) *m/z* calcd for $C_{19}H_{23}O_2NNa$ 336.13604, found 336.13614 [M+Na$^+$]. $[\alpha]_D^{25}$ = +71.2 (*c* 0.14, EtOAc). HPLC conditions: ADH column, *n*-hexane:*i*-PrOH = 95:5, flow rate = 1.0 mL·min^{-1}, t_R = 17.7 min (major), t_R = 23.5 min (minor).

(*S*)-*N*-[2-Methoxy-2-(*p*-tolyl)propyl]butyramide (2.38)

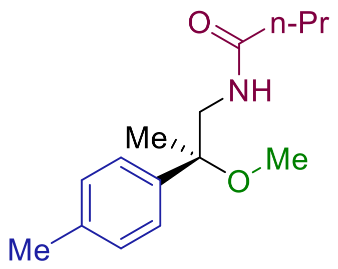

Oil, 69% yield. ^{1}H NMR (400 MHz, $CDCl_3$) δ 7.25 (d, *J* = 9.1 Hz, 2H), 7.16 (d, *J* = 8.2 Hz, 2H), 5.71 (s, 1H), 3.66 (dd, *J* = 13.7, 7.1 Hz, 1H), 3.33 (dd, *J* = 13.6, 4.3 Hz, 1H), 3.10 (s, 3H), 2.34 (s, 3H), 2.16 – 2.09 (m, 2H), 1.63 (dd, *J* = 14.8, 7.4 Hz, 2H), 1.51 (s, 3H), 0.92 (t, *J* = 7.4 Hz, 3H). ^{13}C NMR (101 MHz, $CDCl_3$) δ 173.4, 139.9, 137.6, 129.6 (2C), 126.5 (2C), 79.0, 50.8, 49.3, 39.3, 21.5, 21.5, 19.7, 14.2. IR (film) ν (cm^{-1}) 3299, 2169, 1642, 1487, 1227, 1019, 898. HR-MS (ESI) *m/z* calcd for $C_{15}H_{24}O_2N$ 250.18016, found 250.18043 [M+H$^+$]. $[\alpha]_D^{25}$ = +89.7 (*c* 0.14, EtOAc). HPLC conditions: ADH column, *n*-hexane:*i*-PrOH = 95:5, flow rate = 1.0 mL·min^{-1}, t_R = 17.0 min (major), t_R = 30.3 min (minor).

(1*R*,2*R*)-1-(4-Methoxyphenyl)-1-(*p*-tolyl)propan-2-amine (2.39)

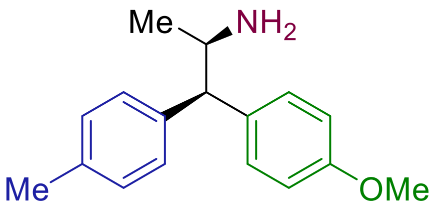

Oil, 64% yield. ^{1}H NMR (400 MHz, $CDCl_3$) 7.23 (d, *J* = 8.1 Hz, 2H), 7.17 (d, *J* = 8.7 Hz, 2H), 7.10 (d, *J* = 7.8 Hz, 2H), 6.80 (d, *J* = 8.8 Hz, 2H), 3.77 – 3.67 (m, 4H), 3.58 (d, *J* = 10.2 Hz, 1H), 2.50 (brs, 2H), 2.28 (s, 3H), 1.10 (d, *J* = 6.2 Hz, 3H).^{13}C NMR (101 MHz, $CDCl_3$) δ 158.2, 139.9, 136.4, 135.5, 129.7, 129.0, 128.1, 114.1, 60.0, 55.3, 50.5, 21.5, 21.1. IR (film) ν (cm^{-1}) 3750, 3734, 2980, 2158, 1758, 1372, 1233, 814. HR-MS (ESI) *m/z* calcd for $C_{17}H_{22}ON$ 256.16959, found 256.16948 [M+H$^+$]; $[\alpha]_D^{25}$ = +15.8 (*c* 0.14, EtOAc). HPLC conditions: ADH column, *n*-hexane:*i*-PrOH = 90:10, flow rate = 0.8 mL·min^{-1}, t_R = 9.9 min (minor), t_R = 11.8 min (major).

1-[(2*R*,3*S*)-3-(4-Methoxyphenyl)-2-methylindolin-1-yl]butan-1-one (2.40)

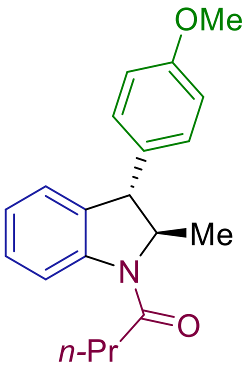

Oil, 98% yield. ^{1}H NMR (500 MHz, $CDCl_3$) δ 8.36 – 8.18 (m, 1H), 7.27 (t, J = 7.1 Hz, 1H), 7.11 (d, J = 7.4 Hz, 1H), 7.04 (td, J = 7.4, 1.1 Hz, 1H), 4.36 – 4.18 (m, 1H), 3.98 – 3.82 (m, 1H), 3.76 (s, 3H), 2.52 – 2.29 (m, 1H), 1.74 (q, J = 7.4 Hz, 2H), 1.44 (d, J = 6.4 Hz, 3H), 1.33 – 1.18 (m, 1H), 0.97 (t, J = 7.1, 1H). ^{13}C NMR (126 MHz, $CDCl_3$) δ 171.3, 158.7, 141.7, 136.2, 134.0, 128.3, 127.9 (2C), 125.8, 124.3, 118.3, 114.3 (2C), 65.0, 56.0, 53.9, 37.1, 29.8, 18. 7, 14.0. IR (film) ν (cm^{-1}) 2975, 1685, 1512, 1275, 1198, 1043, 811. HR-MS (ESI) *m/z* calcd for $C_{20}H_{24}O_2N$ 310.18016, found 310.18004 [M+H$^+$]. $[\alpha]_D^{25}$ = +141.3 (*c* 0.14, EtOAc). HPLC conditions: ADH column, *n*-hexane:*i*-PrOH = 95:5, flow rate = 1.0 mL·min^{-1}, t_R = 11.7 min (major), t_R = 20.6 min (minor).

X-Ray Crystallographic data

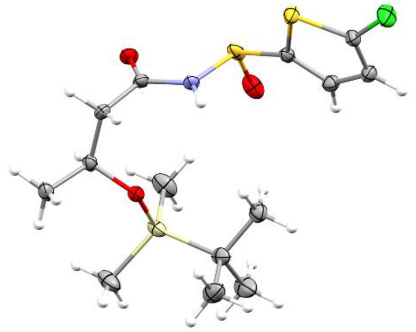

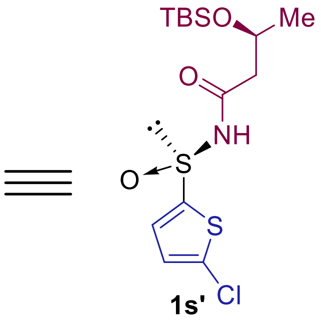

X-Ray–Crystallographic Data for **1s’** (CCDC number: 2270374)
ORTEP representation of the molecule (50% probability ellipsoids; H-atoms given arbitrary displacement parameters for clarity)

Identification code	KIR0905
Empirical formula	$C_{14}H_{24}ClNO_3S_2Si$
Formula weight	382.00
Temperature/K	160.0(1)
Crystal system	orthorhombic
Space group	$P2_12_12$
a/Å	24.2525(3)
b/Å	9.17680(10)
c/Å	8.76130(10)
α/°	90
β/°	90
γ/°	90
Volume/Å^3	1949.92(4)
Z	4
ρ_{calc}g/cm^3	1.301
μ/mm^{-1}	4.412
F(000)	808.0
Crystal size/mm^3	0.2 × 0.15 × 0.08
Radiation	Cu Kα (λ = 1.54184)
2Θ range for data collection/°	7.29 to 148.968
Index ranges	$-30 \le h \le 27$, $-11 \le k \le 9$, $-10 \le l \le 10$
Reflections collected	19748
Independent reflections	3989 [R_{int} = 0.0313, R_{sigma} = 0.0170]
Data/restraints/parameters	3989/0/209
Goodness-of-fit on F^2	1.065
Final R indexes [I>=2σ (I)]	R_1 = 0.0228, wR_2 = 0.0607
Final R indexes [all data]	R_1 = 0.0236, wR_2 = 0.0617
Largest diff. peak/hole / e Å^{-3}	0.22/-0.19
Flack parameter	-0.014(5)

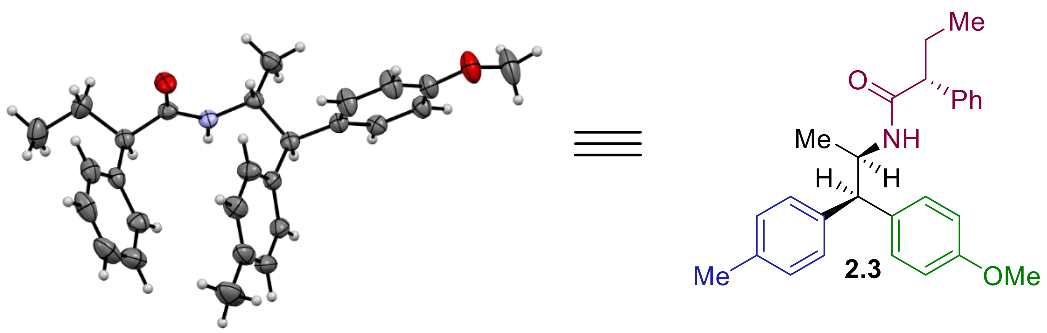

X-Ray–Crystallographic Data for **2c** (CCDC number: 2212927)

ORTEP representation of the molecule (50% probability ellipsoids; H-atoms given arbitrary displacement parameters for clarity)

Crystallised from		CH_2Cl_2 / *n*-hexane
Empirical formula		$C_{27}H_{31}NO_2$
Formula weight [g mol^{-1}]		401.53
Crystal colour, habit		colourless, needle
Crystal dimensions [mm]		0.07 × 0.08 × 0.26
Temperature [K]		160(1)
Crystal system		tetragonal
Space group		$P4_1$ (#76)
Z		4
Reflections for cell determination		16157
2θ range for cell determination [°]		8–146
Unit cell parameters	a [Å]	10.85493(6)
	b [Å]	10.85493(6)
	c [Å]	19.81352(17)
	α [°]	90
	β [°]	90
	γ [°]	90
	V [Å^3]	2334.62(3)
$F(000)$		864
D_x [g cm^{-3}]		1.142
μ(Cu $K\alpha$) [mm^{-1}]		0.553
Scan type		ω
$2\theta_{(max)}$ [°]		146.1
Transmission factors (min; max)		0.335; 0.958

Total reflections measured	22077
Symmetry independent reflections	4608
R_{int}	0.019
Reflections with $I > 2\sigma(I)$	4484
Reflections used in refinement	4608
Parameters refined; restraints	280; 1
Final $R(F)$ [$I > 2\sigma(I)$ reflections]	0.0254
$wR(F^2)$ (all data)	0.0662
Weights:	$w = [\sigma^2(F_o^2) + (0.0370P)^2 + 0.1980P]^{-1}$ where $P = (F_o^2 + 2F_c^2)/3$
Goodness of fit	1.026
Secondary extinction coefficient	0.0005(1)
Final Δ_{max}/σ	0.000
$\Delta\rho$ (max; min) [e Å^{-3}]	0.10; -0.09
$\sigma(d_{(C-C)})$ [Å]	0.002 – 0.003

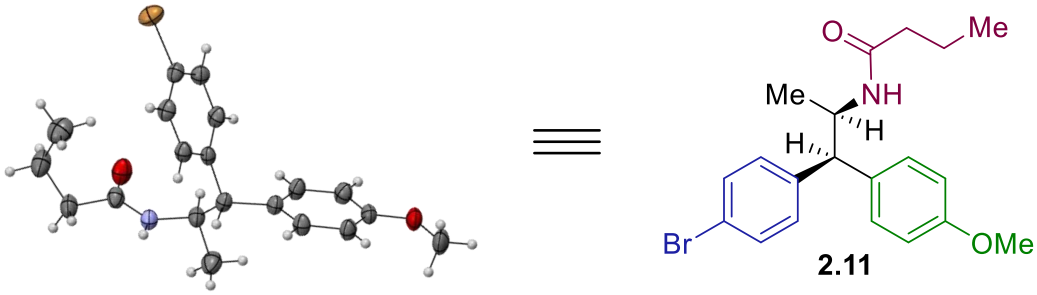

2.11

X-Ray–Crystallographic Data for **2.11** (CCDC number: 2212932)

ORTEP representation of the molecule (50% probability ellipsoids; H-atoms given arbitrary displacement parameters for clarity)

Crystallised from	CH_2Cl_2 / *n*-pentane
Empirical formula	$C_{20}H_{24}BrNO_2$
Formula weight [g mol^{-1}]	390.31
Crystal colour, habit	colourless, irregular prism
Crystal dimensions [mm]	0.05 × 0.06 × 0.10
Temperature [K]	160(1)
Crystal system	monoclinic
Space group	*C*2 (#5)
Z	4

Reflections for cell determination	14142
2θ range for cell determination [°]	8 – 153
Unit cell parameters a [Å]	19.9825(4)
b [Å]	9.4160(2)
c [Å]	10.3129(2)
α [°]	90
β [°]	97.590(2)
γ [°]	90
V [Å^3]	1923.43(7)
$F(000)$	808
D_x [g cm^{-3}]	1.348
μ(Cu $K\alpha$) [mm^{-1}]	2.994
Scan type	ω
$2\theta_{(max)}$ [°]	153.3
Transmission factors (min; max)	0.808; 0.946
Total reflections measured	19868
Symmetry independent reflections	3999
R_{int}	0.029
Reflections with $I > 2\sigma(I)$	3892
Reflections used in refinement	3999
Parameters refined; restraints	224; 1
Final $R(F)$ [$I > 2\sigma(I)$ reflections]	0.0264
$wR(F^2)$ (all data)	0.0693
Weights:	$w = [\sigma^2(F_o^2) + (0.0382P)^2 + 1.1179P]^{-1}$ where $P = (F_o^2 + 2F_c^2)/3$
Goodness of fit	1.029
Final Δ_{max}/σ	0.001
$\Delta\rho$ (max; min) [e Å^{-3}]	0.48; -0.41
$\sigma(d_{(C-C)})$ [Å]	0.003 – 0.006

Copies of NMR spectra and HPLC traces

(*S*)-Benzenesulfinamide

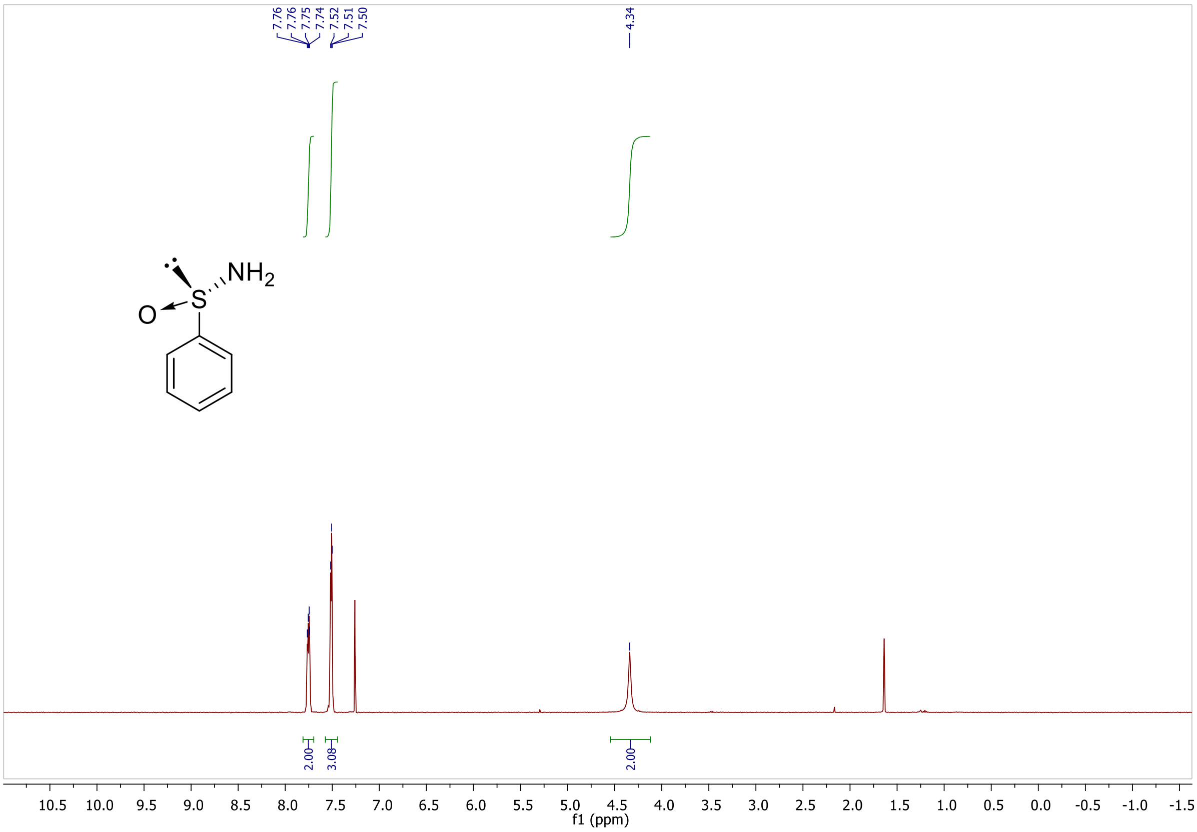
7.76
7.76
7.75
7.74
7.52
7.51
7.50
4.34
NH2
O
S
2.00
3.08
2.00
10.5 10.0 9.5 9.0 8.5 8.0 7.5 7.0 6.5 6.0 5.5 5.0 4.5 4.0 3.5 3.0 2.5 2.0 1.5 1.0 0.5 0.0 -0.5 -1.0 -1.5
f1 (ppm)

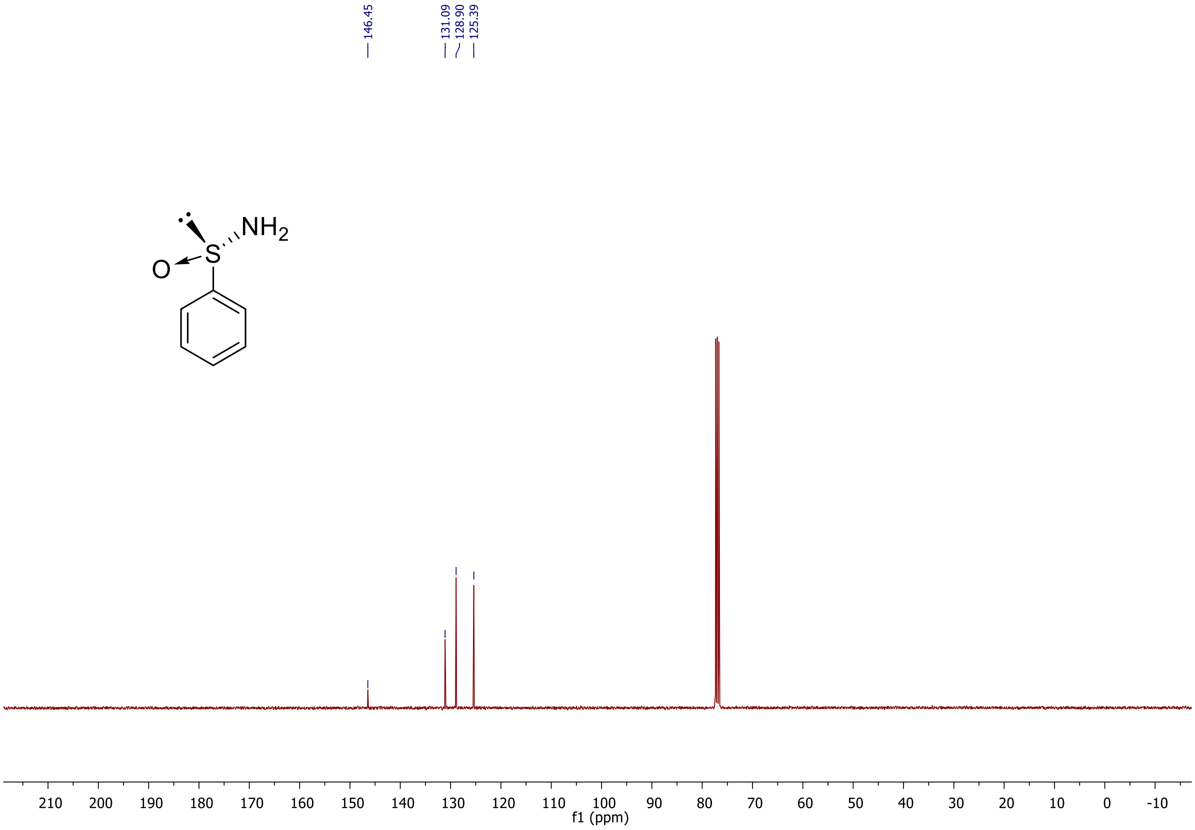
146.45
131.09
128.90
125.39
NH2
O
S
210 200 190 180 170 160 150 140 130 120 110 100 90 80 70 60 50 40 30 20 10 0 -10
f1 (ppm)

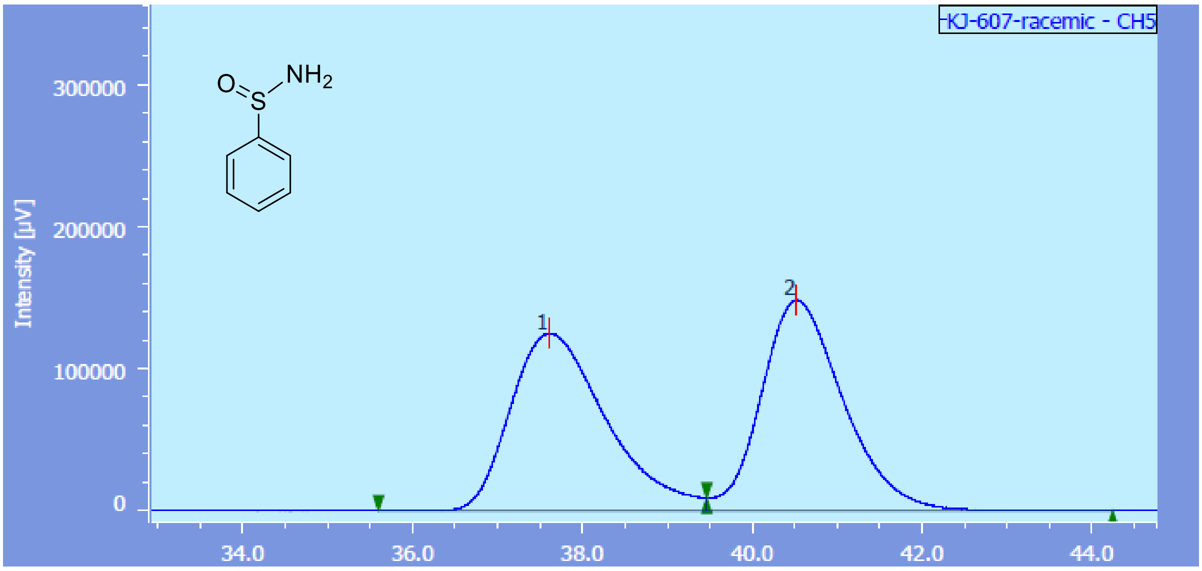

#	Peak Name	CH	tR [min]	Area [µV·sec]	Height [µV]	Area%	Height%	Quantity	NTP	Resolution	Symmetry Factor	Warning
1	Unknown	5	37.607	9792905	124957	49.592	45.742	N/A	5480	1.552	N/A	
2	Unknown	5	40.520	9953984	148223	50.408	54.258	N/A	8765	N/A	N/A	

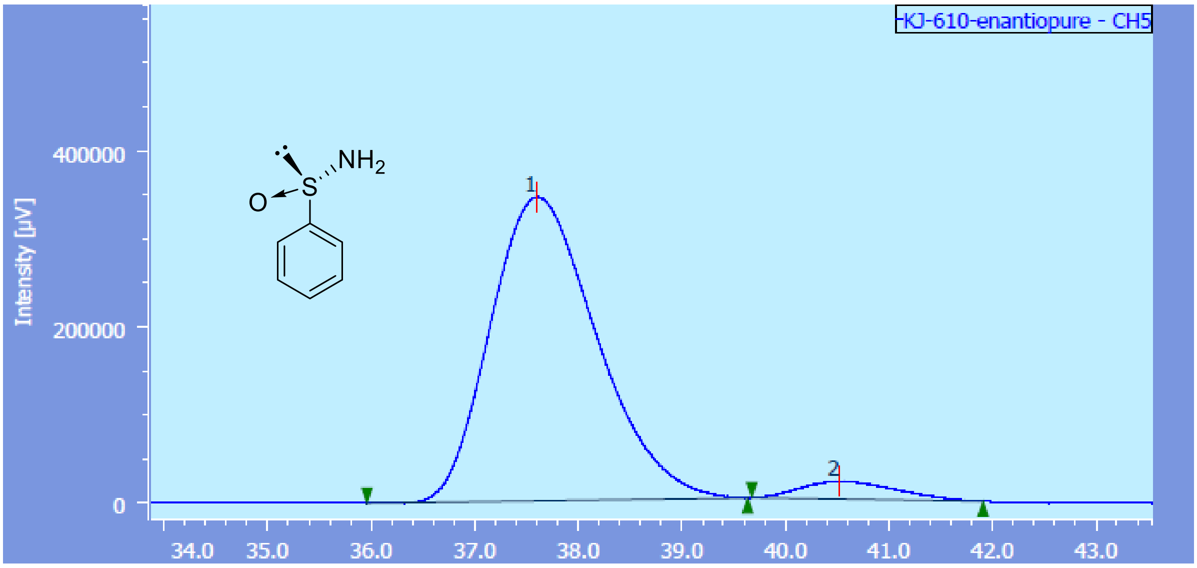

#	Peak Name	CH	tR [min]	Area [µV·sec]	Height [µV]	Area%	Height%	Quantity	NTP	Resolution	Symmetry Factor	Warning
1	Unknown	5	37.603	24600037	344837	94.923	94.414	N/A	6273	1.595	1.264	
2	Unknown	5	40.527	1315731	20404	5.077	5.586	N/A	8336	N/A	1.302	

(*S*)-4-Bromobenzenesulfinamide

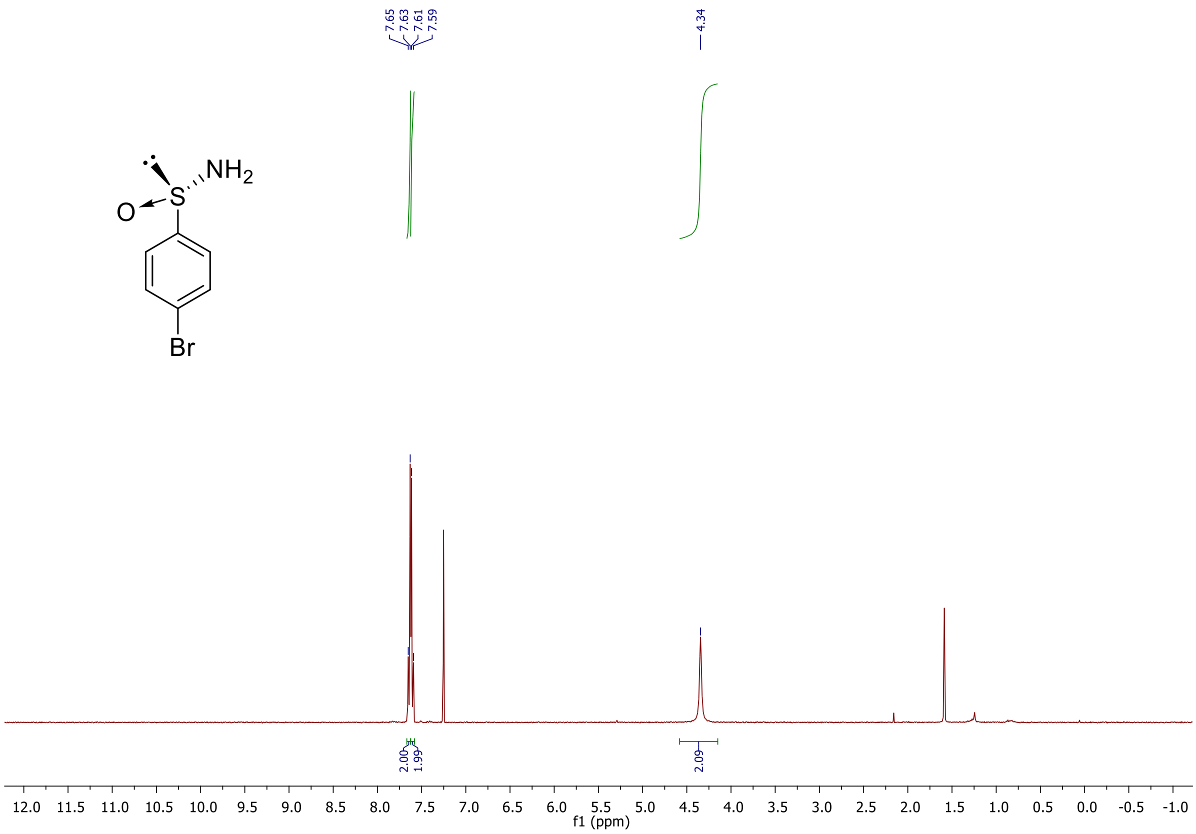

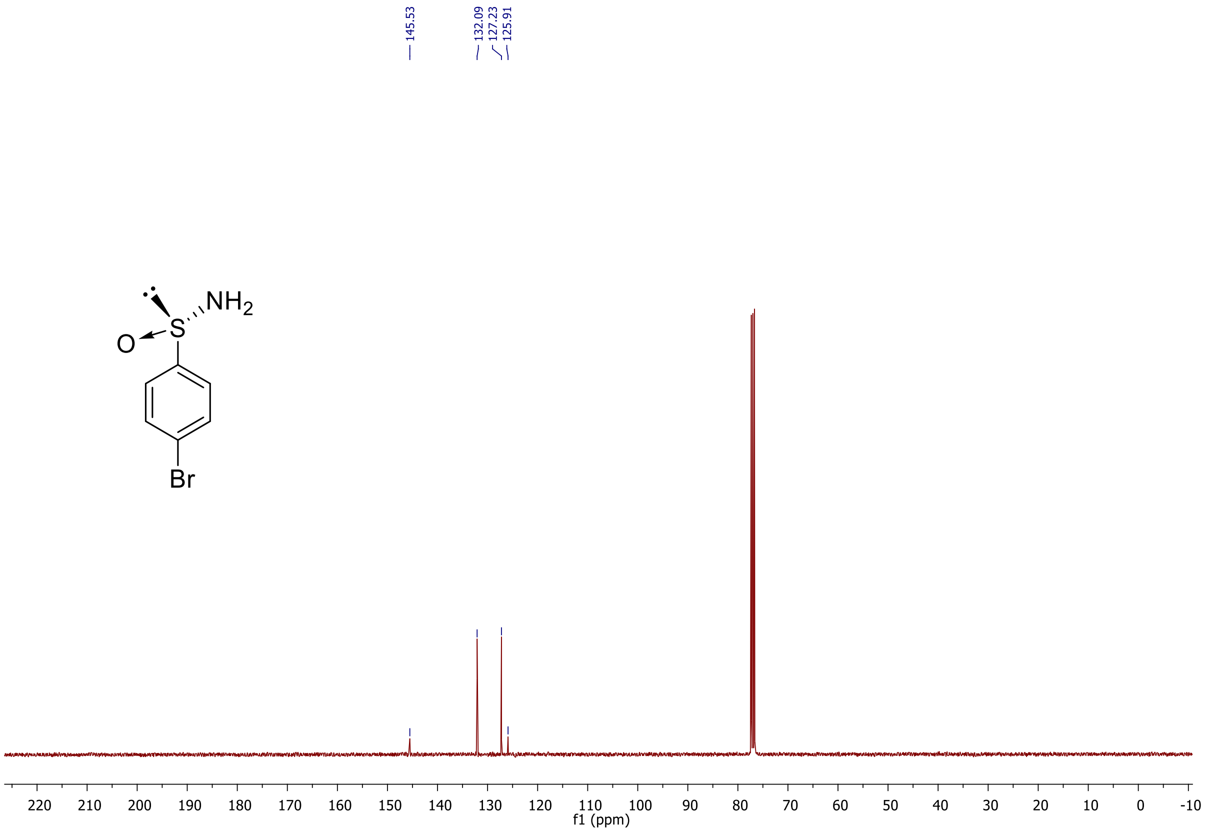

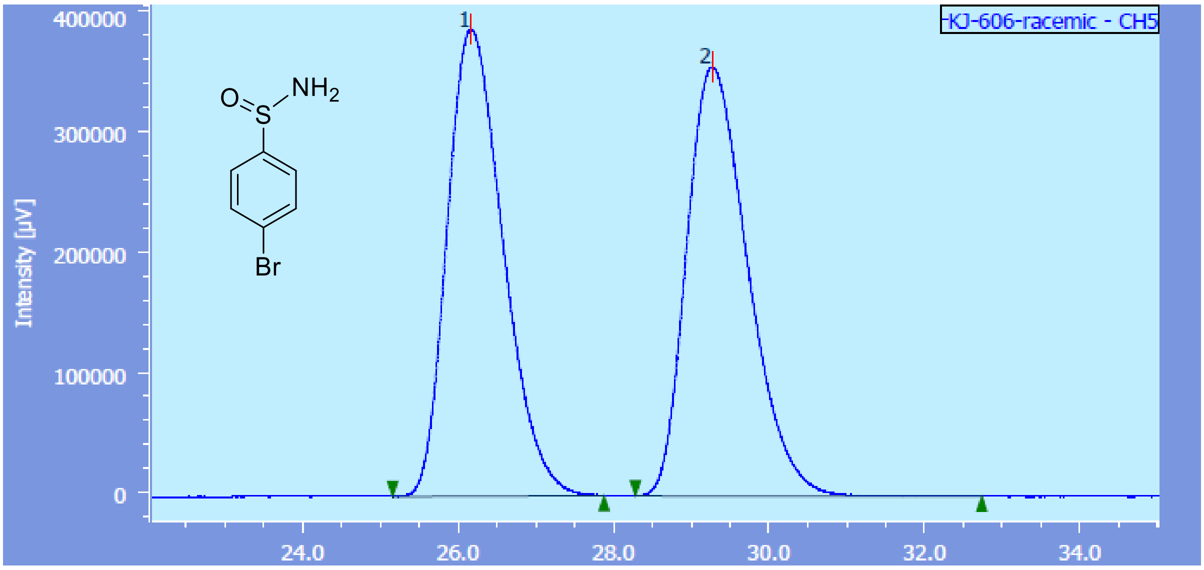

#	Peak Name	CH	tR [min]	Area [µV·sec]	Height [µV]	Area%	Height%	Quantity	NTP	Resolution	Symmetry Factor	Warning
1	Unknown	5	26.163	19423094	387960	49.859	52.124	N/A	6220	2.242	1.307	
2	Unknown	5	29.267	19533189	356335	50.141	47.876	N/A	6524	N/A	1.344	

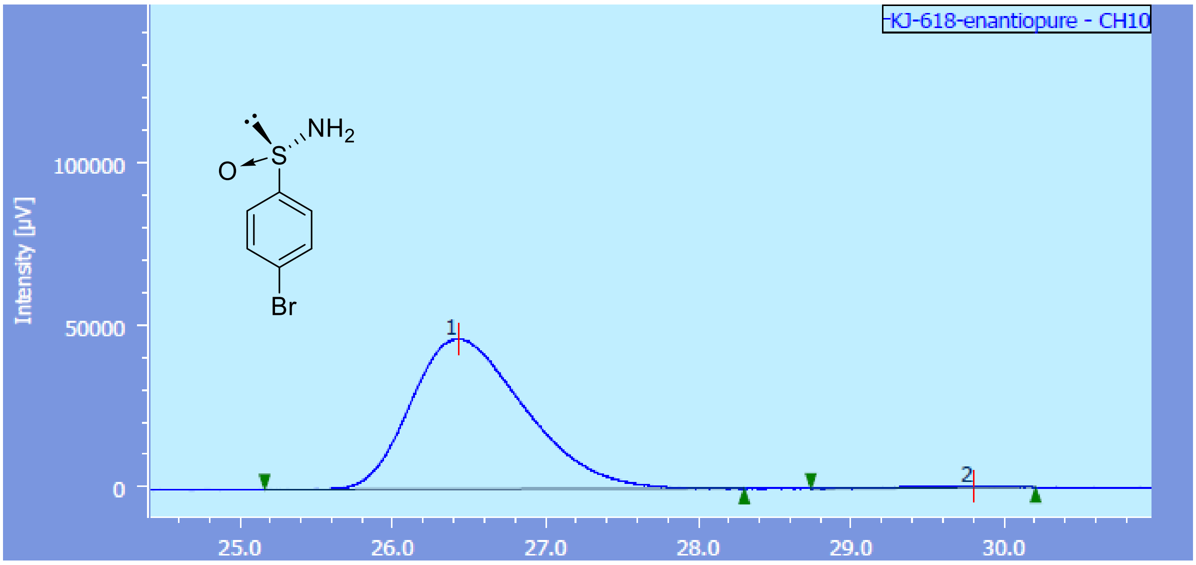

#	Peak Name	CH	tR [min]	Area [µV·sec]	Height [µV]	Area%	Height%	Quantity	NTP	Resolution	Symmetry Factor	Warning
1	Unknown	10	26.427	2410273	46436	99.208	99.070	N/A	5916	2.569	1.355	
2	Unknown	10	29.800	19240	436	0.792	0.930	N/A	8963	N/A	0.710	

(*S*)-4-Fluorobenzenesulfinamide

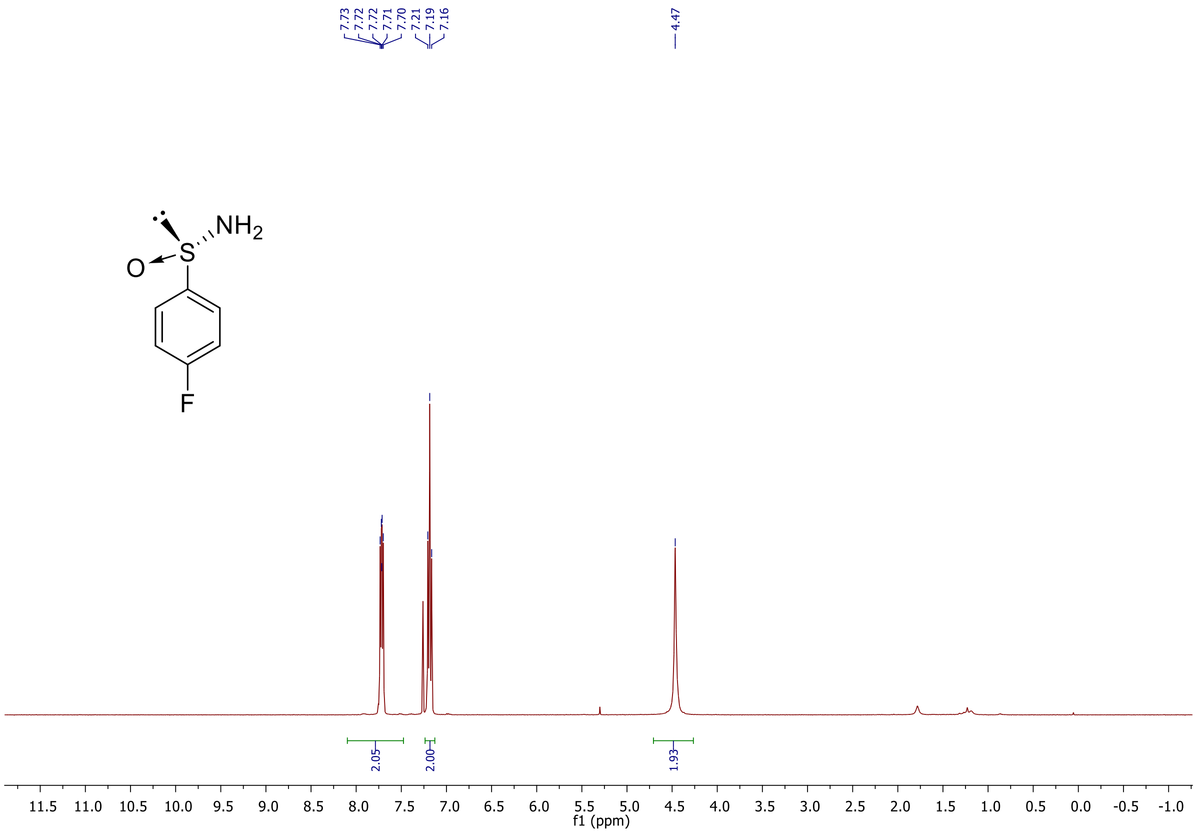

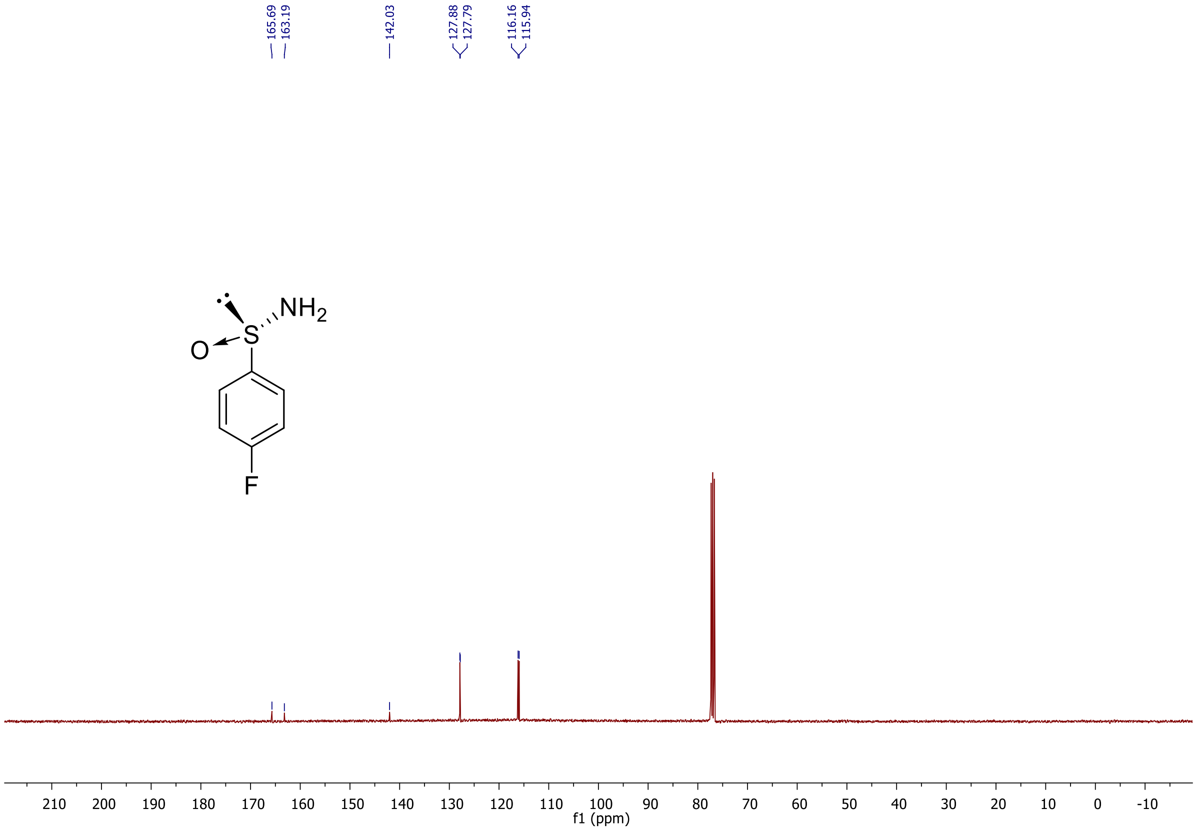

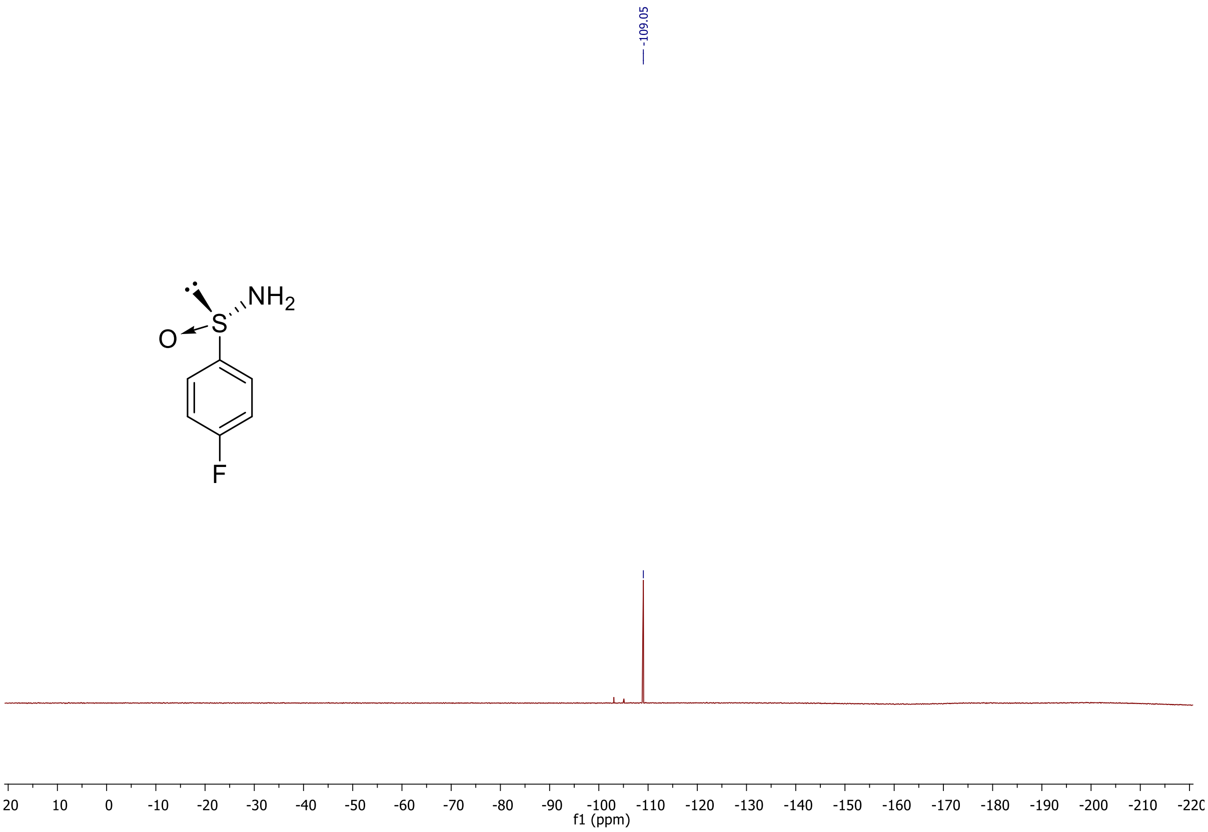

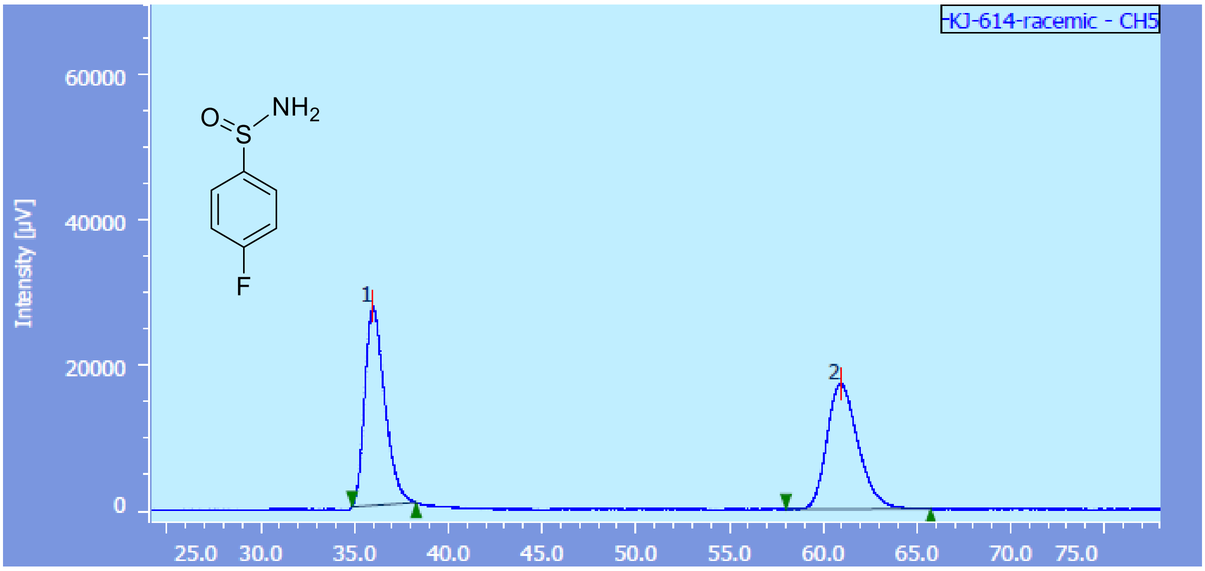

#	Peak Name	CH	tR [min]	Area [µV·sec]	Height [µV]	Area%	Height%	Quantity	NTP	Resolution	Symmetry Factor	Warning
1	Unknown	5	35.983	2030498	27350	50.292	61.281	N/A	5534	10.100	1.388	
2	Unknown	5	60.917	2006917	17281	49.708	38.719	N/A	6530	N/A	1.276	

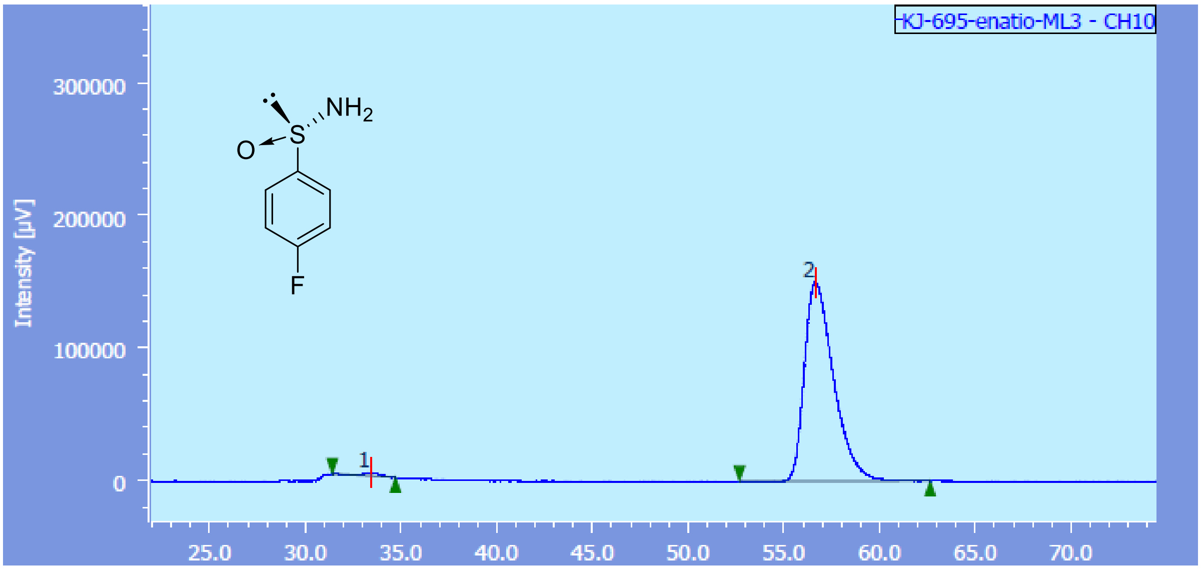

#	Peak Name	CH	tR [min]	Area [µV·sec]	Height [µV]	Area%	Height%	Quantity	NTP	Resolution	Symmetry Factor	Warning
1	Unknown	10	33.407	179996	2708	1.118	1.775	N/A	6718	10.678	0.781	
2	Unknown	10	56.643	15918480	149893	98.882	98.225	N/A	6869	N/A	1.522	

4-(Trifluoromethyl)benzenesulfinamide

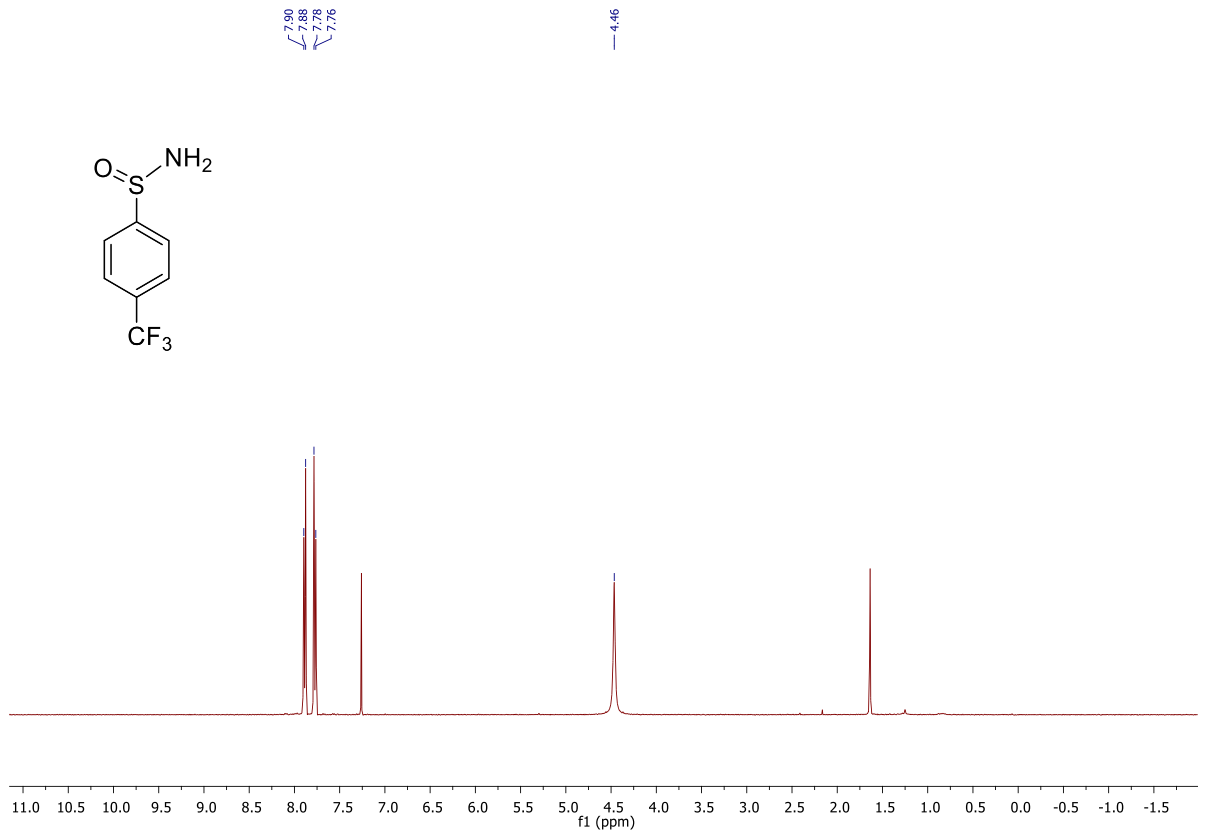

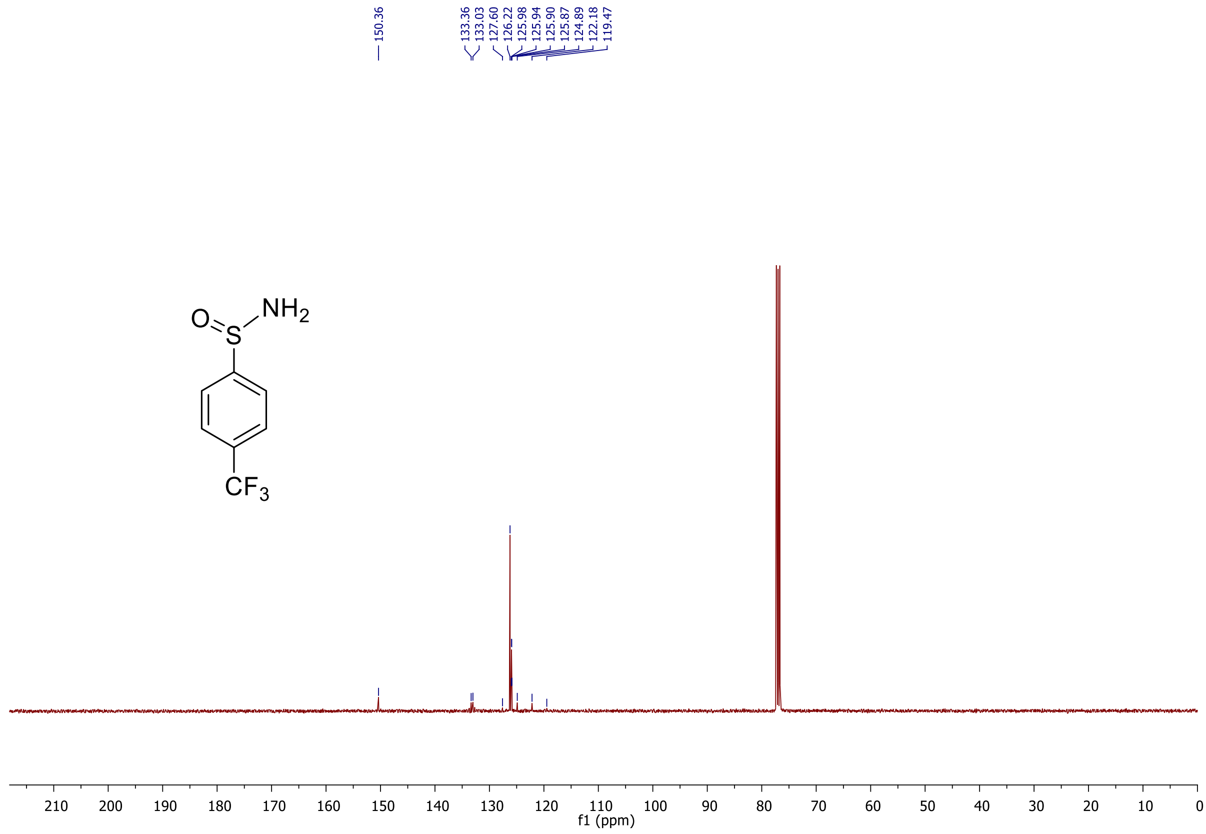

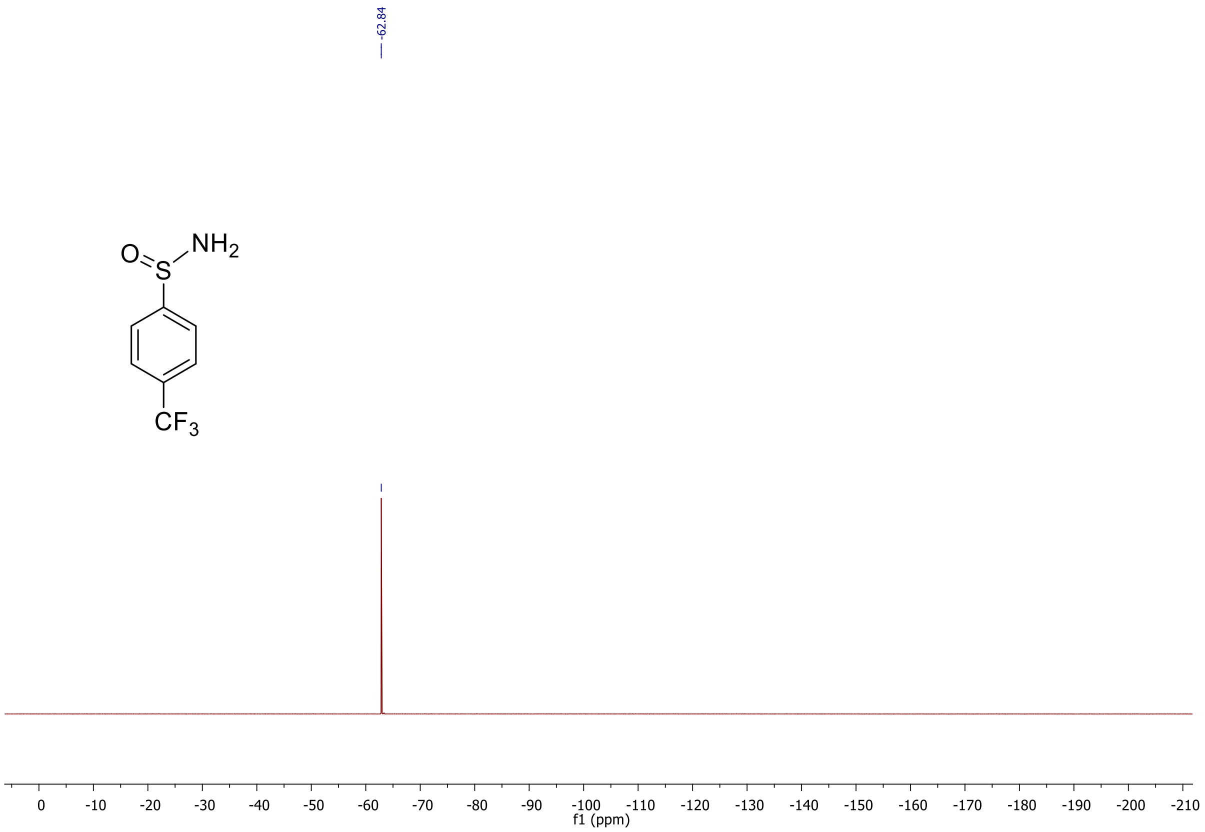
-62.84
O
S
NH2
CF3
0 -10 -20 -30 -40 -50 -60 -70 -80 -90 -100 -110 -120 -130 -140 -150 -160 -170 -180 -190 -200 -210
f1 (ppm)

(*S*)-4-Methoxybenzenesulfinamide

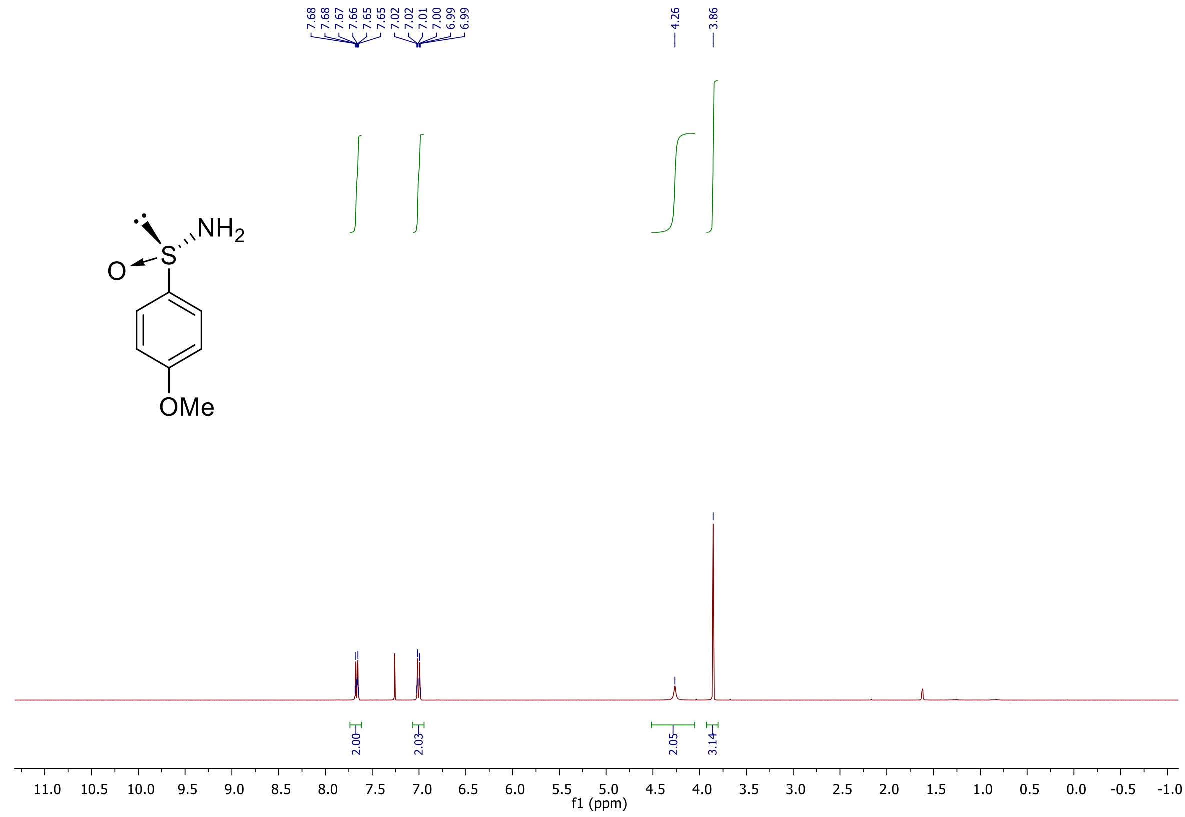

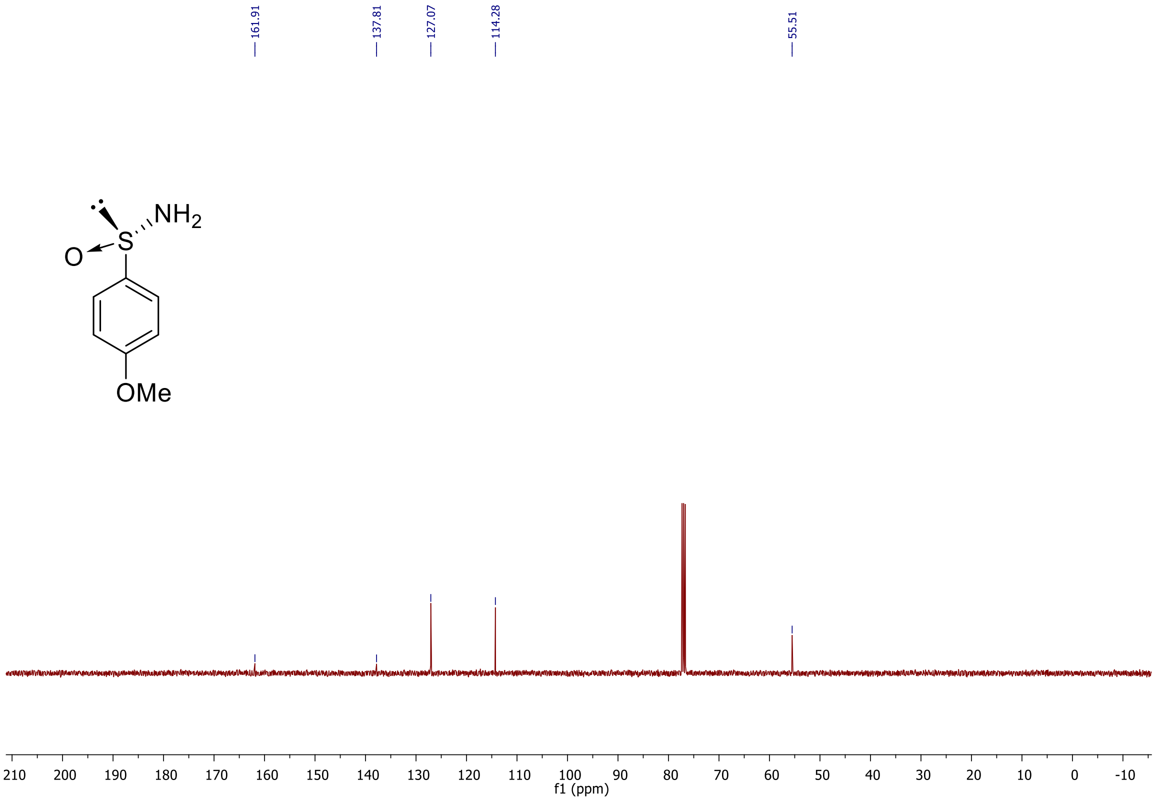

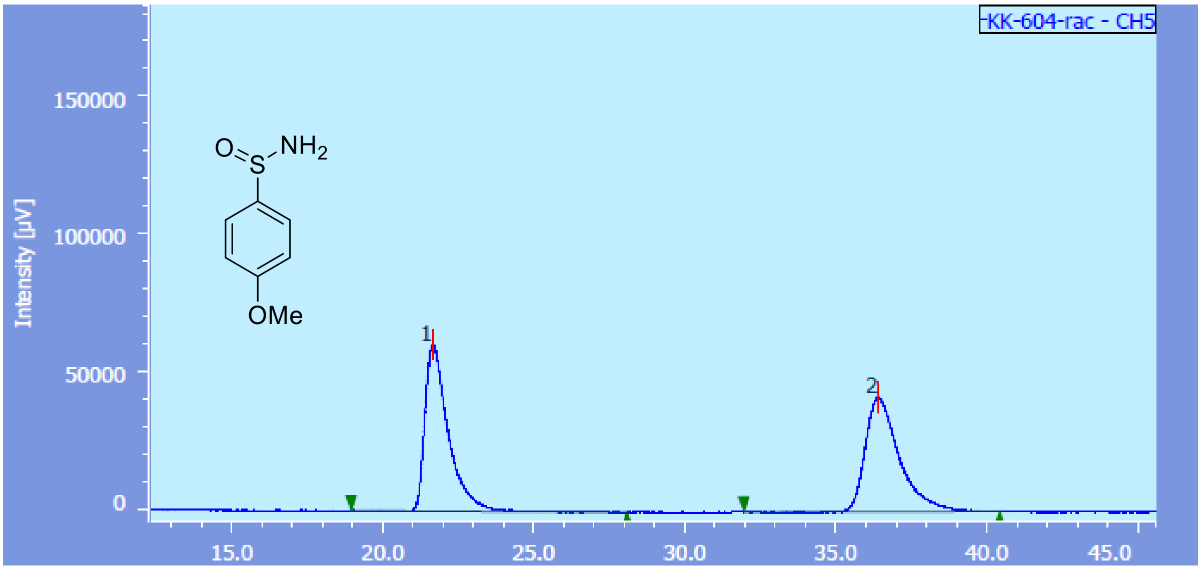

#	Peak Name	CH	tR [min]	Area [µV·sec]	Height [µV]	Area%	Height%	Quantity	NTP	Resolution	Symmetry Factor	Warning
1	Unknown	5	21.653	3242020	60306	49.931	59.233	N/A	4431	9.175	1.936	
2	Unknown	5	36.403	3250968	41506	50.069	40.767	N/A	5736	N/A	1.648	

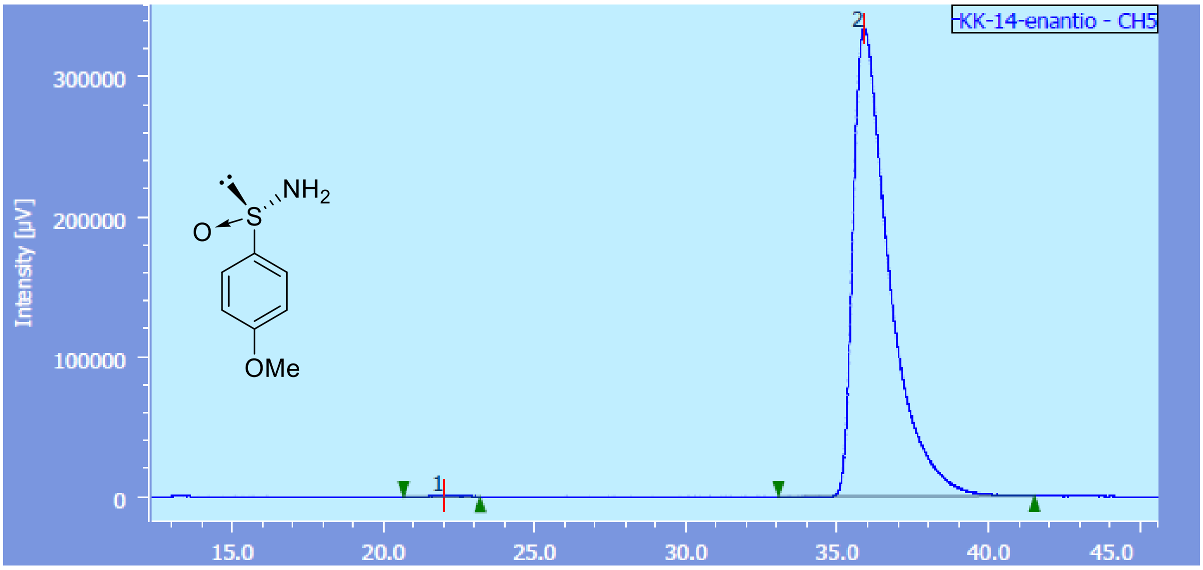

#	Peak Name	CH	tR [min]	Area [µV·sec]	Height [µV]	Area%	Height%	Quantity	NTP	Resolution	Symmetry Factor	Warning
1	Unknown	5	22.020	52109	1102	0.187	0.329	N/A	5430	8.619	1.323	
2	Unknown	5	35.883	27786272	333769	99.813	99.671	N/A	4999	N/A	2.204	

3-Methoxybenzenesulfinamide

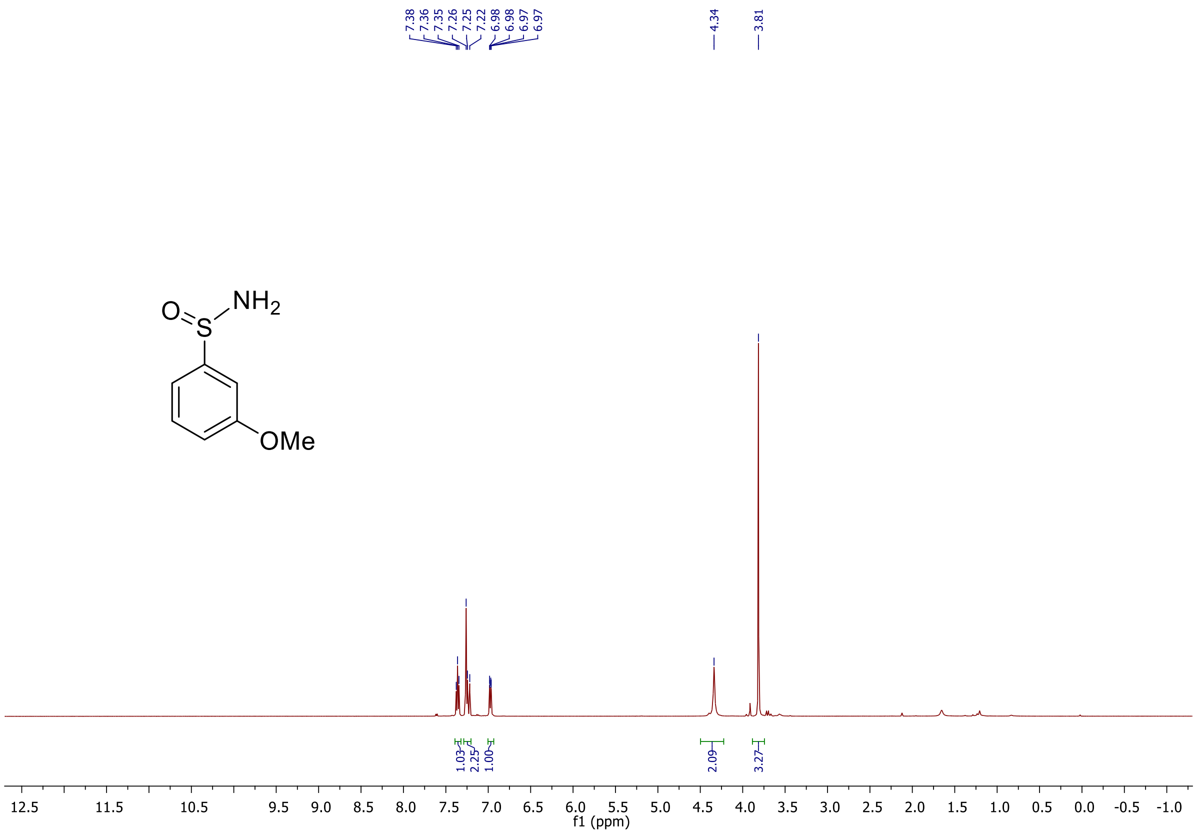

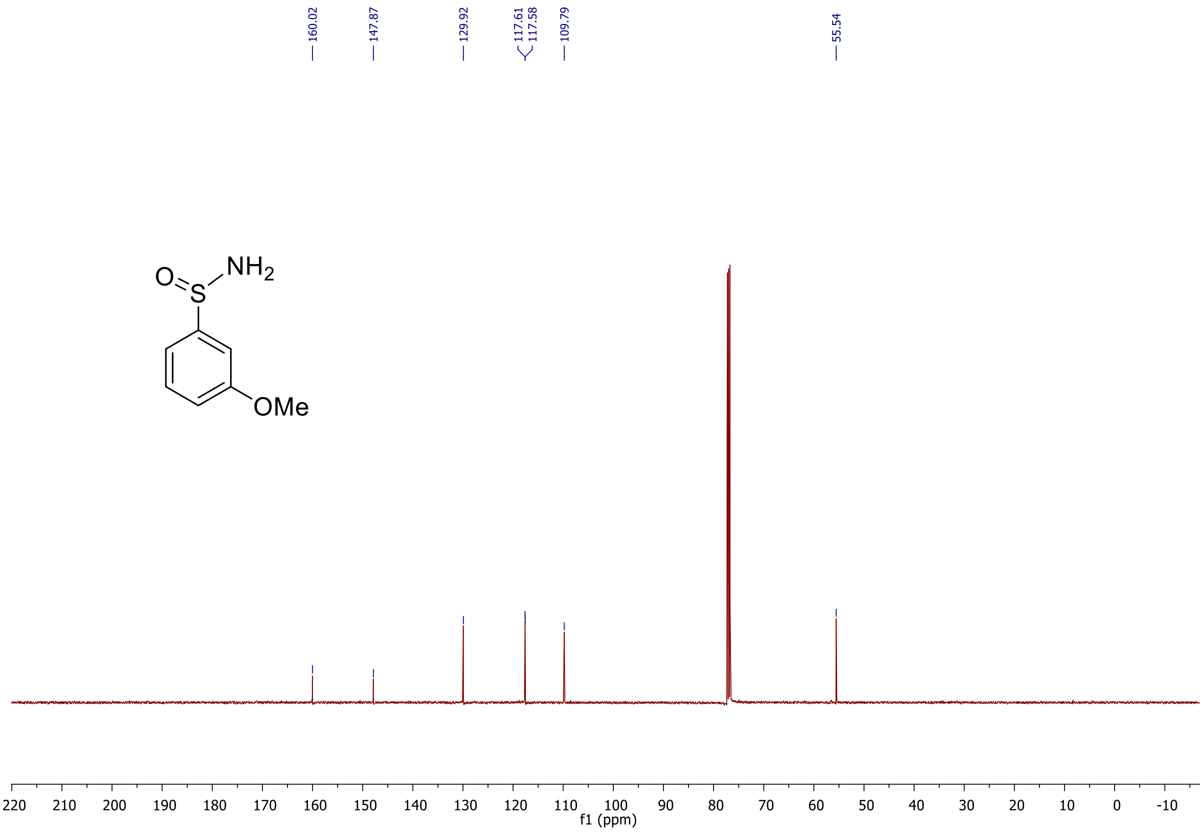

3-Bromobenzenesulfinamide

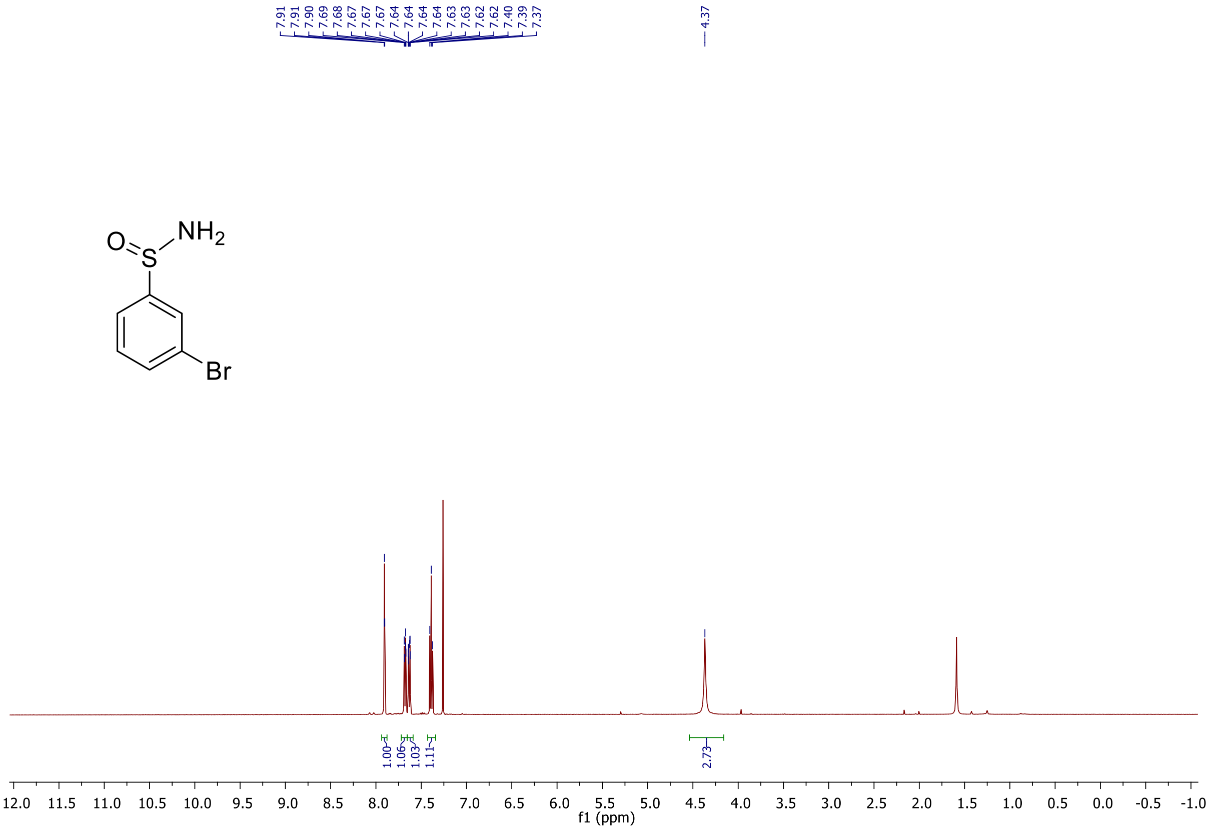

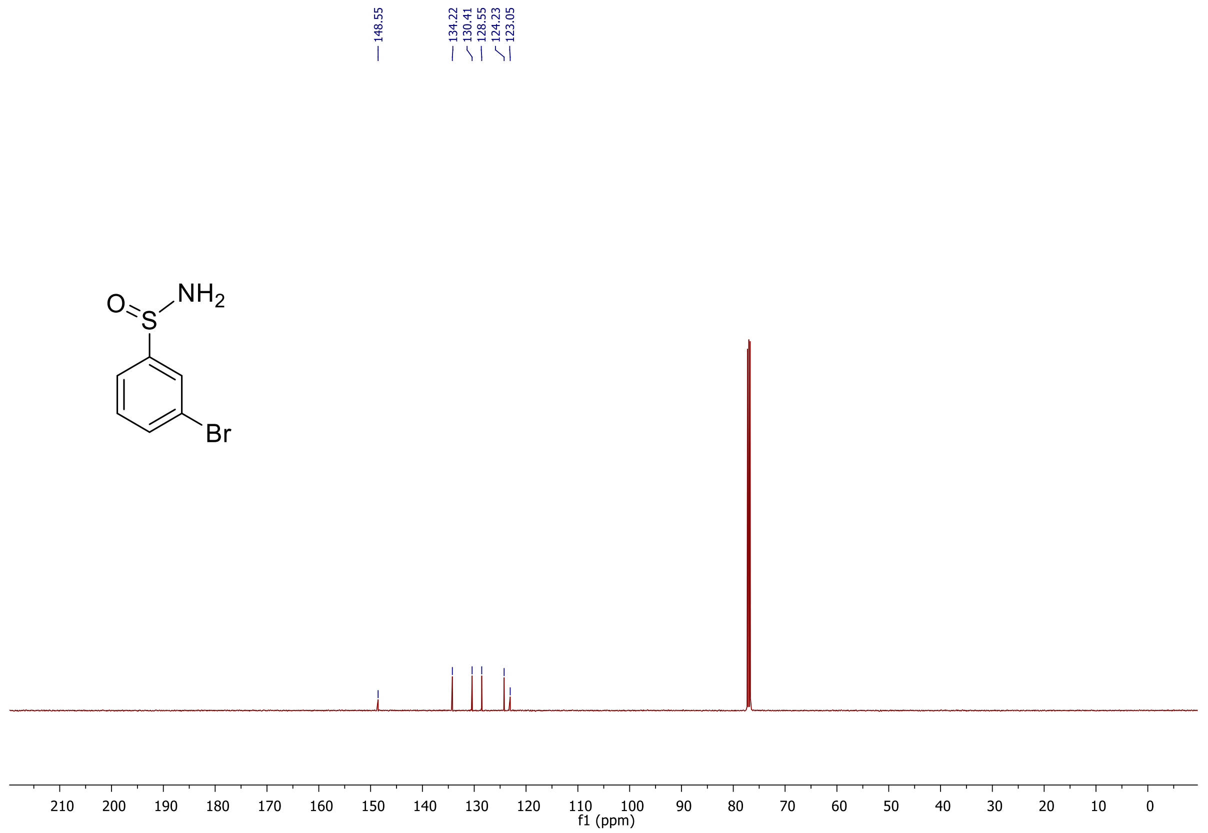

(*S*)-2-Methylbenzenesulfinamide

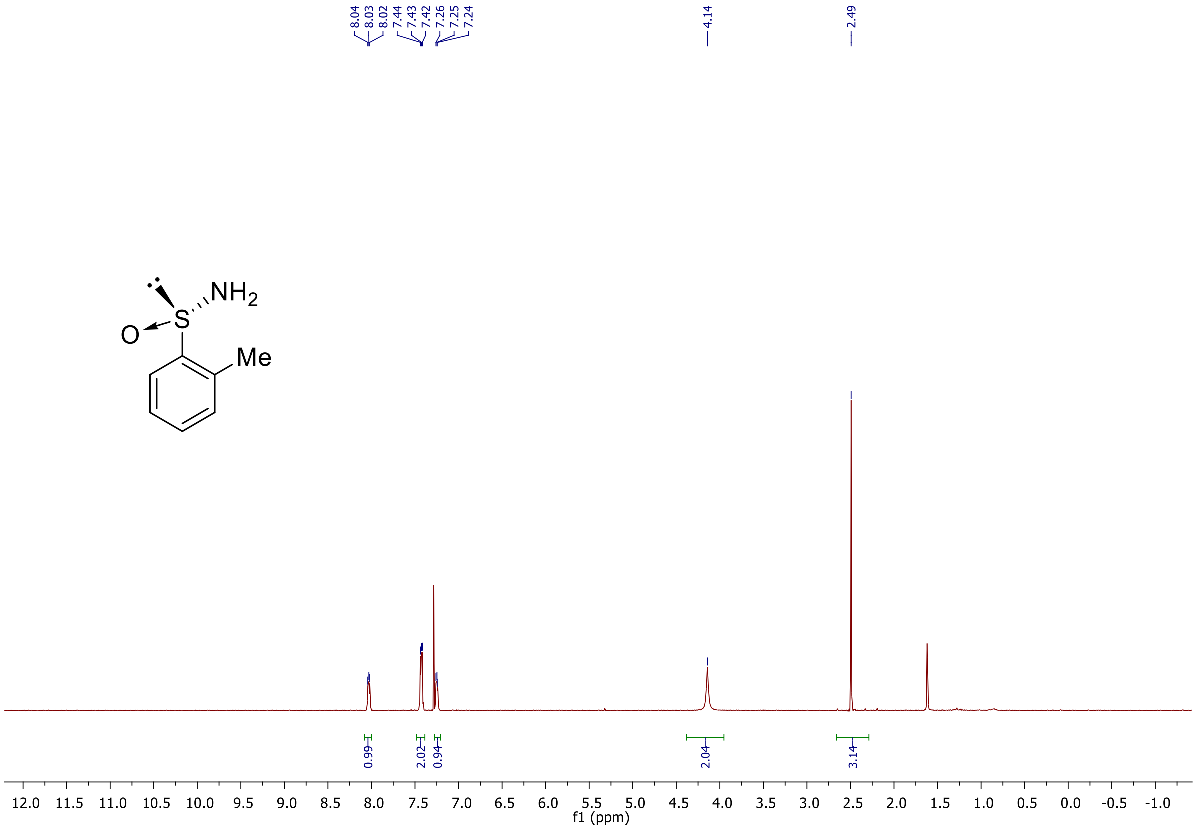

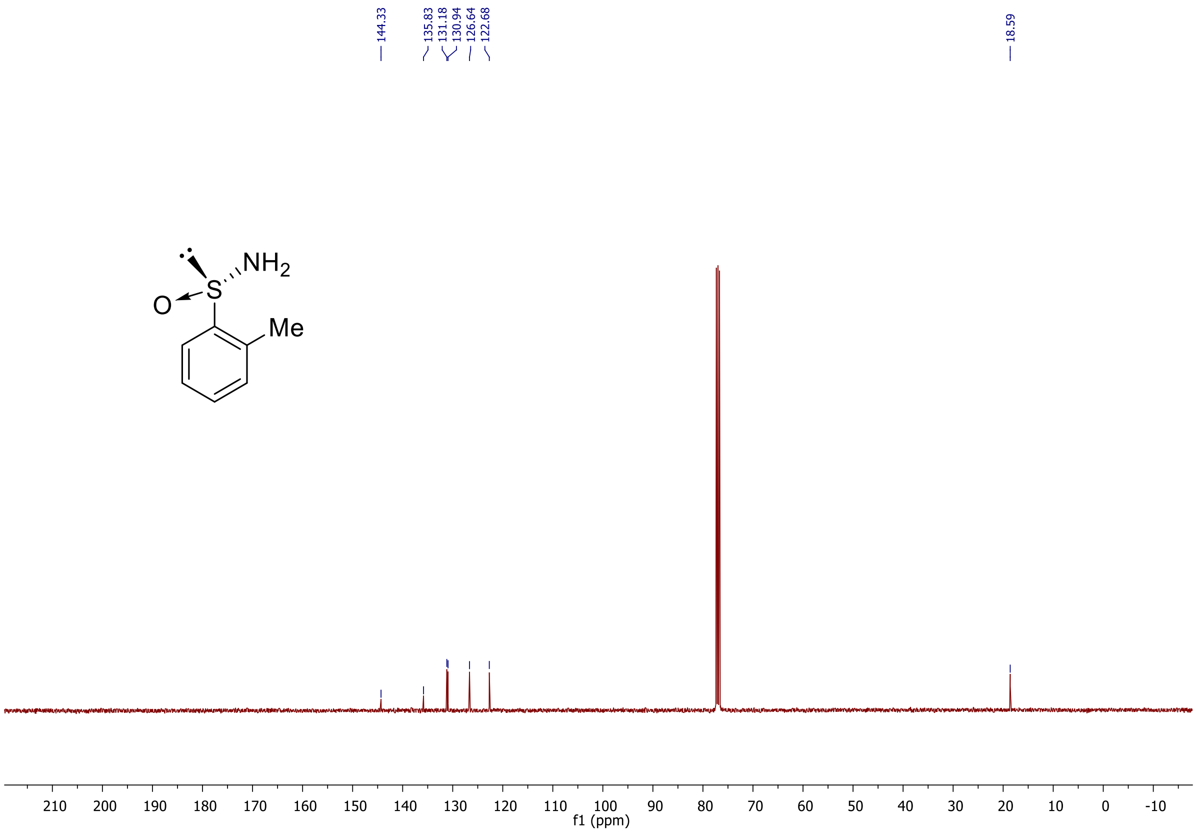

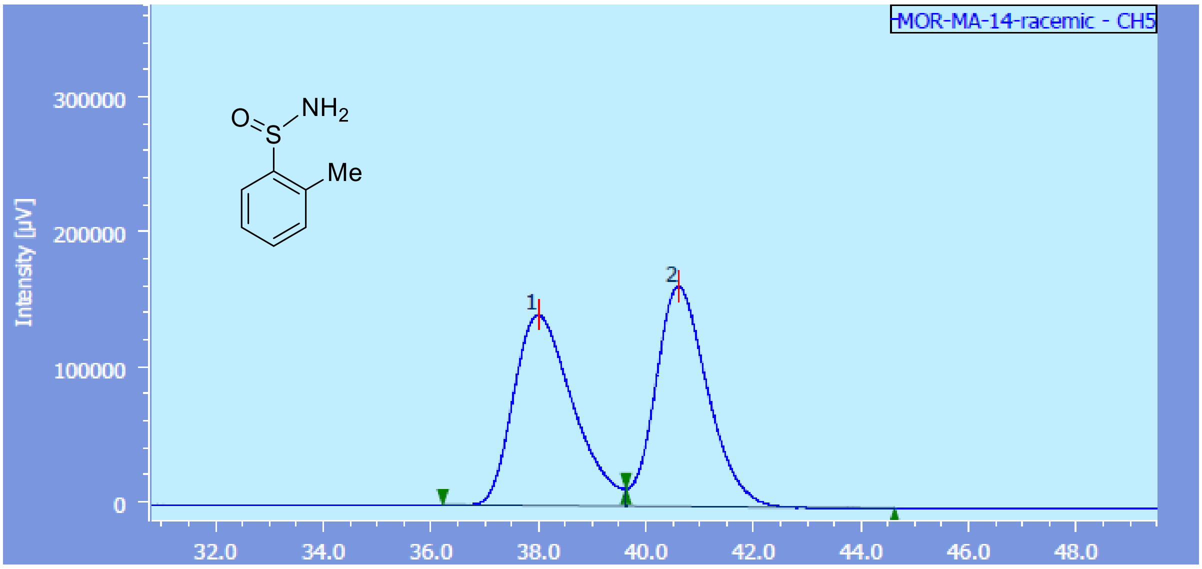

#	Peak Name	CH	tR [min]	Area [µV·sec]	Height [µV]	Area%	Height%	Quantity	NTP	Resolution	Symmetry Factor	Warning
1	Unknown	5	37.993	10855559	141338	49.777	46.391	N/A	5633	1.388	N/A	
2	Unknown	5	40.603	10952988	163332	50.223	53.609	N/A	8661	N/A	N/A	

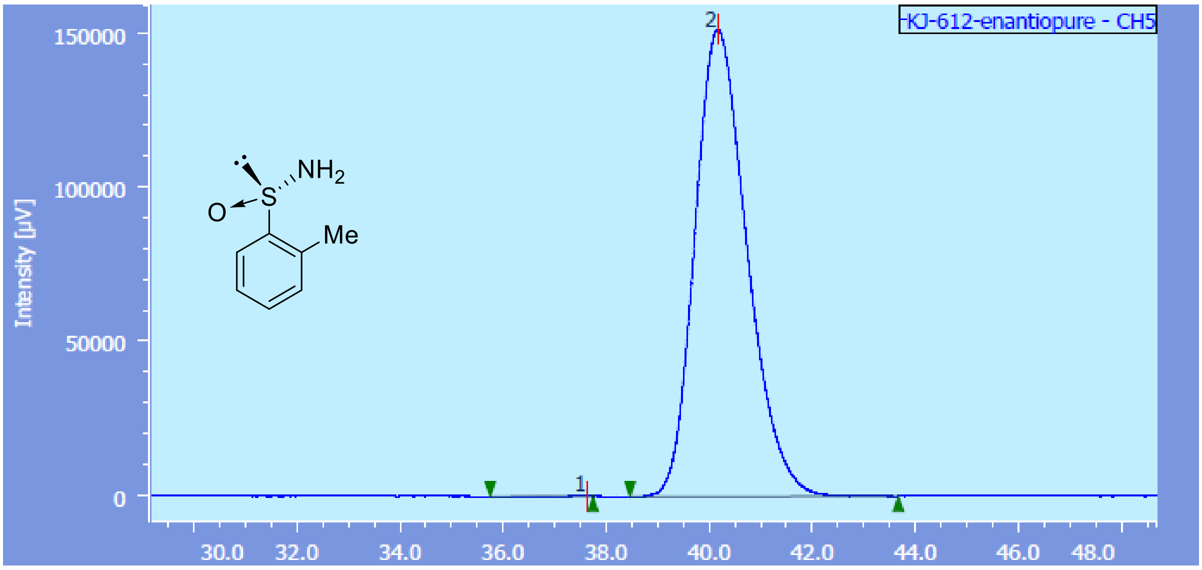

#	Peak Name	CH	tR [min]	Area [µV·sec]	Height [µV]	Area%	Height%	Quantity	NTP	Resolution	Symmetry Factor	Warning
1	Unknown	5	37.627	344	34	0.003	0.023	N/A	2037	0.983	0.532	
2	Unknown	5	40.163	10687027	151716	99.997	99.977	N/A	7601	N/A	1.251	

(*S*)-2-Bromobenzenesulfinamide

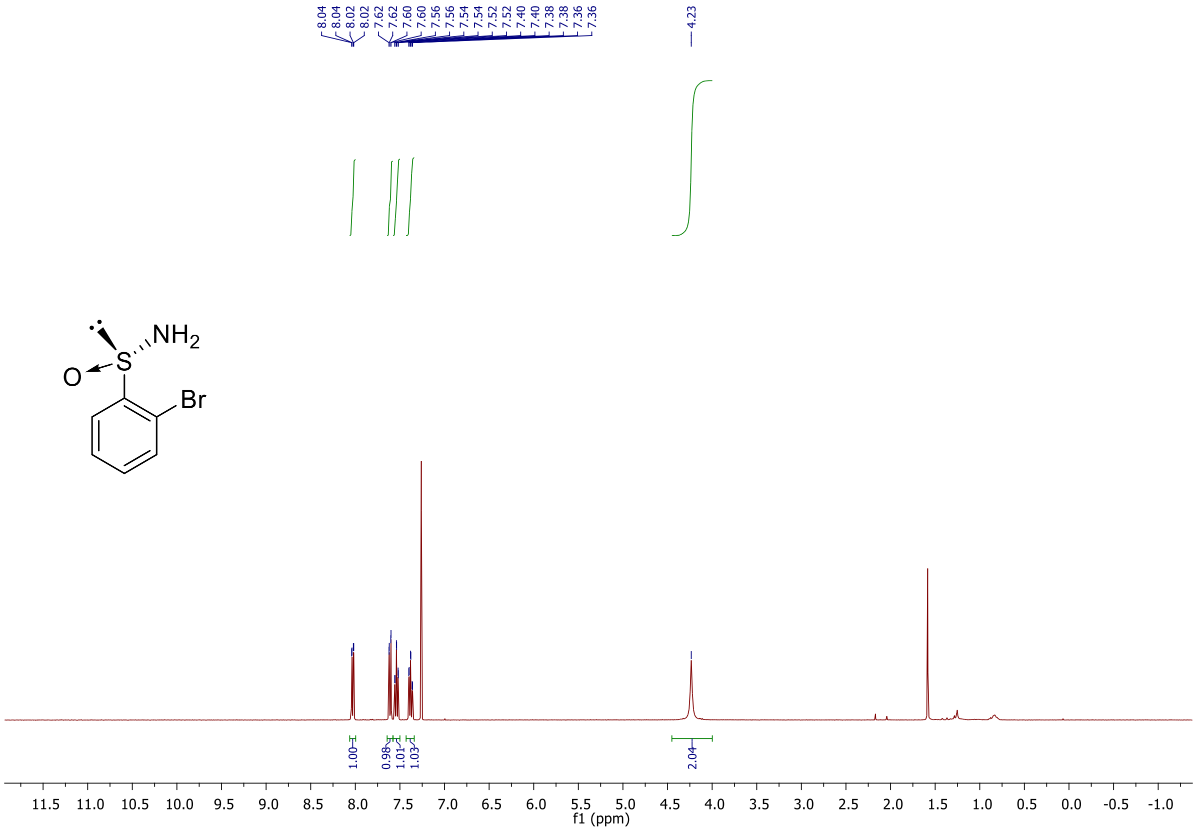

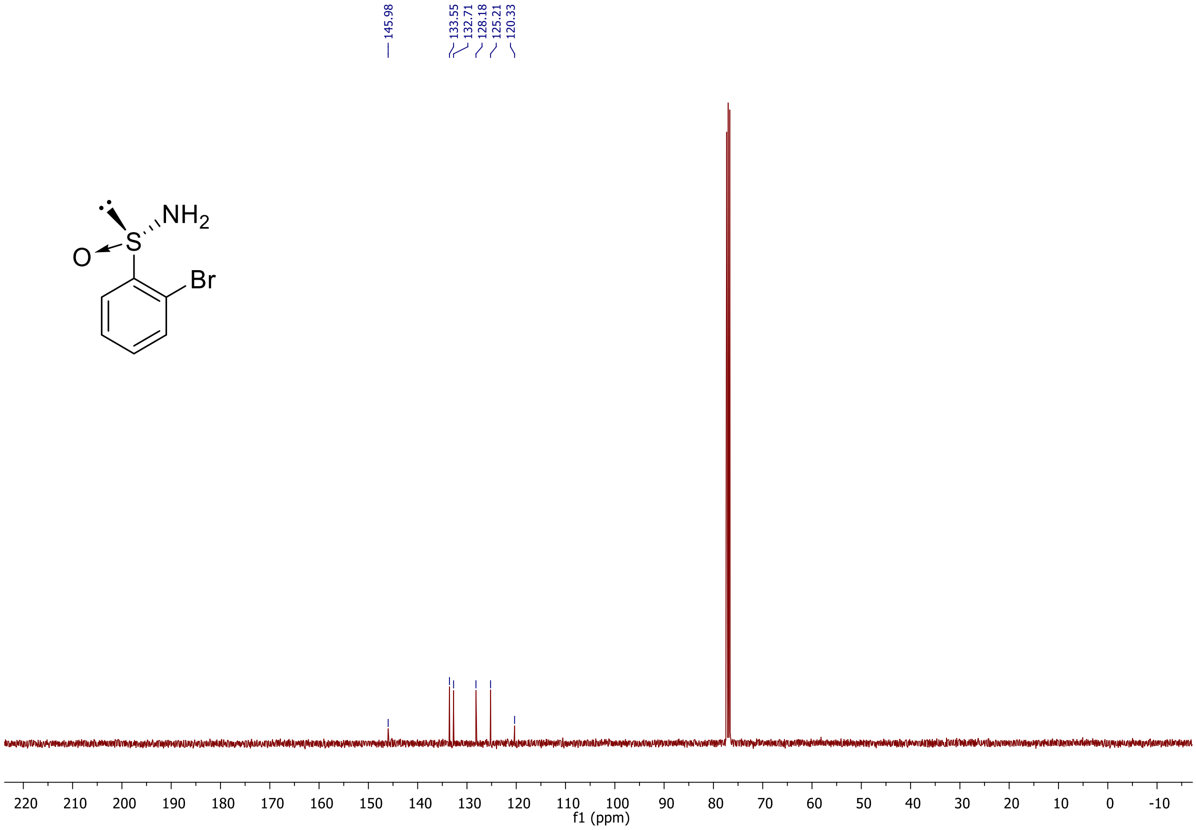

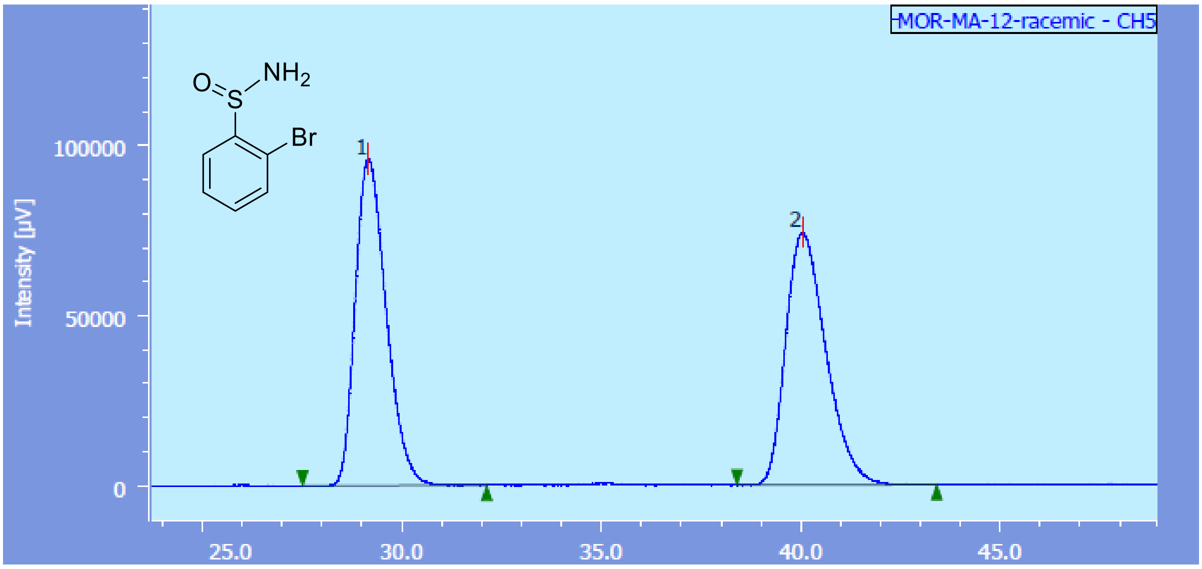

#	Peak Name	CH	tR [min]	Area [µV·sec]	Height [µV]	Area%	Height%	Quantity	NTP	Resolution	Symmetry Factor	Warning
1	Unknown	5	29.167	5173734	95932	49.880	56.412	N/A	6739	6.681	1.319	
2	Unknown	5	40.040	5198730	74123	50.120	43.588	N/A	7558	N/A	1.350	

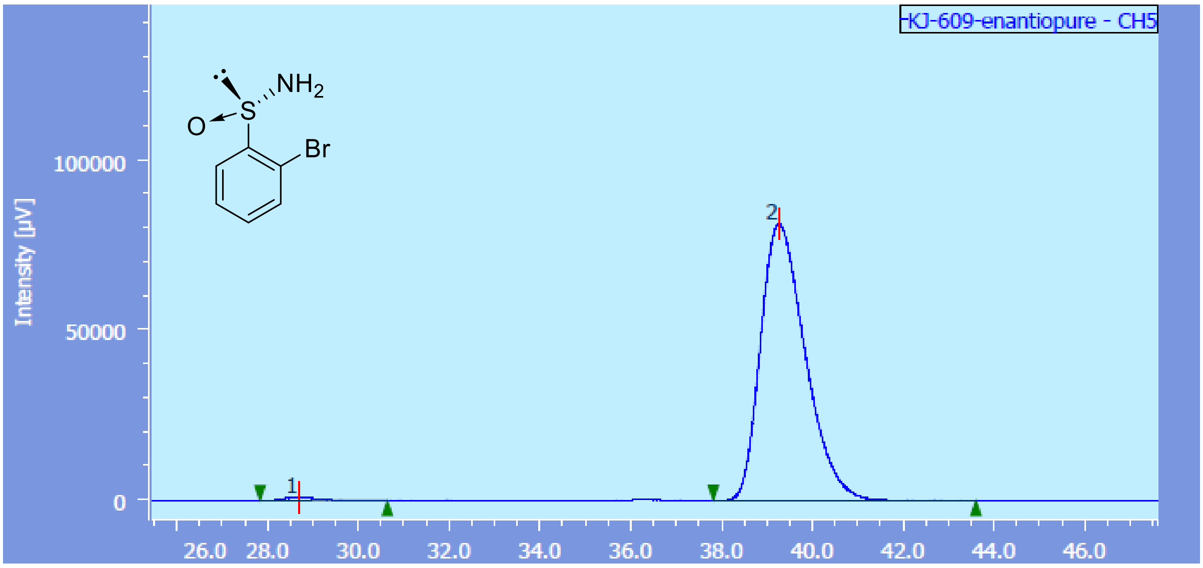

#	Peak Name	CH	tR [min]	Area [µV·sec]	Height [µV]	Area%	Height%	Quantity	NTP	Resolution	Symmetry Factor	Warning
1	Unknown	5	28.683	50280	973	0.894	1.182	N/A	7232	6.736	1.215	
2	Unknown	5	39.260	5574268	81333	99.106	98.818	N/A	7617	N/A	1.375	

(*S*)-5-Chlorothiophene-2-sulfinamide

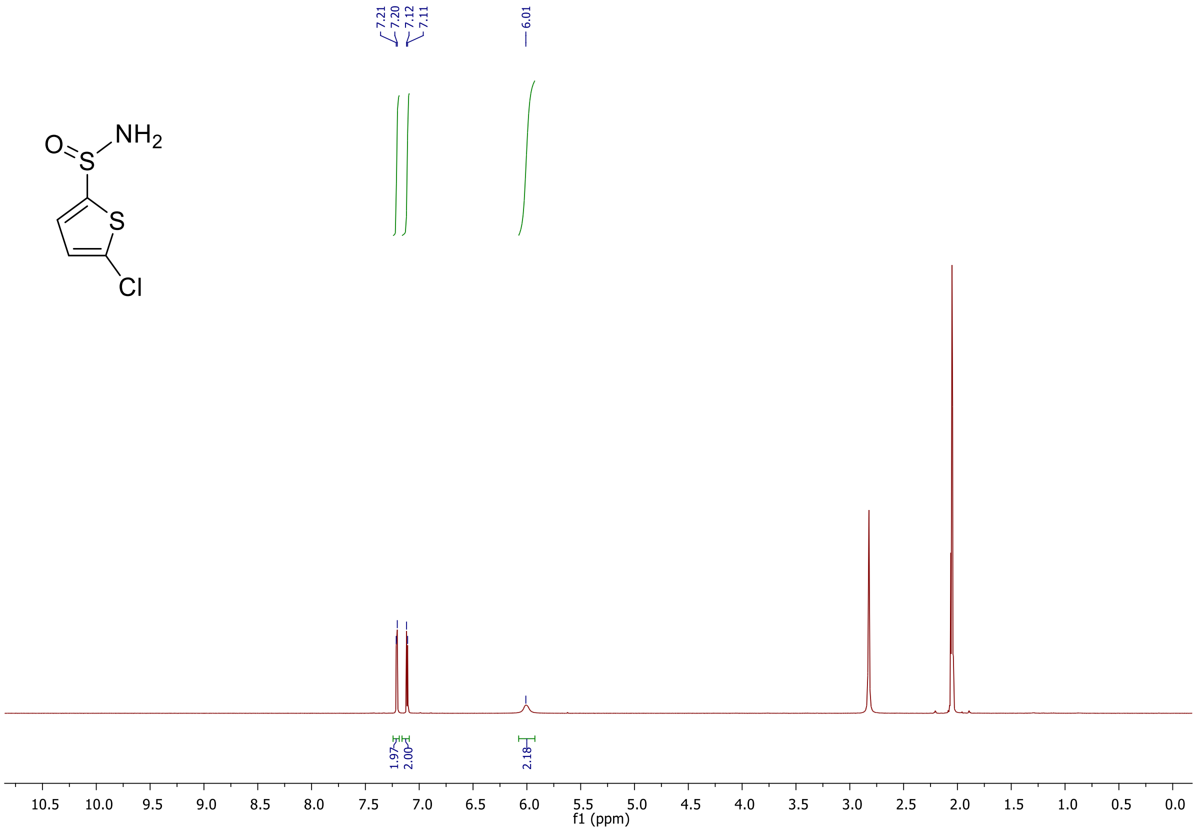

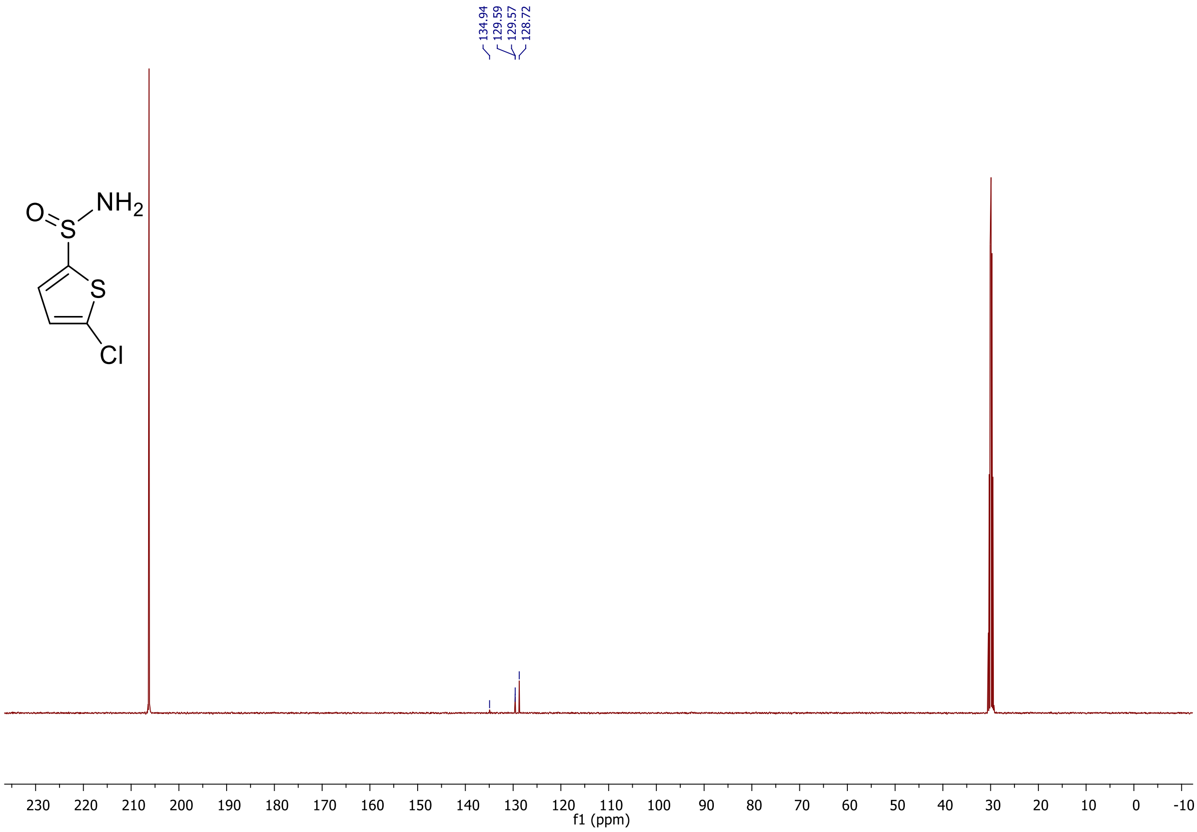

(*S*)-*N*-(*p*-Tolylsulfinyl)butyramide (1a)

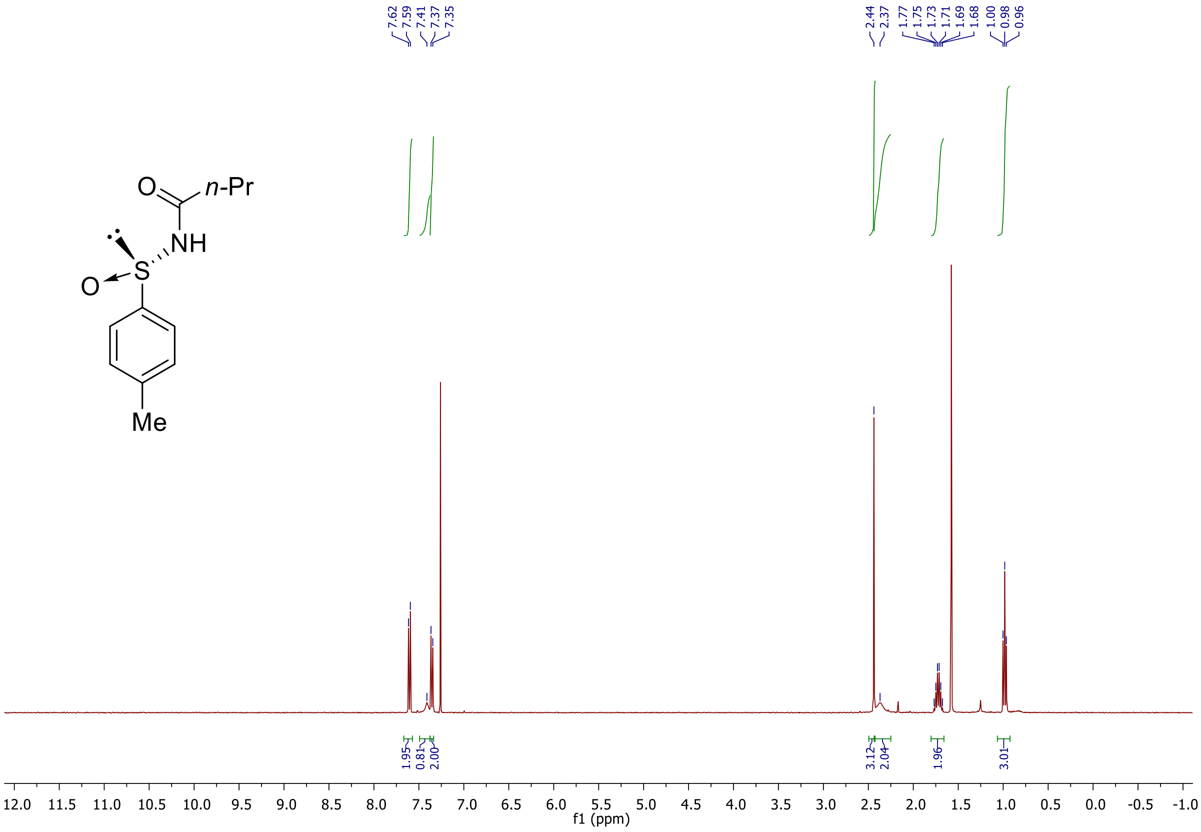

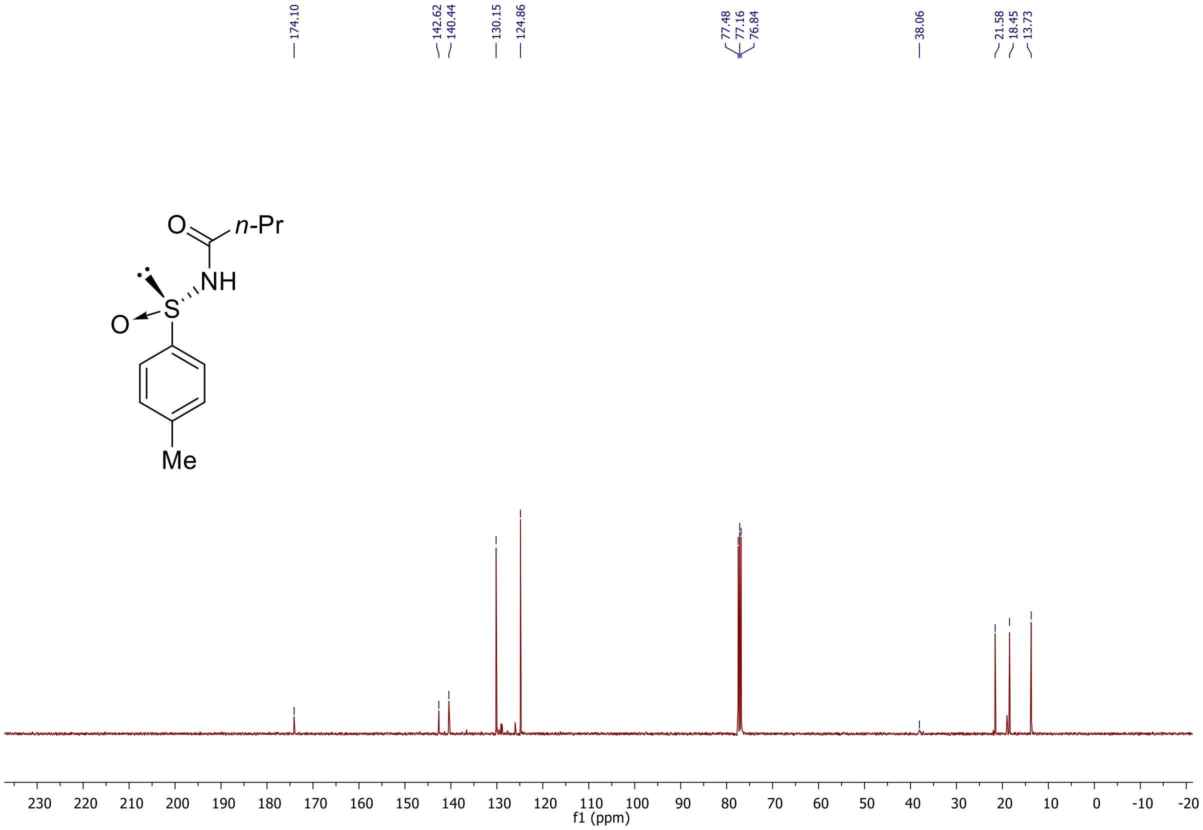

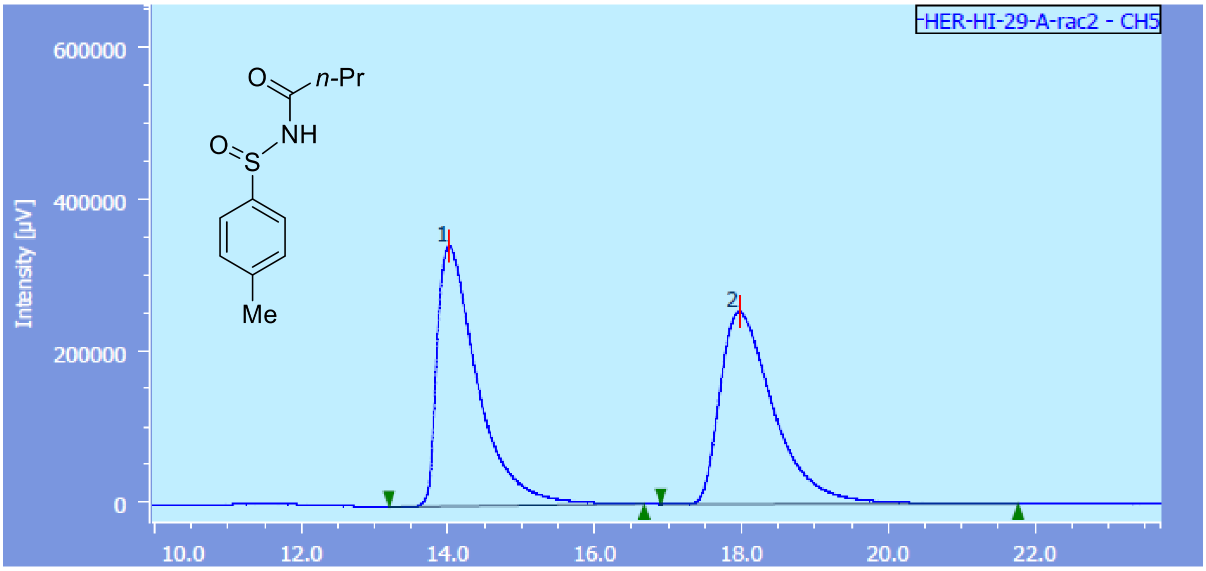

#	Peak Name	CH	tR [min]	Area [μV·sec]	Height [μV]	Area%	Height%	Quantity	NTP	Resolution	Symmetry Factor	Warning
1	Unknown	5	14.013	13156022	342830	50.356	57.487	N/A	3560	3.561	2.447	
2	Unknown	5	17.967	12969964	253536	49.644	42.513	N/A	3121	N/A	1.780	

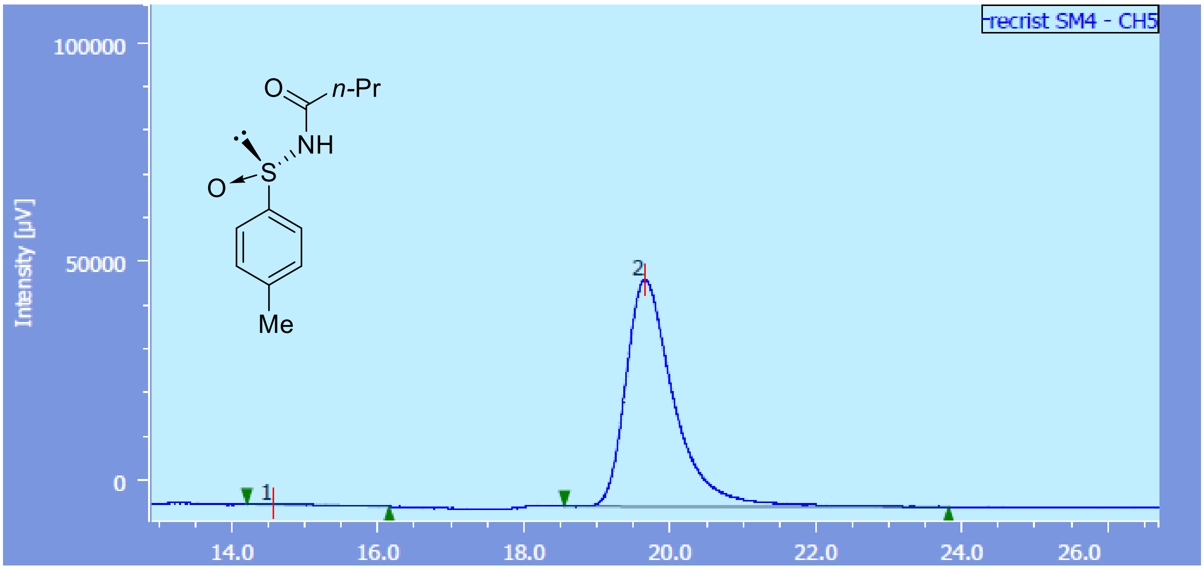

#	Peak Name	CH	tR [min]	Area [μV·sec]	Height [μV]	Area%	Height%	Quantity	NTP	Resolution	Symmetry Factor	Warning
1	Unknown	5	14.560	9835	219	0.413	0.420	N/A	2596	4.530	3.036	
2	Unknown	5	19.657	2373144	51859	99.587	99.580	N/A	4988	N/A	1.534	

(*R*)-*N*-(*p*-Tolylsulfinyl)butyramide (1a')

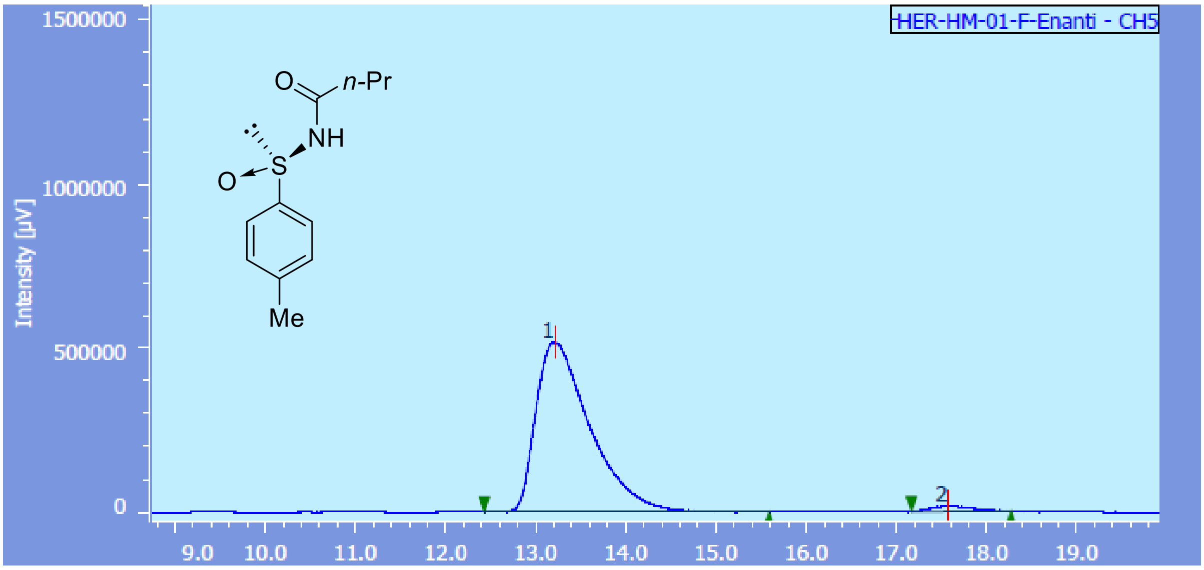

#	Peak Name	CH	tR [min]	Area [µV·sec]	Height [µV]	Area%	Height%	Quantity	NTP	Resolution	Symmetry Factor	Warning
1	Unknown	5	13.207	21612927	517133	97.670	97.085	N/A	2460	4.435	1.837	
2	Unknown	5	17.573	515556	15528	2.330	2.915	N/A	5978	N/A	1.348	

(*S*)-2-(4-Methoxyphenyl)-*N*-(*p*-tolylsulfinyl)acetamide (1b)

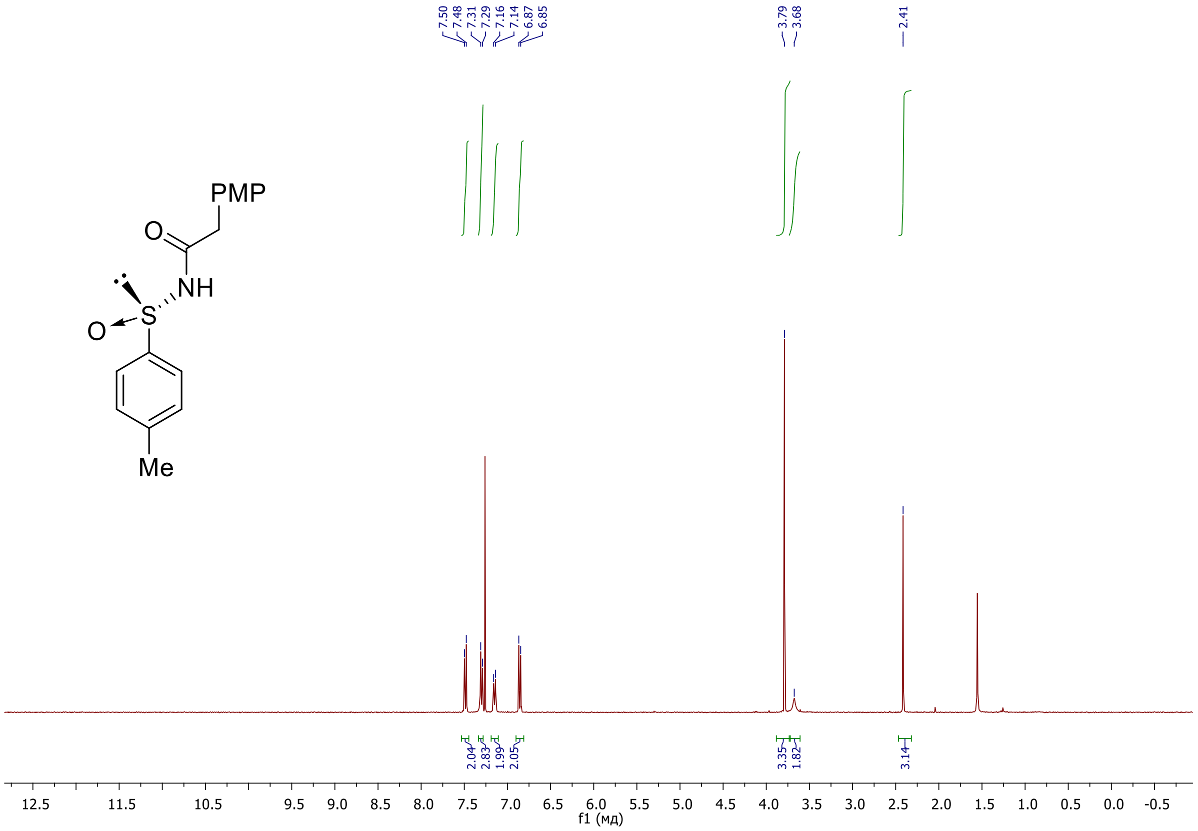

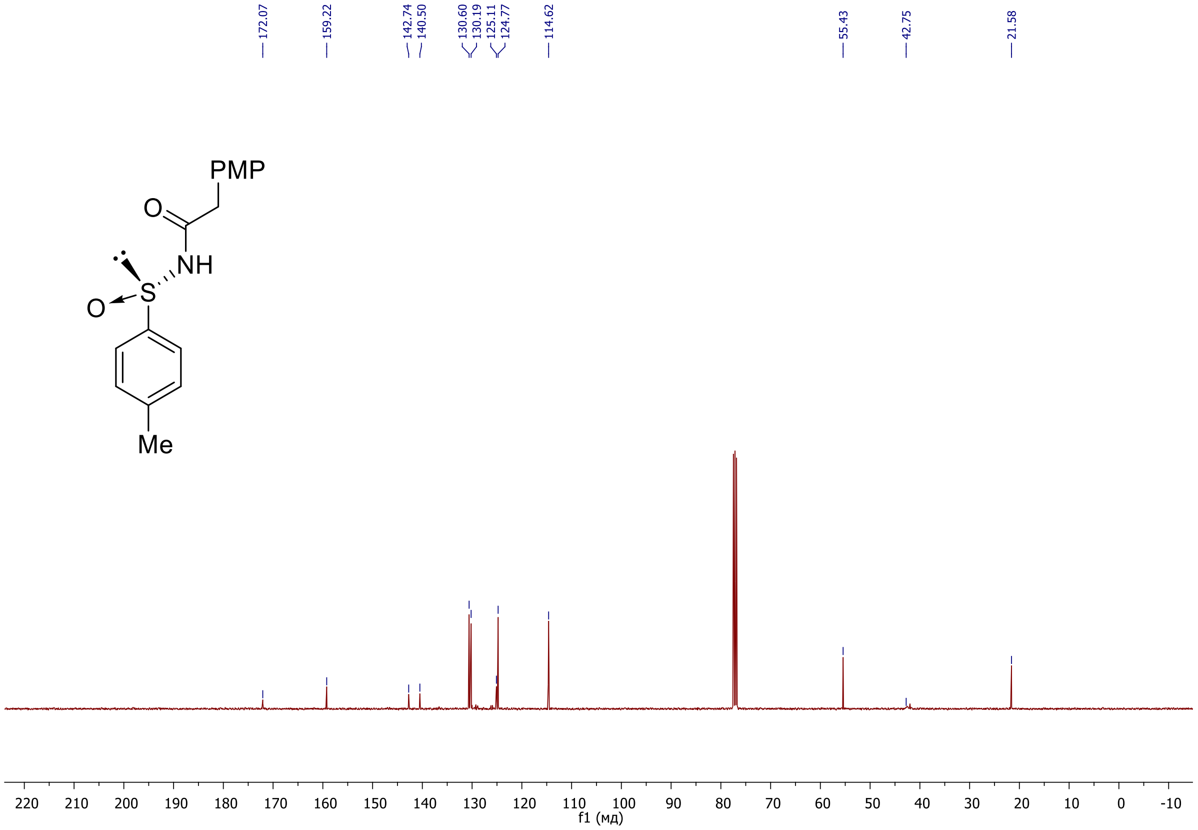

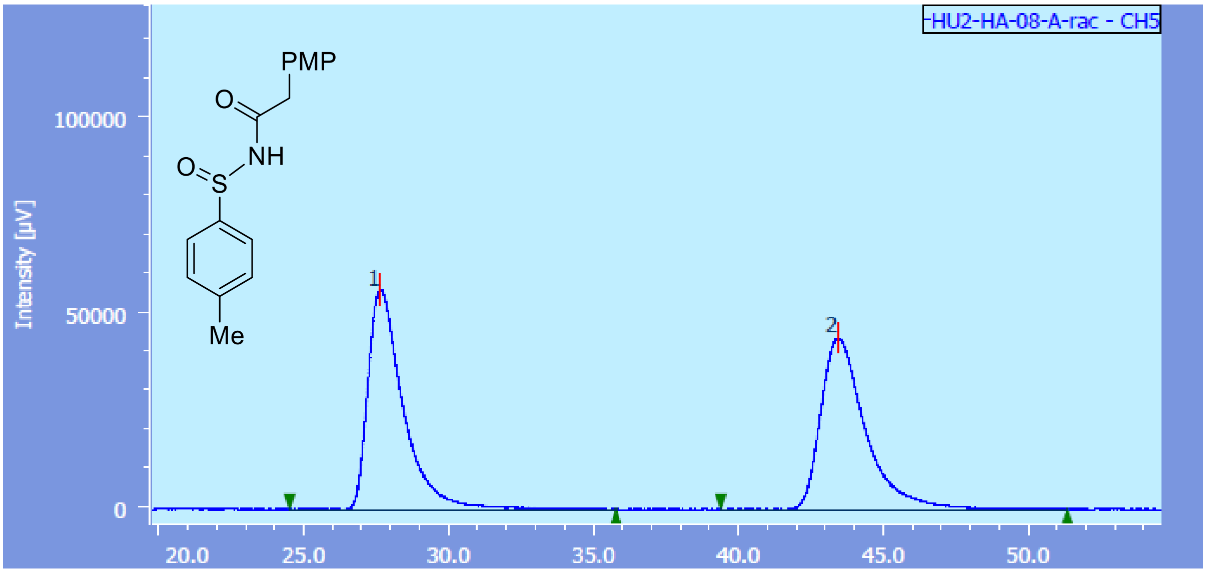

#	Peak Name	CH	tR [min]	Area [µV·sec]	Height [µV]	Area%	Height%	Quantity	NTP	Resolution	Symmetry Factor	Warning
1	Unknown	5	27.657	4802962	56457	50.435	56.160	N/A	2894	6.783	1.814	
2	Unknown	5	43.447	4720151	44072	49.565	43.840	N/A	4427	N/A	1.648	

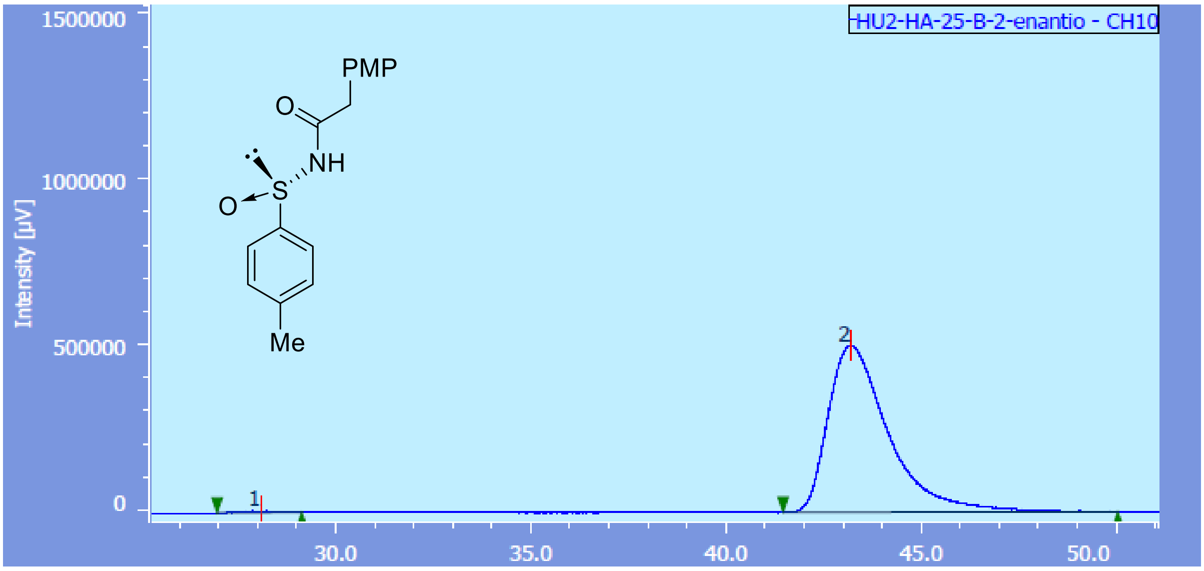

#	Peak Name	CH	tR [min]	Area [µV·sec]	Height [µV]	Area%	Height%	Quantity	NTP	Resolution	Symmetry Factor	Warning
1	Unknown	10	28.087	293726	4498	0.539	0.887	N/A	3822	6.770	0.987	
2	Unknown	10	43.193	54177830	502891	99.461	99.113	N/A	4226	N/A	1.753	

[(*R*)-2-Phenyl-*N*-(*S*)-*p*-tolylsulfinyl]butanamide (1c)

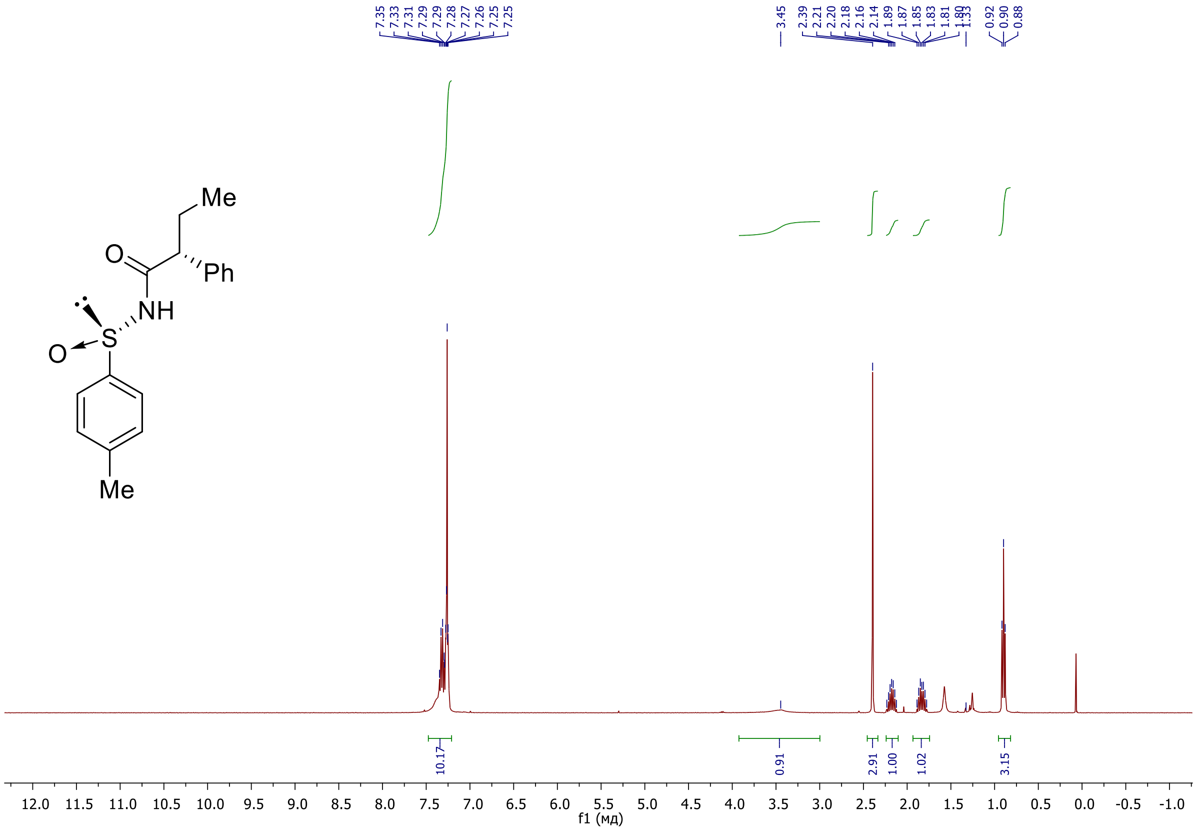

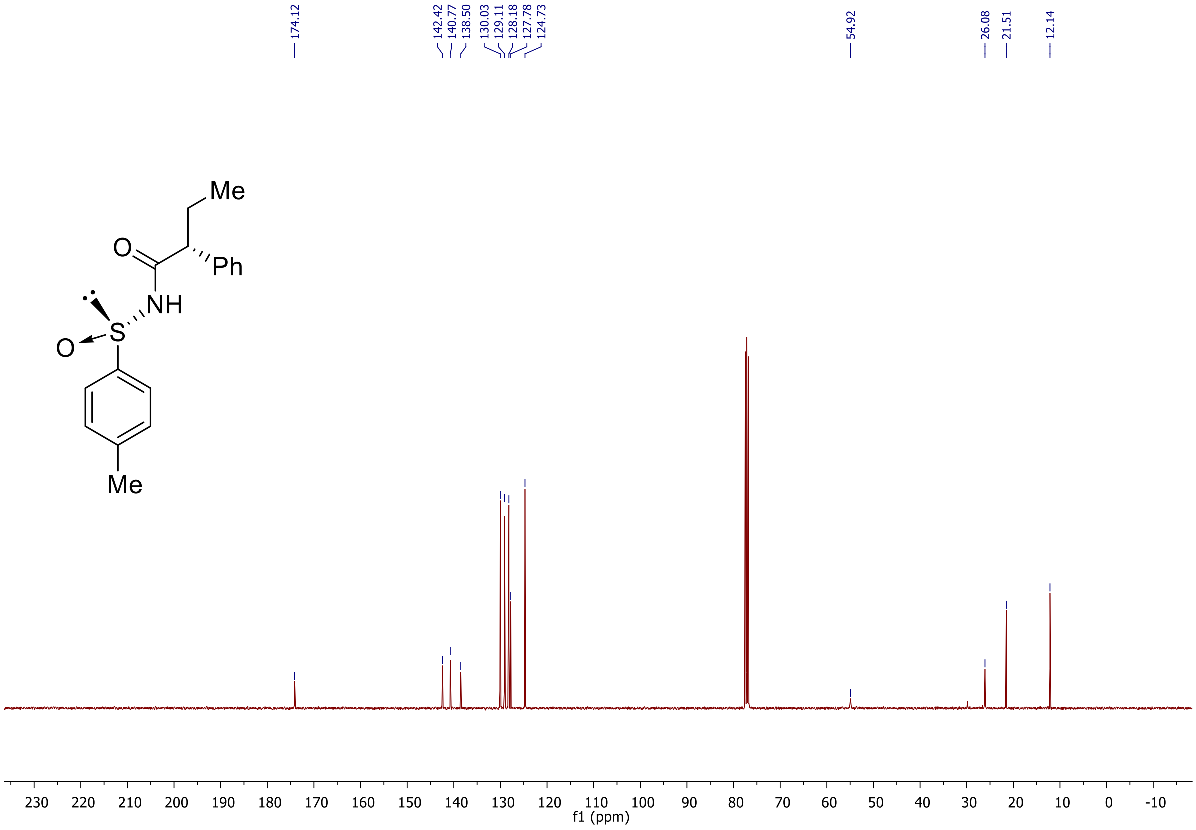

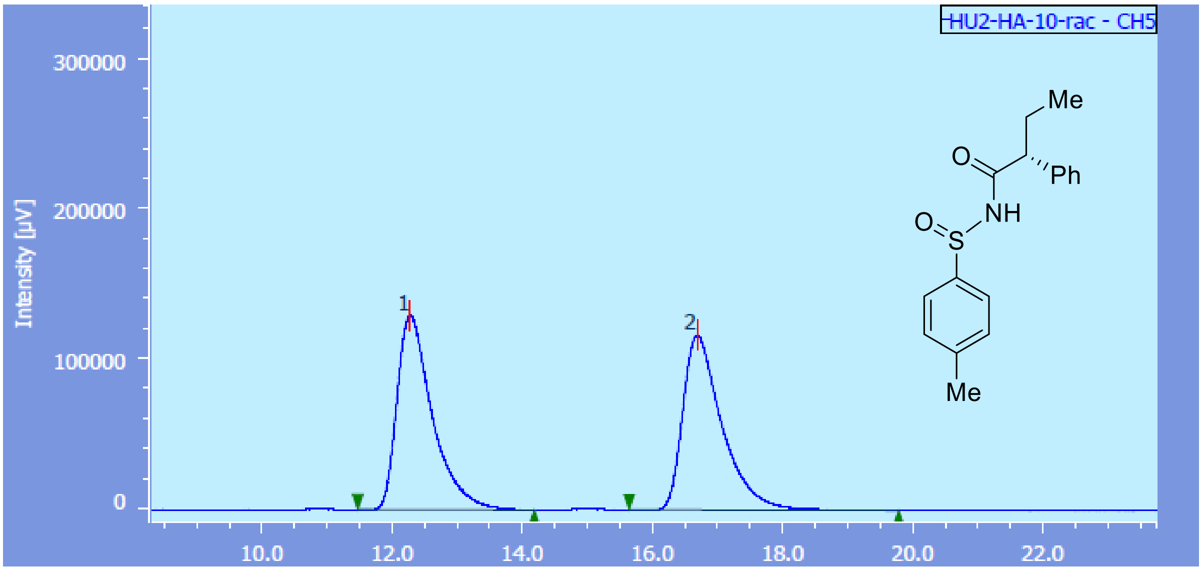

#	Peak Name	CH	tR [min]	Area [μV·sec]	Height [μV]	Area%	Height%	Quantity	NTP	Resolution	Symmetry Factor	Warning
1	Unknown	5	12.277	4903134	129486	50.094	52.588	N/A	2746	4.472	1.693	
2	Unknown	5	16.683	4884766	116740	49.906	47.412	N/A	4125	N/A	1.678	

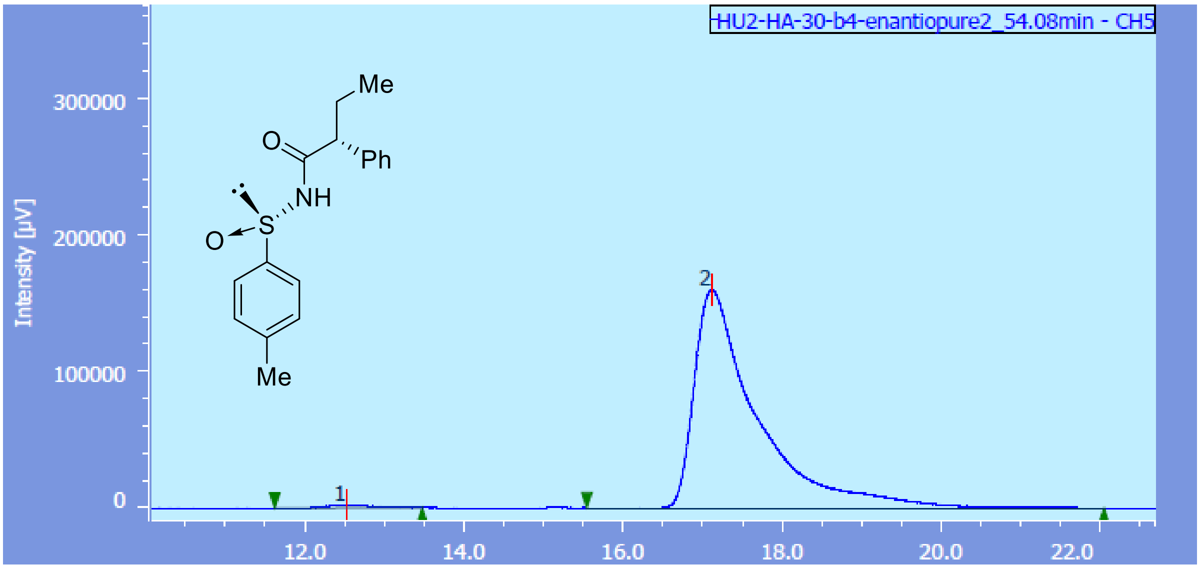

#	Peak Name	CH	tR [min]	Area [μV·sec]	Height [μV]	Area%	Height%	Quantity	NTP	Resolution	Symmetry Factor	Warning
1	Unknown	5	12.530	90369	2192	1.026	1.353	N/A	2075	4.053	1.394	
2	Unknown	5	17.117	8715269	159798	98.974	98.647	N/A	3428	N/A	2.910	

(*S*)-*N*-(*p*-Tolylsulfinyl)cyclohexanecarboxamide (1d)

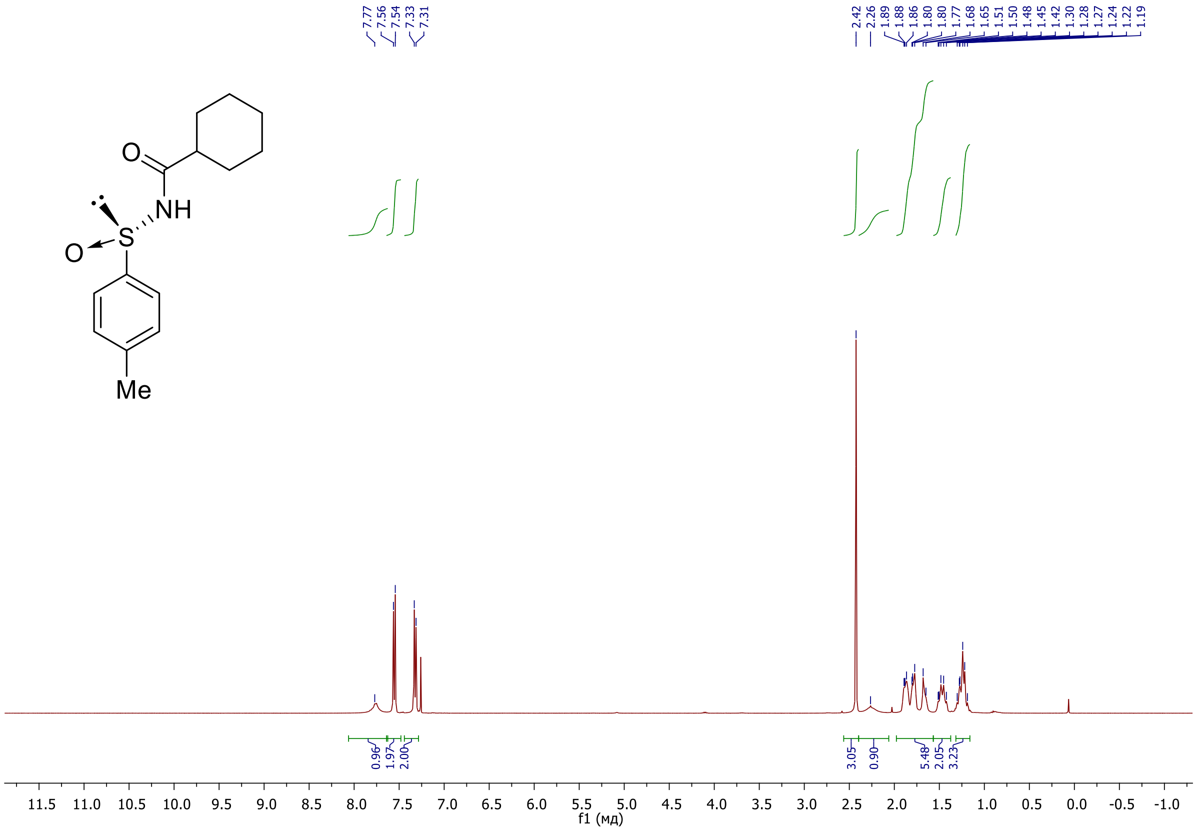

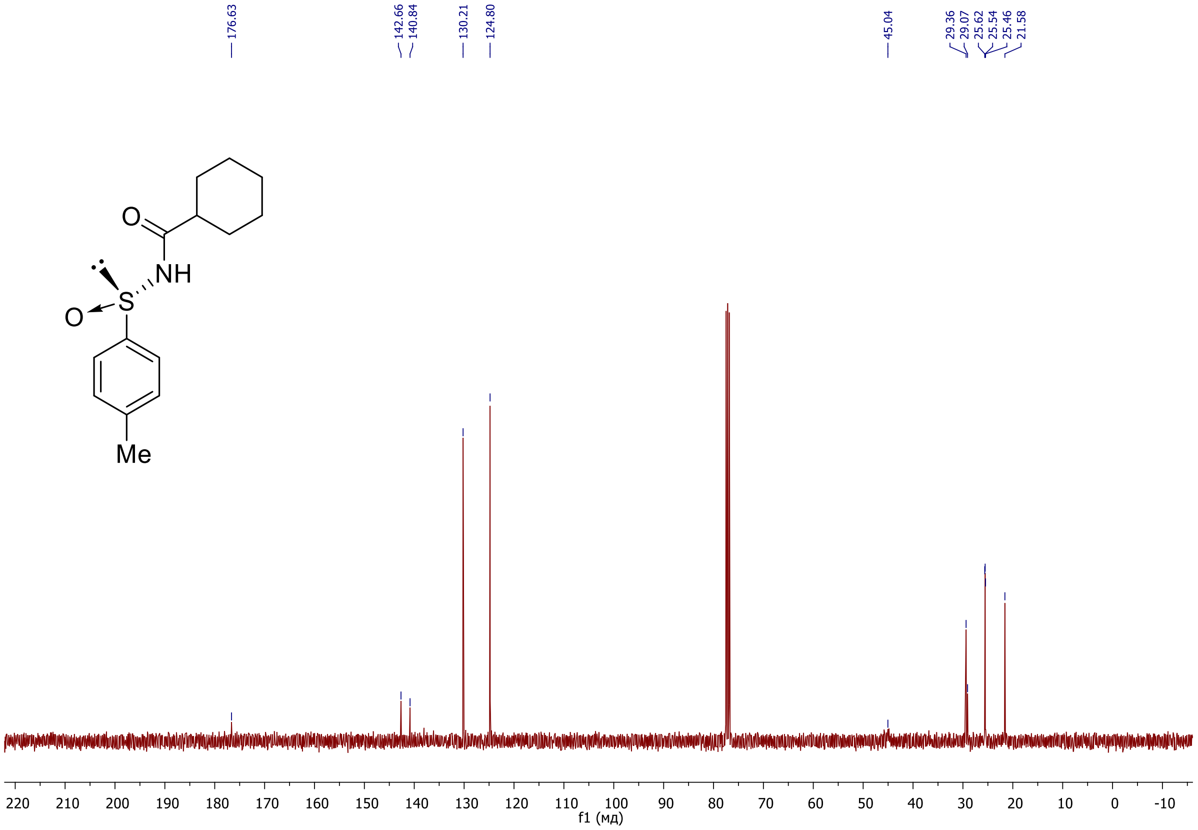

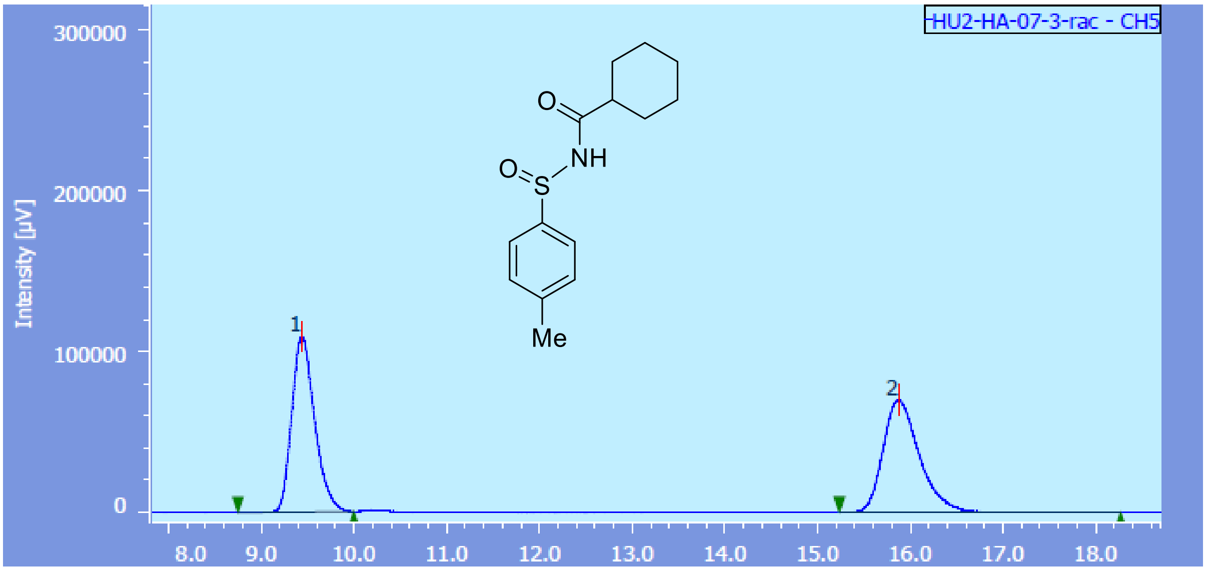

#	Peak Name	CH	tR [min]	Area [µV·sec]	Height [µV]	Area%	Height%	Quantity	NTP	Resolution	Symmetry Factor	Warning
1	Unknown	5	9.437	1870832	108940	49.630	60.920	N/A	7184	11.350	1.278	
2	Unknown	5	15.870	1898757	69883	50.370	39.080	N/A	8433	N/A	1.356	

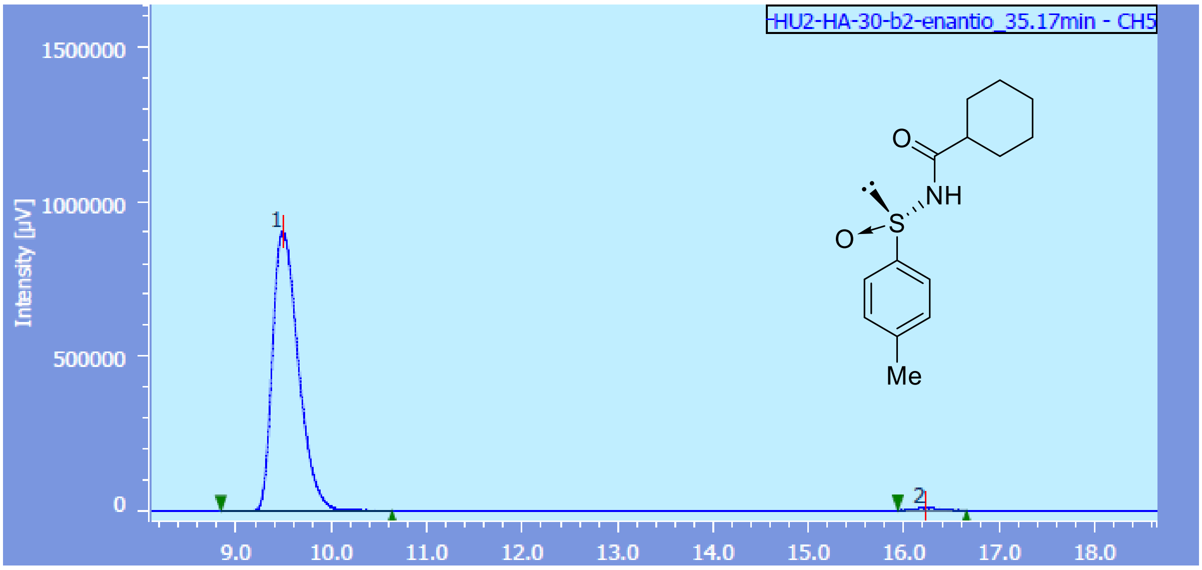

#	Peak Name	CH	tR [min]	Area [µV·sec]	Height [µV]	Area%	Height%	Quantity	NTP	Resolution	Symmetry Factor	Warning
1	Unknown	5	9.493	17044454	902356	98.927	99.093	N/A	5889	12.084	1.431	
2	Unknown	5	16.227	184785	8261	1.073	0.907	N/A	10867	N/A	1.209	

Ethyl (*S*)-4-oxo-4-[(*p*-tolylsulfinyl)amino]butanoate (1e)

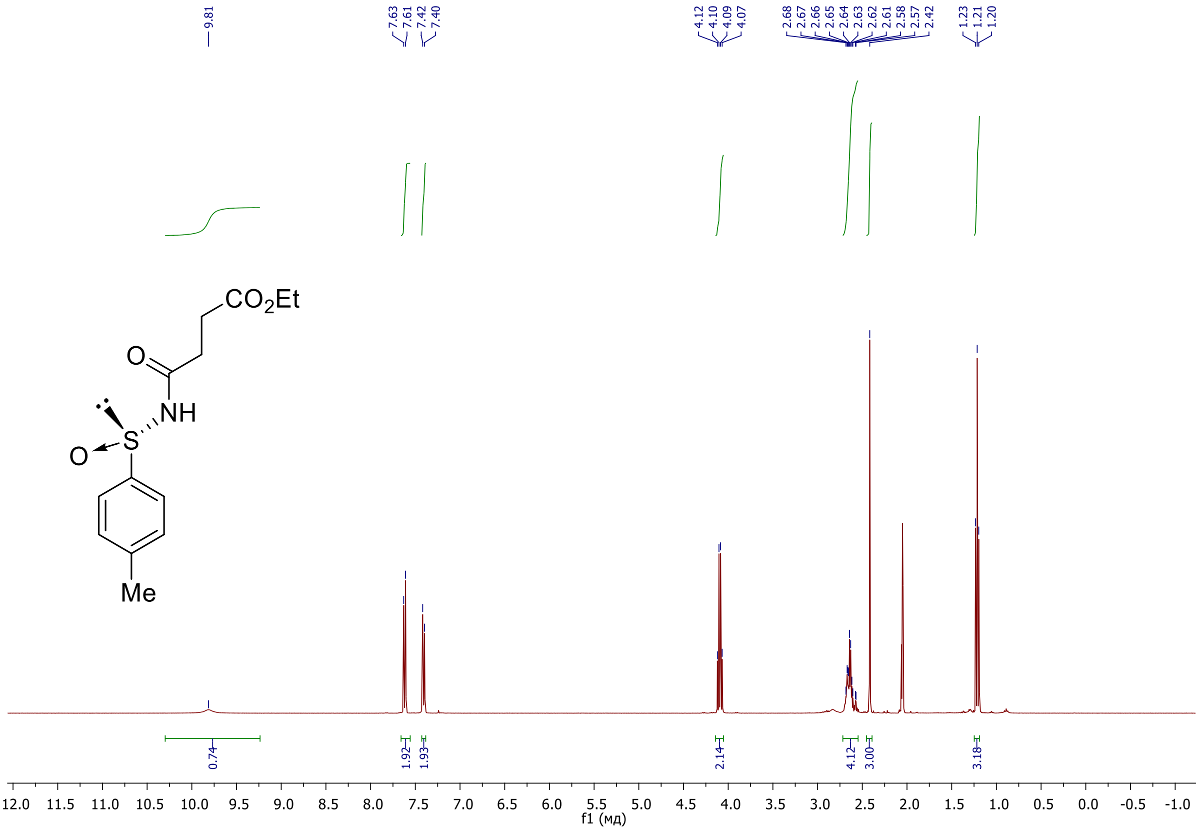

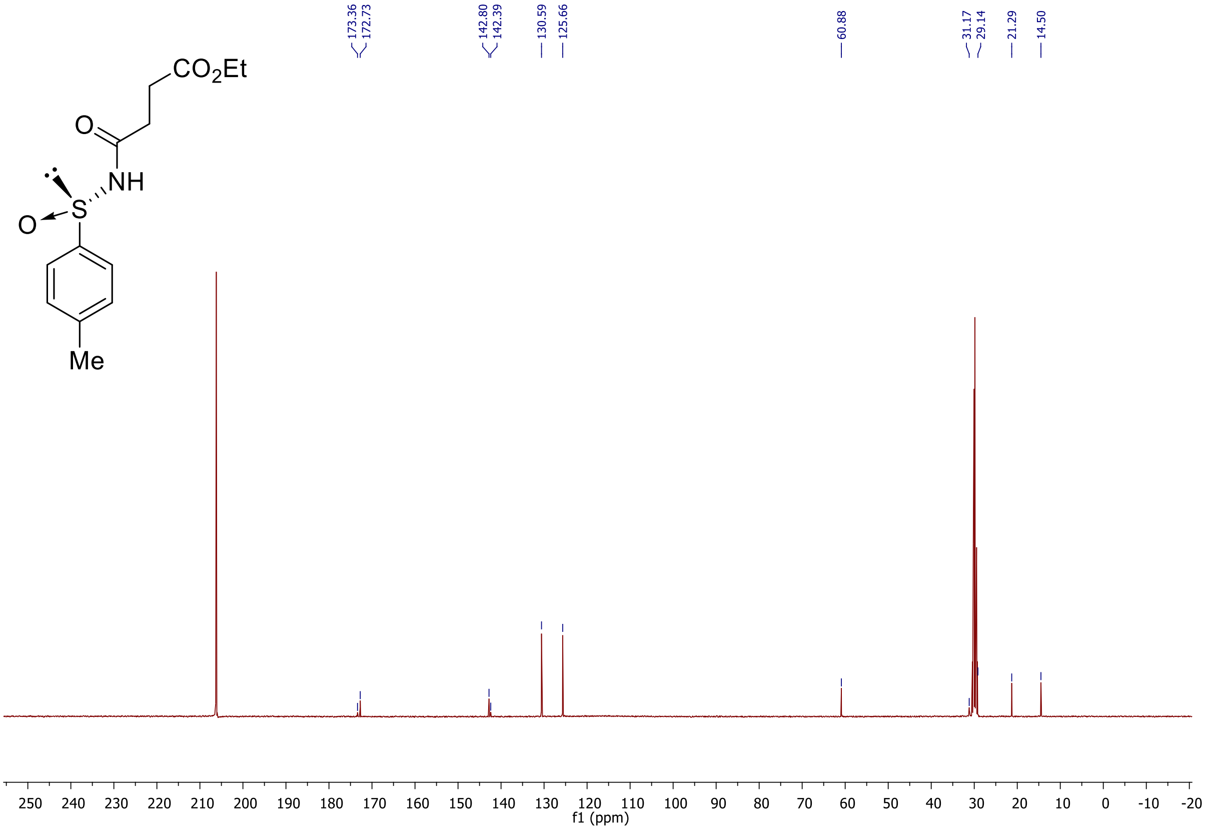

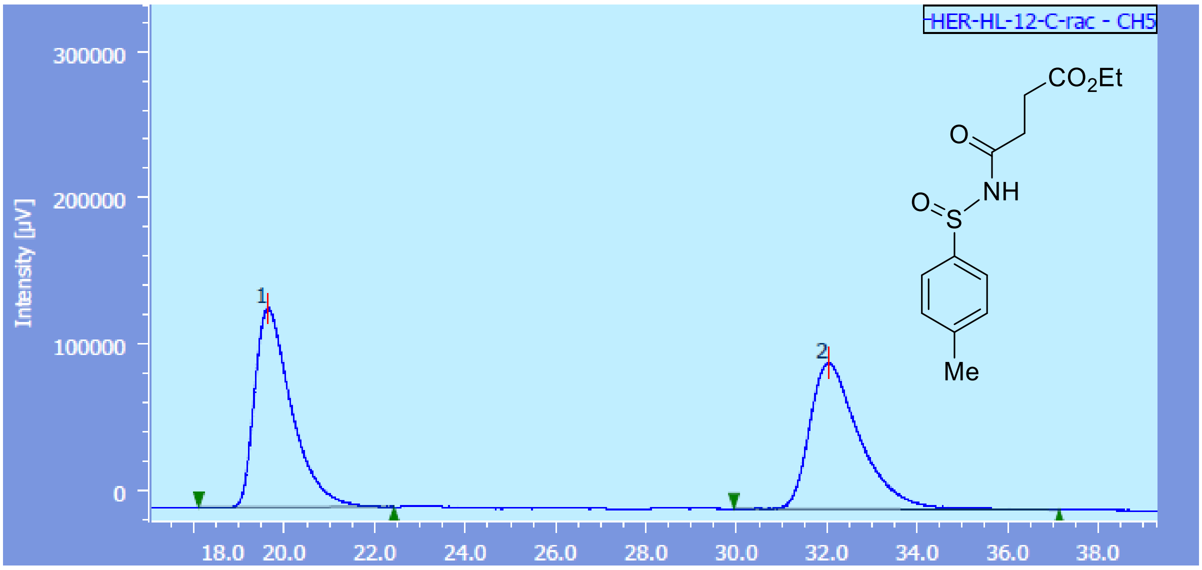

#	Peak Name	CH	tR [min]	Area [µV·sec]	Height [µV]	Area%	Height%	Quantity	NTP	Resolution	Symmetry Factor	Warning
1	Unknown	5	19.637	7732542	136189	50.093	57.667	N/A	2994	7.391	1.722	
2	Unknown	5	32.033	7703974	99974	49.907	42.333	N/A	4418	N/A	1.585	

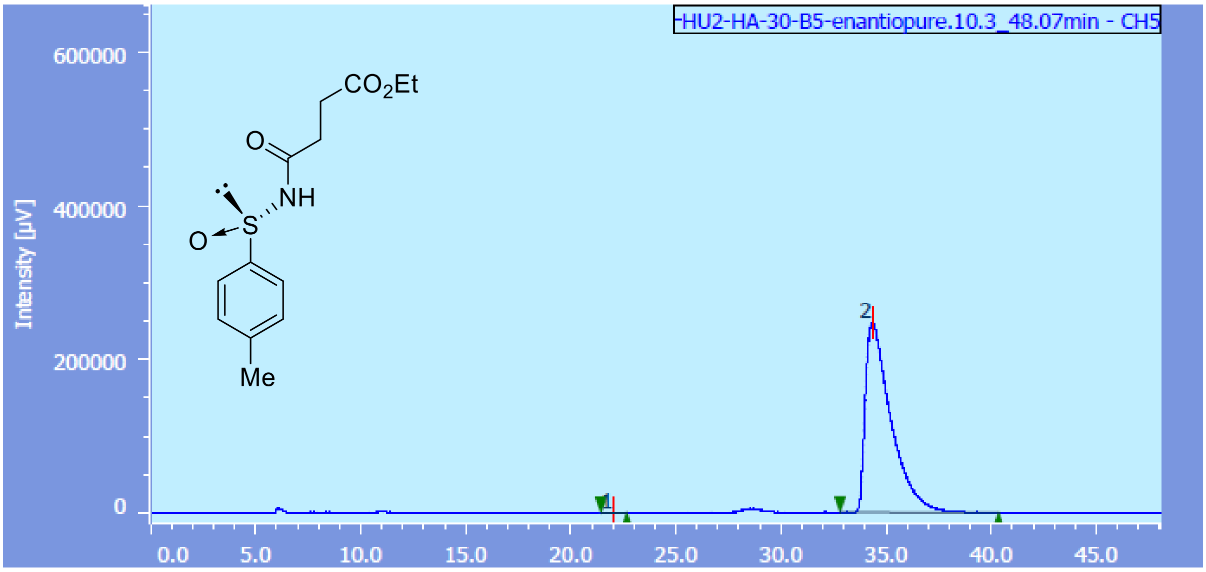

#	Peak Name	CH	tR [min]	Area [µV·sec]	Height [µV]	Area%	Height%	Quantity	NTP	Resolution	Symmetry Factor	Warning
1	Unknown	5	22.033	20594	483	0.098	0.195	N/A	5358	7.448	1.001	
2	Unknown	5	34.333	20941011	246931	99.902	99.805	N/A	4246	N/A	2.515	

(*S*)-3-[(*tert*-Butyldimethylsilyl)oxy)-*N*-(*S*)-*p*-tolylsulfinyl]butanamide (1f)

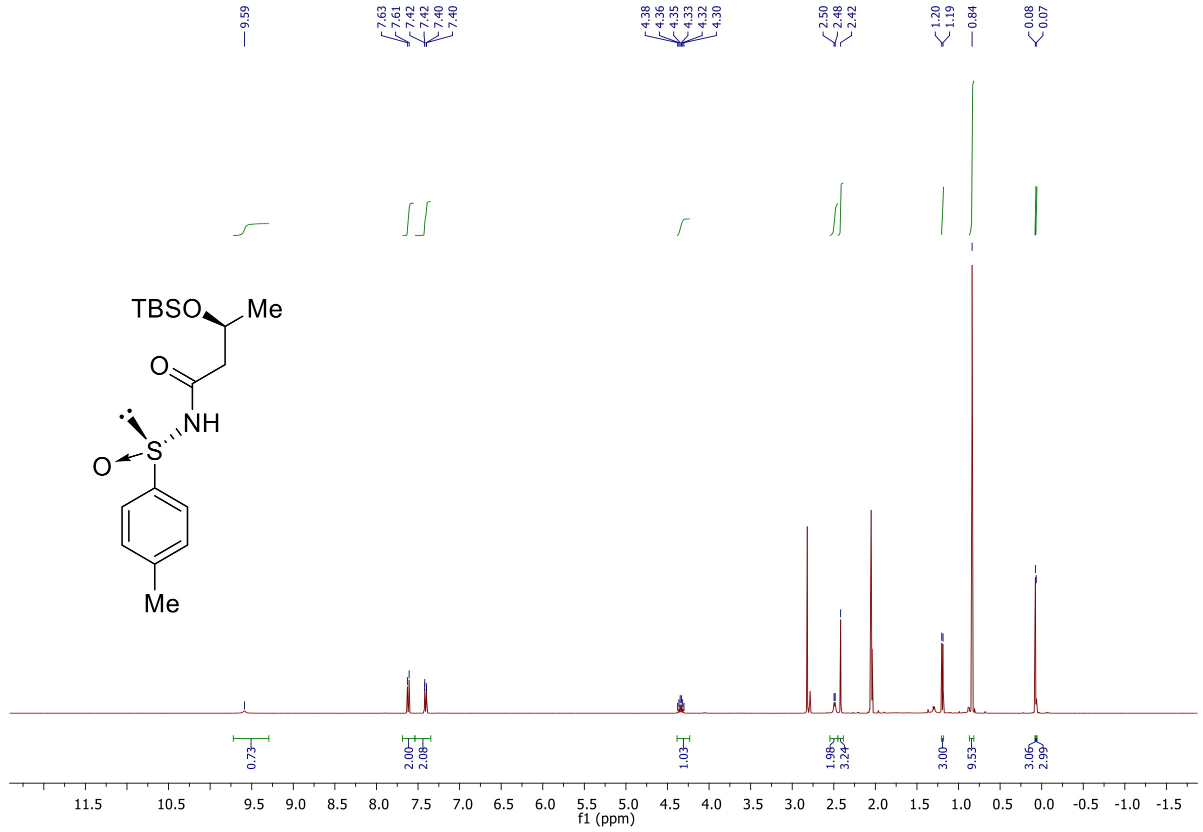

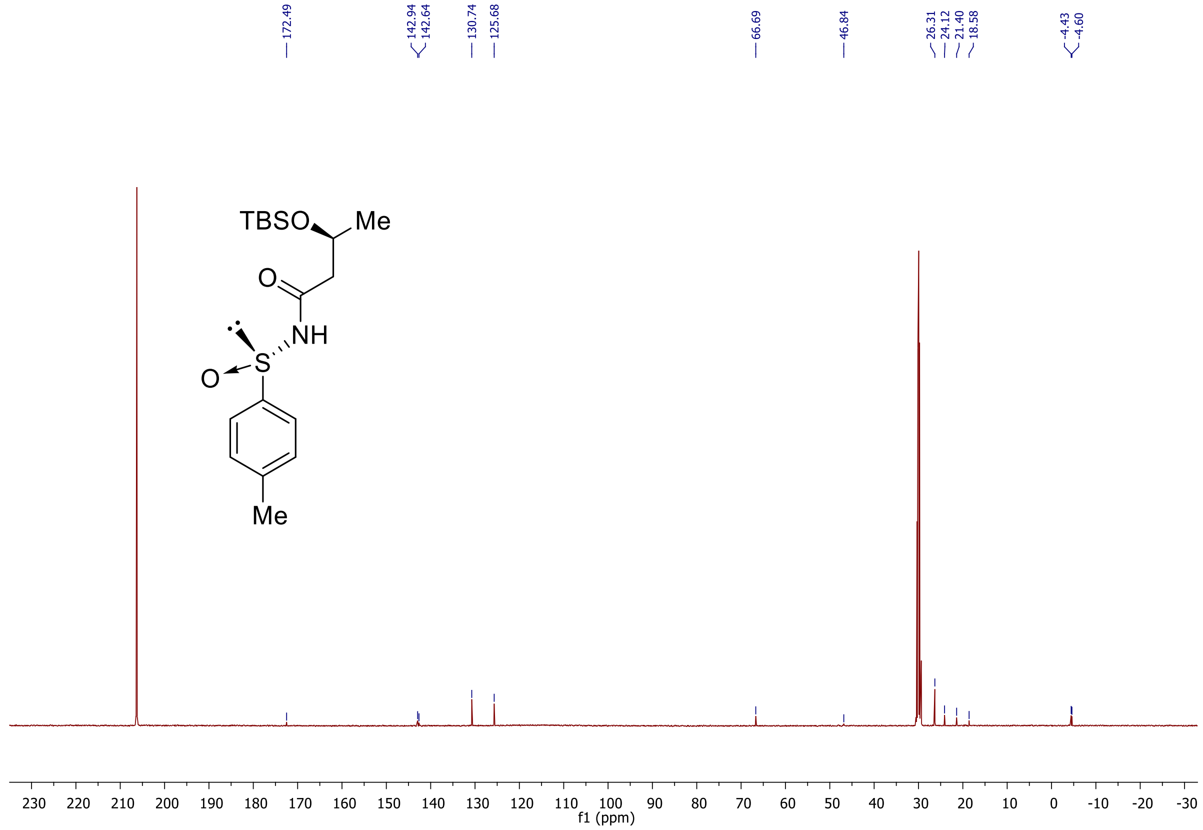

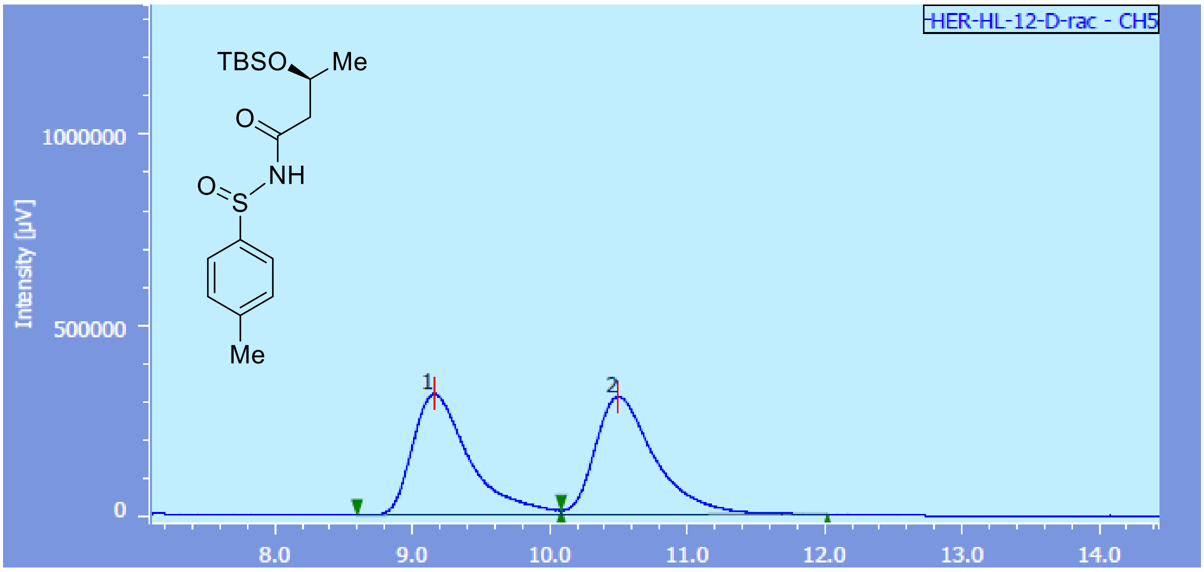

#	Peak Name	CH	tR [min]	Area [µV·sec]	Height [µV]	Area%	Height%	Quantity	NTP	Resolution	Symmetry Factor	Warning
1	Unknown	5	9.160	9092305	316149	49.336	50.615	N/A	2722	1.852	1.784	
2	Unknown	5	10.500	9337092	308462	50.664	49.385	N/A	3148	N/A	1.540	

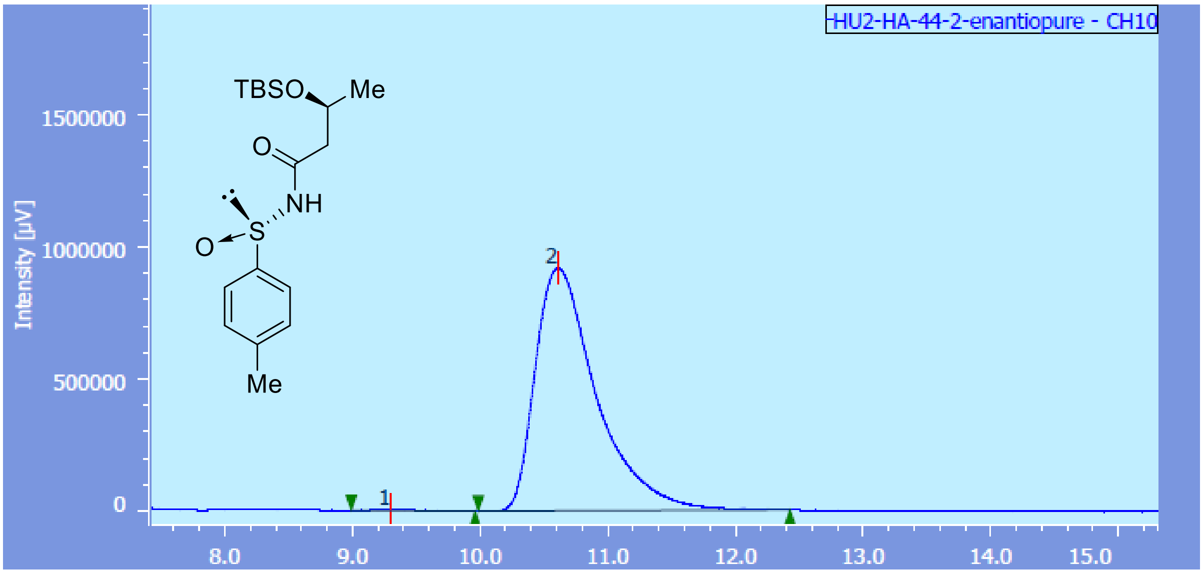

#	Peak Name	CH	tR [min]	Area [µV·sec]	Height [µV]	Area%	Height%	Quantity	NTP	Resolution	Symmetry Factor	Warning
1	Unknown	10	9.300	126475	4834	0.411	0.524	N/A	2804	1.723	1.454	
2	Unknown	10	10.610	30612006	918476	99.589	99.476	N/A	2662	N/A	1.744	

(*S*)-*N*- (*p*-Tolylsulfinyl)benzamide (1g)

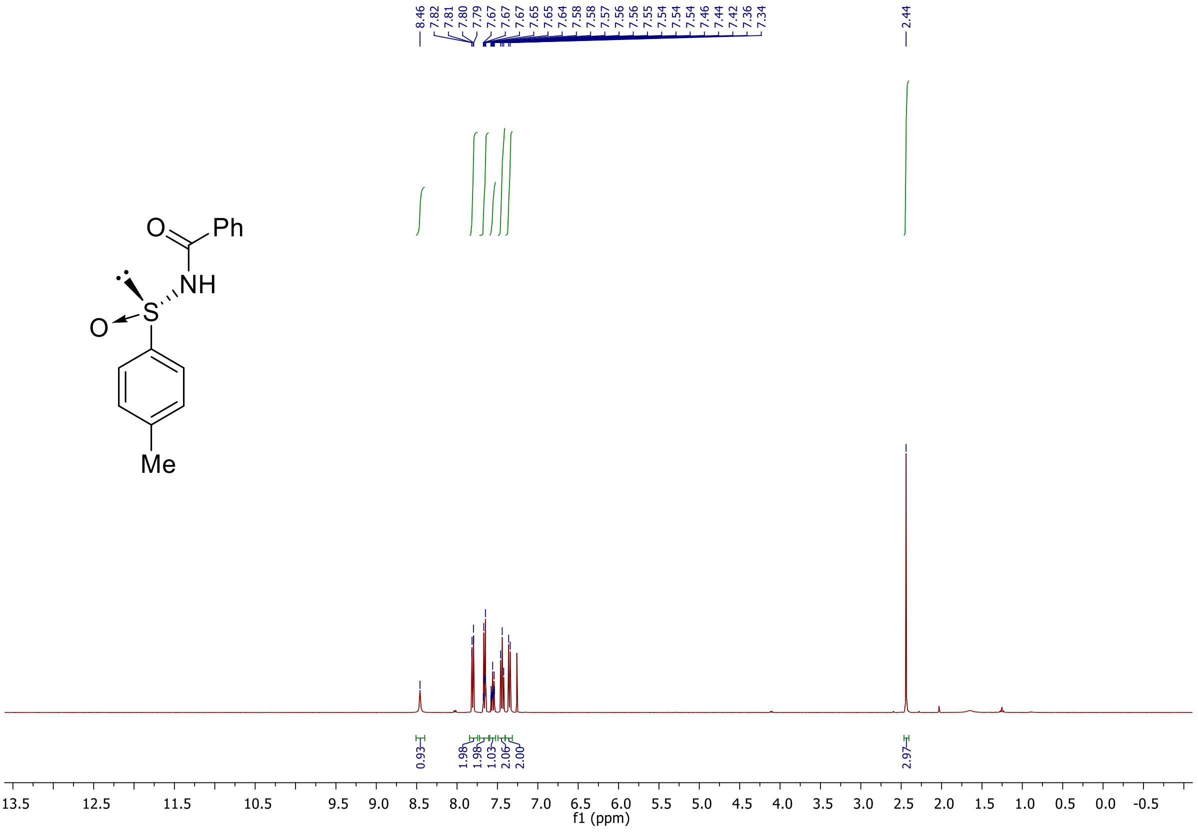

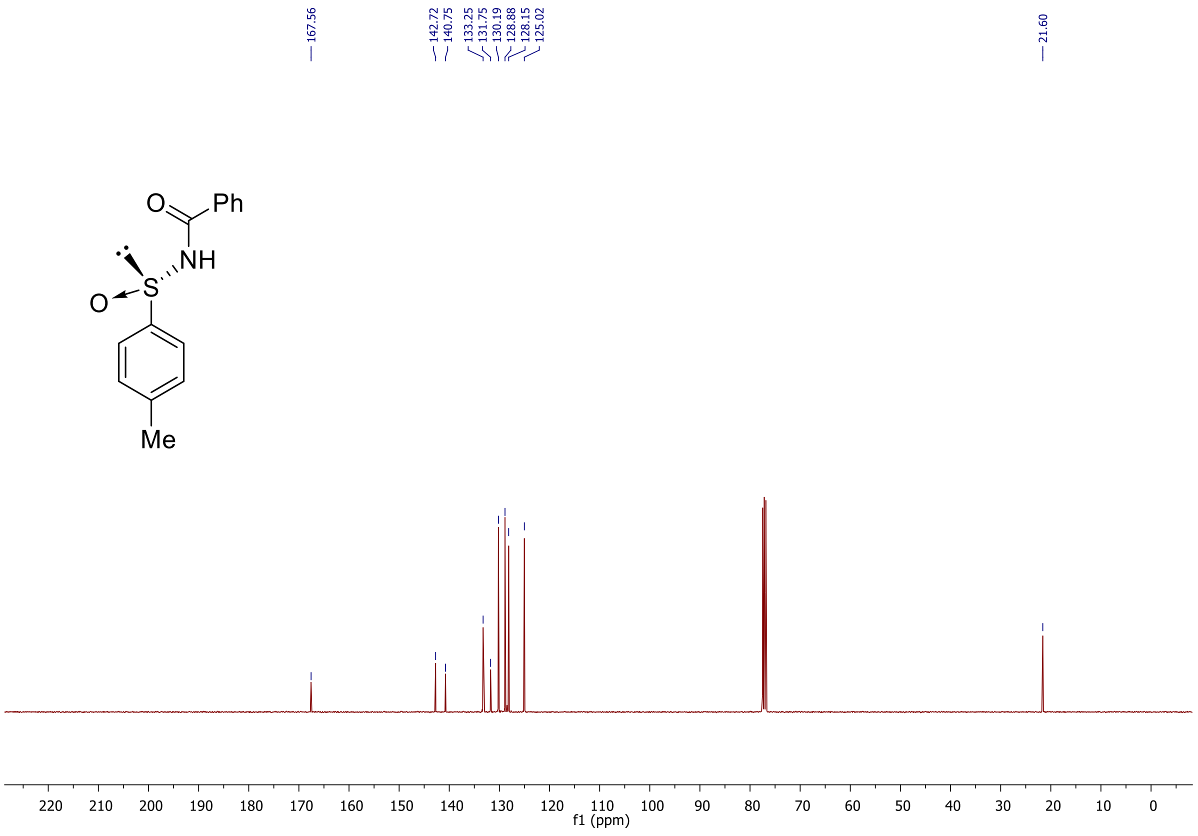

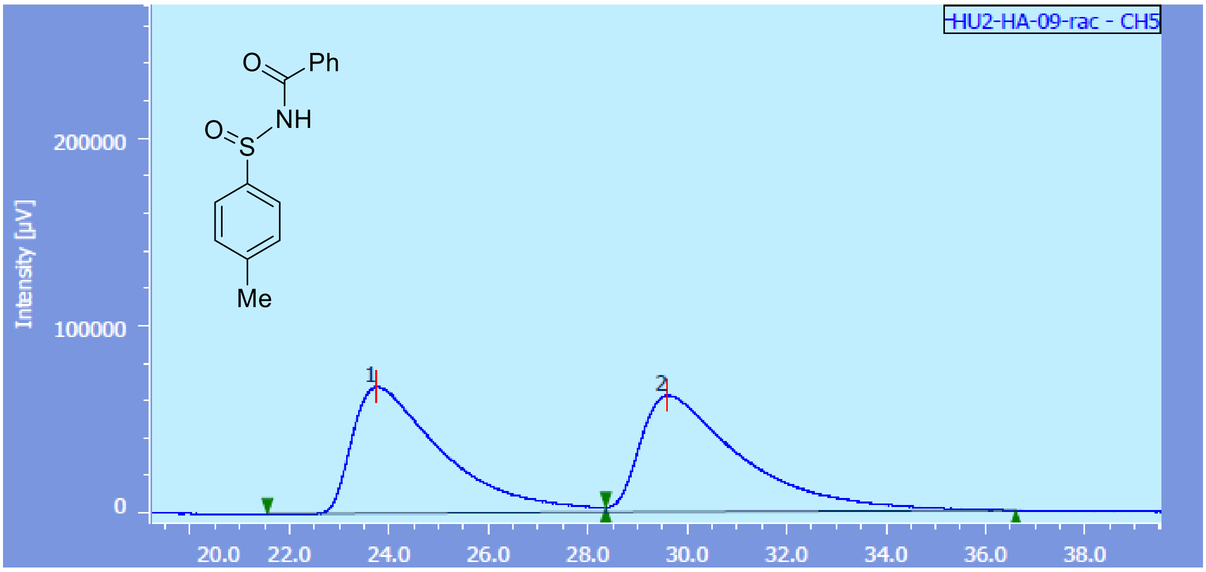

#	Peak Name	CH	tR [min]	Area [µV·sec]	Height [µV]	Area%	Height%	Quantity	NTP	Resolution	Symmetry Factor	Warning
1	Unknown	5	23.757	8575546	67677	49.129	52.084	N/A	924	1.787	2.723	
2	Unknown	5	29.607	8879743	62262	50.871	47.916	N/A	1186	N/A	2.467	

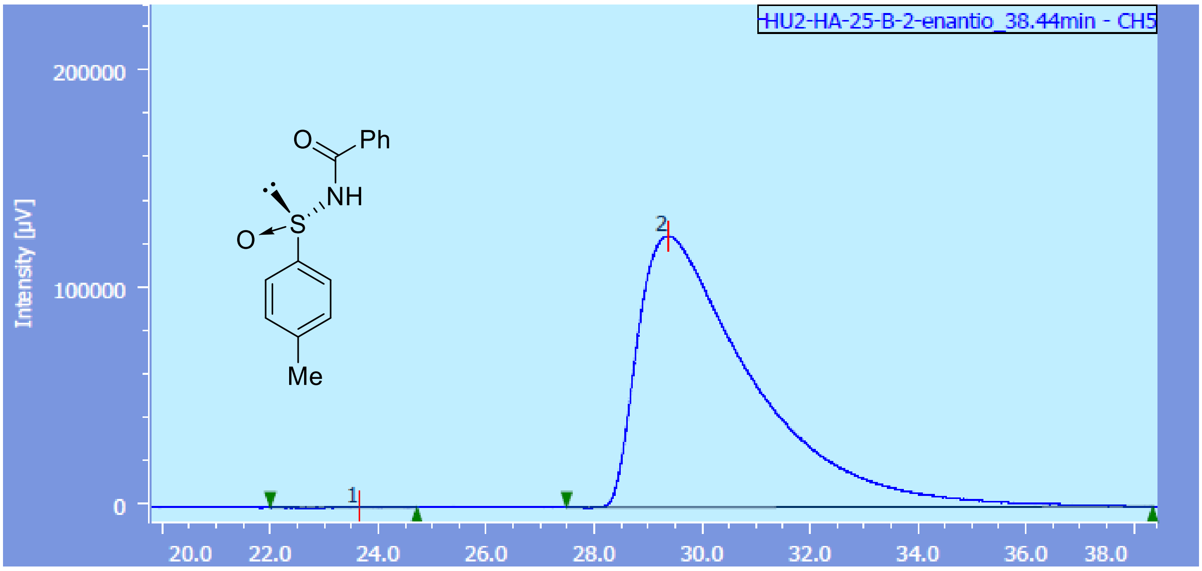

#	Peak Name	CH	tR [min]	Area [µV·sec]	Height [µV]	Area%	Height%	Quantity	NTP	Resolution	Symmetry Factor	Warning
1	Unknown	5	23.653	1652	115	0.009	0.092	N/A	41045	2.827	0.834	
2	Unknown	5	29.373	18457418	124958	99.991	99.908	N/A	1071	N/A	2.790	

(*S*)-*N*- (*p*-Tolylsulfinyl) furan-2-carboxamide (1h)

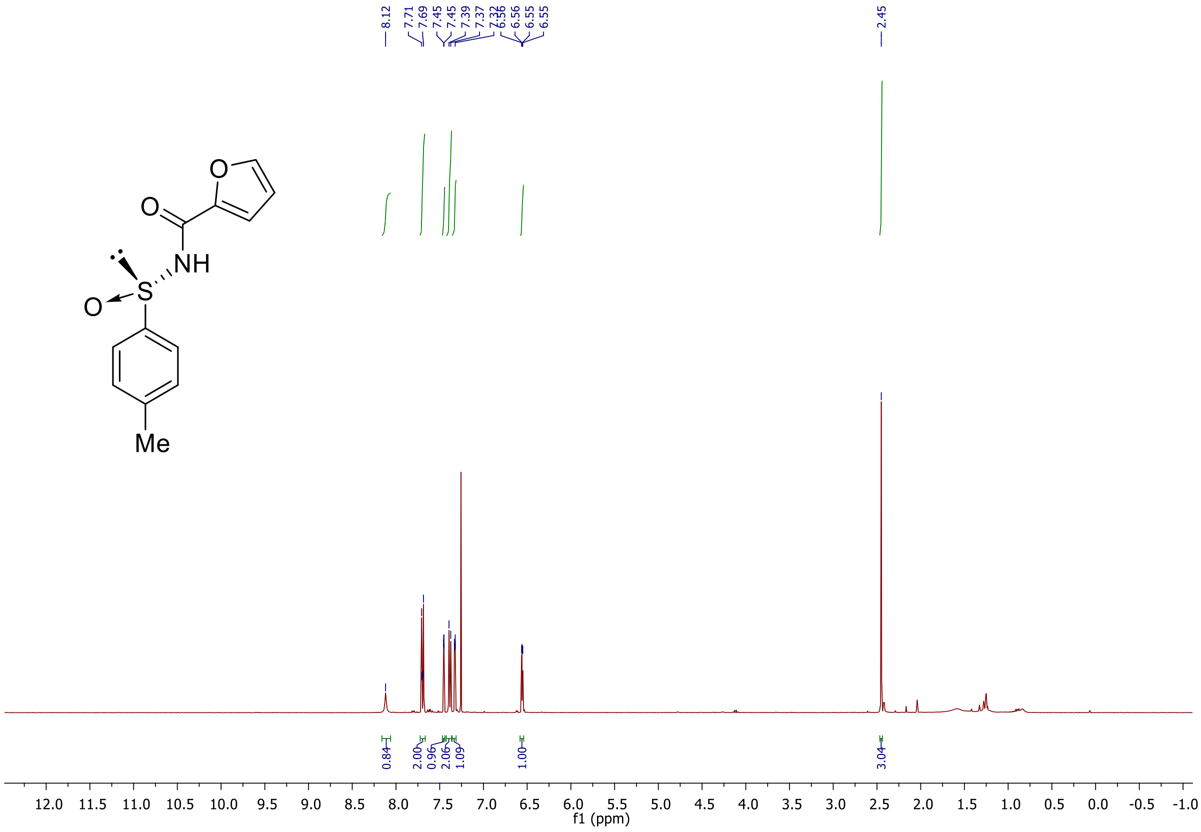

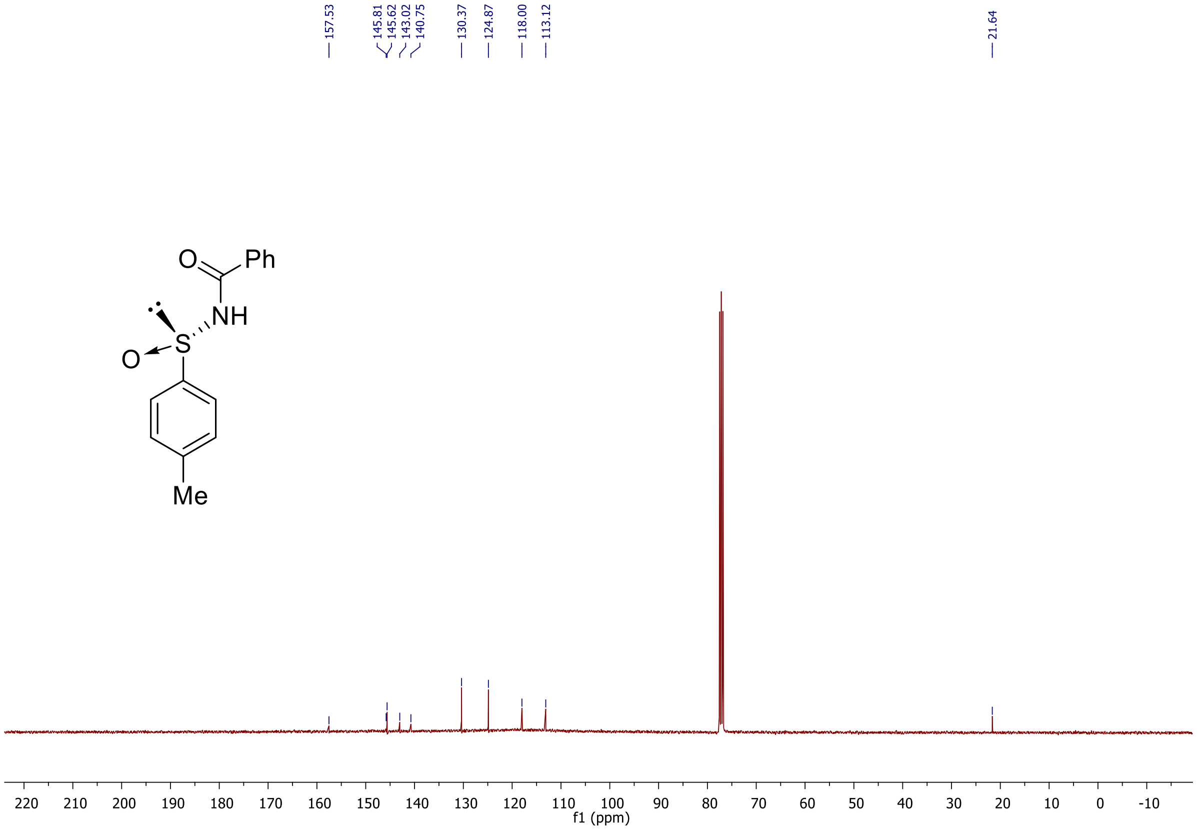

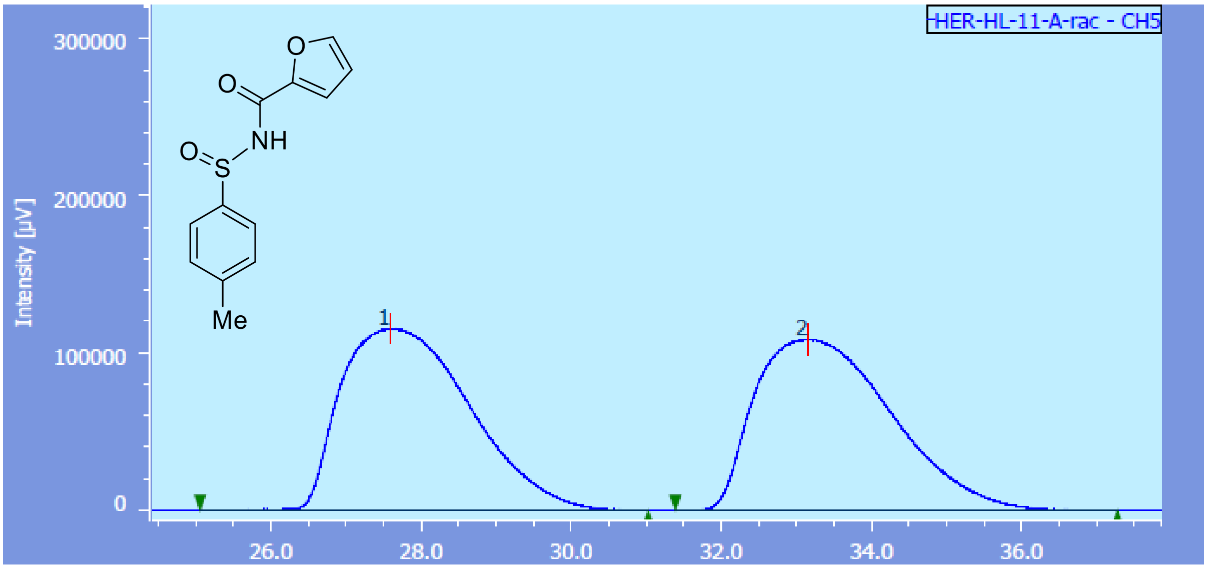

#	Peak Name	CH	tR [min]	Area [μV·sec]	Height [μV]	Area%	Height%	Quantity	NTP	Resolution	Symmetry Factor	Warning
1	Unknown	5	27.593	13844898	115555	50.048	51.529	N/A	1106	1.635	1.553	
2	Unknown	5	33.157	13818524	108697	49.952	48.471	N/A	1431	N/A	1.556	

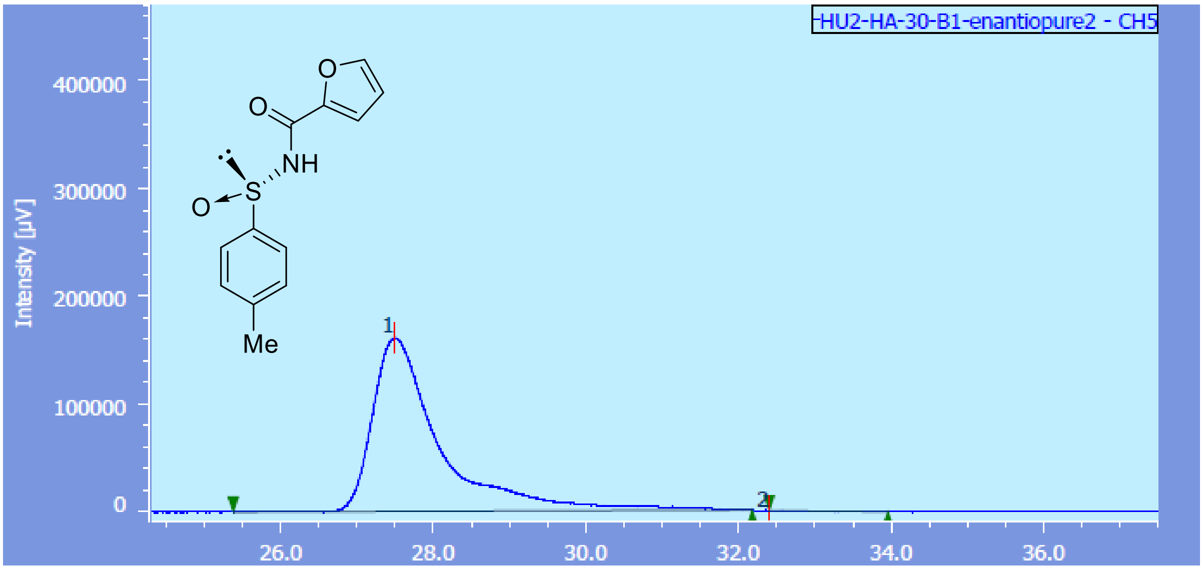

#	Peak Name	CH	tR [min]	Area [μV·sec]	Height [μV]	Area%	Height%	Quantity	NTP	Resolution	Symmetry Factor	Warning
1	Unknown	5	27.493	9847452	160912	100.000	99.995	N/A	7036	N/A	2.288	
2	Unknown	5	32.403	2	8	0.000	0.005	N/A	N/A	N/A	N/A	

***ter*t-Butyl (*S*)-(*p*-tolylsulfinyl)carbamate (1i)**

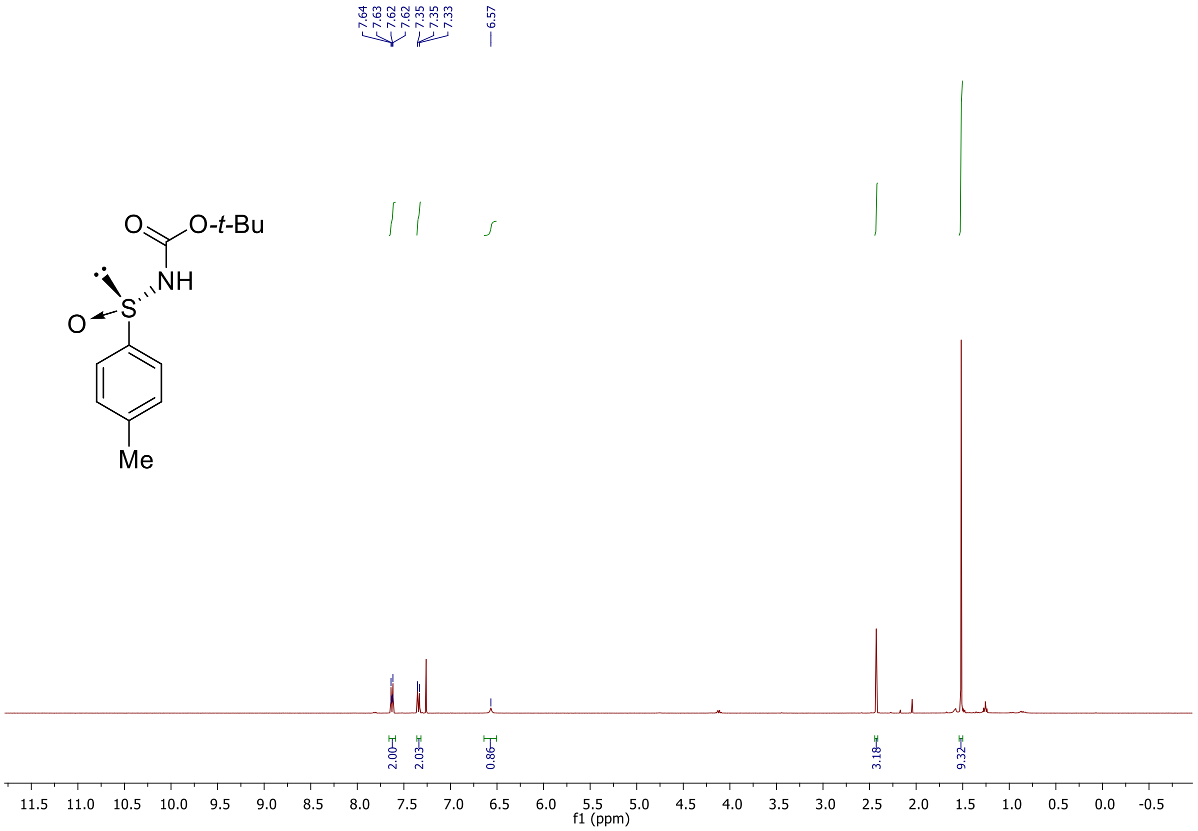

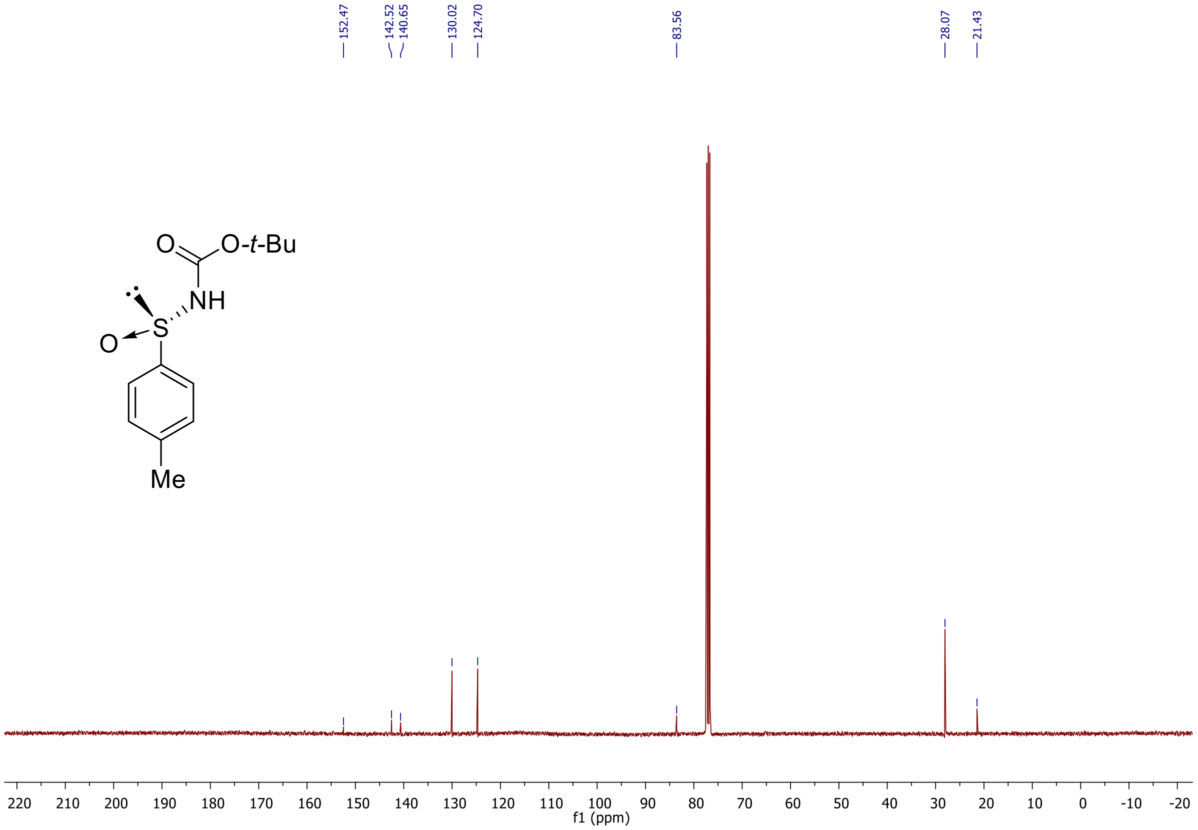

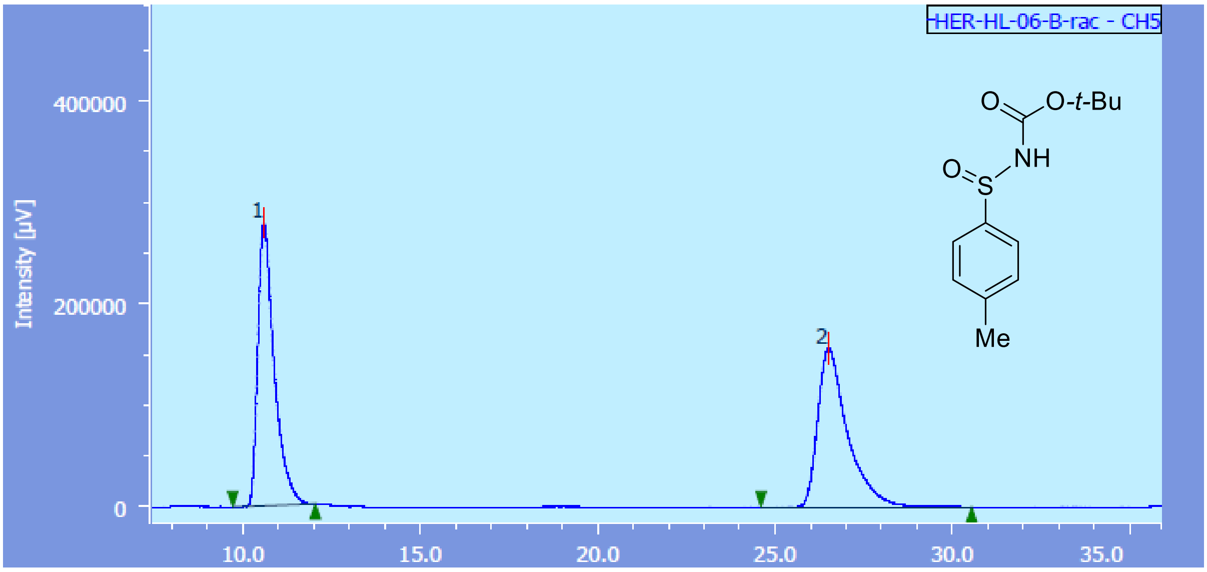

#	Peak Name	CH	tR [min]	Area [µV·sec]	Height [µV]	Area%	Height%	Quantity	NTP	Resolution	Symmetry Factor	Warning
1	Unknown	5	10.593	8989591	278899	49.638	64.066	N/A	2806	14.519	1.685	
2	Unknown	5	26.507	9120533	156430	50.362	35.934	N/A	5752	N/A	1.705	

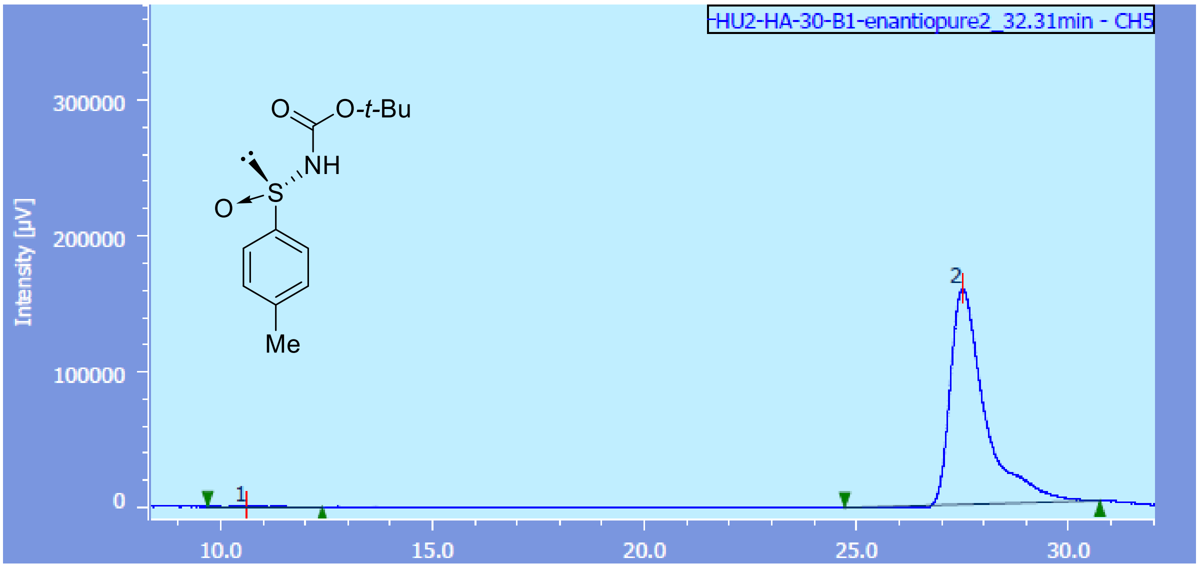

#	Peak Name	CH	tR [min]	Area [µV·sec]	Height [µV]	Area%	Height%	Quantity	NTP	Resolution	Symmetry Factor	Warning
1	Unknown	5	10.630	8632	149	0.095	0.094	N/A	378	9.705	1.458	
2	Unknown	5	27.493	9052358	159093	99.905	99.906	N/A	7185	N/A	2.114	

(*S*)-*N*-(Phenylsulfinyl)butyramide (1j)

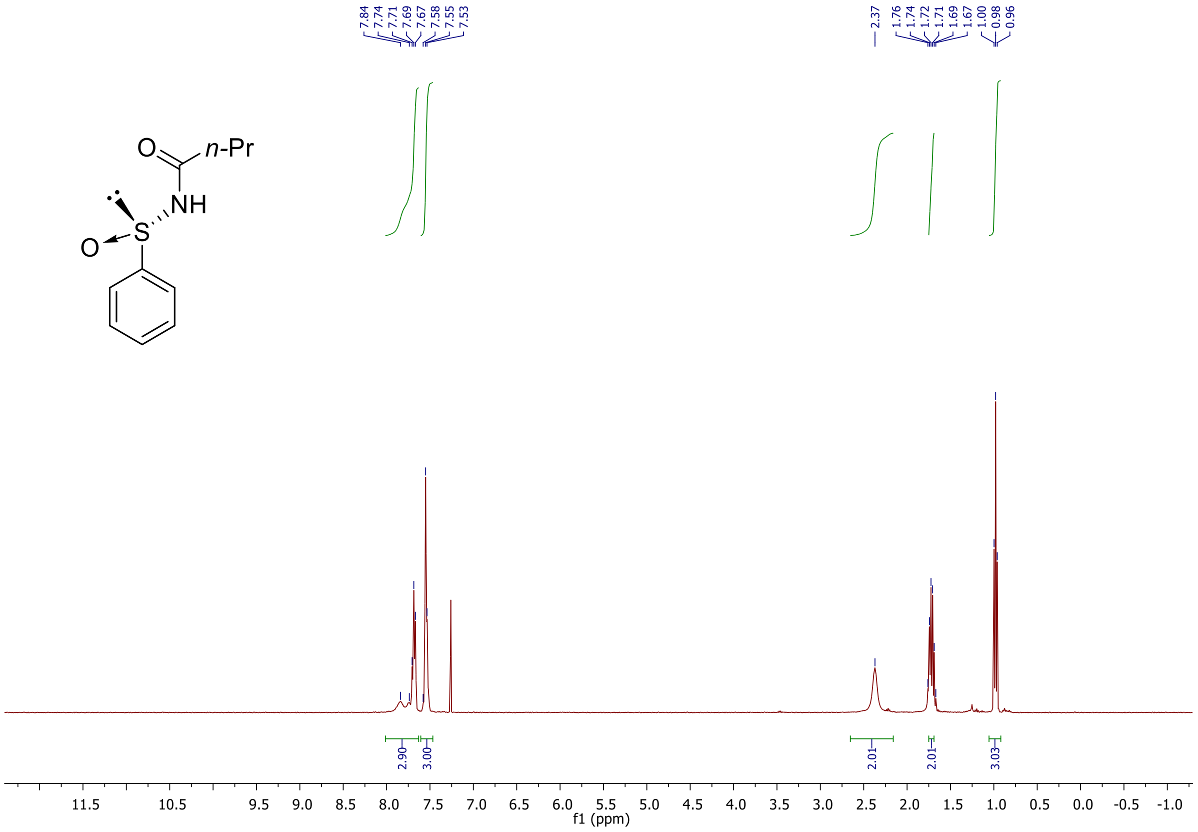

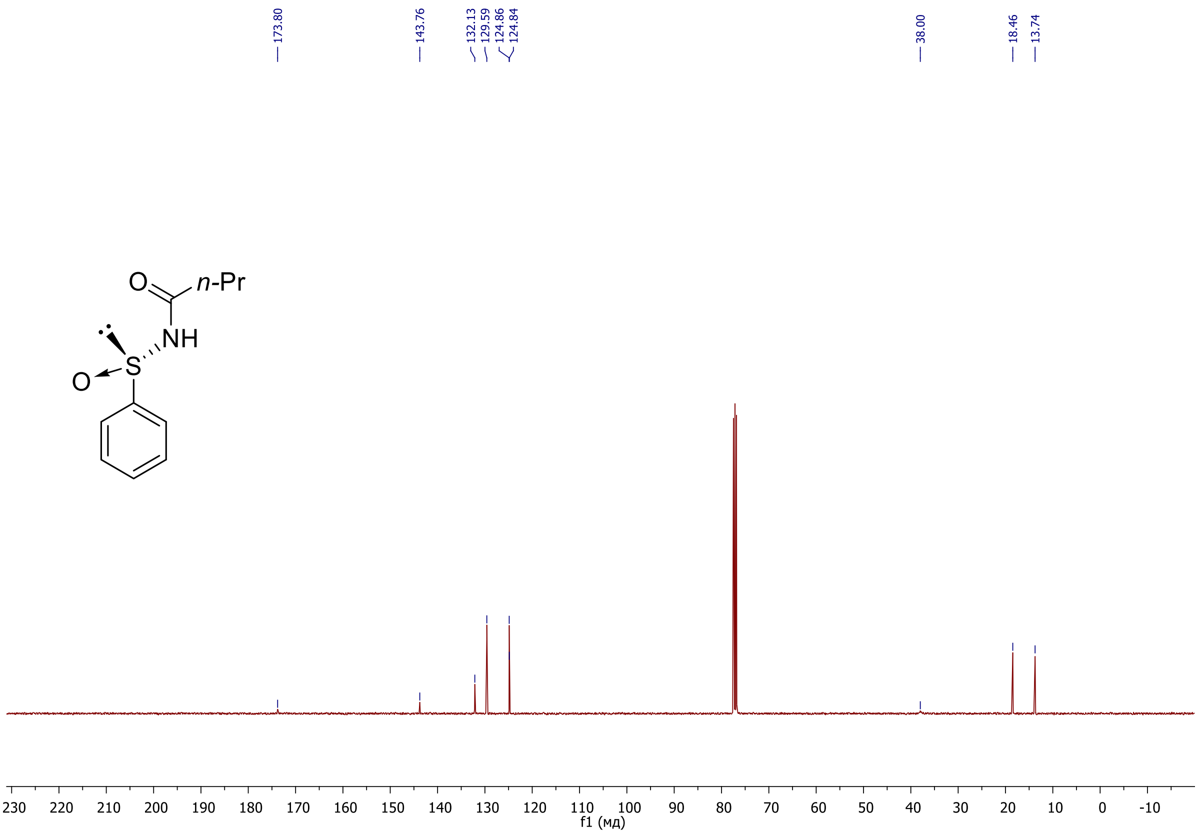

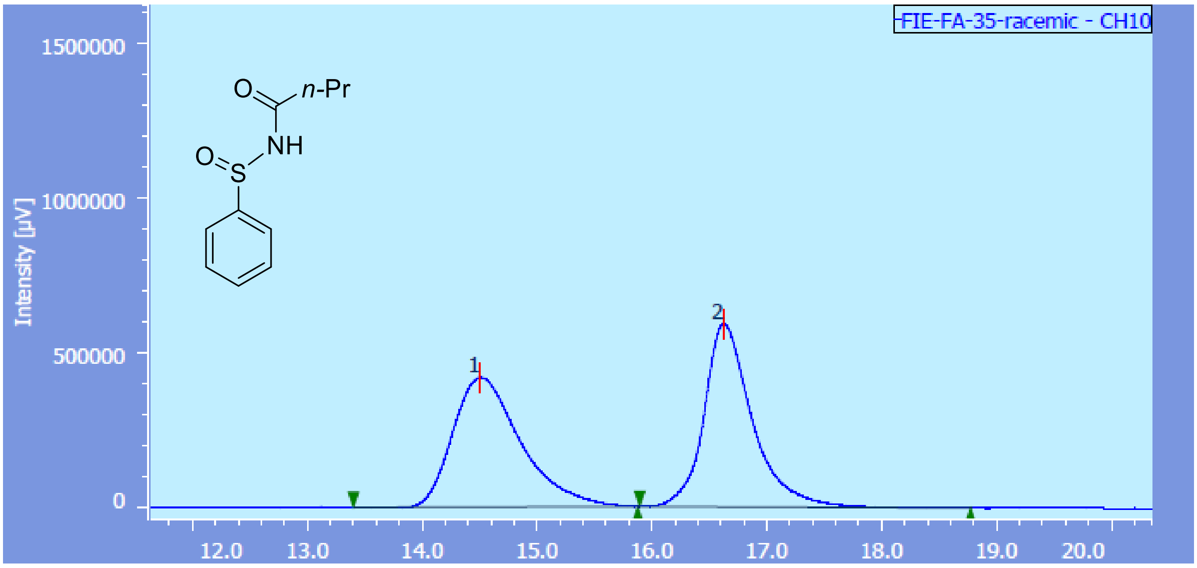

#	Peak Name	CH	tR [min]	Area [µV·sec]	Height [µV]	Area%	Height%	Quantity	NTP	Resolution	Symmetry Factor	Warning
1	Unknown	10	14.507	17257618	419232	49.839	41.470	N/A	3045	2.431	1.461	
2	Unknown	10	16.627	17369224	591693	50.161	58.530	N/A	9106	N/A	1.325	

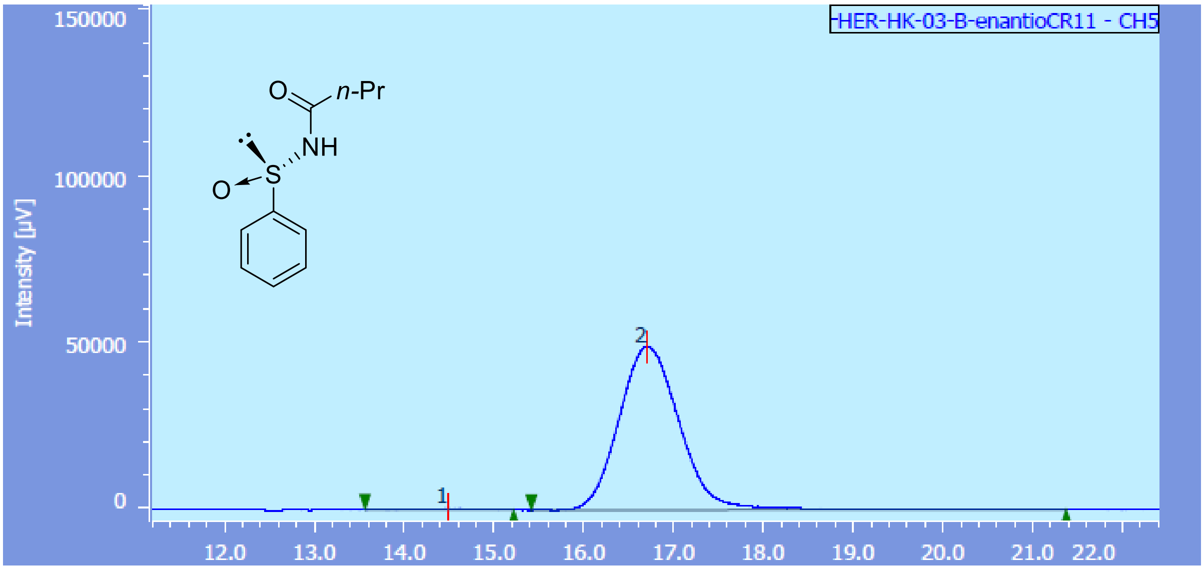

#	Peak Name	CH	tR [min]	Area [µV·sec]	Height [µV]	Area%	Height%	Quantity	NTP	Resolution	Symmetry Factor	Warning
1	Unknown	5	14.490	11261	319	0.487	0.645	N/A	3107	1.963	0.929	
2	Unknown	5	16.703	2301252	49100	99.513	99.355	N/A	2991	N/A	1.133	

(*S*)-*N*-[(4-Bromophenyl)sulfinyl]butyramide (1k)

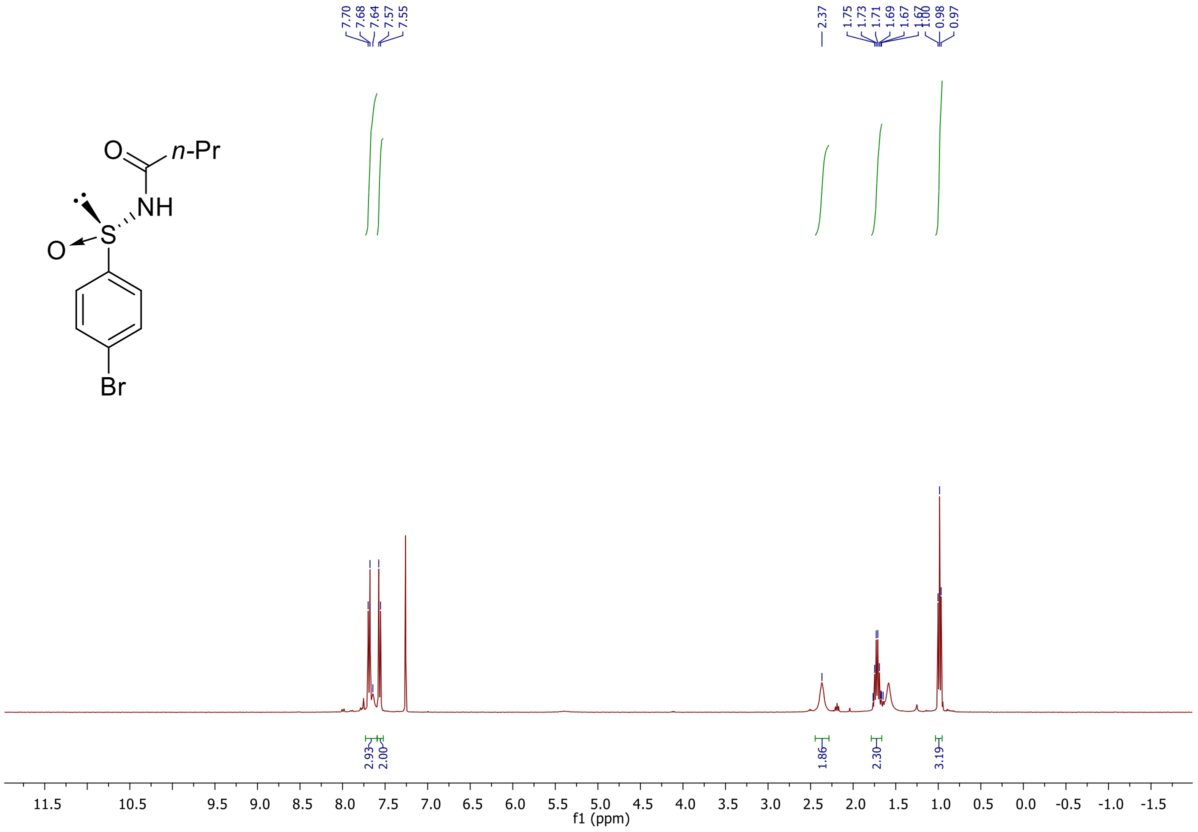

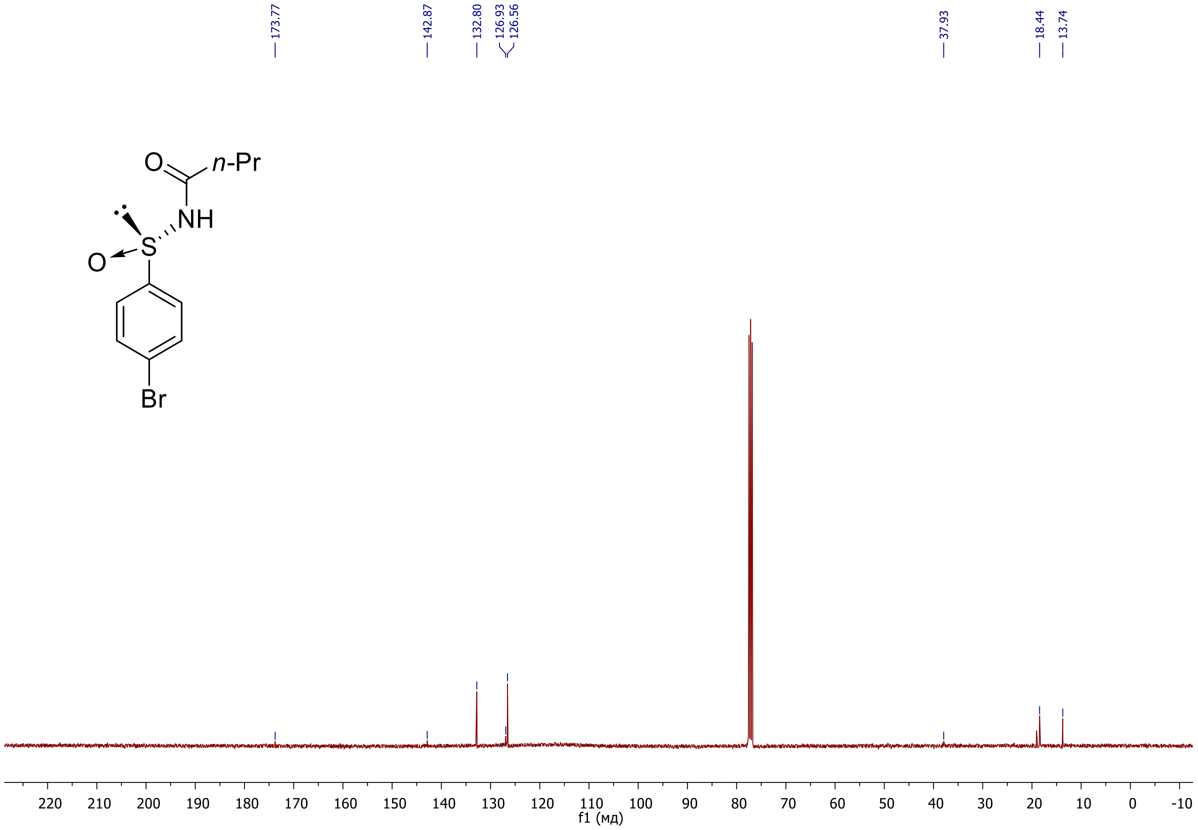

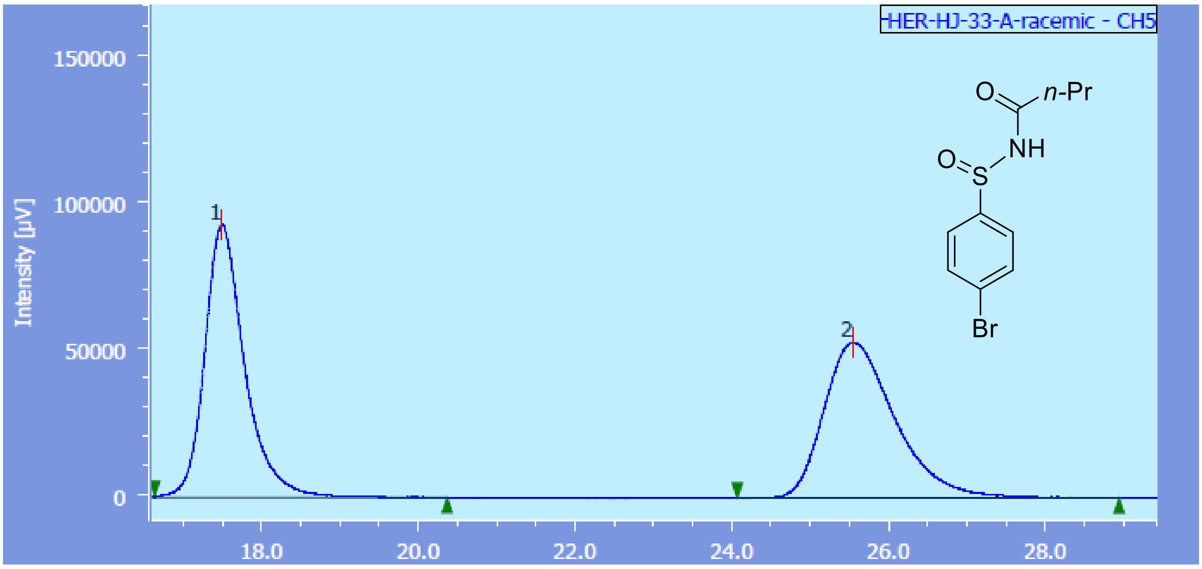

#	Peak Name	CH	tR [min]	Area [µV·sec]	Height [µV]	Area%	Height%	Quantity	NTP	Resolution	Symmetry Factor	Warning
1	Unknown	5	17.493	3311646	92997	49.619	63.630	N/A	6504	6.514	1.324	
2	Unknown	5	25.553	3362454	53156	50.381	36.370	N/A	4013	N/A	1.406	

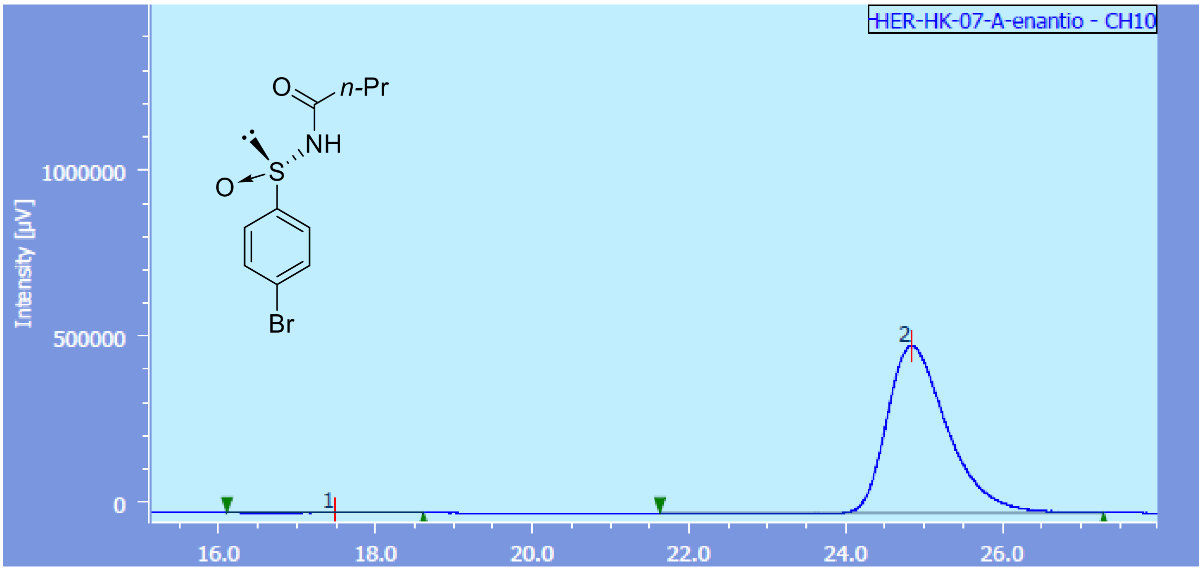

#	Peak Name	CH	tR [min]	Area [µV·sec]	Height [µV]	Area%	Height%	Quantity	NTP	Resolution	Symmetry Factor	Warning
1	Unknown	10	17.487	21923	740	0.082	0.147	N/A	9061	6.999	2.477	
2	Unknown	10	24.837	26851532	502151	99.918	99.853	N/A	5250	N/A	1.442	

(*S*)-*N*-[(4-Fluorophenyl)sulfinyl]butyramide (1l)

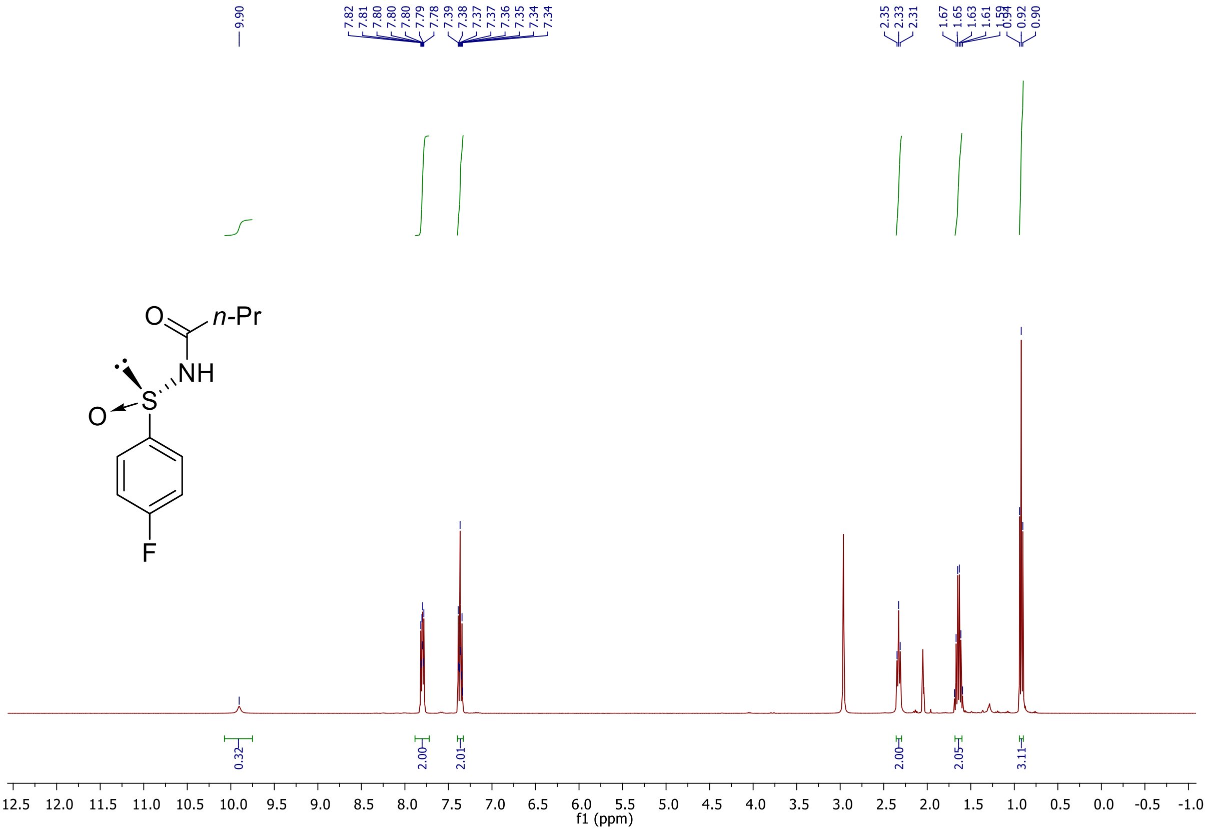

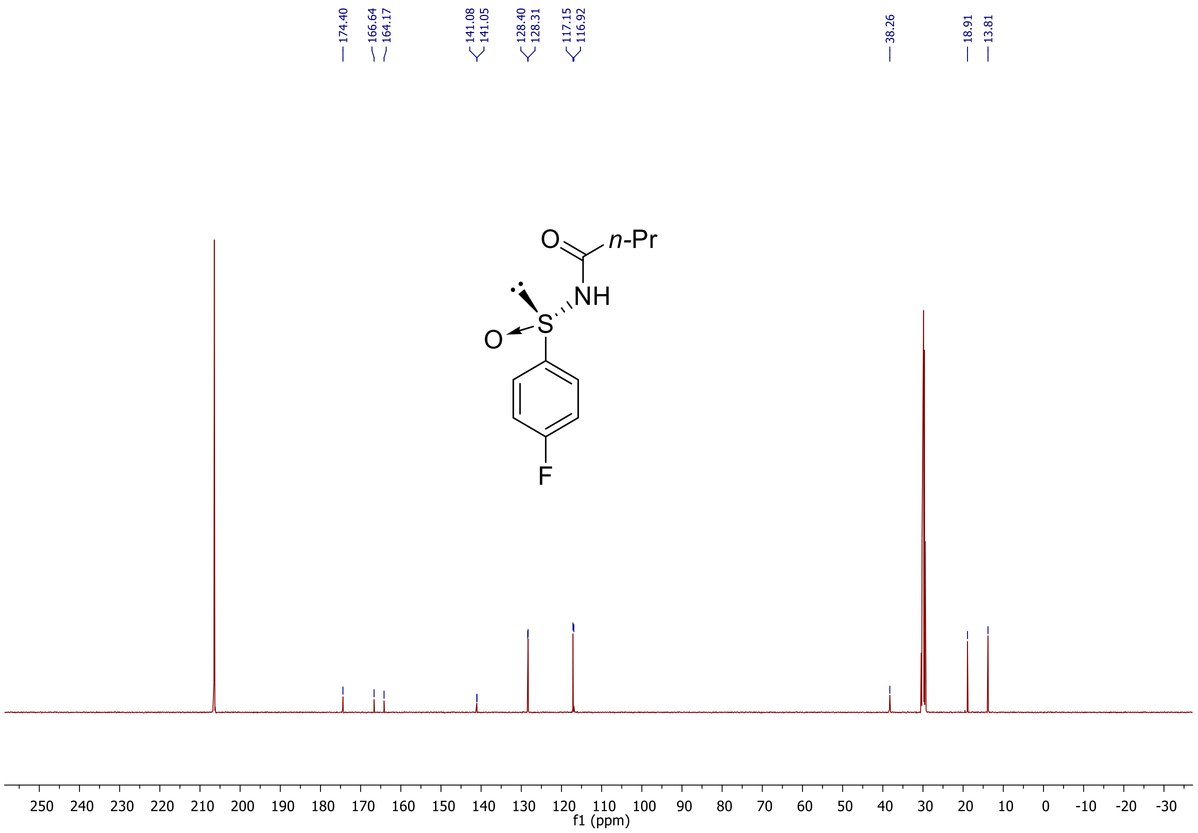

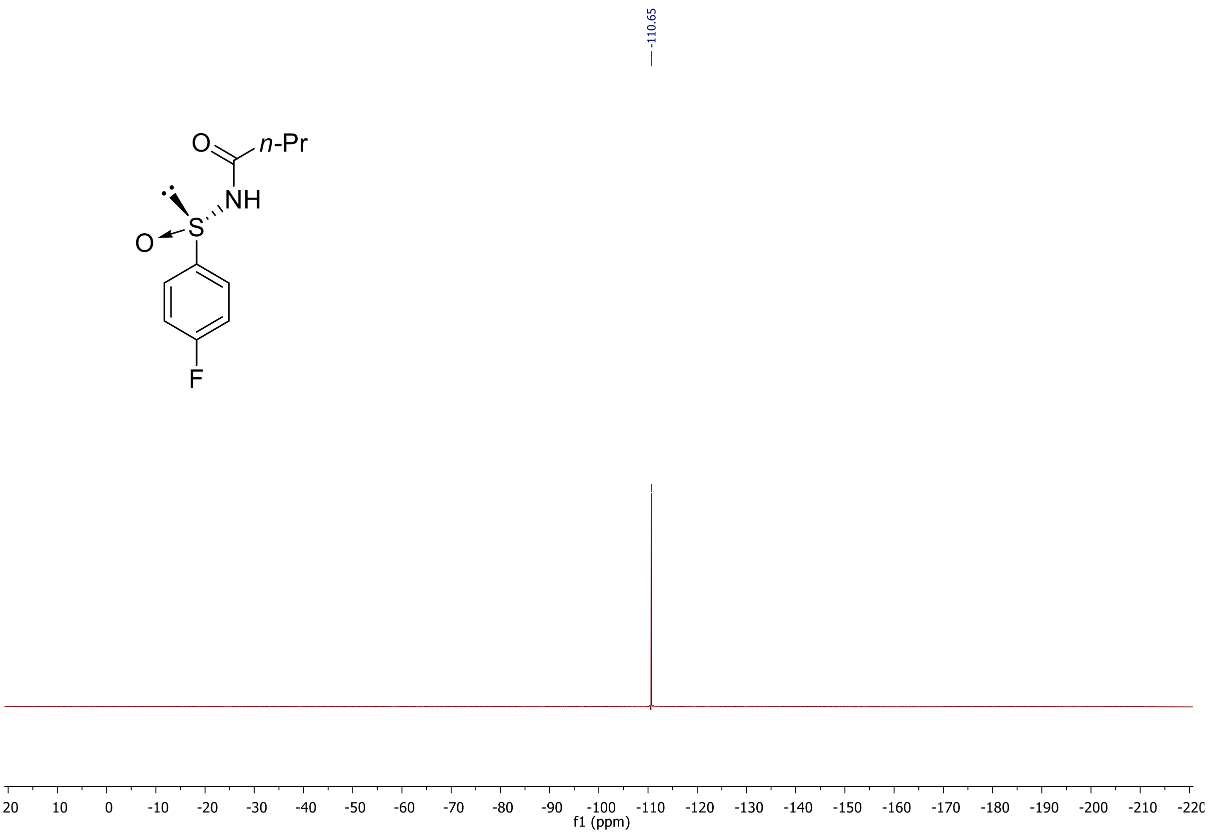
-110.65
O
n-Pr
NH
S
O
F
20 10 0 -10 -20 -30 -40 -50 -60 -70 -80 -90 -100 -110 -120 -130 -140 -150 -160 -170 -180 -190 -200 -210 -220
f1 (ppm)

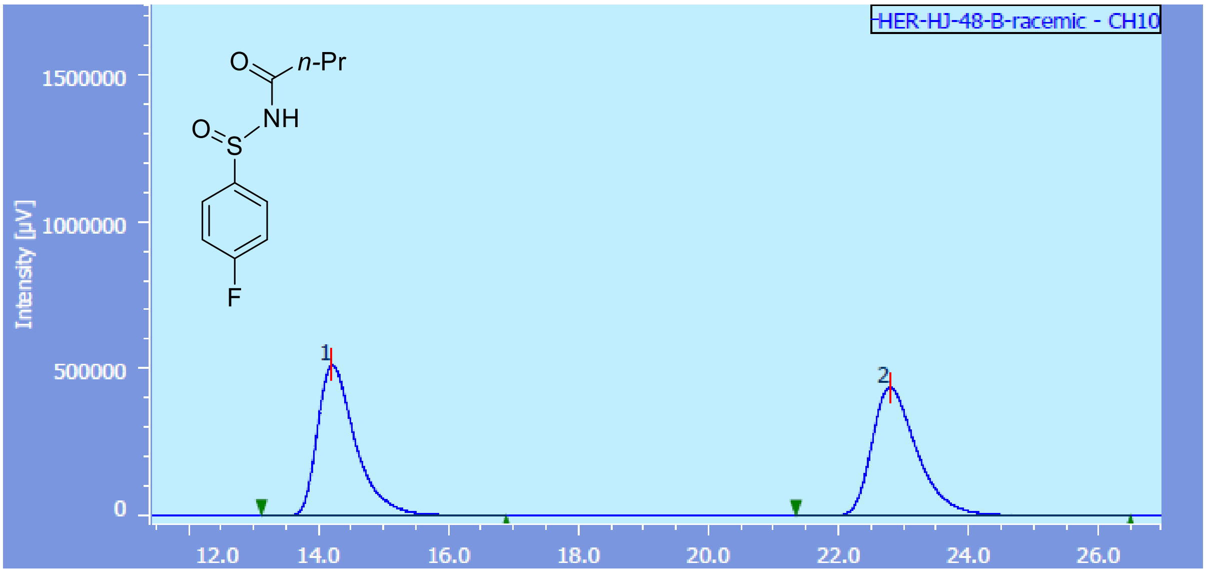

#	Peak Name	CH	tR [min]	Area [μV·sec]	Height [μV]	Area%	Height%	Quantity	NTP	Resolution	Symmetry Factor	Warning
1	Unknown	10	14.210	20878121	512810	49.660	54.018	N/A	3115	7.650	1.586	
2	Unknown	10	22.790	21163984	436519	50.340	45.982	N/A	5487	N/A	1.446	

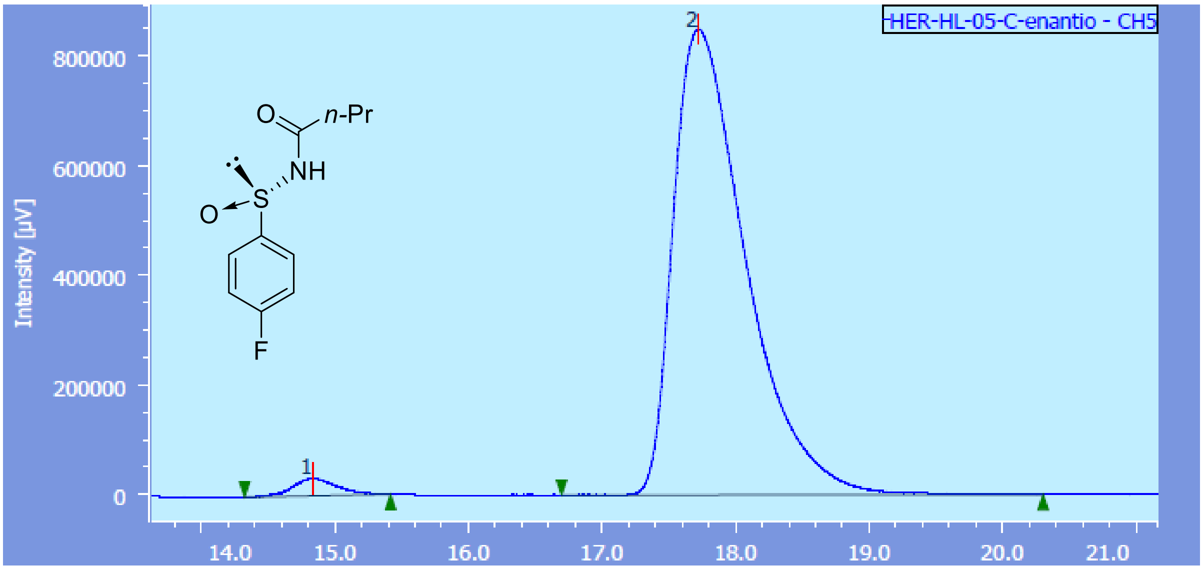

#	Peak Name	CH	tR [min]	Area [μV·sec]	Height [μV]	Area%	Height%	Quantity	NTP	Resolution	Symmetry Factor	Warning
1	Unknown	5	14.833	743568	31551	2.264	3.583	N/A	9939	3.713	1.149	
2	Unknown	5	17.723	32097481	849068	97.736	96.417	N/A	5390	N/A	1.801	

(*S*)-3-[(*tert*-Butyldimethylsilyl)oxy]-*N*-[(*S*)-(4-trifluoromethylphenyl)sulfinyl]butanamide (1m)

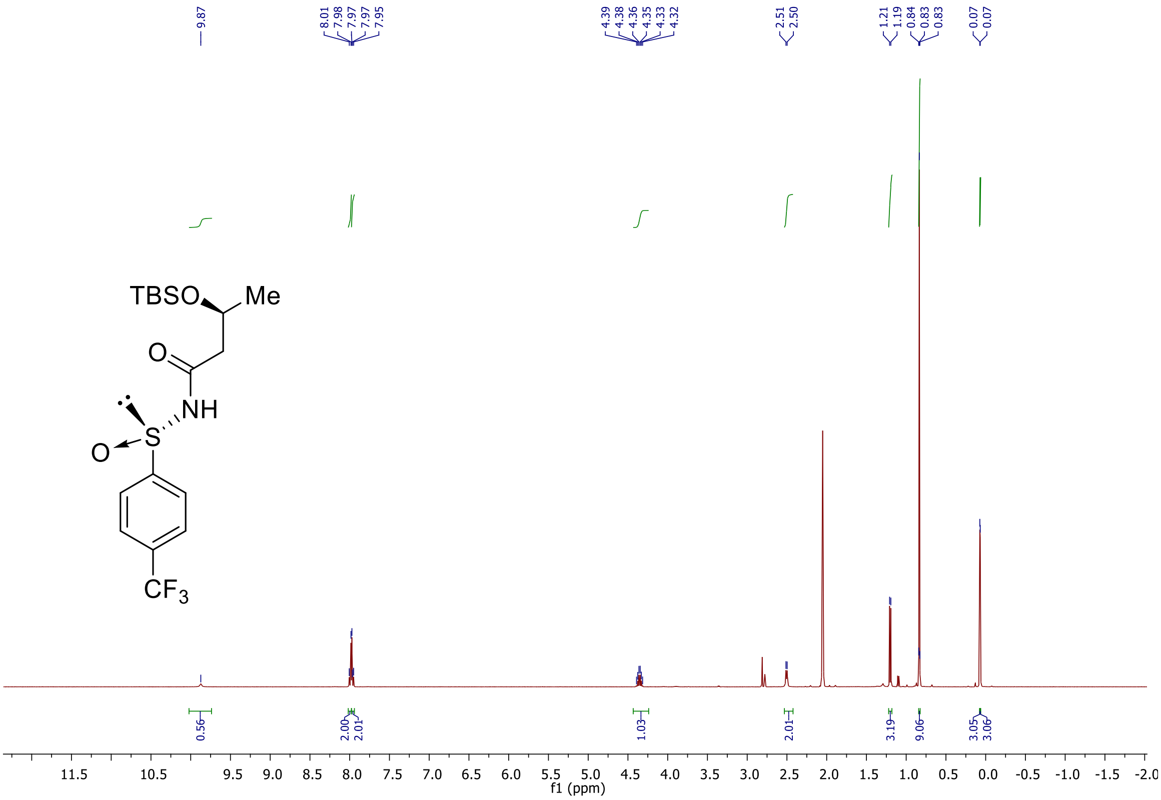

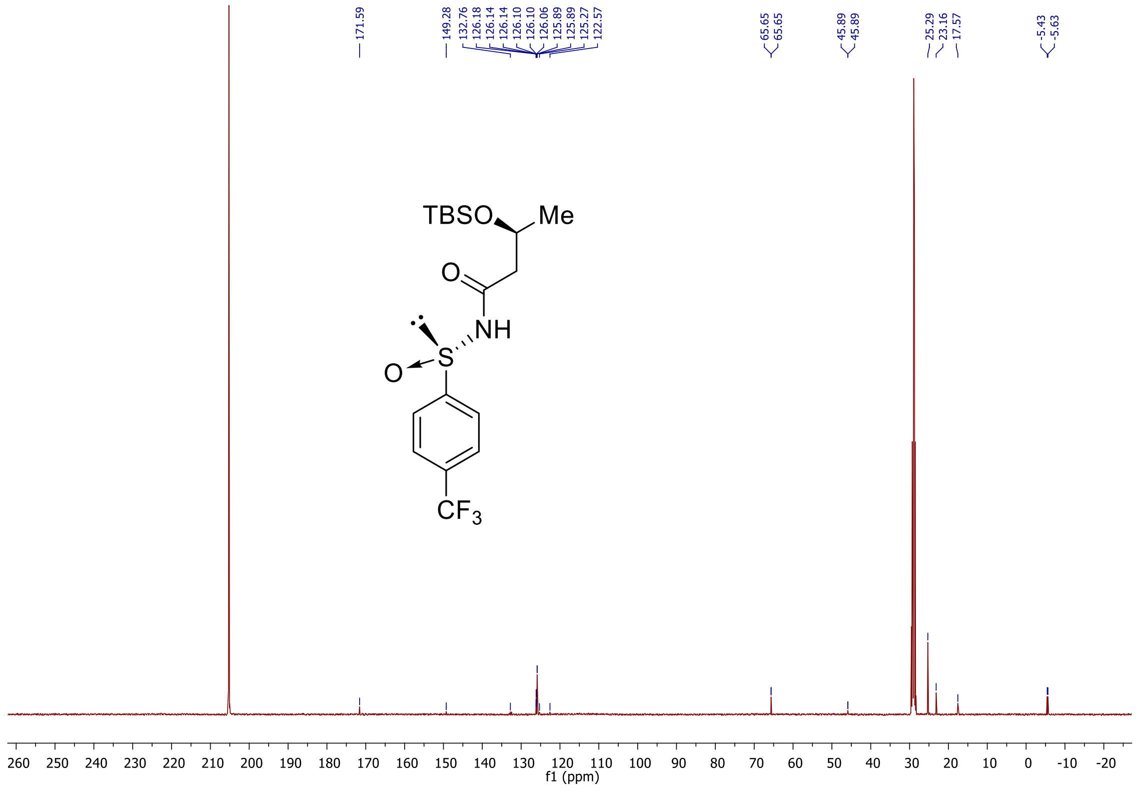

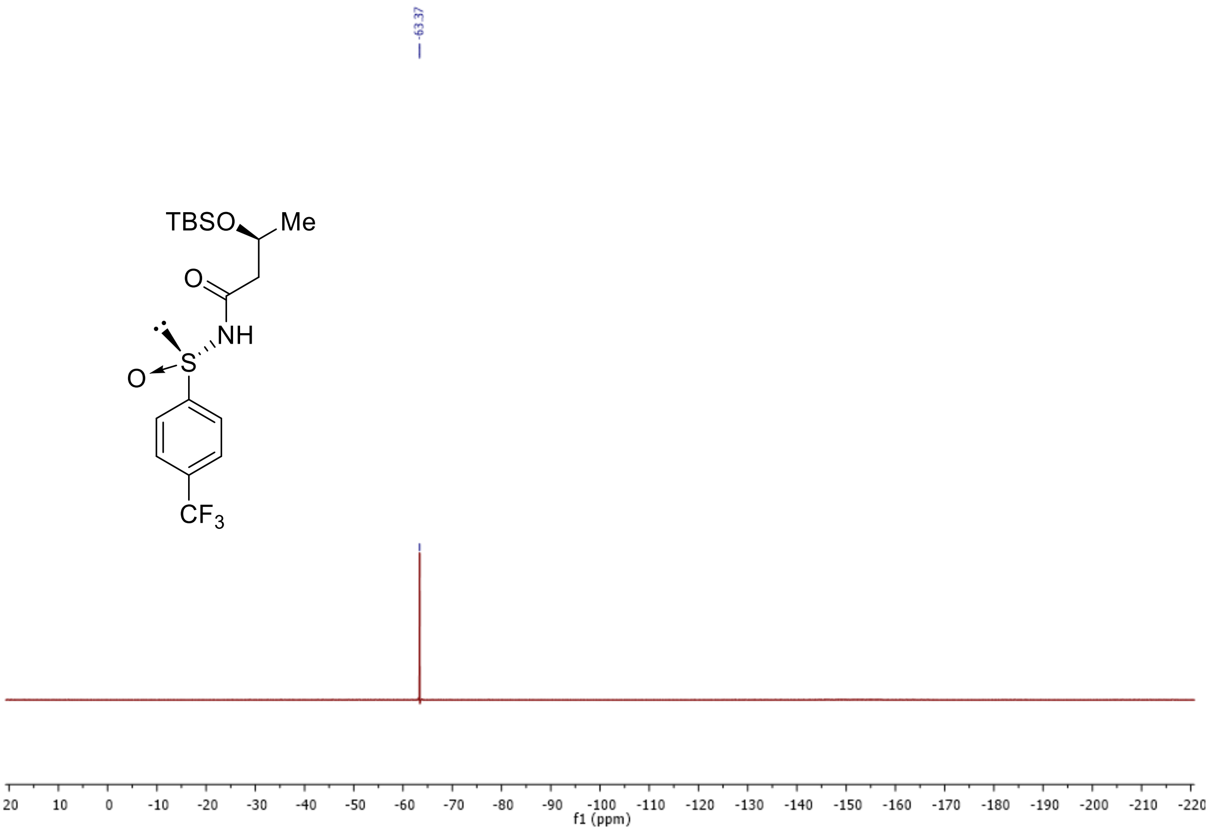

-63.37
TBSO
Me
O
NH
S
O
CF3
20
10
0
-10
-20
-30
-40
-50
-60
-70
-80
-90
-100
-110
-120
-130
-140
-150
-160
-170
-180
-190
-200
-210
-220
f1 (ppm)

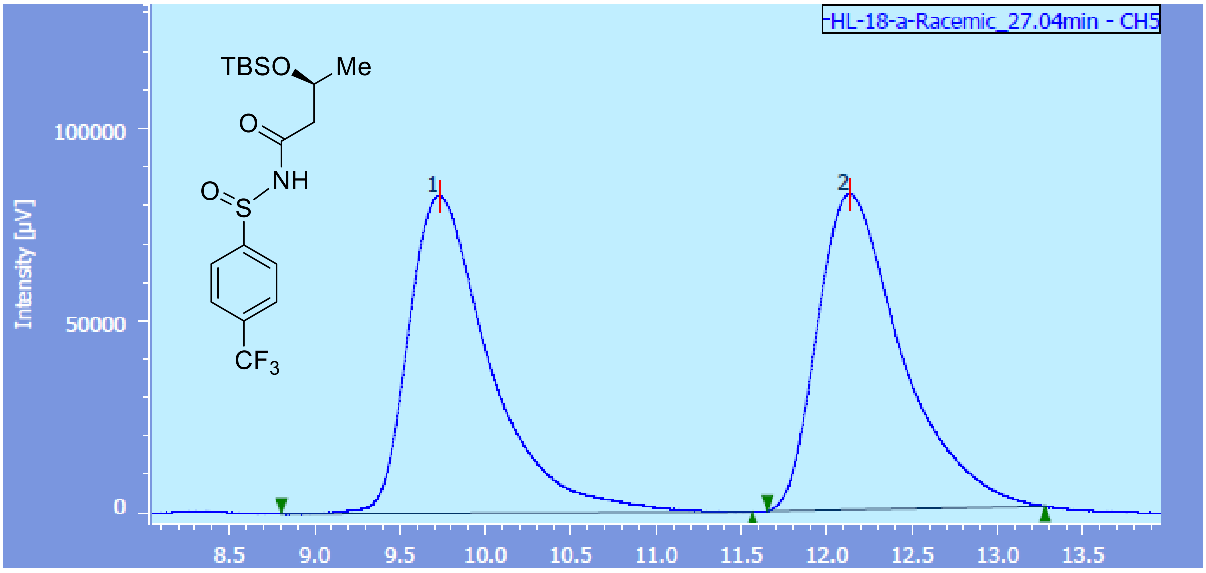

#	Peak Name	CH	tR [min]	Area [μV·sec]	Height [μV]	Area%	Height%	Quantity	NTP	Resolution	Symmetry Factor	Warning
1	Unknown	5	9.730	2752279	82509	49.341	50.148	N/A	2310	2.877	1.759	
2	Unknown	5	12.133	2825769	82021	50.659	49.852	N/A	3144	N/A	1.613	

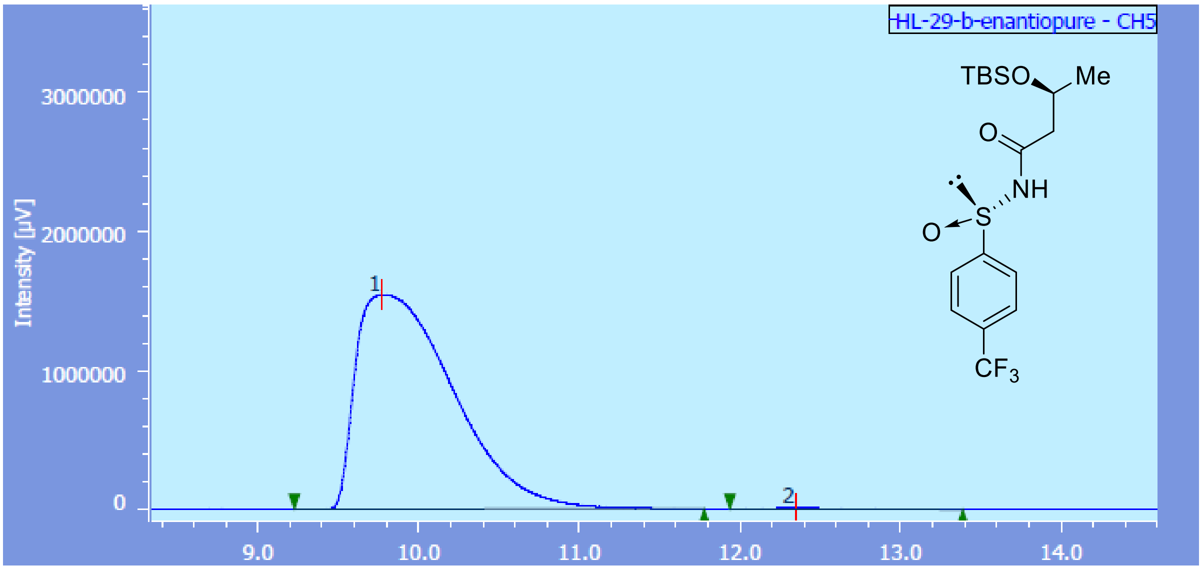

#	Peak Name	CH	tR [min]	Area [μV·sec]	Height [μV]	Area%	Height%	Quantity	NTP	Resolution	Symmetry Factor	Warning
1	Unknown	5	9.770	65100596	1540624	99.640	99.549	N/A	1189	2.612	2.406	
2	Unknown	5	12.343	234991	6986	0.360	0.451	N/A	3434	N/A	1.767	

(*S*)-*N*-[(4-Methoxyphenyl)sulfinyl]butyramide (1n)

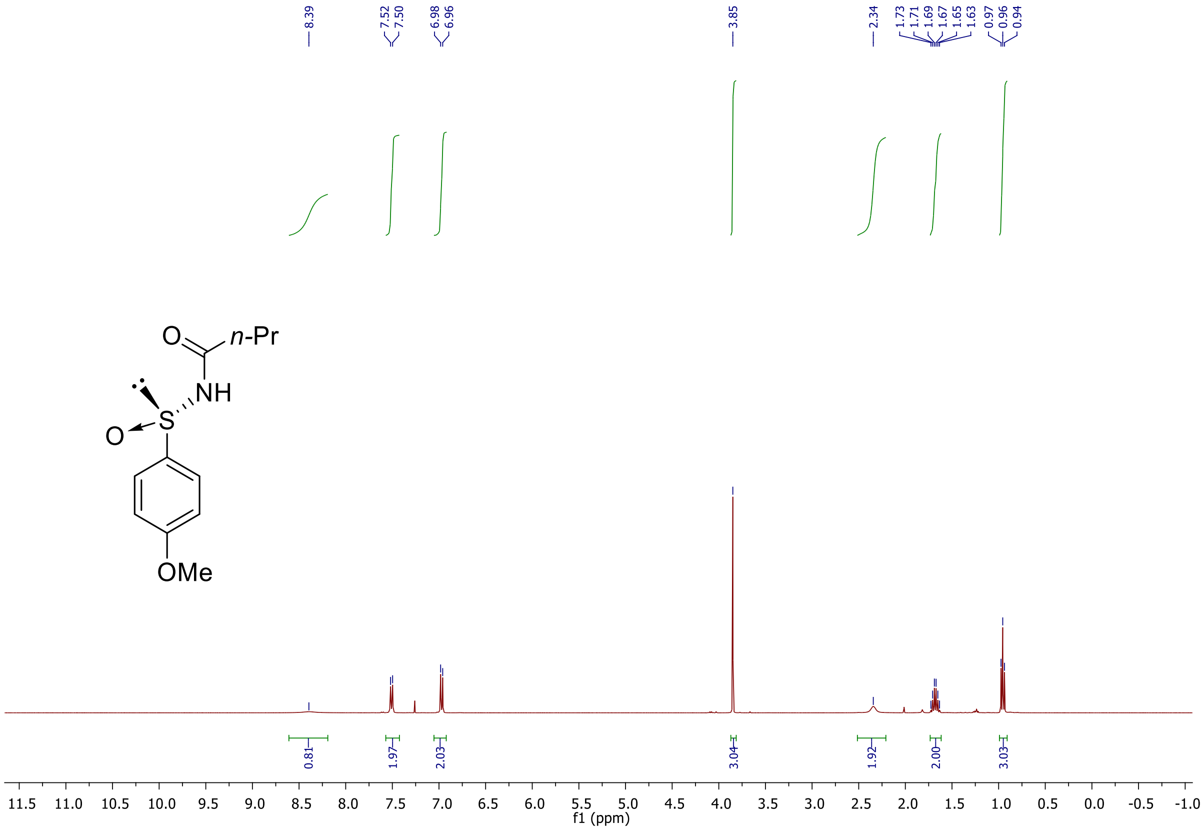

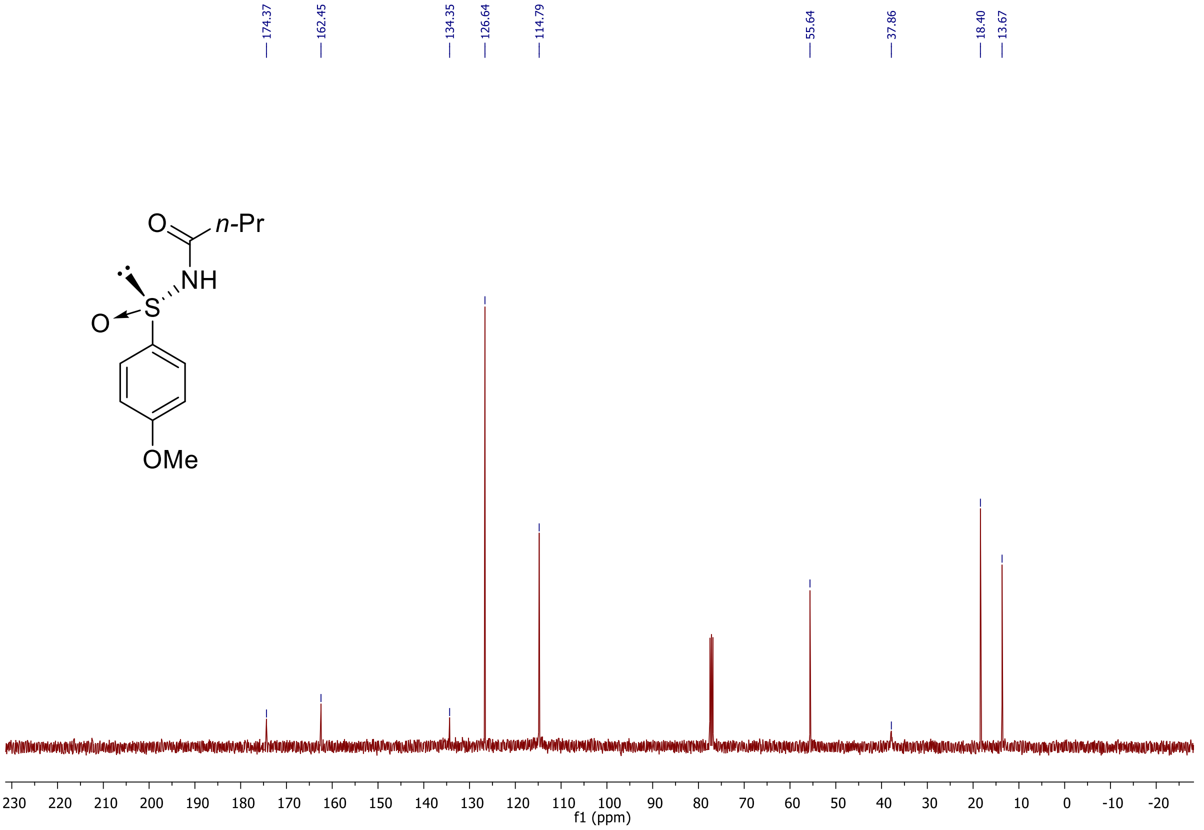

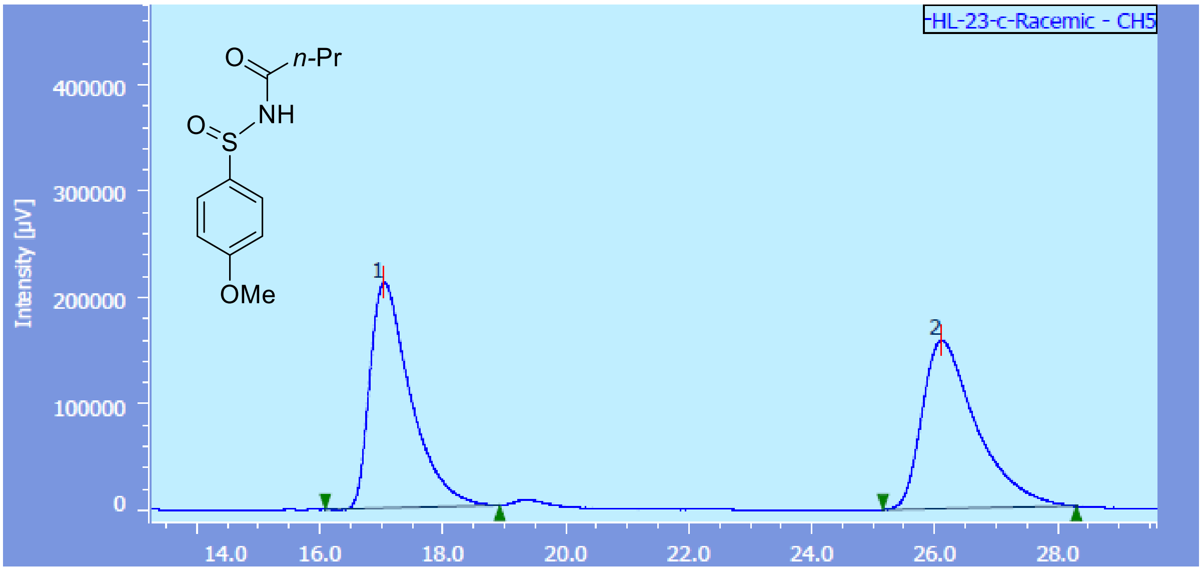

#	Peak Name	CH	tR [min]	Area [µV·sec]	Height [µV]	Area%	Height%	Quantity	NTP	Resolution	Symmetry Factor	Warning
1	Unknown	5	17.047	9757068	212476	50.092	57.338	N/A	3469	6.749	1.791	
2	Unknown	5	26.113	9721053	158093	49.908	42.662	N/A	4623	N/A	1.708	

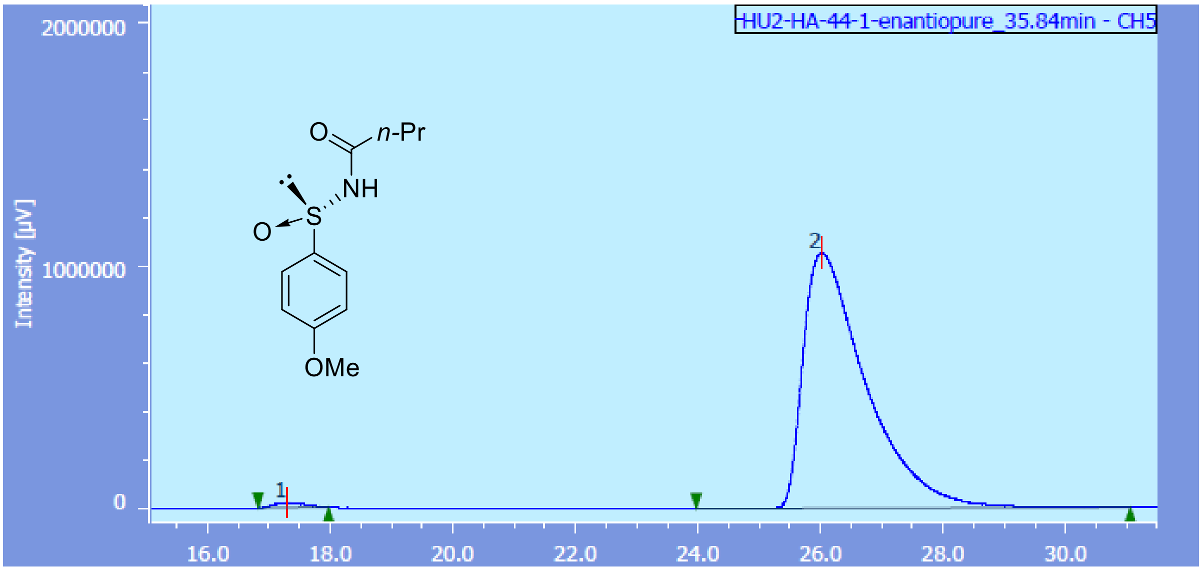

#	Peak Name	CH	tR [min]	Area [µV·sec]	Height [µV]	Area%	Height%	Quantity	NTP	Resolution	Symmetry Factor	Warning
1	Unknown	5	17.300	721161	19943	0.955	1.861	N/A	4713	6.296	1.210	
2	Unknown	5	26.017	74796744	1051852	99.045	98.139	N/A	3463	N/A	2.259	

(*S*)-3-[(*tert*-Butyldimethylsilyl)oxy]-*N*-[(*S*)-(3-methoxyphenyl)sulfinyl]butanamide (1o)

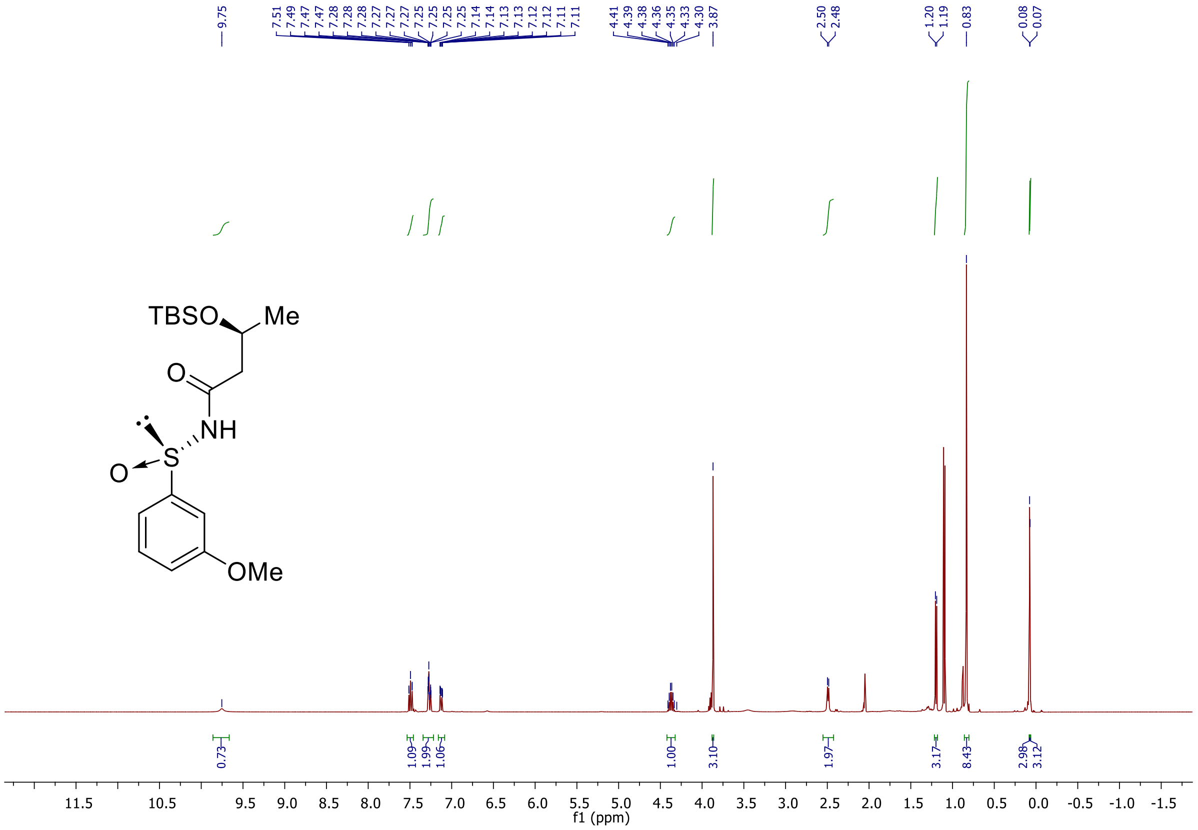

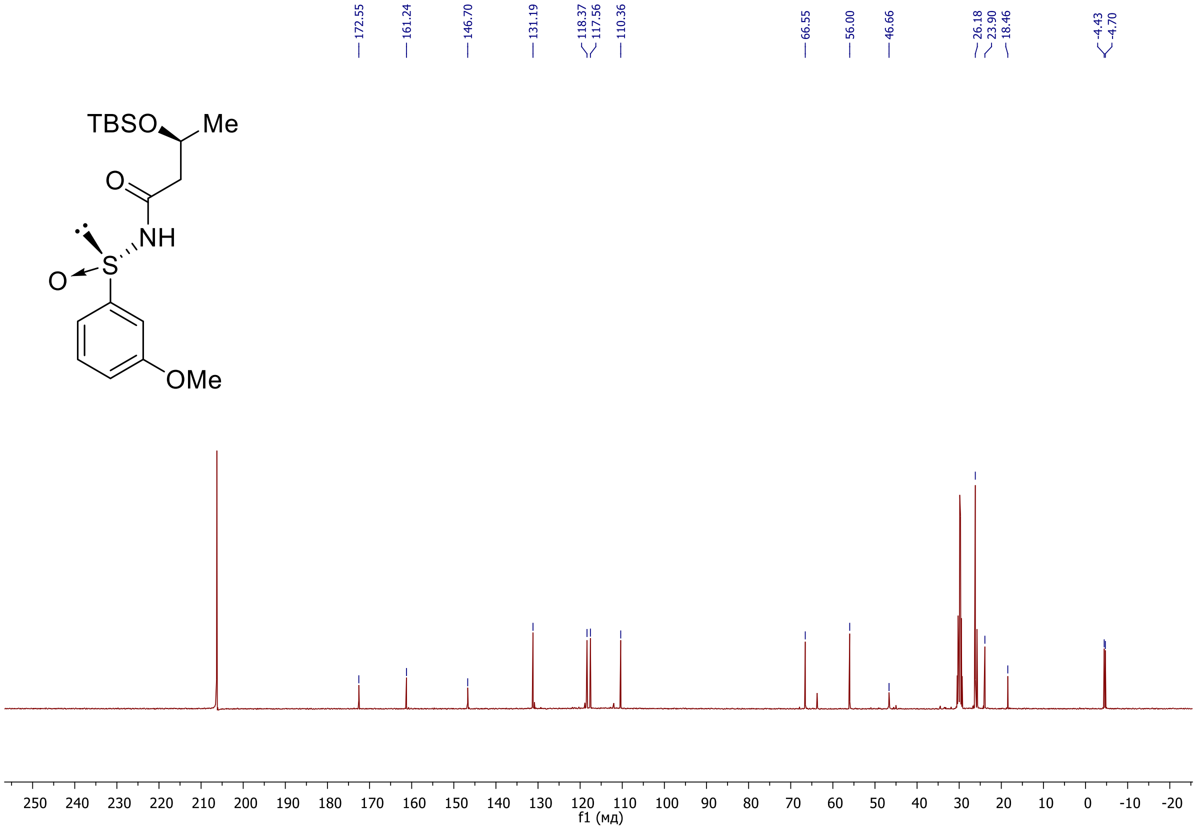

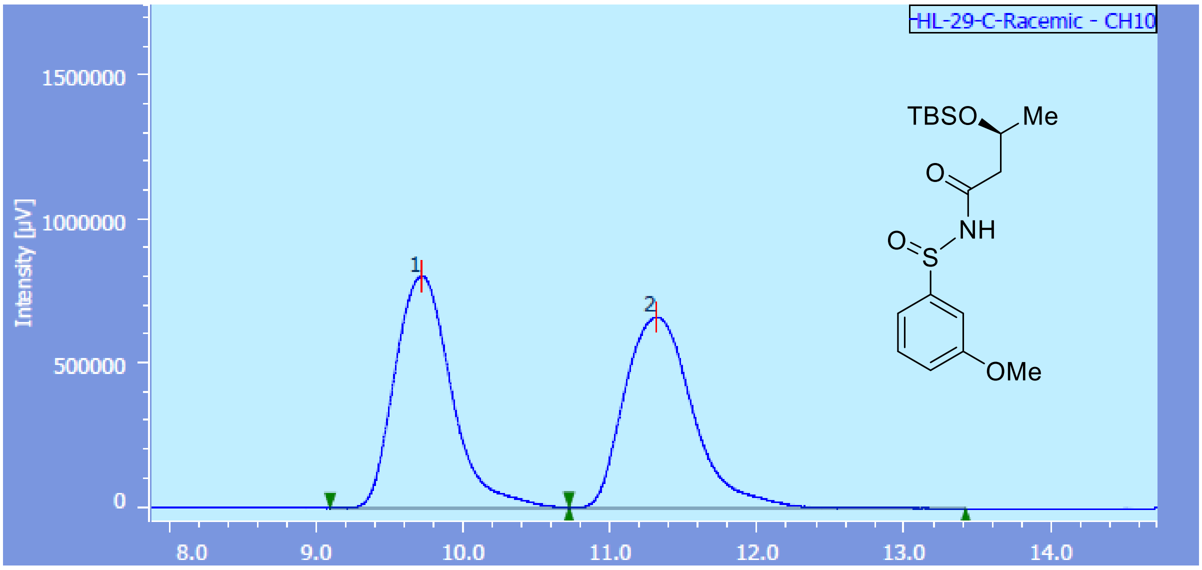

#	Peak Name	CH	tR [min]	Area [µV·sec]	Height [µV]	Area%	Height%	Quantity	NTP	Resolution	Symmetry Factor	Warning
1	Unknown	10	9.717	21653594	806050	50.036	54.826	N/A	3124	2.078	1.338	
2	Unknown	10	11.317	21622439	664151	49.964	45.174	N/A	2847	N/A	1.371	

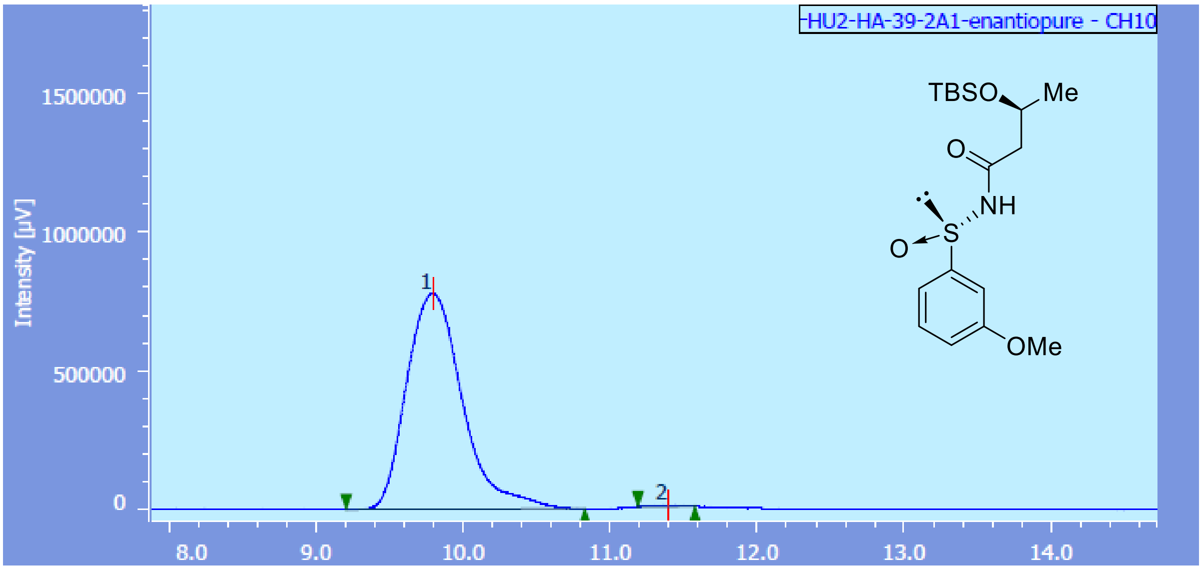

#	Peak Name	CH	tR [min]	Area [µV·sec]	Height [µV]	Area%	Height%	Quantity	NTP	Resolution	Symmetry Factor	Warning
1	Unknown	10	9.793	20563691	778199	99.664	99.409	N/A	3295	2.834	1.401	
2	Unknown	10	11.393	69268	4624	0.336	0.591	N/A	10274	N/A	0.950	

(*S*)-3-[(*tert*-Butyldimethylsilyl)oxy]-*N*-[(*S*)-(3-bromophenyl))sulfinyl]butanamide (1p)

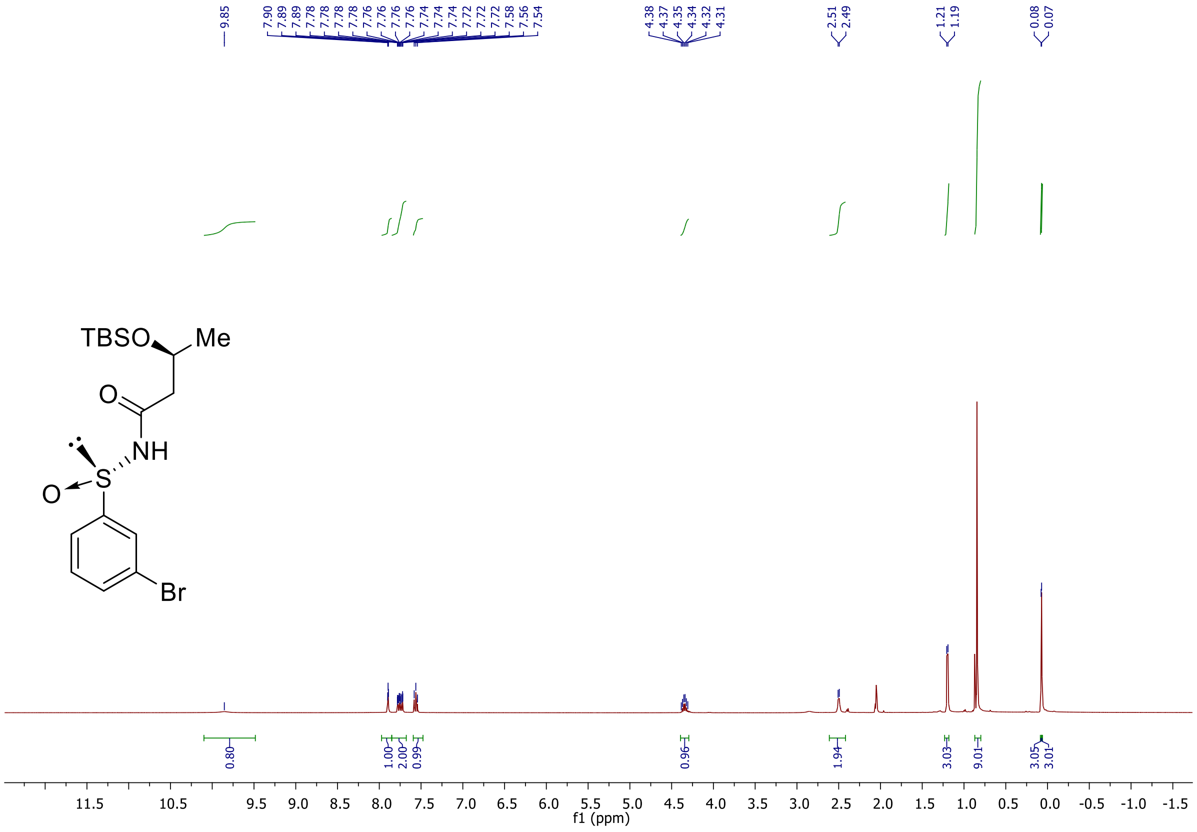

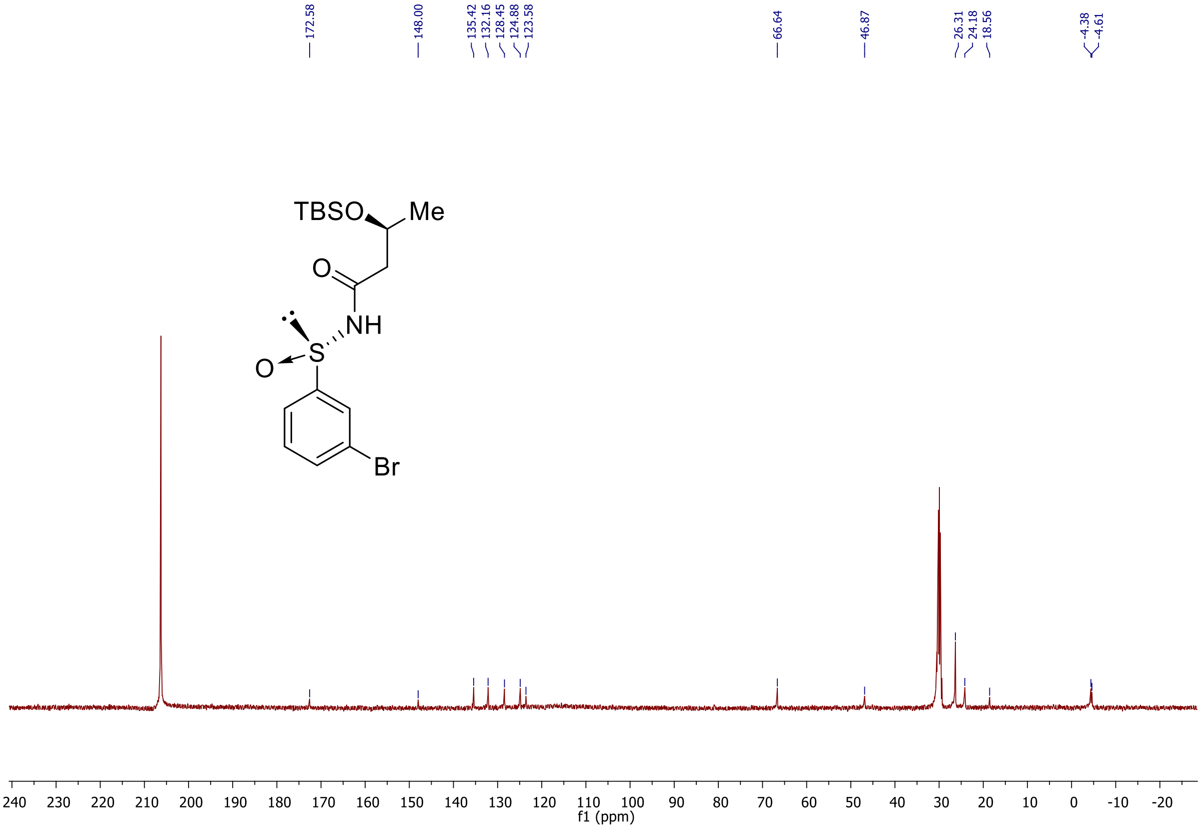

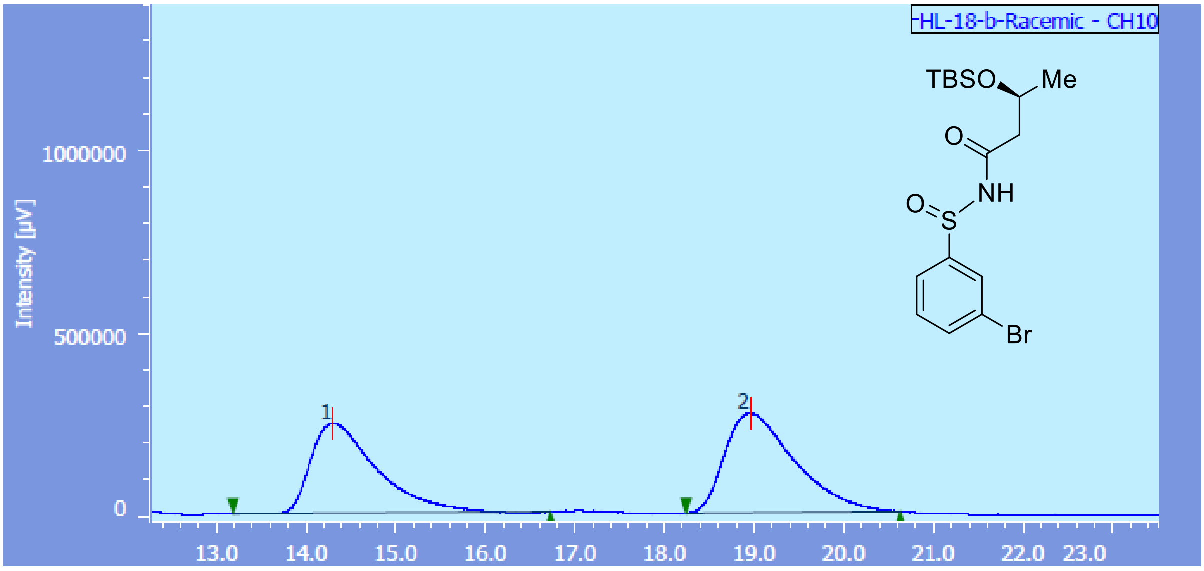

#	Peak Name	CH	tR [min]	Area [μV·sec]	Height [μV]	Area%	Height%	Quantity	NTP	Resolution	Symmetry Factor	Warning
1	Unknown	10	14.300	12544939	245464	45.825	47.386	N/A	1989	3.464	1.976	
2	Unknown	10	18.953	14830957	272549	54.175	52.614	N/A	2884	N/A	1.666	

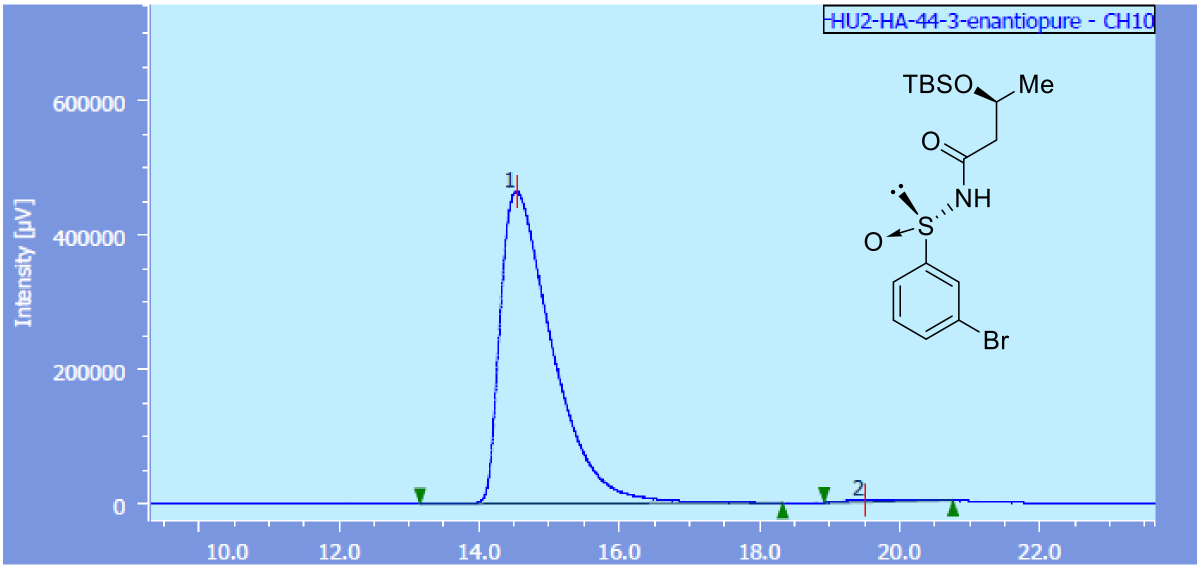

#	Peak Name	CH	tR [min]	Area [μV·sec]	Height [μV]	Area%	Height%	Quantity	NTP	Resolution	Symmetry Factor	Warning
1	Unknown	10	14.533	24051251	464838	99.225	99.297	N/A	1957	3.714	2.120	
2	Unknown	10	19.503	187736	3289	0.775	0.703	N/A	3246	N/A	1.618	

(*S*)-*N*-(*o*-Tolylsulfinyl)butyramide (1q)

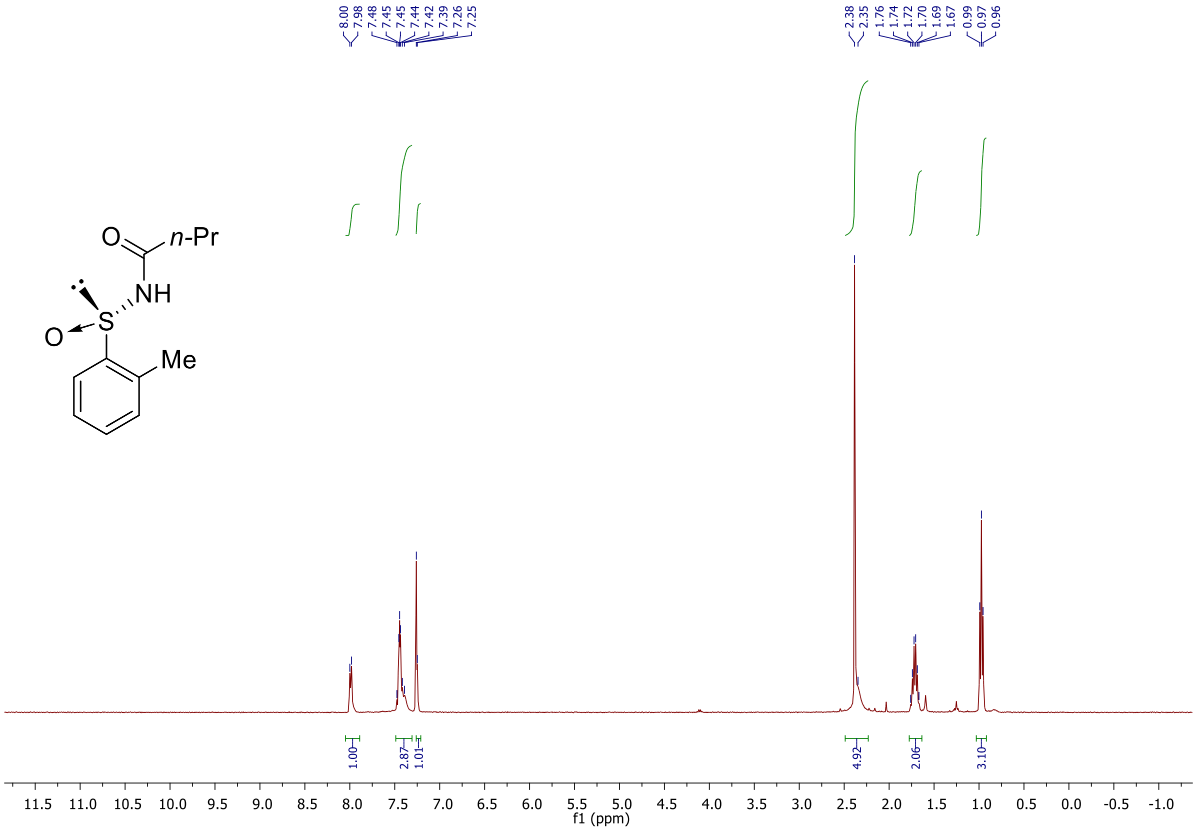

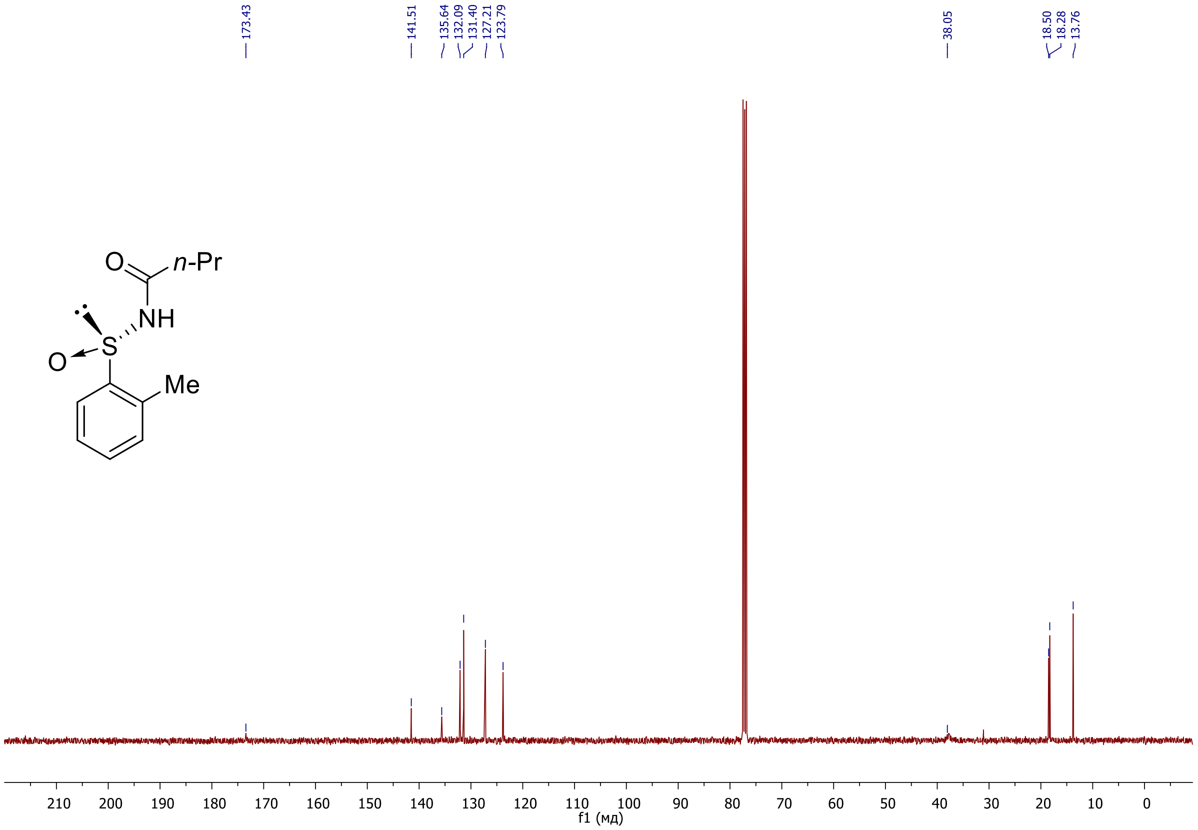

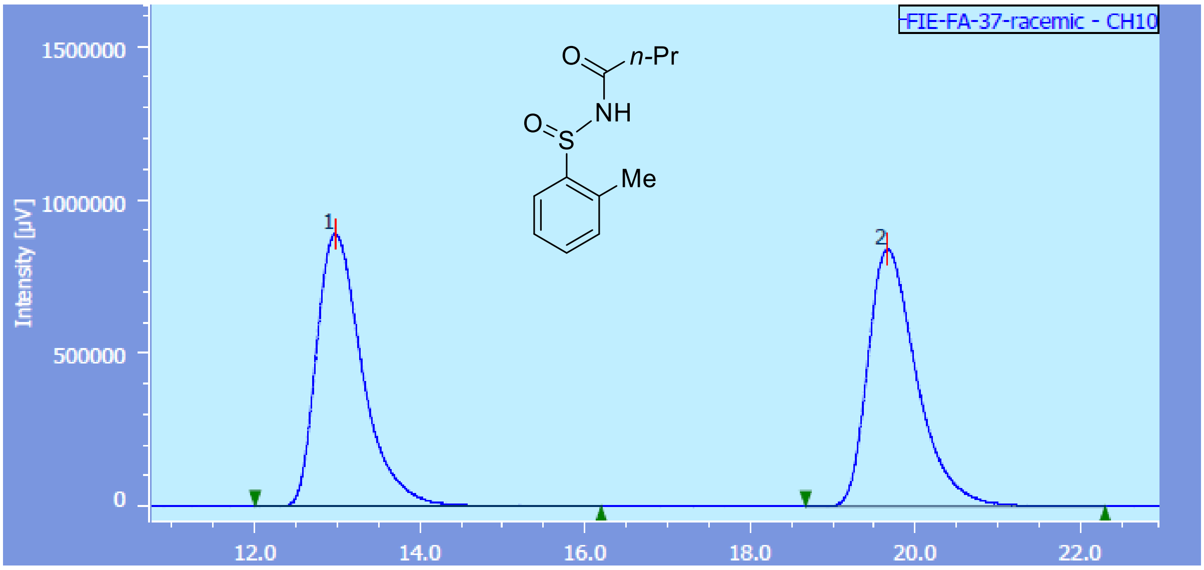

#	Peak Name	CH	tR [min]	Area [µV·sec]	Height [µV]	Area%	Height%	Quantity	NTP	Resolution	Symmetry Factor	Warning
1	Unknown	10	12.977	34507364	888165	49.992	51.433	N/A	2786	6.602	1.502	
2	Unknown	10	19.663	34518665	838675	50.008	48.567	N/A	5637	N/A	1.473	

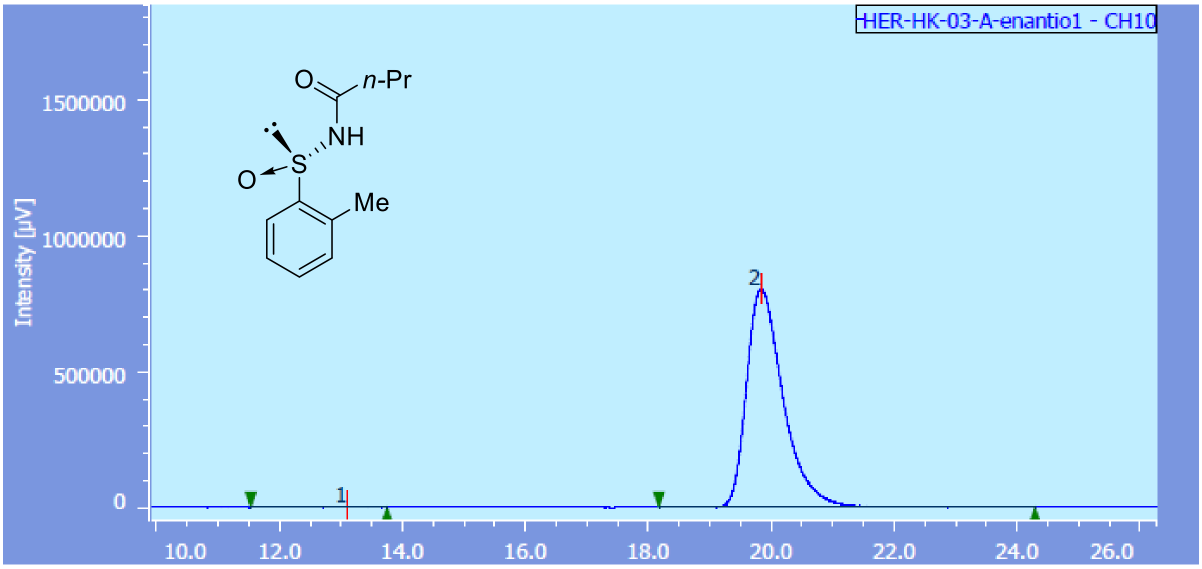

#	Peak Name	CH	tR [min]	Area [µV·sec]	Height [µV]	Area%	Height%	Quantity	NTP	Resolution	Symmetry Factor	Warning
1	Unknown	10	13.110	118324	3765	0.353	0.468	N/A	3736	7.075	1.196	
2	Unknown	10	19.833	33395775	801458	99.647	99.532	N/A	5733	N/A	1.504	

(*S*)-*N*-[(*o*-Bromophenyl)sulfinyl]butyramide (1r)

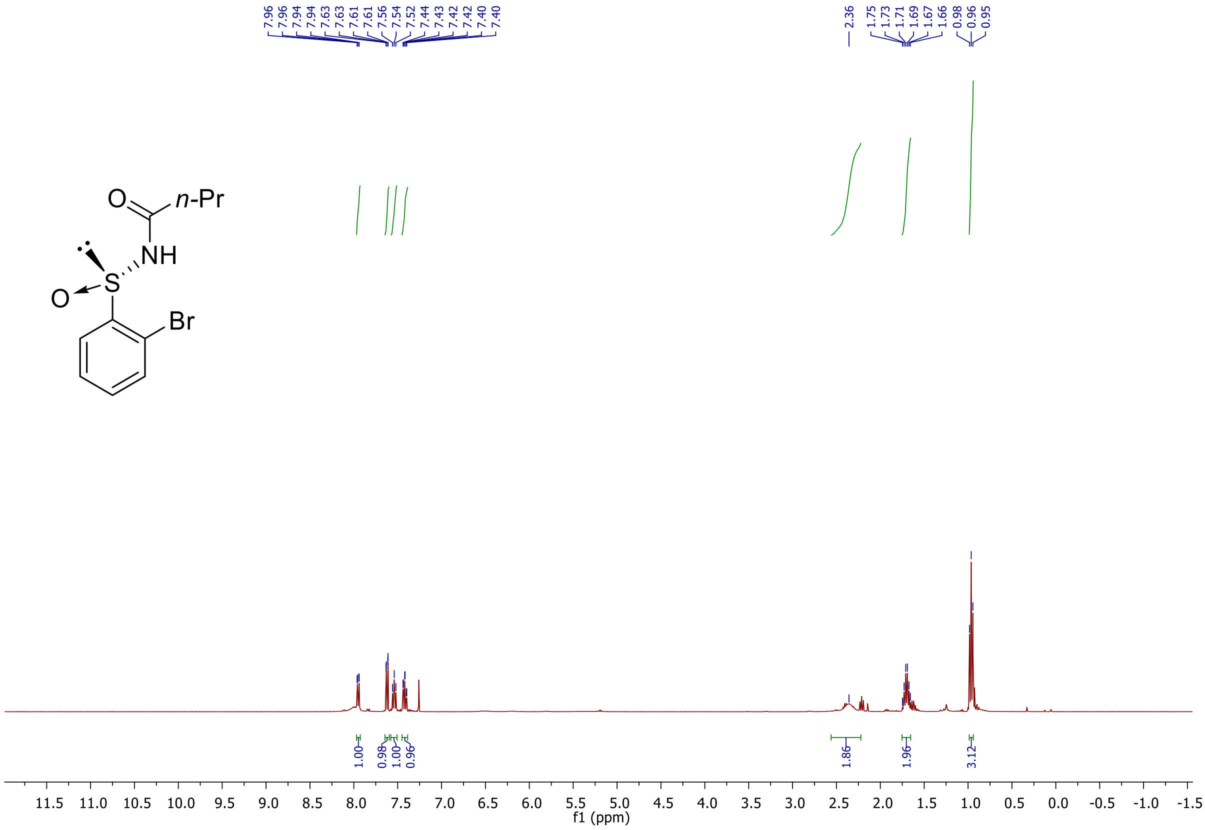

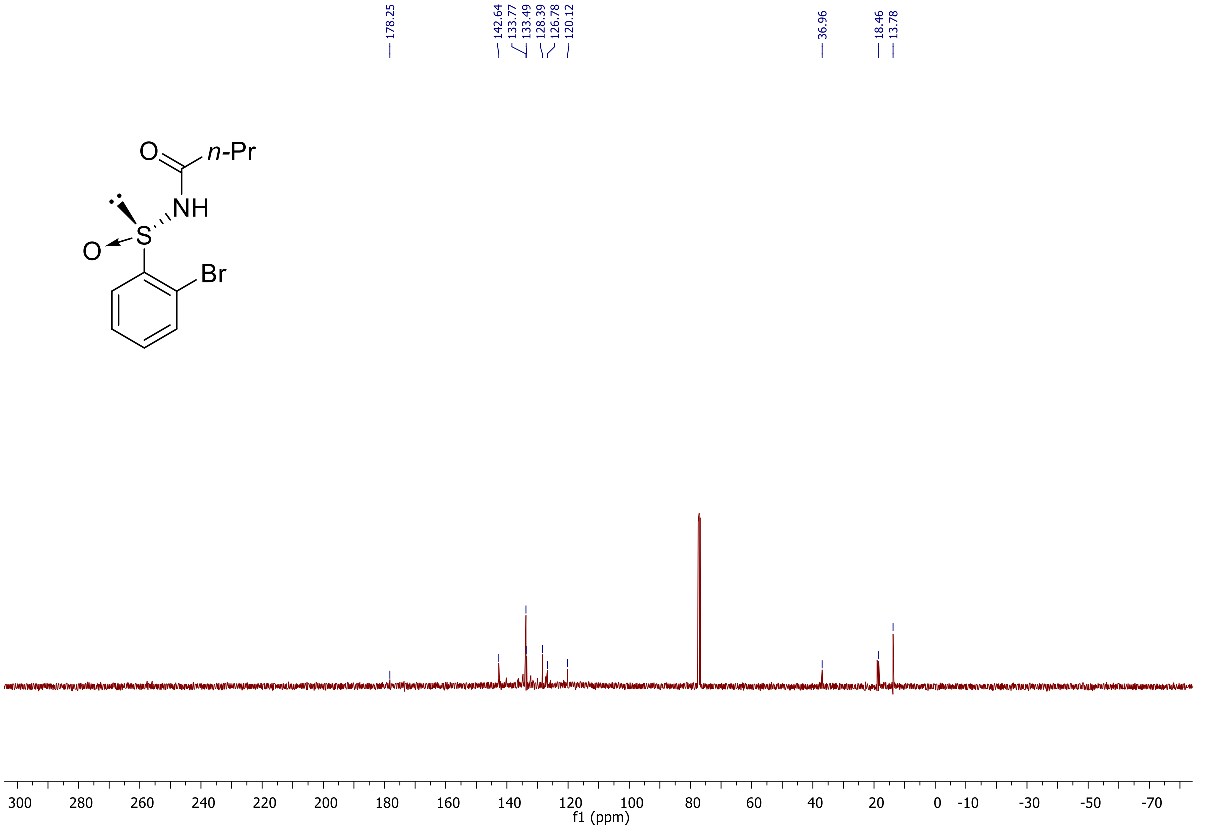

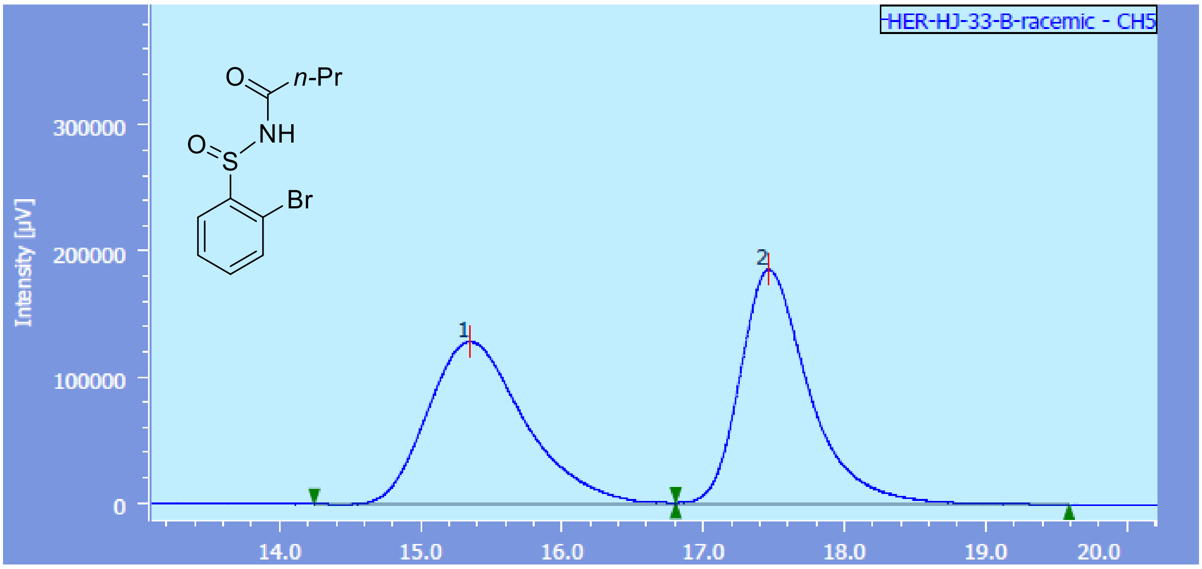

#	Peak Name	CH	tR [min]	Area [µV·sec]	Height [µV]	Area%	Height%	Quantity	NTP	Resolution	Symmetry Factor	Warning
1	Unknown	5	15.350	6193981	129108	49.331	40.934	N/A	2466	2.039	1.321	
2	Unknown	5	17.463	6361853	186298	50.669	59.066	N/A	6876	N/A	1.433	

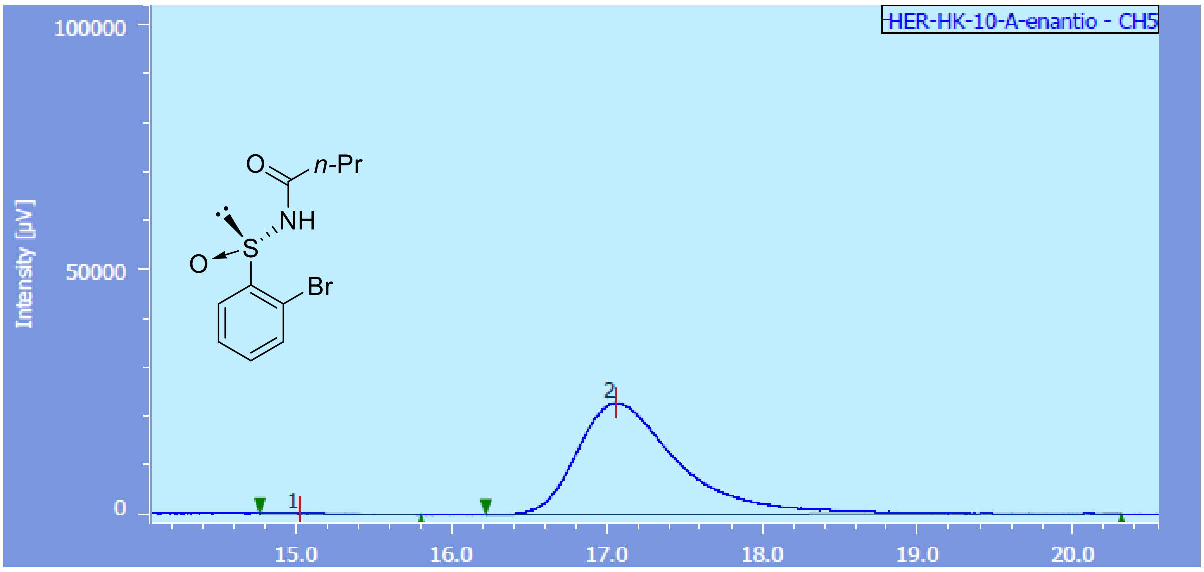

#	Peak Name	CH	tR [min]	Area [µV·sec]	Height [µV]	Area%	Height%	Quantity	NTP	Resolution	Symmetry Factor	Warning
1	Unknown	5	15.023	2466	103	0.236	0.448	N/A	3853	1.969	2.574	
2	Unknown	5	17.060	1043474	22847	99.764	99.552	N/A	3803	N/A	1.642	

***(S)*-3-[(*tert*-Butyldimethylsilyl)oxy]-*N*-[(*S*)-(5-chlorothiophen-2-yl)sulfinyl]butanamide (1s)**

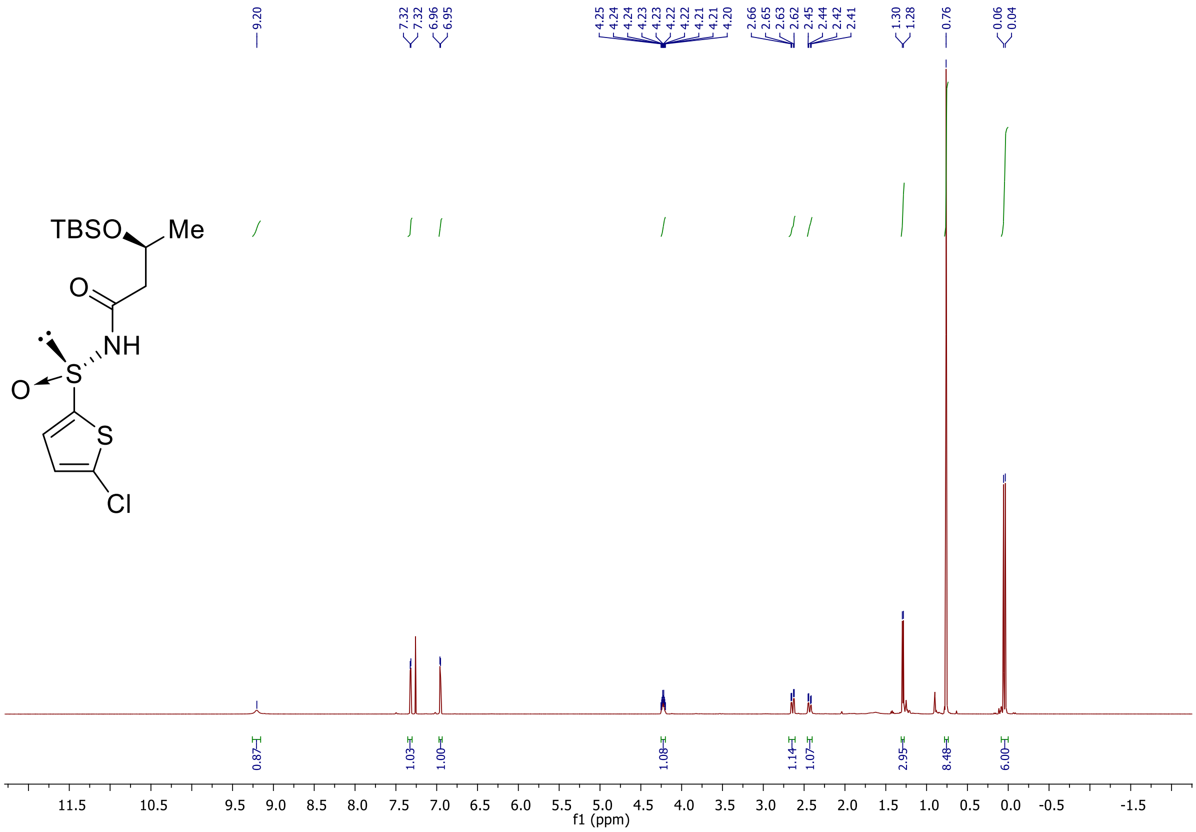

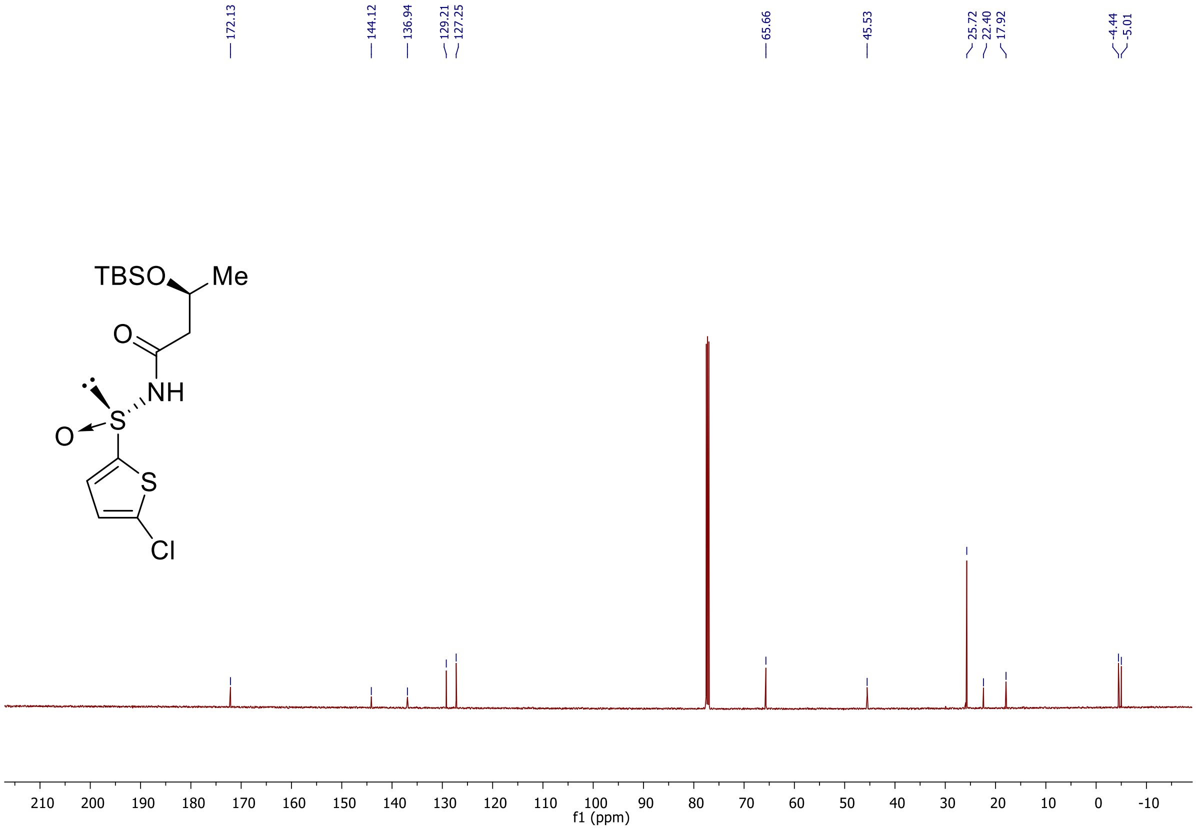

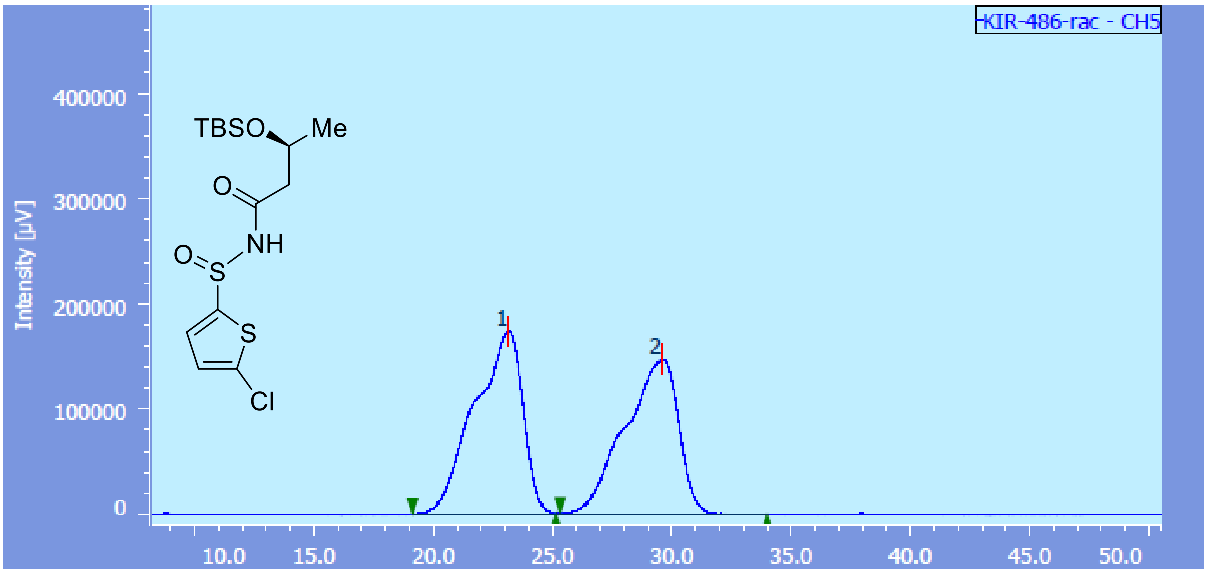

#	Peak Name	CH	tR [min]	Area [µV·sec]	Height [µV]	Area%	Height%	Quantity	NTP	Resolution	Symmetry Factor	Warning
1	Unknown	5	23.147	24597076	174235	51.603	54.176	N/A	485	1.478	0.732	
2	Unknown	5	29.593	23068771	147371	48.397	45.824	N/A	680	N/A	0.737	

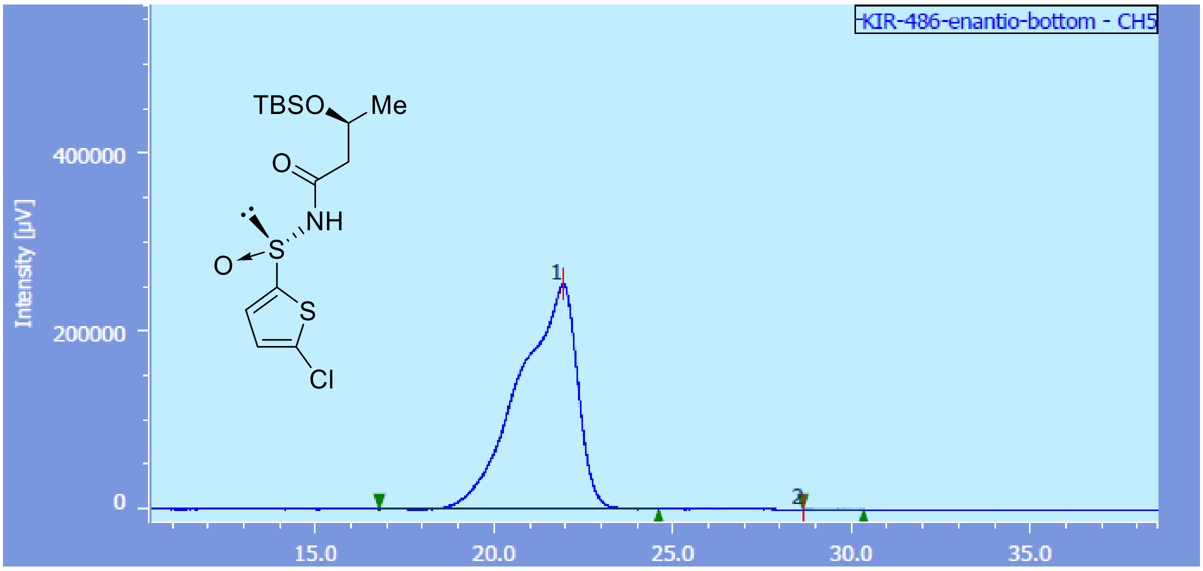

#	Peak Name	CH	tR [min]	Area [µV·sec]	Height [µV]	Area%	Height%	Quantity	NTP	Resolution	Symmetry Factor	Warning
1	Unknown	5	21.933	28419074	253856	99.990	99.975	N/A	754	2.263	0.681	
2	Unknown	5	28.673	2966	63	0.010	0.025	N/A	1706	N/A	25.947	

(*R*)-3-[(*tert*-Butyldimethylsilyl)oxy]-*N*-[(*S*)-(5-chlorothiophen-2-yl)sulfinyl]butanamide (1s')

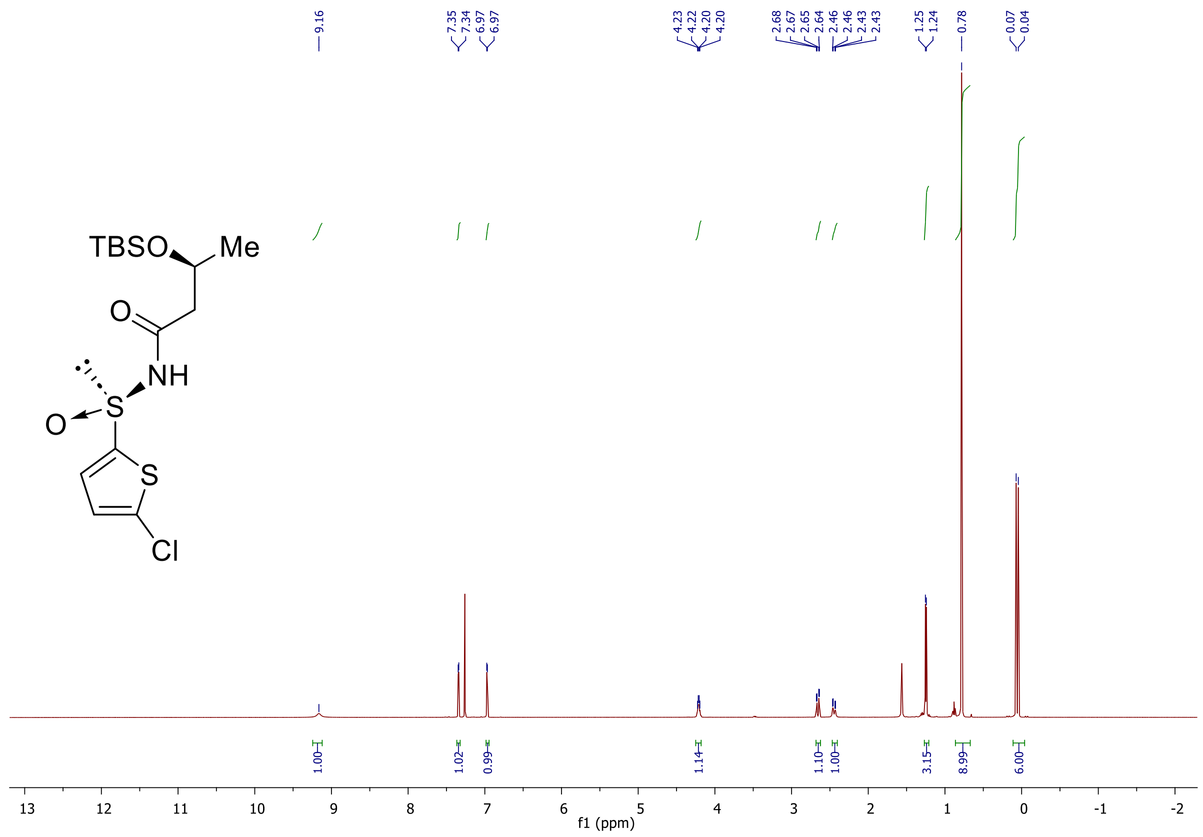

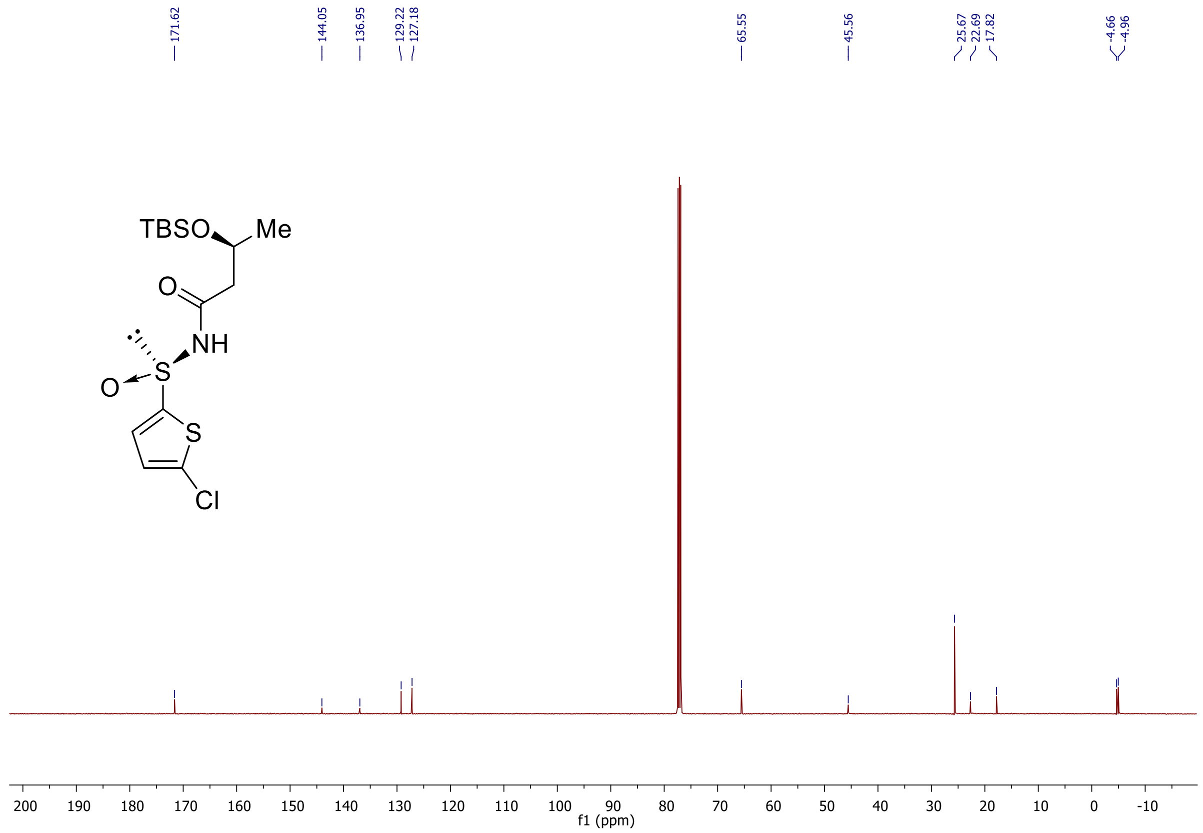

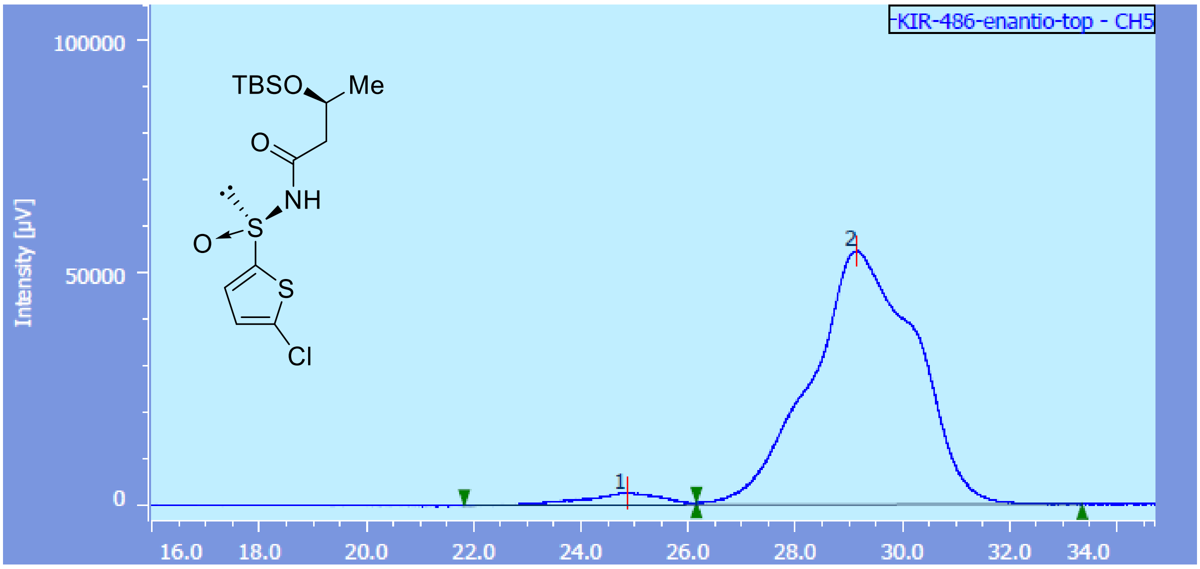

#	Peak Name	CH	tR [min]	Area [µV·sec]	Height [µV]	Area%	Height%	Quantity	NTP	Resolution	Symmetry Factor	Warning
1	Unknown	5	24.850	261582	2546	3.395	4.459	N/A	1325	1.340	N/A	
2	Unknown	5	29.157	7442258	54554	96.605	95.541	N/A	986	N/A	0.988	

(S)-3-[(*tert*-Butyldimethylsilyl)oxy]-*N*-[(*S*)-(thiophen-2-yl)sulfinyl]butanamide (1r)

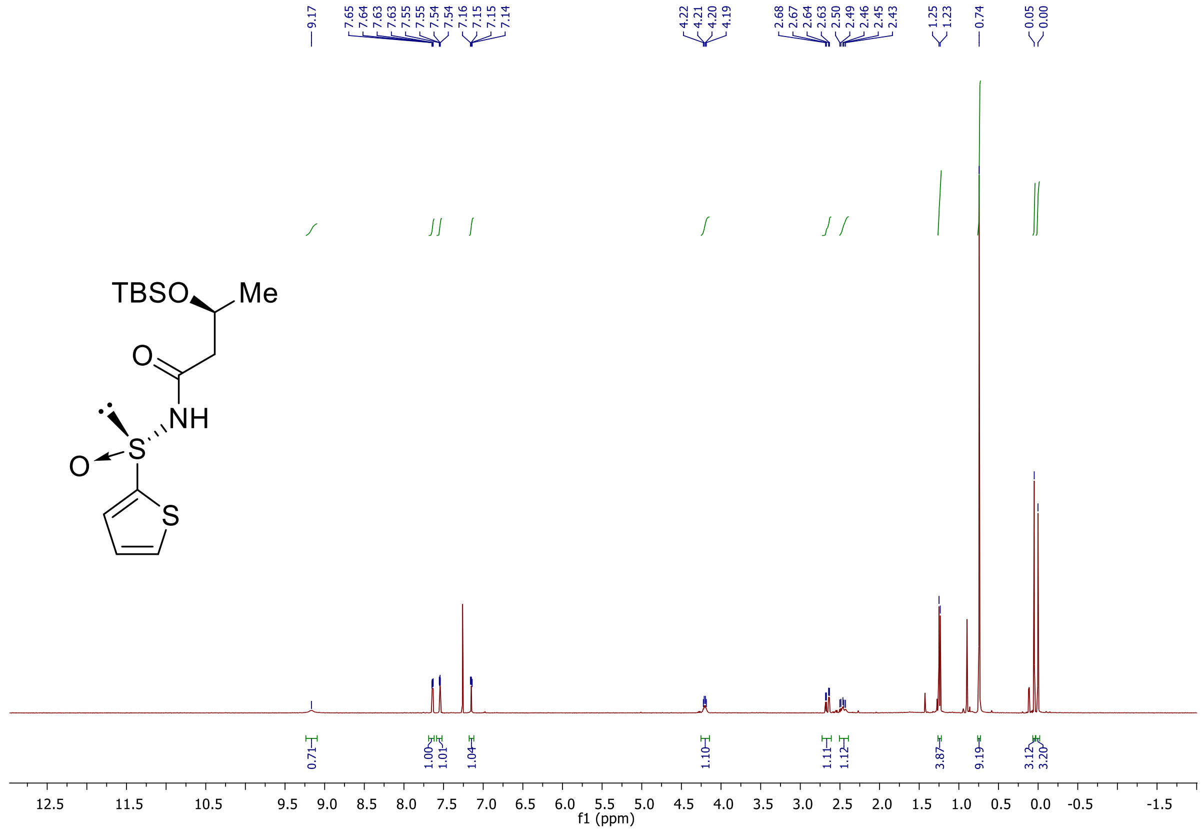

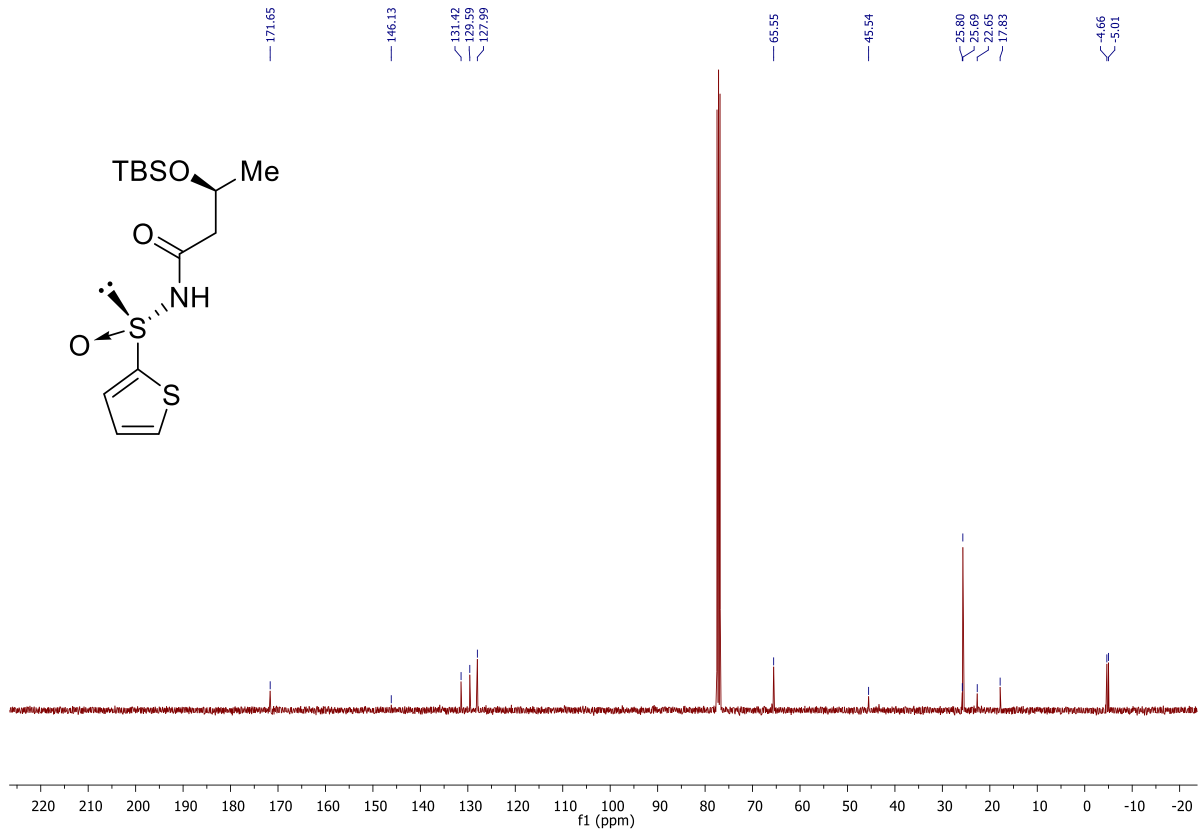

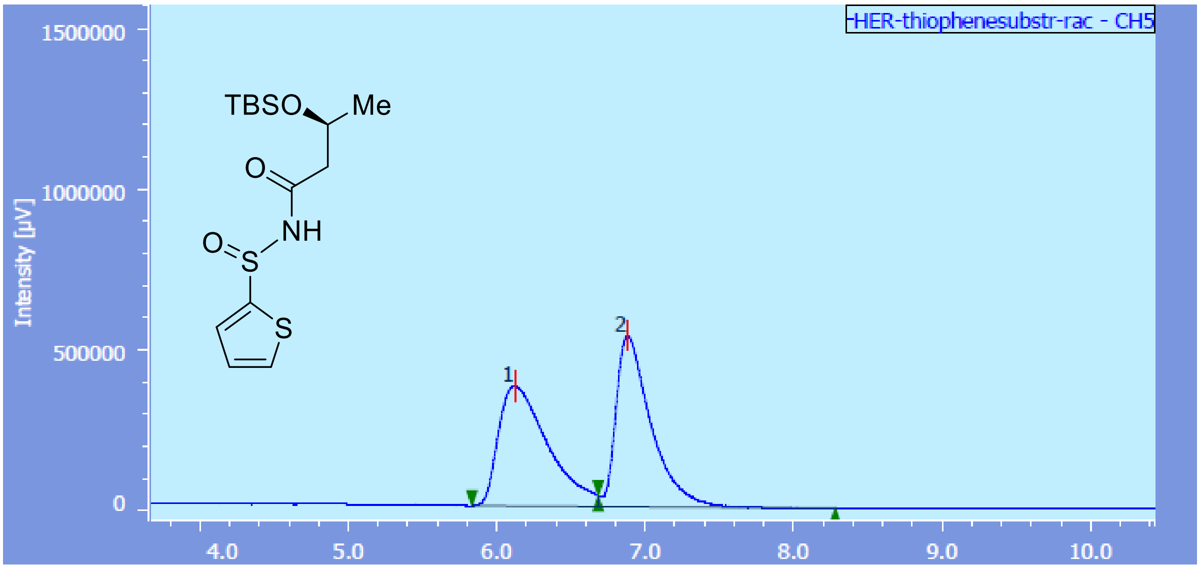

#	Peak Name	CH	tR [min]	Area [µV·sec]	Height [µV]	Area%	Height%	Quantity	NTP	Resolution	Symmetry Factor	Warning
1	Unknown	5	6.120	8610631	372842	49.625	41.225	N/A	1665	1.519	N/A	
2	Unknown	5	6.880	8740785	531572	50.375	58.775	N/A	4651	N/A	N/A	

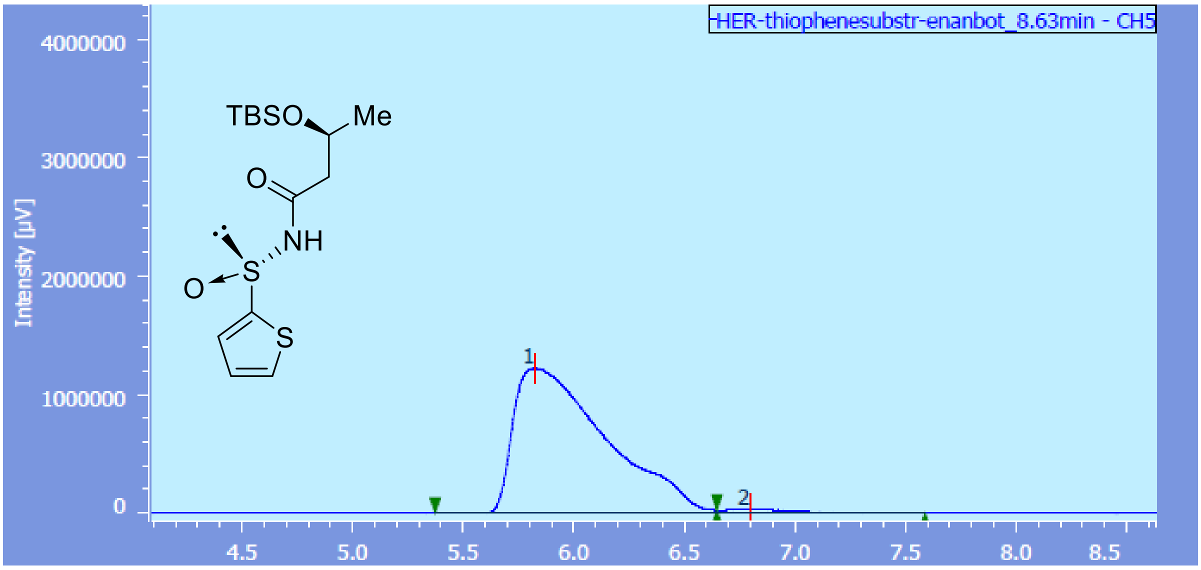

#	Peak Name	CH	tR [min]	Area [µV·sec]	Height [µV]	Area%	Height%	Quantity	NTP	Resolution	Symmetry Factor	Warning
1	Unknown	5	5.827	35506457	1220872	98.042	97.305	N/A	970	N/A	2.587	
2	Unknown	5	6.797	708990	33820	1.958	2.695	N/A	N/A	N/A	N/A	

***(R)*-3-((tert-butyldimethylsilyl)oxy)-*N*-((*S*)-(thiophen-2-yl)sulfinyl)butanamide (1r')**

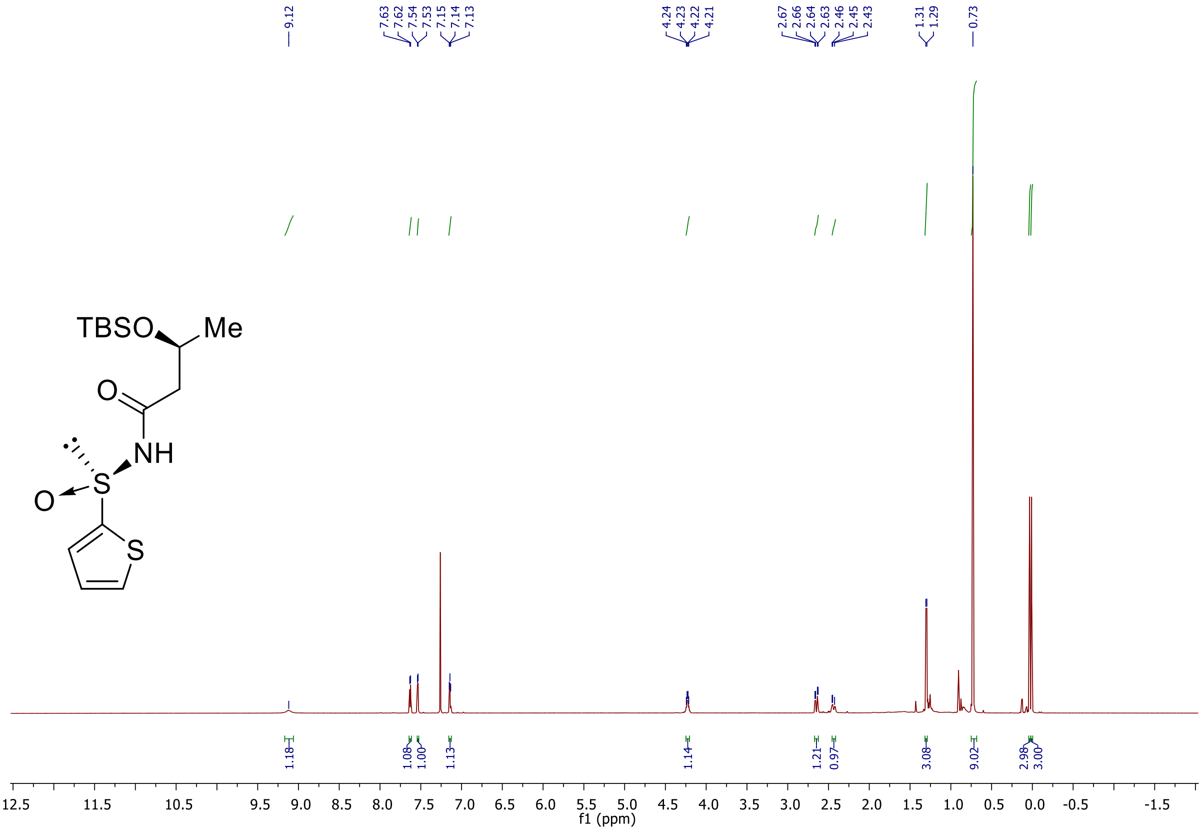

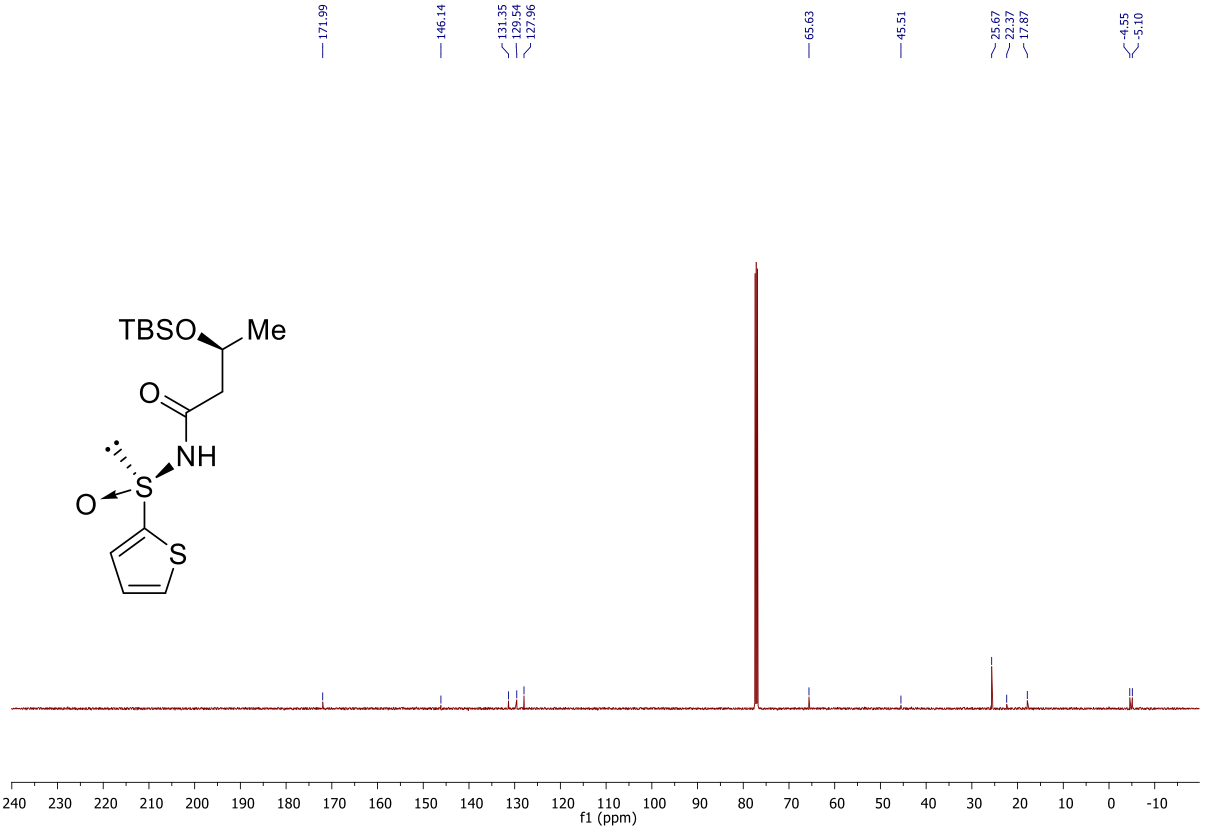

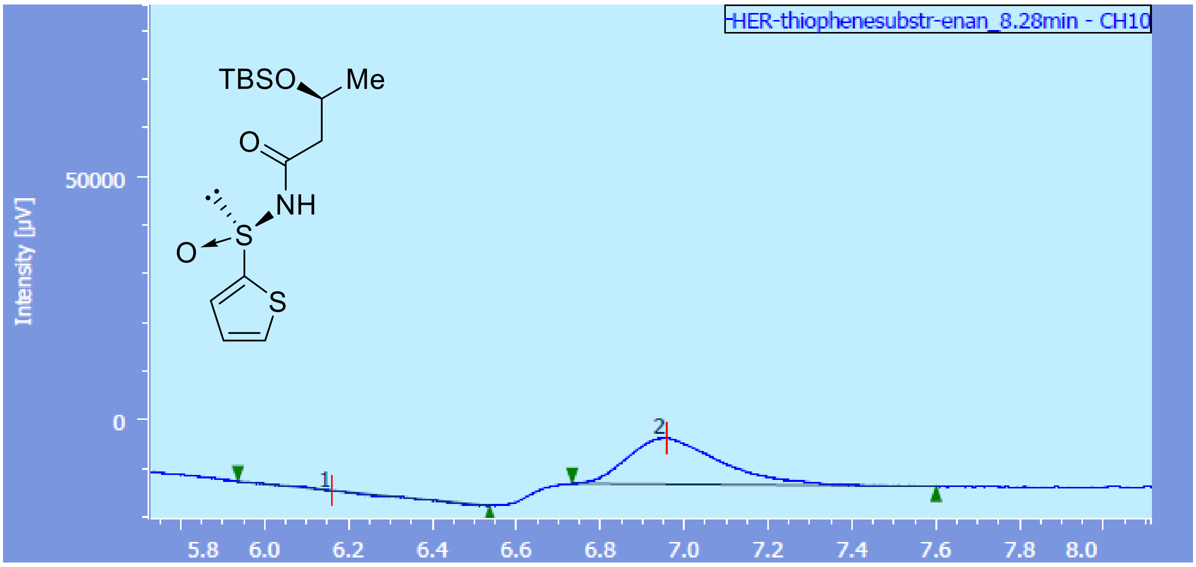

#	Peak Name	CH	tR [min]	Area [µV·sec]	Height [µV]	Area%	Height%	Quantity	NTP	Resolution	Symmetry Factor	Warning
1	Unknown	10	6.160	2943	49	1.977	0.519	N/A	1378	1.513	1.345	
2	Unknown	10	6.957	145940	9476	98.023	99.481	N/A	5029	N/A	1.496	

Tetrabutylammonium butyryl(*p*-tolylsulfinyl)amide (3)

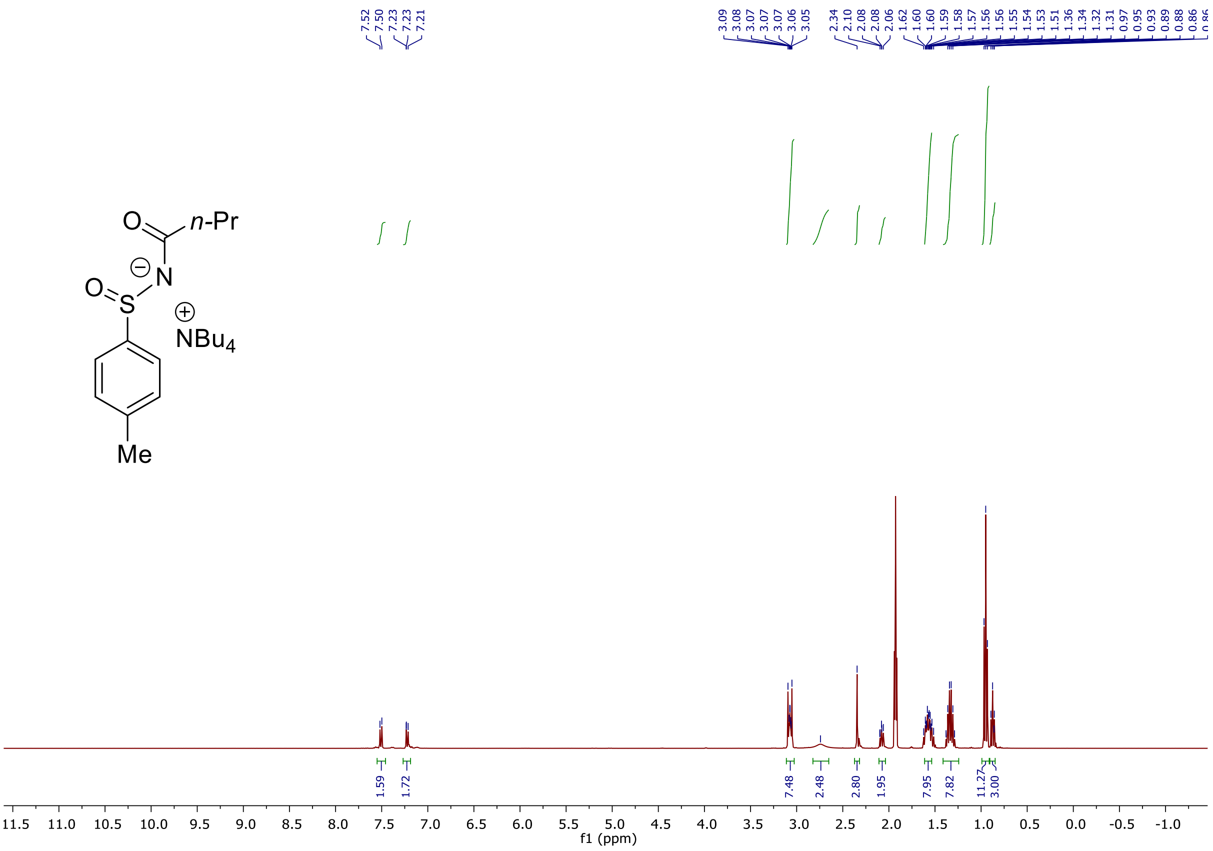

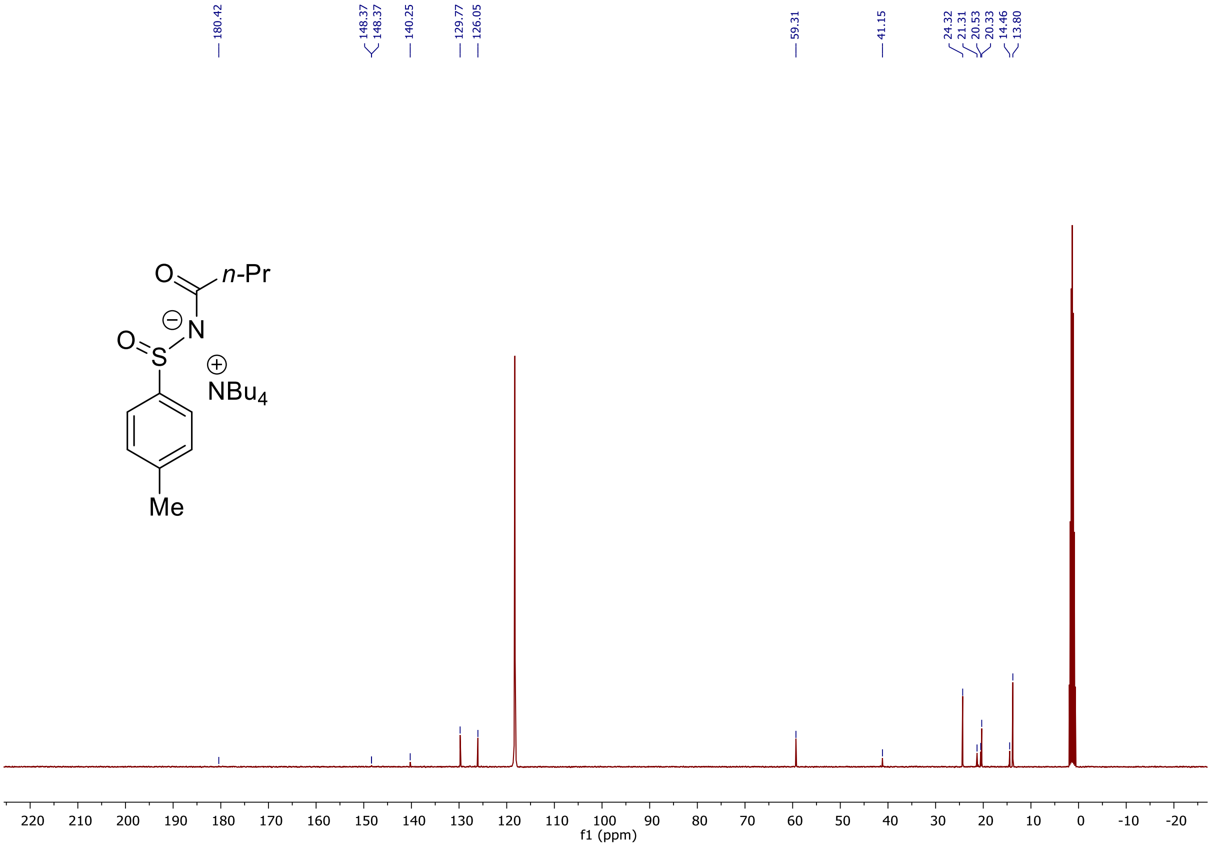

N-[(1*R*,2*R*)-1-(4-Methoxyphenyl)-1-(*p*-tolyl)propan-2-yl]butyramide (2.1)

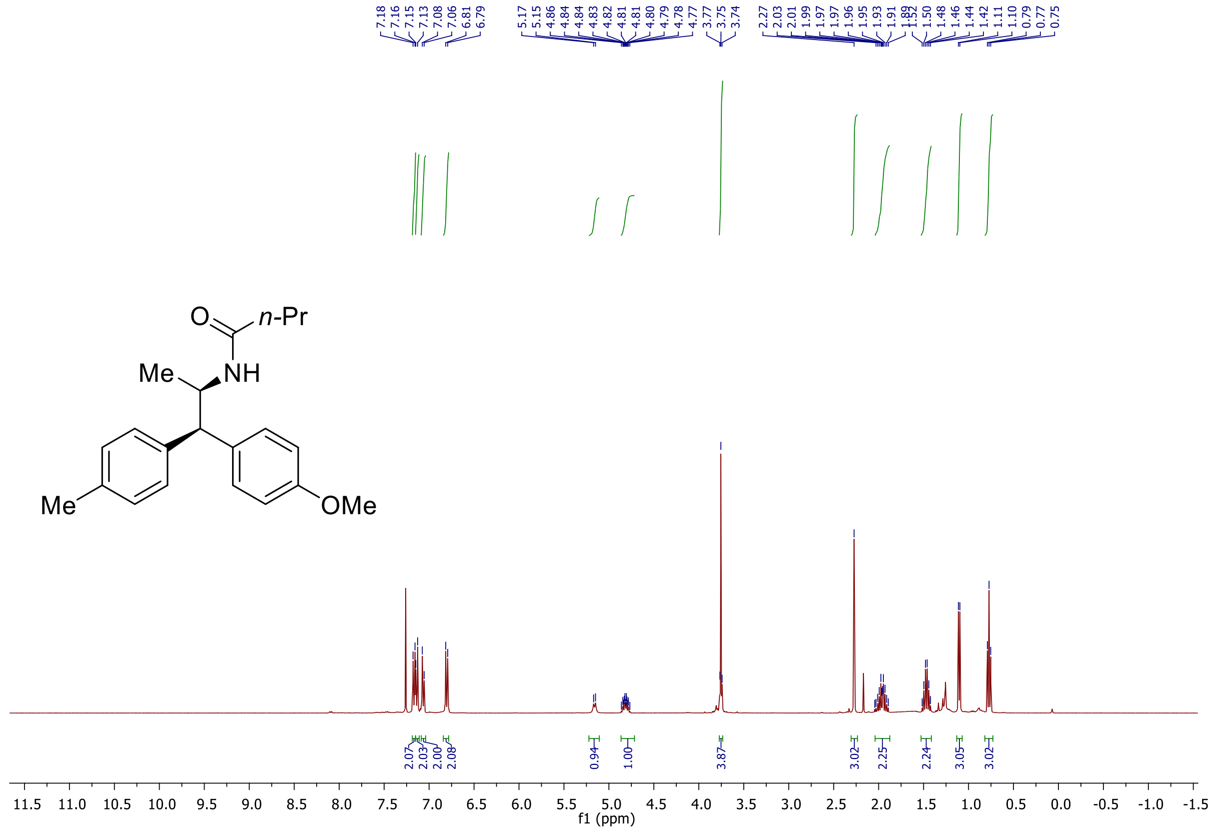

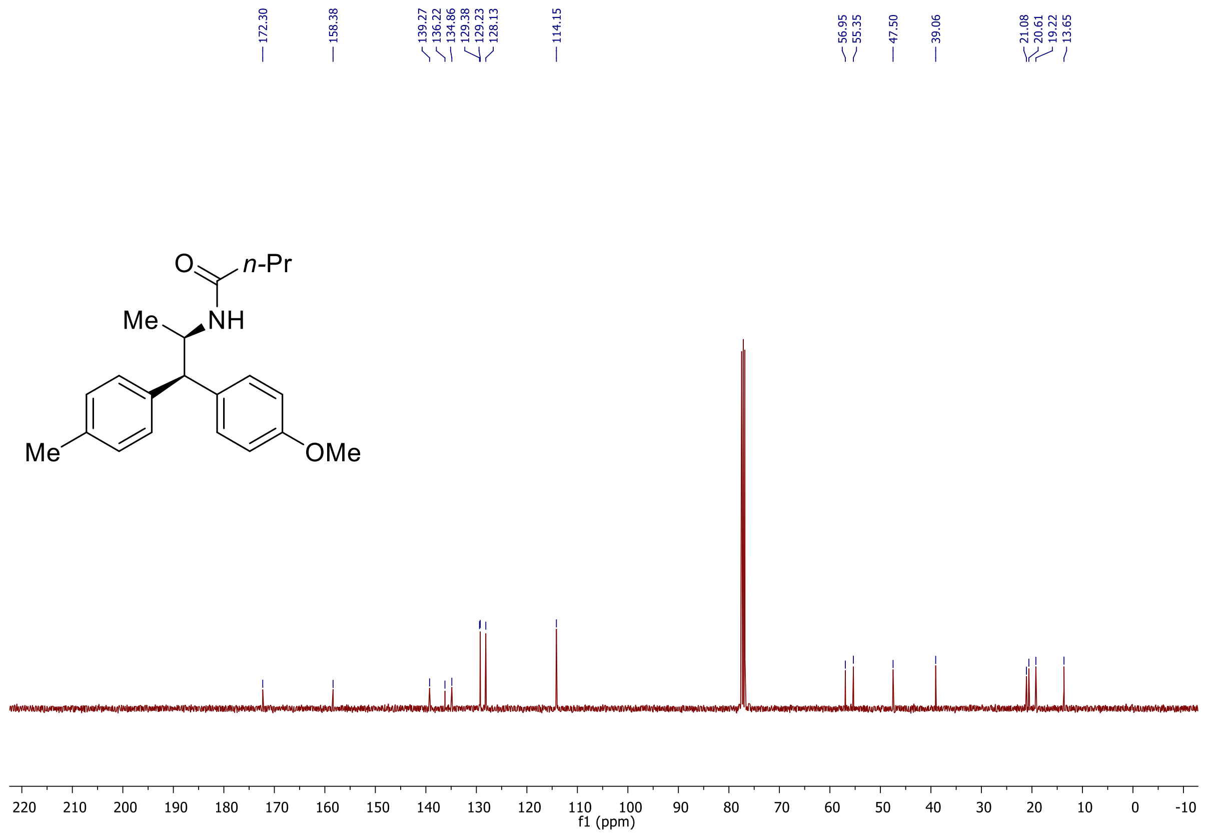

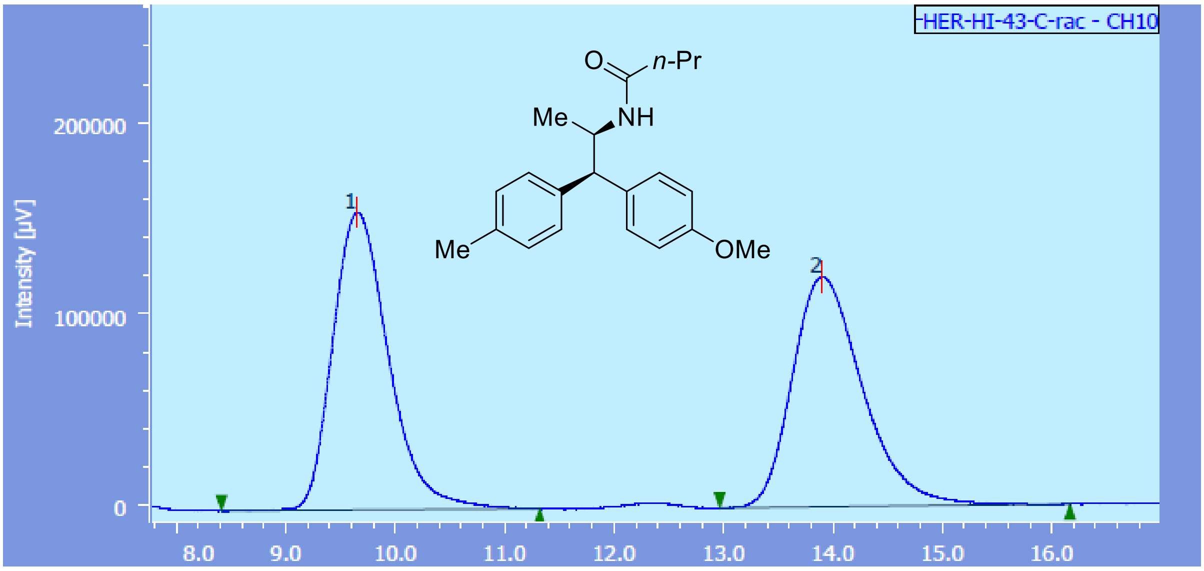

#	Peak Name	CH	tR [min]	Area [µV·sec]	Height [µV]	Area%	Height%	Quantity	NTP	Resolution	Symmetry Factor	Warning
1	Unknown	10	9.653	5527047	155360	50.722	56.384	N/A	1772	4.121	1.321	
2	Unknown	10	13.903	5369671	120178	49.278	43.616	N/A	2335	N/A	1.333	

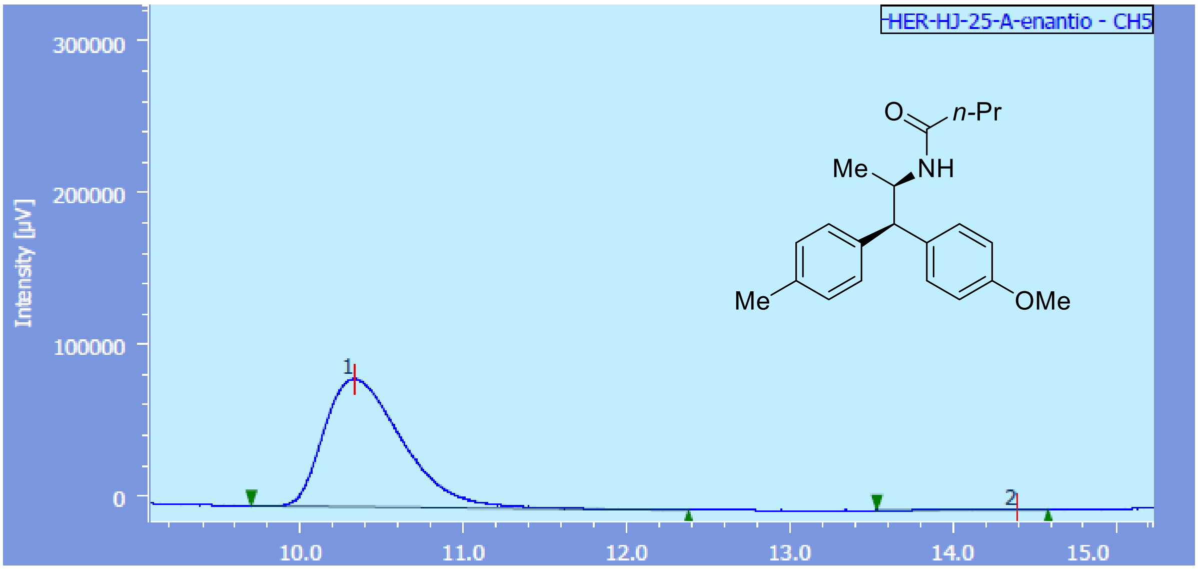

#	Peak Name	CH	tR [min]	Area [µV·sec]	Height [µV]	Area%	Height%	Quantity	NTP	Resolution	Symmetry Factor	Warning
1	Unknown	5	10.333	2817808	84419	99.830	99.683	N/A	2234	5.779	1.517	
2	Unknown	5	14.393	4802	268	0.170	0.317	N/A	11618	N/A	0.781	

N-[(1*R*,2*R*)-1-(4-Methoxyphenyl)-1-(*p*-tolyl)propan-2-yl]butyramide (2.1) (1 mmol scale)

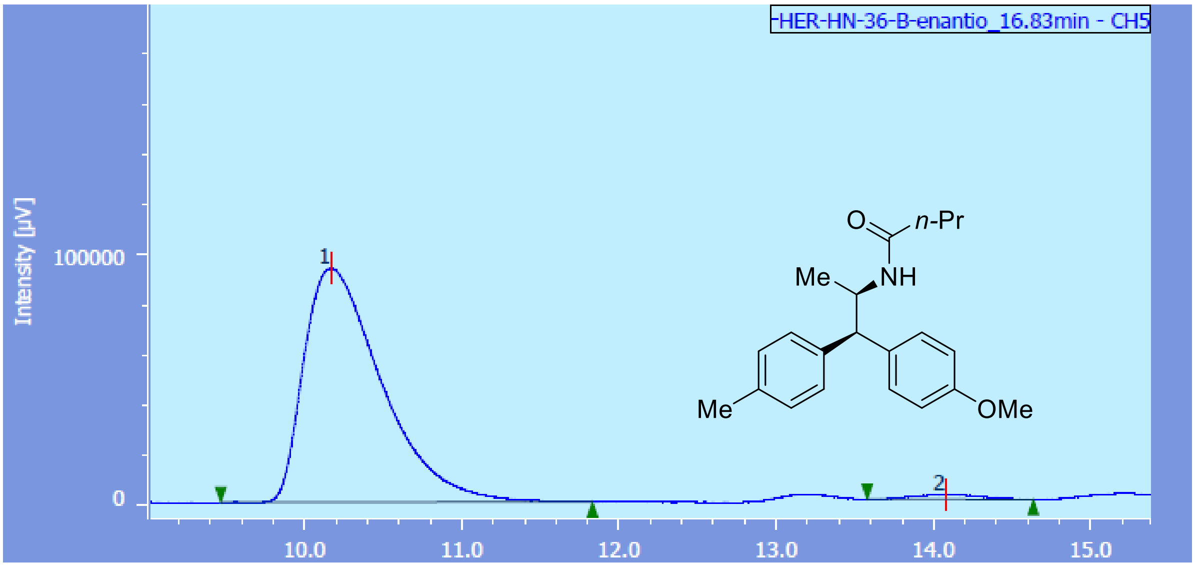

#	Peak Name	CH	tR [min]	Area [µV·sec]	Height [µV]	Area%	Height%	Quantity	NTP	Resolution	Symmetry Factor	Warning
1	Unknown	5	10.170	3202469	93054	98.265	98.067	N/A	2080	4.484	1.805	
2	Unknown	5	14.077	56559	1834	1.735	1.933	N/A	4335	N/A	1.168	

N-[(1*S*,2*S*)-1-(4-Methoxyphenyl)-1-(*p*-tolyl)propan-2-yl]butyramide (2a')

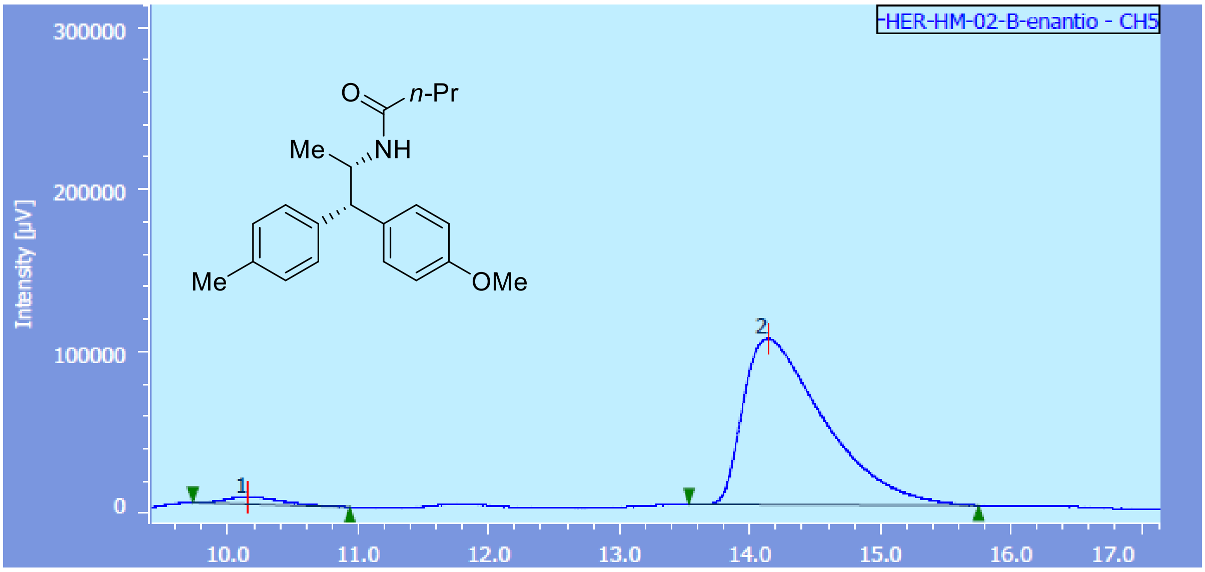

#	Peak Name	CH	tR [min]	Area [µV·sec]	Height [µV]	Area%	Height%	Quantity	NTP	Resolution	Symmetry Factor	Warning
1	Unknown	5	10.153	132361	4497	2.919	4.211	N/A	2814	4.251	1.493	
2	Unknown	5	14.137	4402548	102296	97.081	95.789	N/A	2578	N/A	2.130	

2-(4-Methoxyphenyl)-*N*-[(1*R*,2*R*)-1-(4-methoxyphenyl)-1-(*p*-tolyl)propan-2-yl]acetamide (2.2)

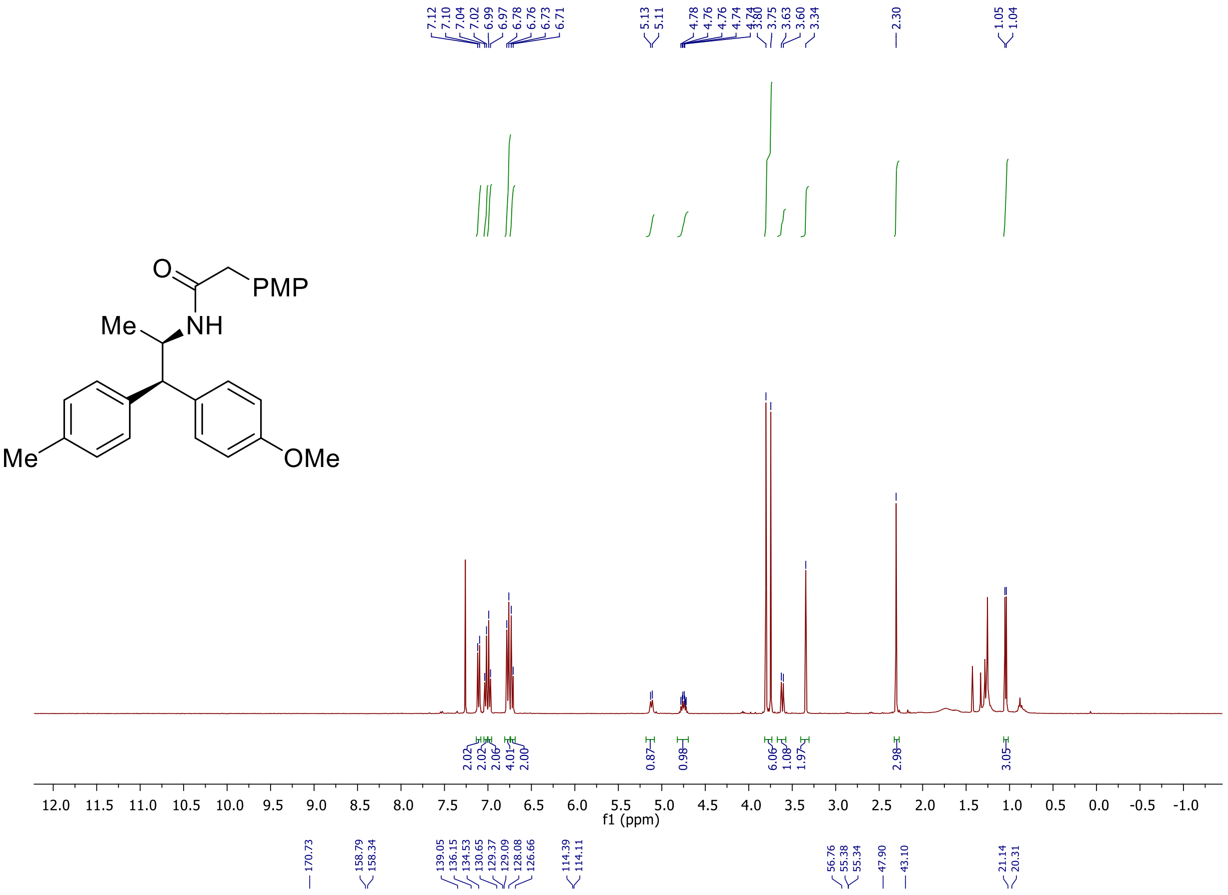

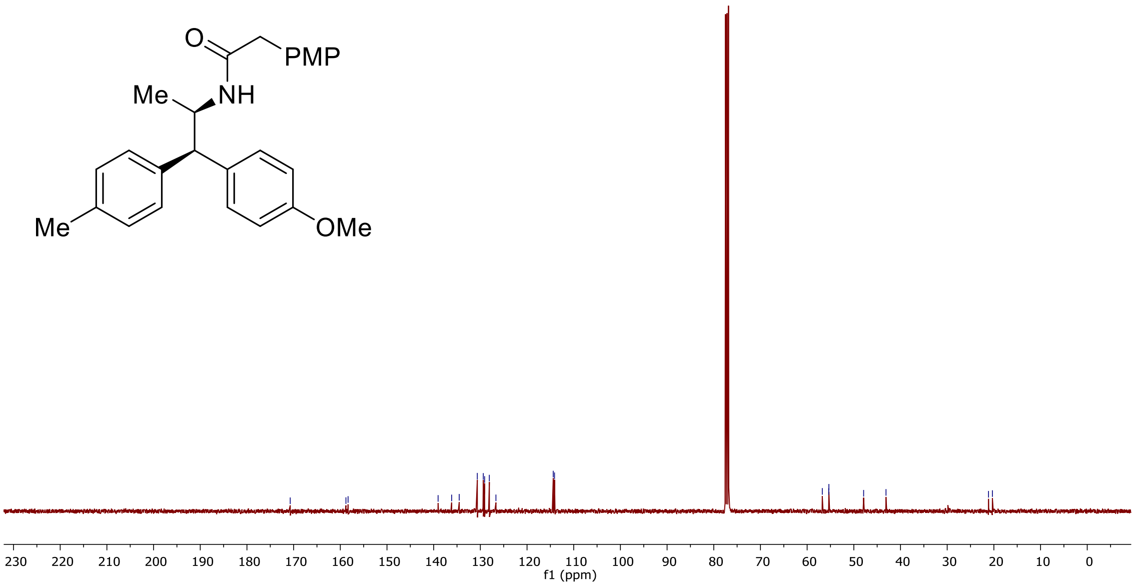

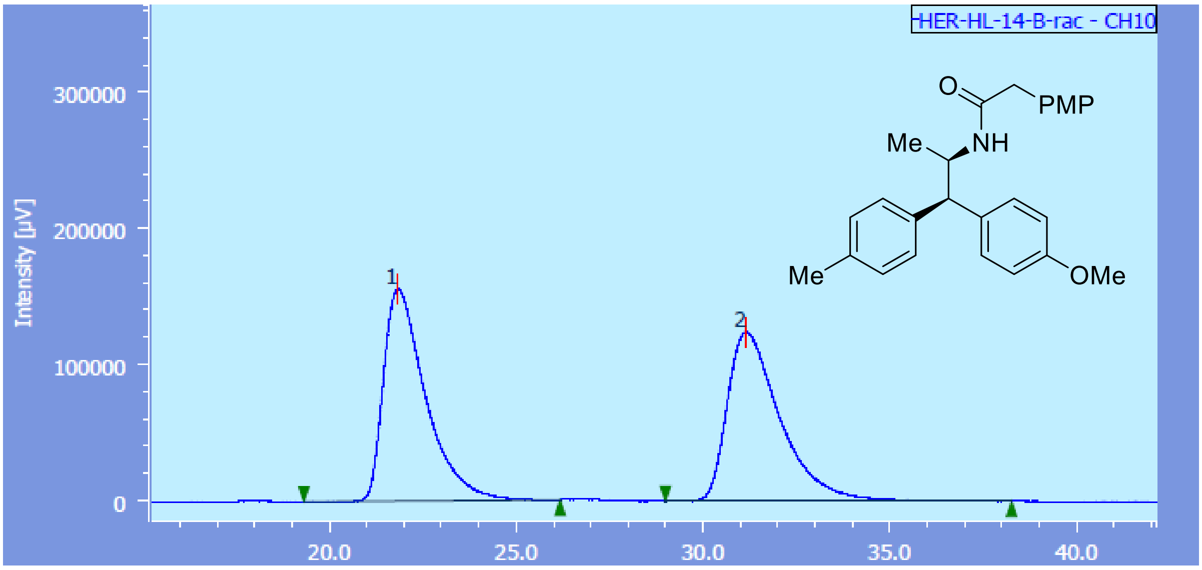

#	Peak Name	CH	tR [min]	Area [μV·sec]	Height [μV]	Area%	Height%	Quantity	NTP	Resolution	Symmetry Factor	Warning
1	Unknown	10	21.847	12249005	155821	49.869	55.630	N/A	1966	4.211	1.823	
2	Unknown	10	31.167	12313418	124284	50.131	44.370	N/A	2554	N/A	1.728	

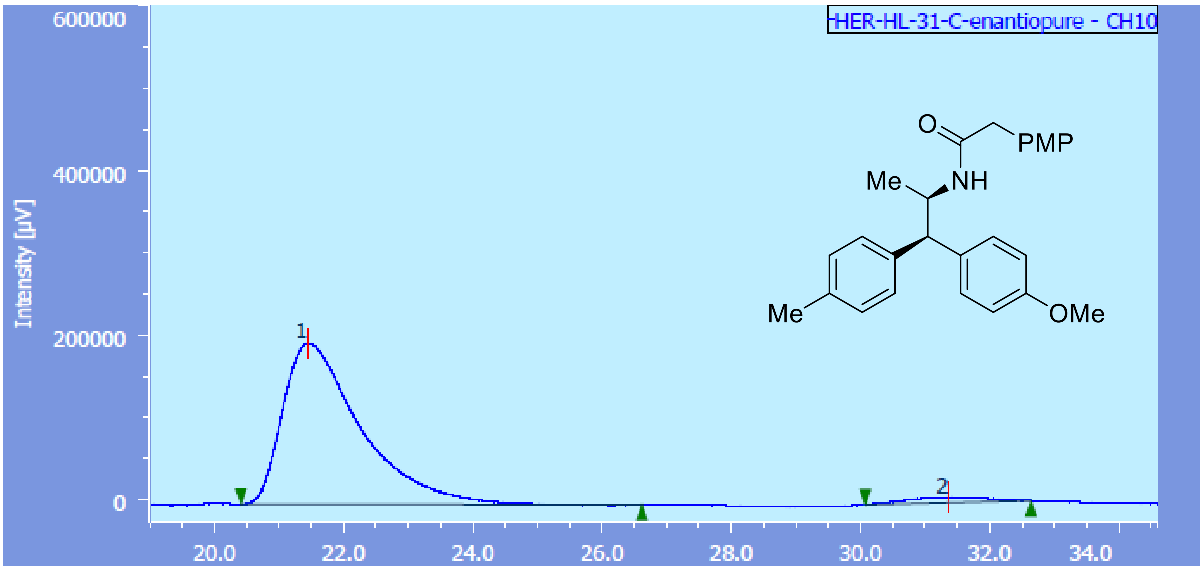

#	Peak Name	CH	tR [min]	Area [μV·sec]	Height [μV]	Area%	Height%	Quantity	NTP	Resolution	Symmetry Factor	Warning
1	Unknown	10	21.447	16367710	195468	96.701	96.830	N/A	1728	4.371	1.995	
2	Unknown	10	31.363	558471	6399	3.299	3.170	N/A	2547	N/A	0.996	

(*R*)-*N*-[(1*R*2*R*)-1-(4-Methoxyphenyl)-1-(*p*-tolyl)propan-2-yl]-2-phenylbutanamide (2.3)

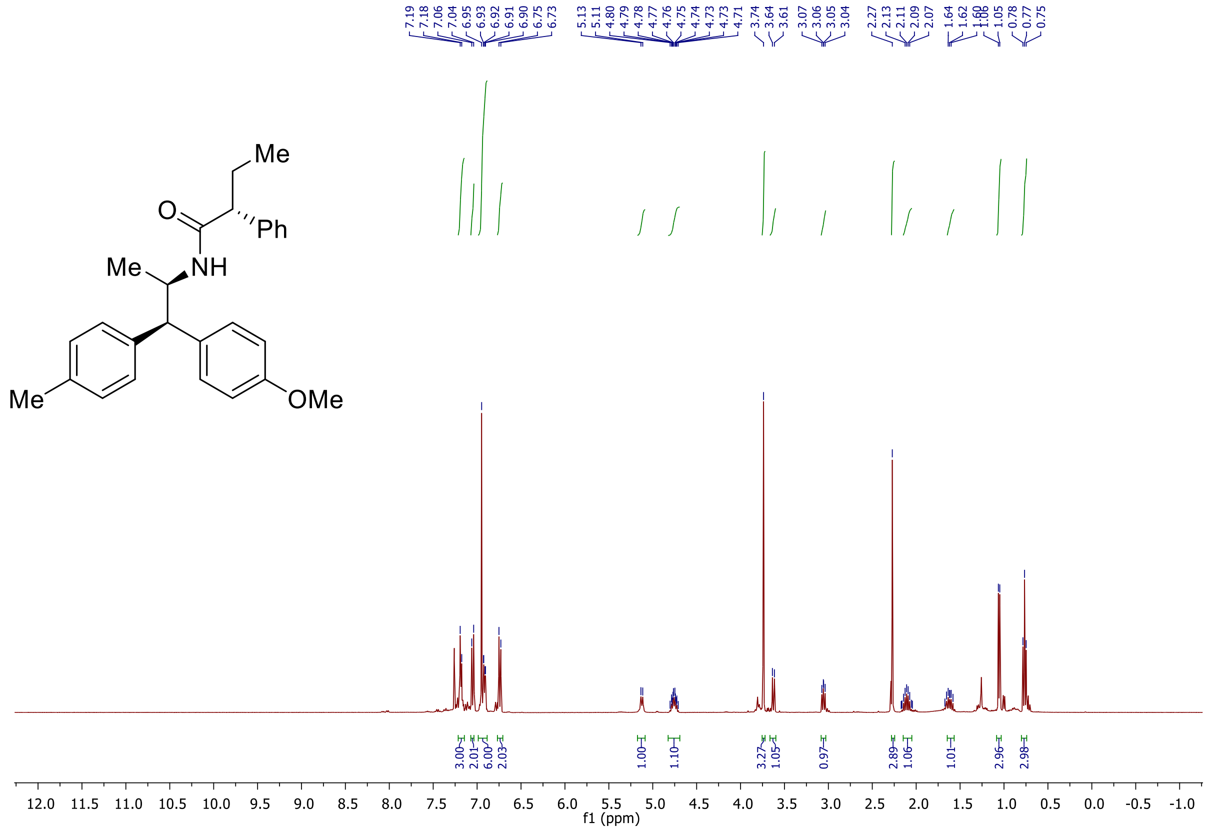

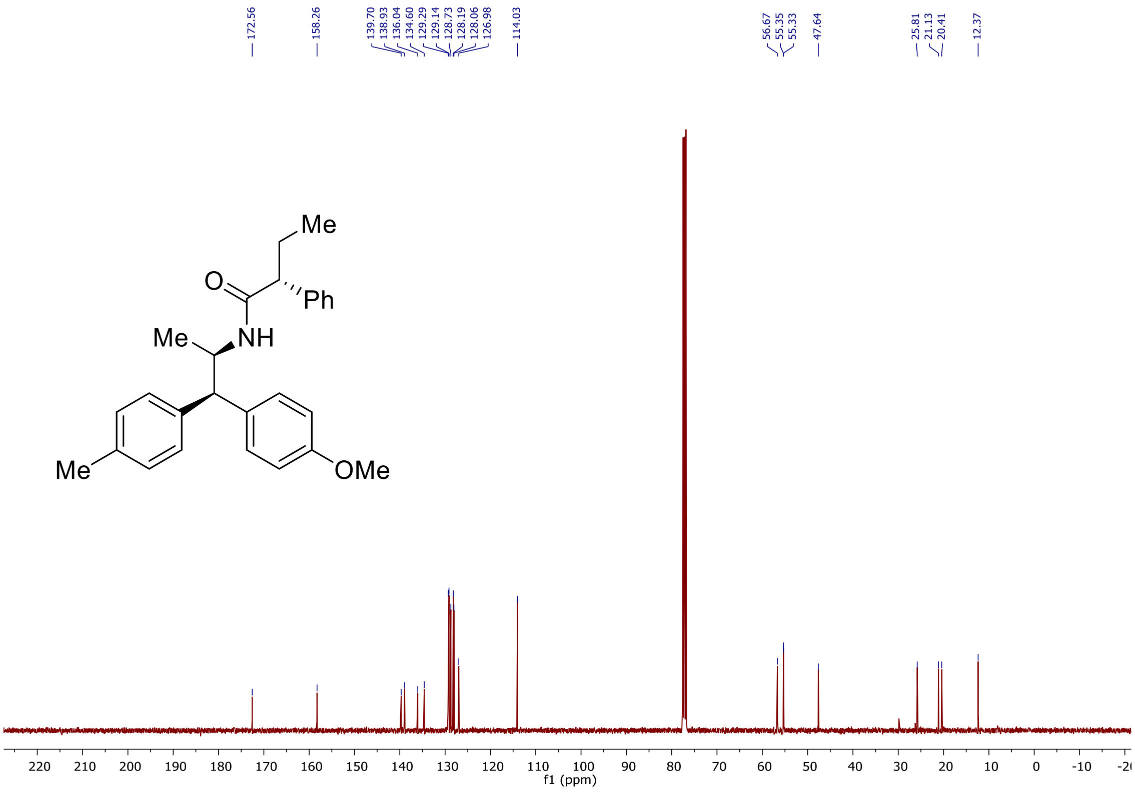

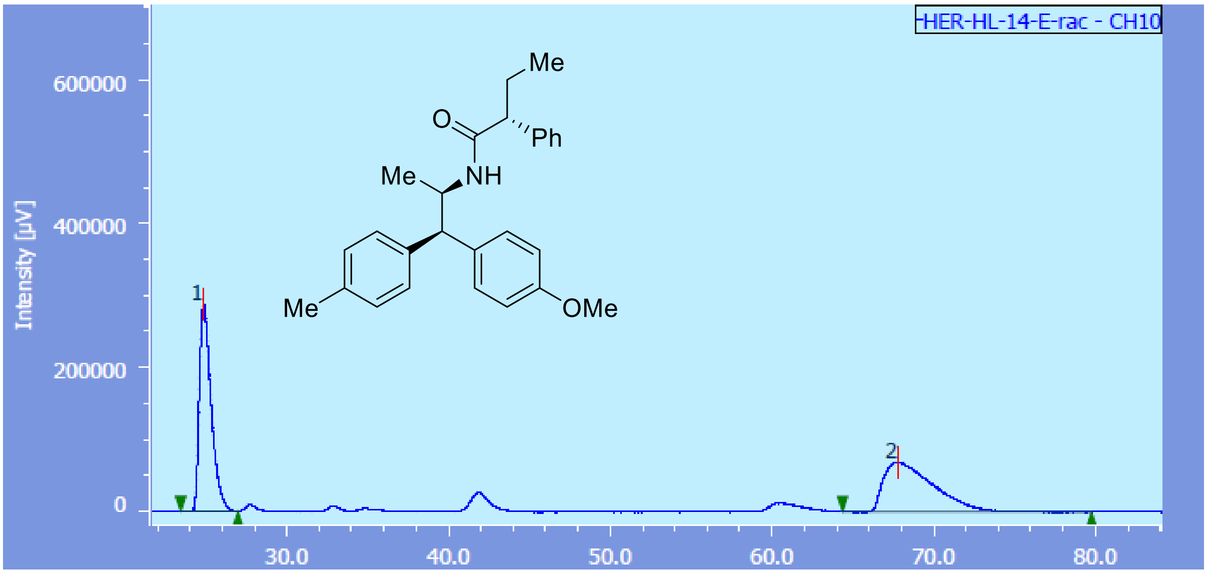

#	Peak Name	CH	tR [min]	Area [µV·sec]	Height [µV]	Area%	Height%	Quantity	NTP	Resolution	Symmetry Factor	Warning
1	Unknown	10	24.857	14653038	287714	49.810	80.700	N/A	5790	12.096	1.698	
2	Unknown	10	67.787	14764884	68811	50.190	19.300	N/A	2178	N/A	2.273	

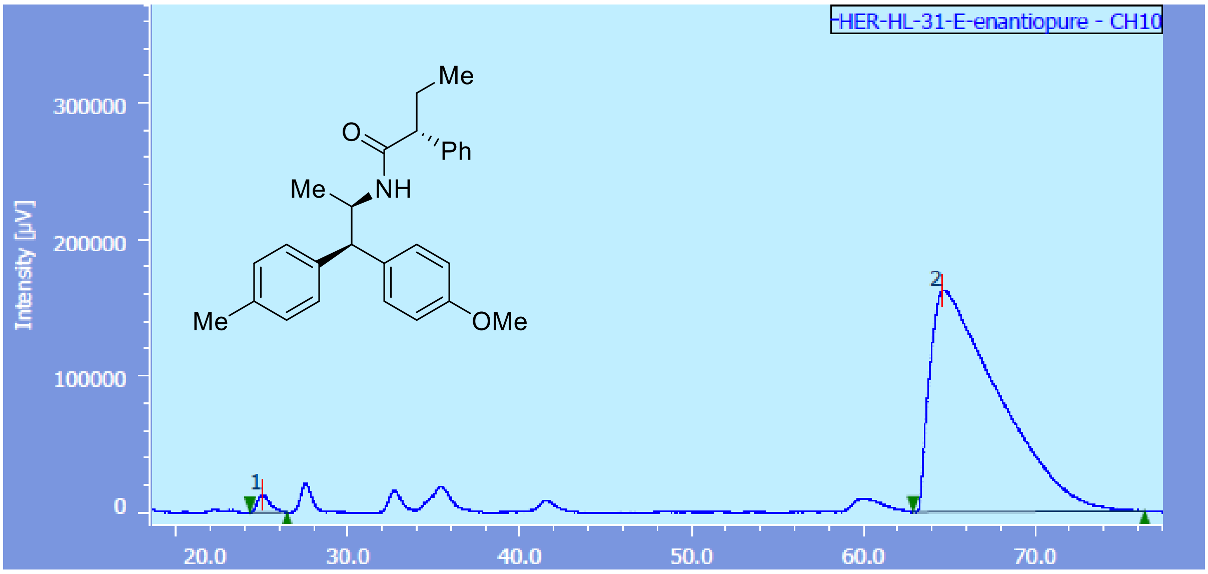

#	Peak Name	CH	tR [min]	Area [µV·sec]	Height [µV]	Area%	Height%	Quantity	NTP	Resolution	Symmetry Factor	Warning
1	Unknown	10	25.010	676295	13021	1.475	7.438	N/A	5606	9.058	1.431	
2	Unknown	10	64.607	45160520	162029	98.525	92.562	N/A	1210	N/A	3.547	

N-[(1R,2R)-1-(4-Methoxyphenyl)-1-(p-tolyl)propan-2-yl]cyclohexanecarboxamide (2.4)

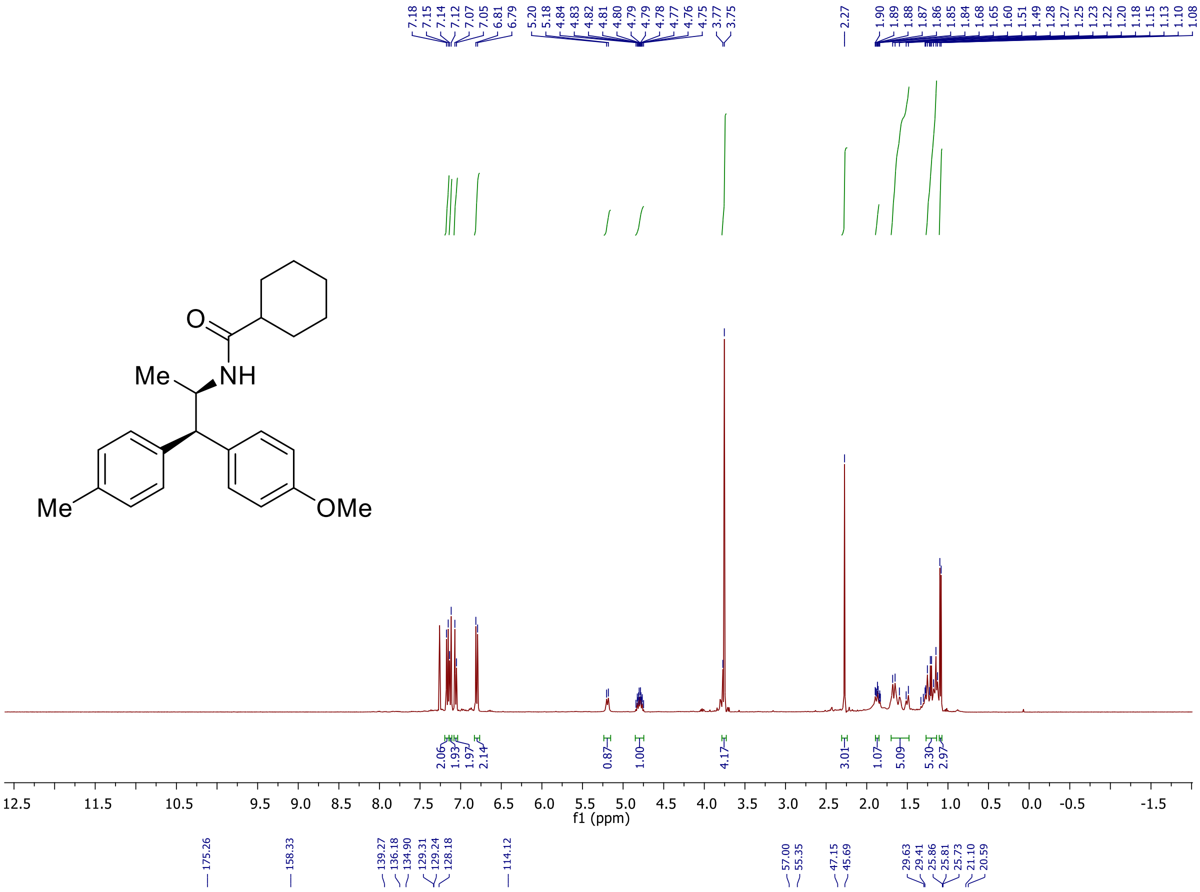

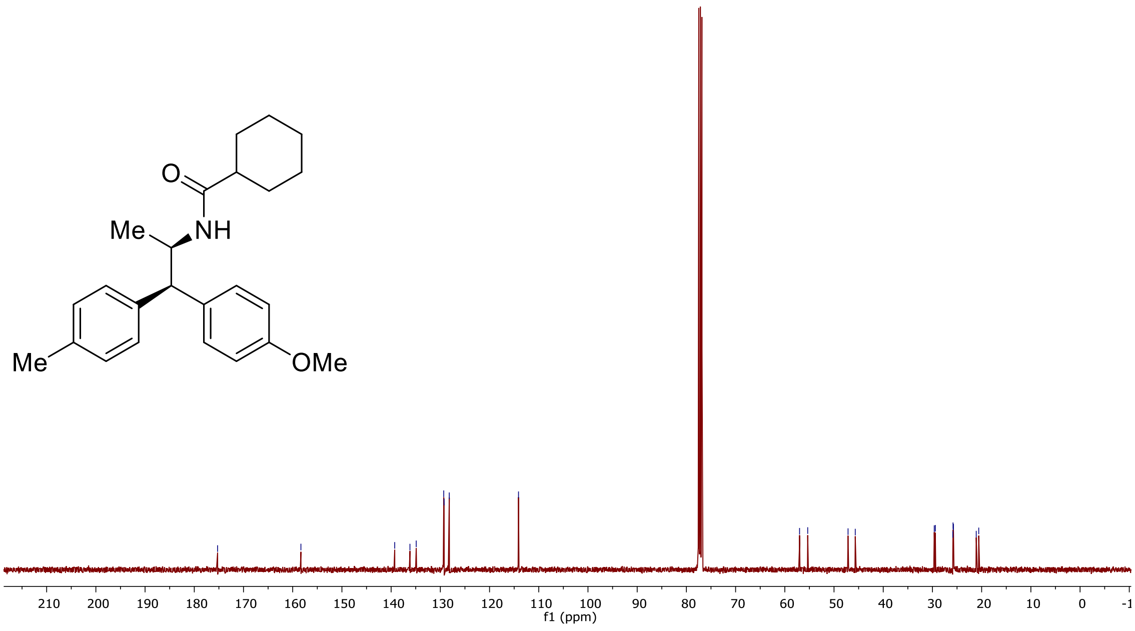

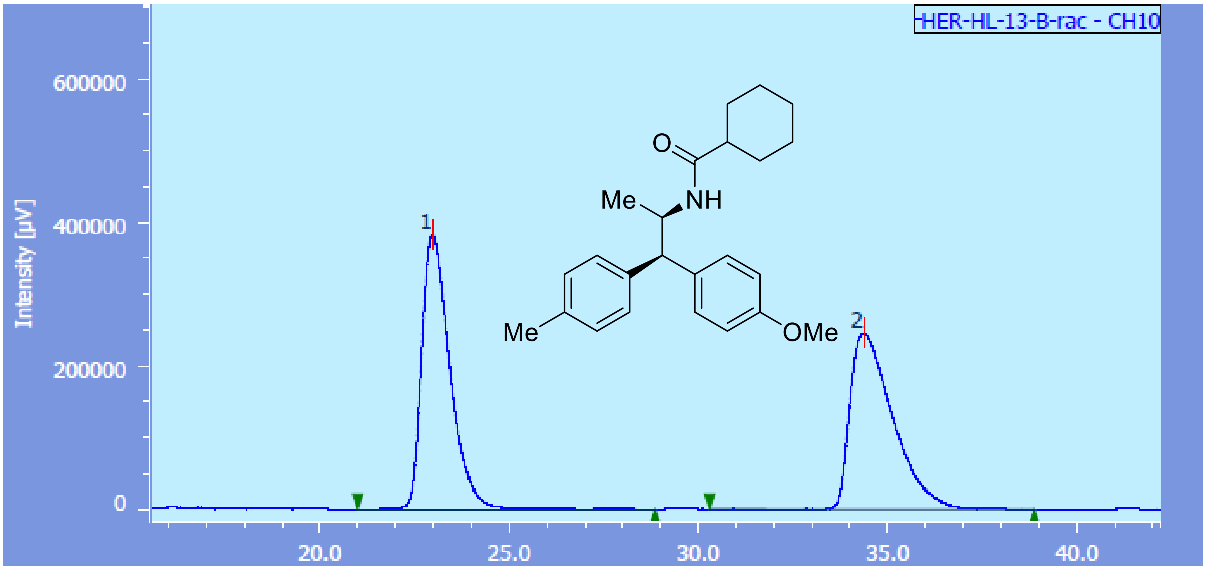

#	Peak Name	CH	tR [min]	Area [µV·sec]	Height [µV]	Area%	Height%	Quantity	NTP	Resolution	Symmetry Factor	Warning
1	Unknown	10	22.973	19800611	382995	50.525	60.889	N/A	4822	6.772	1.541	
2	Unknown	10	34.357	19388736	246012	49.475	39.111	N/A	4505	N/A	1.986	

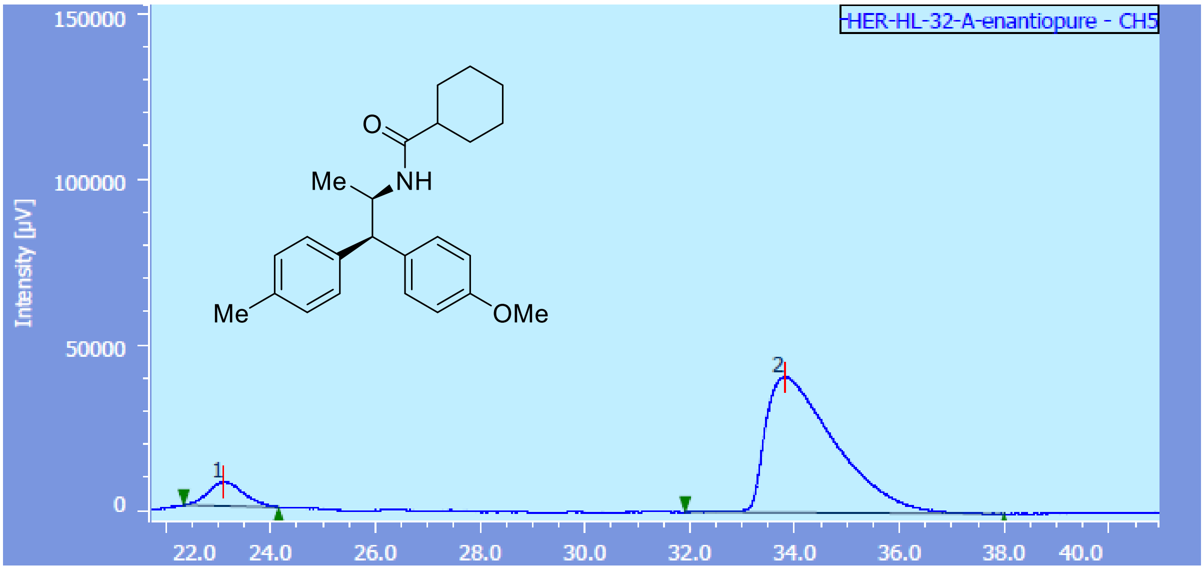

#	Peak Name	CH	tR [min]	Area [µV·sec]	Height [µV]	Area%	Height%	Quantity	NTP	Resolution	Symmetry Factor	Warning
1	Unknown	5	23.113	346989	7336	8.438	15.205	N/A	5427	5.867	1.160	
2	Unknown	5	33.817	3765452	40912	91.562	84.795	N/A	3167	N/A	2.365	

Ethyl 4-[((1*R*,2*R*)-1-(4-Methoxyphenyl)-1-(*p*-tolyl)propan-2-yl)amino]-4-oxobutanoate (2.5)

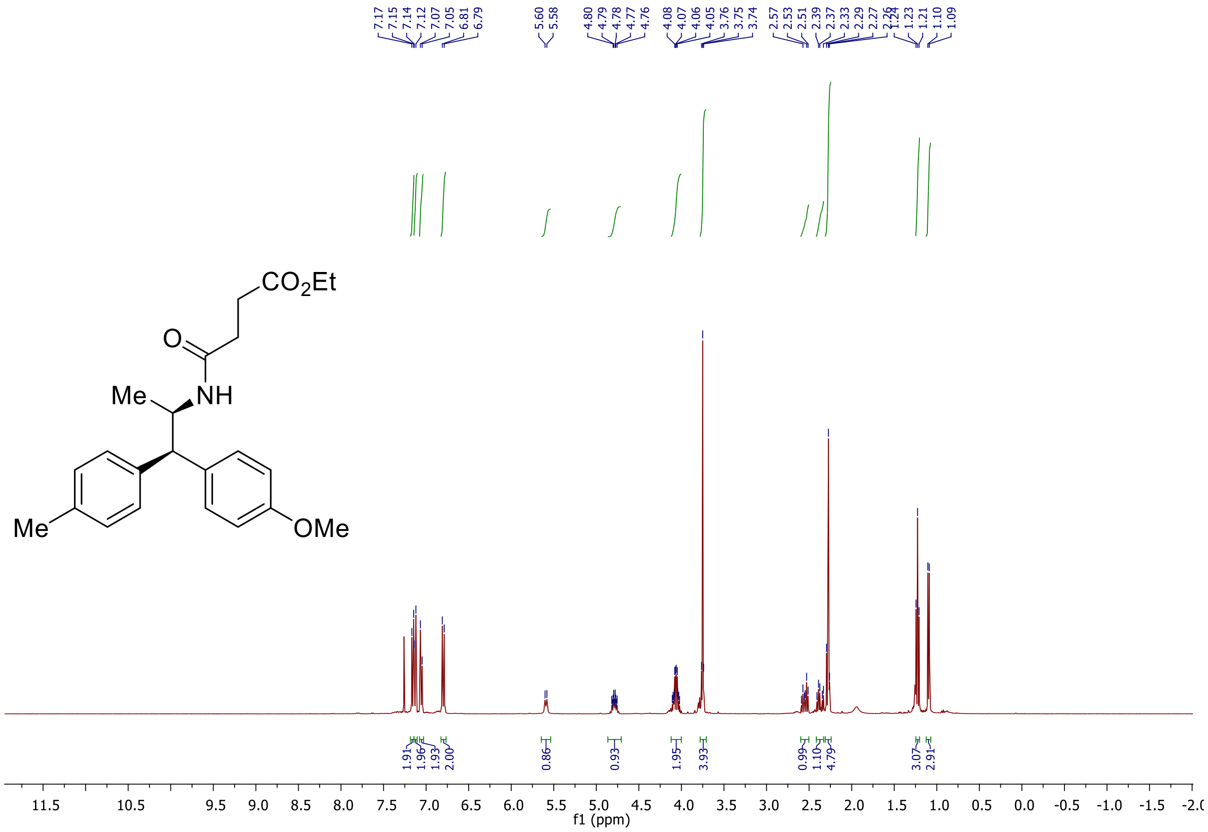

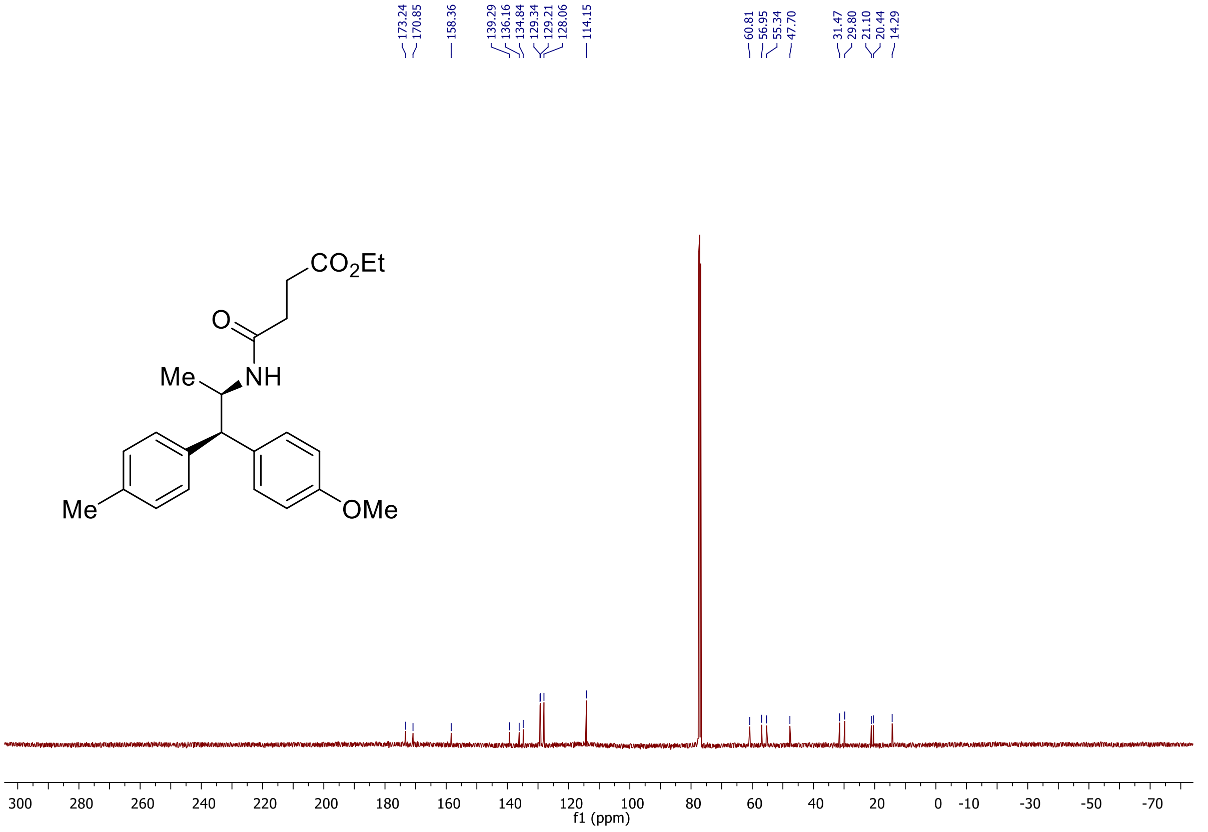

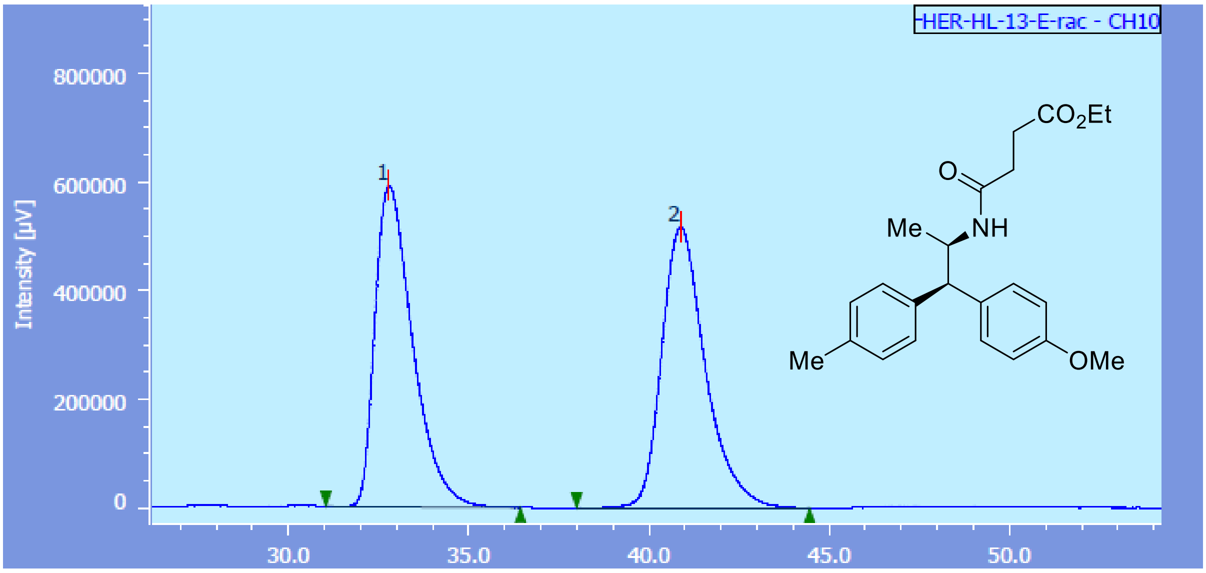

#	Peak Name	CH	tR [min]	Area [µV·sec]	Height [µV]	Area%	Height%	Quantity	NTP	Resolution	Symmetry Factor	Warning
1	Unknown	10	32.787	42910586	591283	49.644	53.347	N/A	4894	4.053	1.615	
2	Unknown	10	40.873	43525157	517098	50.356	46.653	N/A	5912	N/A	1.349	

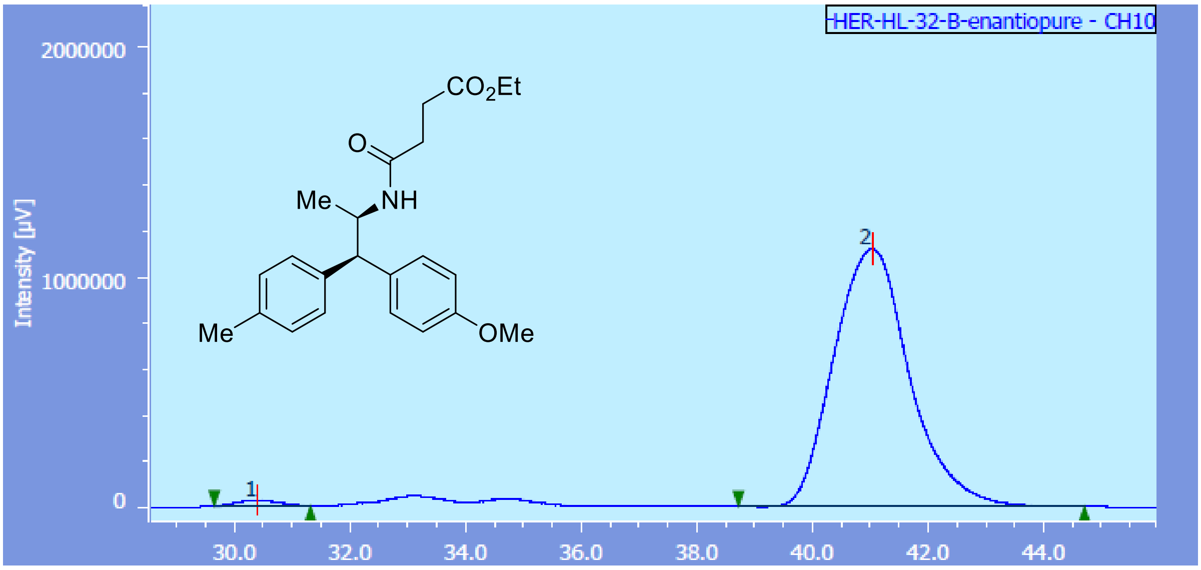

#	Peak Name	CH	tR [min]	Area [µV·sec]	Height [µV]	Area%	Height%	Quantity	NTP	Resolution	Symmetry Factor	Warning
1	Unknown	10	30.400	1269814	25140	1.233	2.200	N/A	7494	5.653	1.099	
2	Unknown	10	41.027	101755692	1117526	98.767	97.800	N/A	4814	N/A	1.181	

(*S*)-3-[(*tert*-Butyldimethylsilyl)oxy)-*N*-((1*R*,2*R*)-1-(4-methoxyphenyl)-1-(*p*-tolyl)propan-2-yl]butanamide (2.6)

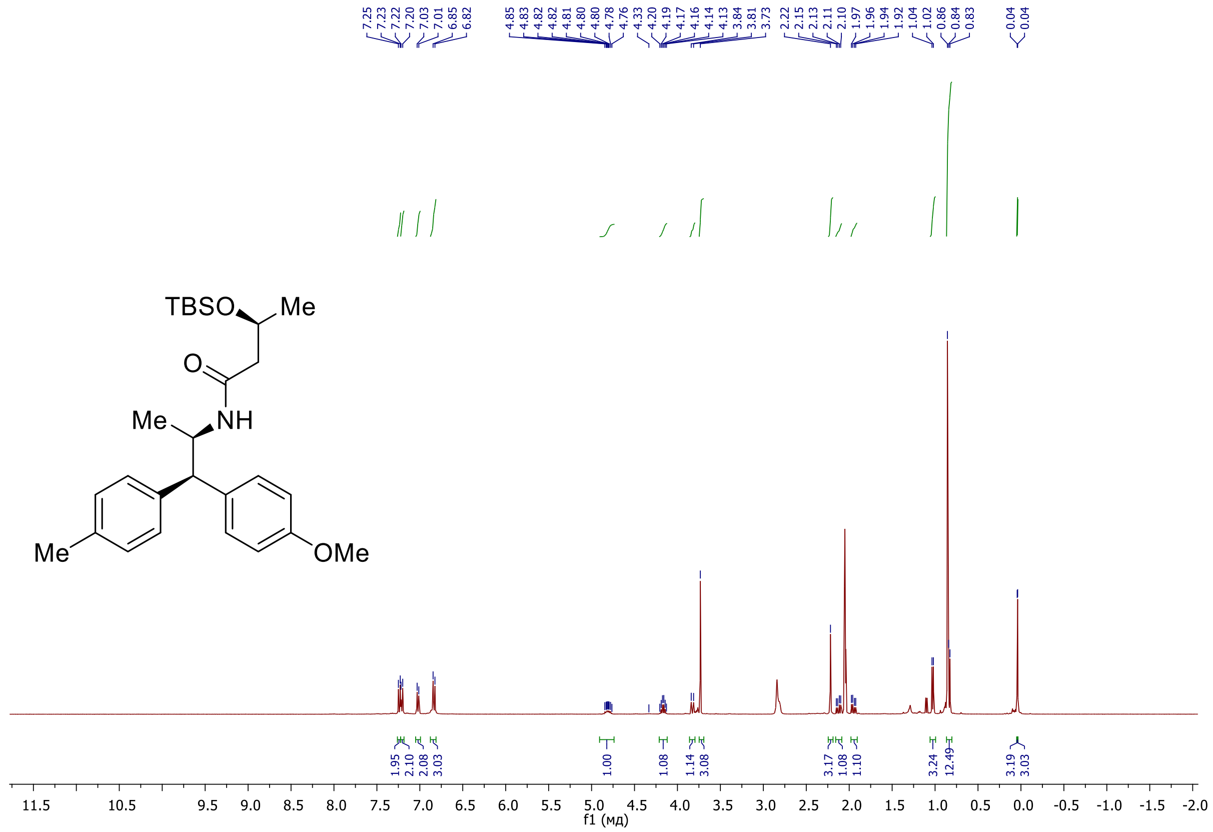

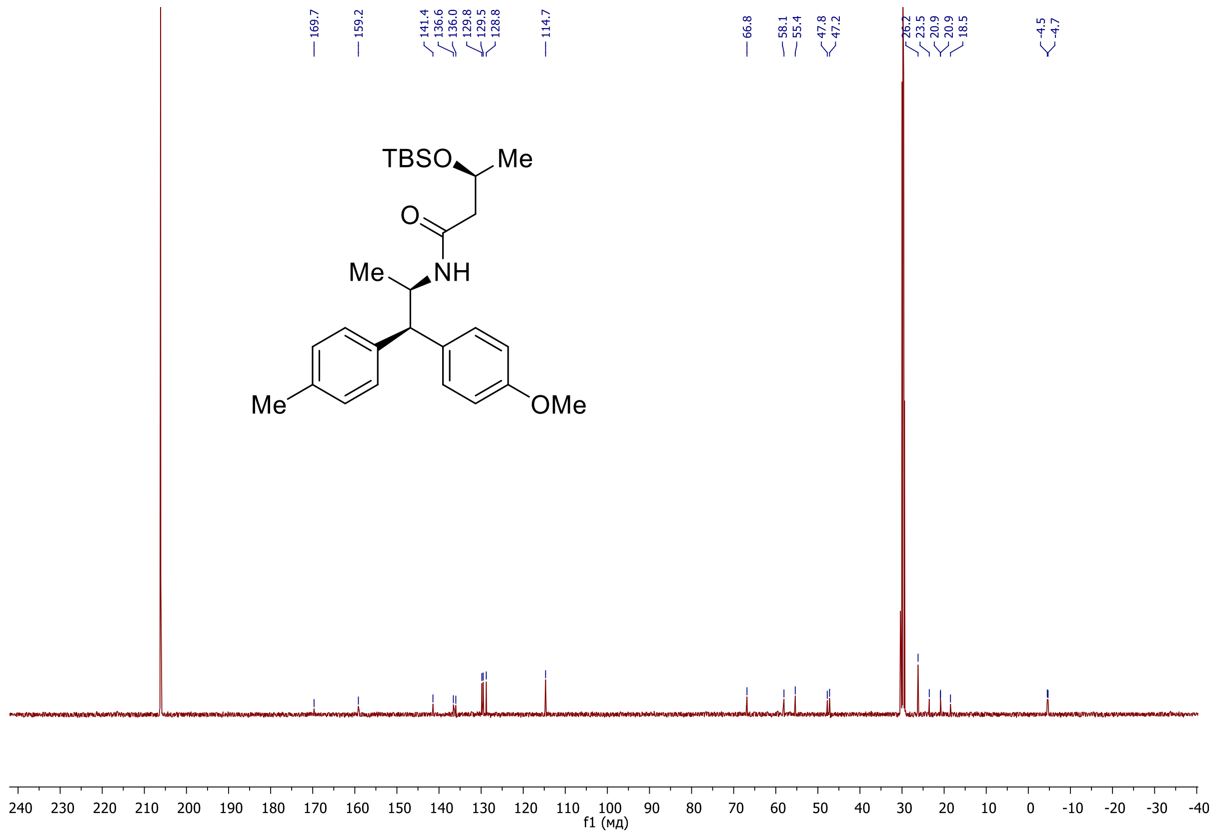

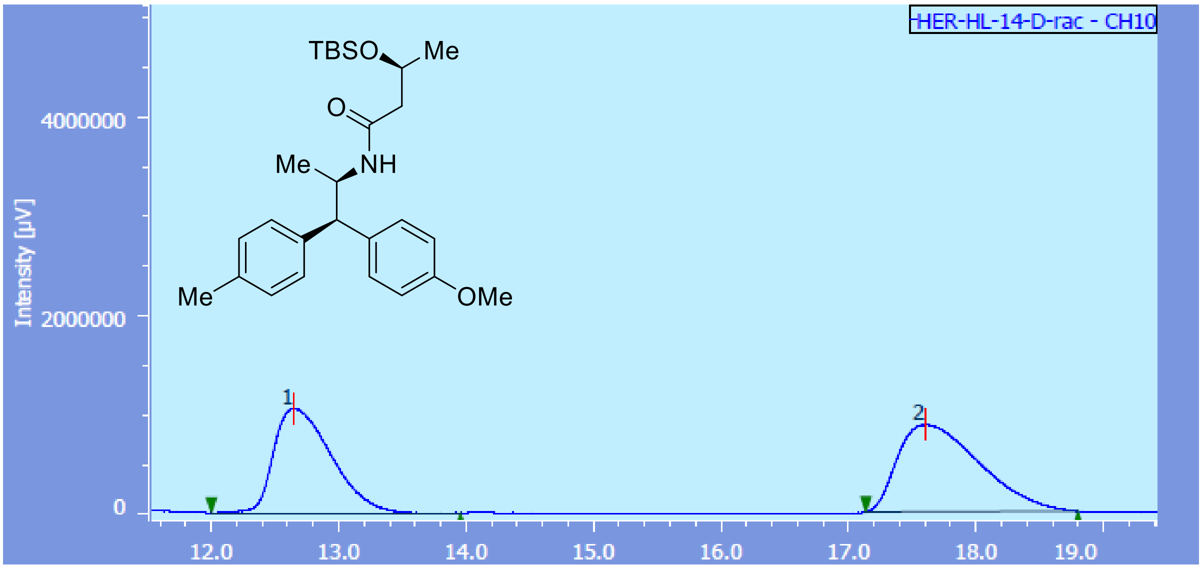

#	Peak Name	CH	tR [min]	Area [µV·sec]	Height [µV]	Area%	Height%	Quantity	NTP	Resolution	Symmetry Factor	Warning
1	Unknown	10	12.650	32857613	1049288	45.606	54.488	N/A	3650	4.829	1.586	
2	Unknown	10	17.600	39188435	876442	54.394	45.512	N/A	3340	N/A	1.733	

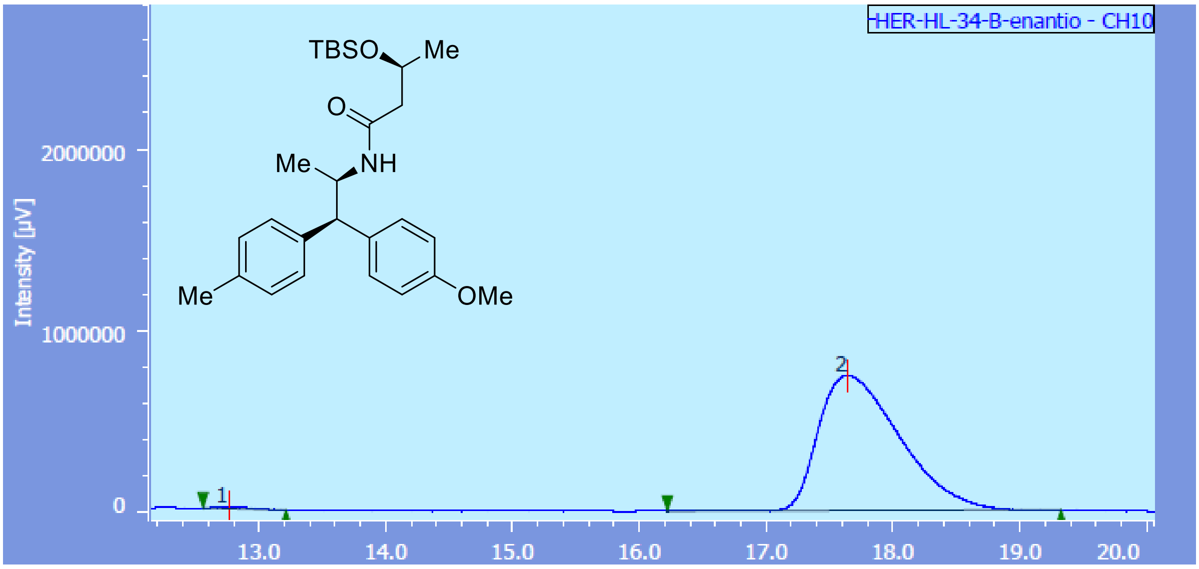

#	Peak Name	CH	tR [min]	Area [µV·sec]	Height [µV]	Area%	Height%	Quantity	NTP	Resolution	Symmetry Factor	Warning
1	Unknown	10	12.760	174025	8027	0.524	1.070	N/A	7240	5.464	1.509	
2	Unknown	10	17.643	33021429	742306	99.476	98.930	N/A	3502	N/A	1.621	

N-[(1R,2R)-1-(4-Methoxyphenyl)-1-(p-tolyl)propan-2-yl]benzamide (2.7)

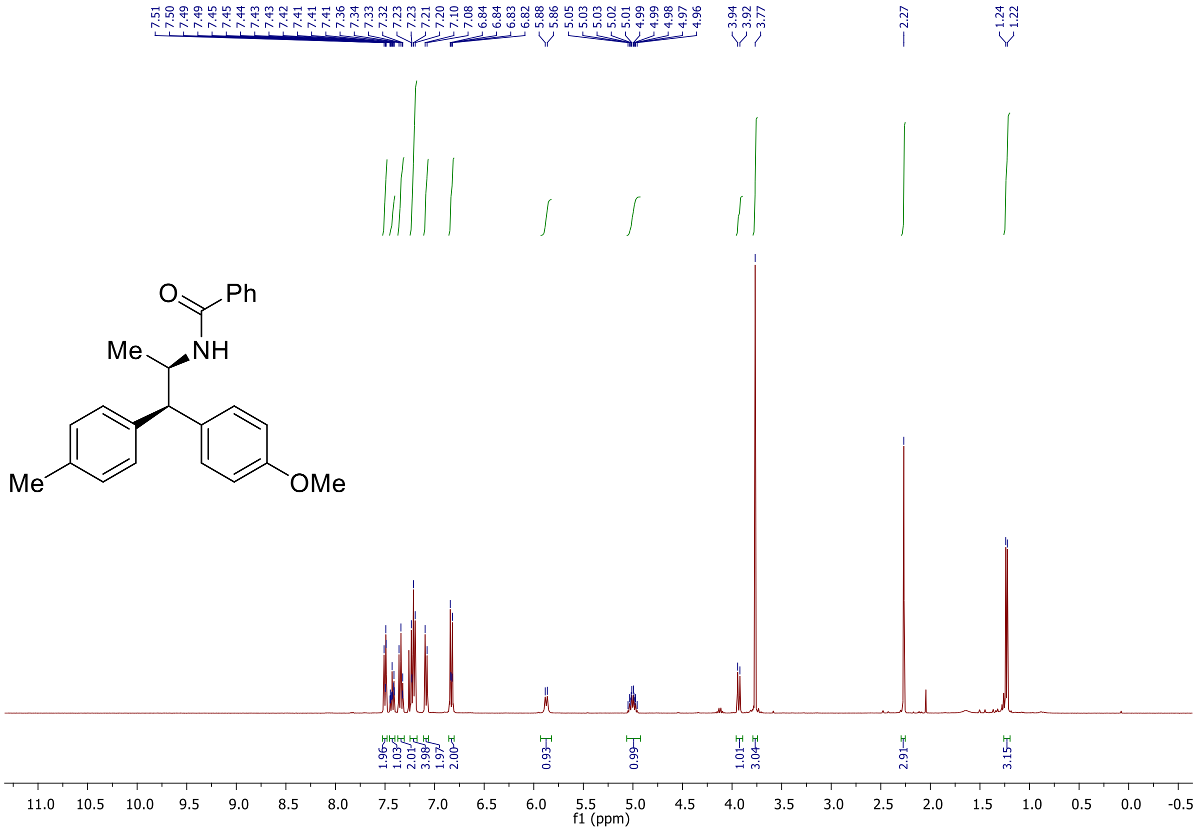

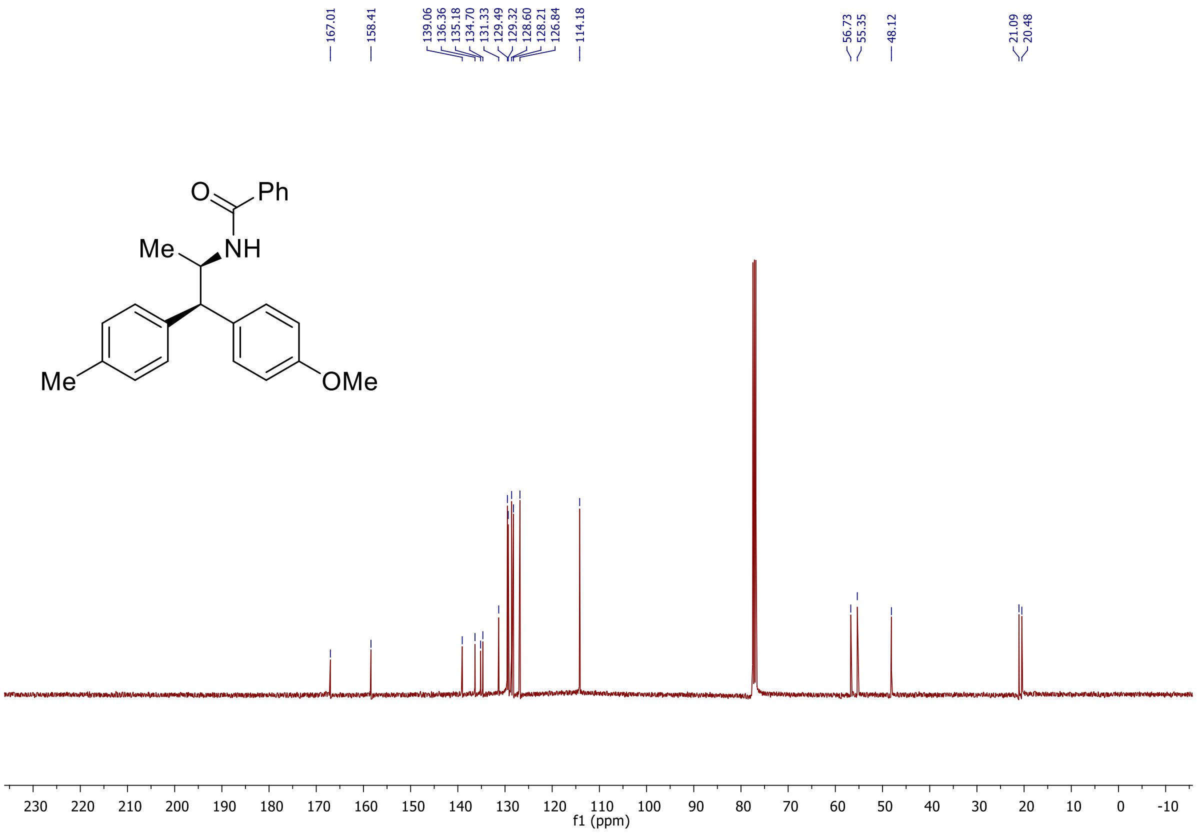

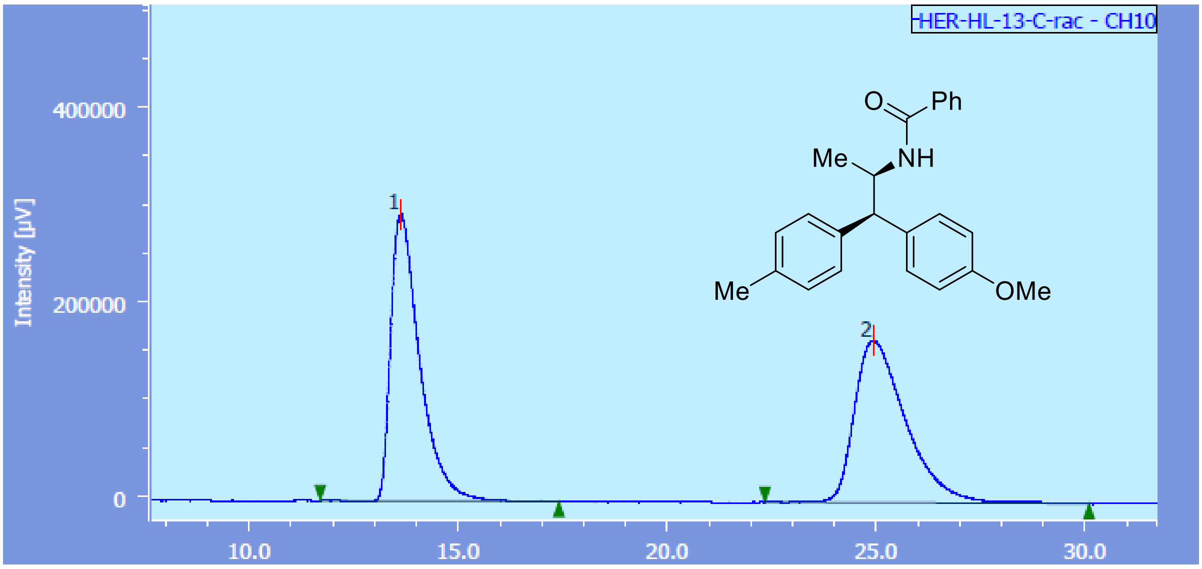

#	Peak Name	CH	tR [min]	Area [µV·sec]	Height [µV]	Area%	Height%	Quantity	NTP	Resolution	Symmetry Factor	Warning
1	Unknown	10	13.623	14215842	295290	49.709	63.848	N/A	2040	6.695	1.867	
2	Unknown	10	24.923	14382094	167200	50.291	36.152	N/A	2095	N/A	1.631	

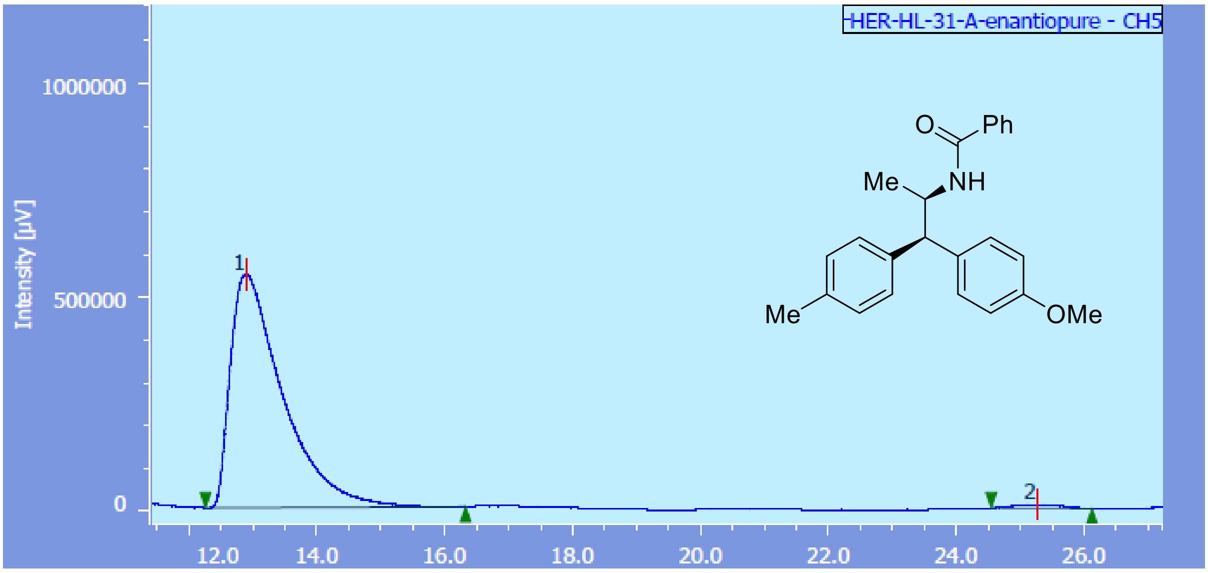

#	Peak Name	CH	tR [min]	Area [µV·sec]	Height [µV]	Area%	Height%	Quantity	NTP	Resolution	Symmetry Factor	Warning
1	Unknown	5	12.890	31008634	546102	98.508	98.352	N/A	1335	8.652	2.367	
2	Unknown	5	25.253	469702	9148	1.492	1.648	N/A	4824	N/A	1.105	

N-[(1*R*,2*R*)-1-(4-Methoxyphenyl)-1-(*p*-tolyl)propan-2-yl]furan-2-carboxamide (2.8)

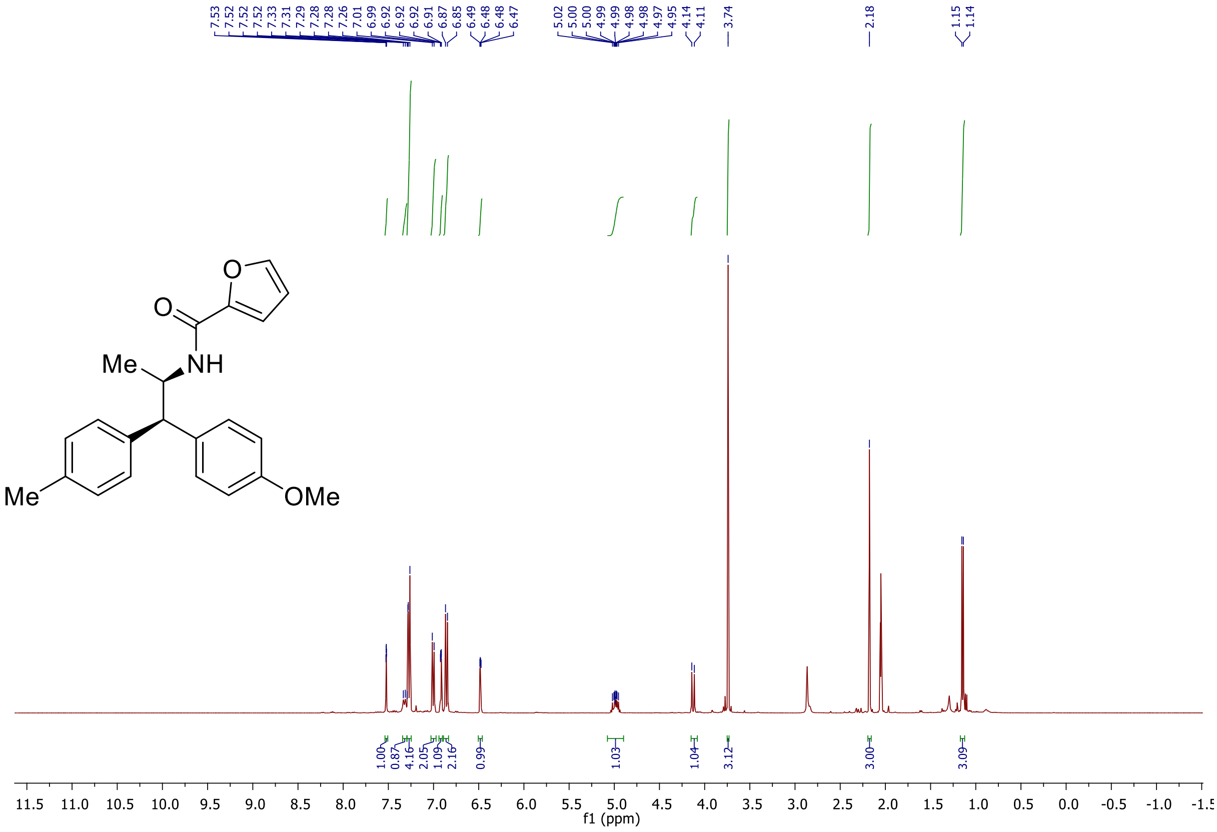

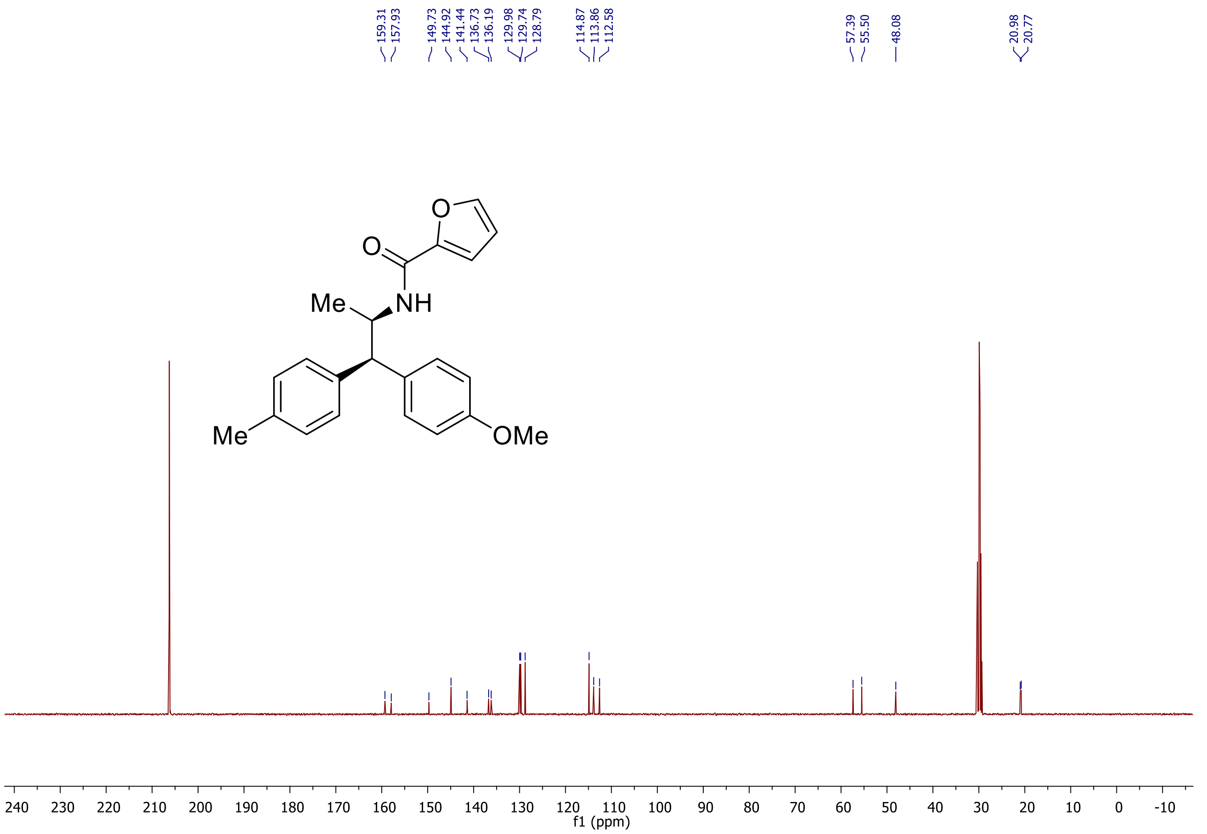

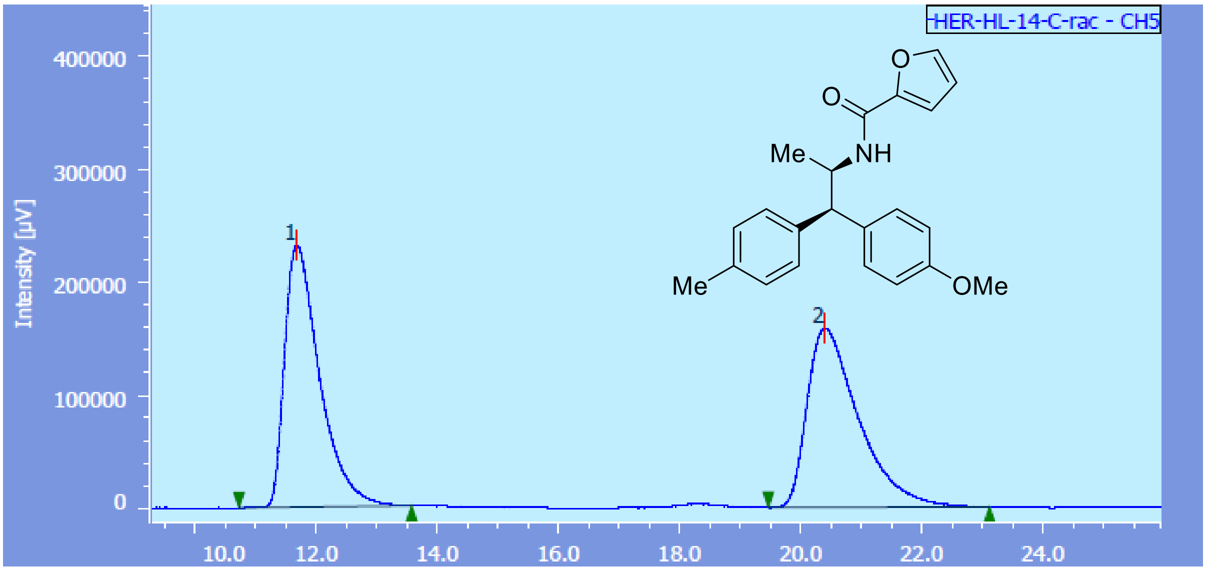

#	Peak Name	CH	tR [min]	Area [µV·sec]	Height [µV]	Area%	Height%	Quantity	NTP	Resolution	Symmetry Factor	Warning
1	Unknown	5	11.677	9285908	231741	49.720	59.384	N/A	2115	6.967	1.812	
2	Unknown	5	20.397	9390527	158500	50.280	40.616	N/A	2981	N/A	1.809	

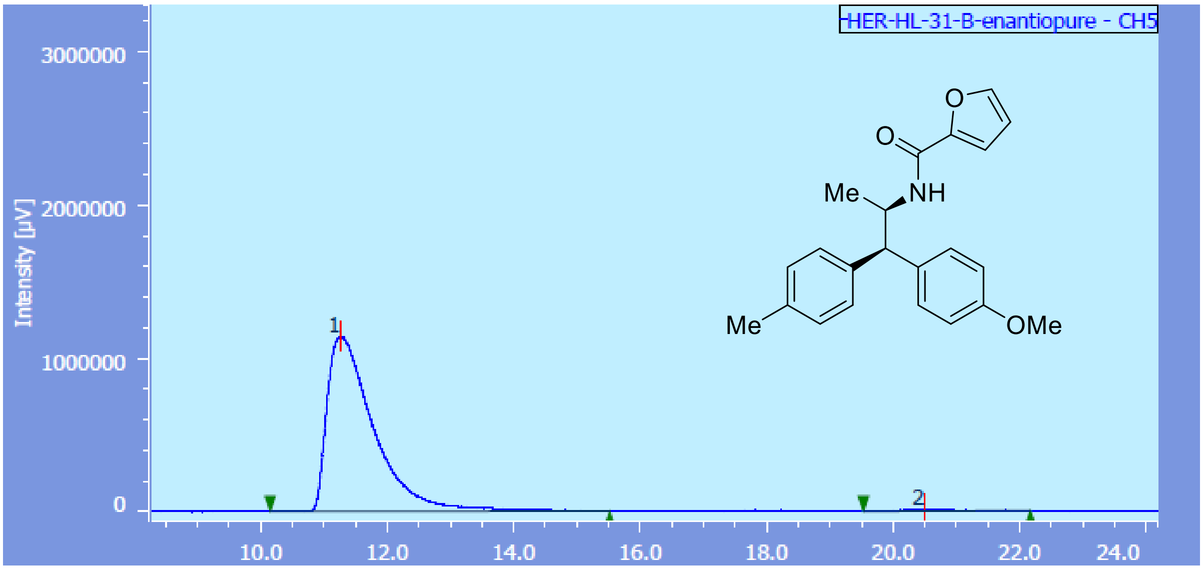

#	Peak Name	CH	tR [min]	Area [µV·sec]	Height [µV]	Area%	Height%	Quantity	NTP	Resolution	Symmetry Factor	Warning
1	Unknown	5	11.267	58721349	1140882	98.756	98.970	N/A	1285	6.806	2.392	
2	Unknown	5	20.500	739972	11876	1.244	1.030	N/A	3140	N/A	1.661	

tert-Butyl-[(1*R*,2*R*)-1-(4-methoxyphenyl)-1-(*p*-tolyl)propan-2-yl]carbamate (2.9)

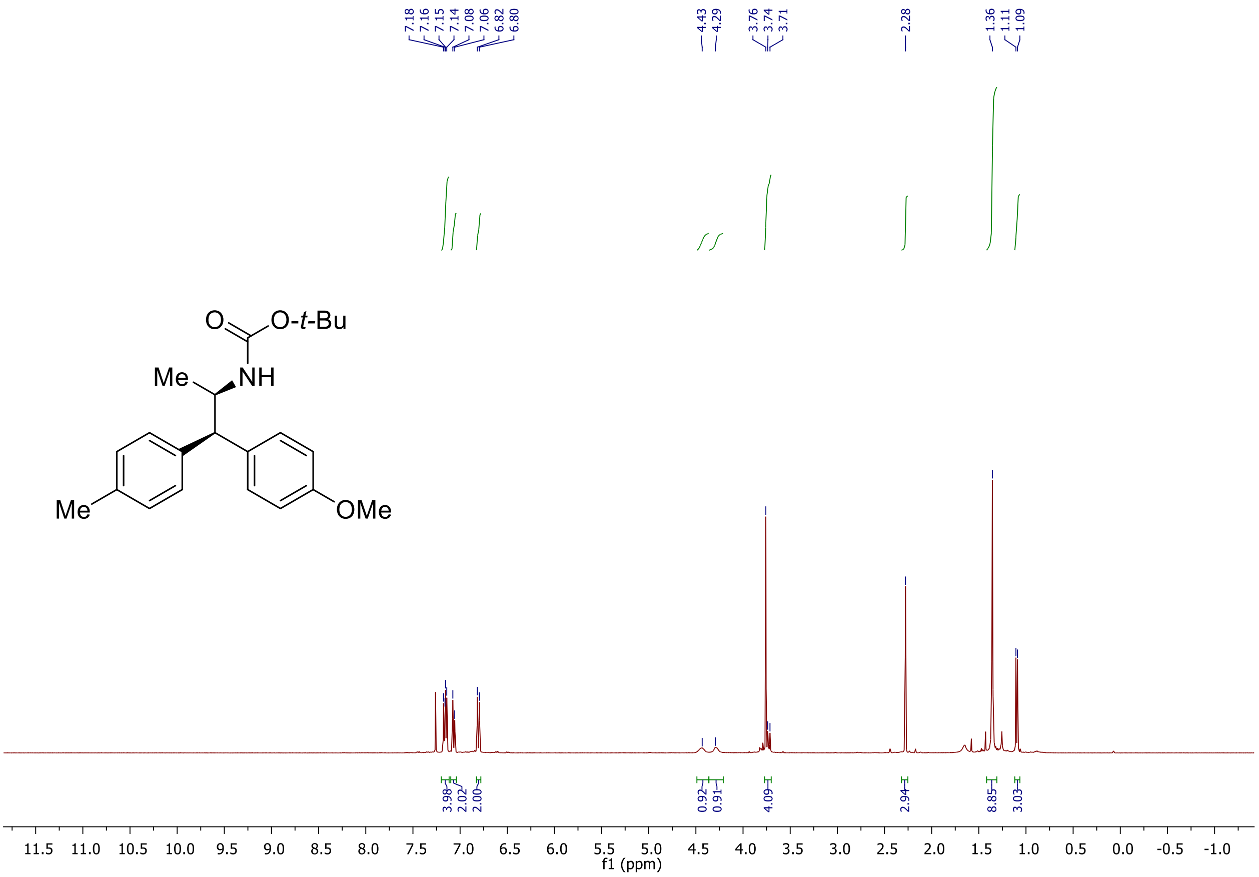

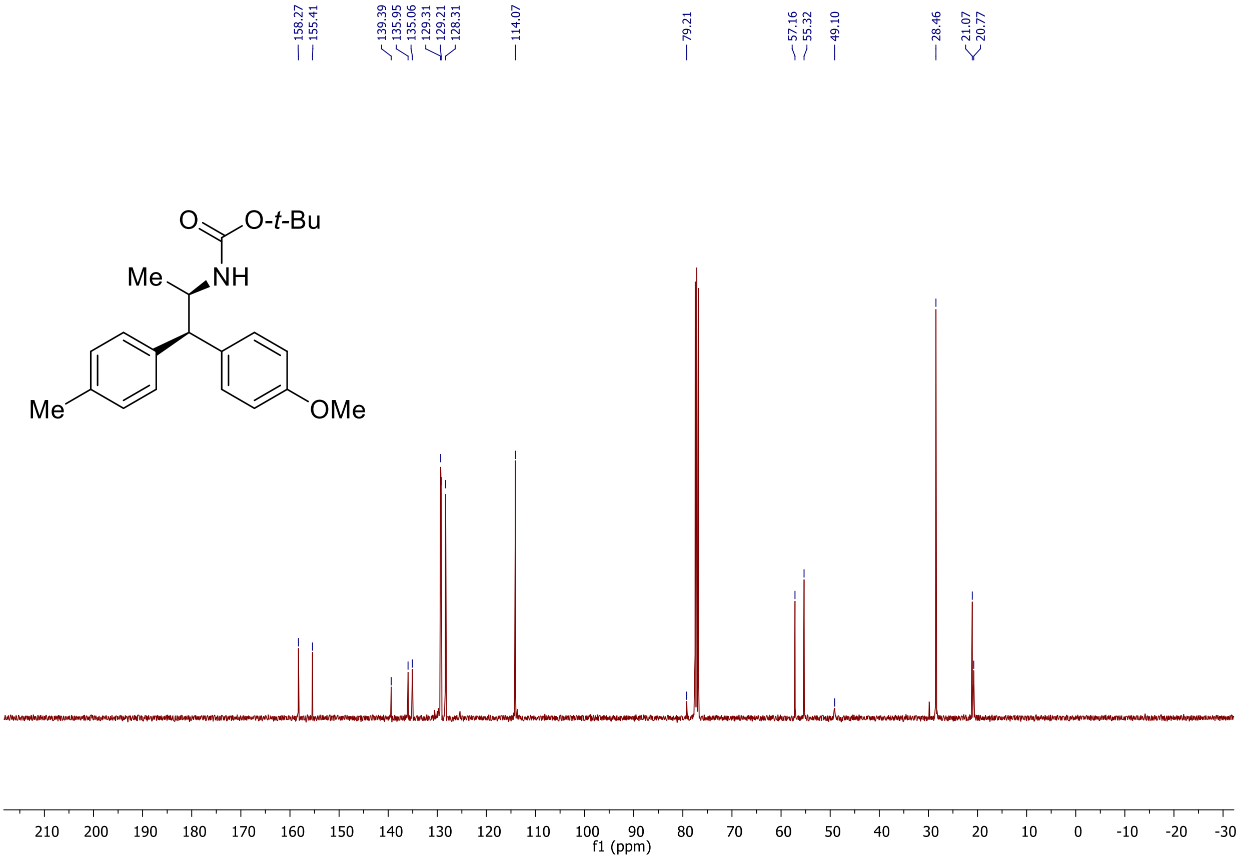

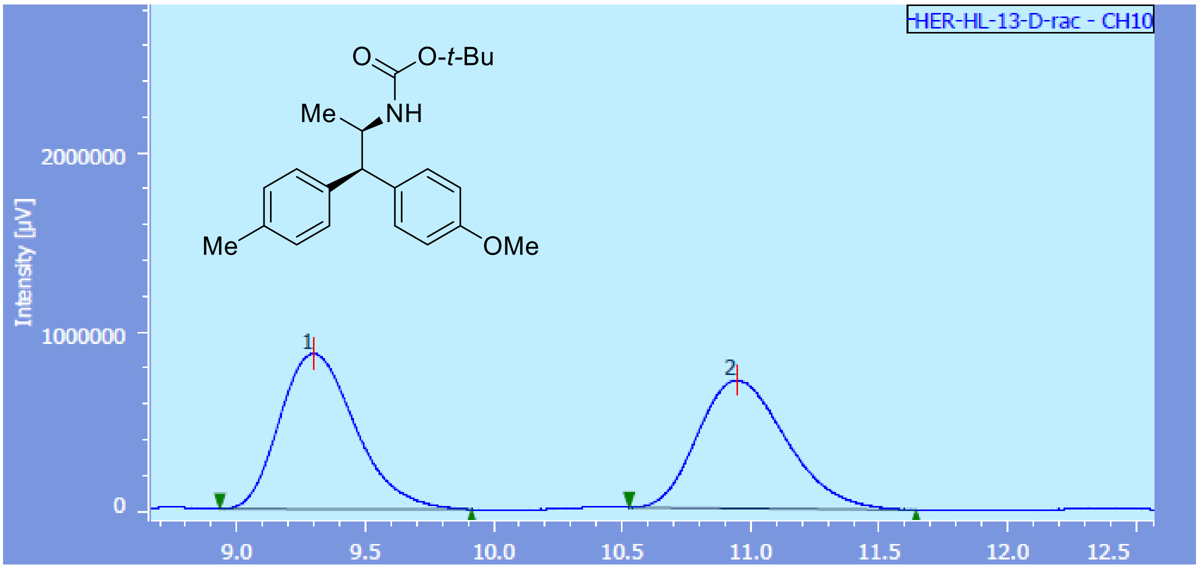

#	Peak Name	CH	tR [min]	Area [μV·sec]	Height [μV]	Area%	Height%	Quantity	NTP	Resolution	Symmetry Factor	Warning
1	Unknown	10	9.300	17816959	868302	50.898	54.834	N/A	4762	2.805	1.268	
2	Unknown	10	10.947	17188389	715210	49.102	45.166	N/A	4709	N/A	1.244	

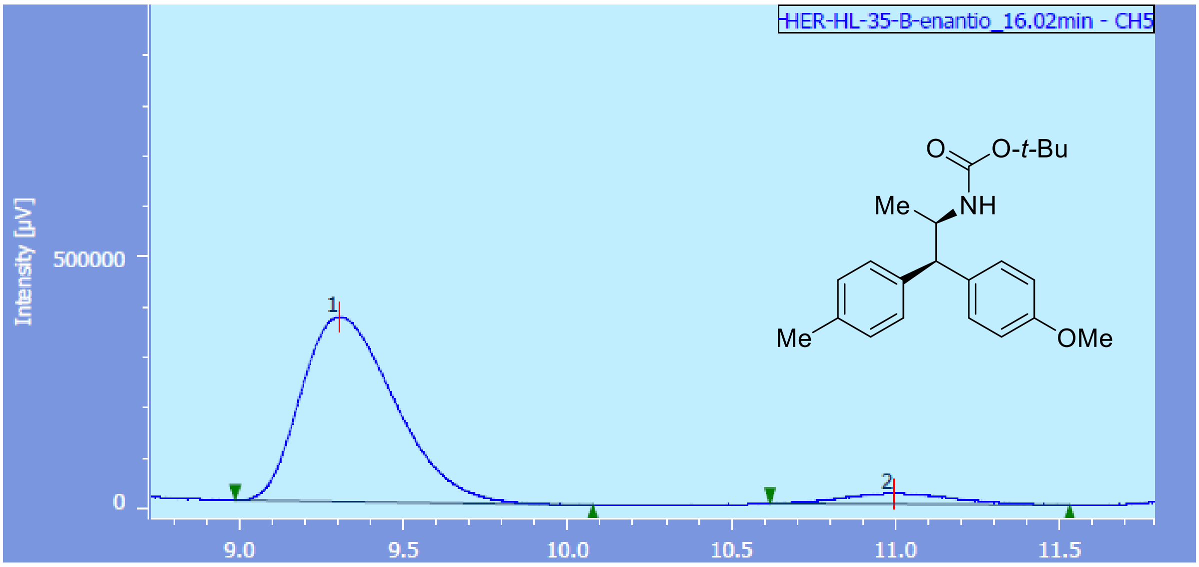

#	Peak Name	CH	tR [min]	Area [μV·sec]	Height [μV]	Area%	Height%	Quantity	NTP	Resolution	Symmetry Factor	Warning
1	Unknown	5	9.303	7303921	367135	93.731	94.765	N/A	4902	2.889	1.383	
2	Unknown	5	10.993	488520	20279	6.269	5.235	N/A	4698	N/A	1.149	

N-[(1R,2R)-1-(4-Methoxyphenyl)-1-phenylpropan-2-yl]butyramide (2.10)

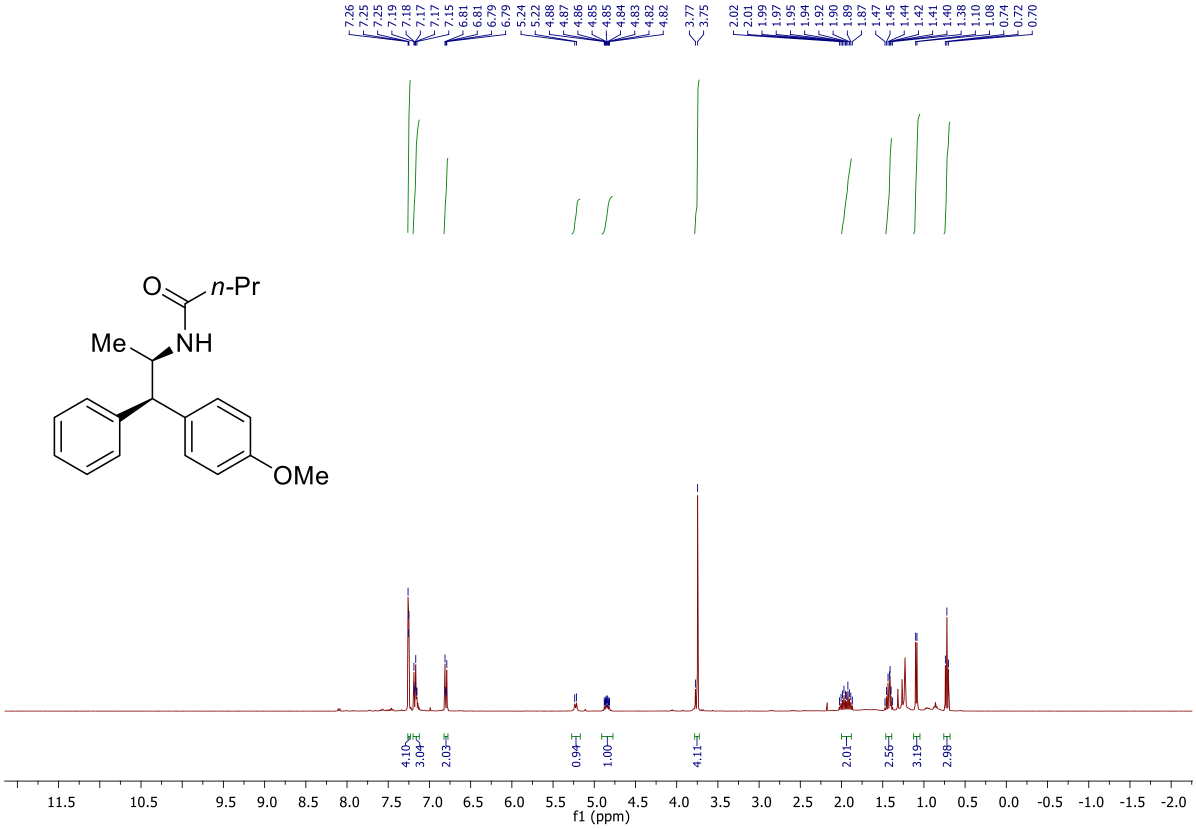

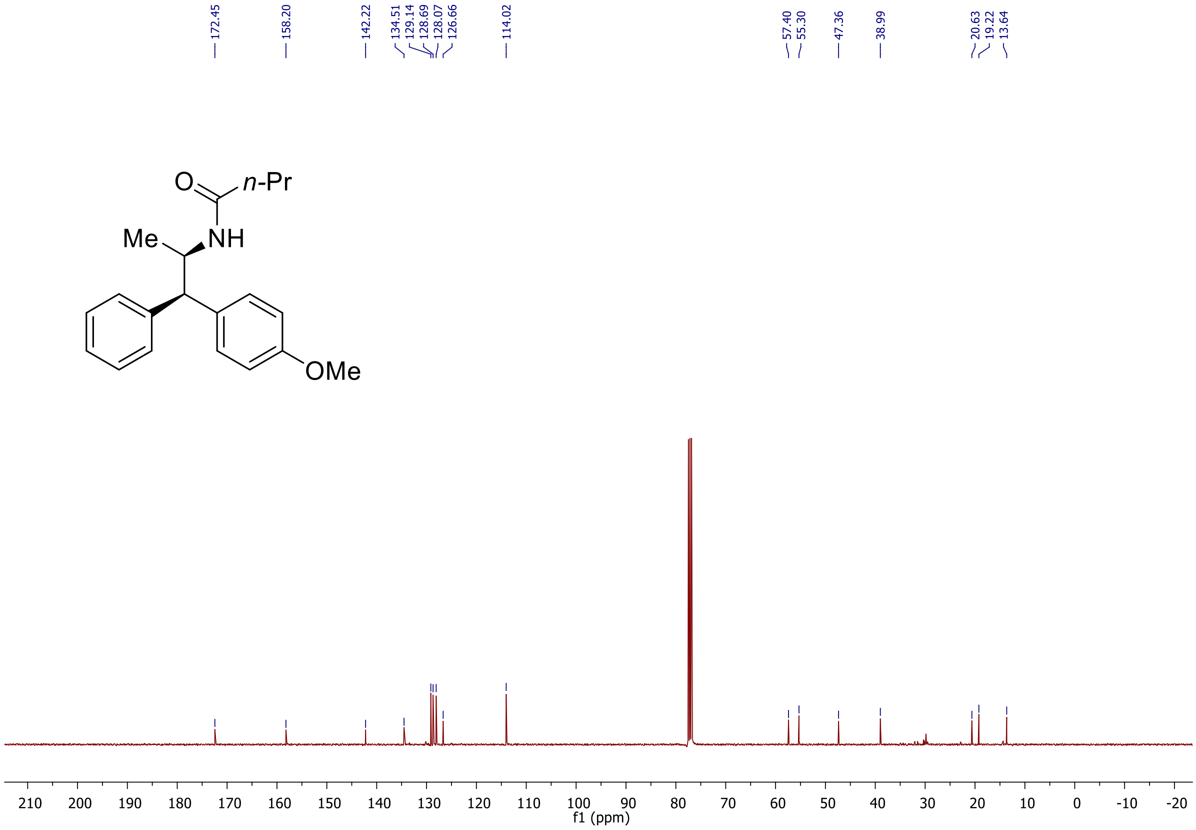

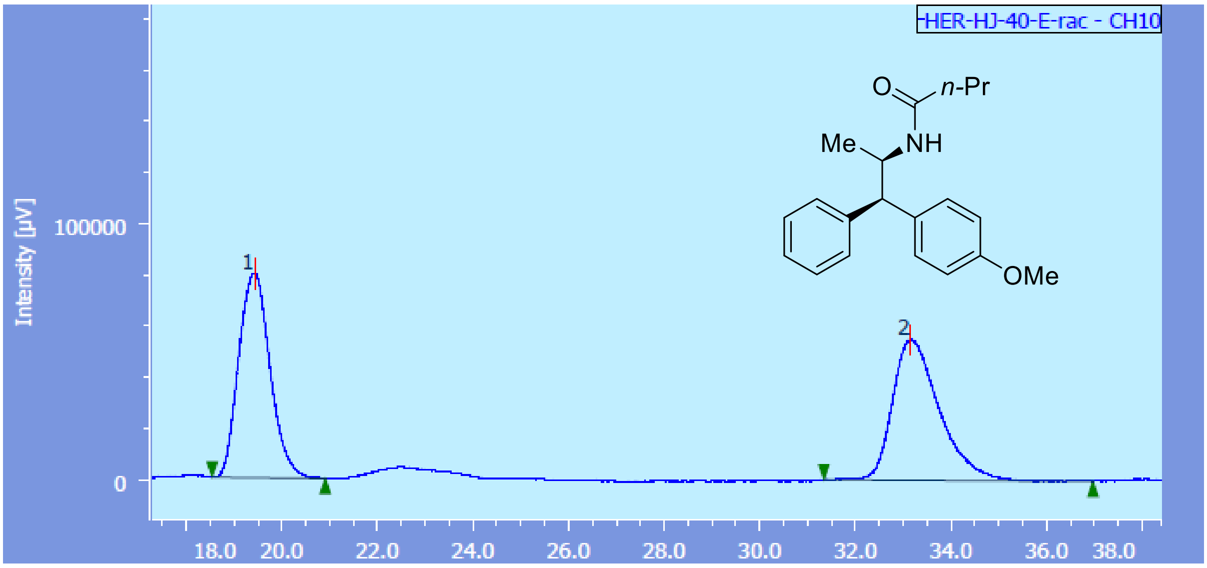

#	Peak Name	CH	tR [min]	Area [µV·sec]	Height [µV]	Area%	Height%	Quantity	NTP	Resolution	Symmetry Factor	Warning
1	Unknown	10	19.427	3644138	79815	49.311	59.261	N/A	4075	9.329	1.149	
2	Unknown	10	33.147	3746043	54869	50.689	40.739	N/A	5860	N/A	1.481	

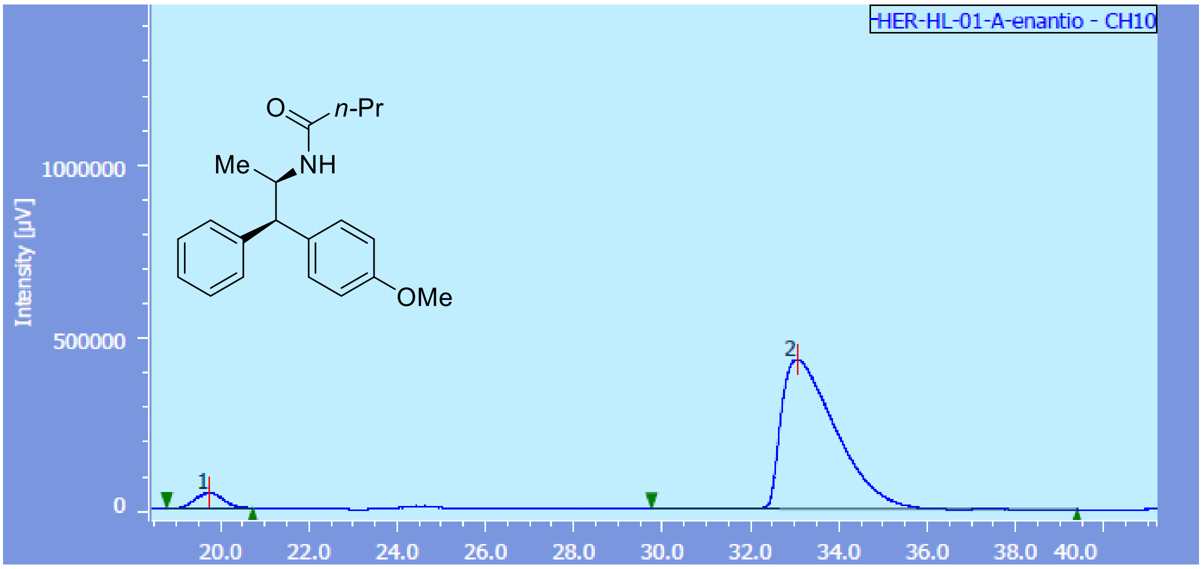

#	Peak Name	CH	tR [min]	Area [µV·sec]	Height [µV]	Area%	Height%	Quantity	NTP	Resolution	Symmetry Factor	Warning
1	Unknown	10	19.747	1910827	42968	4.895	9.063	N/A	4224	7.631	1.096	
2	Unknown	10	33.043	37128177	431147	95.105	90.937	N/A	3364	N/A	2.293	

N-[(1S,2R)-1-(4-Bromophenyl)-1-(4-methoxyphenyl)propan-2-yl]butyramide (2.11)

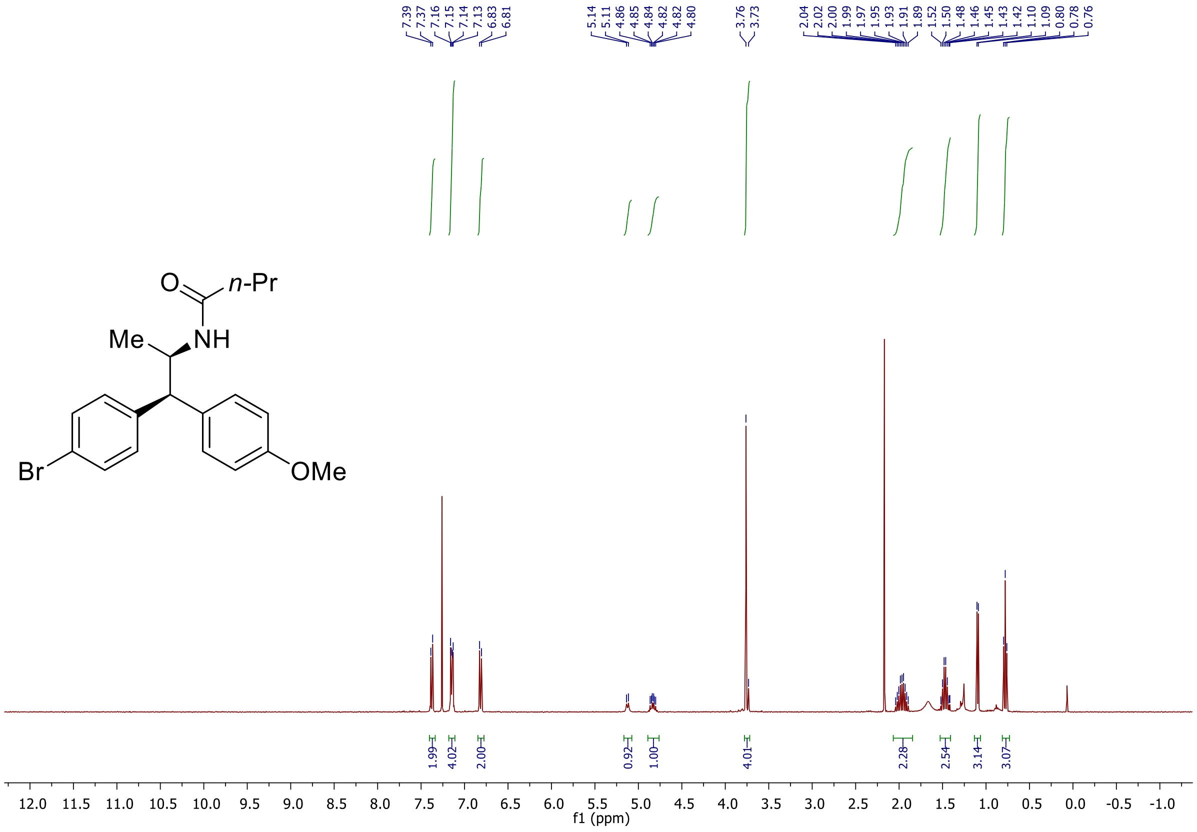

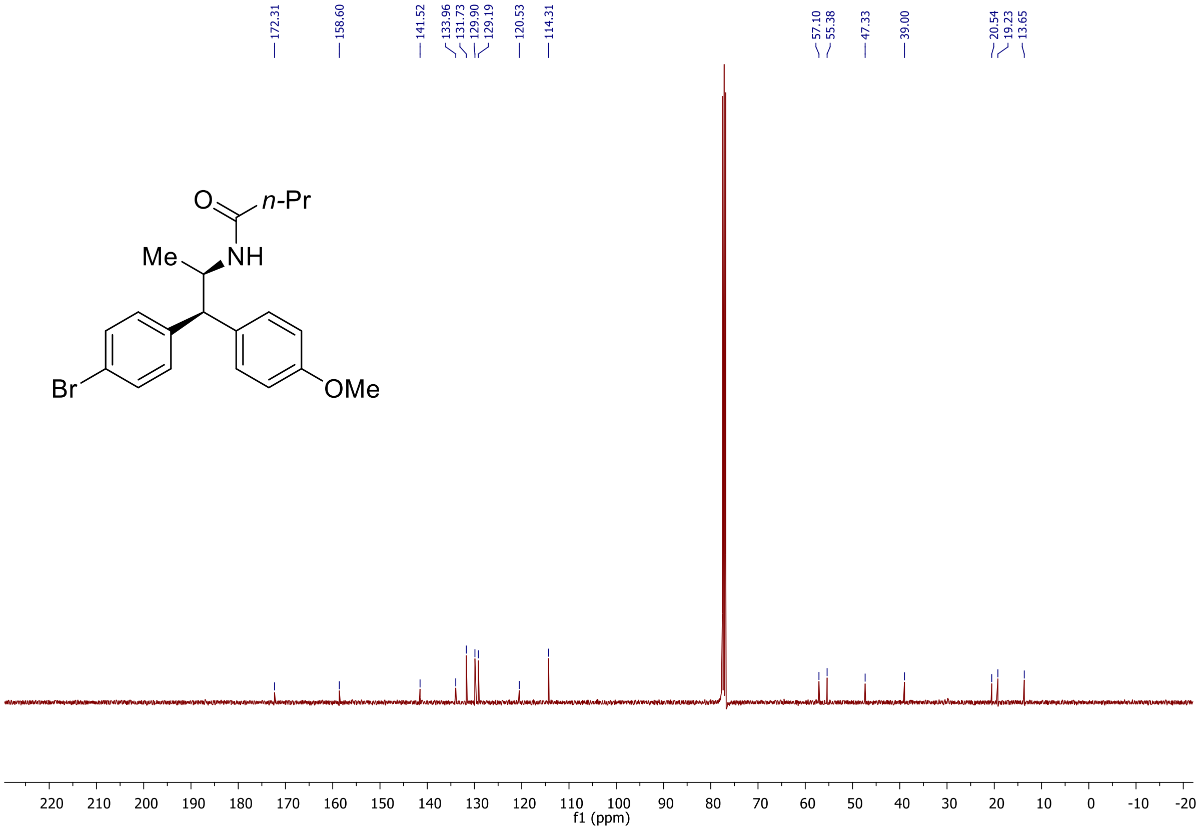

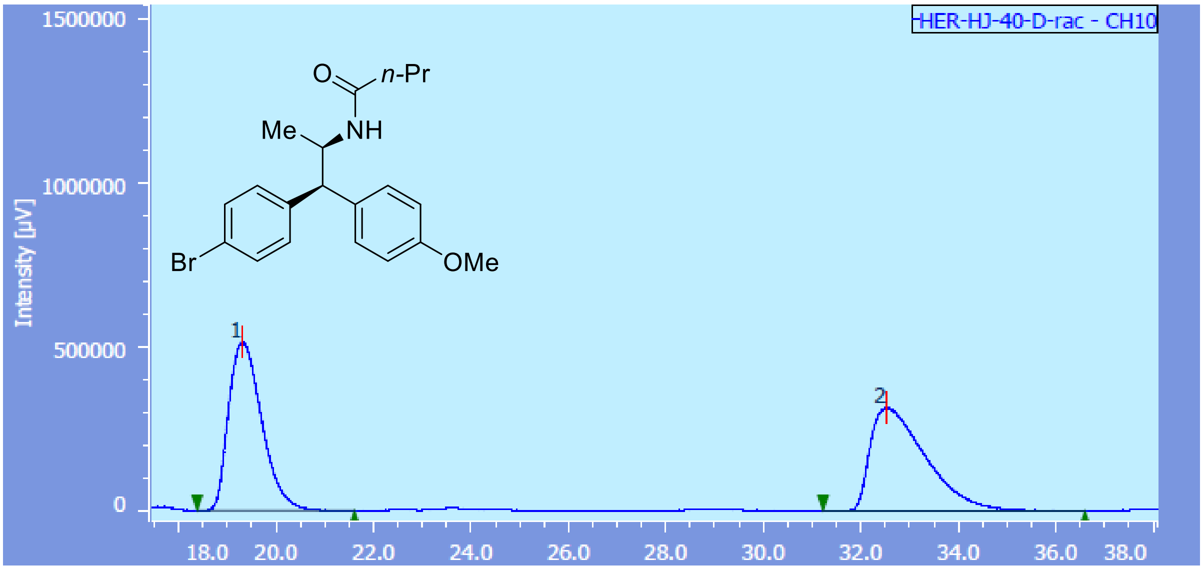

#	Peak Name	CH	tR [min]	Area [µV·sec]	Height [µV]	Area%	Height%	Quantity	NTP	Resolution	Symmetry Factor	Warning
1	Unknown	10	19.310	24365576	512823	49.615	62.032	N/A	3741	7.937	1.412	
2	Unknown	10	32.527	24743487	313885	50.385	37.968	N/A	3927	N/A	2.137	

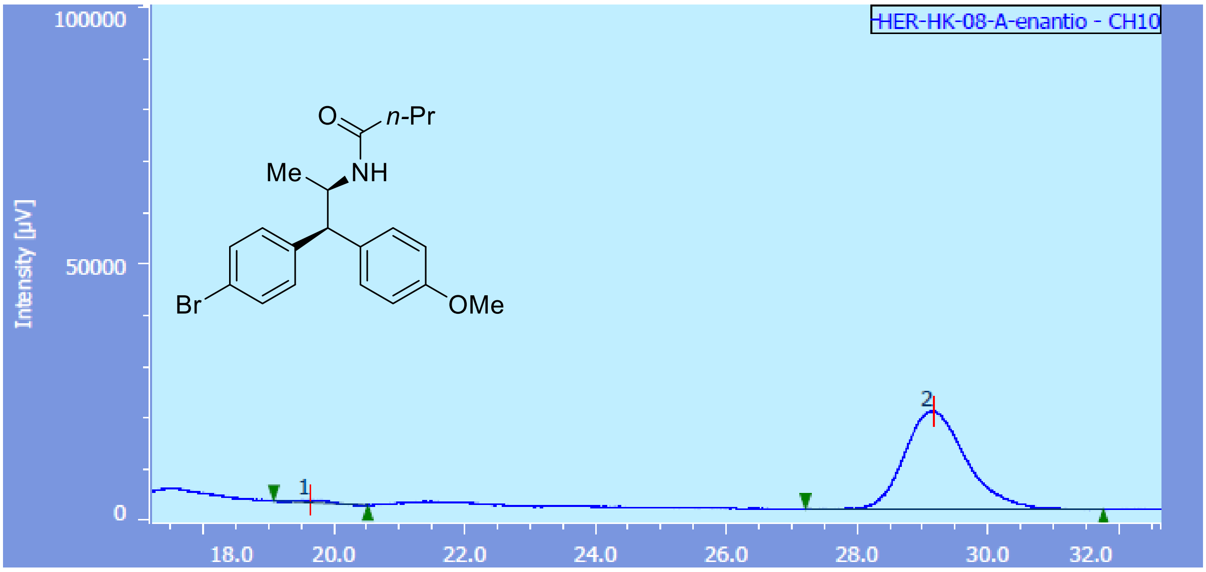

#	Peak Name	CH	tR [min]	Area [µV·sec]	Height [µV]	Area%	Height%	Quantity	NTP	Resolution	Symmetry Factor	Warning
1	Unknown	10	19.650	14683	509	1.155	2.591	N/A	9661	7.703	1.337	
2	Unknown	10	29.167	1256404	19156	98.845	97.409	N/A	4836	N/A	1.229	

N-[(1*S*,2*R*)-1-(4-Fluorophenyl)-1-(4-methoxyphenyl)propan-2-yl]butyramide (2.12)

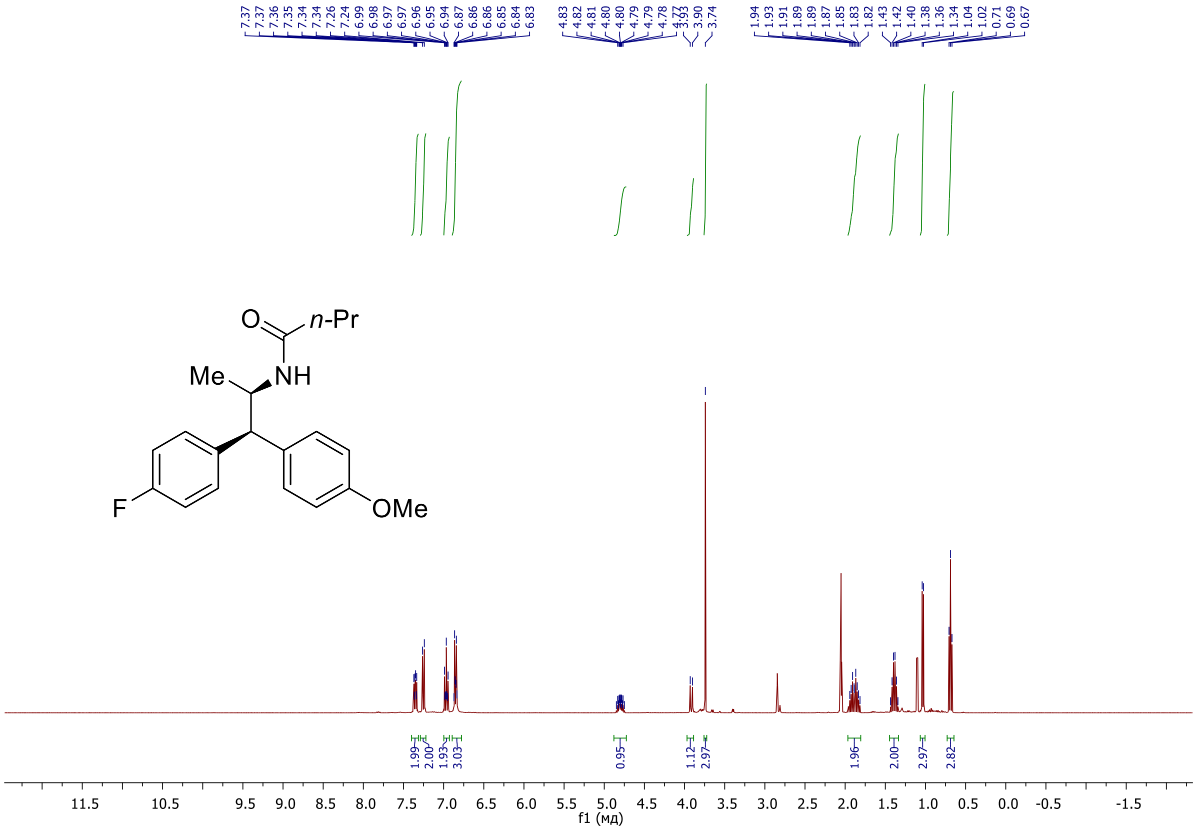

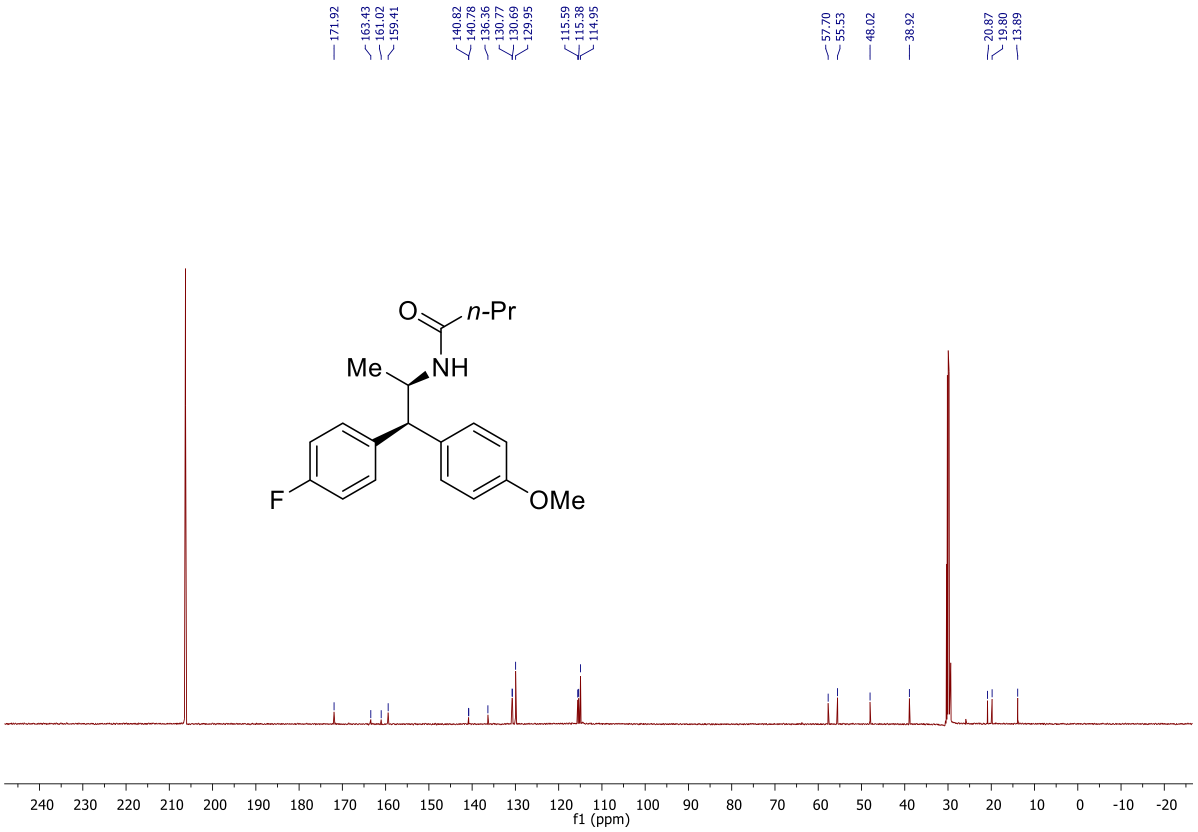

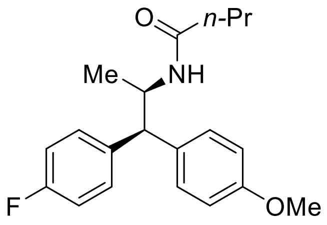
n-Pr
O
NH
Me
F
OMe

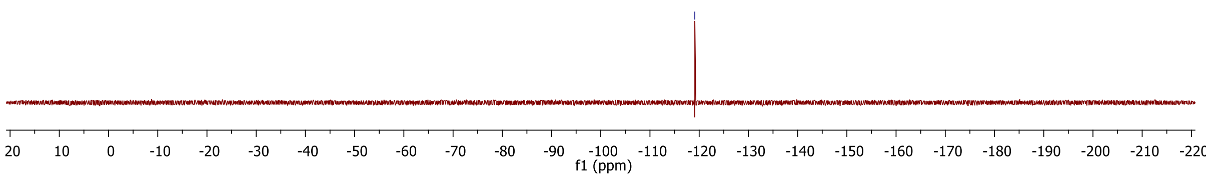
-119.12
20
10
0
-10
-20
-30
-40
-50
-60
-70
-80
-90
-100
-110
-120
-130
-140
-150
-160
-170
-180
-190
-200
-210
f1 (ppm)

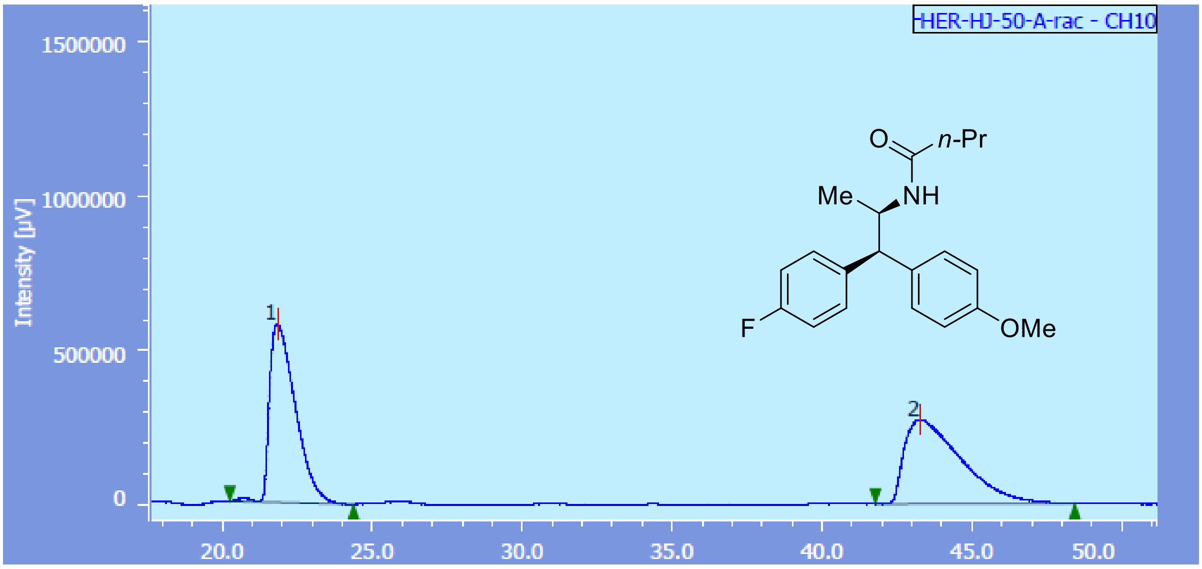

#	Peak Name	CH	tR [min]	Area [µV·sec]	Height [µV]	Area%	Height%	Quantity	NTP	Resolution	Symmetry Factor	Warning
1	Unknown	10	21.837	34643922	577148	49.236	68.033	N/A	3009	8.402	2.084	
2	Unknown	10	43.263	35718751	271186	50.764	31.967	N/A	2414	N/A	2.461	

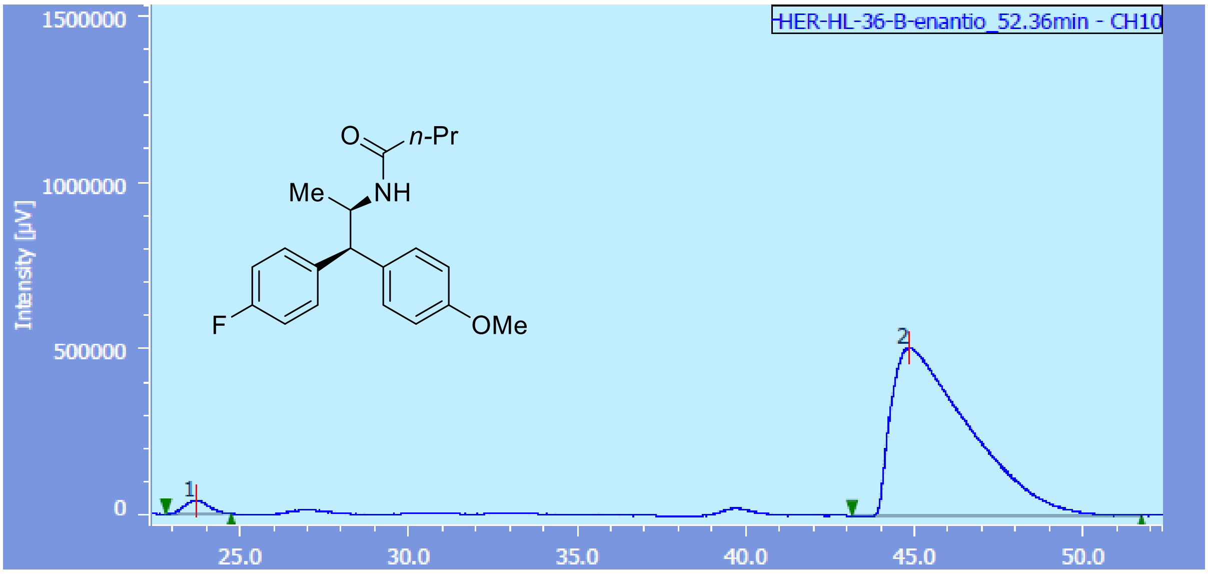

#	Peak Name	CH	tR [min]	Area [µV·sec]	Height [µV]	Area%	Height%	Quantity	NTP	Resolution	Symmetry Factor	Warning
1	Unknown	10	23.707	2094638	40122	2.528	7.378	N/A	4515	7.435	1.130	
2	Unknown	10	44.843	80760761	503664	97.472	92.622	N/A	1748	N/A	2.981	

(*S*)-3-[(*tert*-Butyldimethylsilyl)oxy)-*N*-((1*R*,2*R*)-1-(4-methoxyphenyl)-1-(4-(trifluoromethyl)phenyl)propan-2-yl]butanamide (2.13)

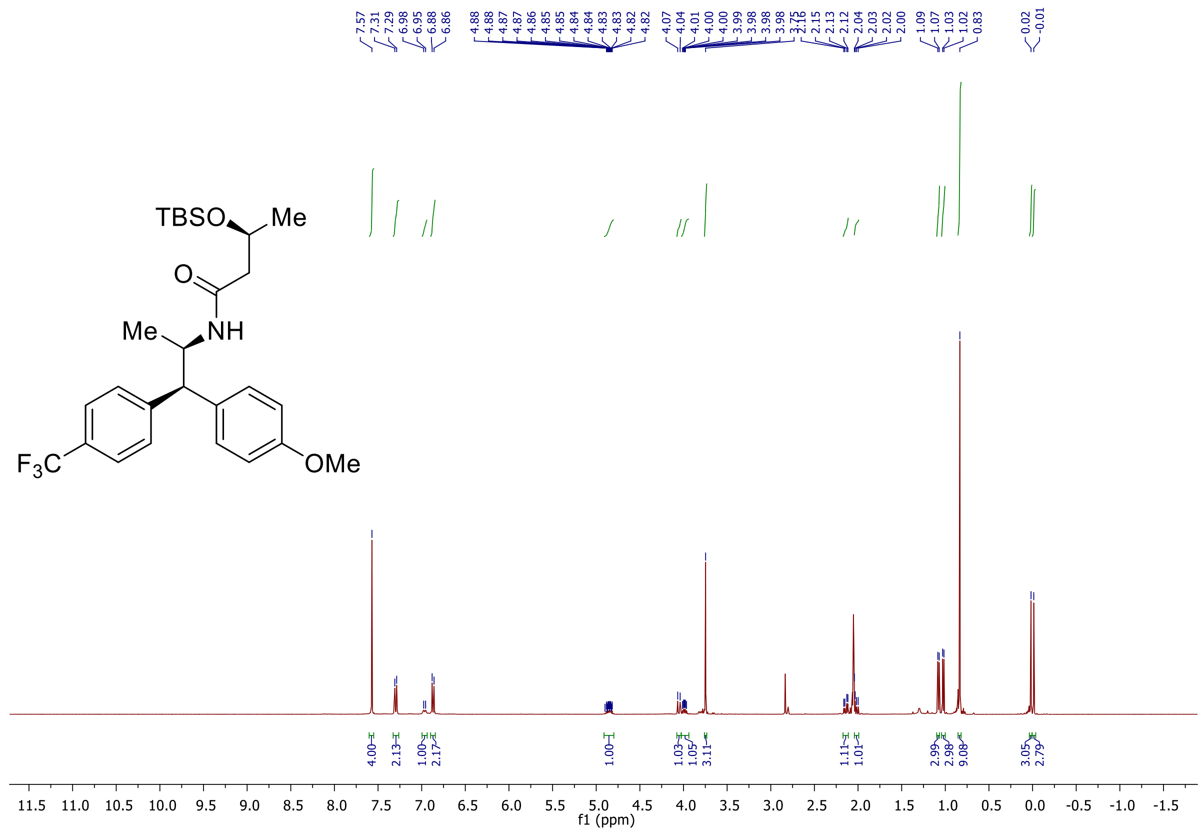

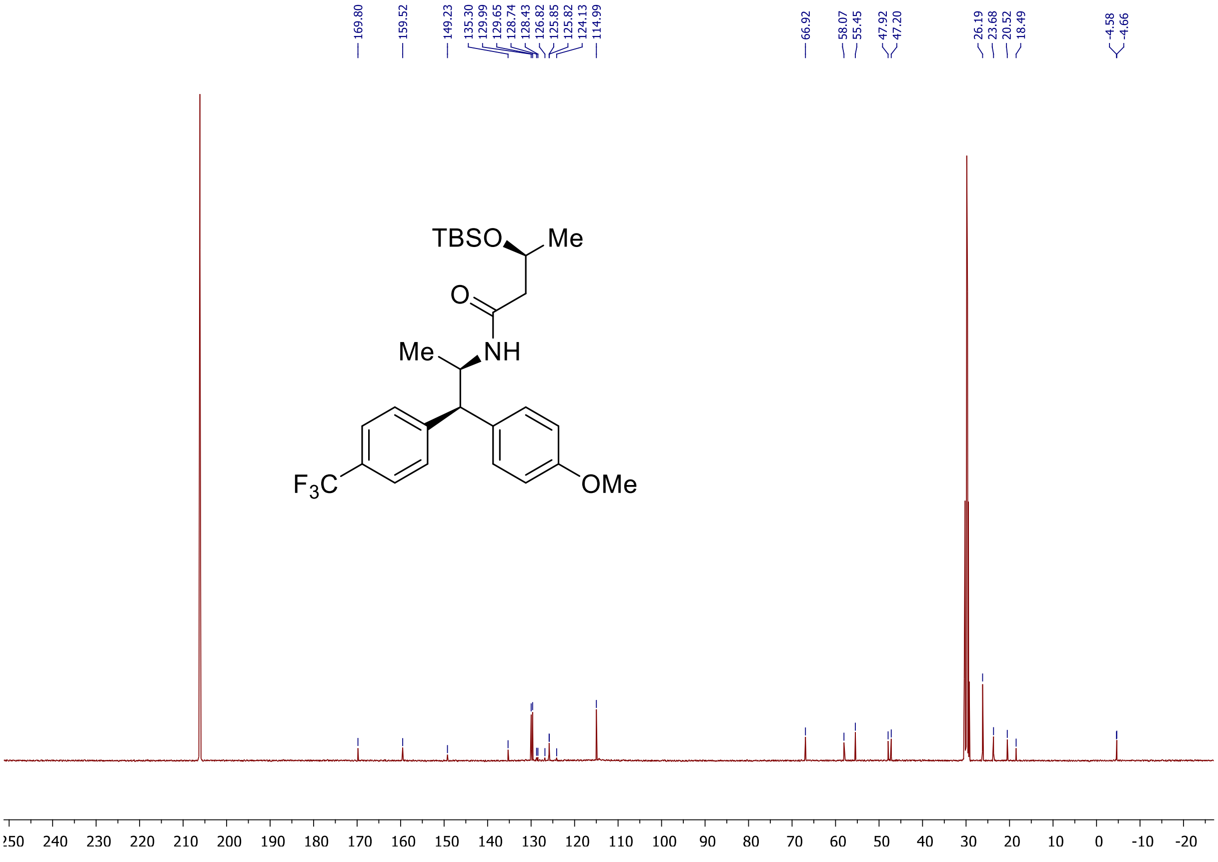

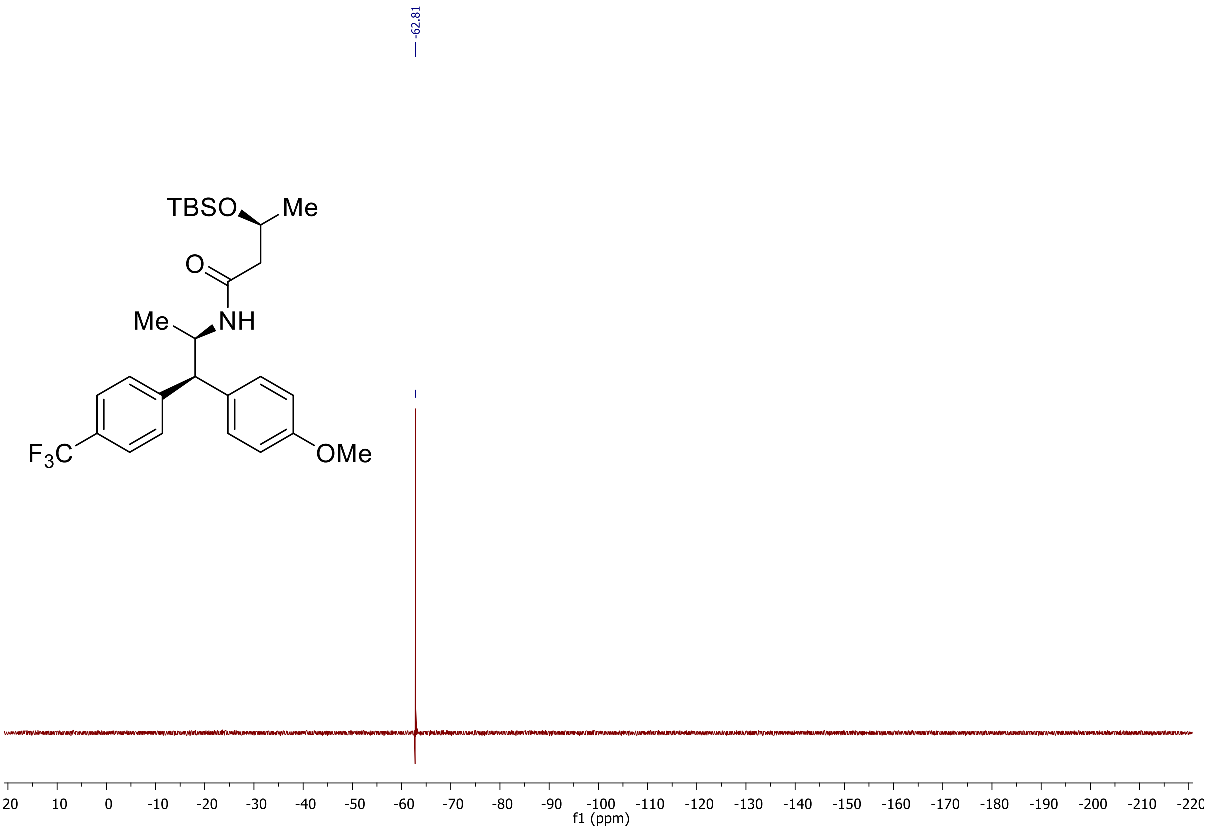
-62.81
TBSO
Me
O
Me
NH
F3C
OMe
20 10 0 -10 -20 -30 -40 -50 -60 -70 -80 -90 -100 -110 -120 -130 -140 -150 -160 -170 -180 -190 -200 -210 -220
f1 (ppm)

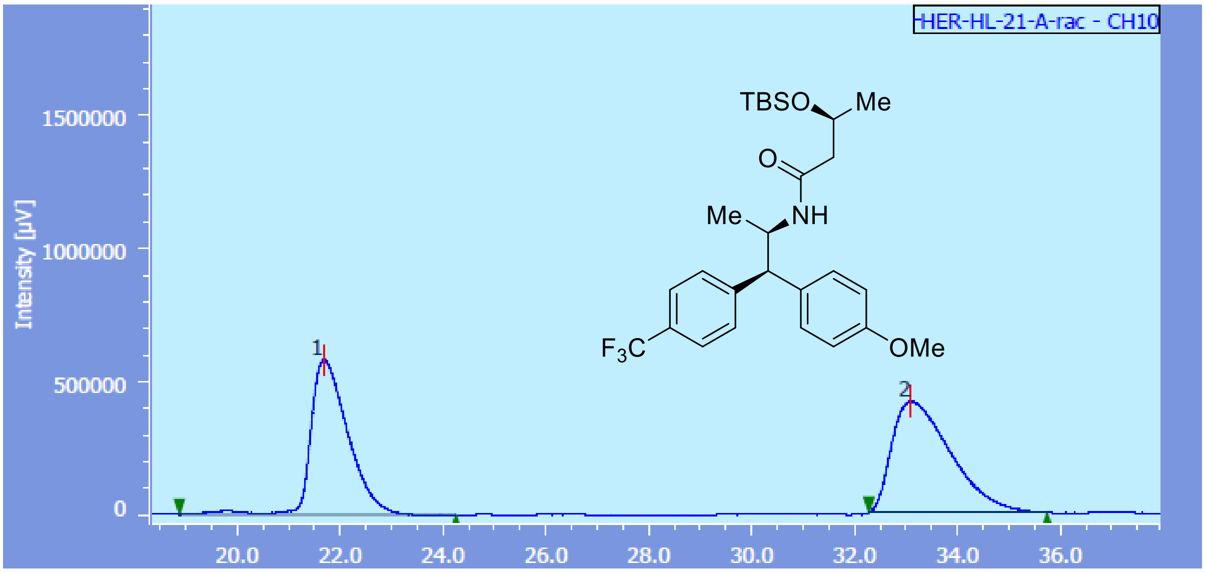

#	Peak Name	CH	tR [min]	Area [µV·sec]	Height [µV]	Area%	Height%	Quantity	NTP	Resolution	Symmetry Factor	Warning
1	Unknown	10	21.683	29259424	579920	46.919	58.177	N/A	4571	6.726	1.686	
2	Unknown	10	33.093	33102633	416897	53.081	41.823	N/A	3902	N/A	1.844	

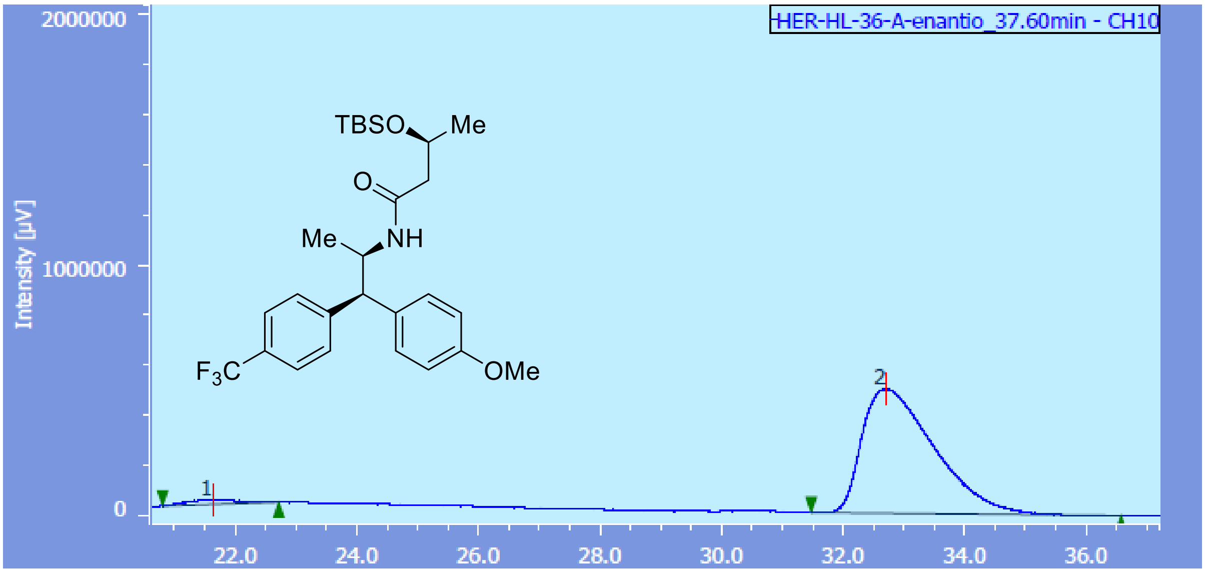

#	Peak Name	CH	tR [min]	Area [µV·sec]	Height [µV]	Area%	Height%	Quantity	NTP	Resolution	Symmetry Factor	Warning
1	Unknown	10	21.637	1055336	17514	2.525	3.427	N/A	2663	5.737	1.129	
2	Unknown	10	32.707	40735981	493503	97.475	96.573	N/A	3560	N/A	1.836	

_N_-[(1_R_,2_R_)-1-Cyclohexyl-2-(4-methoxyphenyl)-2-(3,4,5-trimethoxyphenyl)ethyl]butyramide (2.14)

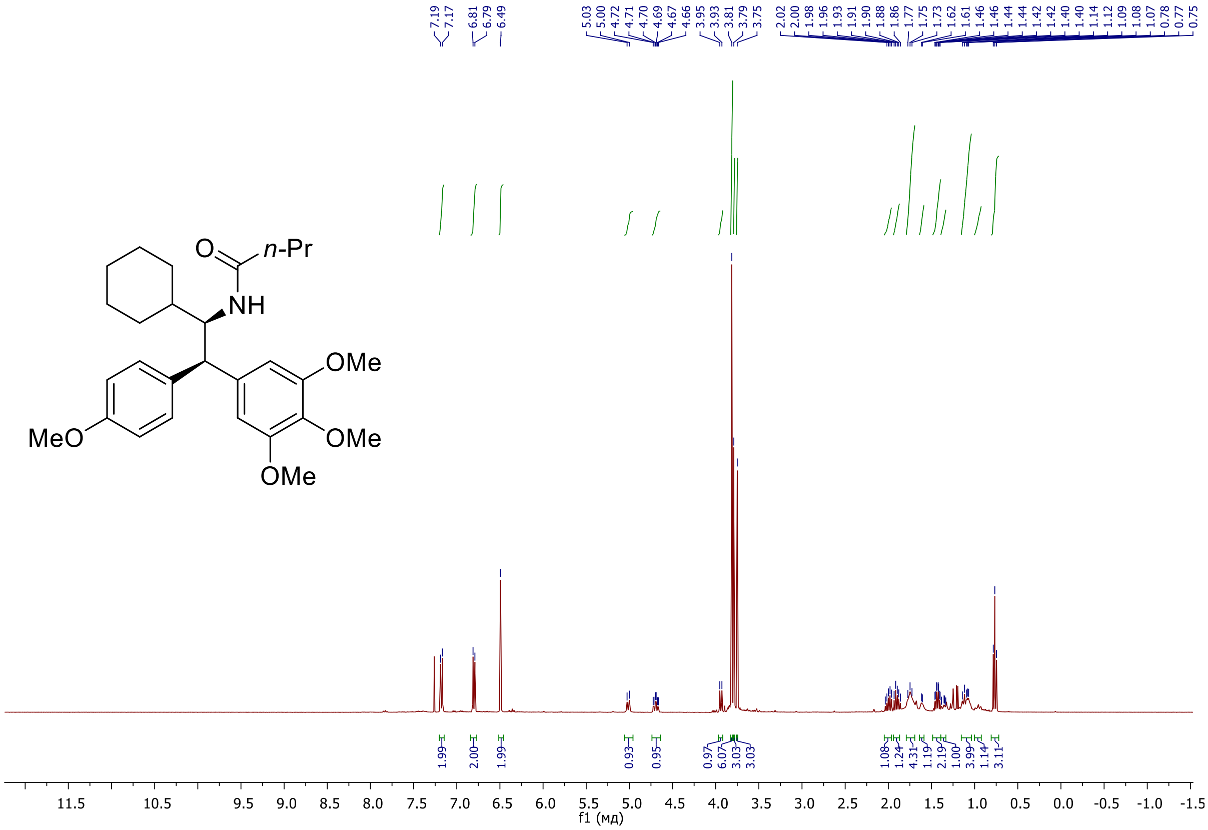

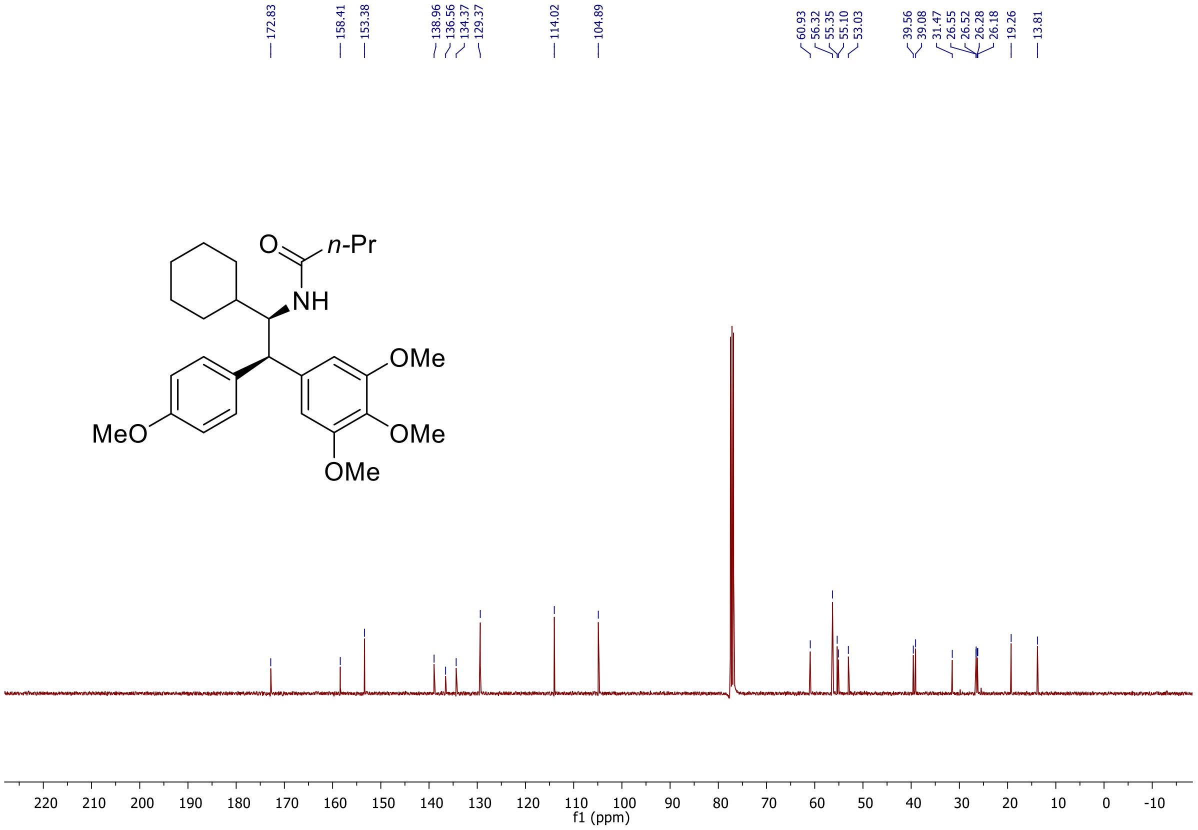

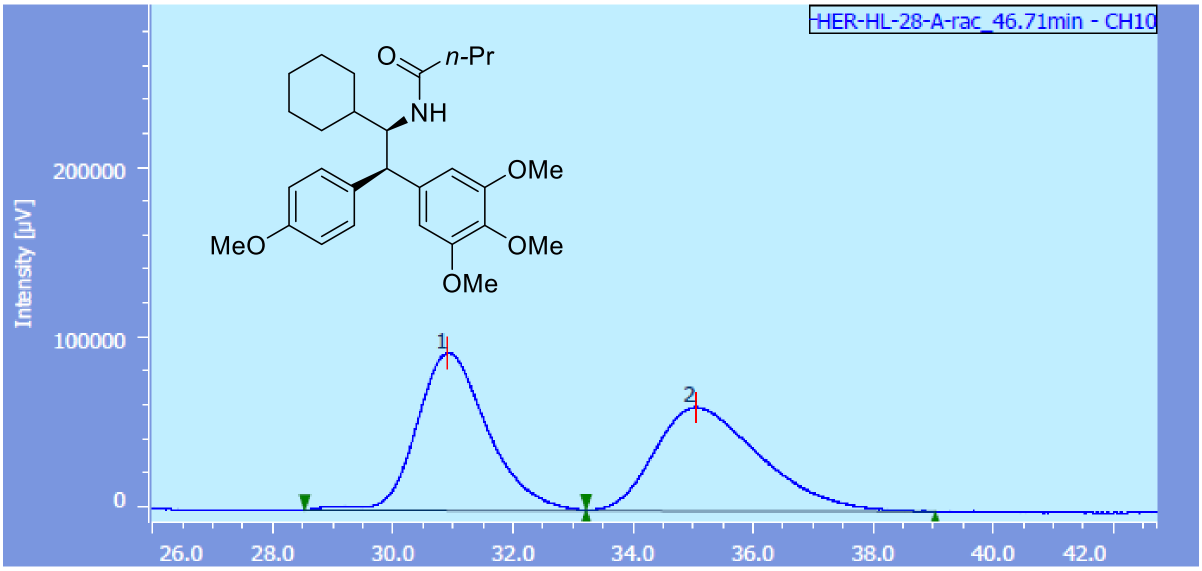

#	Peak Name	CH	tR [min]	Area [µV·sec]	Height [µV]	Area%	Height%	Quantity	NTP	Resolution	Symmetry Factor	Warning
1	Unknown	10	30.920	7602436	92815	50.552	60.345	N/A	3509	1.563	1.261	
2	Unknown	10	35.053	7436420	60992	49.448	39.655	N/A	1902	N/A	1.424	

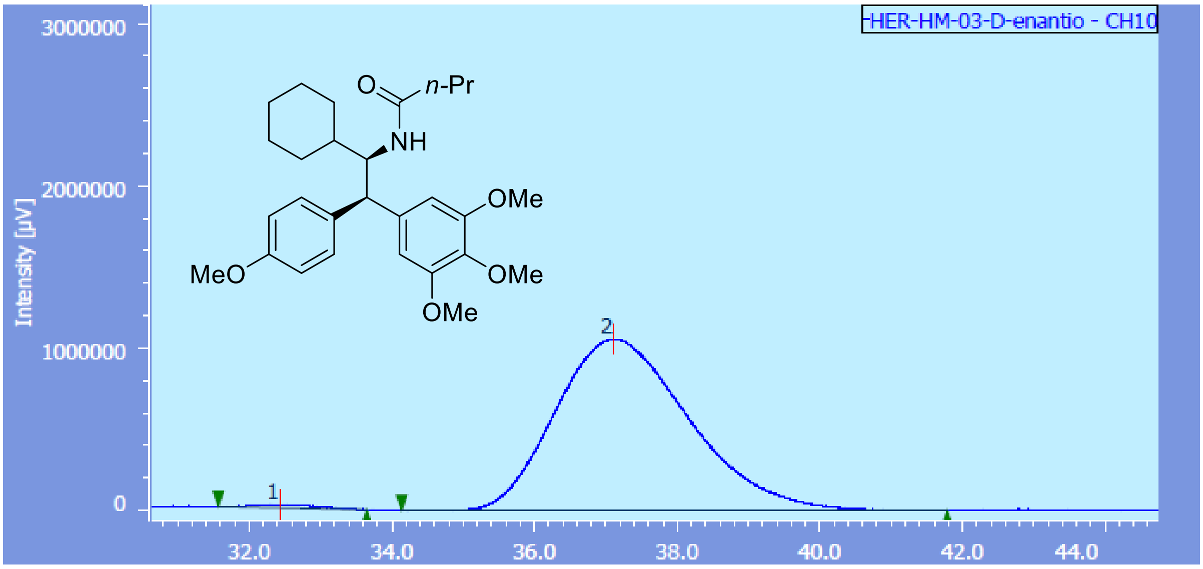

#	Peak Name	CH	tR [min]	Area [µV·sec]	Height [µV]	Area%	Height%	Quantity	NTP	Resolution	Symmetry Factor	Warning
1	Unknown	10	32.427	1334711	21073	0.964	1.957	N/A	5529	1.808	1.205	
2	Unknown	10	37.103	137151354	1055774	99.036	98.043	N/A	1858	N/A	1.279	

(*S*)-3-[(*tert*-Butyldimethylsilyl)oxy)-*N*-((1*S*,2*R*)-1-(3-methoxyphenyl)-1-(4-methoxyphenyl)propan-2-yl]butanamide (2.15)

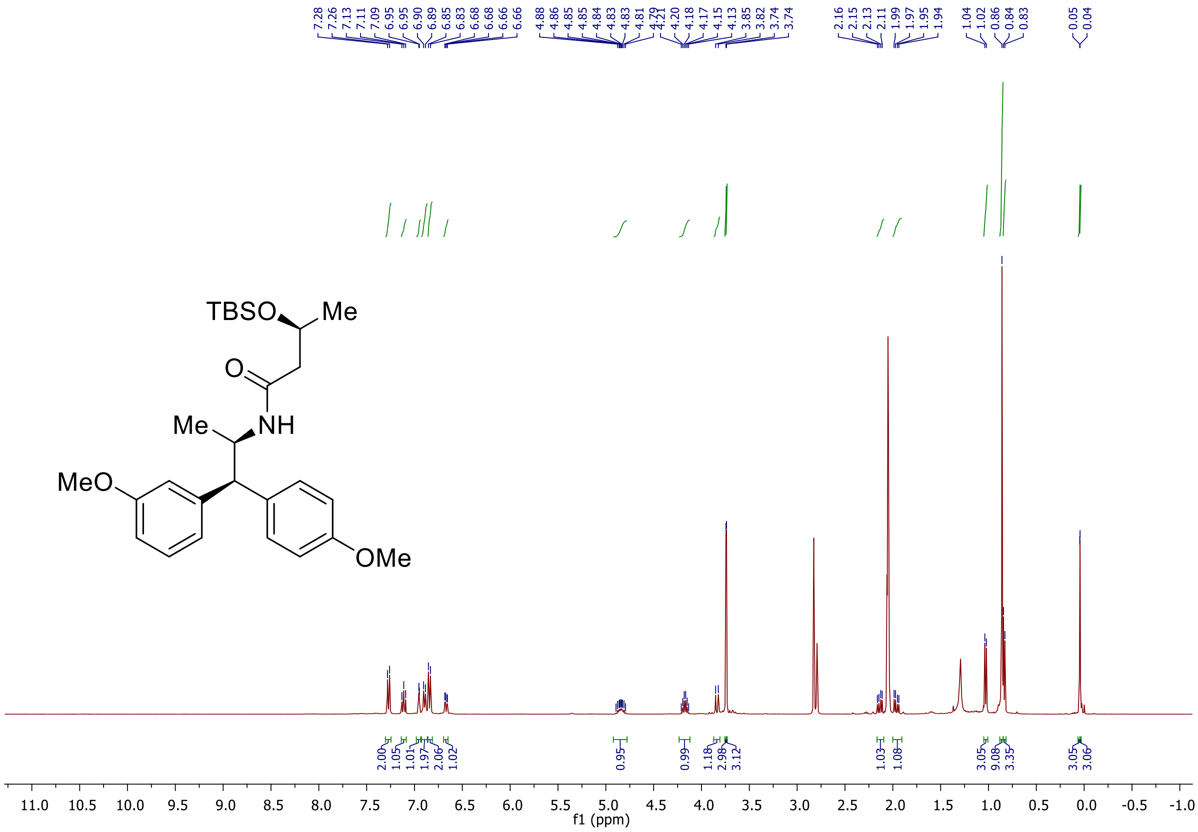

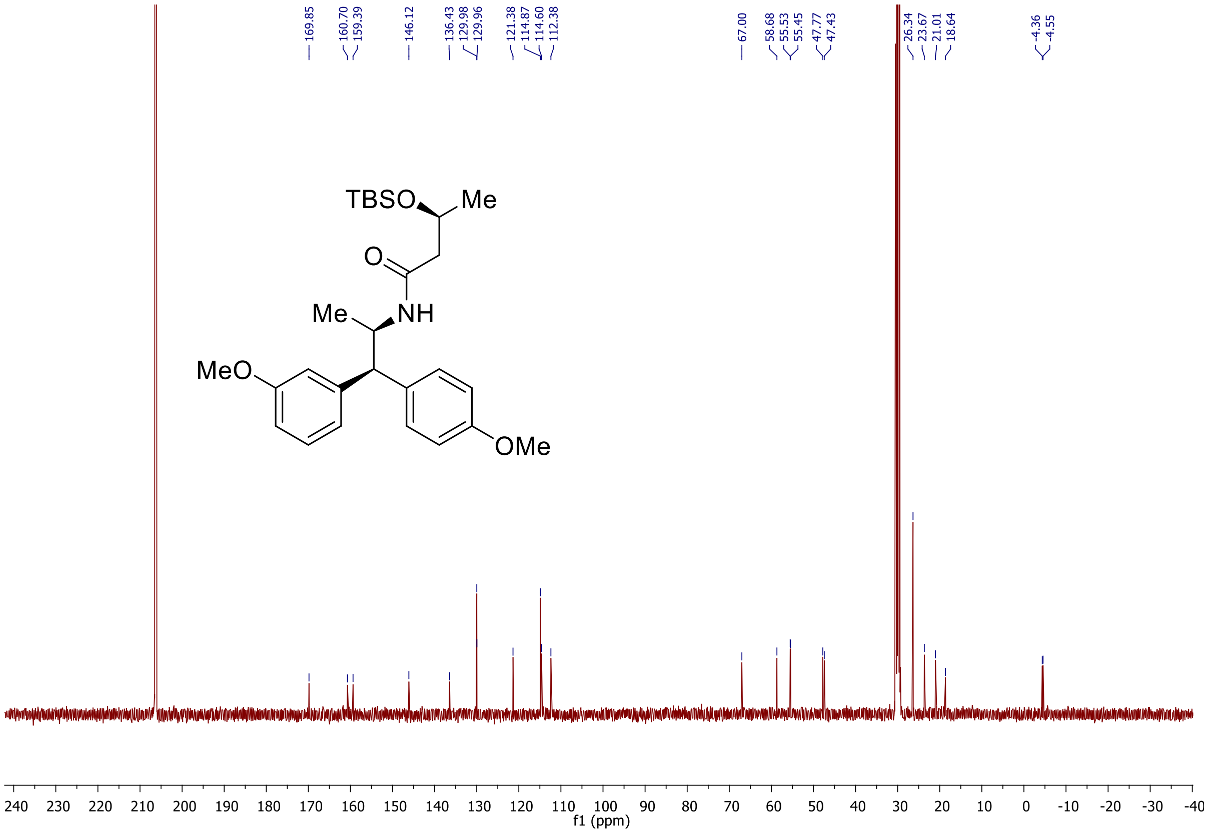

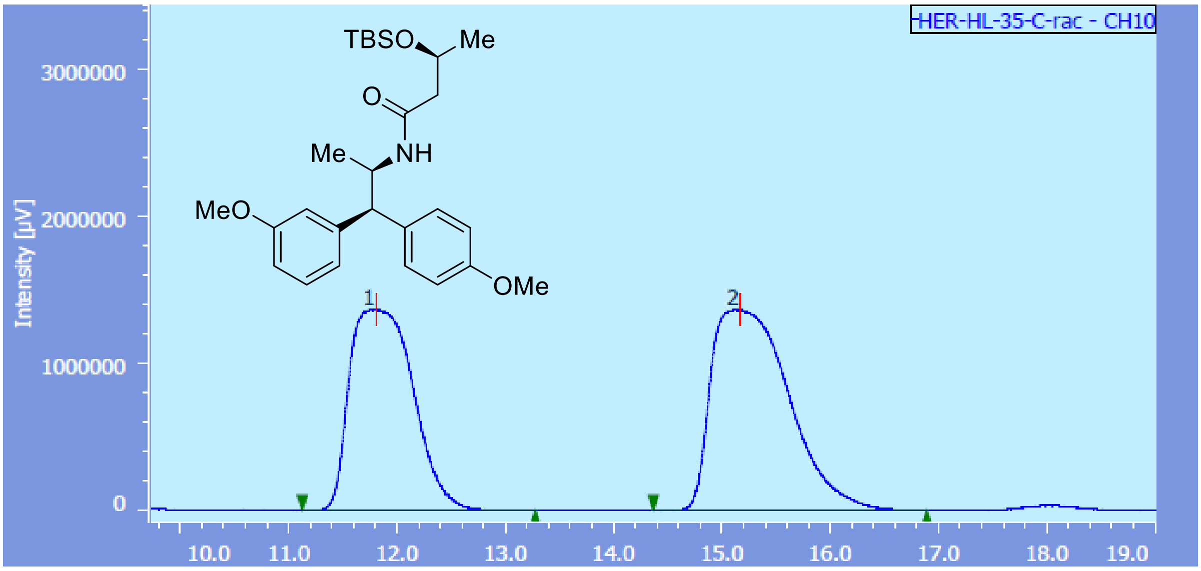

#	Peak Name	CH	tR [min]	Area [µV·sec]	Height [µV]	Area%	Height%	Quantity	NTP	Resolution	Symmetry Factor	Warning
1	Unknown	10	11.803	55934417	1363134	44.946	50.025	N/A	1761	2.719	1.381	
2	Unknown	10	15.163	68513786	1361746	55.054	49.975	N/A	2010	N/A	1.713	

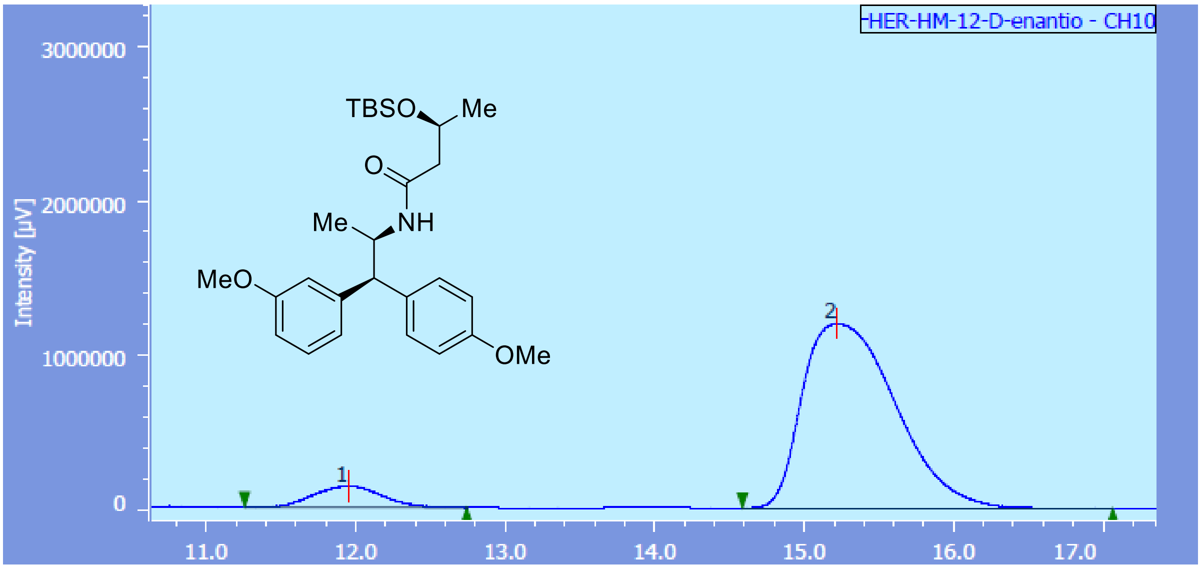

#	Peak Name	CH	tR [min]	Area [µV·sec]	Height [µV]	Area%	Height%	Quantity	NTP	Resolution	Symmetry Factor	Warning
1	Unknown	10	11.953	4362392	137780	7.786	10.334	N/A	3152	3.240	1.052	
2	Unknown	10	15.220	51669125	1195543	92.214	89.666	N/A	2706	N/A	1.534	

(*S*)-3-[(*tert*-Butyldimethylsilyl)oxy)-*N*-((1*S*,2*R*)-1-(3-bromophenyl)-1-(4-methoxyphenyl)propan-2-yl]butanamide (2.16)

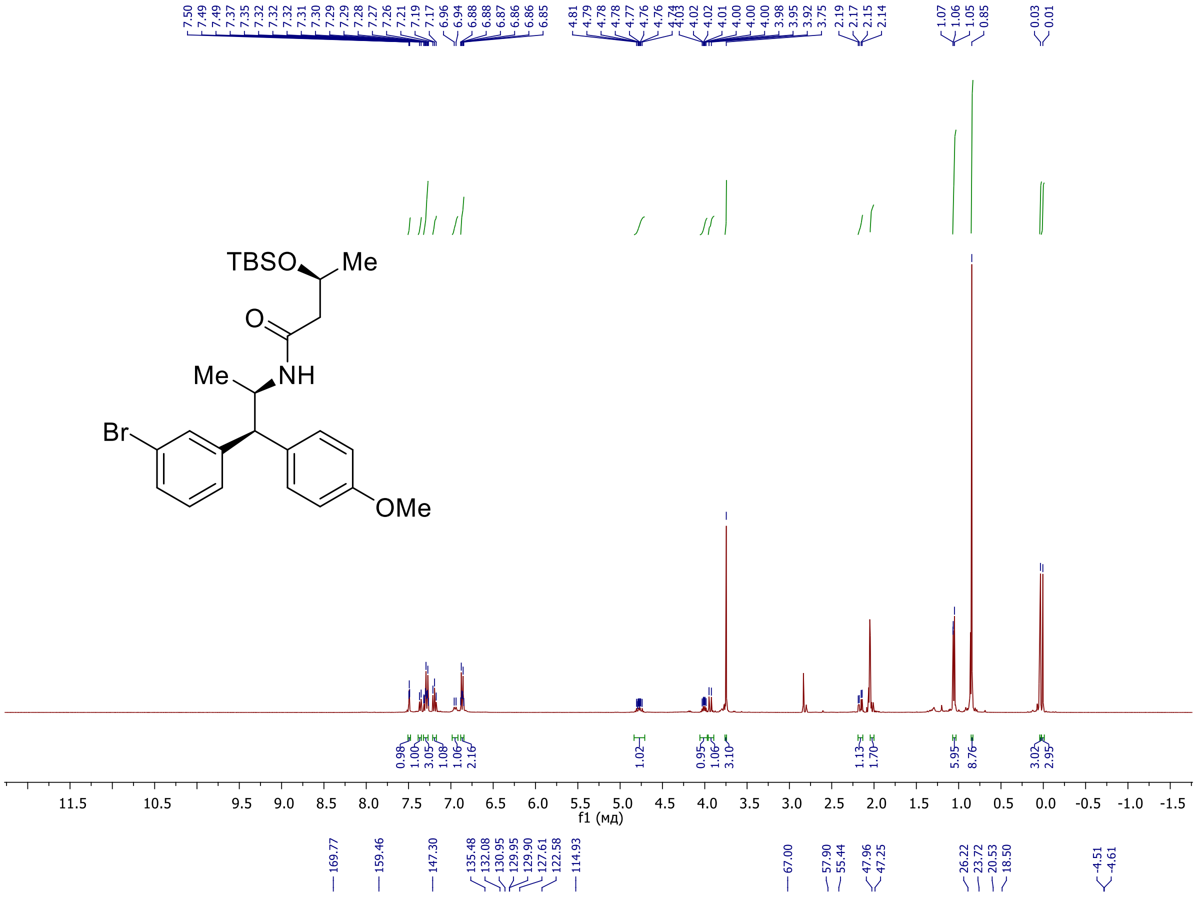

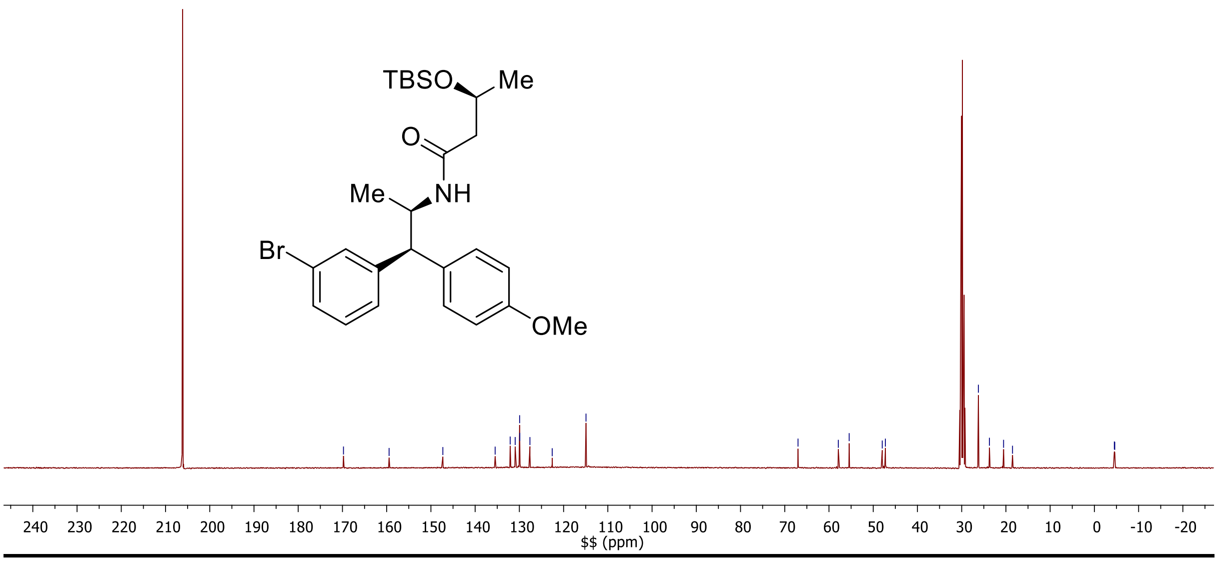

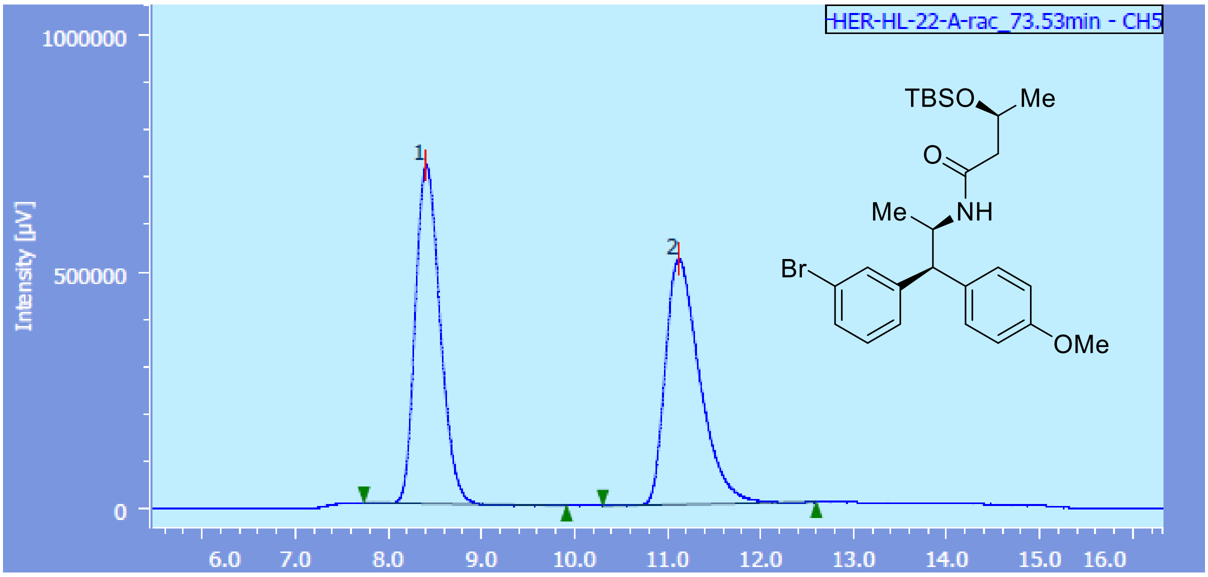

#	Peak Name	CH	tR [min]	Area [μV·sec]	Height [μV]	Area%	Height%	Quantity	NTP	Resolution	Symmetry Factor	Warning
1	Unknown	5	8.407	13165014	714568	49.700	57.933	N/A	4747	4.737	1.250	
2	Unknown	5	11.120	13324049	518866	50.300	42.067	N/A	4534	N/A	1.530	

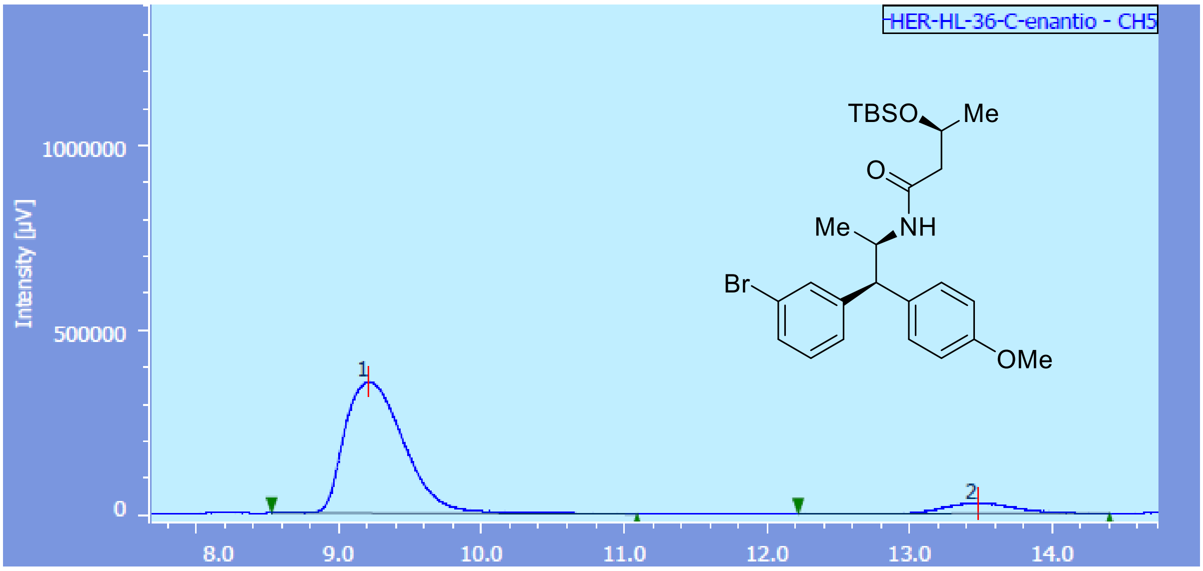

#	Peak Name	CH	tR [min]	Area [μV·sec]	Height [μV]	Area%	Height%	Quantity	NTP	Resolution	Symmetry Factor	Warning
1	Unknown	5	9.213	10340049	355049	91.065	92.710	N/A	2271	4.960	1.436	
2	Unknown	5	13.477	1014577	27917	8.935	7.290	N/A	3218	N/A	1.215	

N-[(1*S*,2*R*)-1-(4-Methoxyphenyl)-1-(*o*-tolyl)propan-2-yl]butyramide (2.17)

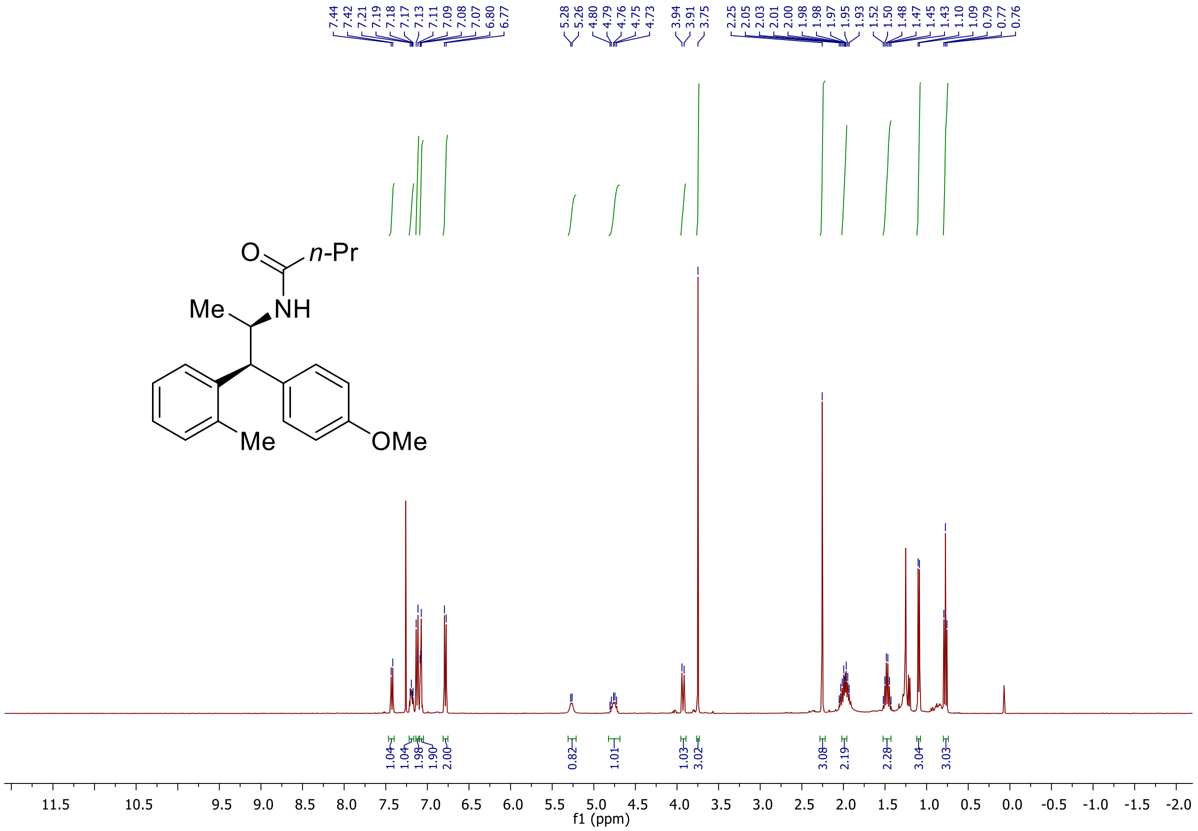

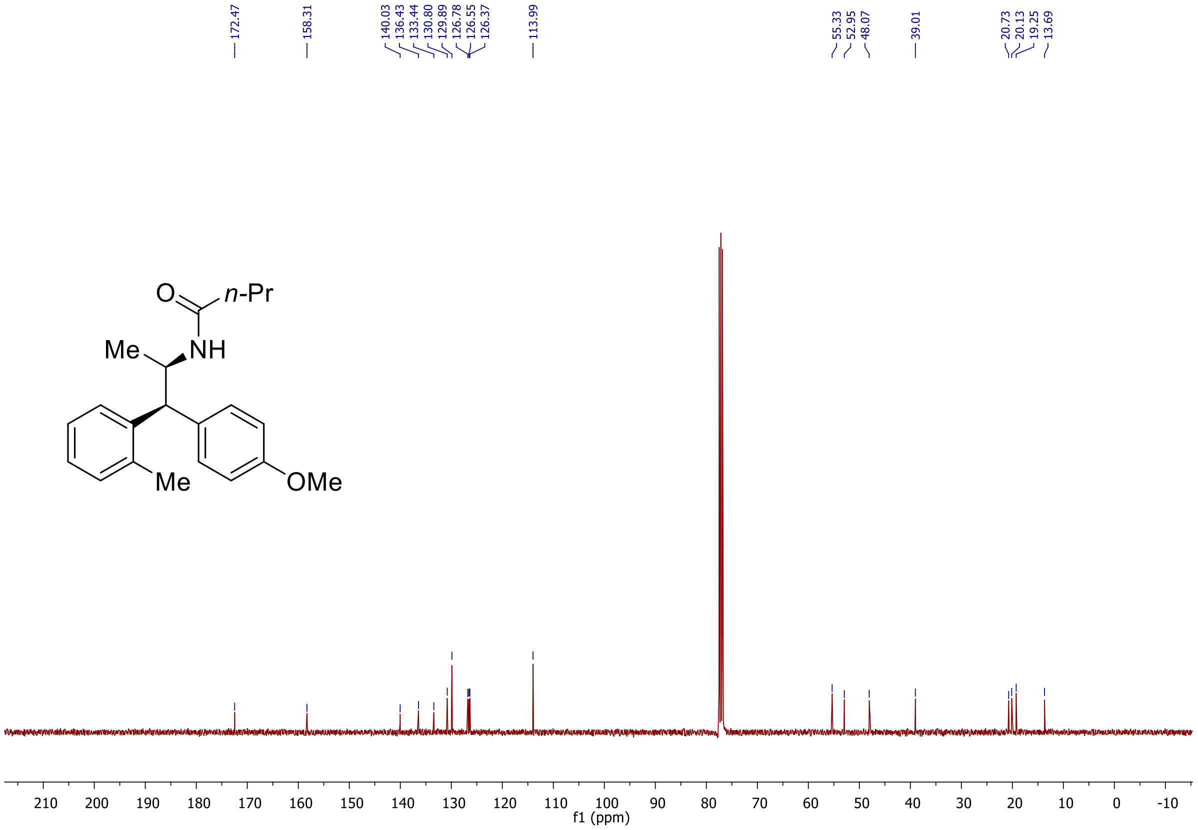

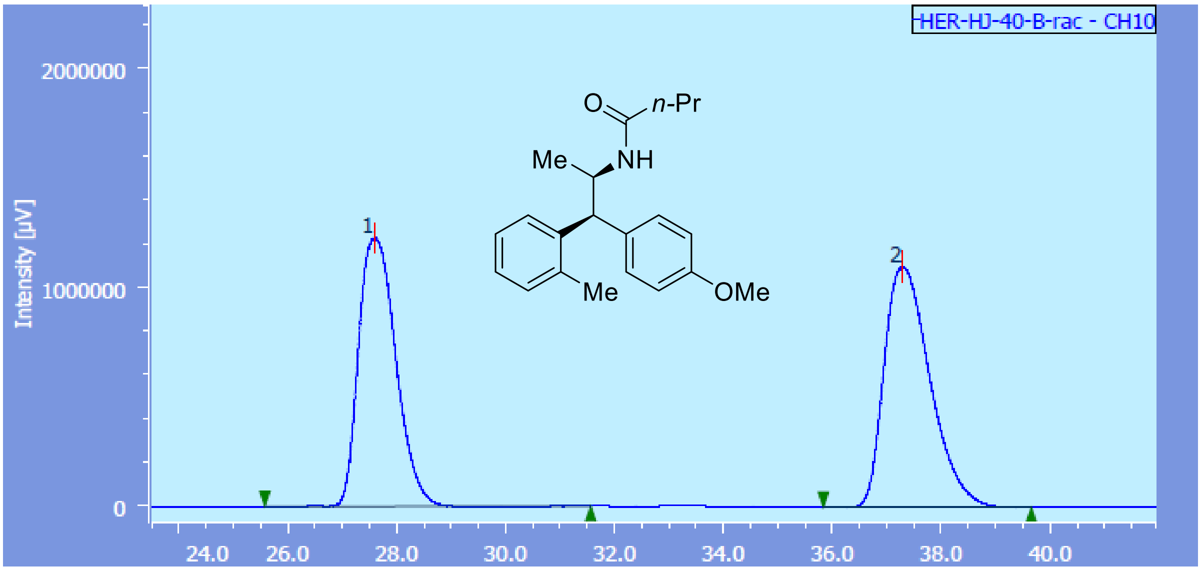

#	Peak Name	CH	tR [min]	Area [μV·sec]	Height [μV]	Area%	Height%	Quantity	NTP	Resolution	Symmetry Factor	Warning
1	Unknown	10	27.597	58438610	1227480	47.542	52.746	N/A	7521	6.899	1.306	
2	Unknown	10	37.290	64482365	1099688	52.458	47.254	N/A	9324	N/A	1.438	

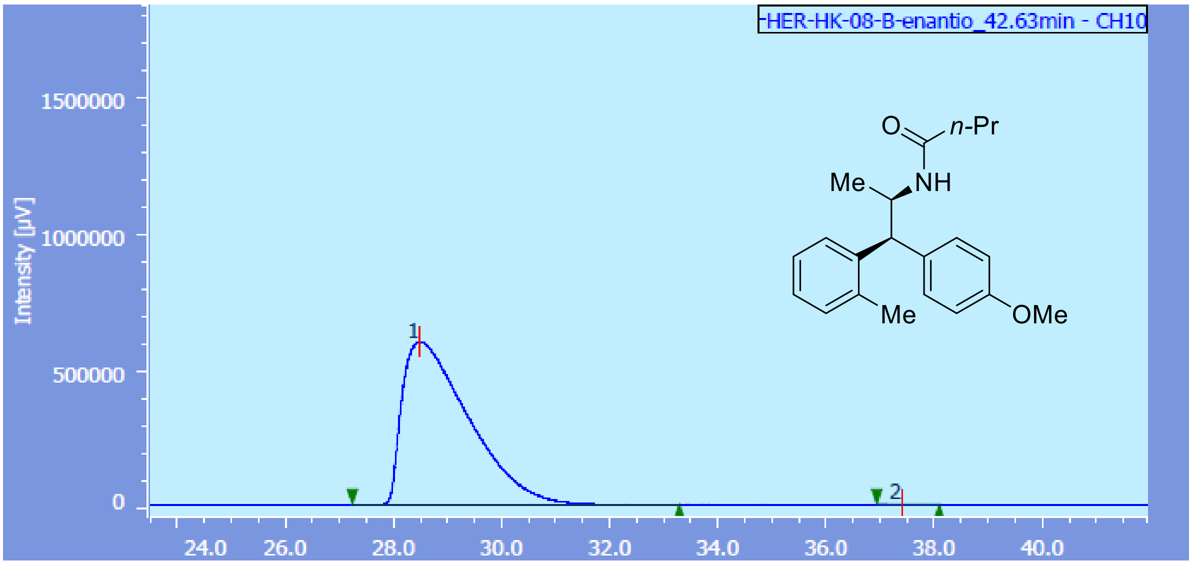

#	Peak Name	CH	tR [min]	Area [μV·sec]	Height [μV]	Area%	Height%	Quantity	NTP	Resolution	Symmetry Factor	Warning
1	Unknown	10	28.490	50755639	594052	99.946	99.870	N/A	2490	5.245	2.532	
2	Unknown	10	37.400	27417	771	0.054	0.130	N/A	17756	N/A	1.220	

***N*-[(1*S*,2*R*)-1-(2-Bromophenyl)-1-(4-methoxyphenyl)propan-2-yl]butyramide (2.18)**

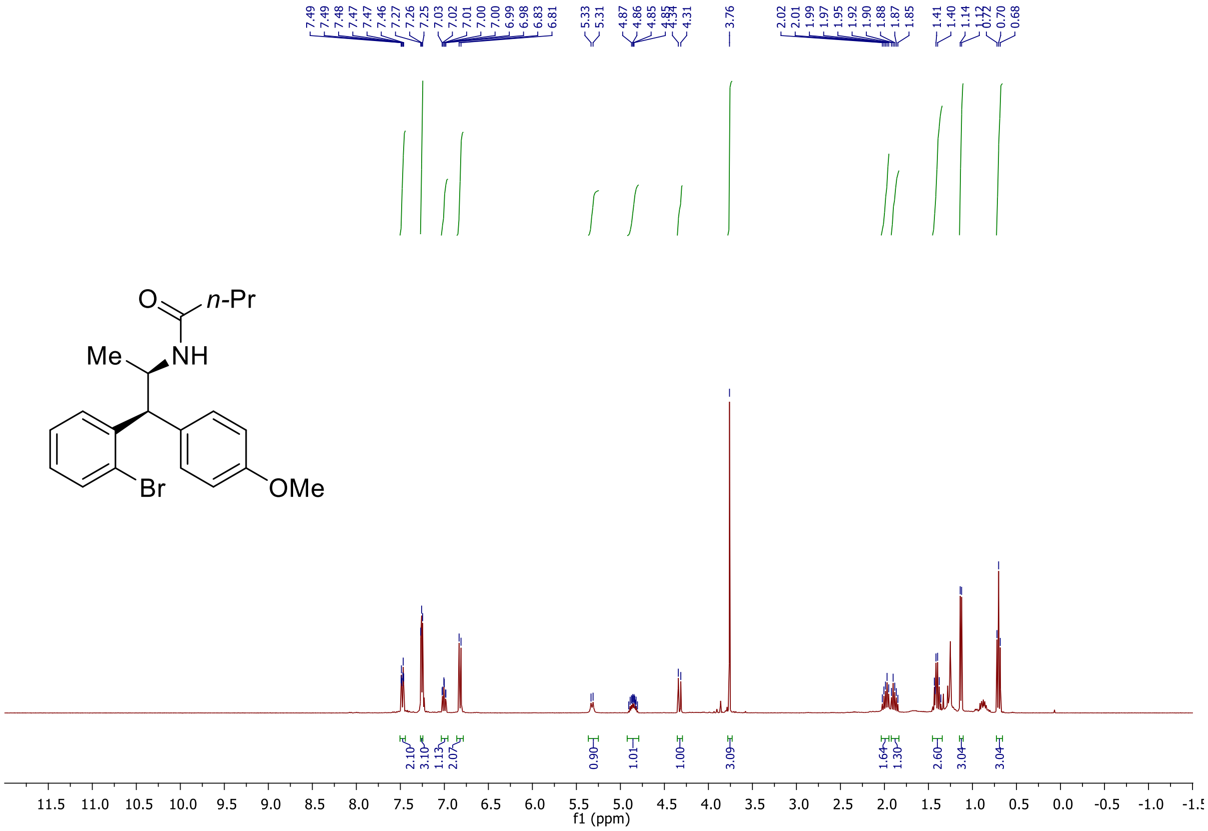

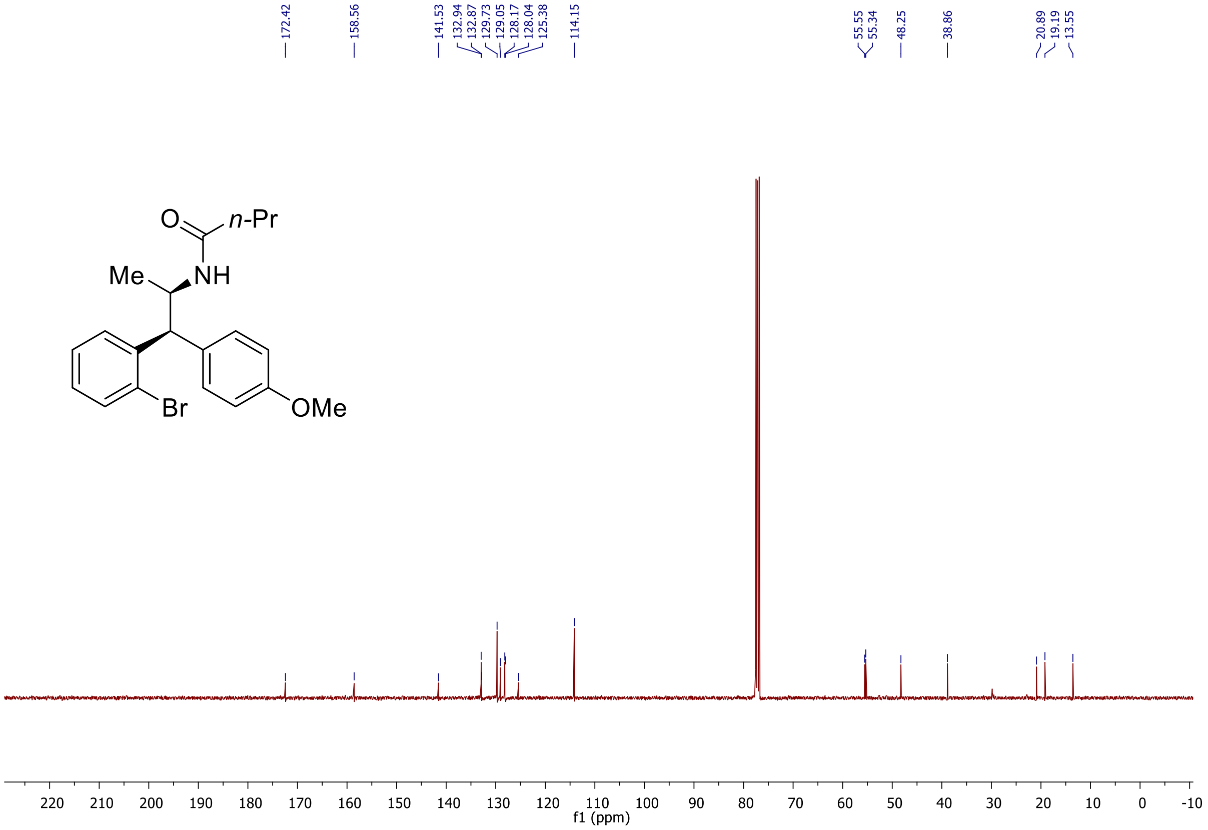

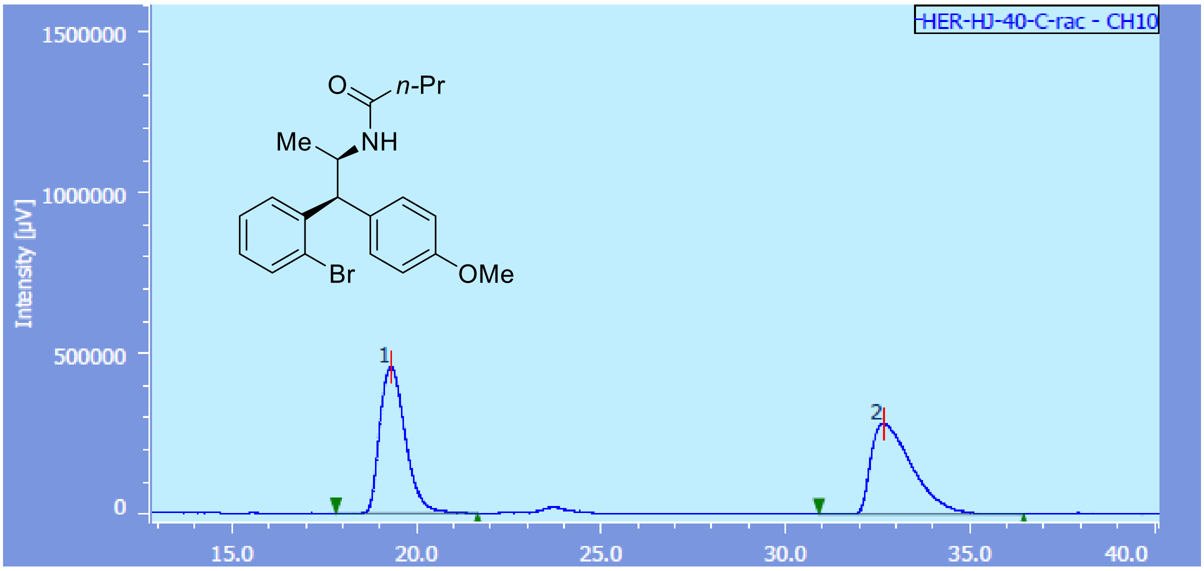

#	Peak Name	CH	tR [min]	Area [μV·sec]	Height [μV]	Area%	Height%	Quantity	NTP	Resolution	Symmetry Factor	Warning
1	Unknown	10	19.303	21927355	454152	50.113	61.829	N/A	3699	8.057	1.380	
2	Unknown	10	32.647	21828888	280374	49.887	38.171	N/A	4052	N/A	2.084	

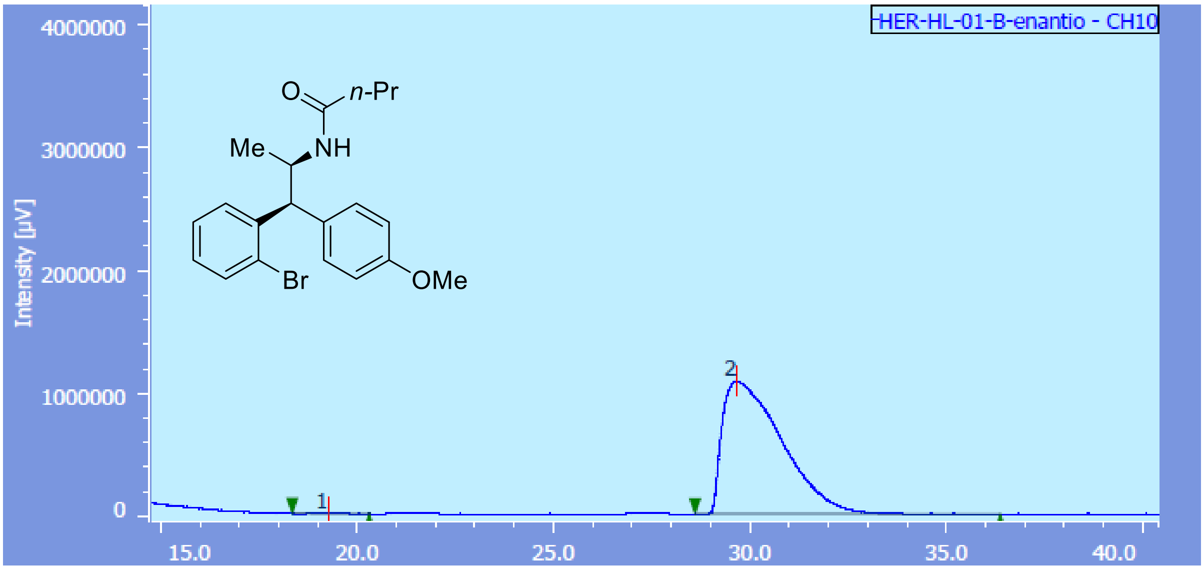

#	Peak Name	CH	tR [min]	Area [μV·sec]	Height [μV]	Area%	Height%	Quantity	NTP	Resolution	Symmetry Factor	Warning
1	Unknown	10	19.277	239167	5487	0.210	0.506	N/A	4957	5.272	1.370	
2	Unknown	10	29.663	113889976	1079375	99.790	99.494	N/A	1726	N/A	2.708	

(*S*)-3-[(*tert*-Butyldimethylsilyl)oxy]-*N*-[(1*R*,2*R*)-1-(5-chlorothiophen-2-yl)-1-(4-methoxyphenyl)propan-2-yl]butanamide (2.19)

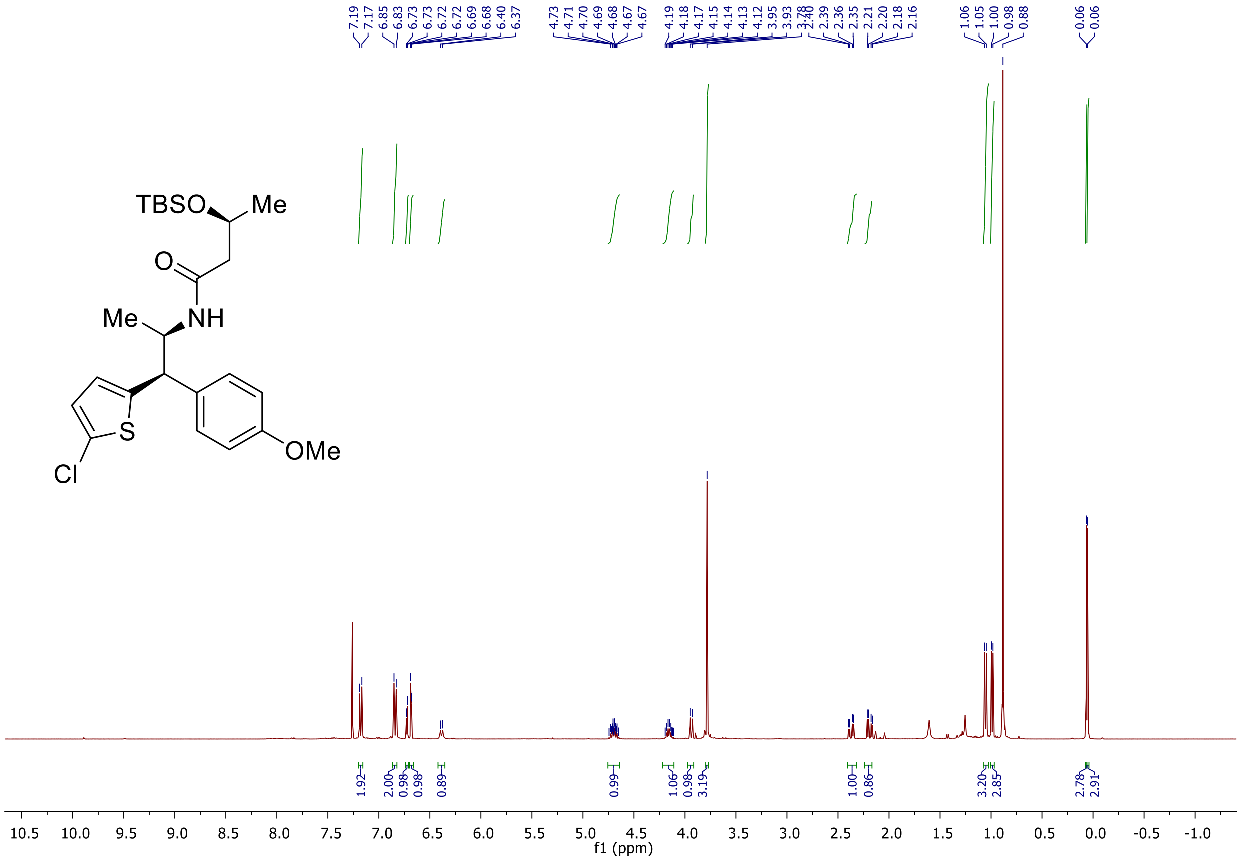

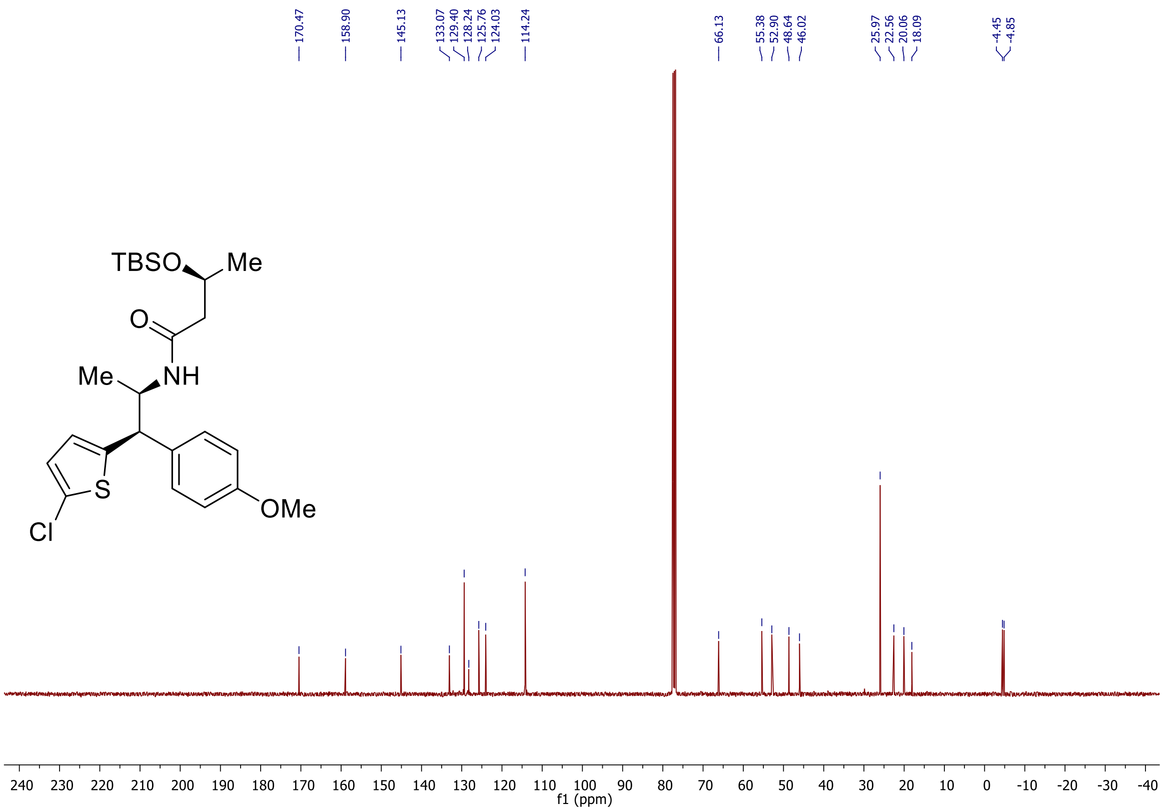

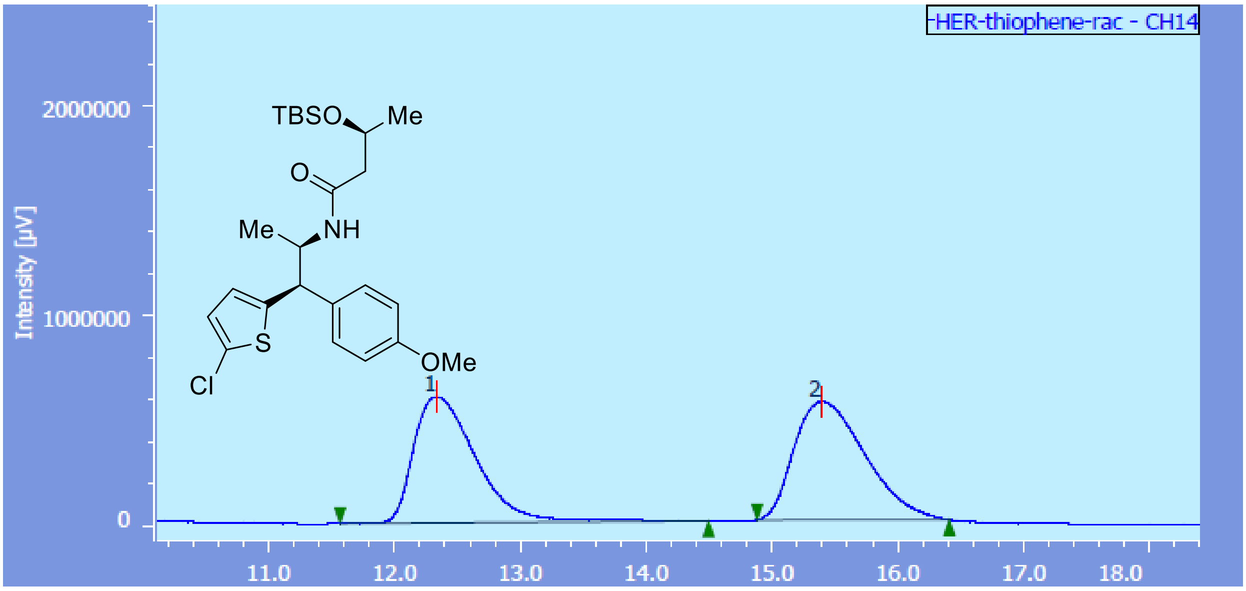

#	Peak Name	CH	tR [min]	Area [µV·sec]	Height [µV]	Area%	Height%	Quantity	NTP	Resolution	Symmetry Factor	Warning
1	Unknown	14	12.337	20614270	599918	48.288	51.610	N/A	3078	3.149	1.604	
2	Unknown	14	15.397	22075934	562493	51.712	48.390	N/A	3379	N/A	1.454	

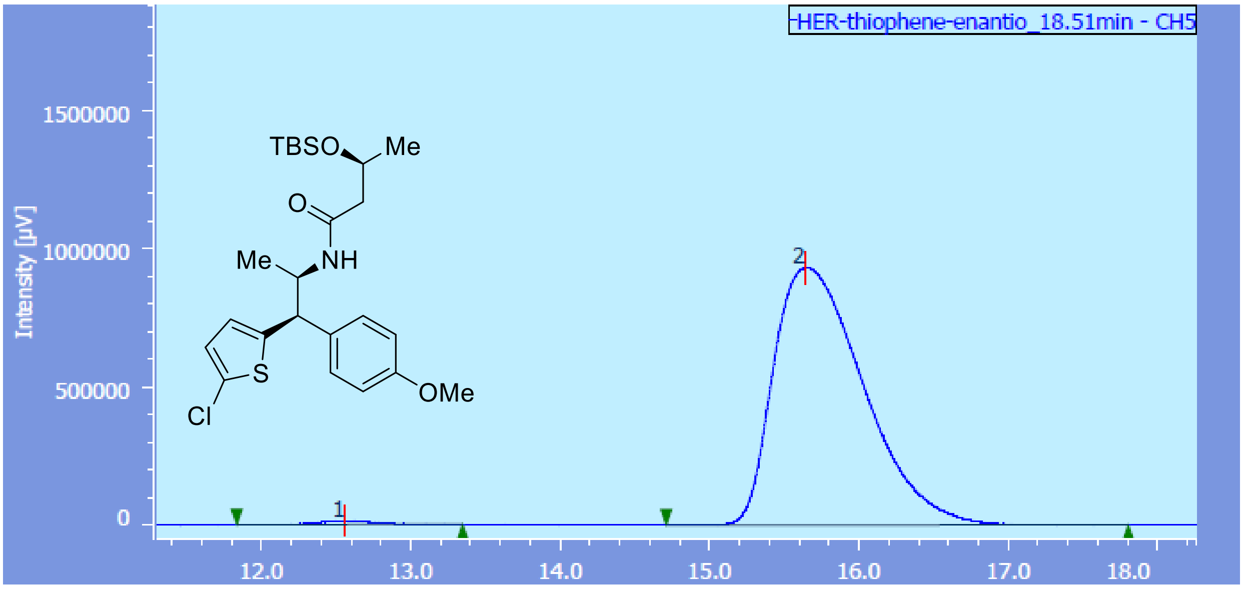

#	Peak Name	CH	tR [min]	Area [µV·sec]	Height [µV]	Area%	Height%	Quantity	NTP	Resolution	Symmetry Factor	Warning
1	Unknown	5	12.563	385224	14922	0.980	1.579	N/A	5486	3.435	1.030	
2	Unknown	5	15.643	38925452	929969	99.020	98.421	N/A	3125	N/A	1.633	

(*S*)-3-[(tert-Butyldimethylsilyl)oxy]-*N*-[(1*R*,2*R*)-1-(5-chlorothiophen-2-yl)-1-(4-methoxyphenyl)propan-2-yl]butanamide (2.20)

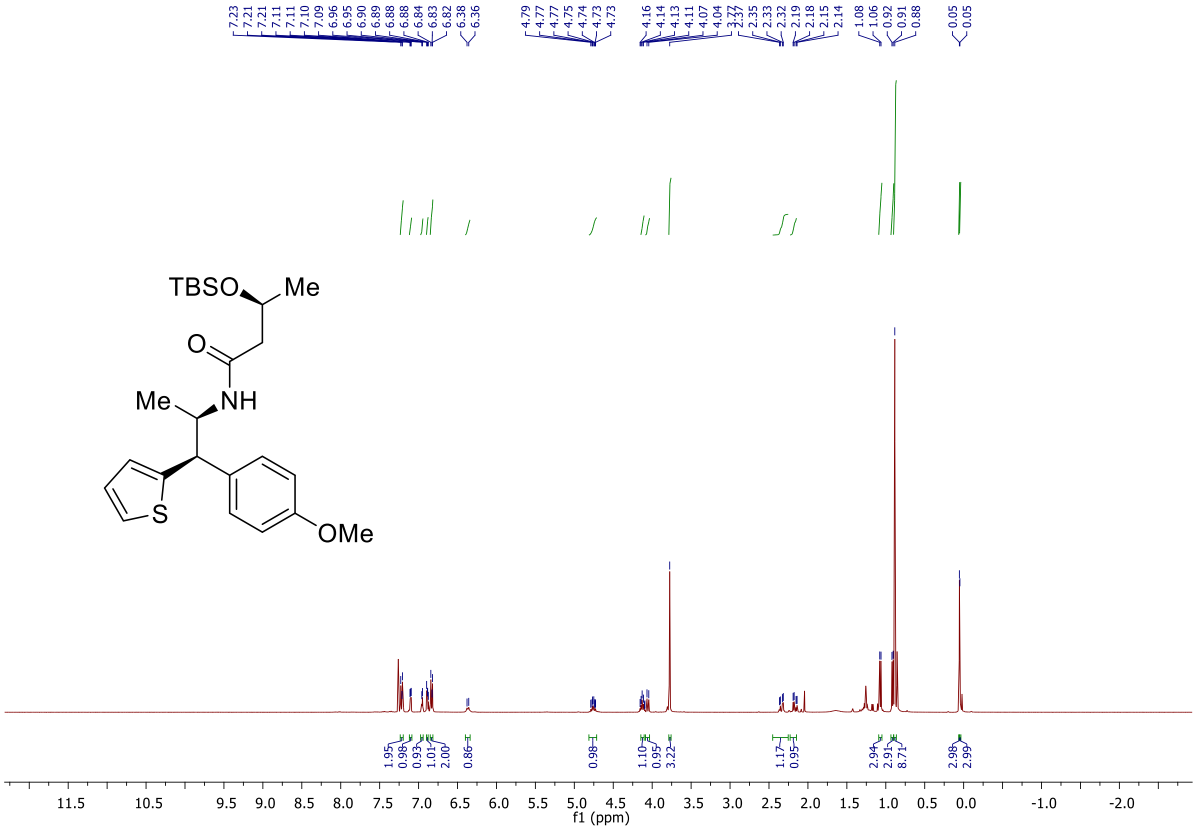

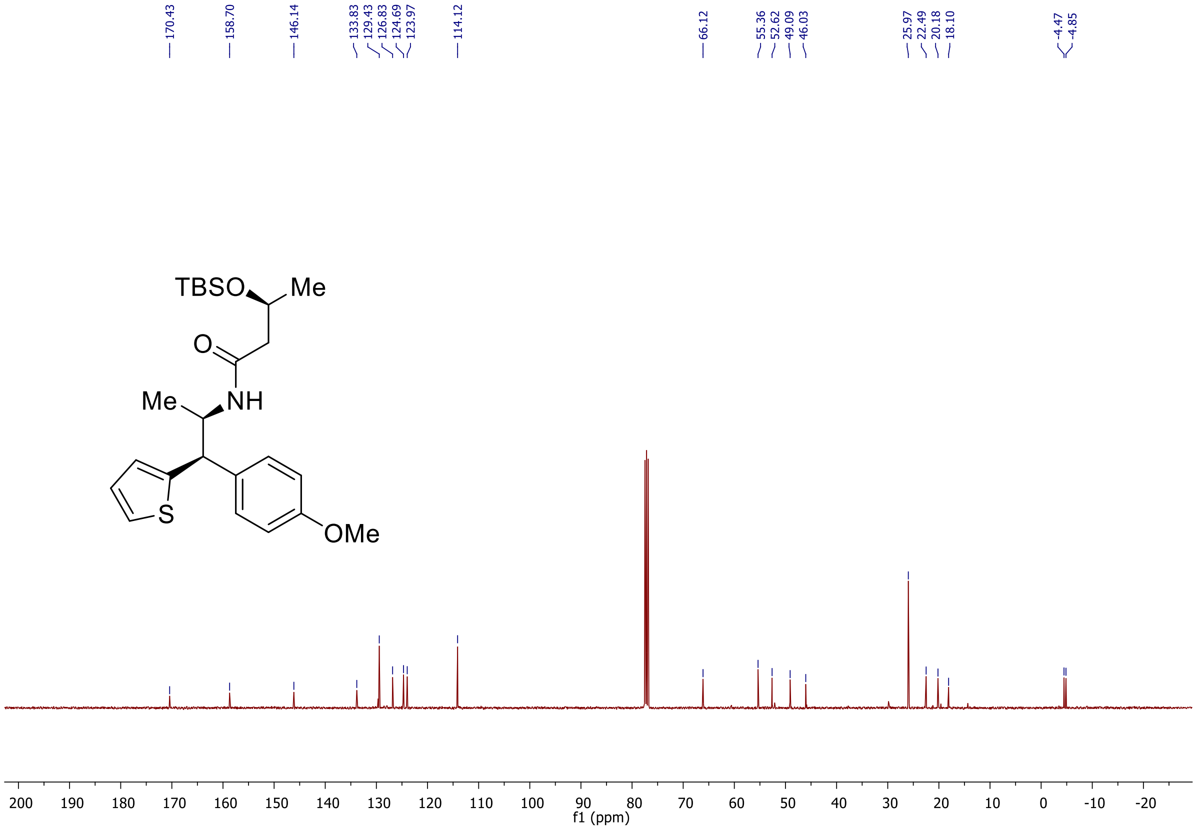

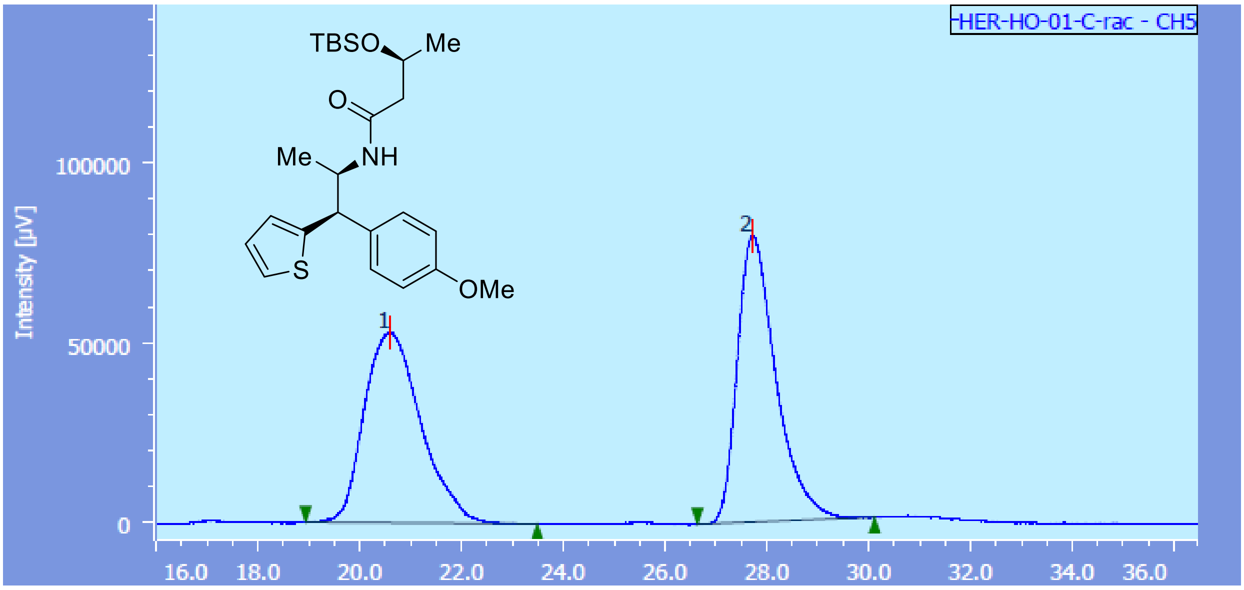

#	Peak Name	CH	tR [min]	Area [µV·sec]	Height [µV]	Area%	Height%	Quantity	NTP	Resolution	Symmetry Factor	Warning
1	Unknown	5	20.603	4105087	52993	49.402	39.933	N/A	1630	4.223	1.241	
2	Unknown	5	27.720	4204493	79713	50.598	60.067	N/A	6868	N/A	1.501	

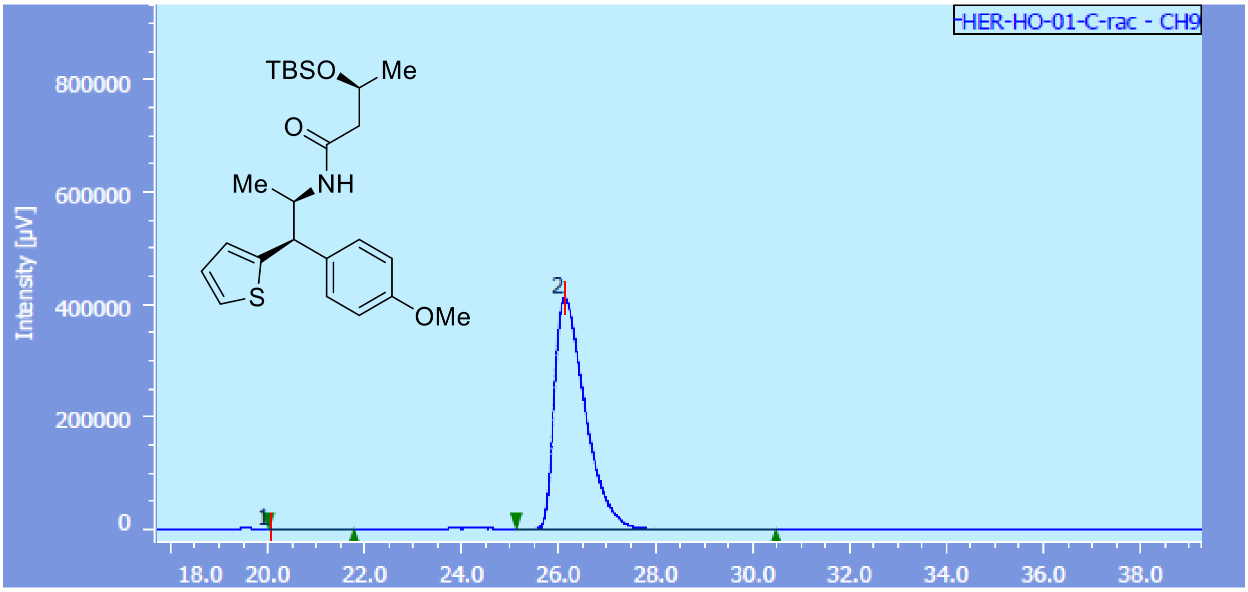

#	Peak Name	CH	tR [min]	Area [µV·sec]	Height [µV]	Area%	Height%	Quantity	NTP	Resolution	Symmetry Factor	Warning
1	Unknown	9	20.057	138	85	0.001	0.021	N/A	4704700	10.314	20.074	
2	Unknown	9	26.123	18080769	410733	99.999	99.979	N/A	8365	N/A	1.822	

N-[(1*R*,2*R*)-1-(4-Methoxyphenyl)-1,4-di-*p*-tolylbutan-2-yl]butyramide (2.21)

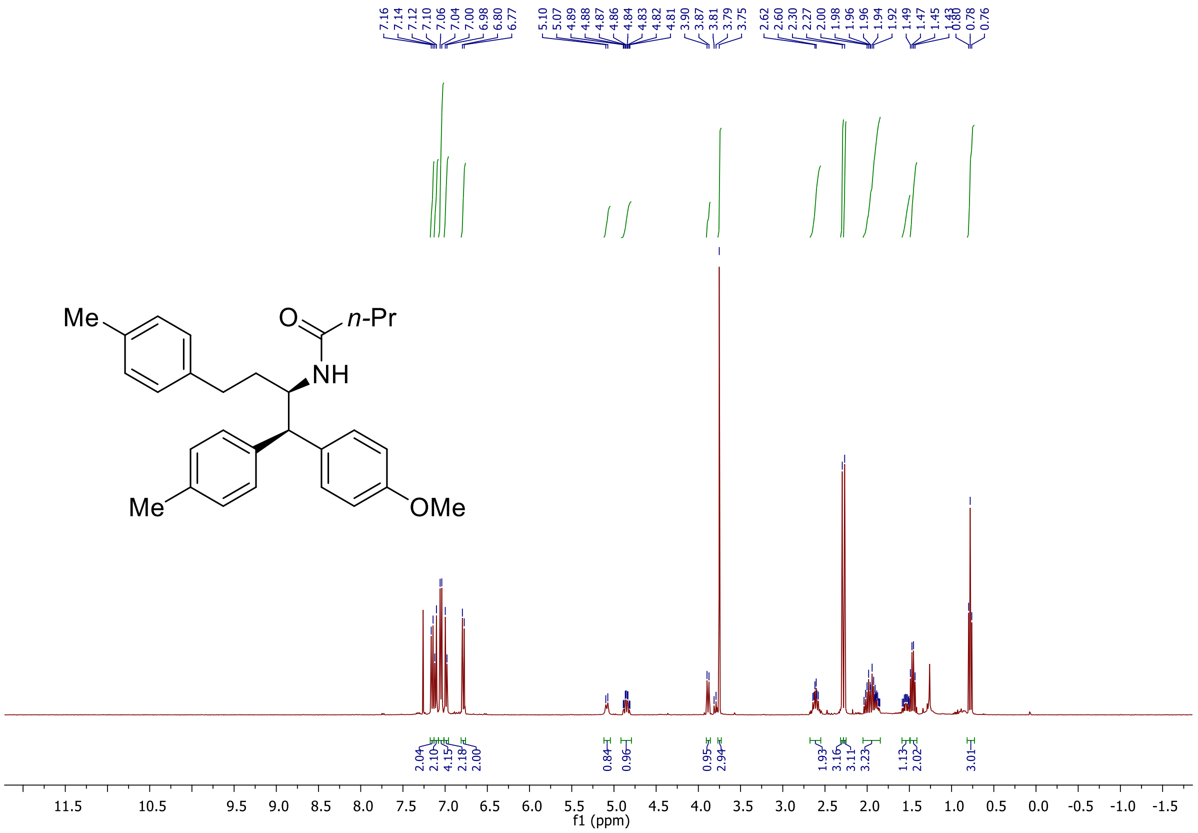

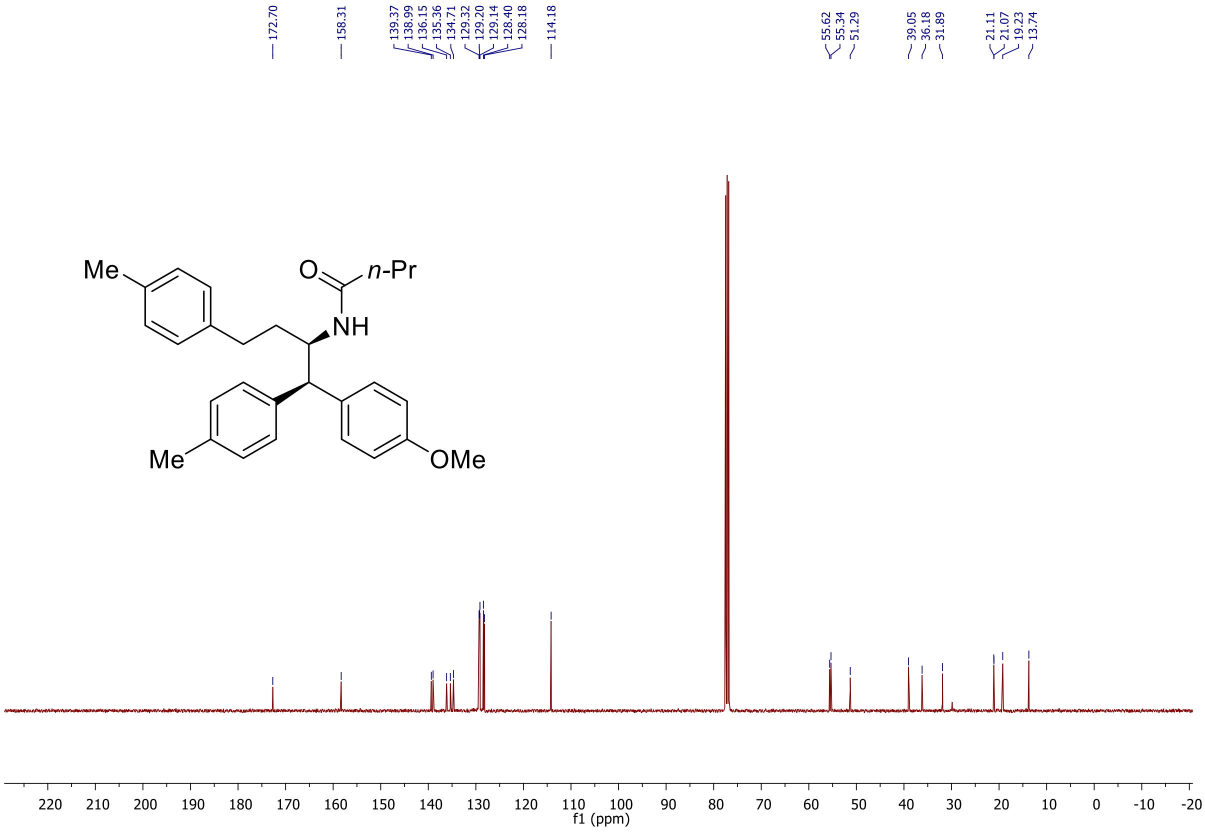

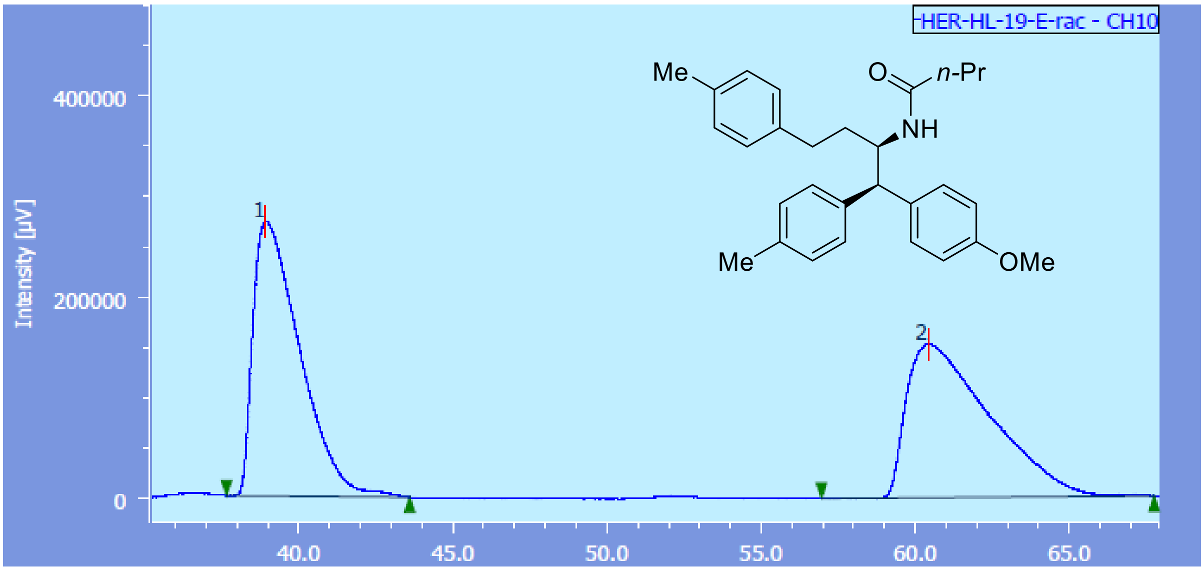

#	Peak Name	CH	tR [min]	Area [μV·sec]	Height [μV]	Area%	Height%	Quantity	NTP	Resolution	Symmetry Factor	Warning
1	Unknown	10	38.940	28813841	272087	50.169	64.091	N/A	3092	5.514	2.247	
2	Unknown	10	60.420	28619777	152444	49.831	35.909	N/A	2326	N/A	2.400	

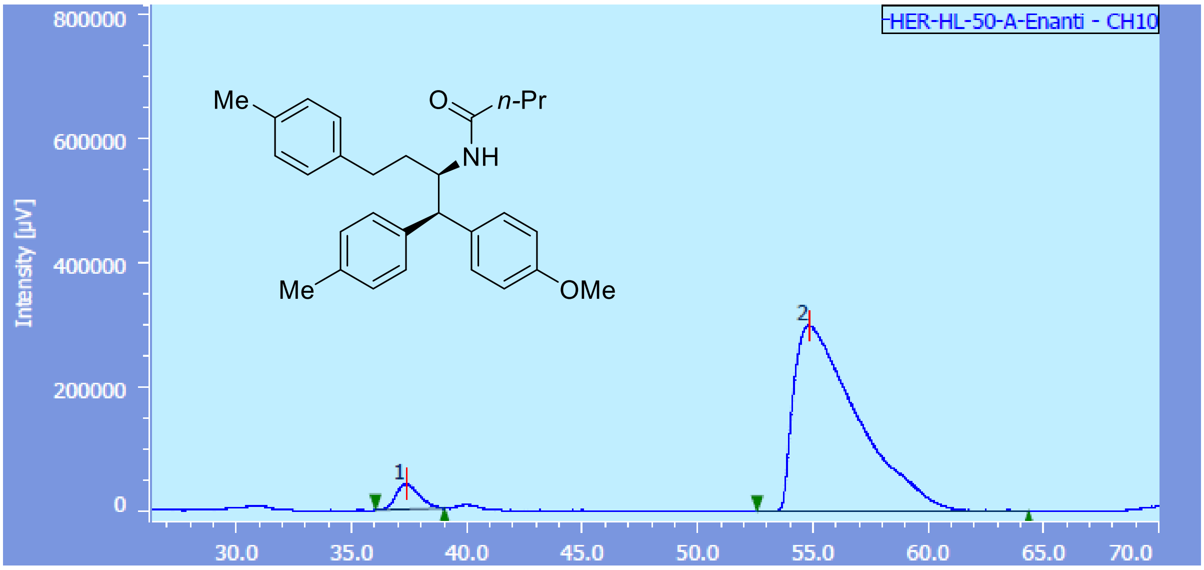

#	Peak Name	CH	tR [min]	Area [μV·sec]	Height [μV]	Area%	Height%	Quantity	NTP	Resolution	Symmetry Factor	Warning
1	Unknown	10	37.353	2927249	41162	4.830	12.090	N/A	6195	5.096	1.264	
2	Unknown	10	54.820	57672107	299289	95.170	87.910	N/A	1943	N/A	2.894	

N-[(1R,2R)-1-Cyclohexyl-2-(4-methoxyphenyl)-2-(p-tolyl)ethyl]butyramide (2.22)

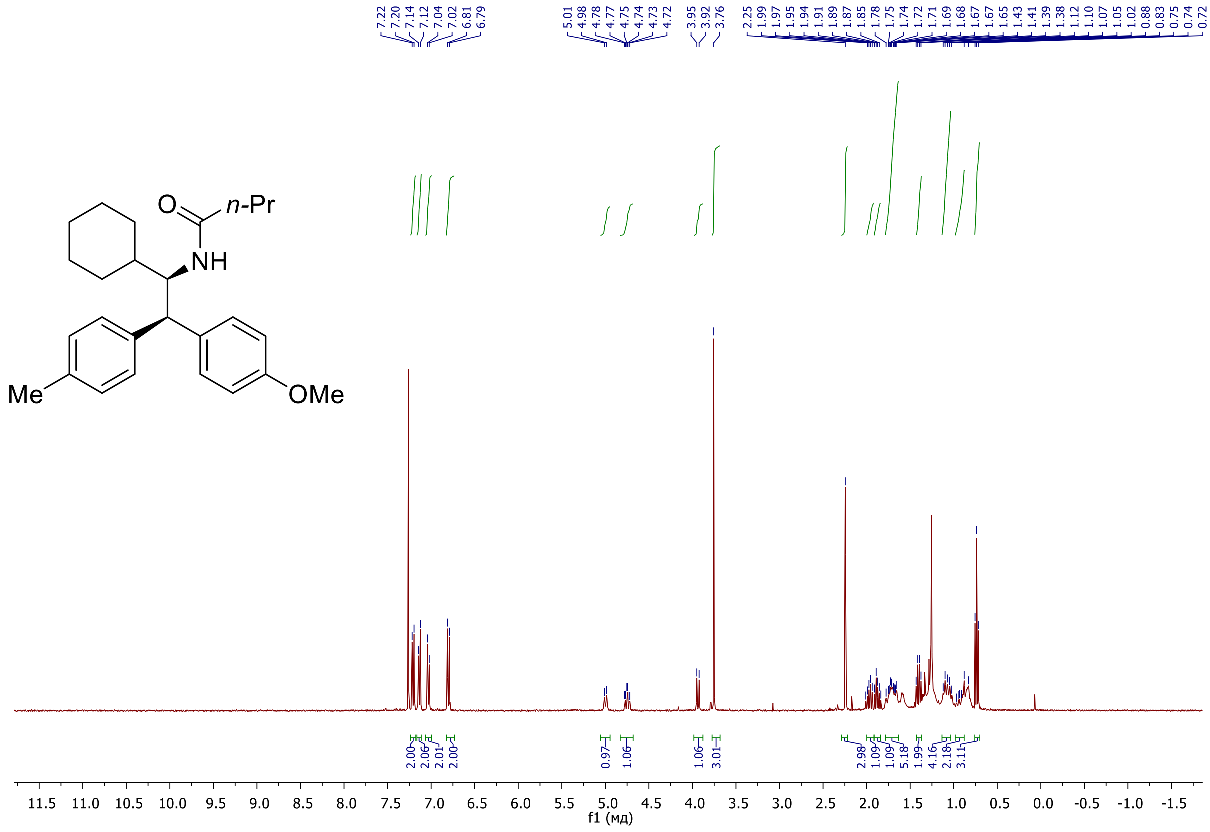

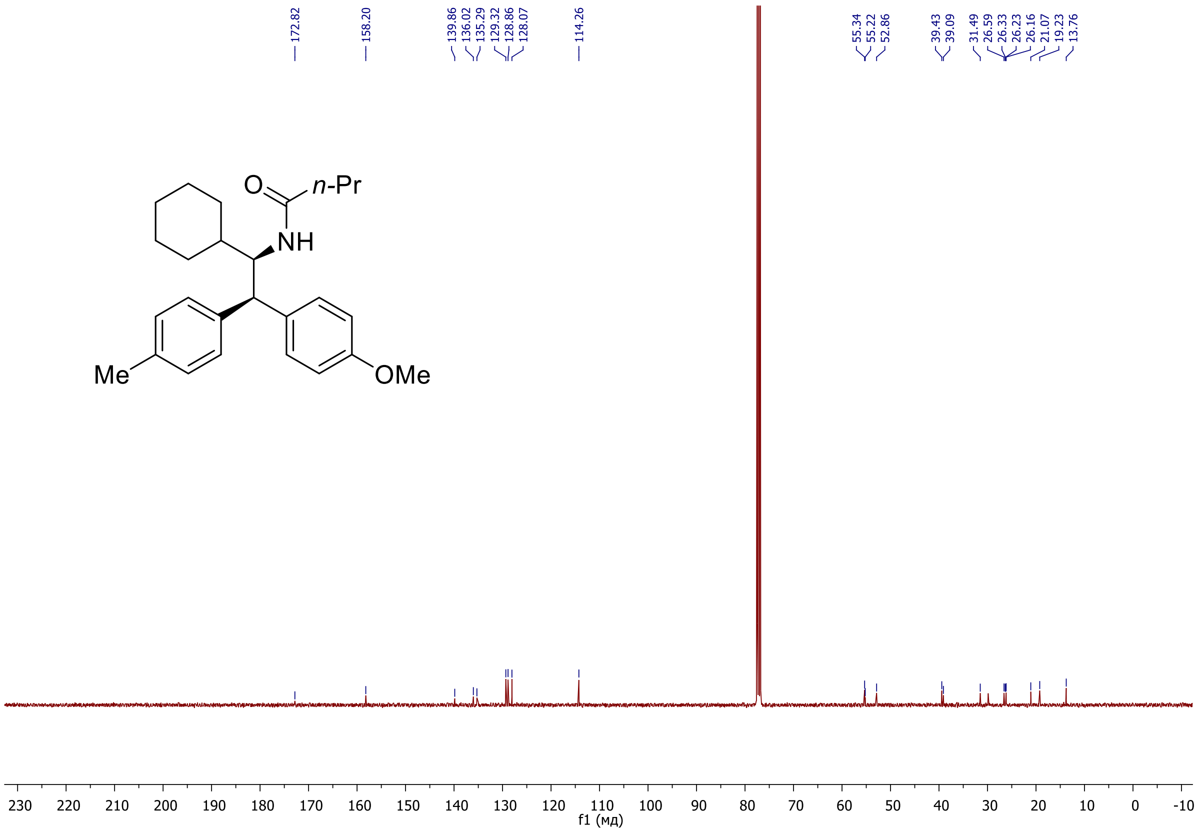

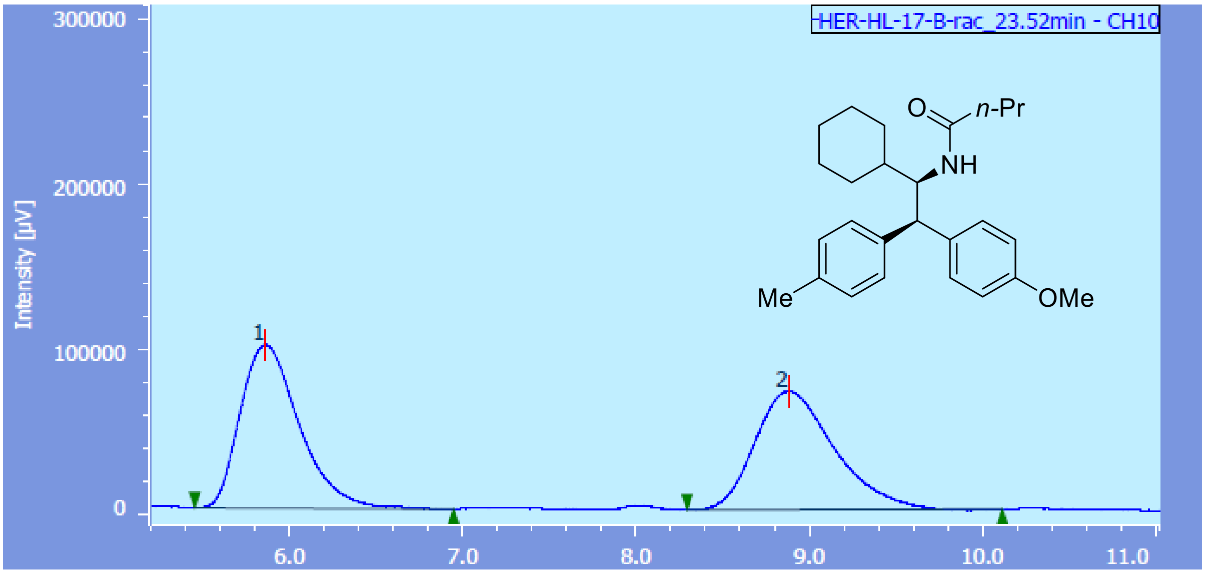

#	Peak Name	CH	tR [min]	Area [µV·sec]	Height [µV]	Area%	Height%	Quantity	NTP	Resolution	Symmetry Factor	Warning
1	Unknown	10	5.860	2346428	98738	50.710	57.964	N/A	1447	4.199	1.396	
2	Unknown	10	8.880	2280724	71604	49.290	42.036	N/A	1848	N/A	1.336	

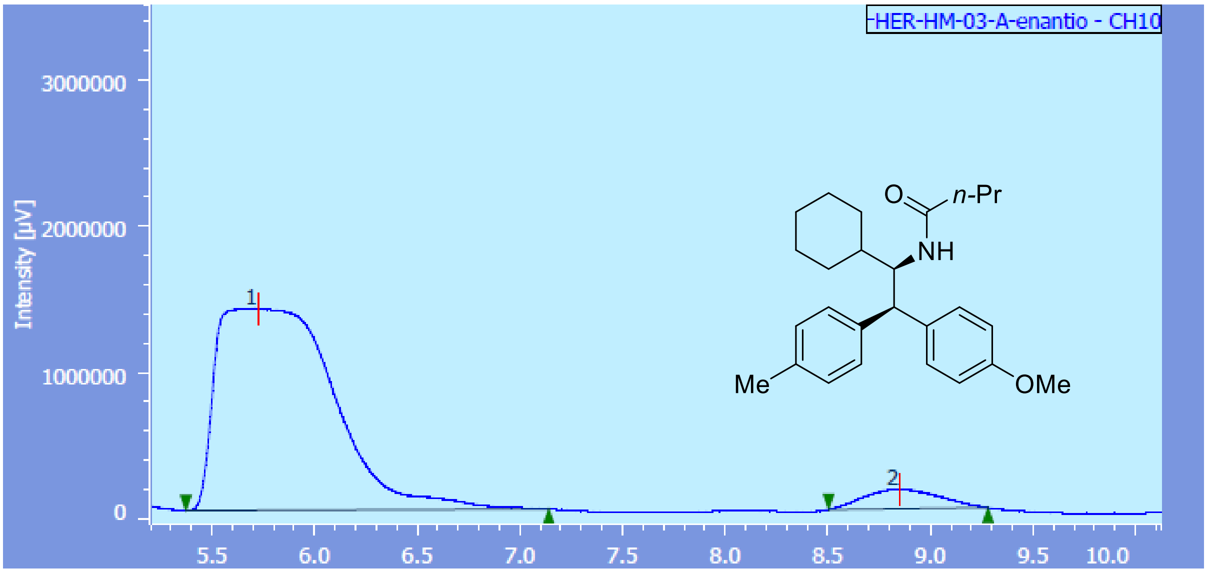

#	Peak Name	CH	tR [min]	Area [µV·sec]	Height [µV]	Area%	Height%	Quantity	NTP	Resolution	Symmetry Factor	Warning
1	Unknown	10	5.723	55070453	1376323	94.212	91.241	N/A	463	3.500	2.098	
2	Unknown	10	8.847	3383403	132118	5.788	8.759	N/A	2374	N/A	1.129	

N-((1*R*,2*R*)-1-Cyclohexyl-2-(*p*-tolyl)-2-(3,4,5-trimethoxyphenyl)ethyl)butyramide (2.23)

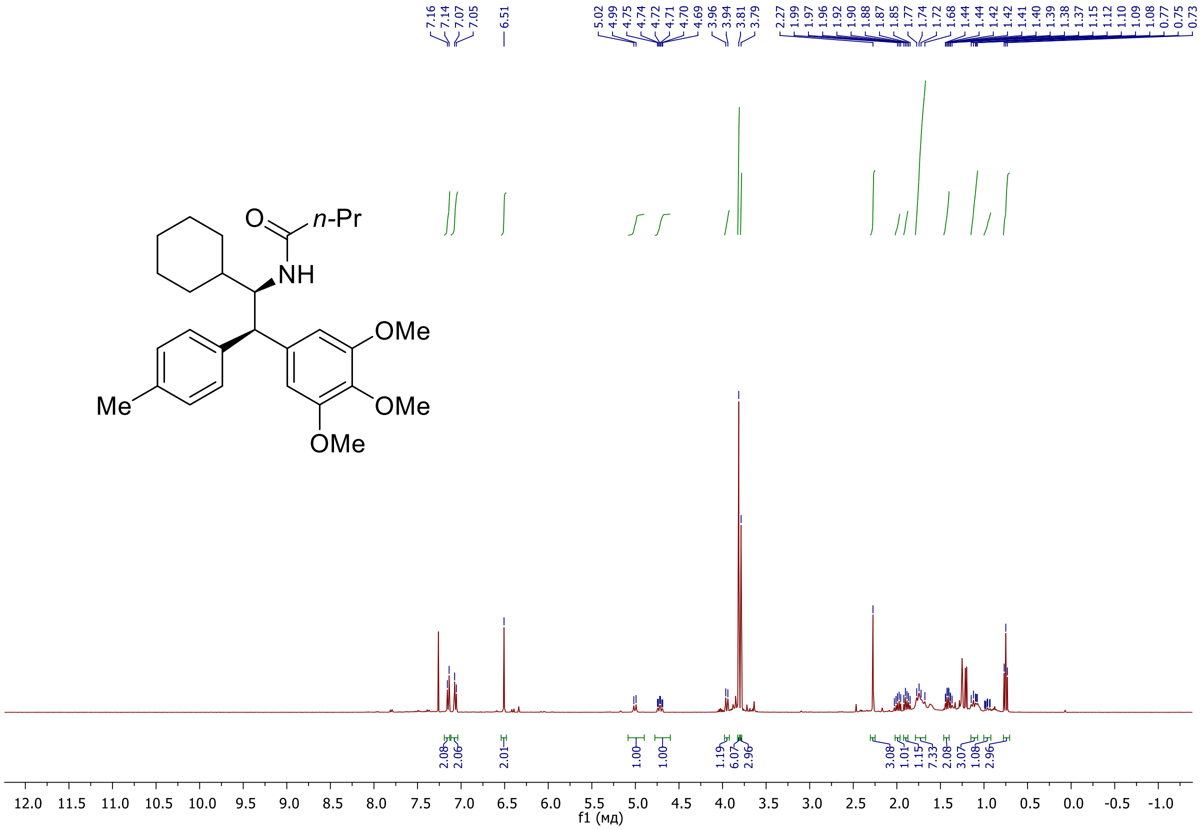

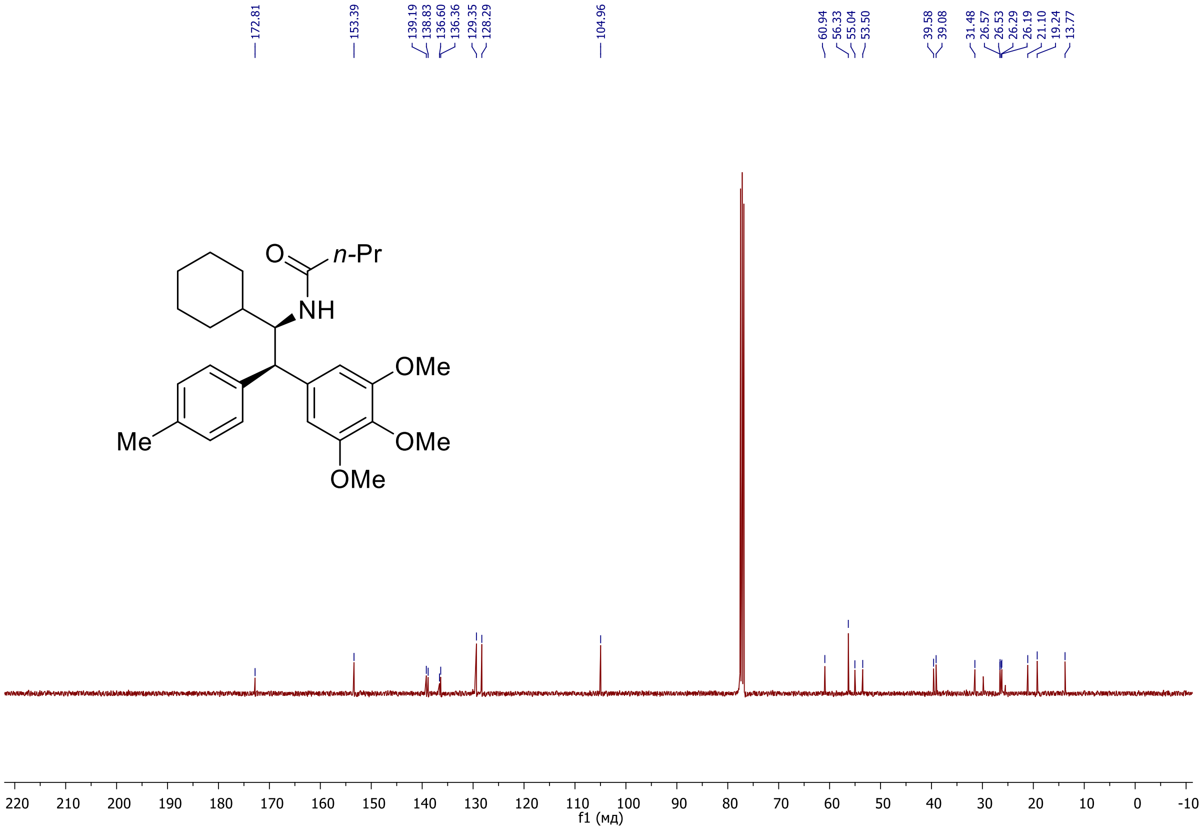

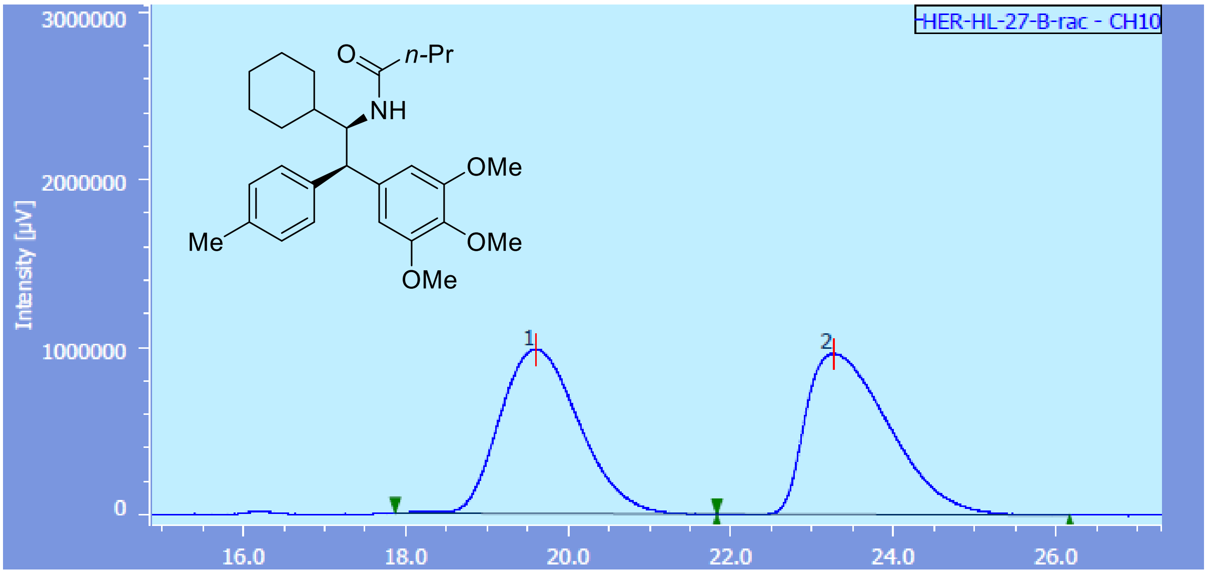

#	Peak Name	CH	tR [min]	Area [µV·sec]	Height [µV]	Area%	Height%	Quantity	NTP	Resolution	Symmetry Factor	Warning
1	Unknown	10	19.597	66717497	980557	49.384	50.569	N/A	1851	1.958	1.229	
2	Unknown	10	23.260	68381393	958473	50.616	49.431	N/A	2326	N/A	1.794	

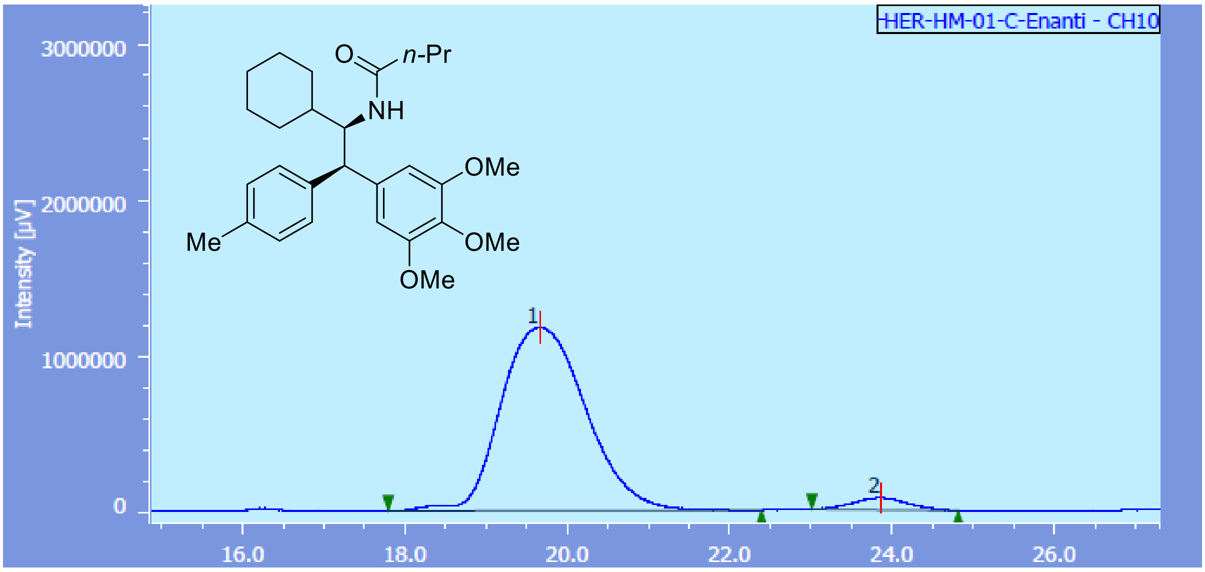

#	Peak Name	CH	tR [min]	Area [µV·sec]	Height [µV]	Area%	Height%	Quantity	NTP	Resolution	Symmetry Factor	Warning
1	Unknown	10	19.657	85532133	1171546	95.896	93.896	N/A	1639	2.613	1.225	
2	Unknown	10	23.860	3660705	76166	4.104	6.104	N/A	5525	N/A	1.046	

N-((1R,2R)-2-(4-Methoxyphenyl)-1-(tetrahydro-2H-pyran-4-yl)-2-(p-tolyl)ethyl)

butyramide (2.24)

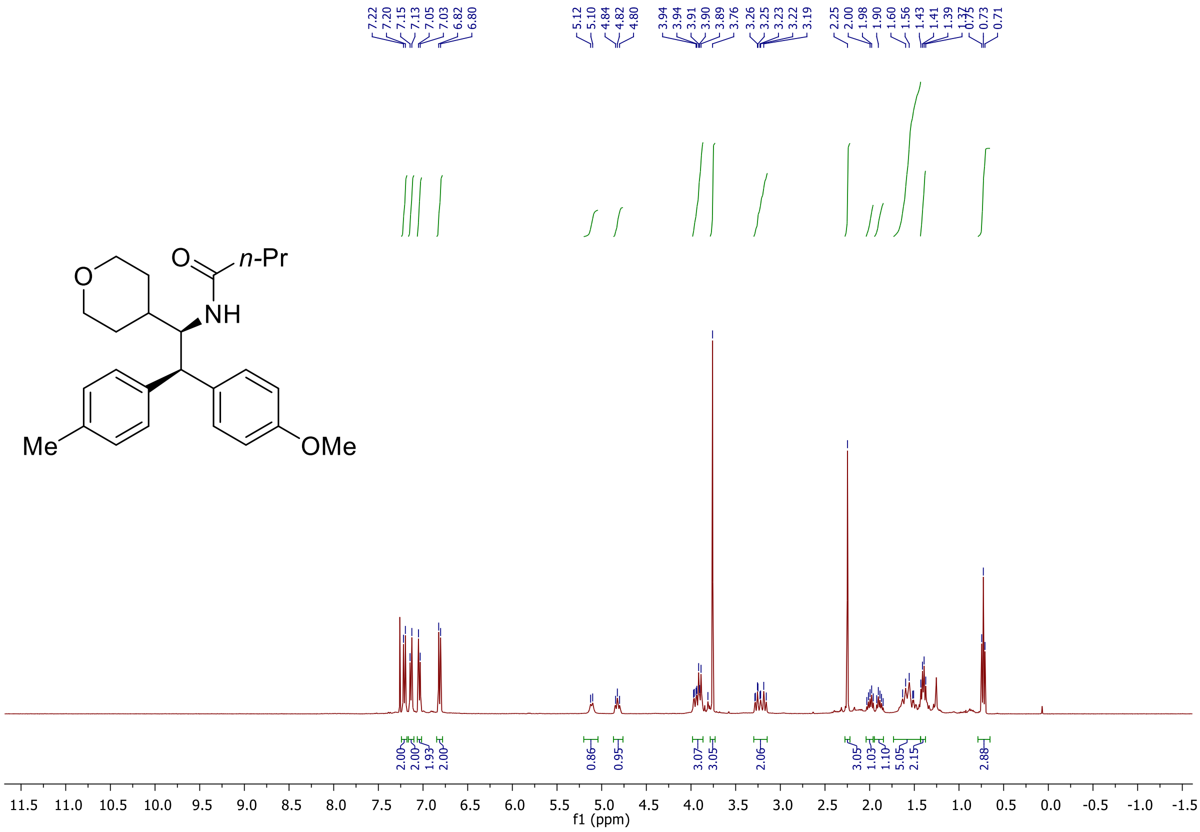

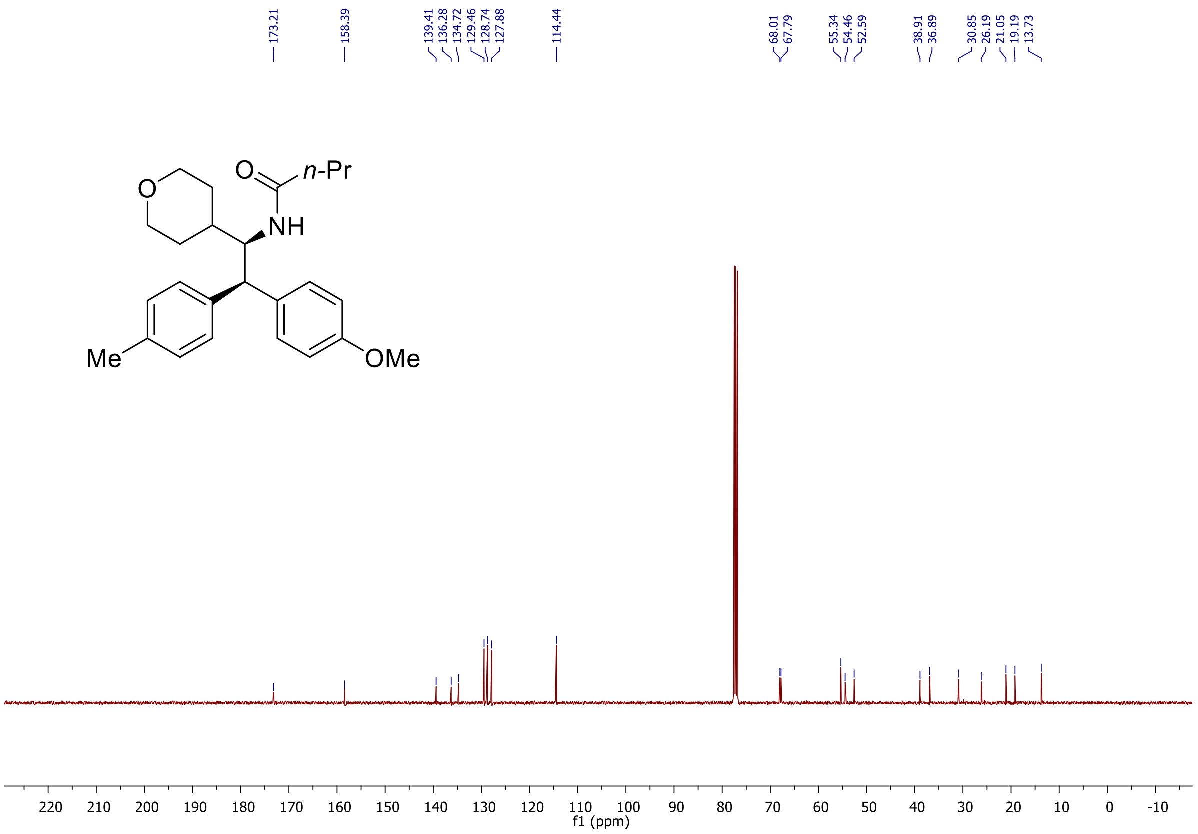

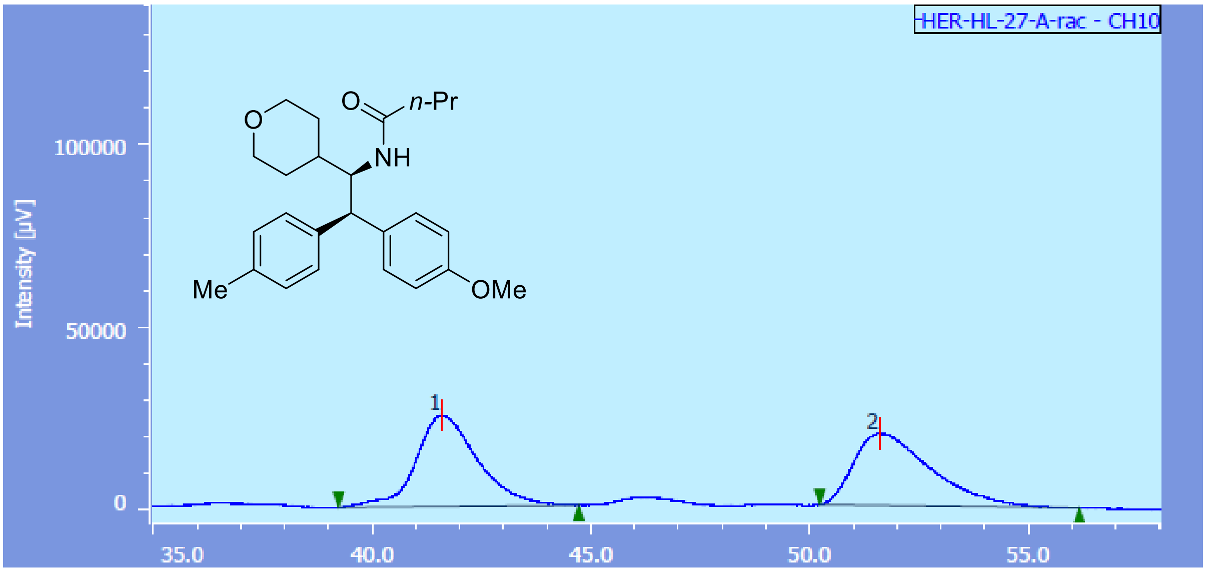

#	Peak Name	CH	tR [min]	Area [µV·sec]	Height [µV]	Area%	Height%	Quantity	NTP	Resolution	Symmetry Factor	Warning
1	Unknown	10	41.587	2440950	24833	50.294	55.864	N/A	4711	3.567	1.105	
2	Unknown	10	51.590	2412410	19619	49.706	44.136	N/A	4159	N/A	1.807	

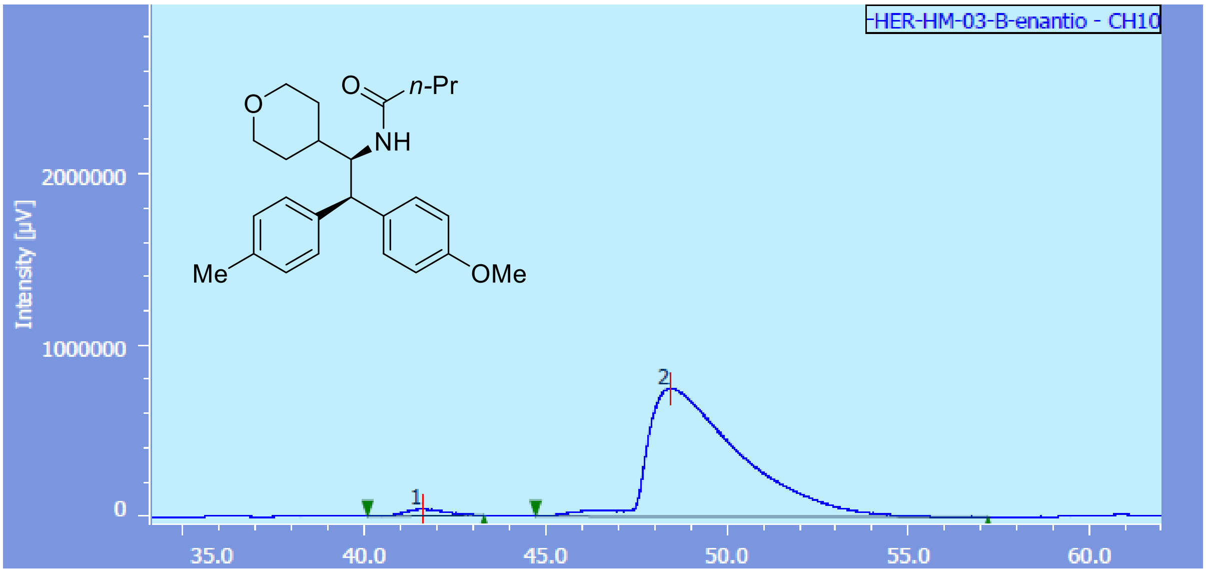

#	Peak Name	CH	tR [min]	Area [µV·sec]	Height [µV]	Area%	Height%	Quantity	NTP	Resolution	Symmetry Factor	Warning
1	Unknown	10	41.607	2993459	37835	2.234	4.825	N/A	6009	2.110	1.216	
2	Unknown	10	48.437	131011826	746367	97.766	95.175	N/A	1988	N/A	2.906	

(2*S*,3*R*)-2-Butyramido-3-(4-methoxyphenyl)-3-(*p*-tolyl)propyl acetate (2.25)

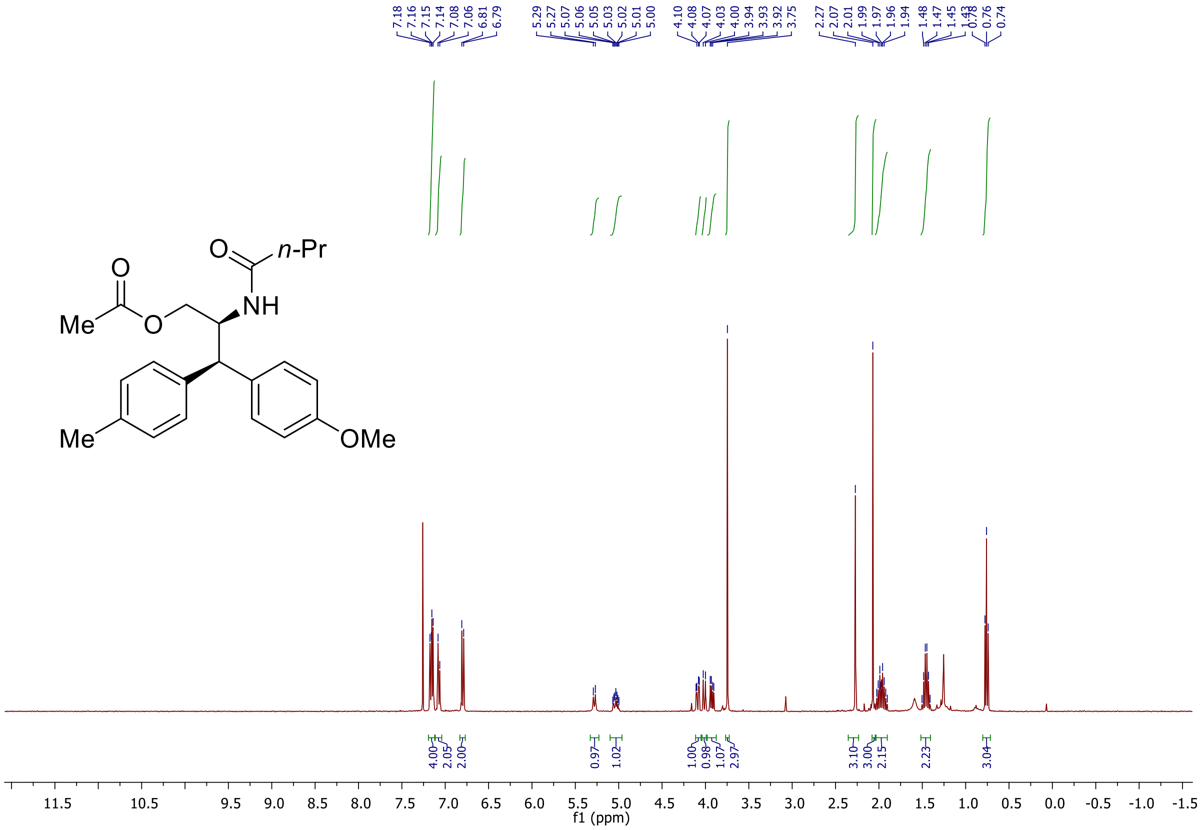

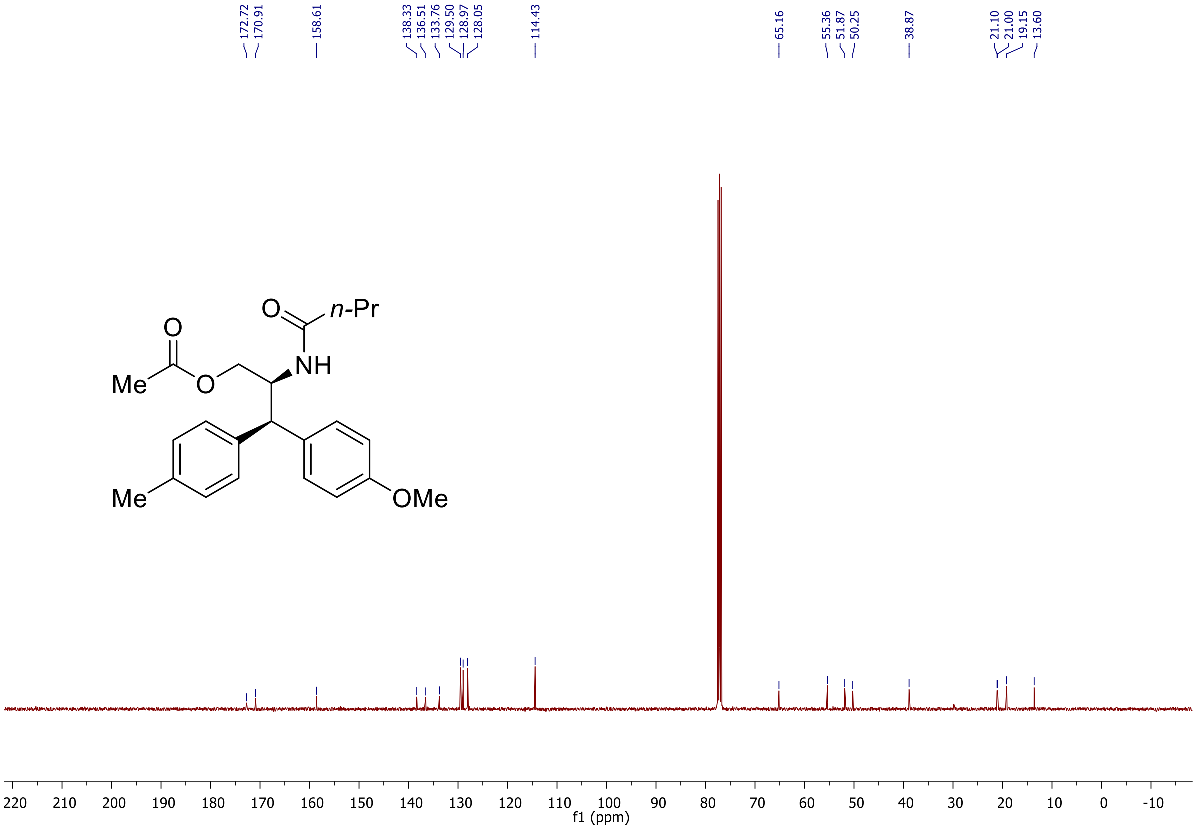

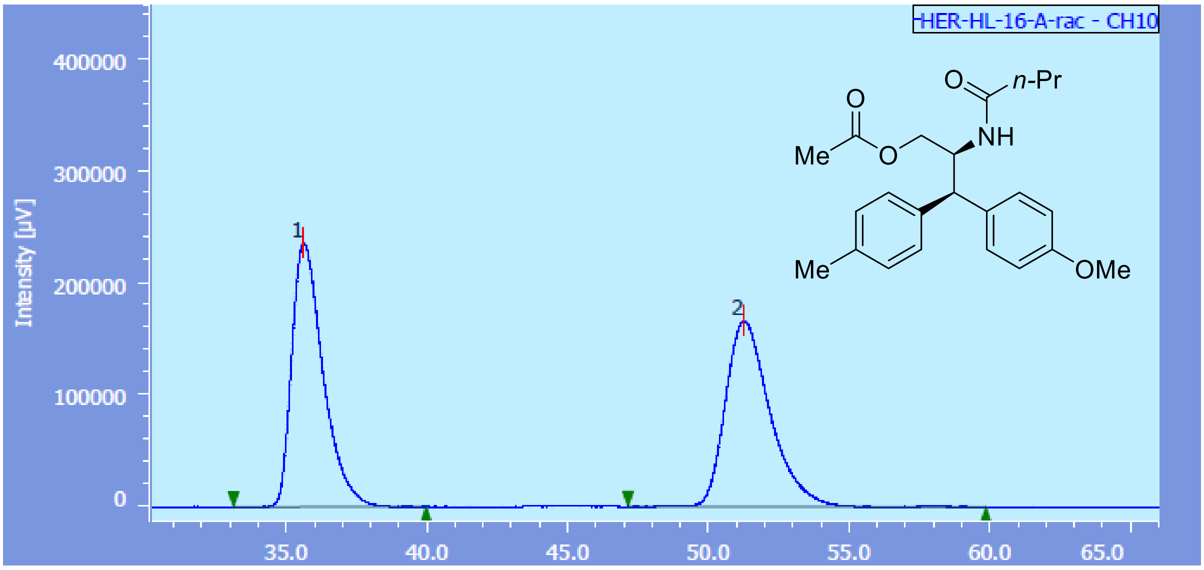

#	Peak Name	CH	tR [min]	Area [µV·sec]	Height [µV]	Area%	Height%	Quantity	NTP	Resolution	Symmetry Factor	Warning
1	Unknown	10	35.630	18214494	235738	49.637	58.634	N/A	5168	6.537	1.554	
2	Unknown	10	51.277	18480734	166313	50.363	41.366	N/A	5298	N/A	1.389	

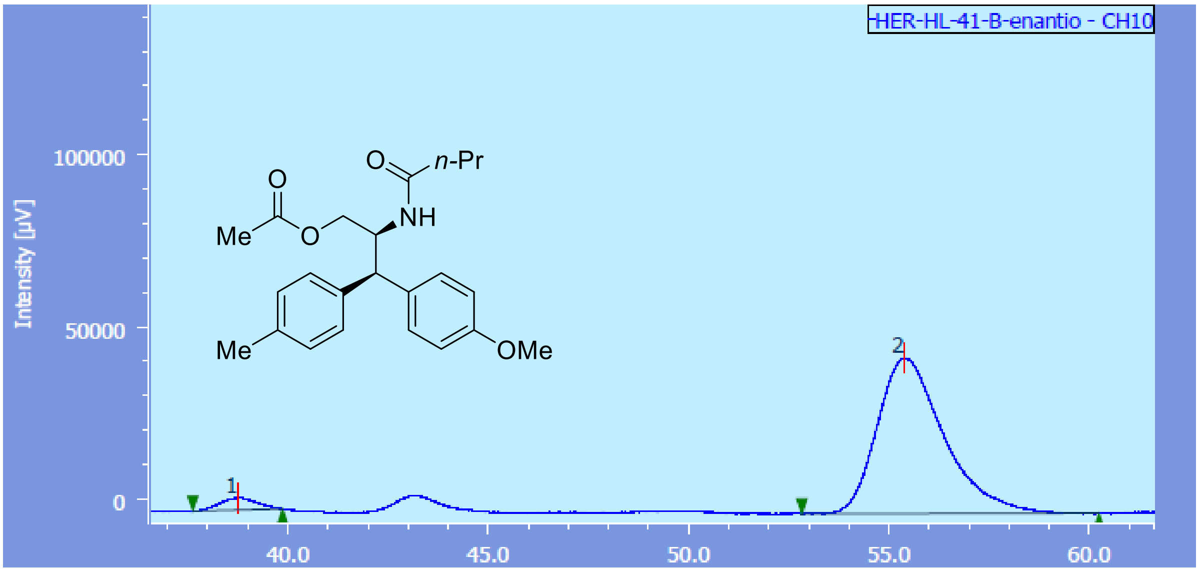

#	Peak Name	CH	tR [min]	Area [µV·sec]	Height [µV]	Area%	Height%	Quantity	NTP	Resolution	Symmetry Factor	Warning
1	Unknown	10	38.757	227093	3504	4.165	7.198	N/A	7349	7.037	1.034	
2	Unknown	10	55.393	5225058	45178	95.835	92.802	N/A	5710	N/A	1.424	

N-[(1R,2R,4R)-1-(4-Methoxyphenyl)-4,8-dimethyl-1-(p-tolyl)non-7-en-2-yl]butyramide (2.26)

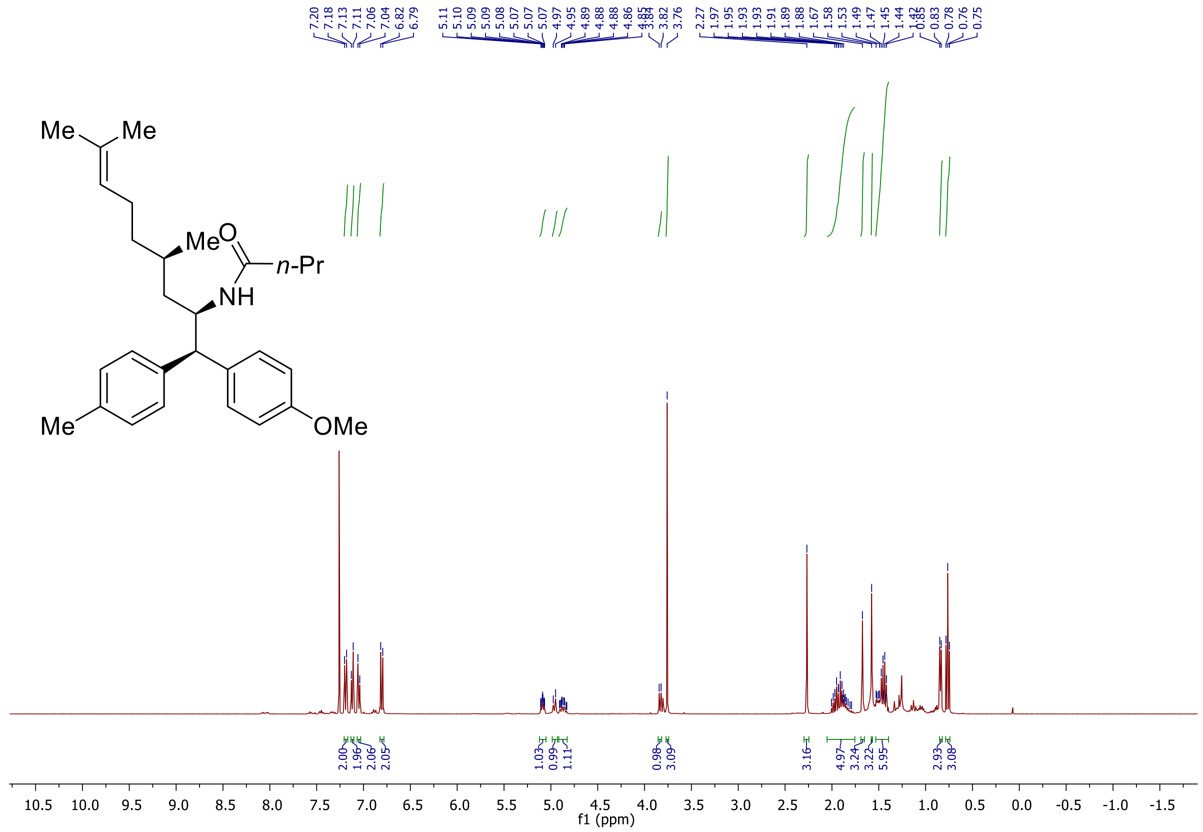

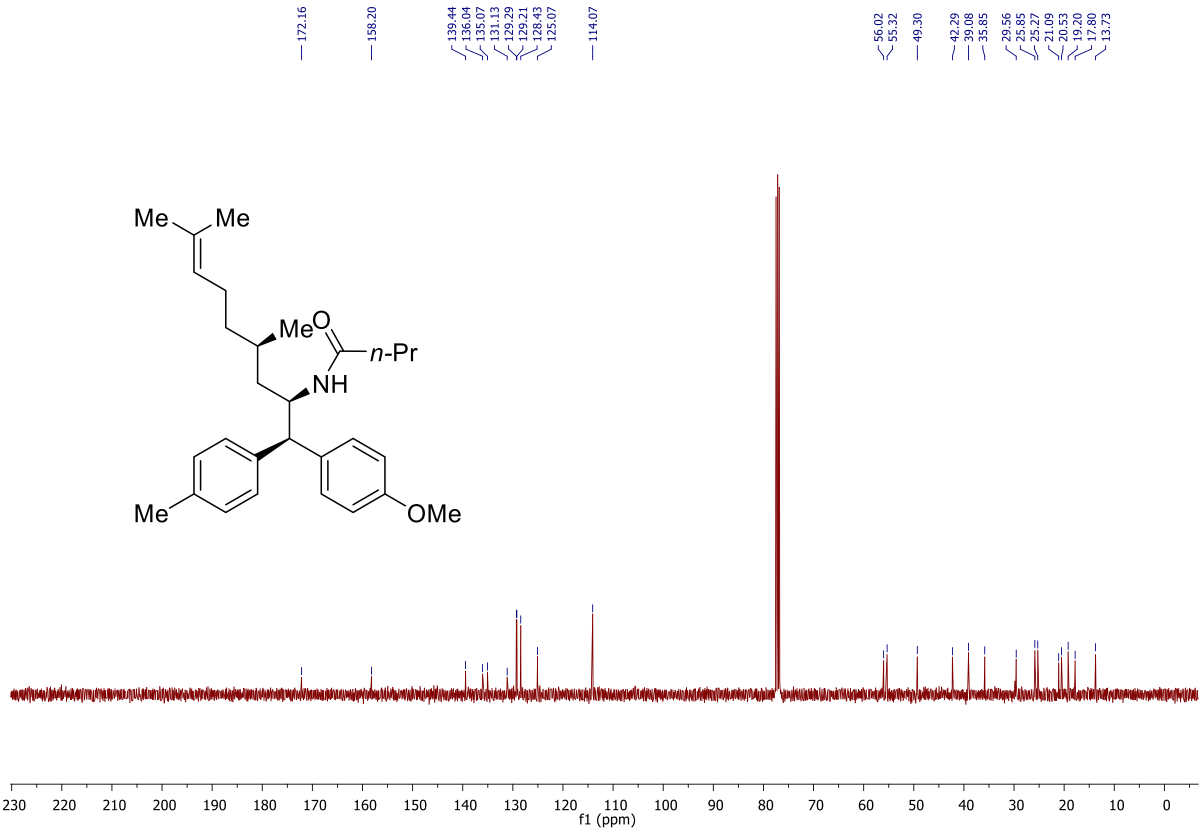

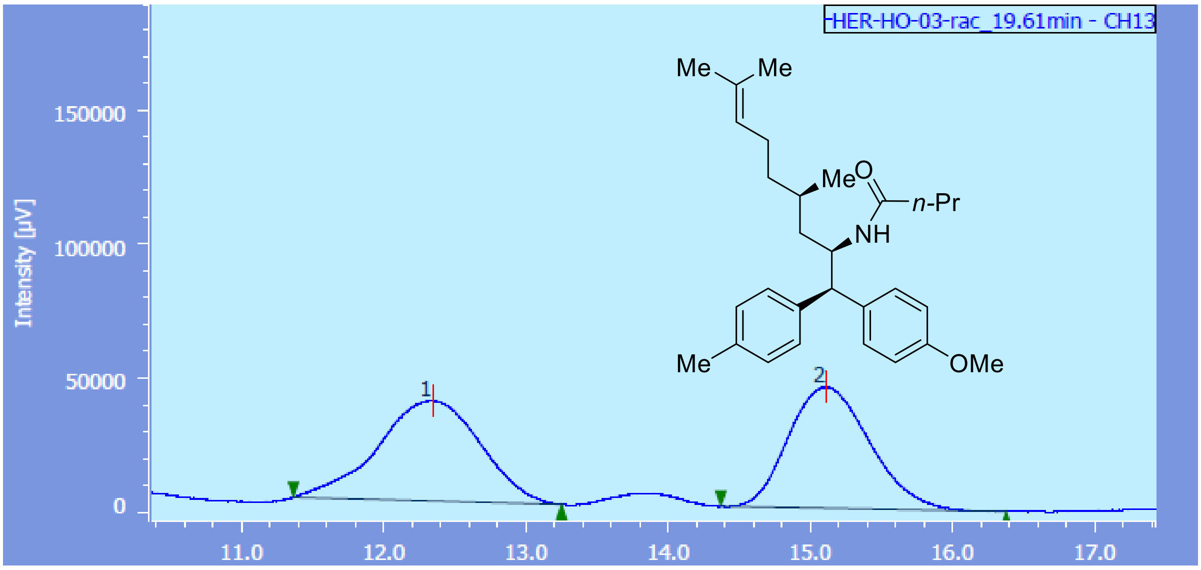

#	Peak Name	CH	tR [min]	Area [µV·sec]	Height [µV]	Area%	Height%	Quantity	NTP	Resolution	Symmetry Factor	Warning
1	Unknown	13	12.343	1876636	37435	51.127	45.285	N/A	1411	2.329	0.916	
2	Unknown	13	15.110	1793913	45231	48.873	54.715	N/A	3208	N/A	1.189	

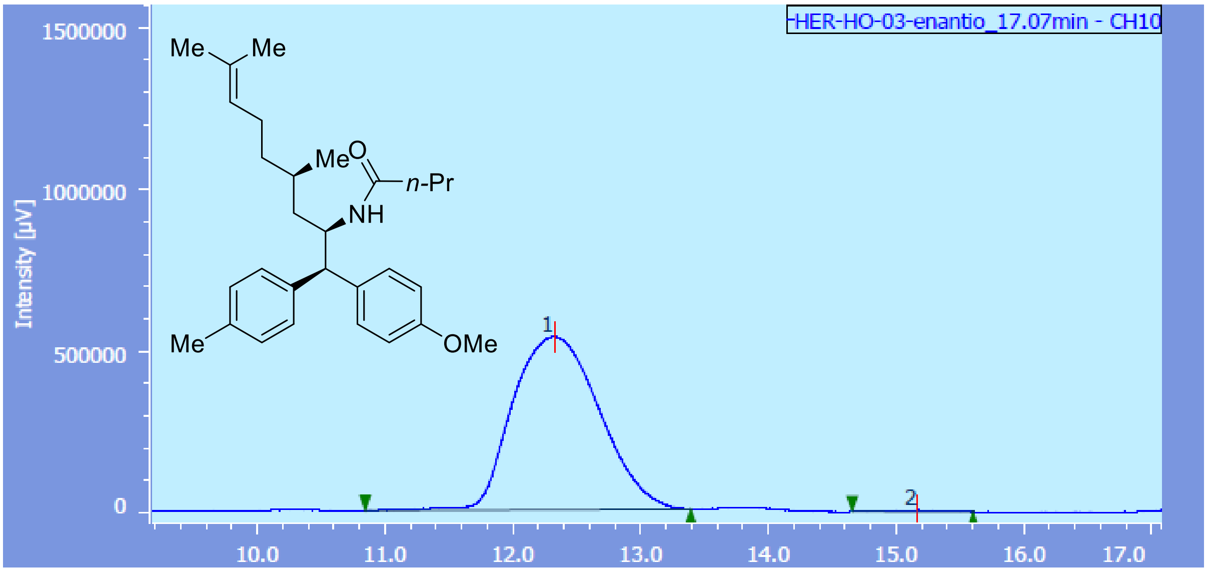

#	Peak Name	CH	tR [min]	Area [µV·sec]	Height [µV]	Area%	Height%	Quantity	NTP	Resolution	Symmetry Factor	Warning
1	Unknown	10	12.323	25294827	534153	99.391	99.069	N/A	1424	2.626	1.179	
2	Unknown	10	15.167	154887	5020	0.609	0.931	N/A	4915	N/A	0.945	

(*S*)-*N*-(2-Butyramido-1-(*p*-tolyl)ethyl)-4-methoxybenzamide (2.27)

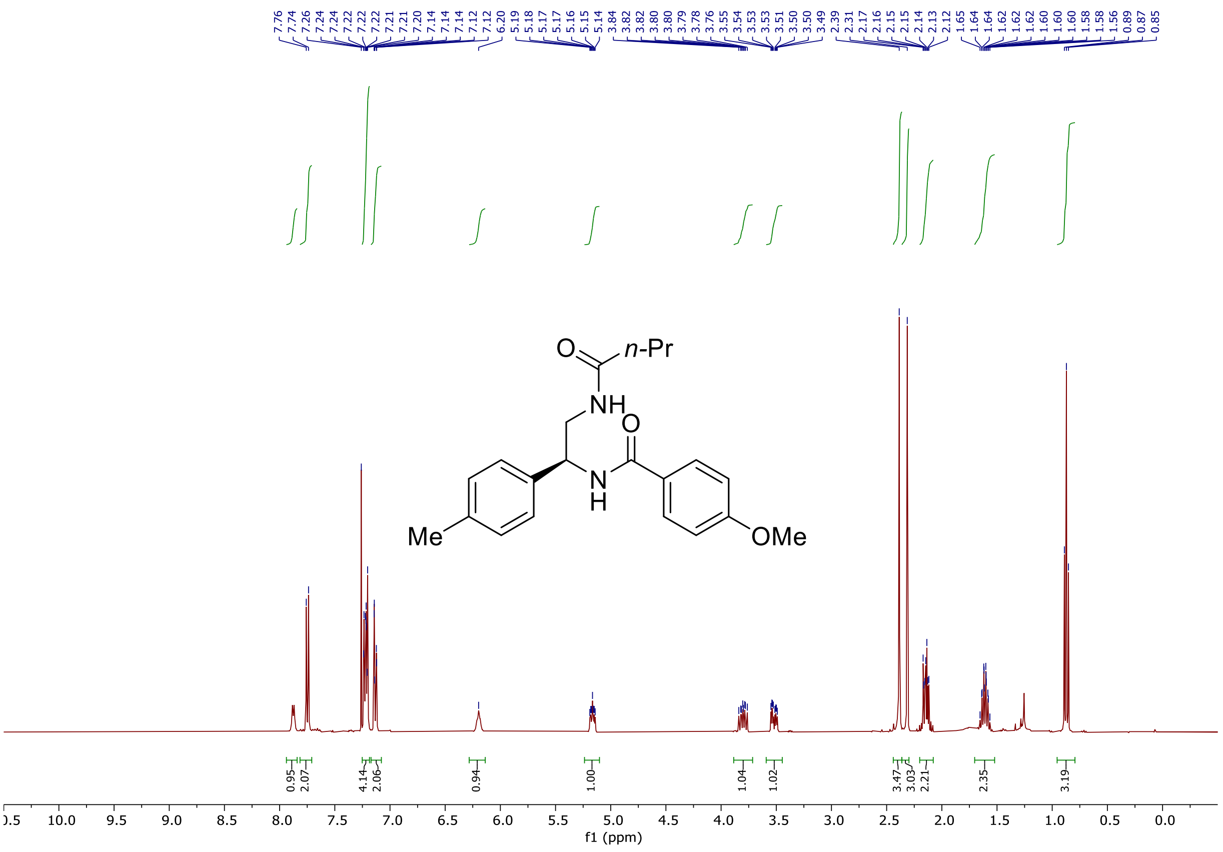

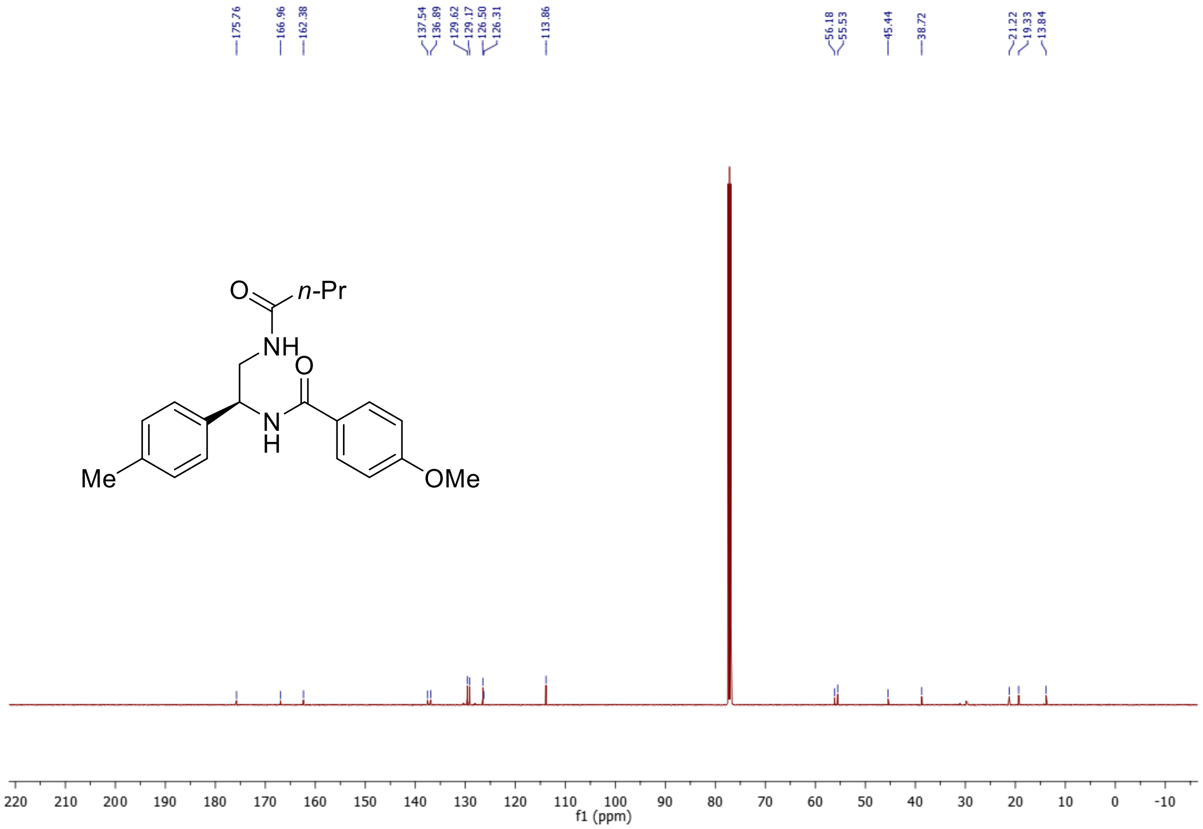

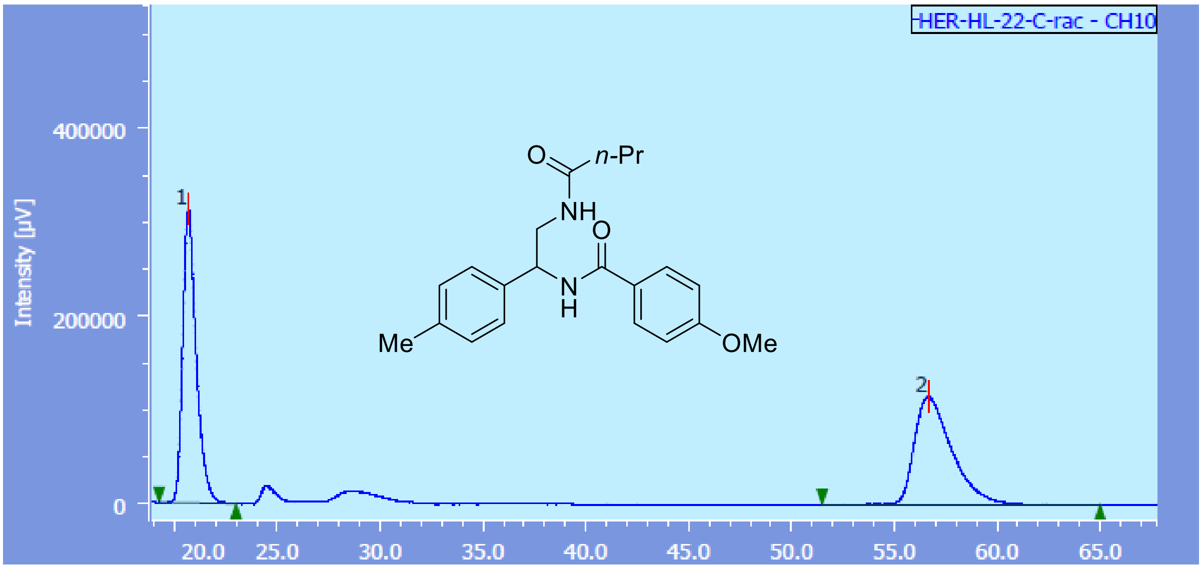

#	Peak Name	CH	tR [min]	Area [μV·sec]	Height [μV]	Area%	Height%	Quantity	NTP	Resolution	Symmetry Factor	Warning
1	Unknown	10	20.683	14304861	313658	49.458	73.127	N/A	5198	16.479	1.406	
2	Unknown	10	56.630	14618610	115265	50.542	26.873	N/A	4928	N/A	1.625	

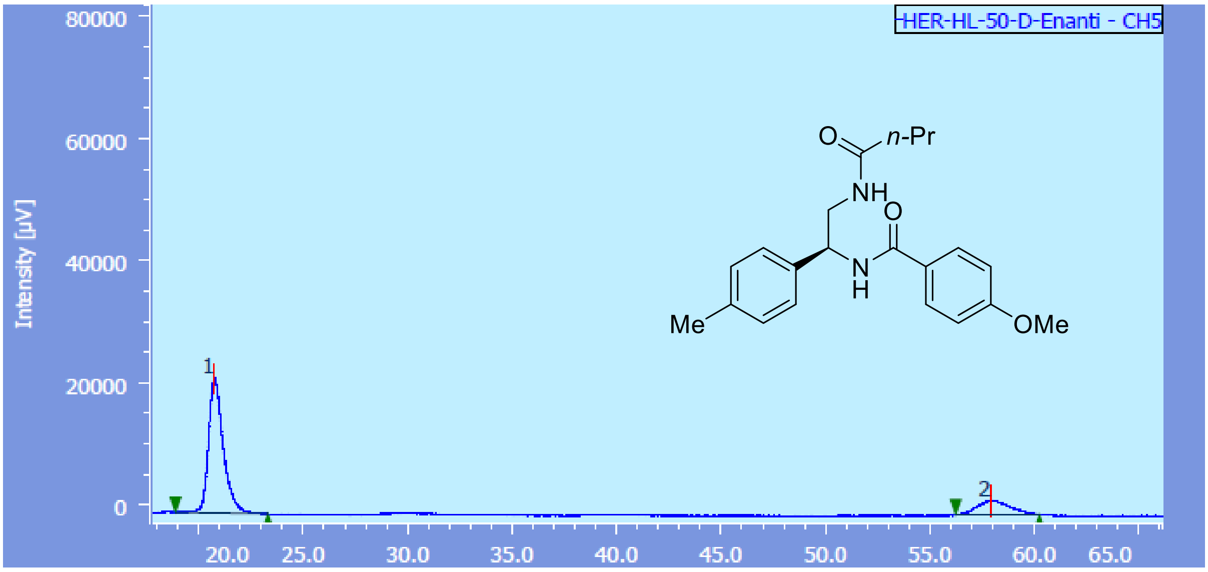

#	Peak Name	CH	tR [min]	Area [μV·sec]	Height [μV]	Area%	Height%	Quantity	NTP	Resolution	Symmetry Factor	Warning
1	Unknown	5	20.757	1085703	21999	81.057	90.476	N/A	4727	18.018	1.402	
2	Unknown	5	57.940	253732	2316	18.943	9.524	N/A	6254	N/A	1.225	

(*S*)-*N*-(2-Butyramido-1-(*p*-tolyl)ethyl)-4-methylbenzamide (2.28)

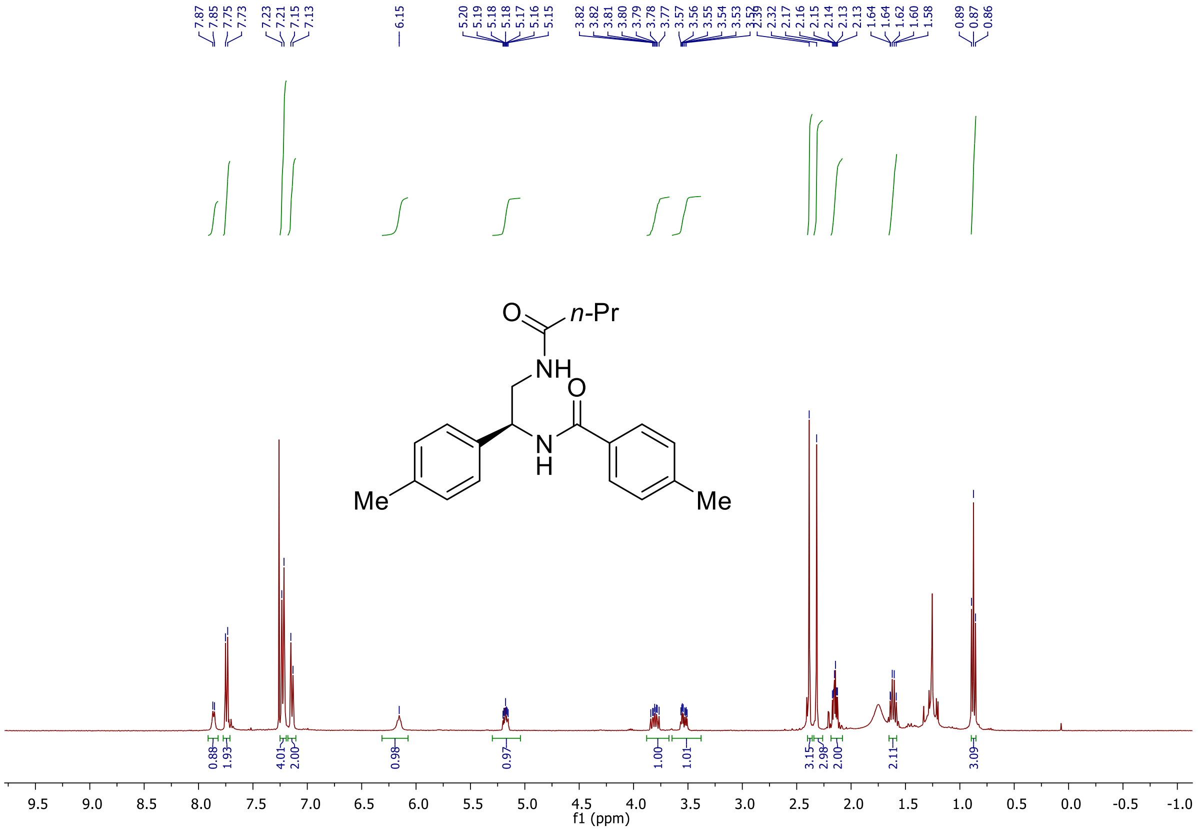

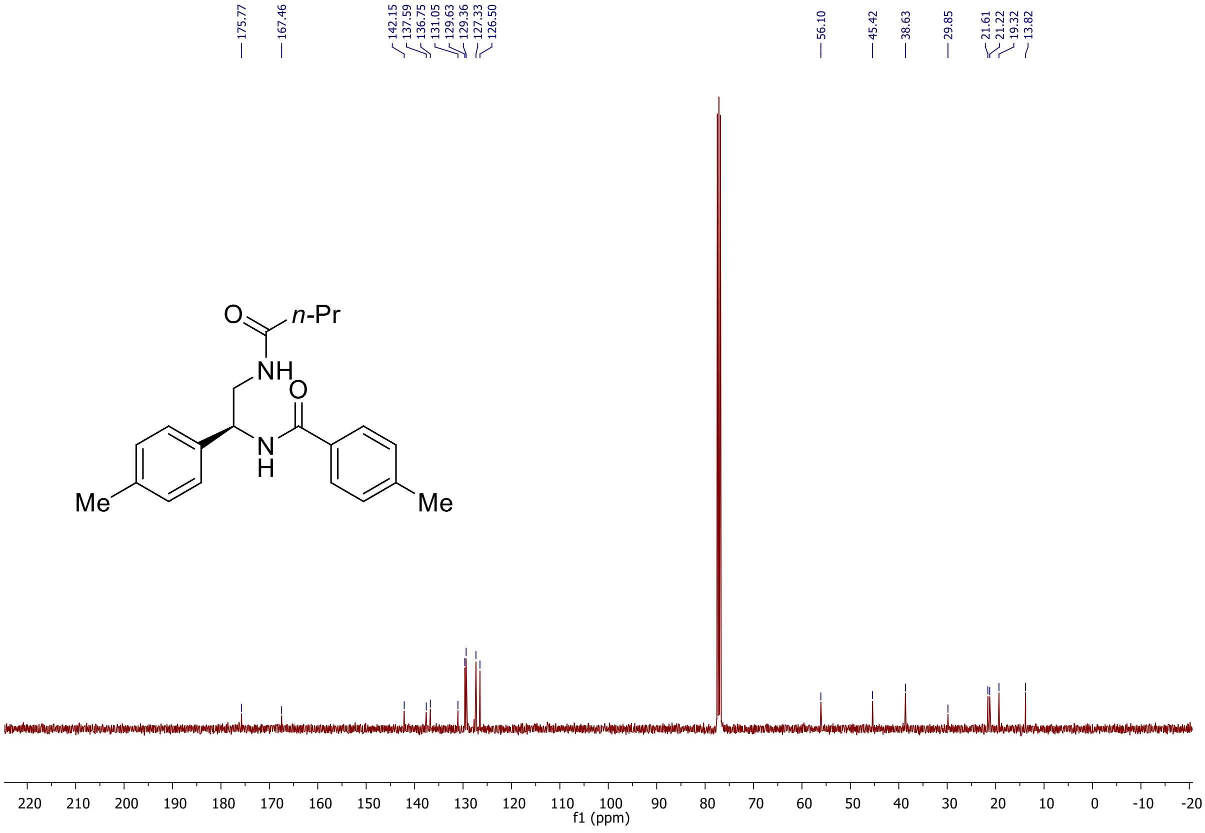

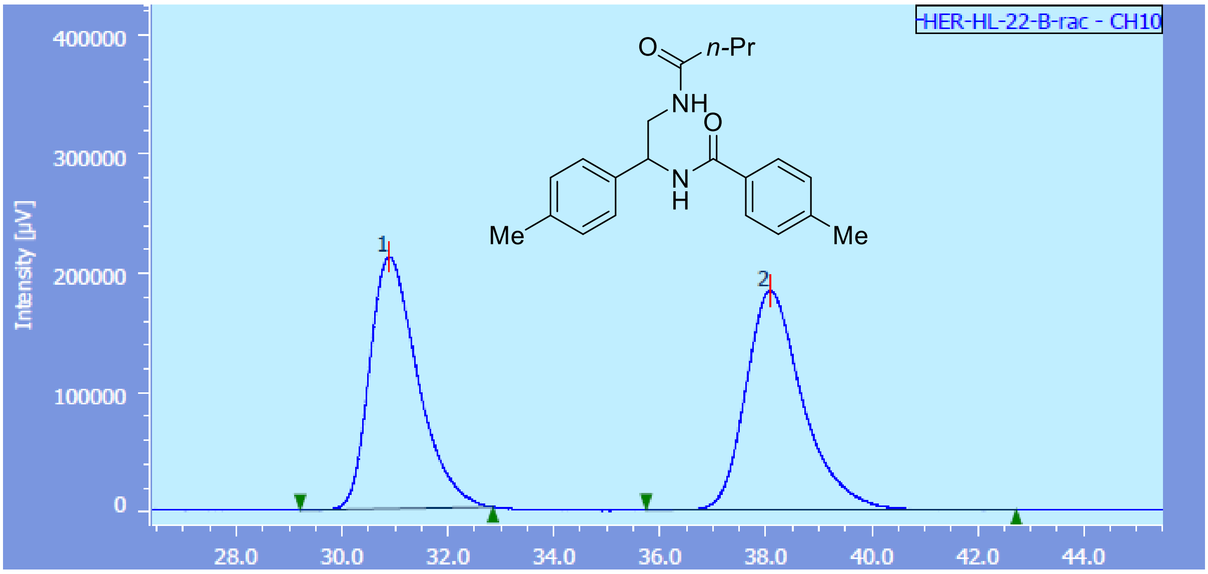

#	Peak Name	CH	tR [min]	Area [μV·sec]	Height [μV]	Area%	Height%	Quantity	NTP	Resolution	Symmetry Factor	Warning
1	Unknown	10	30.893	13342880	211274	49.312	53.502	N/A	5715	4.100	1.414	
2	Unknown	10	38.080	13715462	183613	50.688	46.498	N/A	6558	N/A	1.347	

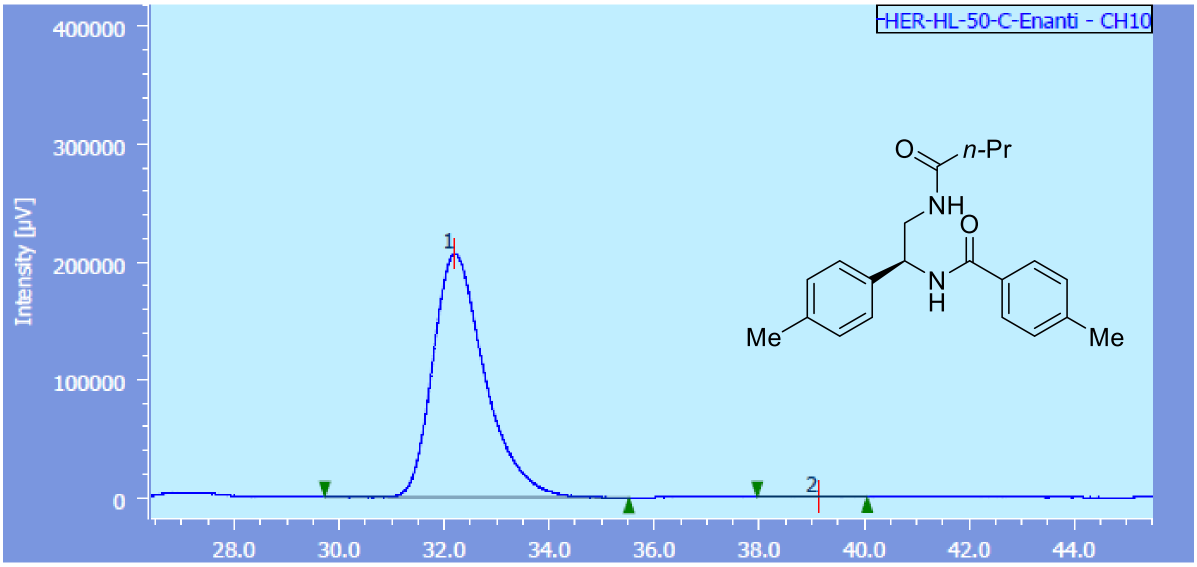

#	Peak Name	CH	tR [min]	Area [μV·sec]	Height [μV]	Area%	Height%	Quantity	NTP	Resolution	Symmetry Factor	Warning
1	Unknown	10	32.203	14205218	206825	99.857	99.776	N/A	5473	4.786	1.393	
2	Unknown	10	39.120	20350	465	0.143	0.224	N/A	18290	N/A	0.812	

(*S*)-*N*-(2-Butyramido-1-(*p*-tolyl)ethyl)- 2-methoxybenzamide (2.29)

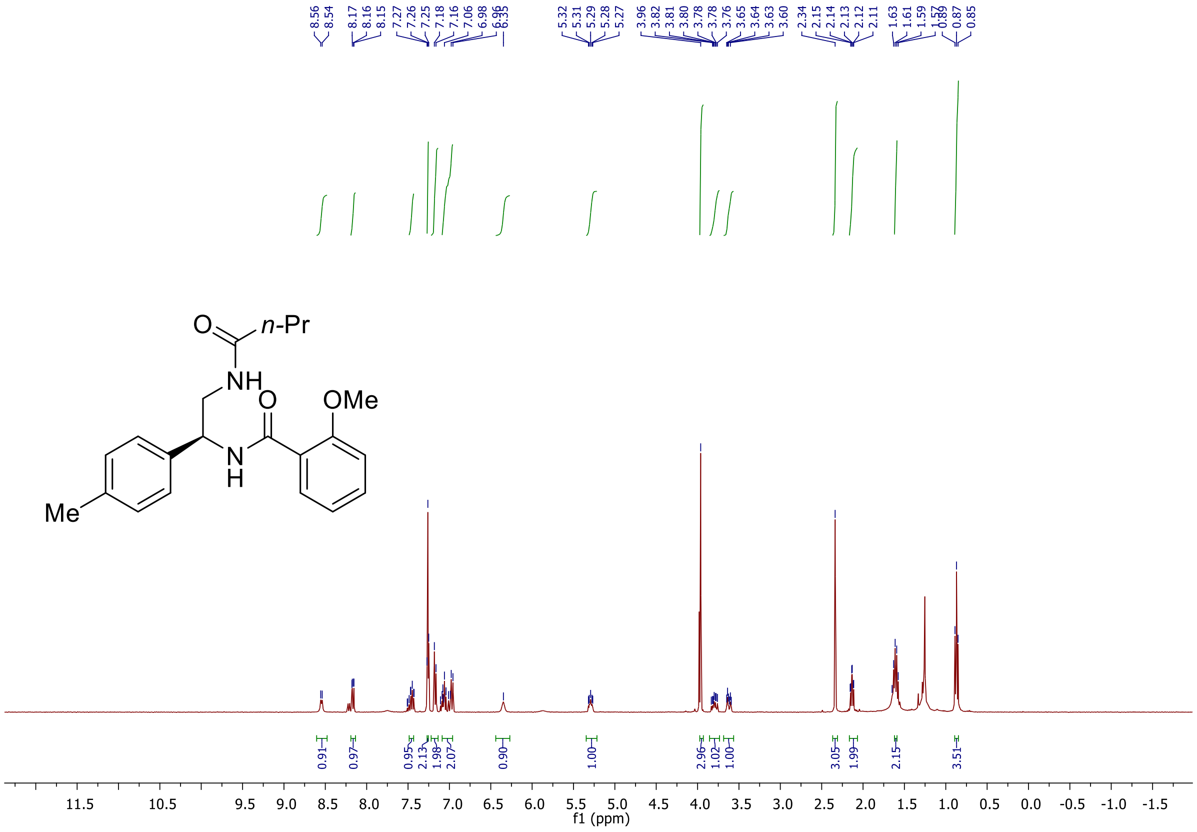

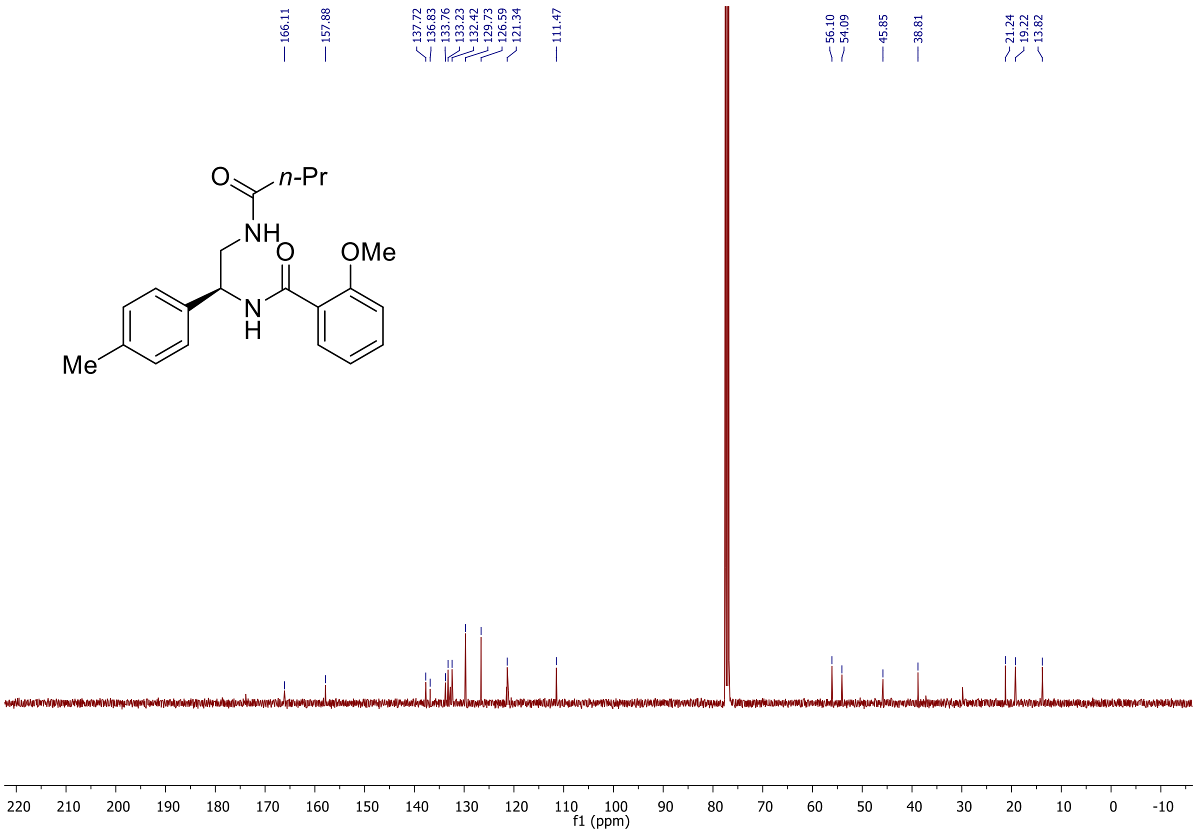

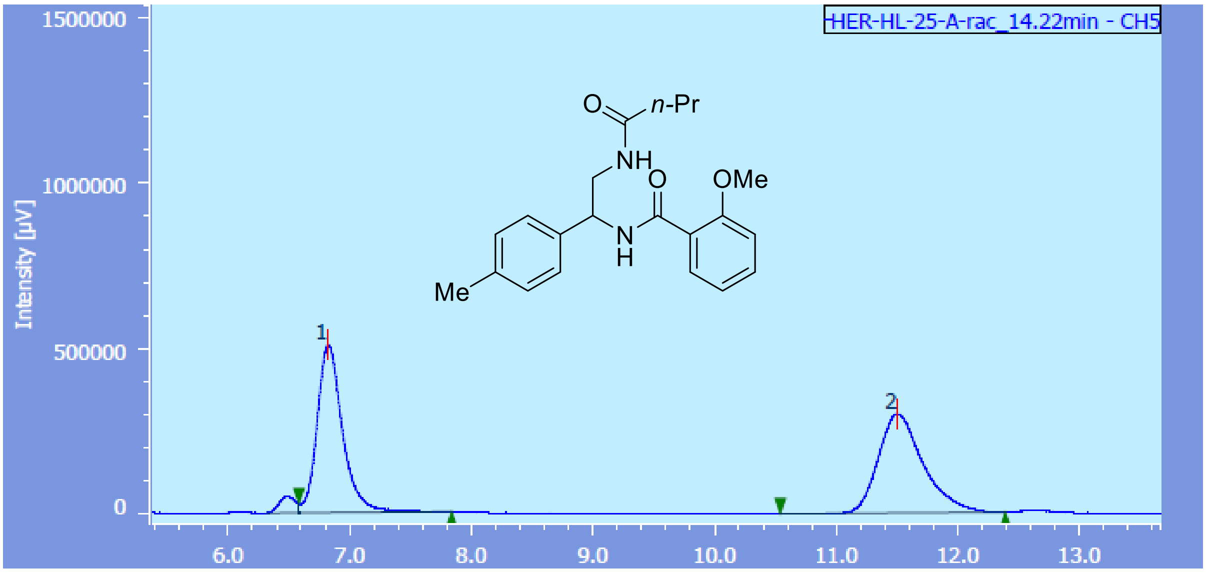

#	Peak Name	CH	tR [min]	Area [μV·sec]	Height [μV]	Area%	Height%	Quantity	NTP	Resolution	Symmetry Factor	Warning
1	Unknown	5	6.823	7379077	507303	49.052	62.904	N/A	5799	9.351	N/A	
2	Unknown	5	11.503	7664171	299171	50.948	37.096	N/A	5085	N/A	1.342	

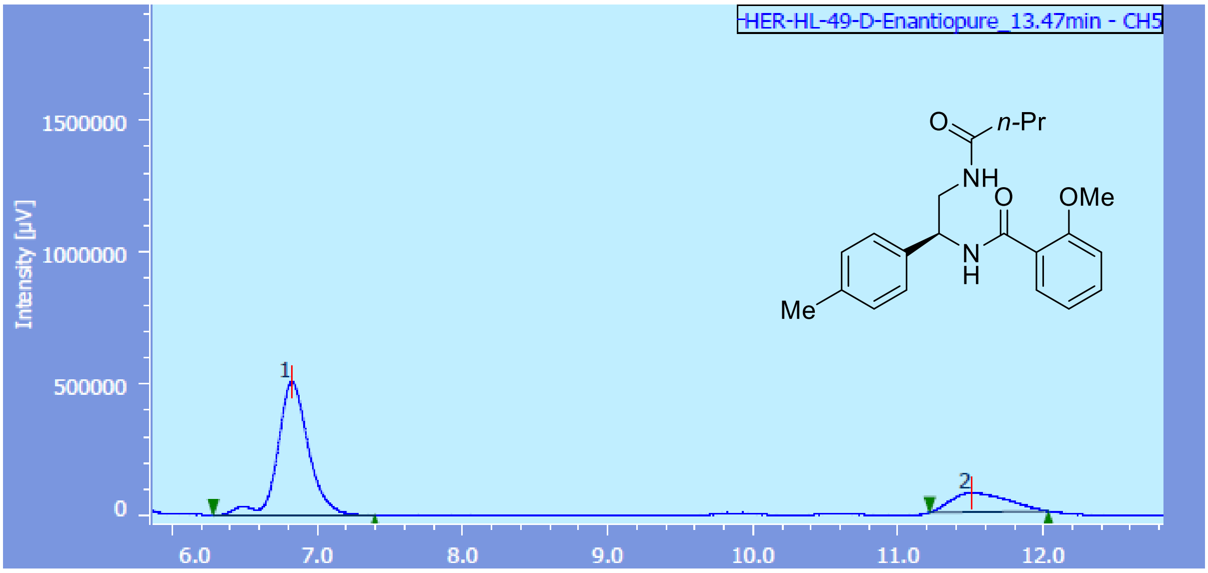

#	Peak Name	CH	tR [min]	Area [μV·sec]	Height [μV]	Area%	Height%	Quantity	NTP	Resolution	Symmetry Factor	Warning
1	Unknown	5	6.823	7415149	505222	79.108	87.296	N/A	5777	8.381	0.888	
2	Unknown	5	11.507	1958343	73521	20.892	12.704	N/A	3653	N/A	1.408	

(*S*)-*N*-(2-Butyramido-1-(*p*-tolyl)ethyl)- 2-methylbenzamide (2.30)

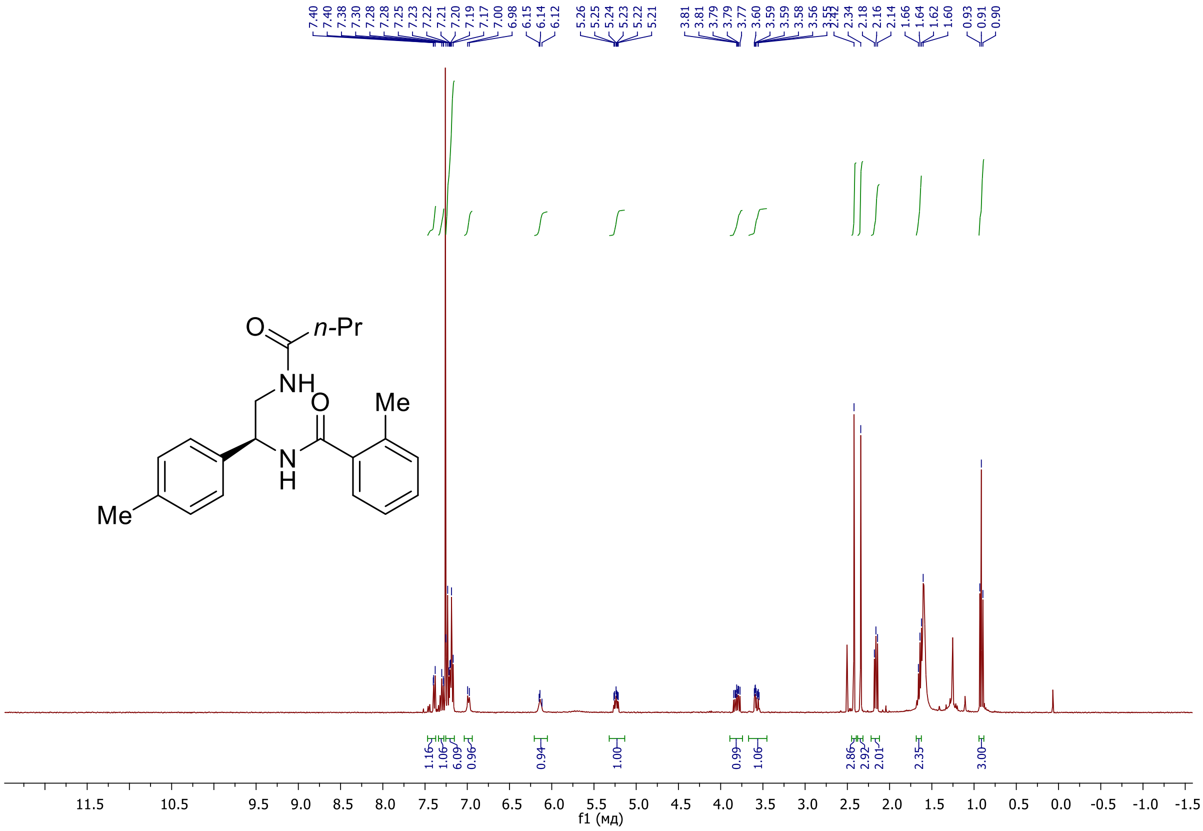

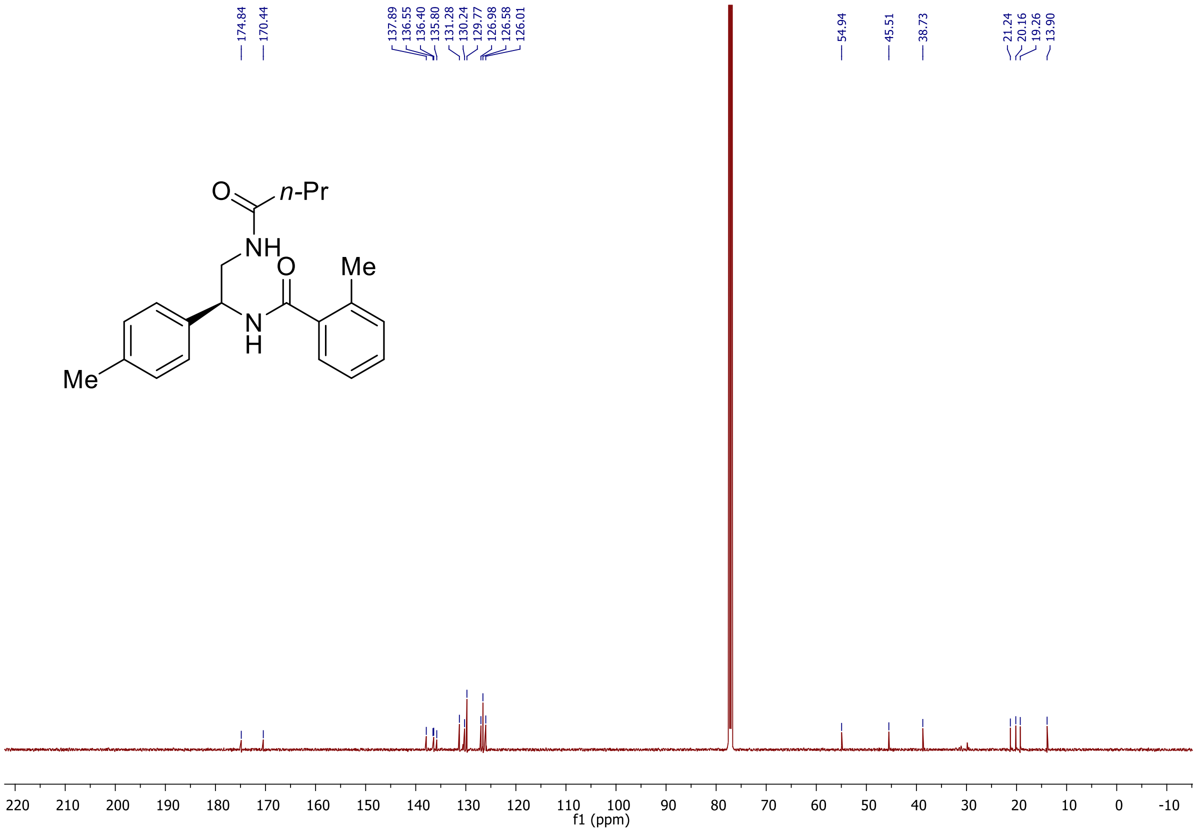

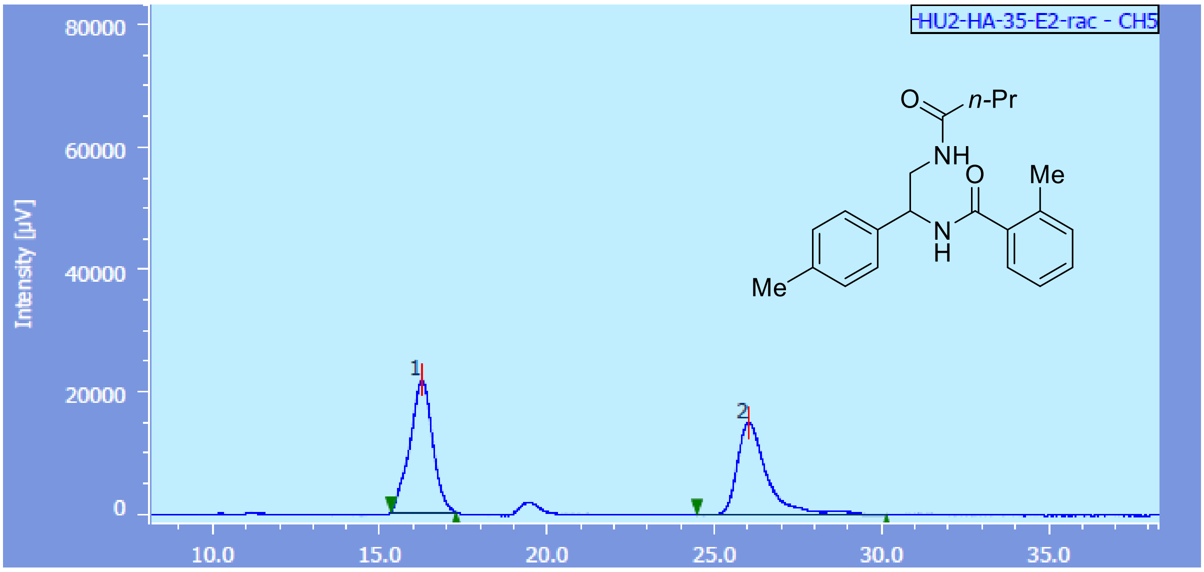

#	Peak Name	CH	tR [min]	Area [μV·sec]	Height [μV]	Area%	Height%	Quantity	NTP	Resolution	Symmetry Factor	Warning
1	Unknown	5	16.257	1023307	21675	52.366	59.065	N/A	2937	7.434	0.977	
2	Unknown	5	26.030	930848	15022	47.634	40.935	N/A	5255	N/A	1.748	

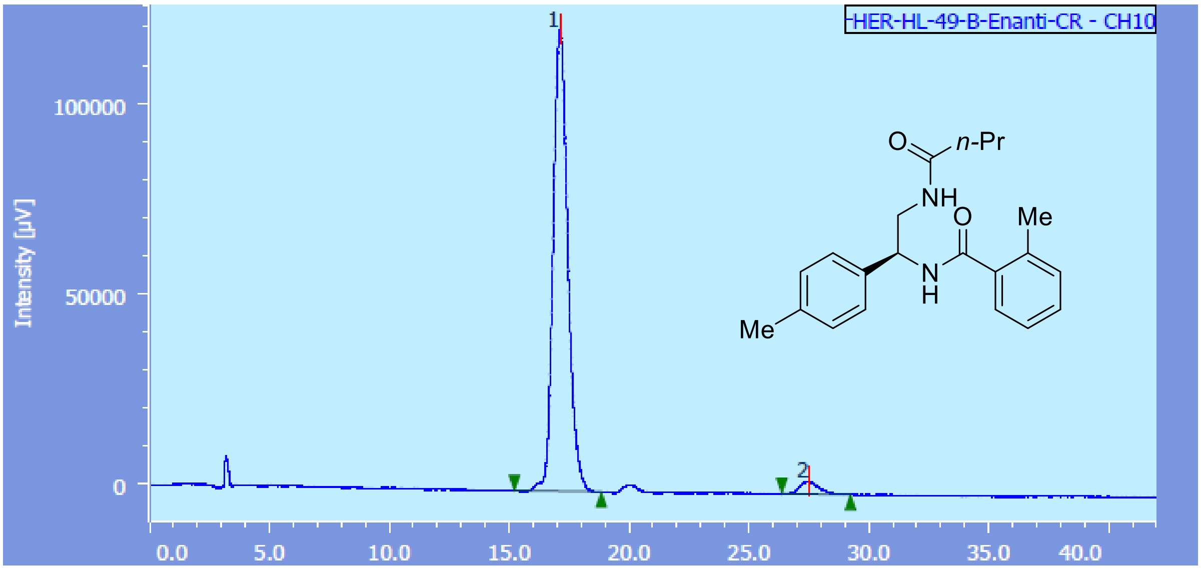

#	Peak Name	CH	tR [min]	Area [μV·sec]	Height [μV]	Area%	Height%	Quantity	NTP	Resolution	Symmetry Factor	Warning
1	Unknown	10	17.113	5129612	121162	96.462	97.309	N/A	3940	8.182	1.205	
2	Unknown	10	27.497	188130	3351	3.538	2.691	N/A	5721	N/A	1.244	

N-[(1*S*,2*R*)-2-Butyramido-1-phenylpropyl]benzamide (2.31)

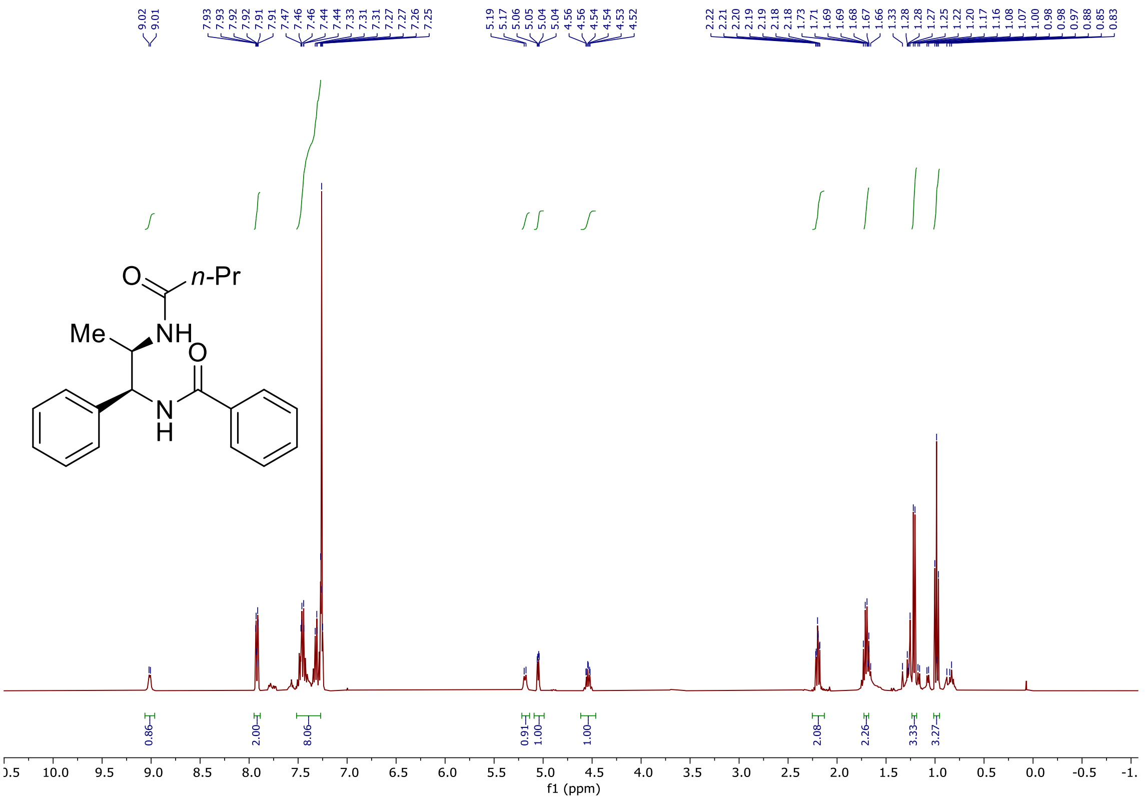

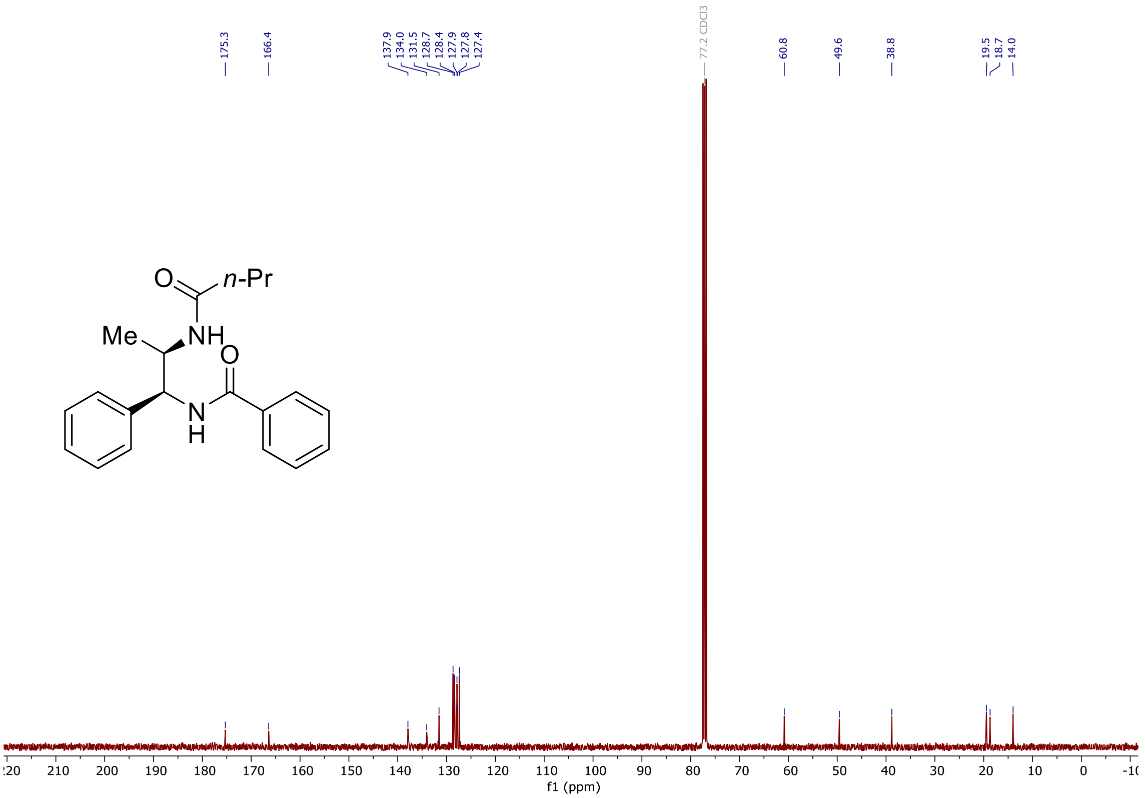

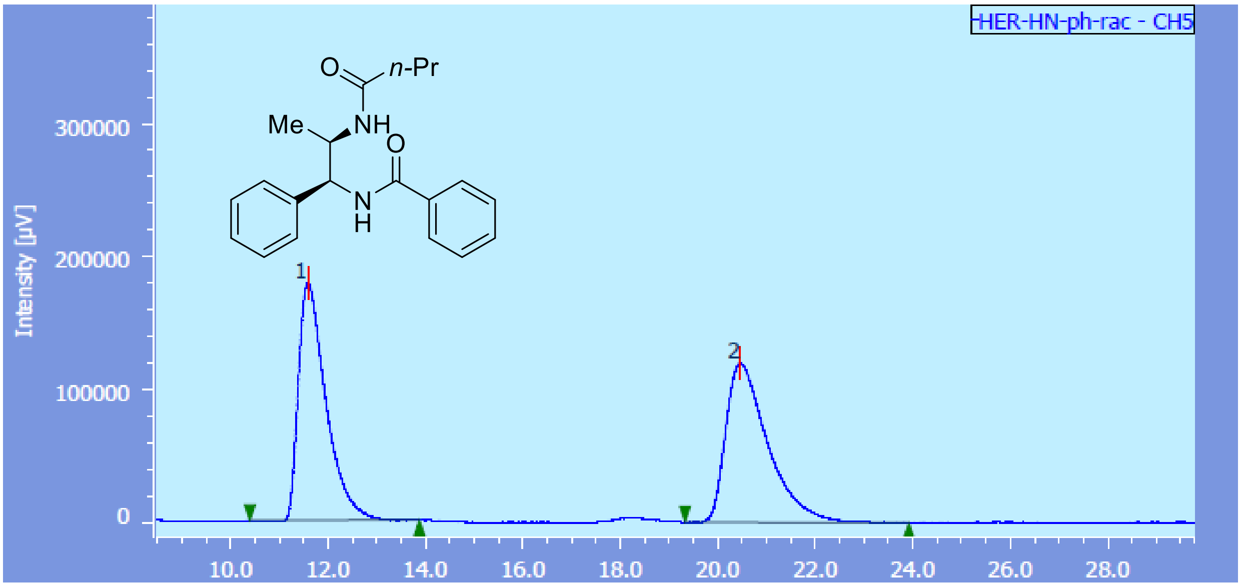

#	Peak Name	CH	tR [min]	Area [µV·sec]	Height [µV]	Area%	Height%	Quantity	NTP	Resolution	Symmetry Factor	Warning
1	Unknown	5	11.587	7014916	178680	49.333	59.852	N/A	2157	7.090	1.853	
2	Unknown	5	20.463	7204542	119854	50.667	40.148	N/A	2928	N/A	1.795	

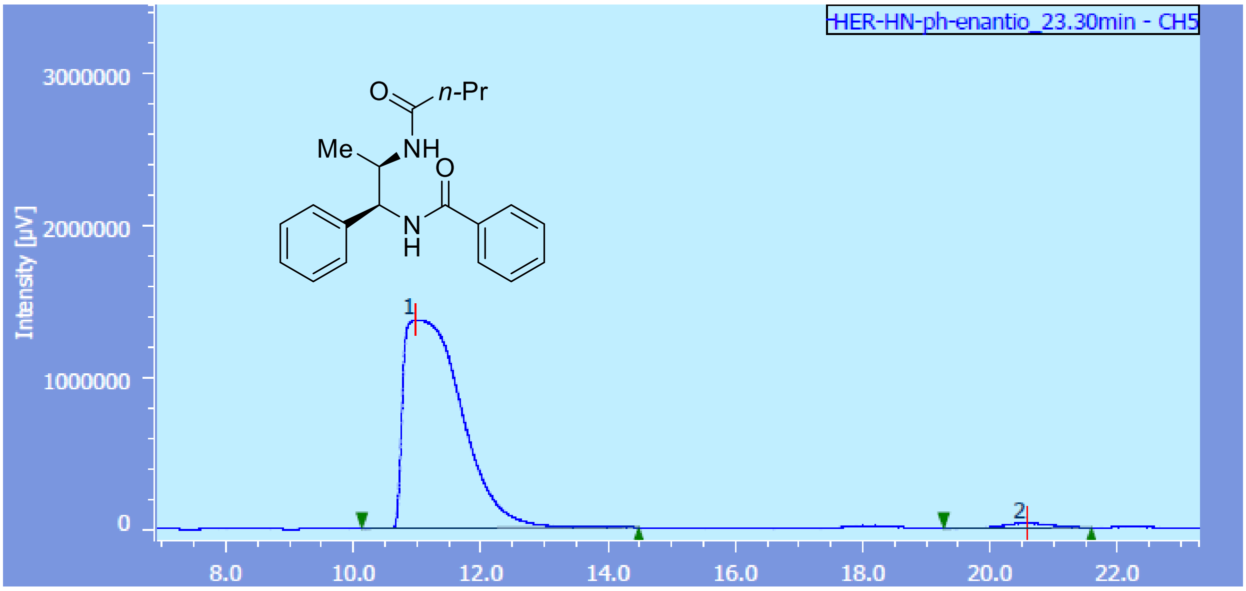

#	Peak Name	CH	tR [min]	Area [µV·sec]	Height [µV]	Area%	Height%	Quantity	NTP	Resolution	Symmetry Factor	Warning
1	Unknown	5	10.983	87420678	1370110	98.109	97.650	N/A	669	6.237	3.014	
2	Unknown	5	20.567	1685175	32971	1.891	2.350	N/A	3541	N/A	1.114	

N-((1*S*,2*R*)-2-Butyramido-1-(*p*-tolyl)propyl)benzamide (2.32)

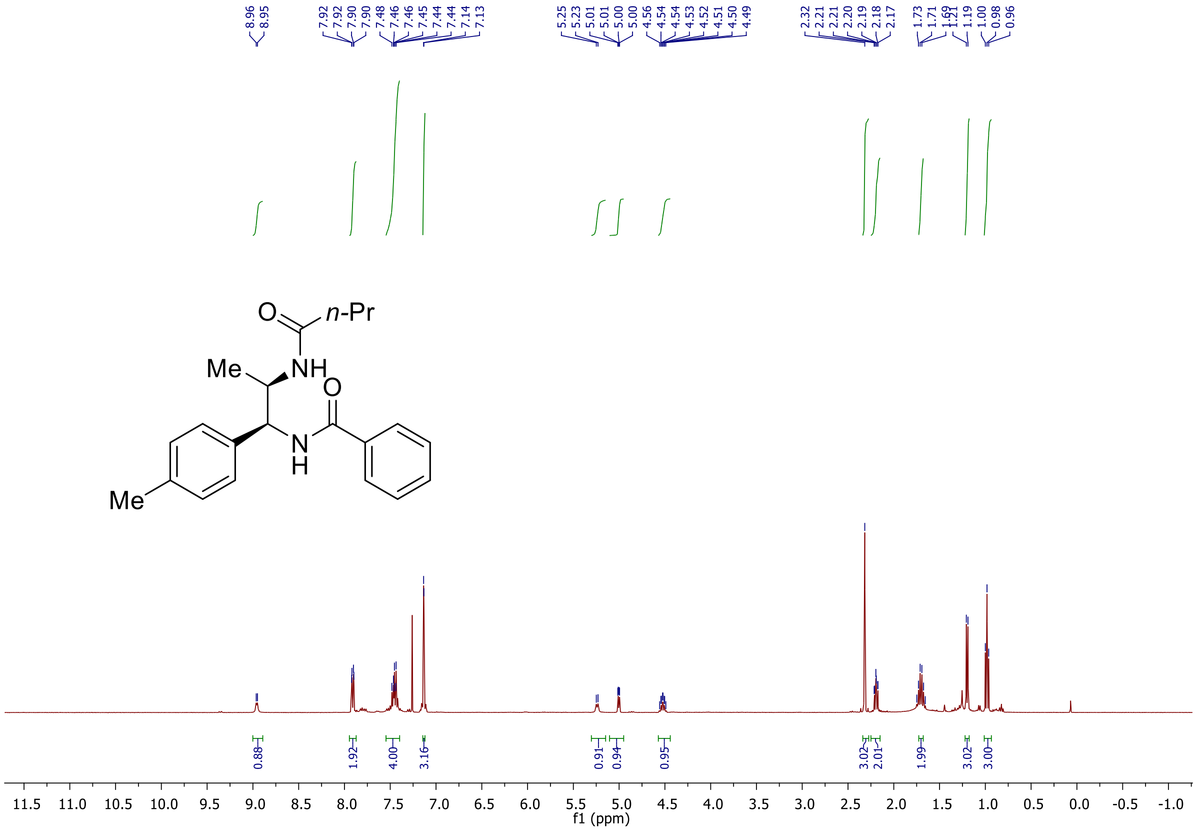

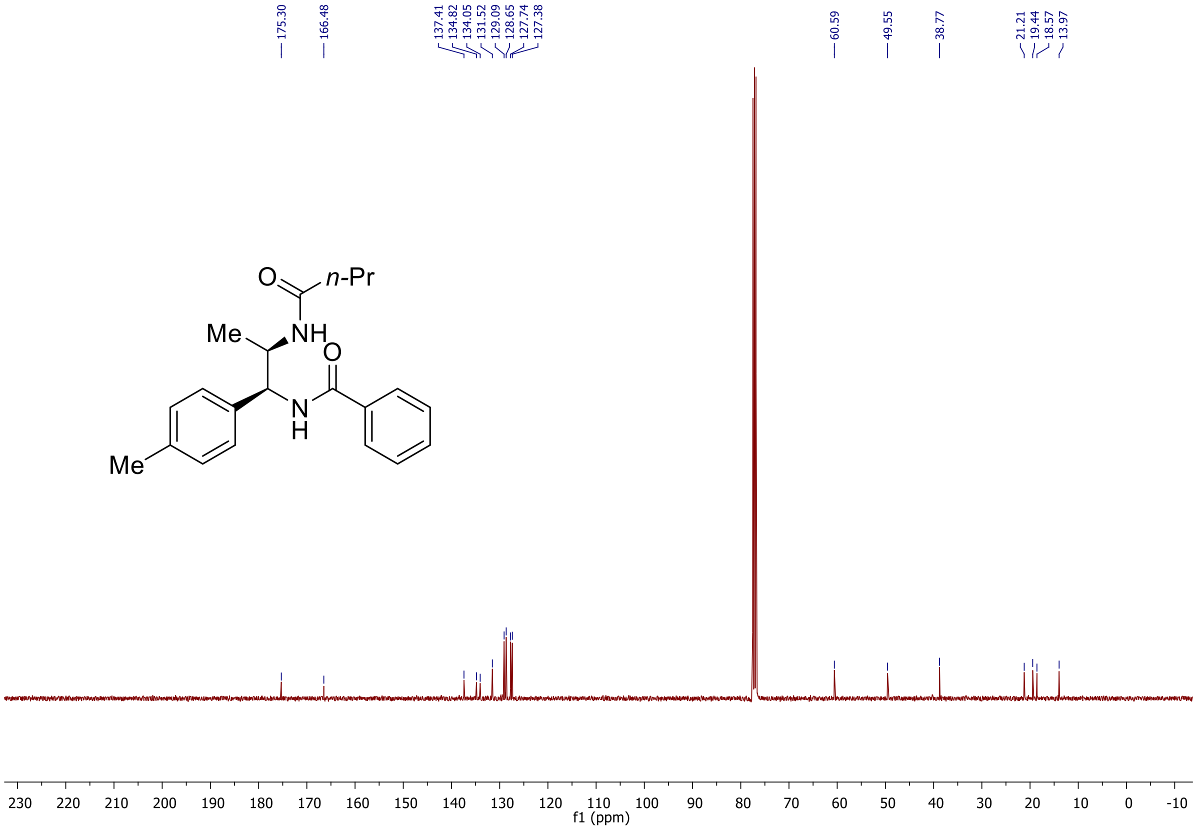

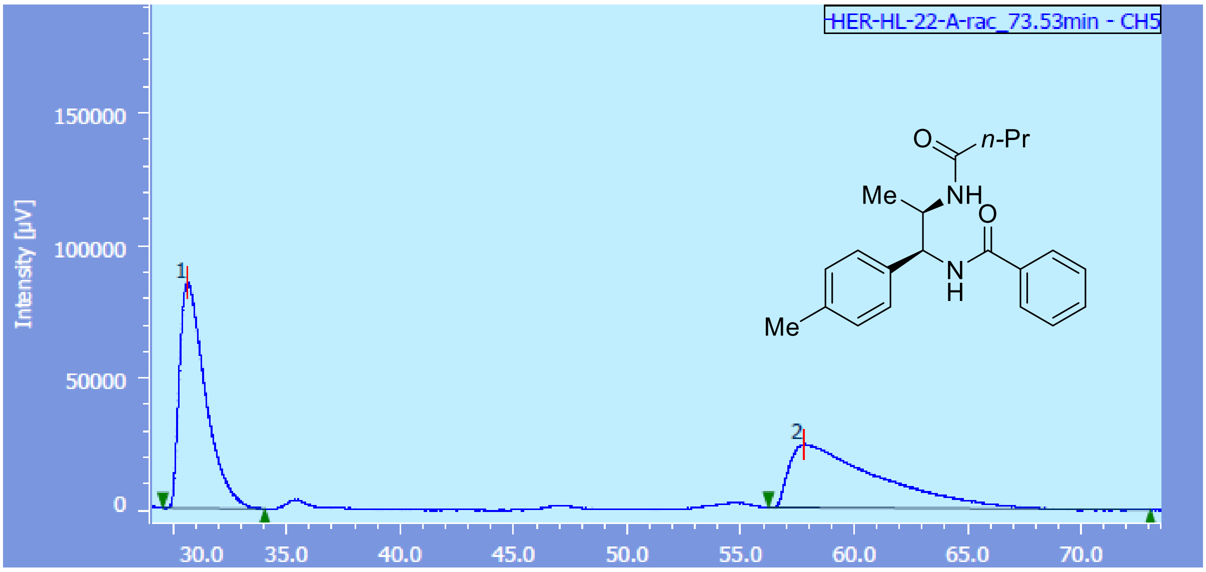

#	Peak Name	CH	tR [min]	Area [µV·sec]	Height [µV]	Area%	Height%	Quantity	NTP	Resolution	Symmetry Factor	Warning
1	Unknown	5	30.643	6807013	85158	50.992	78.090	N/A	3591	6.101	2.103	
2	Unknown	5	57.777	6542283	23893	49.008	21.910	N/A	1131	N/A	4.436	

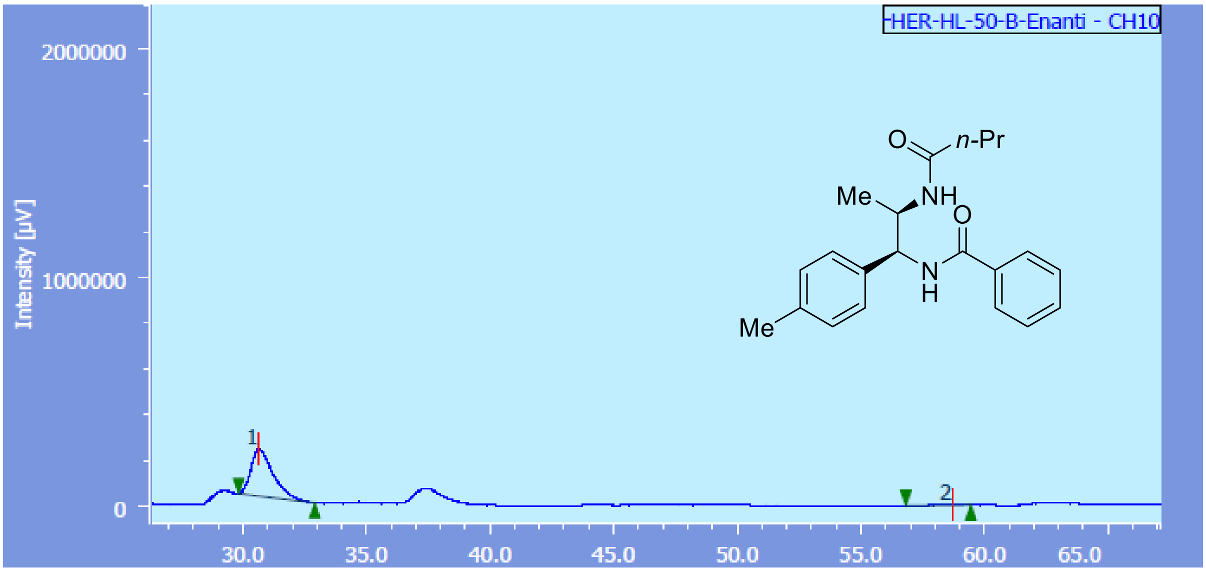

#	Peak Name	CH	tR [min]	Area [µV·sec]	Height [µV]	Area%	Height%	Quantity	NTP	Resolution	Symmetry Factor	Warning
1	Unknown	10	30.660	12808056	207339	98.157	98.366	N/A	6148	15.869	1.556	
2	Unknown	10	58.700	240527	3444	1.843	1.634	N/A	14073	N/A	0.802	

N-[(1*S*,2*R*)-2-Butyramido-1-(4-methoxyphenyl)propyl]benzamide (2.33)

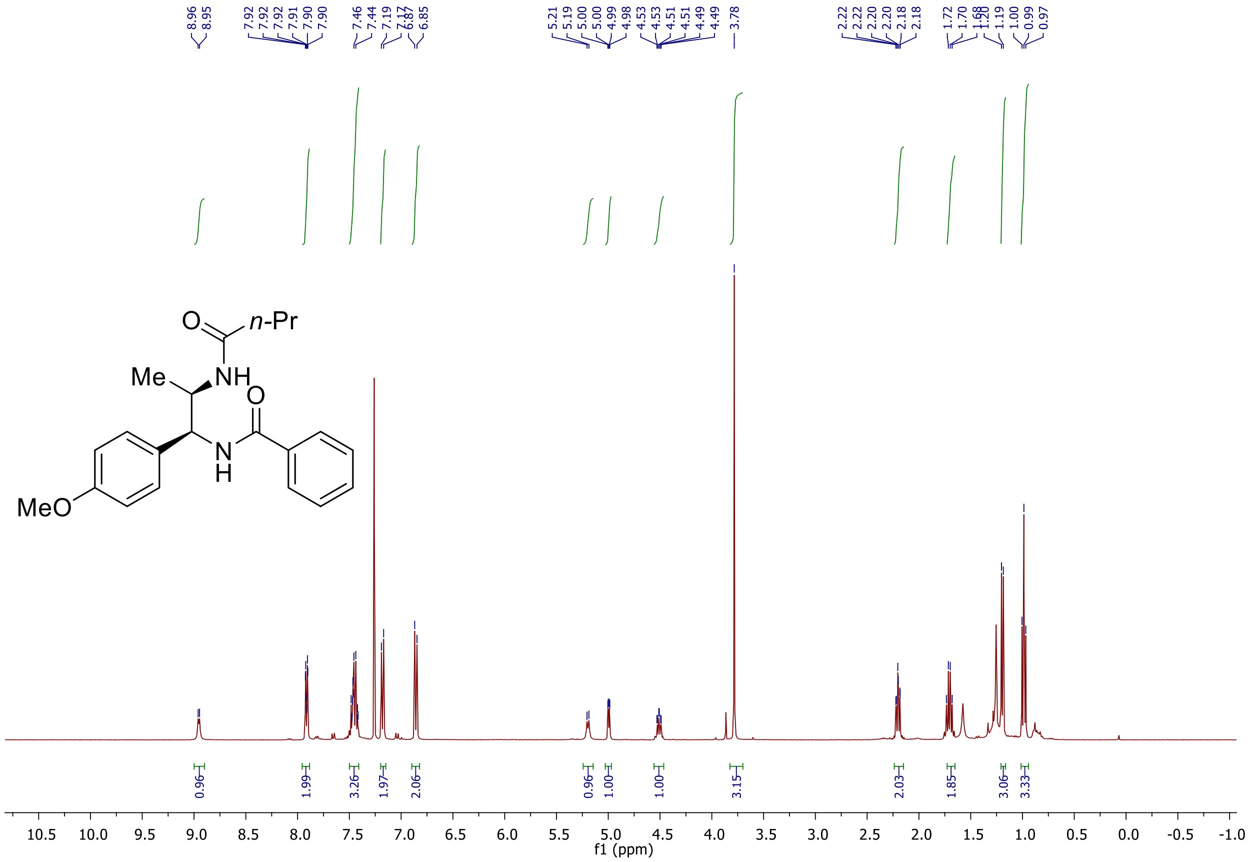

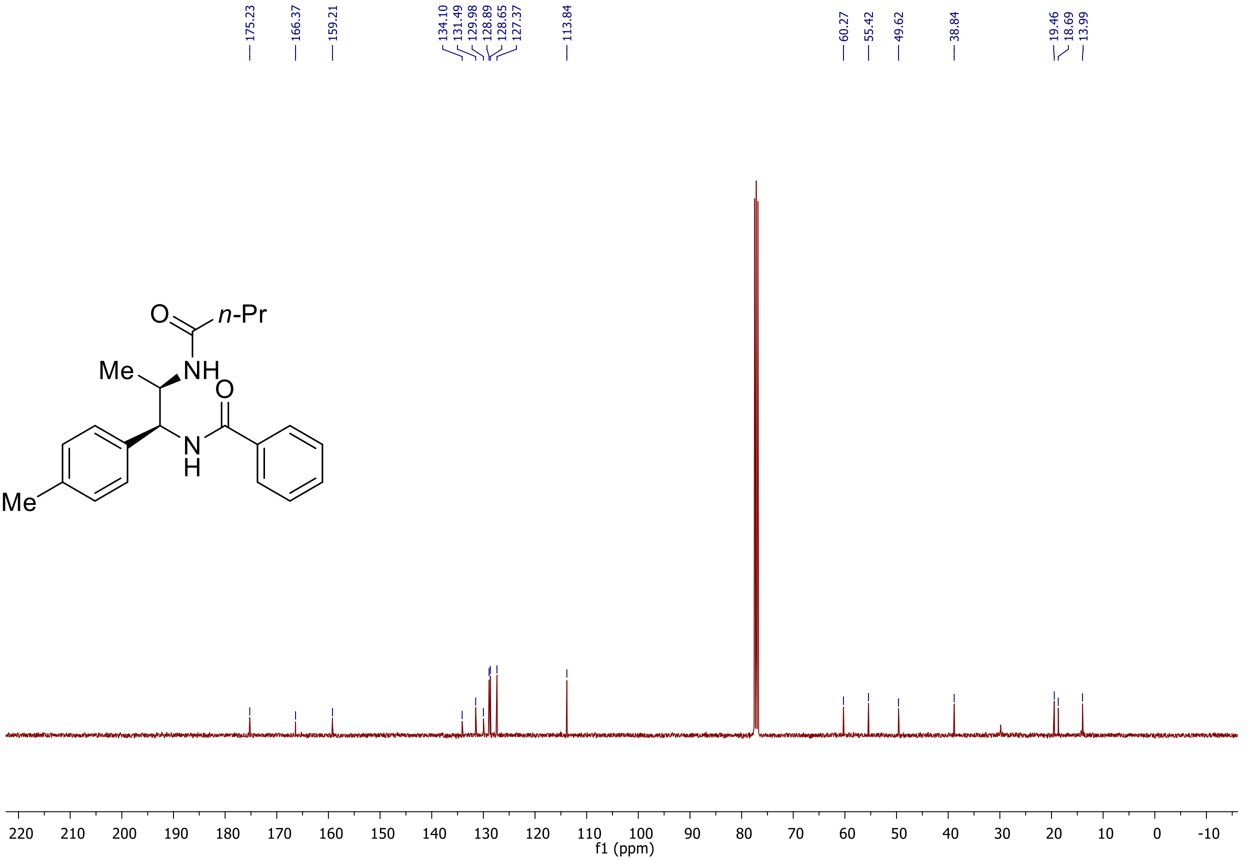

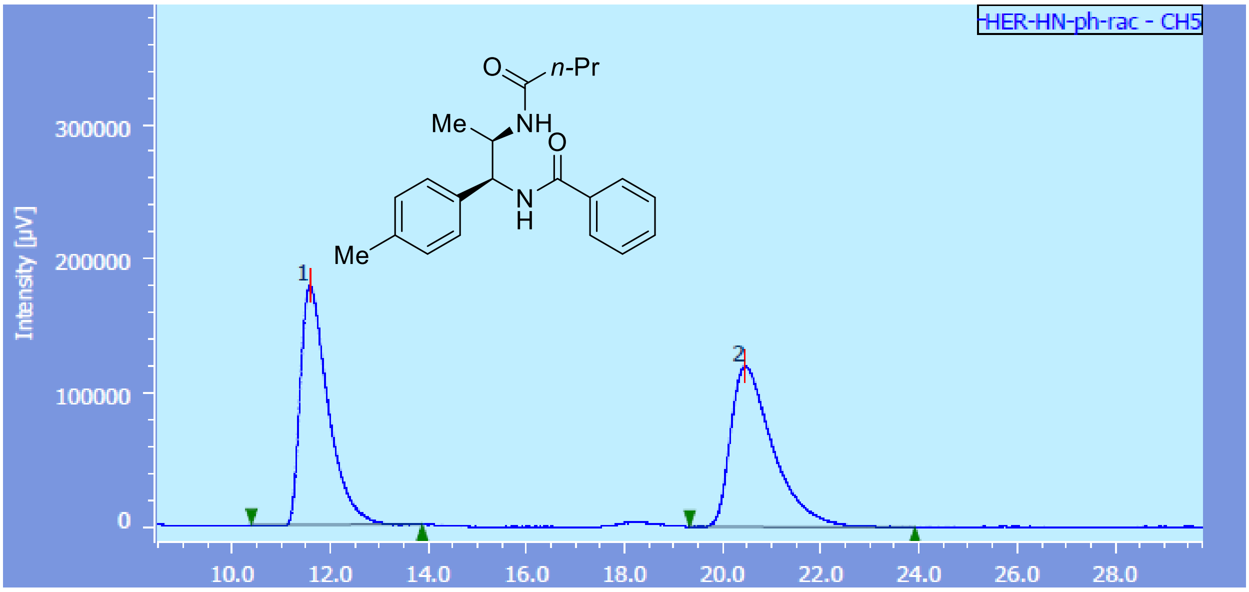

#	Peak Name	CH	tR [min]	Area [µV·sec]	Height [µV]	Area%	Height%	Quantity	NTP	Resolution	Symmetry Factor	Warning
1	Unknown	5	11.587	7014916	178680	49.333	59.852	N/A	2157	7.090	1.853	
2	Unknown	5	20.463	7204542	119854	50.667	40.148	N/A	2928	N/A	1.795	

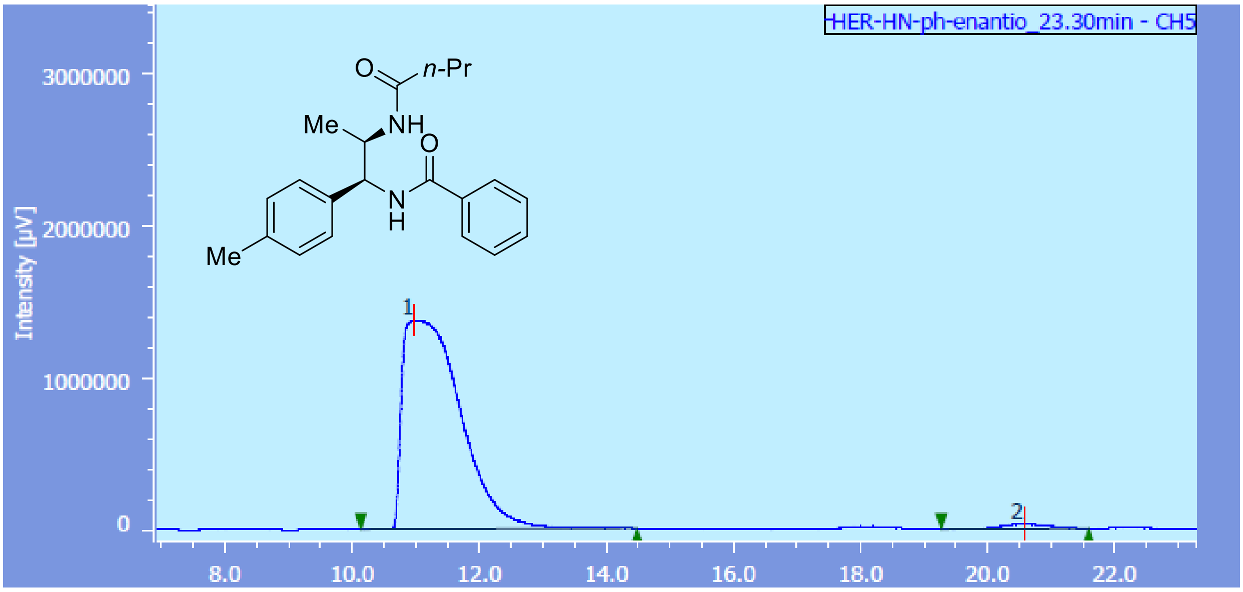

#	Peak Name	CH	tR [min]	Area [µV·sec]	Height [µV]	Area%	Height%	Quantity	NTP	Resolution	Symmetry Factor	Warning
1	Unknown	5	10.983	87420678	1370110	98.109	97.650	N/A	669	6.237	3.014	
2	Unknown	5	20.567	1685175	32971	1.891	2.350	N/A	3541	N/A	1.114	

(*S*)-*N*-(1-Butyramido-2-(*p*-tolyl)propan-2-yl)benzamide (2.34)

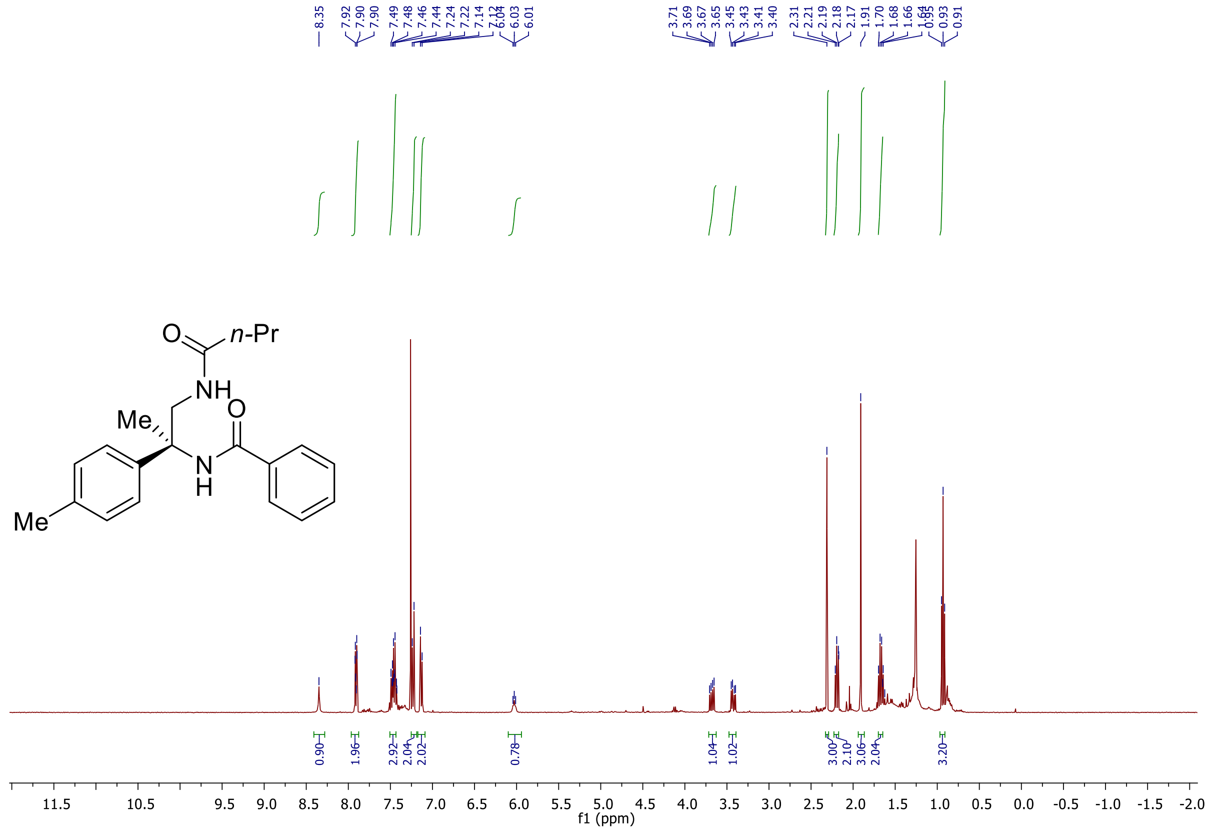

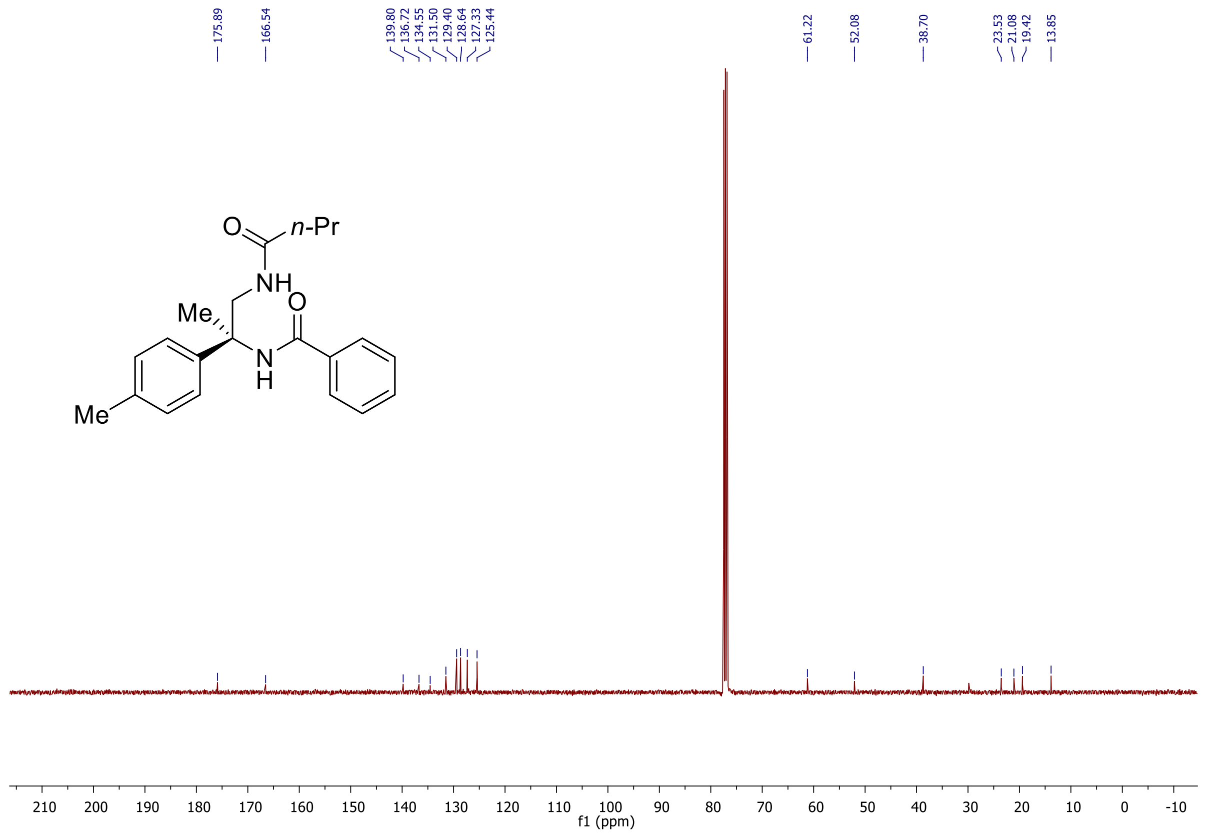

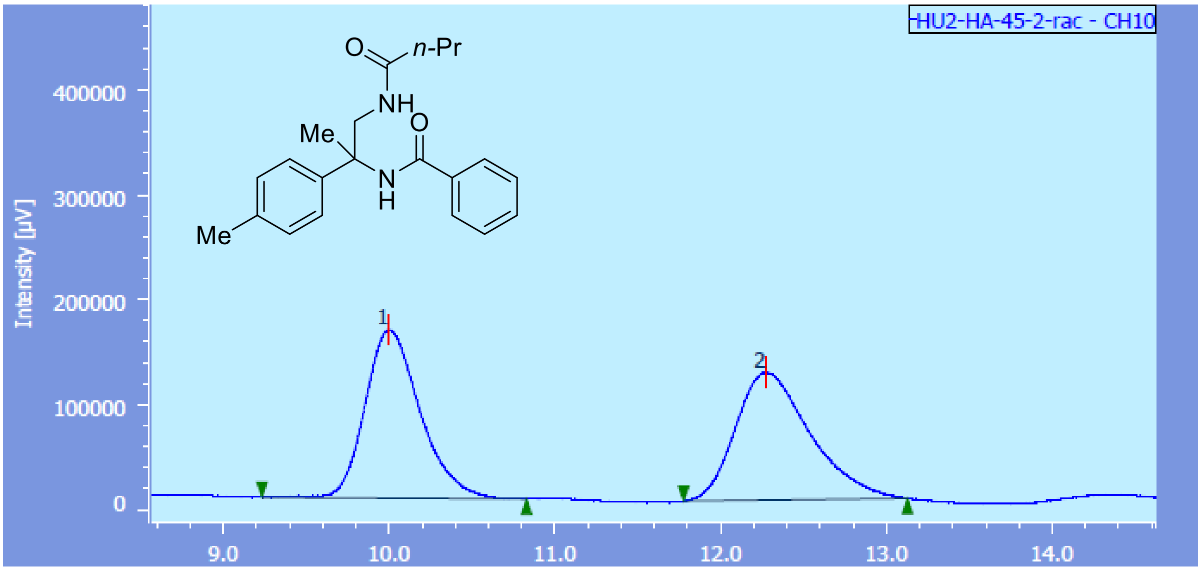

#	Peak Name	CH	tR [min]	Area [µV·sec]	Height [µV]	Area%	Height%	Quantity	NTP	Resolution	Symmetry Factor	Warning
1	Unknown	10	10.000	3712739	160557	49.313	56.880	N/A	4577	3.254	1.271	
2	Unknown	10	12.273	3816247	121715	50.687	43.120	N/A	3677	N/A	1.384	

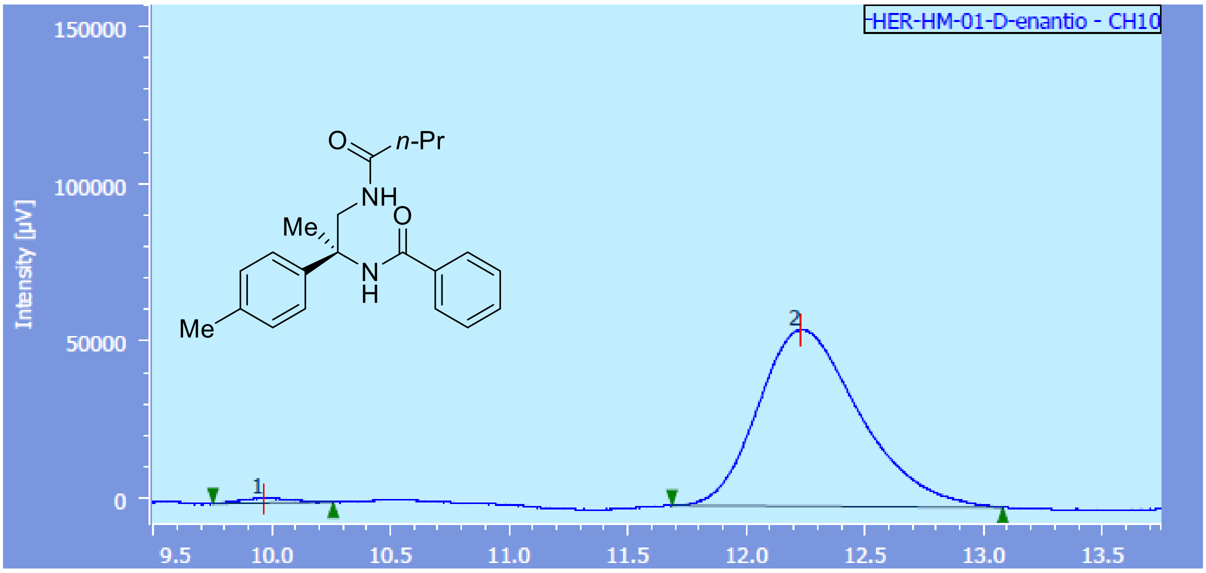

#	Peak Name	CH	tR [min]	Area [µV·sec]	Height [µV]	Area%	Height%	Quantity	NTP	Resolution	Symmetry Factor	Warning
1	Unknown	10	9.967	22413	1486	1.321	2.581	N/A	8483	3.758	1.132	
2	Unknown	10	12.230	1674228	56091	98.679	97.419	N/A	3986	N/A	1.305	

(*S*)-*N*-(2-Butoxy-2-(*p*-tolyl)ethyl)butyramide (2.35)

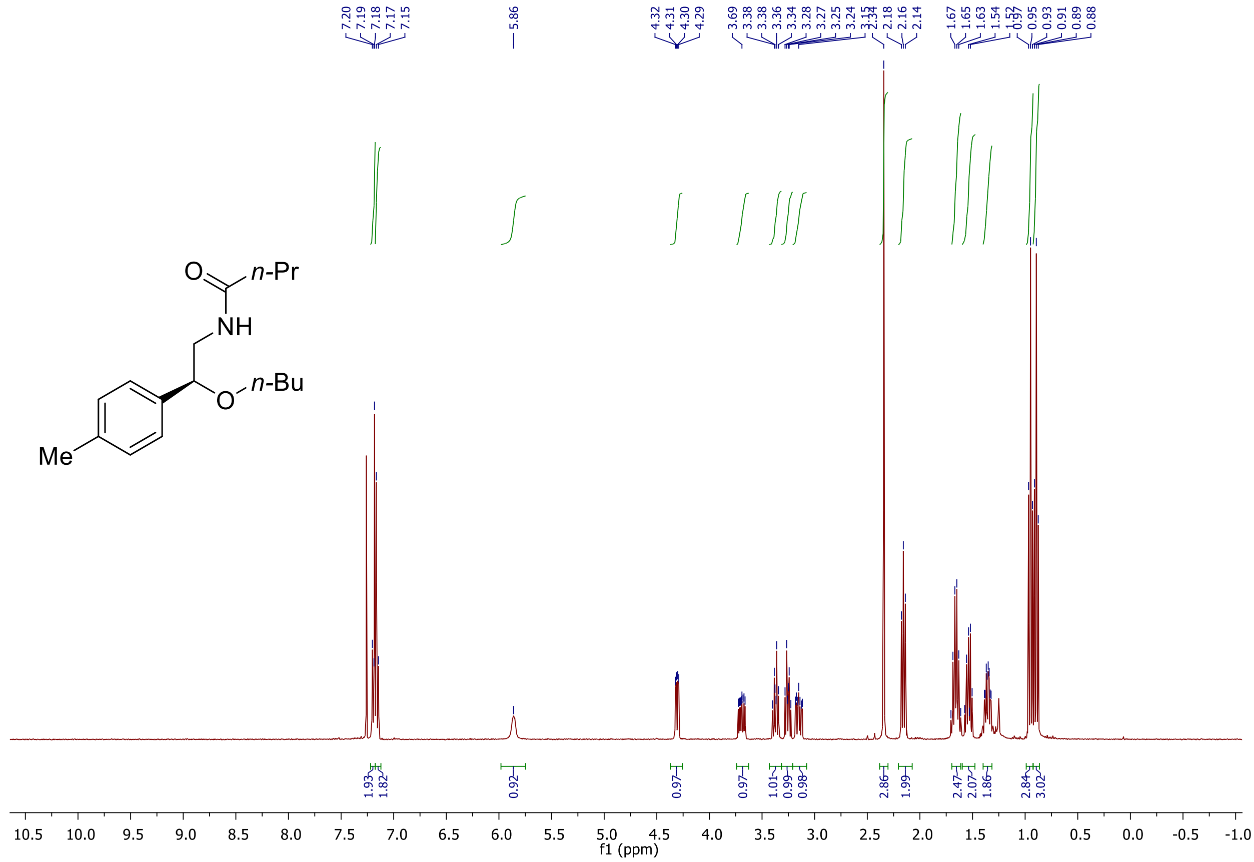

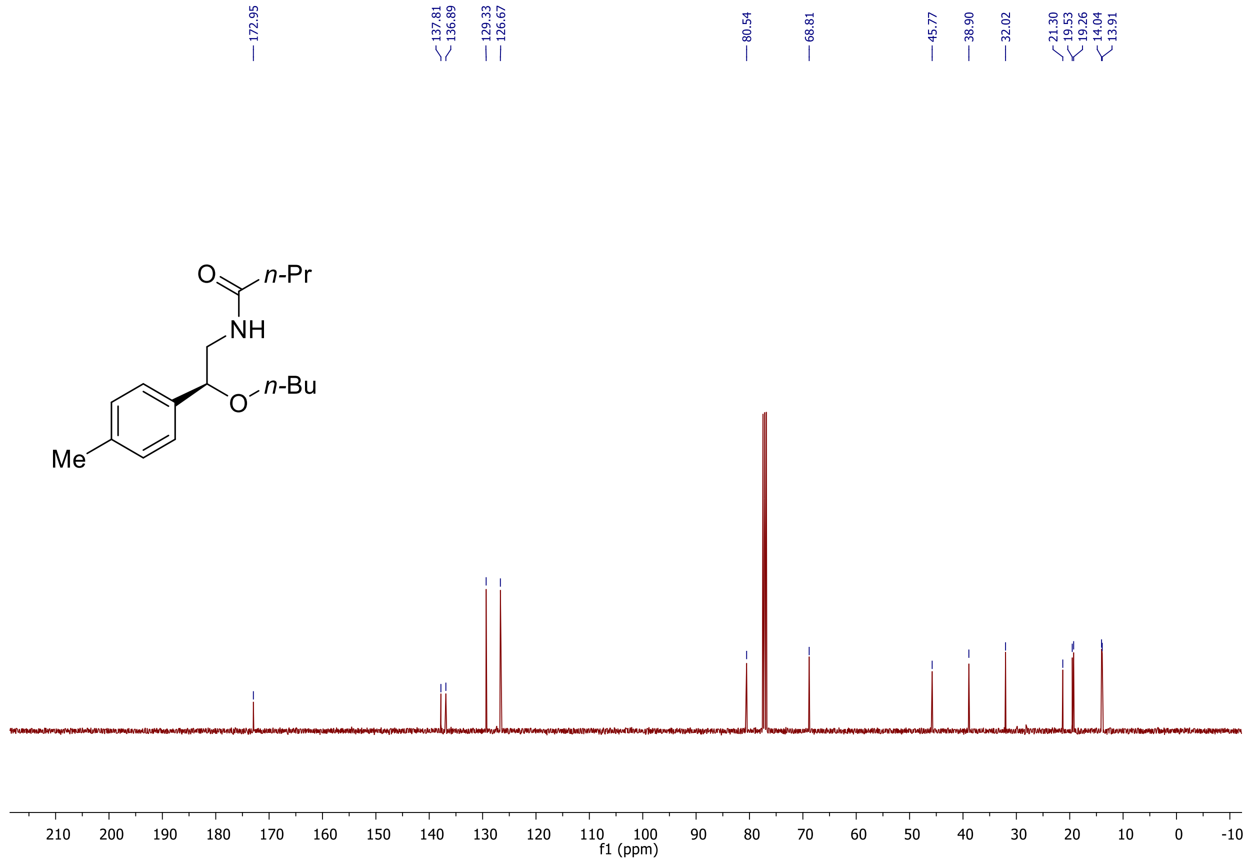

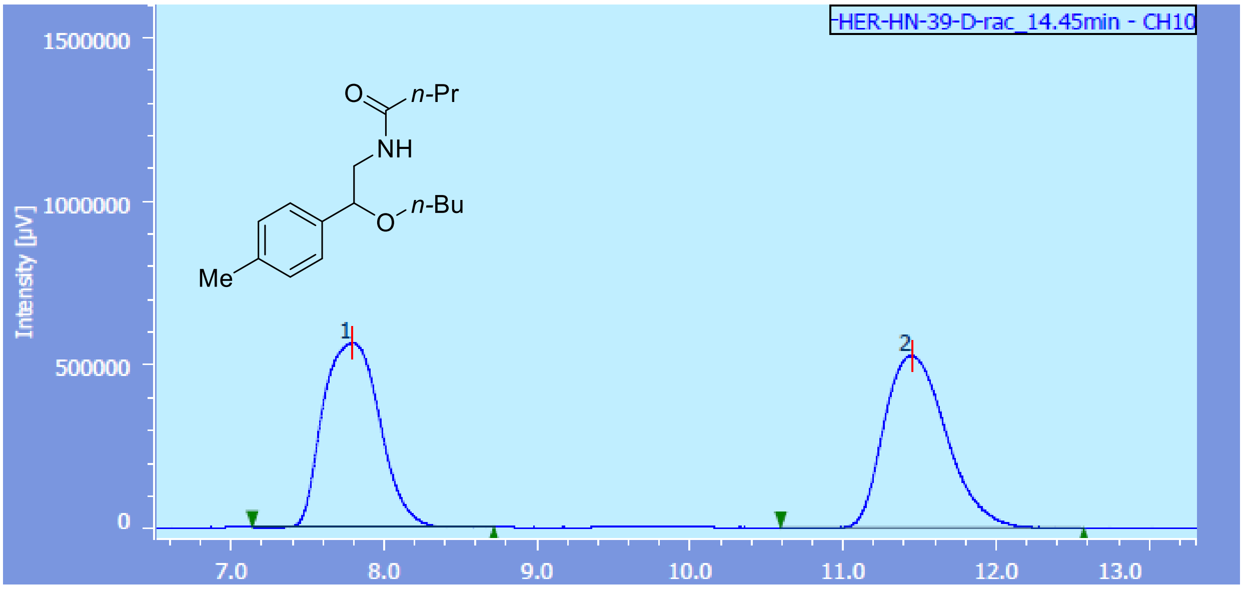

#	Peak Name	CH	tR [min]	Area [μV·sec]	Height [μV]	Area%	Height%	Quantity	NTP	Resolution	Symmetry Factor	Warning
1	Unknown	10	7.793	14516584	562472	49.348	51.727	N/A	1891	4.966	1.122	
2	Unknown	10	11.450	14900195	524924	50.652	48.273	N/A	3635	N/A	1.292	

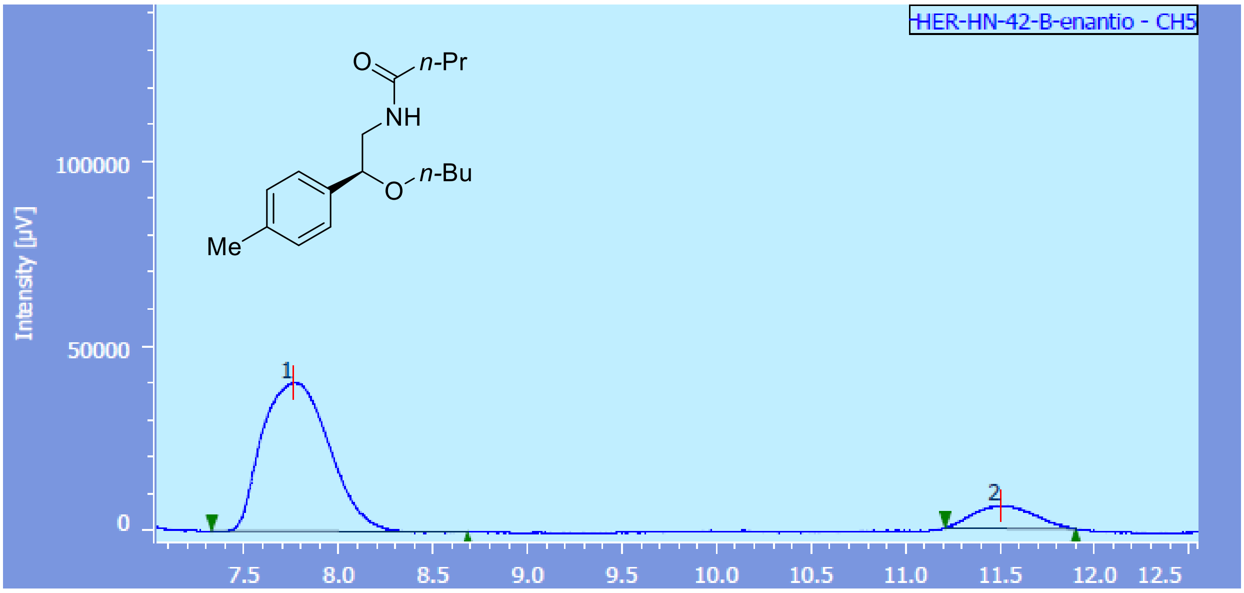

#	Peak Name	CH	tR [min]	Area [μV·sec]	Height [μV]	Area%	Height%	Quantity	NTP	Resolution	Symmetry Factor	Warning
1	Unknown	5	7.763	994761	40192	87.807	86.817	N/A	2011	5.624	1.236	
2	Unknown	5	11.507	138135	6103	12.193	13.183	N/A	5134	N/A	1.155	

(*S*)-*N*-[2-*tert*-Butoxy-2-(*p*-tolyl)ethyl]butyramide (2.36)

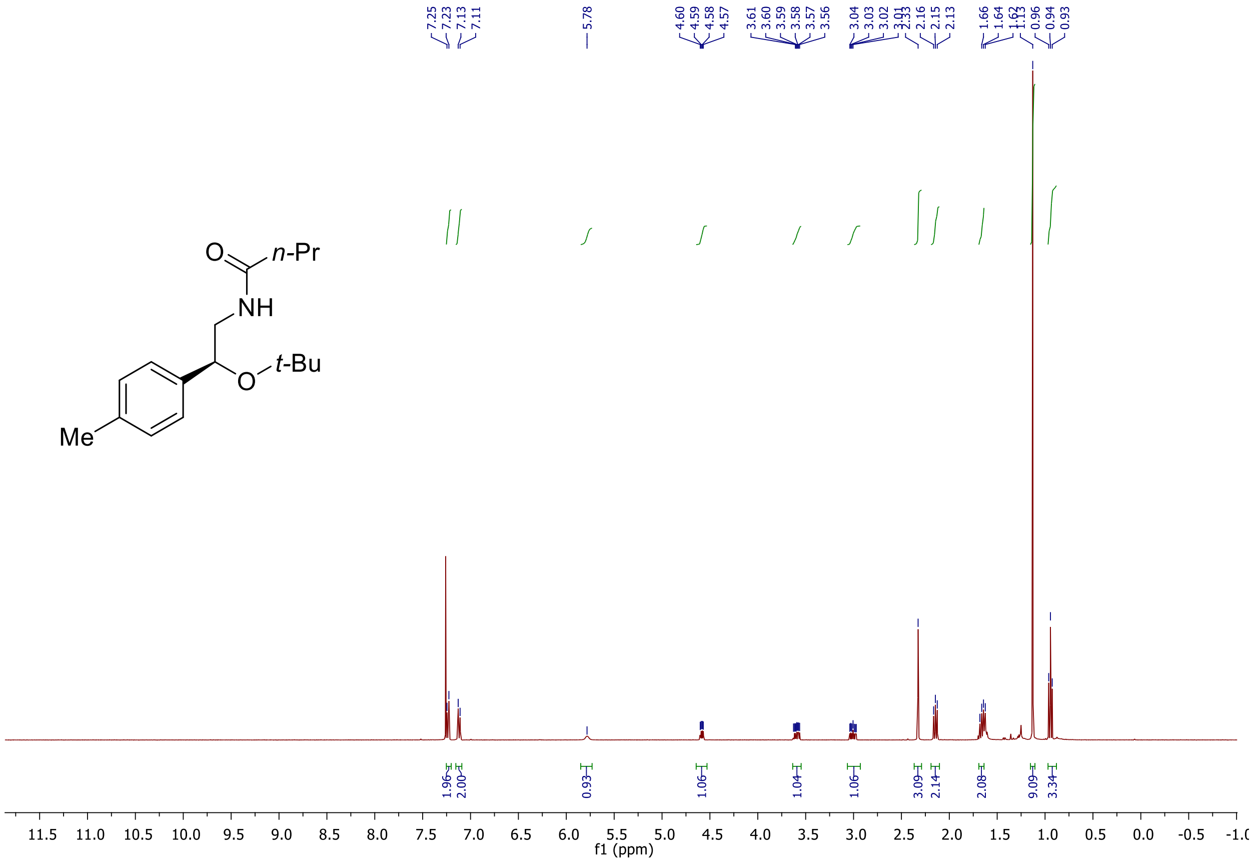

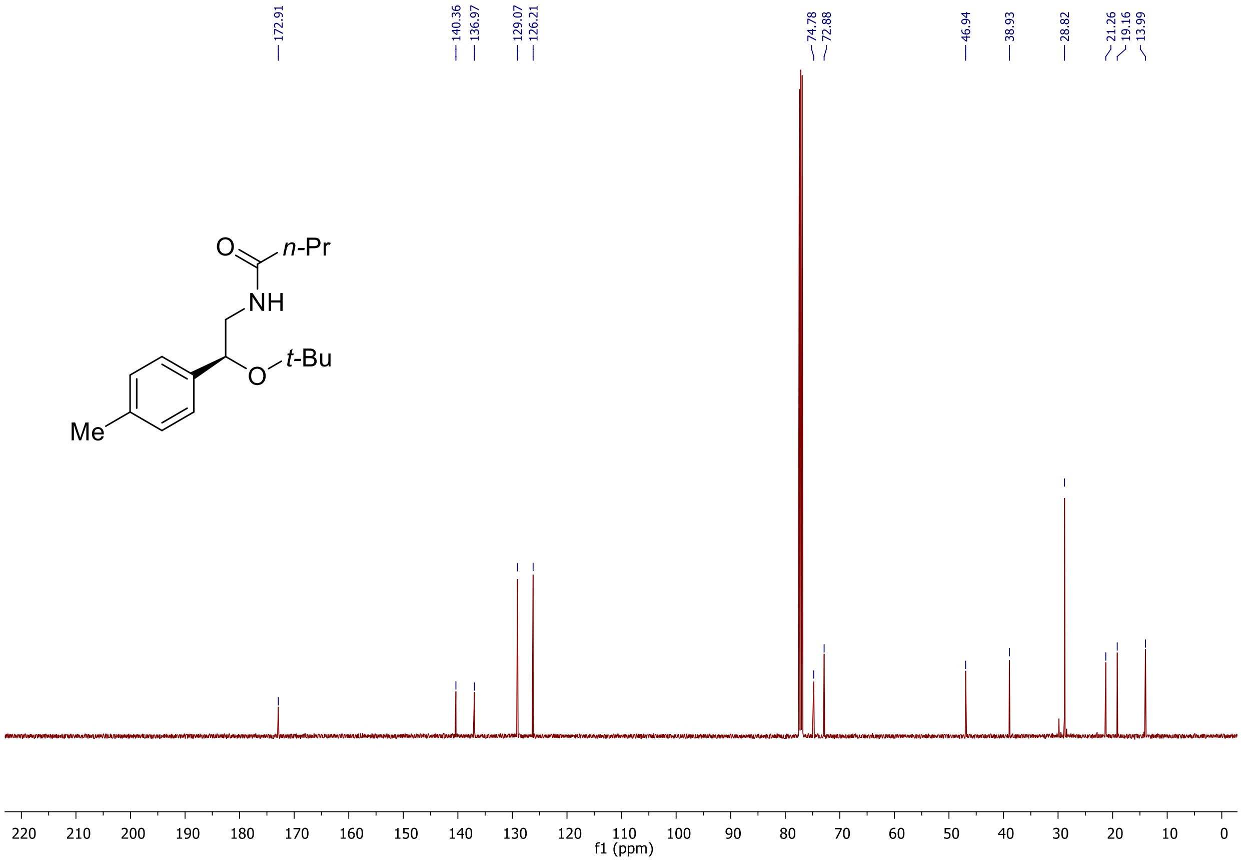

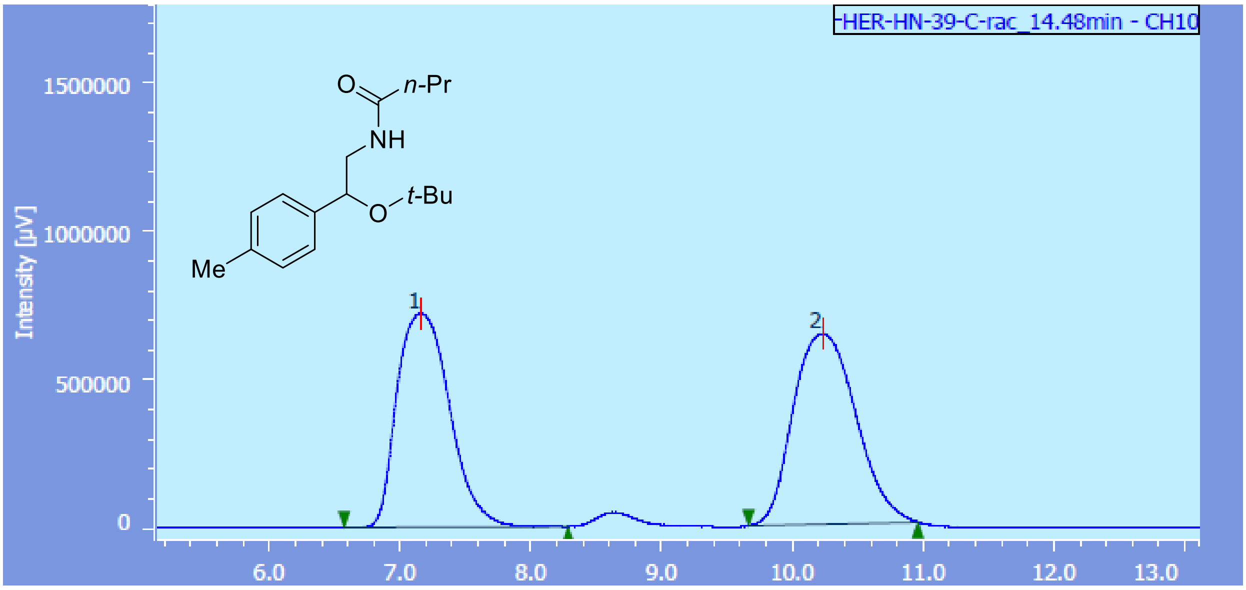

#	Peak Name	CH	tR [min]	Area [µV·sec]	Height [µV]	Area%	Height%	Quantity	NTP	Resolution	Symmetry Factor	Warning
1	Unknown	10	7.160	20147397	718434	48.490	52.832	N/A	1419	3.683	1.291	
2	Unknown	10	10.230	21402296	641424	51.510	47.168	N/A	2016	N/A	1.176	

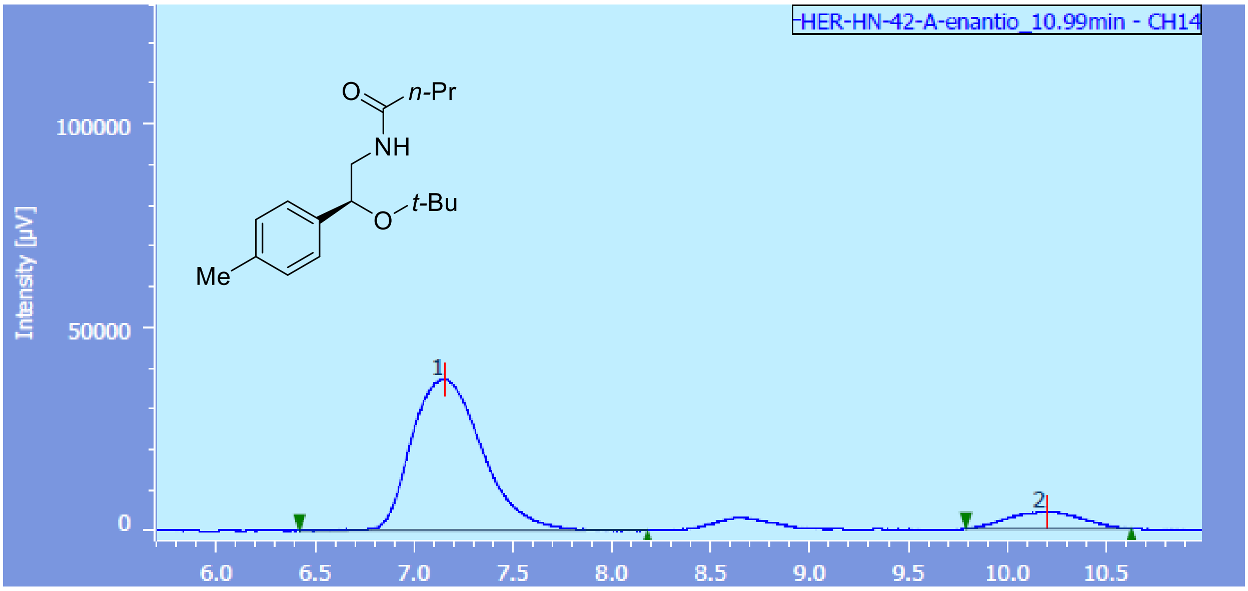

#	Peak Name	CH	tR [min]	Area [µV·sec]	Height [µV]	Area%	Height%	Quantity	NTP	Resolution	Symmetry Factor	Warning
1	Unknown	14	7.153	921600	37164	89.652	90.198	N/A	1875	4.350	1.290	
2	Unknown	14	10.197	106380	4038	10.348	9.802	N/A	3019	N/A	1.019	

(*S*)-*N*-[2-Phenoxy-2-(*p*-tolyl)ethyl]butyramide (2.37)

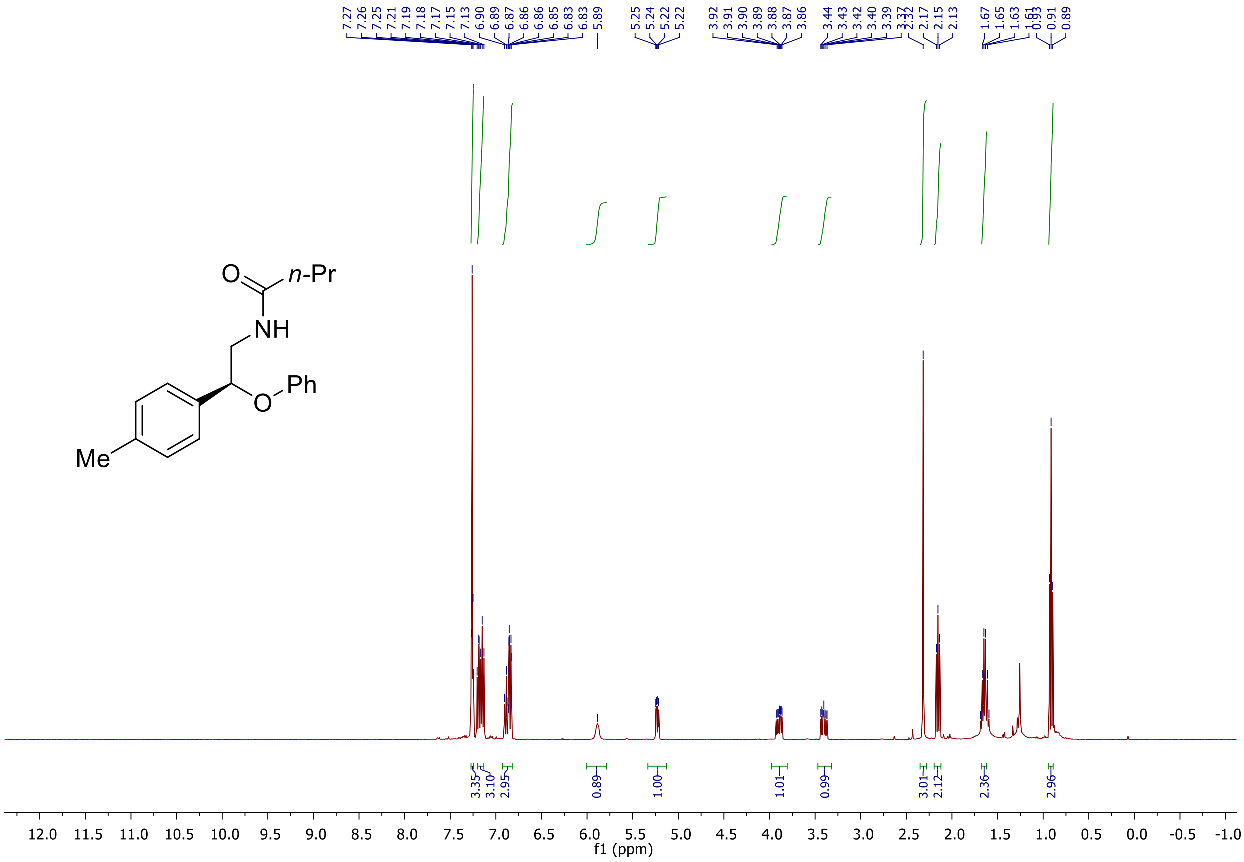

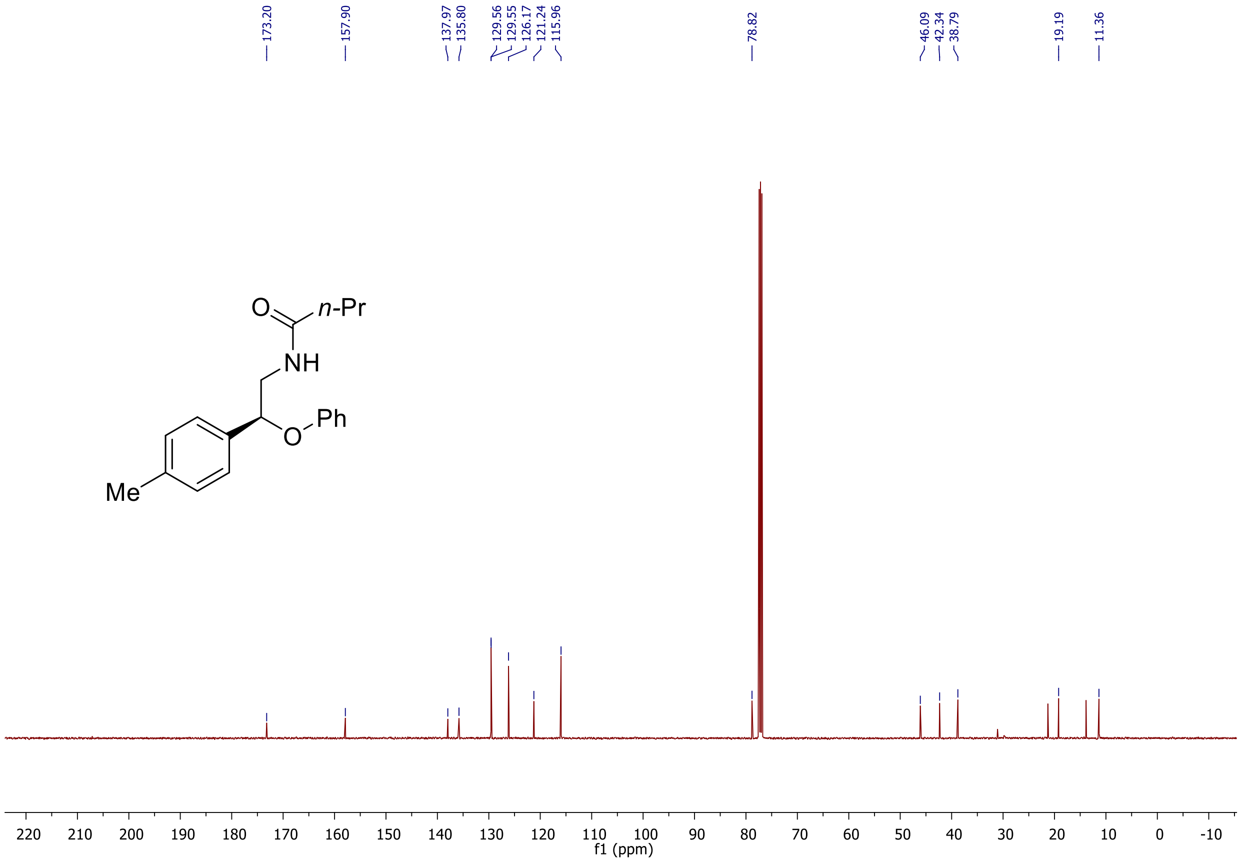

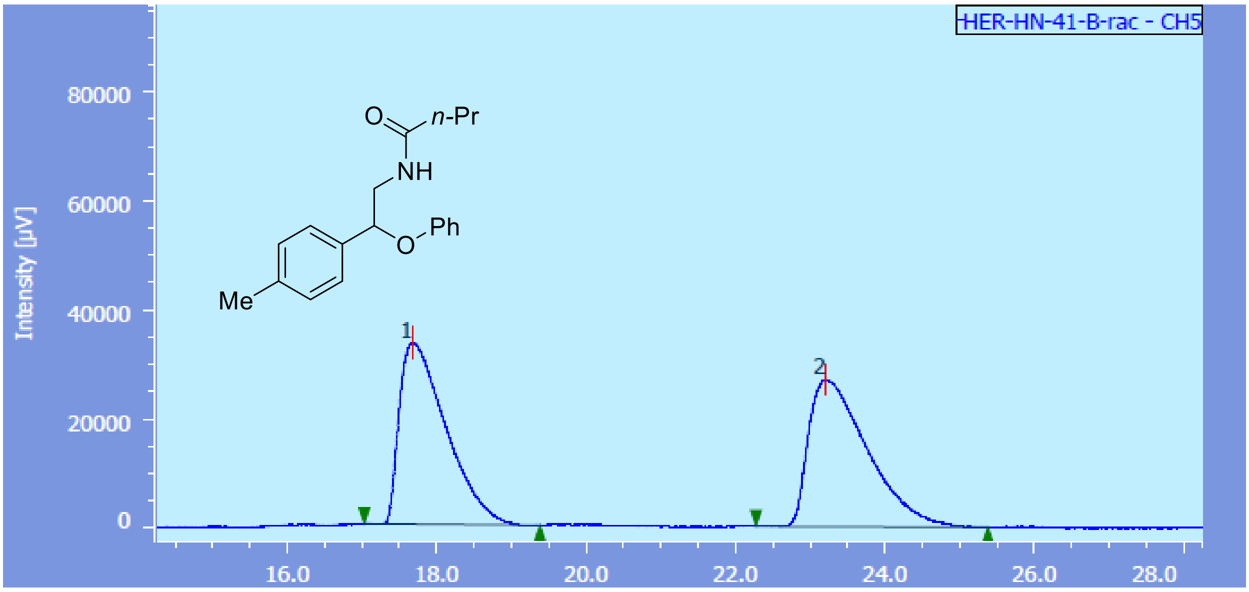

#	Peak Name	CH	tR [min]	Area [µV·sec]	Height [µV]	Area%	Height%	Quantity	NTP	Resolution	Symmetry Factor	Warning
1	Unknown	5	17.683	1443362	33298	49.469	55.306	N/A	3737	4.259	2.164	
2	Unknown	5	23.210	1474371	26908	50.531	44.694	N/A	4126	N/A	2.134	

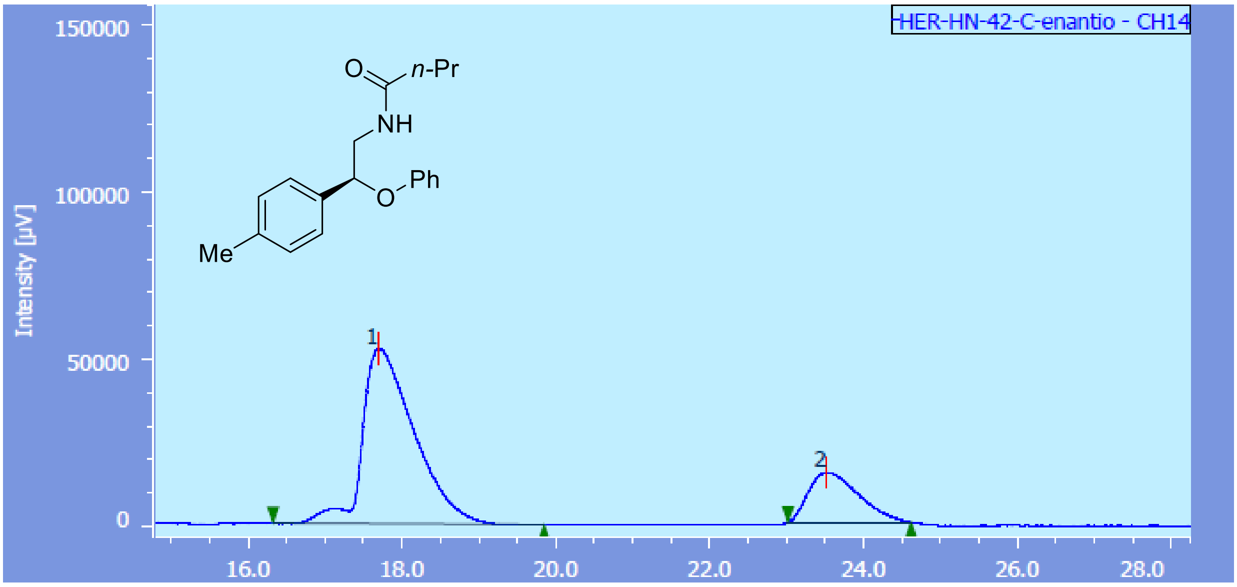

#	Peak Name	CH	tR [min]	Area [µV·sec]	Height [µV]	Area%	Height%	Quantity	NTP	Resolution	Symmetry Factor	Warning
1	Unknown	14	17.700	2460688	52349	78.485	77.797	N/A	3586	4.836	1.213	
2	Unknown	14	23.520	674559	14941	21.515	22.203	N/A	5839	N/A	1.552	

(*S*)-*N*-[2-Methoxy-2-(*p*-tolyl)propyl]butyramide (2.38)

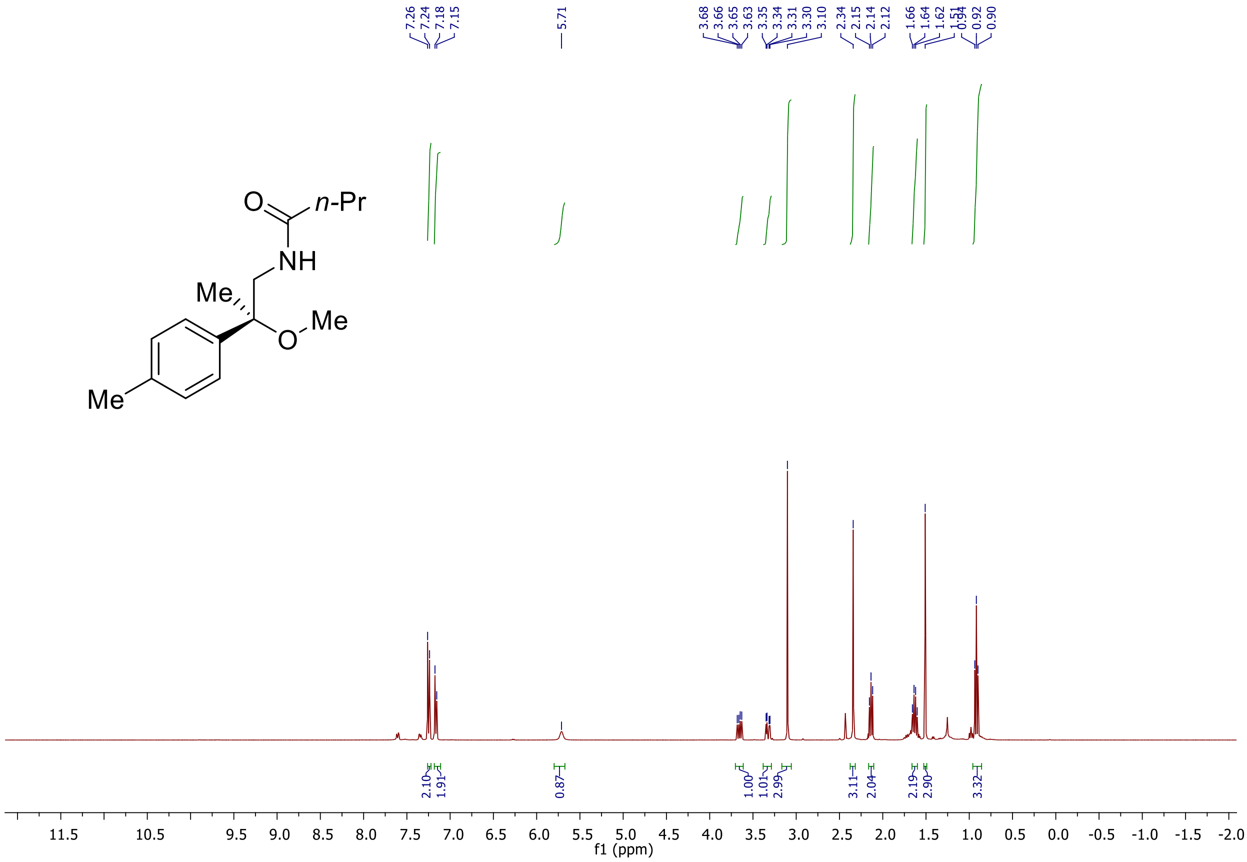

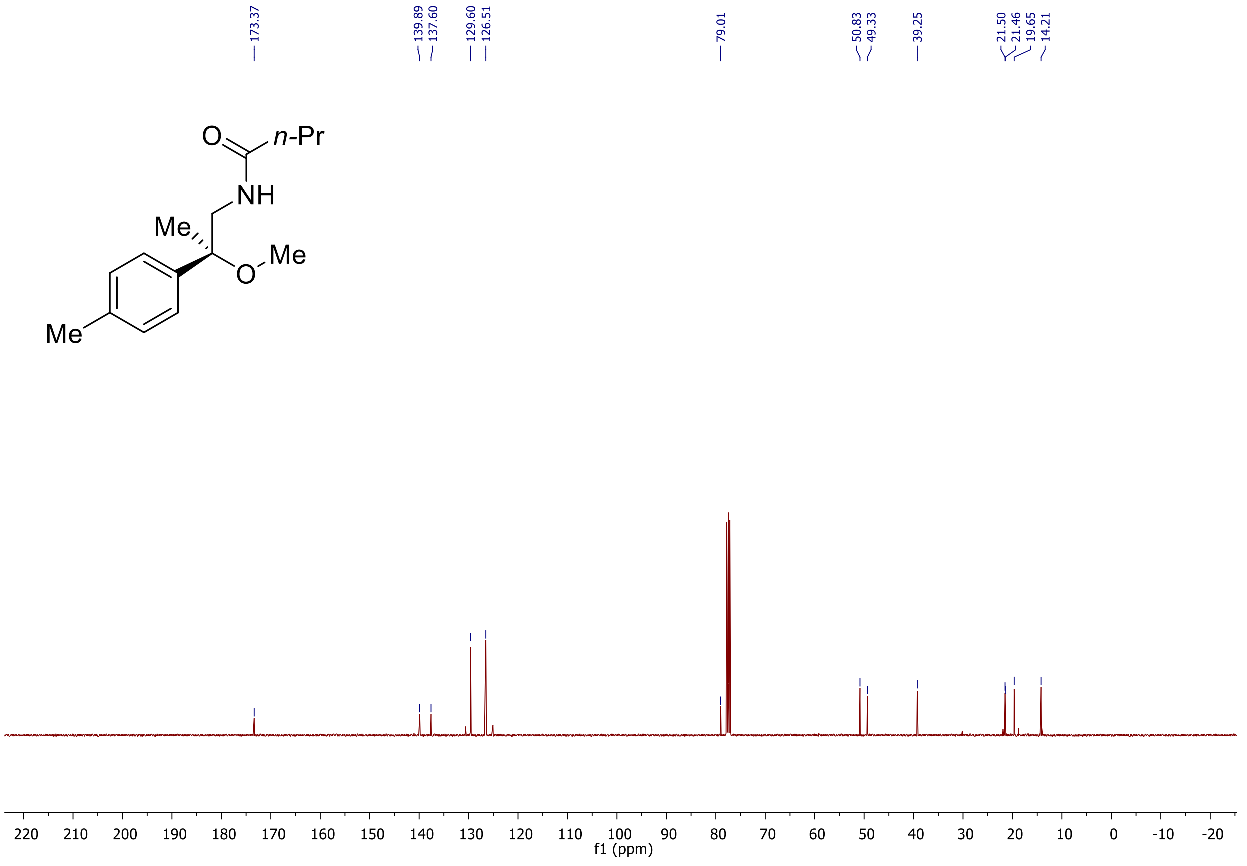

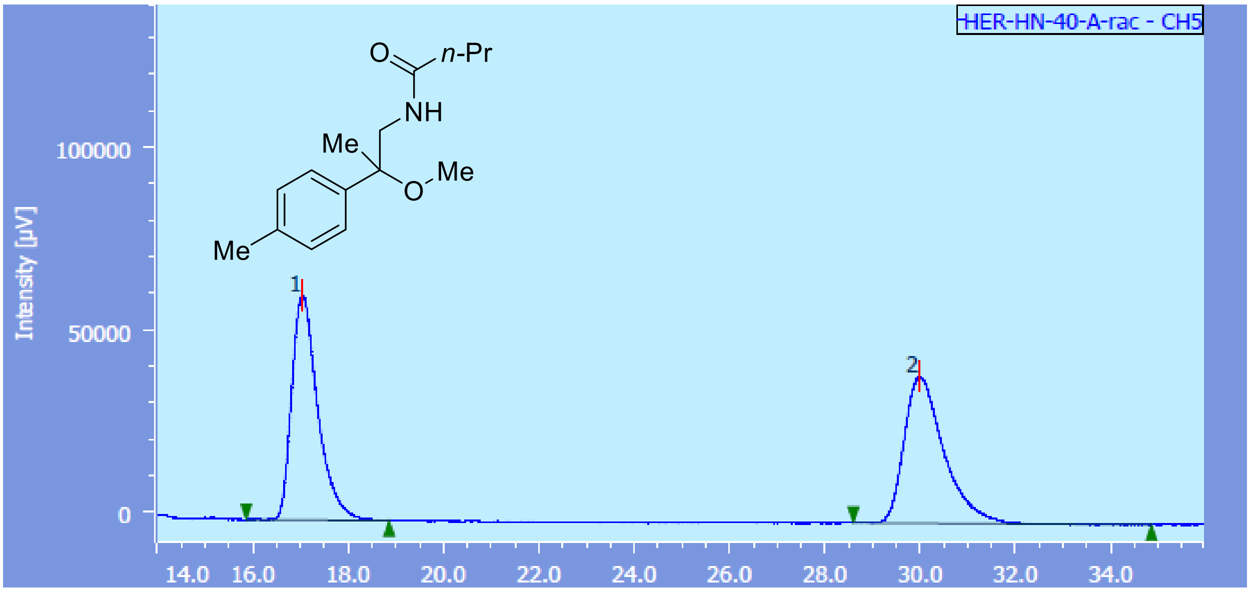

#	Peak Name	CH	tR [min]	Area [µV·sec]	Height [µV]	Area%	Height%	Quantity	NTP	Resolution	Symmetry Factor	Warning
1	Unknown	5	17.040	2331742	61536	50.107	60.505	N/A	5046	10.752	1.481	
2	Unknown	5	29.977	2321759	40168	49.893	39.495	N/A	6808	N/A	1.571	

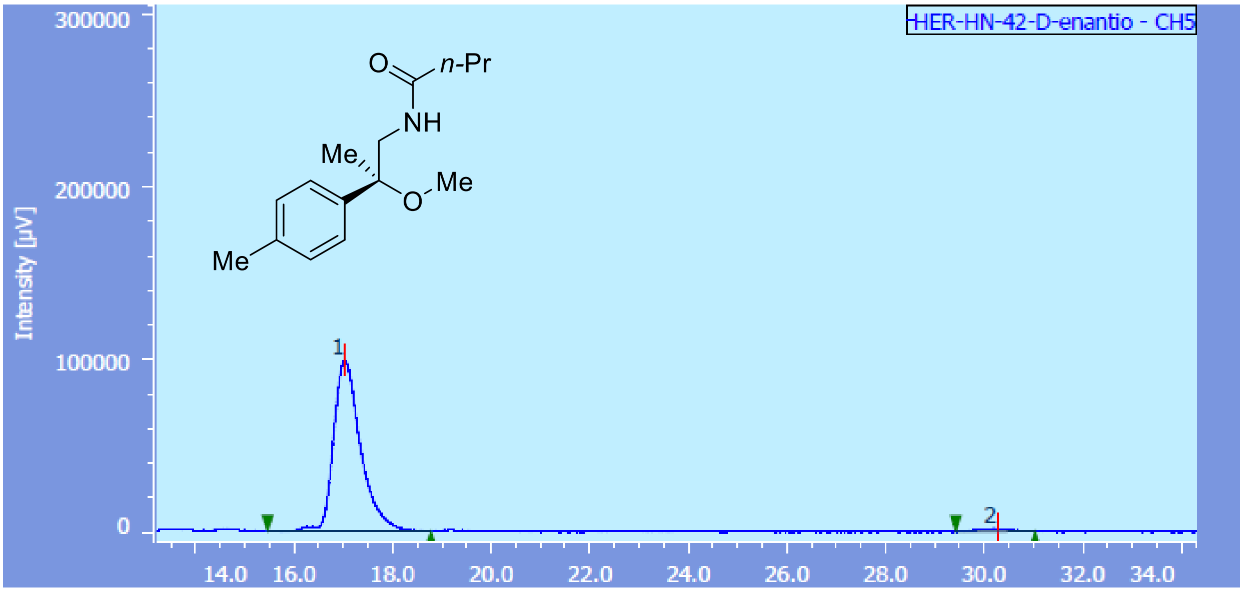

#	Peak Name	CH	tR [min]	Area [µV·sec]	Height [µV]	Area%	Height%	Quantity	NTP	Resolution	Symmetry Factor	Warning
1	Unknown	5	17.030	3771208	98875	98.137	98.453	N/A	5196	11.673	1.481	
2	Unknown	5	30.257	71572	1554	1.863	1.547	N/A	8317	N/A	0.973	

(1*R*,2*R*)-1-(4-Methoxyphenyl)-1-(*p*-tolyl)propan-2-amine (2.39)

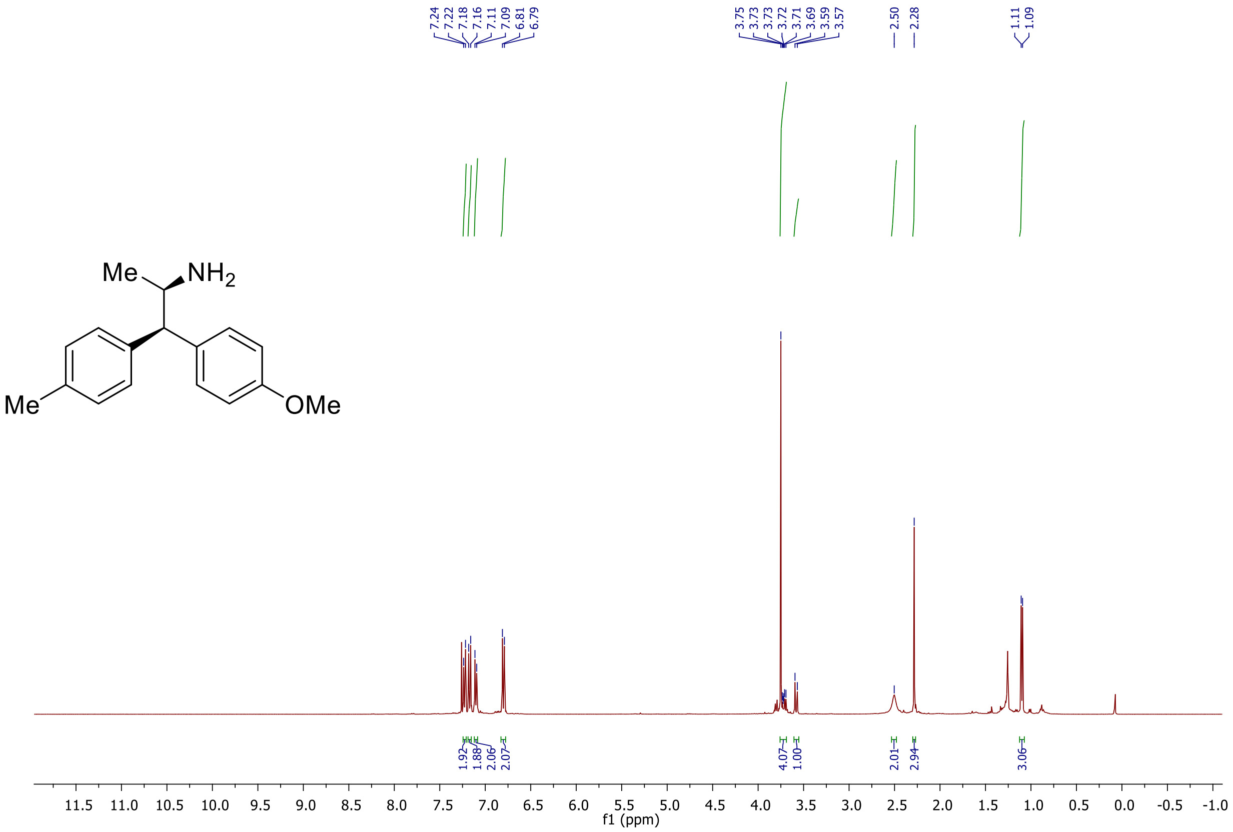

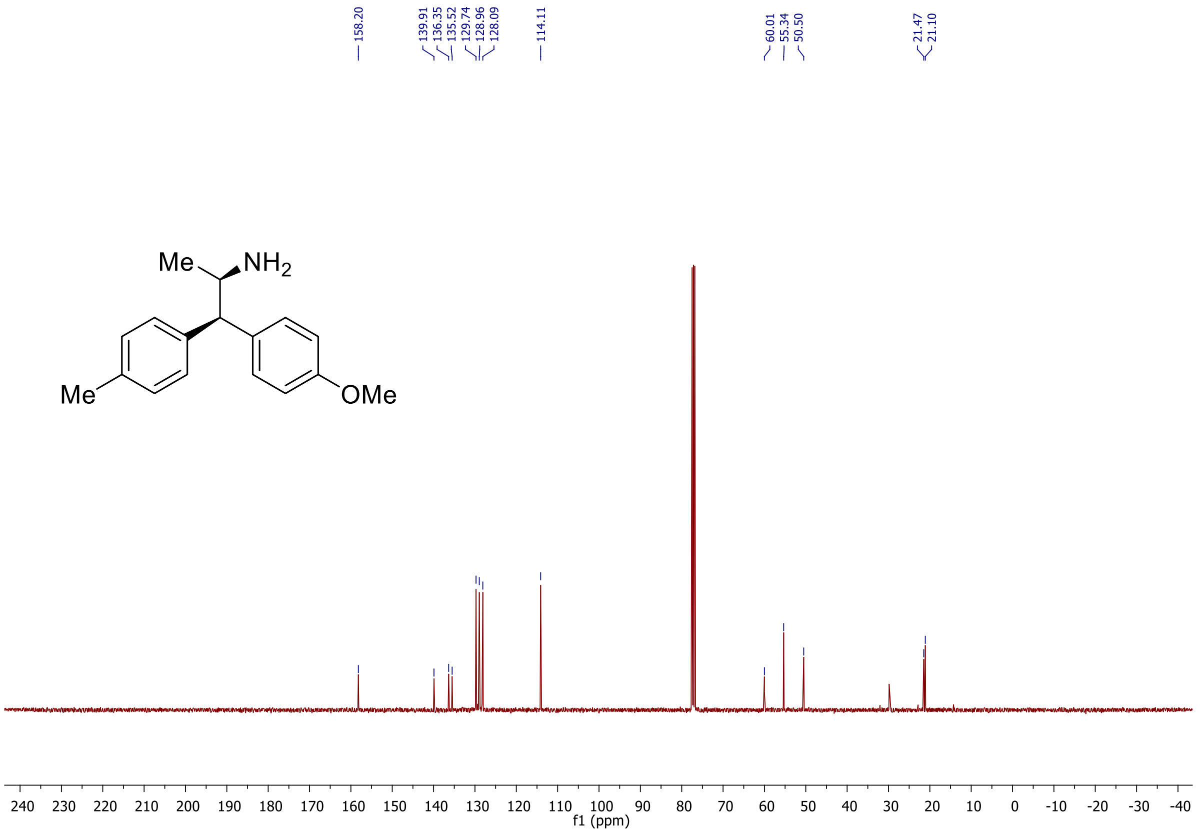

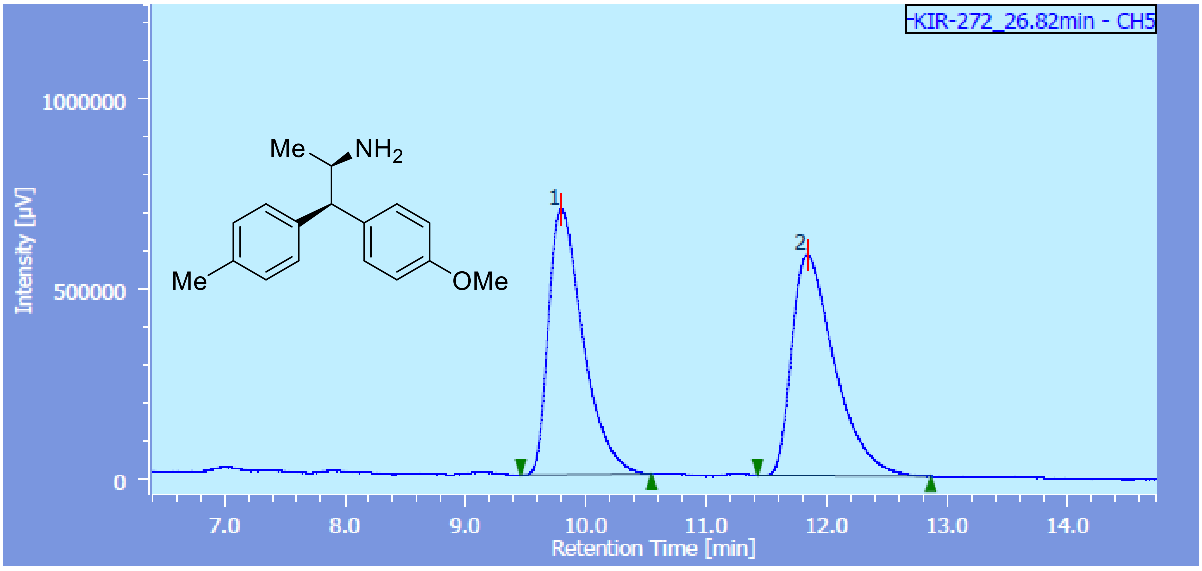

#	Peak Name	CH	tR [min]	Area [μV·sec]	Height [μV]	Area%	Height%	Quantity	NTP	Resolution	Symmetry Factor	Warning
1	Unknown	5	9.797	13972505	699119	49.300	54.657	N/A	5895	3.573	1.650	
2	Unknown	5	11.837	14369028	579987	50.700	45.343	N/A	5567	N/A	1.715	

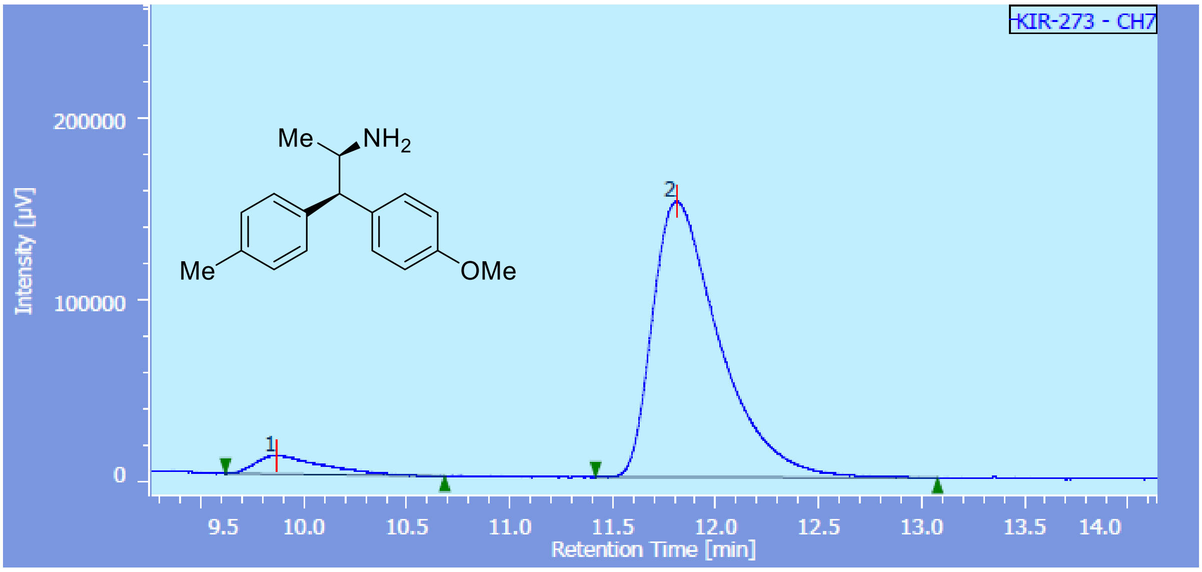

#	Peak Name	CH	tR [min]	Area [μV·sec]	Height [μV]	Area%	Height%	Quantity	NTP	Resolution	Symmetry Factor	Warning
1	Unknown	7	9.870	231488	10293	6.293	6.344	N/A	4501	3.356	1.843	
2	Unknown	7	11.810	3446922	151961	93.707	93.656	N/A	6847	N/A	1.739	

1-[(2*R*,3*S*)-3-(4-Methoxyphenyl)-2-methylindolin-1-yl]butan-1-one (2.40)

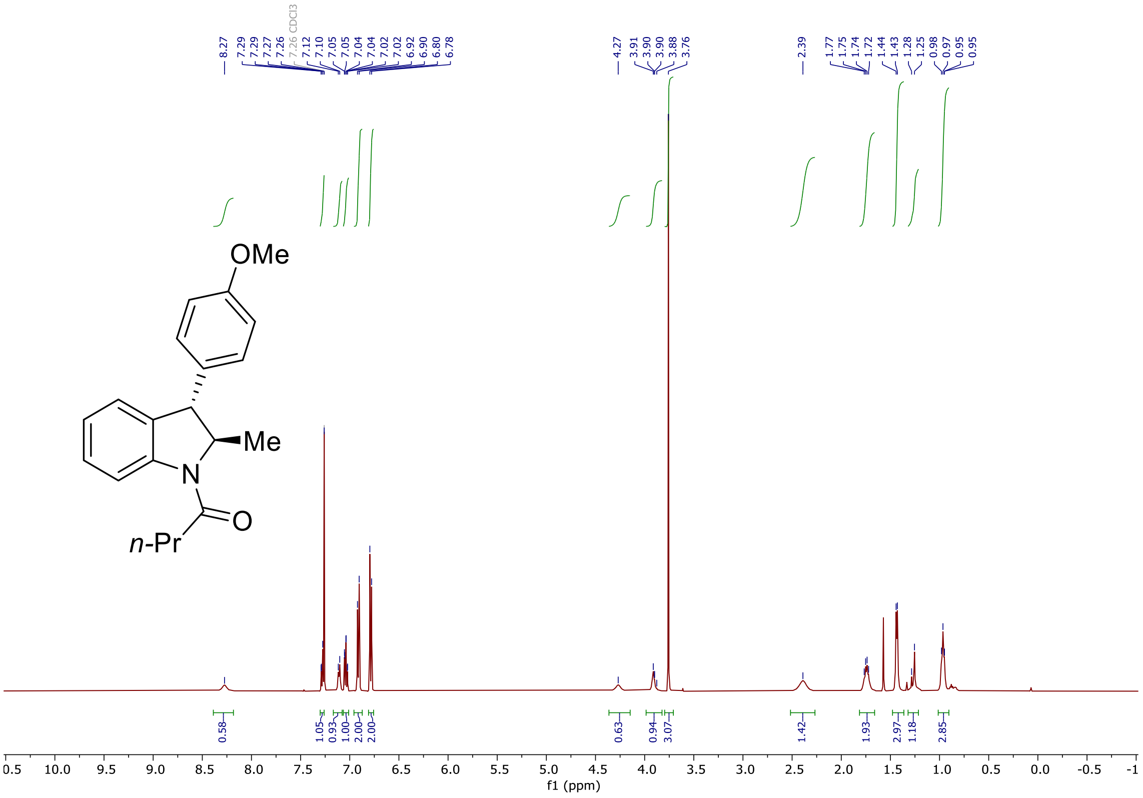

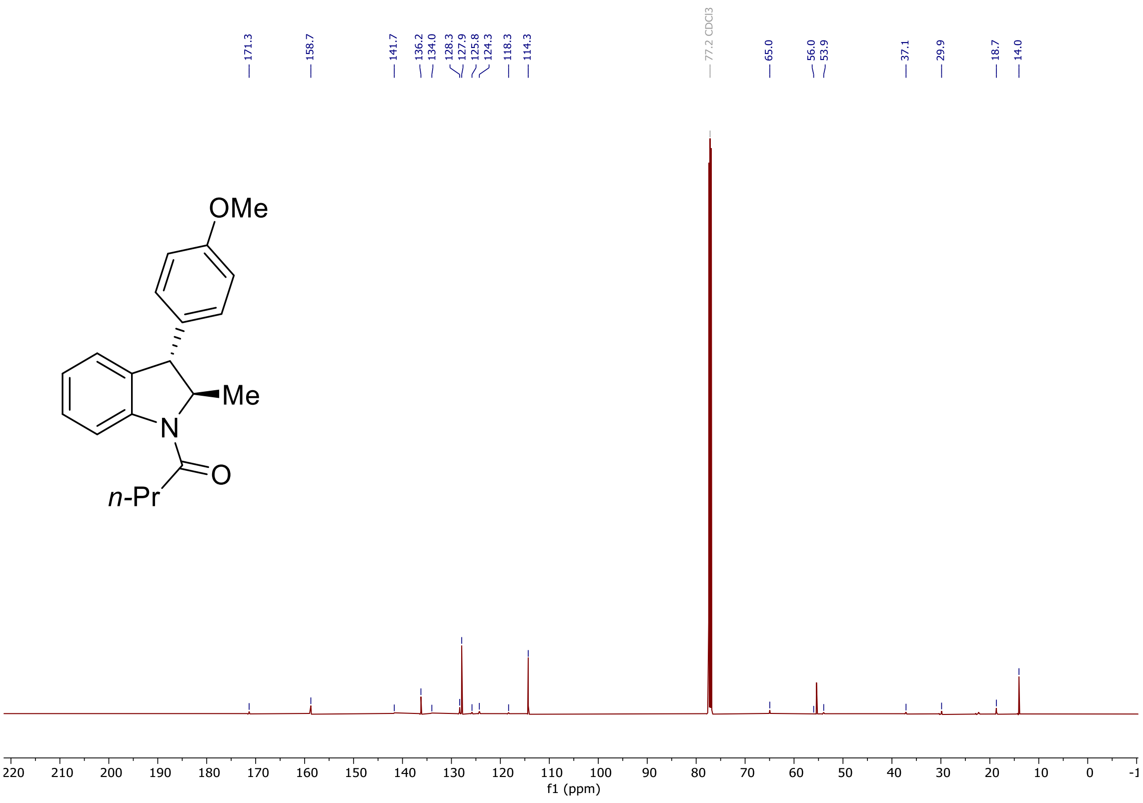

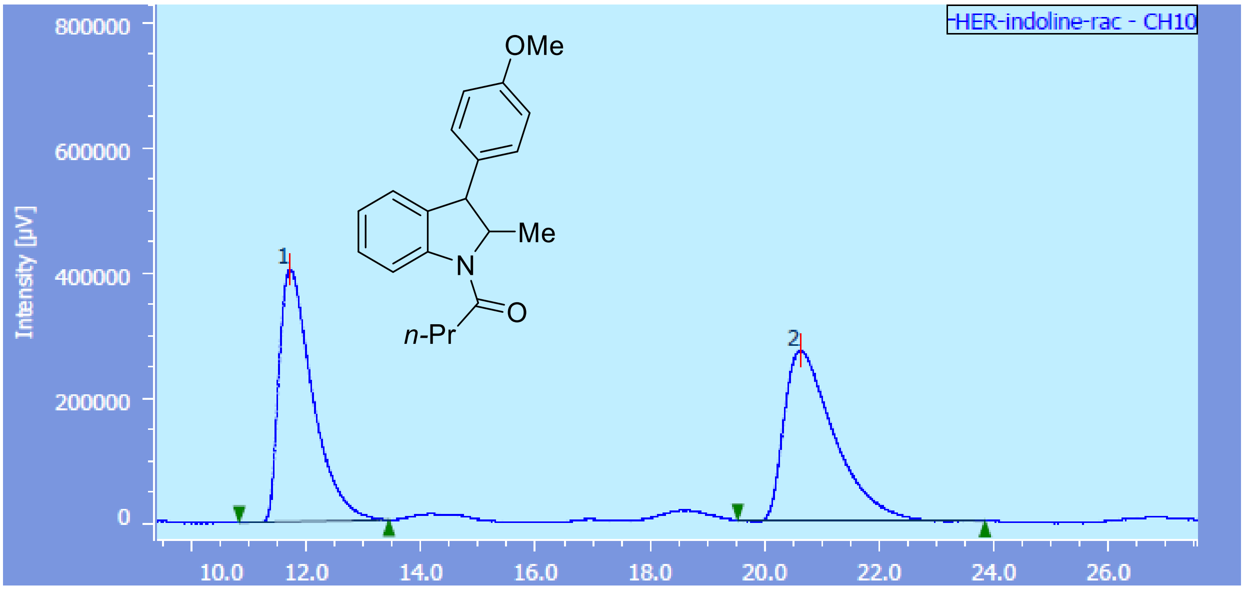

#	Peak Name	CH	tR [min]	Area [µV·sec]	Height [µV]	Area%	Height%	Quantity	NTP	Resolution	Symmetry Factor	Warning
1	Unknown	10	11.717	16385297	402893	49.997	59.755	N/A	2049	6.915	1.987	
2	Unknown	10	20.620	16387589	271352	50.003	40.245	N/A	2844	N/A	1.966	

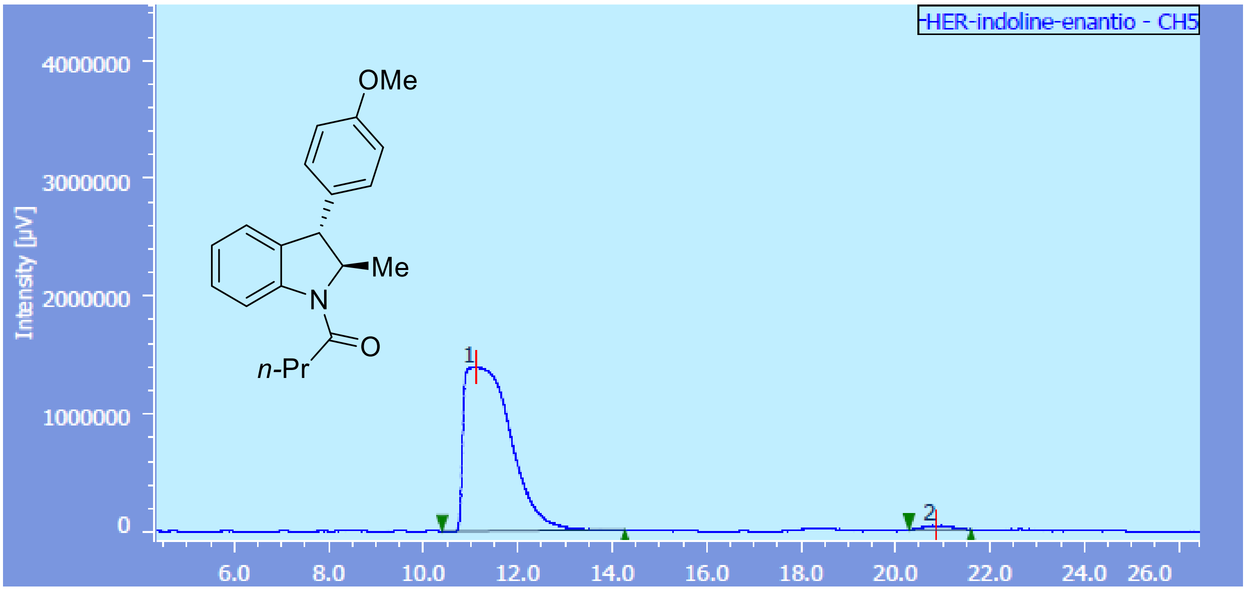

#	Peak Name	CH	tR [min]	Area [µV·sec]	Height [µV]	Area%	Height%	Quantity	NTP	Resolution	Symmetry Factor	Warning
1	Unknown	5	11.110	95405164	1386142	98.479	97.594	N/A	577	6.370	2.709	
2	Unknown	5	20.860	1473507	34176	1.521	2.406	N/A	4681	N/A	1.139	

DFT calculations

All reported structures were optimized at Density Functional Theory level by using the M062x functional[5] as implemented in Gaussian 16.[6] Optimizations were carried out by using the 6-31+G(d,p) basis set for all atoms, in a solvent model[7] (IEFPCM, solvent = 2-propanol) at 233.15 K. Reported energy values correspond to Gibbs Free (G) energies in kcal·mol^{-1}. All structures were optimized without geometrical constraint. Stationary points were characterized by frequency calculations (no negative frequency for minima and one negative frequency for transition states).

Supplementary Table 3. Computed absolute electronic energies and relative free Gibbs energies (Hartrees) for all structures, and imaginary frequencies for the transition states. Ar = 4-OMe-C_6H_4.

	E (M062X)	Correction to G (M062X)	G (M062X)	Freq
$[Ir[(dFCF_3)ppy]_2(dtbpy)]^+$	-2942,00423901	0,620261	-2941,383978	-
$[Ir[(dFCF_3)ppy]_2(dtbpy)]^\bullet$	-2942,1160976	0,616089	-2941,500008	-
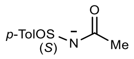	-952.237023879	0.141241	-952.095783	-
**II**	-463.094707195	0.165635	-462.929073	-
**TS_{I-III} (S_S,*R*)**	-1415,35604529	0,327238	-1415,028807	-520,4418
**TS_{I-III} (S_S,*S*)**	-1415.35412344	0.327418	-1415,026706	-431.4252
**III**	-1415,40082841	0,331849	-1415,06898	-
**III´**	-1415.39152727	0.332312	-1415.059216	-
**TS_{III-IV}**	-1415,38180244	0,332326	-1415,049477	-500,3337
**IV**	-1415,44302483	0,332163	-1415,110861	-
**V**	-942,750378482	0,34303	-942,407349	-
**VI**	-1415,5852618	0,33453	-1415,250732	
SO	-473,249085777	-0,014493	-473,263578	-
$PhCO_2H$	-420,681672201	0,090264	-420,591409	-
$PhCO_2K$	-1020,06103253	0,074613	-1019,986419	-
K^+	-599,821088514	-0,012558	-599,833646	-

Calculation of reduction potential of **IV** and **VI** following the procedure described in *Synlett* **2016**, *27*, 714-723:

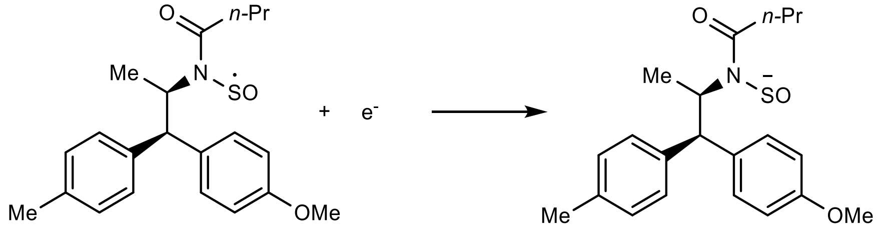

IV **VI**

$$E_{1/2}^{o,calc} = -\frac{(G_{298}[III] - G_{298}[III\text{-}a])}{n_eF} - E_{1/2}^{o,SHE} + E_{1/2}^{o,SCE}$$

n_e = number of electrons transferred

F = Faraday constant (value = 23.061 kcal·mol^{-1}·V^{-1})

$E^{\circ,SHE}_{1/2}$ = Absolute value for the standard hydrogen electrode (SHE, value = 4.281 V)

$E^{\circ,SCE}_{1/2}$ = Potential of the saturated calomel electrode (SCE) relative to SHE in acetonitrile (value = -0.141 V)

G_{298}[**IV**] and G_{298}[**V**] are the Gibbs free energies in 2-propanol from DFT calculations

G_{298} [**IV**] = -1415,110861 Hartrees

G_{298} [**V**] = -1415,250732 Hartrees

$\Delta G^{\circ}_{1/2}$ = (G_{298} [**V**] - G_{298} [**IV**]) = (-1415,250732 + 1415,110861) Hartree * 627.51 kcal·mol^{-1} = -87.77 kcal·mol^{-1}

$$E^{\circ,calc}_{1/2} = -\frac{\Delta G^{\circ}_{1/2}}{n_e F} - E^{\circ,SHE}_{1/2} + E^{\circ,SCE}_{1/2} = -\frac{-86.58\ \text{kcal}\bullet\text{mol}^{-1}}{23.061\ \text{kcal}\bullet\text{mol}^{-1}\ \text{V}^{-1}} - 4.281\ \text{V} - 0.141\ \text{V} = -0.62\ \text{V vs. SCE}$$

References

1. Zhu, R. H. & Shi. X. X., Practical and highly stereoselective method for the preparation of several chiral arylsulfinamides and arylsulfinates based on the spontaneous crystallization of diastereomerically pure *N*-benzyl-*N*-(1-phenylethyl)-arylsulfinamides. *Tetrahedron: Asymmetry* **22,** 387–393, (2011).
2. Antonioletti, R., Bonadie, F., Ciammaichella, A. & Viglianti, A., Lithium hydroxide as base in the Wittig reaction. A simple method for olefin synthesis. *Tetrahedron*, **64**, 4644-4648 (2008).
3. Lin, Q., Dawson, G. & Diao, T., Experimental Electrochemical Potentials of Nickel Complexes. *Synlett.* ***32***, 1606–1620, (2021).
4. Kou. M., Wei. Z., Li. Z. & Xu. B. Copper-catalyzed sulfinyl cross-coupling reaction of sulfinamides. *Org. Lett.*, **46**, 8514–8519 (2022).

5. Zhao, Y. & Truhlar, D. G., The M06 suite of density functionals for main group thermochemistry, thermochemical kinetics, noncovalent interactions, excited states, and transition elements: two new functionals and systematic testing of four M06-class functionals and 12 other functionals. *Theor. Chem. Acc.* **120**, 215-241, (2008).

6. Gaussian 16, Revision C.01; Frisch, M. J. et al. Gaussian, Inc., Wallingford CT, 2016.

7. (a) Cancès, E., Mennucci, B., & Tomasi, J., A new integral equation formalism for the polarizable continuum model: Theoretical background and applications to isotropic and anisotropic dielectrics. *J. Chem. Phys.* **107**, 3032–3047, (1997). (b) Cossi, M., Barone, V., Mennuci, B., & Tomasi, J. Ab initio study of ionic solutions by a polarizable continuum dielectric model. *Chem. Phys. Lett.*, **286**, 253–260, (1998). (c) Tomasi, J. & Mennucci, B. E. Cancès, The IEF version of the PCM solvation method: an overview of a new method addressed to study molecular solutes at the QM ab initio level. *J. Mol. Struct. (Theochem)*, **464**, 211–226, (1999).
